# 3-sulfonyloxyaryl(mesityl)iodonium triflates as 1,2-benzdiyne precursors with activation via *ortho*-deprotonative elimination strategy

**DOI:** 10.1038/s41467-023-37196-3

**Published:** 2023-04-03

**Authors:** Haoyin Yuan, Wenhao Yin, Jili Hu, Yang Li

**Affiliations:** 1 School of Chemistry and Chemical Engineering, Chongqing University, 174 Shazheng Street, Chongqing, 400030 China; 2 College of Chemistry, Jilin University, Changchun, 130012 China

**Keywords:** Synthetic chemistry methodology, Reactive precursors

## Abstract

Benzyne has long captivated the attention of chemists and has gained numerous synthetic achievements. Among typical benzyne generation methods, removal of two vicinal substituents from 1,2-difunctionalized benzenes, i.e., Kobayashi’s protocol, are prevailing, while *ortho*-deprotonative elimination from mono-substituted benzene lags far behind. Despite the advantages of atom economy and ready achievability of precursors, a bottle neck for *ortho*-deprotonative elimination strategy resides in the weak acidity of the *ortho*-hydrogen, which normally demands strong bases as the activating reagents. Here, an efficient aryne generation protocol is developed, where *ortho*-deprotonative elimination on 3-sulfonyloxyaryl(mesityl)iodonium triflates occurs under mild conditions and the generated 3-sulfonyloxyarynes can serve as efficient 1,2-benzdiyne synthons. This array of 1,2-benzdiyne precursors can be conveniently prepared with high functional group tolerance, and densely substituted scaffolds can be accessed as well. Carbonate and fluoride salts are found to serve as efficient activating reagents, which are the weakest bases used in *ortho*-deprotonative elimination strategies. Particularly, this scaffold has predictable chemoselective generation of the designated aryne intermediates. The success of this *ortho*-deprotonative elimination protocol sets up a unique platform with a broad spectrum of synthetic applications.

## Abstract (web summary)

Preparation of functionalized analogues of 1,2-benzdiyne, from heavily substituted as well as benzofused scaffolds, is challenging. Here, the authors develop an array of 3-sulfonyloxyaryl(mesityl)iodonium triflates as 1,2-benzdiyne precursors, triggered from ortho-deprotonative elimination.

## Introduction

A distinct property of benzyne chemistry is the ready assembly of two vicinal chemical bonds in step-economical manner^1–3^. Along with the extensive application of mild generation methods in the past two decades, i.e., Kobayashi’s protocol^4^ and via hexadehydro-Diels-Alder (HDDA) reactions^5^, numerous benzyne transformations have been unraveled^5–20^. Due to the fleeting character of a benzyne species, however, it has to be generated in situ from a precursor and, preferentially, via a slow releasing manner. Consequently, both the constitution of a benzyne precursor and its generation conditions are essential for the success of a benzyne-engaged transformation. In this context, efforts to explore new generation methods have been accompanied with the development of benzyne chemistry. To date, there are two major strategies to generate benzyne, either removal of two vicinal substituents from 1,2-difunctionalized benzene or *ortho*-deprotonative elimination from mono-substituted benzene. Between these two strategies, *ortho*-disubstituted benzyne precursors have been recognized as the most popular ones, primarily due to their ease to be activated and the accommodation of the generated benzyne to various types of reactions. For instance, *o*-silylaryl triflates (Kobayashi’s aryne precursors) have been the most utilized precursors in the past two decades and are accounted for the recent renaissance of benzyne chemistry^20^.

**Fig. 1:**
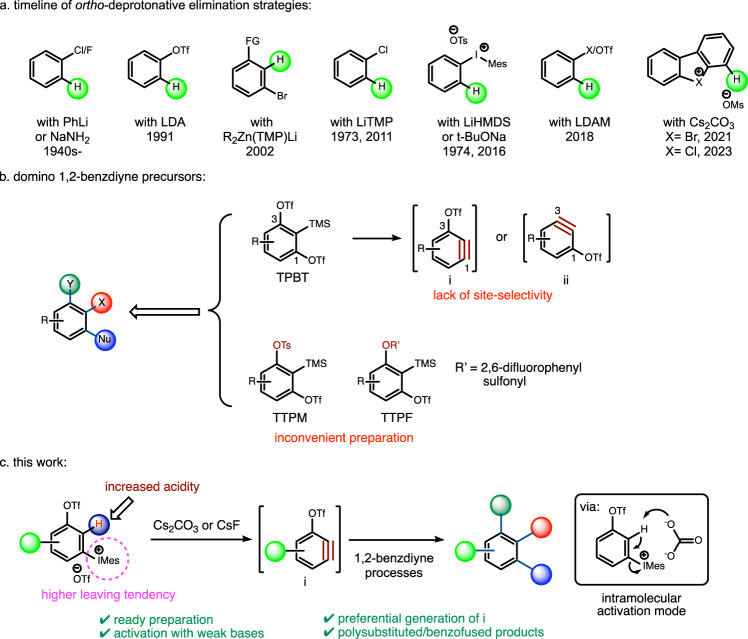
Background and our work. **a** Timeline of *ortho*-deprotonative elimination protocols. **b** Problems associated with the application of previous domino 1,2-benzdiyne precursors. TPBT: 2-(trimethylsilyl)−1,3-phenylene bis(trifluoromethanesulfonate). TTPM: 3-(((trifluoromethyl)sulfonyl)oxy)−2-(trimethylsilyl)phenyl 4-methylbenzenesulfonate. TTPF: 3-(((trifluoromethyl)sulfonyl)oxy)−2-(trimethylsilyl)phenyl 2,6-difluorobenzenesulfonate. **c** Our work.

Despite the facts that *ortho*-deprotonative elimination strategy was first used in the early era of benzyne chemistry and possesses its merit by removing only one leaving group (LG) along with deprotonation, it is now lagging far behind of the current prevailing strategy employing 1,2-disubstituted benzyne precursors. Presumably, a marked obstacle for *ortho*-deprotonative elimination strategy is the weak acidity of the *ortho*-proton of typical leaving groups, which remains to be a challenge in benzyne chemistry. Since 1940s, strong bases, such as phenyllithium and NaNH_2_, have been employed to deprotonate halobenzenes (Fig. 1a)^20^. In 1991, phenyl triflate was used as the precursor, where lithium diisopropylamide (LDA) served as both the activating reagent and nucleophile^21^. To avoid unwanted nucleophilic addition by the base, lithium 2,2,6,6-tetramethylpiperidide (LiTMP)^22–26^, lithium diadamantylamide (LDAM)^27^, and lithium di-alkyl(2,2,6,6-tetramethylpiperidino)zincate (R_2_Zn(TMP)Li) were successfully developed^28,29^. Alternatively, diaryliodonium salts were reported first by Akiyama in 1974^30^ and recently revisited by Stuart^31–34^ to serve as aryne precursors. The advantage for this generation method is based on the fact that the hypernucleofugality of aryliodonio group is million-time better than that of OTf group^35^. However, those strong bases and organometallic reagents used in the aryne generation step fall short of functional group compatibility, which also limit the discovery of benzyne transformations. Very recently, cyclic diaryl λ^3^-bromanes/λ^3^-chloranes were found to serve as aryne precursors, where Cs_2_CO_3_ could efficiently deprotonate the *ortho*-hydrogen to generate the corresponding arynes due to the better leaving tendency of *λ*^3^-bromane/*λ*^3^-chloranes than that of hypervalent iodide^36–38^. The mechanistic study also revealed that deprotonation and C-X bond cleavage should be a concerted process via seven-membered cyclic transition state involving carbonate anion^38^. Although promising, this method is limited to cyclic *λ*^3^-bromanes and only bromo-biaryl compounds were obtained, where the application of acyclic *λ*^3^-bromanes was restricted due to the high reactivity of BrF_3_ that is normally used to prepare acyclic *λ*^3^-bromanes through ligand exchange^39^.

In the past a few years, we spent our effort on 1,2-benzdiyne transformations, where polysubstituted arenes containing biologically active frameworks could be conveniently prepared either via tandem processes or in stepwise manner^18,40–47^. A series of domino aryne precursors based on 2-silylaryl 1,3-bis(sulfonate) framework were developed along with our study (Fig. 1b). Although possessing promising potential, the employment of Kobayashi’s benzyne generation method in our 1,2-benzdiyne system lacks functional group economy. Moreover, two notable restrictions exist to prevent this structural motif from reaching unsymmetrically substituted precursors and/or reaction products. On one hand, it cannot manipulate site-selective generation of 4- or 6-substituted 3-triflyloxybenzyne (intermediate **i** vs **ii**, Fig. 1b) from TPBT-based framework^43^. On the other hand, it is hard to access either 4- or 6-functionalized TTPM/TTPF-based scaffold from unsymmetrically substituted resorcinols due to limited sources of feedstocks as well as the lack of practical method to differentiate two OH groups on them. As a result, only several substituted TPBTs and TTPMs were reported so far^18^. In this context, an emerging need is to judiciously redesign structures of 1,2-benzdiyne equivalents so that a broad range of precursors could be conveniently accessed and a variety of densely substituted arenes bearing potential biological properties can be conceived after 1,2-benzdiyne transformations.

We envisioned that those building blocks that could utilize *ortho*-deprotonative elimination strategy to trigger 1,2-benzdiyne transformations under mild conditions would be more appropriate and practical candidates. To this end, we proposed the following criteria for 1,2-benzdiyne equivalents: (1) mild activation conditions, i.e., with weak base and activation temperature ranging from 0 ^o^C to 100 ^o^C, via *ortho*-deprotonative elimination strategy that possess the advantage of functional group economy; (2) ready preparation for substituted analogues; (3) site-selective generation of the designated aryne upon activation. In our previous studies on 1,2-benzdiyne transformations, carbonate salts were found to efficiently kick out the trimethylsilyl (TMS) group on those precursors in Fig. 1b, the weakened C(aryl)-Si bond of which should be the result of the *σ*-electron-withdrawing (*σ*-EW) effect of two *ortho*-sulfonate groups. Moreover, both Tilley^48^ and Stuart^31–34^ observed the site-selective generation of arynes via *ortho*-deprotonative elimination pathways, which are consistent with the calculation on proximity-induced acidity of C2-hydrogen^49^. In this context, we postulated that as long as the sum of *σ*-EW effect is large enough, the acidity of this C2-H might eventually reach the level to be deprotonated by weak bases. Since many *σ*-EW groups are LGs as well, 1,2-benzdiyne equivalents would be conceived.

Herein, we wish to report our discovery on a mild *ortho*-deprotonative elimination protocol to initiate 1,2-benzdiyne transformations from 3-sulfonyloxyaryl(mesityl)iodonium triflates, in which carbonate and even fluoride salts could serve as efficient activating reagents (Fig. 1c). This scaffold has a substantially expanded precursor scope with preferential generation of substituted 3-sulfonyloxyarynes due to the superb leaving tendency of the mesityliodonio group.

## Results and Discussion

### Initial tests

**Fig. 2:**
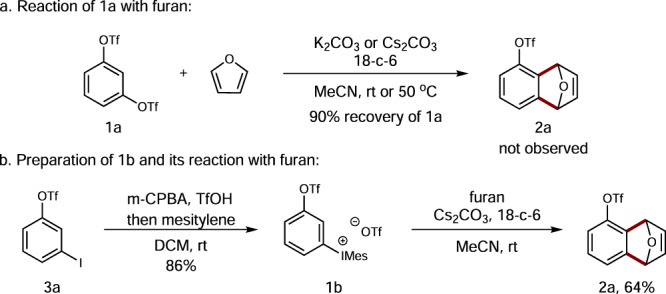
Initial studies. **a** The reaction between **1a** and furan in the presence of carbonate salts furnished no desired cycloadduct **2a**. **b** The preparation of **1b** and its reaction with furan in the presence of Cs_2_CO_3_ gave rise to cycloadduct **2a** in 64% yield.

To probe our hypothesis, we first examined 1,3-phenylene bis(trifluoromethanesulfonate) (**1a**) in order to see whether 3-triflyloxybenzyne **i** could be generated in the presence of carbonate (Fig. 2a). Unfortunately, no [4 + 2]-cycloadduct **2a** with furan was detected in this reaction and 90% of **1a** was recovered, suggesting that its C2-hydrogen is not acidic enough. Inspired by the observation that aryliodonio group is a much better LG than OTf^35,50^, we decided to prepare and examine compound **1b** by introducing a mesityliodonium salt at the *meta*-position of the OTf group (Fig. 2b). Expectedly, the enhanced acidity of the C2-H in response to the presence of two proximal strong *σ*-EWGs, namely an OTf and an aryliodonio group, could eventually allow its deprotonation by carbonates. As shown in eq 2, the preparation of compound **1b** followed a one-pot oxidation protocol from 3-iodophenyl triflate (**3a**)^51^. Upon activation with Cs_2_CO_3_/18-c-6 in acetonitrile, gratifyingly, **2a** was obtained in 64% yield.

### Reaction optimization

Encouraged by this preliminary result, we decided to optimize this transformation. By varying solvents, DCM was found to be a comparable one with acetonitrile (entry 4, Table 1). Unexpectedly, the absence of 18-c-6 in the reaction media could substantially increase the yield of **2a** in both DCM and acetonitrile (entries 5 and 6), despite the fact that Cs_2_CO_3_ has low solubility in both solvents. This observation could be explained by the fact that the cationic diaryliodonium ion itself might help dissolve carbonate through ligand exchange^52^. Consequently, intramolecular deprotonation on C2-podition would occur to allow a smooth generation of 3-triflyloxybenzyne **i** under mild conditions (Fig. 1c)^38^. By contrast, Ochiai et al. reported that 18-c-6 was found to coordinate with aryliodonio groups and, hence, decreases its leaving ability^53^. Next, it was found that K_2_CO_3_ featured **2a** in slightly diminished yield (entry 7). Interestingly, while the employment of CsF furnished **2a** in only 52% yield at room temperature, this yield could raise to 77% when the reaction was carried out at 70 ^o^C in a sealed tube (entry 8). Notably, these activating reagents in our study, efficiently with Cs_2_CO_3_ and even effectively with CsF, are the weakest bases so far through *ortho*-deprotonative elimination strategy^20^. The prominent advantages by employing these weak inorganic bases as the activating reagents are both compatibility to various tandem reaction processes and excellent functional group tolerance as have been proven in our previous studies. In comparison, the absence of base gave no generation of 3-triflyloxybenzyne **i** at all (entry 9).

**Table 1:** Optimization of reaction conditions


Entries	EWG	Base	Additive	Solvent	Yield (%)^*b*^
1	OTf, **1b**	Cs_2_CO_3_	18-c-6	MeCN	**2a**, 64
2	OTf, **1b**	Cs_2_CO_3_	18-c-6	toluene	**2a**, 50
3	OTf, **1b**	Cs_2_CO_3_	18-c-6	THF	**2a**, 56
4	OTf, **1b**	Cs_2_CO_3_	18-c-6	DCM	**2a**, 65
5	OTf, **1b**	Cs_2_CO_3_	\	DCM	**2a**, 91
6	OTf, **1b**	Cs_2_CO_3_	\	MeCN	**2a**, 86
7	OTf, **1b**	K_2_CO_3_	\	DCM	**2a**, 79
8	OTf, **1b**	CsF	\	DCM	**2a**, 52 (77)^*c*^
9	OTf, **1b**	\	\	DCM	nr
10	OTs, **1c**	Cs_2_CO_3_	\	DCM	**2b**, 63
11	OR’, **1d**^*d*^	Cs_2_CO_3_	\	DCM	**2c**, 66

^a^Conditions: slow addition of a solution of **1** (0.4 mmol) in solvent (5 mL) to a suspension of furan (4 mmol), base (1.6 mmol), and additive (0.6 mmol) in solvent (5 mL) at rt over 3 hours. ^*b*^Isolated yield. ^*c*^The reaction was heated at 70 ^o^C in a sealed tube. ^*d*^OR’ = 2,6-difluorophenylsulfonate.

In addition to **1b**, we also prepared compounds **1c** and **1d** containing 3-sulfonyloxy groups other than OTf group (entries 10 and 11, Table 1). When compound **1c** bearing a 3-OTs group was employed, its reaction with furan afforded **2b** in 63% yield. Notably, compound **1c** can be seen as the structural analogue of 1,2-benzdiyne precursor TTPM (Fig. 1b)^45^. Similarly, compound **1d** containing a 2,6-difluorophenylsulfonate group at the C3-position is an analogue of TTPF (Fig. 1b)^47^, and its reaction with furan gave rise to the corresponding [4 + 2]-cycloadduct **2c** in 66% yield. Notably, compounds **1b**-**1d** are bench-stable white solid and do not decompose at room temperature for several months, making them appropriate 1,2-benzdiyne reagents.

### Substrate scope

**Fig. 3:**
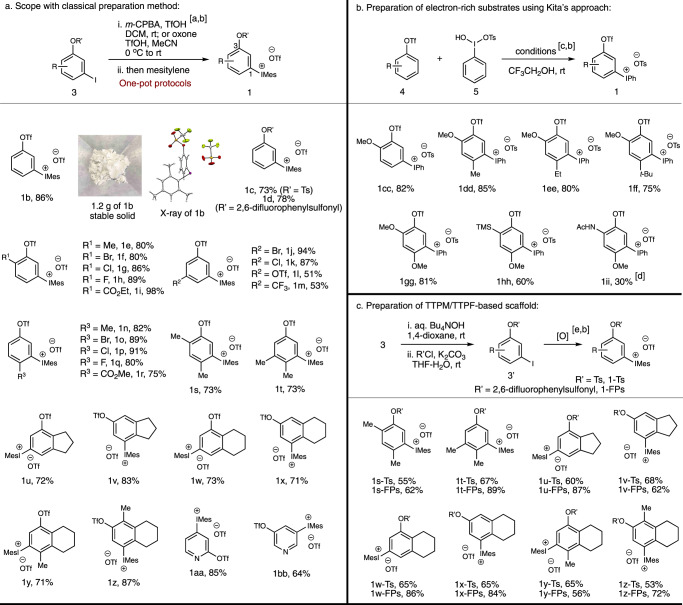
Scope for domino aryne precursors. **a** Scope with classical preparation method. **b** Preparation of electron-rich substrates using Kita’s approach. **c** Preparation of TTPM/TTPF-based scaffold. Reaction conditions: [a] A solution of **3** (1.0 mmol), *m*-CPBA or Oxone (1.5 mmol), triflic acid (2.0 mmol) in solvent (5.0 mL) was stirred from 0 ^o^C to rt for 3 hours; mesitylene (1.1 mmol) was then added. [b] Isolated yield. [c] A solution of **4** (0.22 mmol) and PhI(OH)OTs **5** (0.2 mmol) in 2,2,2-trifluoroethanol (2 mL) was stirred at rt. [d] The reaction was carried out in DCM (2 mL) with NaOTf (1.0 mmol). [e] A solution of **3′** (1.0 mmol), *m*-CPBA or Oxone (1.5 mmol), triflic acid (2.0 mmol) in solvent (5.0 mL) was stirred from 0 ^o^C to rt for 3 hours; mesitylene (1.1 mmol) was then added.

The ready accessibility of functionalized 3-iodophenyl triflates **3** either from commercial feedstocks or via typical manipulations on them allows the convenient synthesis of this array of 1,2-benzdiyne precursors **1**. Along with our study, several practical preparation protocols toward 3-iodophenyl triflates **3** were developed: (a) diazotization-iodination and triflation of substituted 3-aminophenols; (b) *meta*-nitration of *para*-alkylated phenyl triflate/reduction/diazotization-iodination; (3) *para*-nitration of aryl triflate/reduction/*ortho*-iodination of aniline/diazotization-reduction (please see the Supplementary Information (SI) for details). After accessing these substituted 3-iodophenyl triflates **3**, a series of 1,2-benzdiyne precursors **1e**-**1t** were readily synthesized upon oxidation with either *m*-CPBA^51^ or Oxone^54^ using one-pot oxidation protocols (Fig. 3a). Those substituents can be alkyl, halo, ester, trifluoromethyl, and triflyloxy groups. In addition, two pairs of benzofused analogues **1u**-**1x** were obtained. Depending on the location of the mesityliodonio group, different substituted 3-triflyloxybenzyne intermediates could be envisioned in each pair. Furthermore, penta-substituted aryne precursors **1** **y** and **1z** were prepared as well. It is worth mentioning that upon activation the corresponding arynes from compounds **1u**-**1z** are otherwise difficult to be reached from 2-silylaryl 1,3-bis(sulfonate) skeleton (Fig. 1b). Besides, 2,3-pyridiyne equivalent **1aa** and 3,4-pyridiyne equivalent **1bb** were successfully achieved.

We noticed that those substituents on 1,2-benzdiyne precursors in Fig. 3a are either alkyl groups or EWGs. Because electron-rich arenes could compete with mesitylene in the formation of diaryliodonium salt step, strong electron-donating groups (EDGs) were found to be incompatible with the reaction conditions through the above oxidation protocols. In order to incorporate EDGs onto the parent benzene ring, we decided to employ Kita’s approach, where direct dehydrative condensation of electron-rich arenes with PhI(OH)OTs (**5**) (Koser’s reagent) could occur in fluoroalcohol media^55^. As shown in Fig. 3b, the reaction of 2-methoxyphenyl triflate with Koser’s reagent in 2,2,2-trifluoroethanol featured compound **1cc** regioselectively in 82% yield. Activation of **1cc** with Cs_2_CO_3_ in DCM in the presence of furan could furnish the desired [4 + 2]-cycloadduct, confirming that the newly introduced phenyliodonio group on 2-methoxyphenyl triflate is on the *meta*-position of the OTf group. Similarly, compounds **1dd**-**1gg** were readily prepared using this method and the 2-methoxy group serves as an efficient directing group to regulate the incorporation of phenyliodonio group onto the C5-position exclusively. Moreover, 2-silyl group on *p*-methoxyphenyl triflate was found to tolerate the reaction conditions, furnishing compound **1hh** in 60% yield. Notably, compound **1hh** could also be seen as 1,3-benzdiyne equivalent, where chemoselective generation of the designated 3-triflyloxyaryne intermediate should occur. At last, compound **1ii** was also obtained, albeit in only 30% yield. By using this protocol, a series of aryne precursors containing strong electron-donating groups were obtained.

Our previous studies revealed that the leaving ability of the second leaving group on domino aryne precursors has profound influence to the efficiency of these reagents in various cascade processes, such as nucleophilic-ene reactions as well as nucleophilic and Diels-Alder cycloaddition reactions^18^. Consequently, those domino aryne precursors containing either an OTs group (TTPM) or a 2,6-difluorophenylsulfonyloxy (OFPs) group (TTPF) were found to be more appropriate ones than TPBT with two OTf groups. Therefore, it is desirable to find an approach to prepare those alternative yet indispensable aryne precursors containing either an OTs or an OFPs group. Since triflation of phenols belongs to one of the early steps in the preparation of many aryne precursors in Fig. 3a, a direct conversion of the OTf group to either OTs or OFPs group on 3-iodoaryl triflates **3** could provide a divergent solution and would be highly desirable. Pleasingly, by employing Nishiyama’s protocol^56^, deprotection of the OTf group on 3-iodoaryl triflates **3** with tetrabutylammonium hydroxide (Bu_4_NOH) in 1,4-dioxane was realized in quantitative yield. Upon one-pot protection with sulfonyl chloride, 3-iodoaryl sulfonates **3**′ were furnished (Fig. 3c). As such, we chose those iodobenzenes **3** that were used to prepare **1s**-**1z**, because these scaffolds are tetra- and pentasubstituted benzenes and could result in the formation of densely substituted arenes through domino aryne transformations. After oxidation of **3**′, compounds **1s-Ts**-**1z-Ts** as analogues of **1c** and **1s-FPs**-**1z-FPs** as analogues of **1d** could be prepared (Fig. 3c). The expeditious preparation of those 1,2-benzdiyne precursors in Fig. 3 sets up a general and potentially useful platform for 1,2-benzdiyne study.

### Synthetic applications

**Fig. 4:**
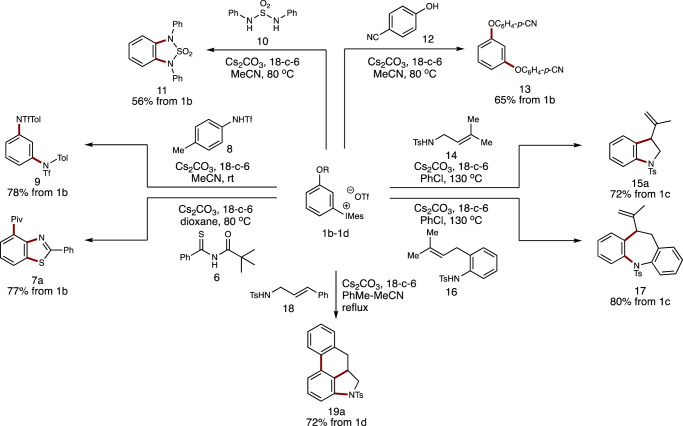
Study on domino aryne reactions with 1b-1d. All reactions are performed on a 0.2 mmol scale. Isolated yields are given.

After successfully prepared those 1,2-benzdiyne precursors, we set out to examine their reactivity in tandem 1,2-benzdiyne transformations in order to see whether this scaffold is compatible with different aryne reaction conditions. As shown in Fig. 4, the reactions of **1b**-**1d** with various substrates were tested. First, the reaction of **1b** with pivaloyl (Piv)-protected benzothioamide **6** afforded benzothiazole **7a** in 77% yield. Moreover, 1,3-diaminobenzene **9** could be produced in 78% yield by treating *N*-triflated aniline **8** with **1b** in acetonitrile at room temperature. Similarly, 1,2-diamination reaction with sulfonamide **10** took place to furnish **11** in 56% yield. Besides, electron-deficient *p*-cyanophenol **12** was found to serve as efficient nucleophile, featuring diaryl ether of resorcinol **13** in 65% yield. Notably, phenols have not yet been employed in our domino aryne transformations. Next, tandem nucleophilic-ene reactions of aryne precursor **1c** with tosylamides **14** and **16** were examined. As have been disclosed that aryne precursor TTPM bearing an OTs leaving group was mandatory for the success of this transformation, because the departure rate of the OTs group matches well with that of the intramolecular ene reaction. Indeed, when aryne precursor **1c** was employed using the standard conditions (chlorobenzene at 130 ^o^C) in our previous study^45^, the corresponding products **15a** and **17** were achieved, respectively, in good yields. At last, cascade nucleophilic and Diels-Alder reactions were examined^47^. Unexpectedly, the reaction of **1c** with cinnamyl amide **18** could feature **19a** in only 36% yield using chlorobenzene at 130 ^o^C. Gratifyingly, by employing **1d** bearing an OFPs group and using toluene-MeCN as binary solvent system, tetracyclic compound **19a** was obtained in 72% yield. This observation indicates that the OFPs group on **1d** is more appropriate than the OTs group on **1c** in this tandem nucleophilic and Diels-Alder reaction process, which is different from our previous domino aryne system in the same transformation^47^. The examples in Fig. 4 clearly illustrated that our 1,2-benzdiyne scaffold has comparable efficiency with those using 2-silylaryl 1,3-bis(sulfonate) skeleton.

**Fig. 5:**
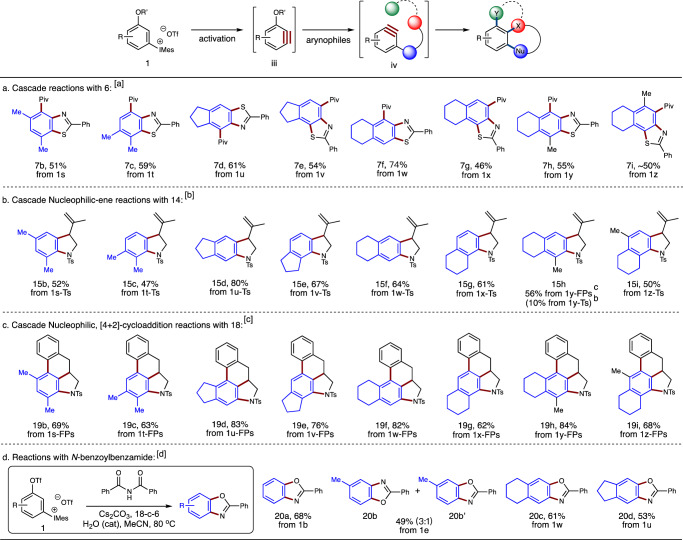
Preparation of densely functionalized arenes. **a** Cascade reactions with **6**. **b** Cascade nucleophilic-one reactions with **14**. **c** Cascade nucleophilic, [4 + 2]-cycloaddition reactions with **18**. **d** Reactions with *N*-benzoylbenzamide. Reaction conditions: [a] Slow addition of a solution of **1** (0.4 mmol) in 1,4-dioxane (3 mL) to a suspension of benzothioamide **6** (0.2 mmol), Cs_2_CO_3_ (1.6 mmol), and 18-c-6 (0.4 mmol) in 1,4-dioxane (3 mL) at 80 ^o^C over 3 hours. [b] Slow addition of a solution of **1-TS** (0.4 mmol) in chlorobenzene (3 mL) to a suspension of tosylamide **14** (0.2 mmol), Cs_2_CO_3_ (1.6 mmol), and 18-c-6 (0.4 mmol) in chlorobenzene (3 mL) at 130 ^o^C over 3 hours. [c] Slow addition of a solution of **1-FPs** (0.4 mmol) in MeCN (7 mL) to a suspension of cinnamyl amide **18** (0.2 mmol), Cs_2_CO_3_ (1.2 mmol), and 18-c-6 (0.1 mmol) in toluene (7 mL) at 100 ^o^C over 4 hours. [d] Slow addition of a solution of **1** (0.8 mmol) in acetonitrile (5 mL) to a suspension of *N*-benzoylbenzamide (0.4 mmol), Cs_2_CO_3_ (1.6 mmol), water (0.08 mmol), and 18-c-6 (0.2 mmol) in acetonitrile (5 mL) at 80 ^o^C over 8 hours.

Beyond mild generation conditions, another distinct advantage for this array of 1,2-benzdiyne precursors is the predictability in both the site-selective generation of 1,2-aryne intermediate and the subsequent aryne reactions. Because of the better-leaving tendency of the aryliodonio group than that of the sulfonyloxy group, 3-sulfonyloxyaryne intermediate **iii** should be always generated preferentially upon deprotonation with base (Fig. 5). Since sulfonyloxy groups are *σ*-EWGs, they could direct the incoming nucleophile to attack their *meta*-positions to release 2,3-aryne **iv** (Fig. 5). Consequently, unsymmetrically substituted 3-sulfonyloxyarynes **iii** would then be converted to the corresponding products via domino aryne processes in regioselective manner. To demonstrate this property, aryne precursors **1s**-**1z** containing two or more additional substituents were chosen for our study. It was envisioned that heavily substituted arenes should be achieved via different domino aryne transformations.

We first examined the reactions between benzothioamide **6** and aryne precursors **1s**-**1z**. Penta-substituted benzothiazoles **7b**-**7g** and hexa-substituted benzothiazoles **7h** and **7i** were obtained in moderate to good yields. Notably, topological isomers **7d** and **7e** were prepared in regiospecific manner from **1u** and **1v**, respectively (Fig. 5a). Similar results were also observed with respect to **7f** and **7g** as well as **7h** and **7i**, those of which were exclusively derived from their corresponding 1,2-benzdiyne precursors. Distinctively, complementary regioselective control could be realized through the employment of this 1,2-benzdiyne scaffold. Next, tandem nucleophilic-ene reactions of **14** with aryne precursors **1s-Ts**-**1z-Ts** were studied, featuring the corresponding indoline products **15b**-**15g** and **15i** in moderate to high yields (Fig. 5b). However, it was found that compound **15h** was achieved in only 10% yield from **1y-Ts**. Gratifyingly, when aryne precursor **1y-FPs** was employed, its reaction with **14** in toluene-MeCN binary solvent was able to deliver **15h** in 56% yield. At last, cascade nucleophilic and Diels-Alder reactions with cinnamyl amide **18** were tested. As shown in Fig. 5c, its reactions with **1s-FPs**-**1z-FPs** could readily construct tetra- and pentacyclic ring systems **19b**-**19i**. These examples revealed that our 1,2-benzdiyne precursors should find a broad spectrum of synthetic applications, where both heavily substituted arenes and benzofused polycyclic ring systems with diverse substitution patterns could be accomplished. Particularly, those structures containing benzothiazole, indoline, and benzofused [6,5,6,6]-tetracyclic ring system possess potential biological activity, which are otherwise almost incapable to access through 2-silylaryl 1,3-bis(sulfonate) framework.

Besides, a reactivity of domino aryne system was discovered with this set of precursor scaffold. As shown in Fig. 5d, the reaction of aryne precursor **1b** with *N*-benzoylbenzamide gave rise to the formation of benzoxazole **20a** in 68% yield. This reaction is apparently different from that with benzothioamide **6**^40^, in which the benzoyl group does not migrate to the 3-position of the parent aryne ring. When aryne precursor **1e** was employed, a mixture of **20b** and **20b’** was obtained in 49% yield with a 3:1 ratio, illustrating that both the nitrogen and the oxygen on *N*-benzoylbenzamide could serve as the first nucleophile to attack the generated 3-triflyloxybenzyne intermediate. By employing **1w** and **1u** as the aryne precursors, the corresponding benzoxazoles **20c** and **20d** were achieved, respectively, in moderate yields.

**Fig. 6:**
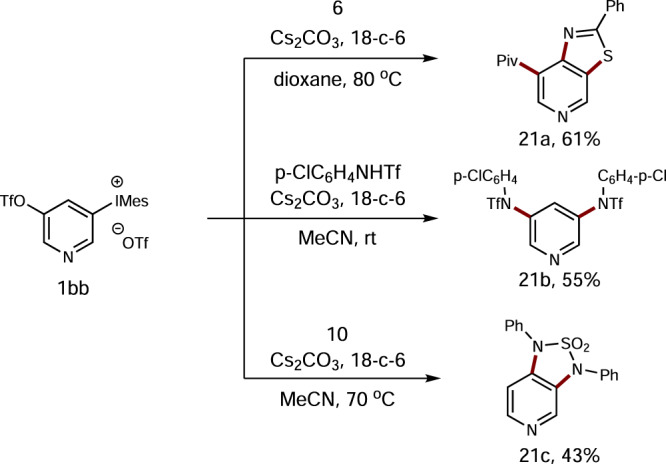
Study on pyridiyne precursor 1bb. All reactions are performed on a 0.2 mmol scale. Isolated yields are given.

In addition, we also carried out preliminary investigations on pyridiyne equivalents. As shown in Fig. 6, 3,4-pyridiyne precursor **1bb** was first examined. Its reaction with benzothioamide **6** could furnish benzothiazole **21a** in 61% yield. Moreover, both 1,3-diamination and 1,2-diamination were tested, and **21b** and **21c** were obtained in moderate yields (Fig. 6). Unfortunately, efforts to apply both tandem nucleophilic-ene reaction as well as nucleophilic and [4 + 2]-cycloaddition processes failed to produce the desired products at all, suggesting that 3,4-pyridiyne precursor **1bb** has different reaction behavior comparing with its 1,2-benzdiyne counterpart **1b**. When 2,3-pyridiyne precursor **1aa** was treated with various arynophiles, only complex mixtures were observed with no formation of the desired 2,3-pyridiyne reaction products.

**Fig. 7:**
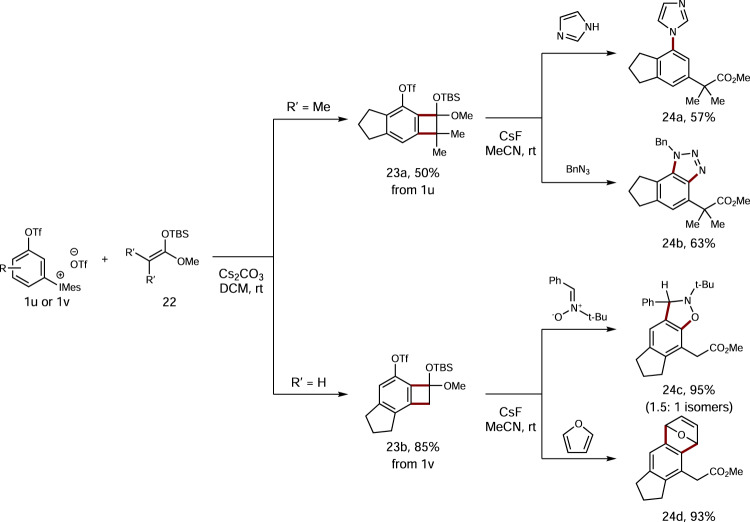
Manipulation through [2 + 2]-cycloaddition, Grob-fragmentation processes. All reactions are performed on a 0.4 mmol scale. Isolated yields are given.

Next, we wanted to see whether this 1,2-benzdiyne scaffold could be applied in the [2 + 2]-cycloaddition, Grob-fragmentation process previously developed by our group^44^. As shown in Fig. 7, we chose aryne precursors **1** **u** and **1** **v** in our study. First, formal [2 + 2]-cycloaddition of **1** **u** with ketene silyl acetal (KSA) **22a** (R’ = Me) could afford cycloadduct **23a** in 50% yield in regioselective manner. Similarly, compound **23b** was obtained in 85% yield from **1** **v** and **22b** (R’ = H) as well. It is worth mentioning that in our previous study the reaction between TPBT and KSAs **22** gave no [2 + 2]-cycloaddition products at all^44^, suggesting that 3-sulfonyloxyaryl(mesityl)iodonium triflates are more amenable to [2 + 2]-cycloaddition reactions than TPBT-based framework. With both tricyclic compounds **23a** and **23b** in hand, we then selected several arynophiles to capture the 2,3-aryne intermediates generated upon activation with CsF in acetonitrile. The reaction of **23a** with imidazole gave the nucleophilic addition product **24a** as a single isomer in 57% yield. Meanwhile, its reaction with benzyl azide furnished a [3 + 2]-cycloadduct **24b** in 63% yield in excellent regioselectivity. On the other hand, the reactions of cycloadduct **23b** with either nitrone or furan produced the corresponding cycloadducts **24c** and **24d** in excellent yields, both of which are pentasubstituted arenes. In view of the potential of this two-step process in accommodating a variety of arynophiles as well as reaction modes, diverse patterns of densely substituted arenes could be foreseen by combining this process with these 1,2-benzdiyne precursors.

**Fig. 8:**
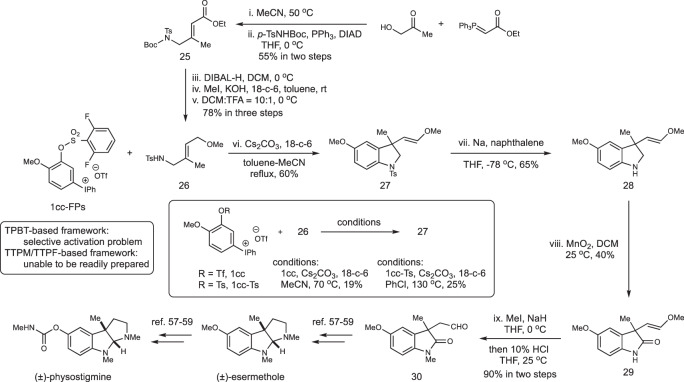
Study toward the synthesis of (±)-esermethole and (±)-physostigmine. A preparation of compound **30** was realized by employing domino aryne nucleophilic-ene reaction between aryne precursor **1cc-FPs** and compound **26** as the key step. After obtaining compound **27**, subsequent deprotection, oxidation, N-methylation, and conversion of the enol methyl ether to aldehyde gave rise to the desired compound **30**. Isolated yields are given.

In order to demonstrate the potential of our domino aryne system, particular those bearing 3-sulfonyloxyaryl(mesityl)iodonium triflate structural motif, we carried out a synthesis toward natural products (±)-esermetole and (±)-physostigmine^57–59^. As shown in Fig. 8, the Wittig reaction between 1-hydroxypropan−2-one and ethyl 2-(triphenyl-*λ*^5^-phosphaneylidene)acetate, followed by Mitsunobu reaction with *tert*-butyl tosylcarbamate (*p*-TsNHBoc), could furnish compound **25** in 55% yield over two steps. Next, sequential reduction, O-methylation, and deprotection on compound **25** delivered compound **26** in 78% yield in a one-pot, three-step fashion. Meanwhile, aryne precursor **1cc-FPs** was readily prepared from 2-methoxyphenol using the Kita’s approach (Fig. 3b). The key step for domino aryne nucleophilic-ene reaction between **1cc-FPs** and compound **26** in toluene-acetonitrile binary solvent system could furnish compound **27** in 60% yield. In this transformation, we noticed that the second leaving group on the domino aryne precursor framework is essential to allow acceptable level of reaction efficiency. In comparison, aryne precursors containing either an OTf group (**1cc**) or an OTs group (**1cc-Ts**) on the 3-position only resulted in the formation of compound **27** in low yield. By contrast, it is difficult to prepare the corresponding domino aryne precursor containing 4-methoxy group based on the TTPM/TTPF framework and there will be no site selective activation upon aryne generation from TPBT-based scaffold (Fig. 1b). Therefore, the construction of compound **27** demonstrates a great potential of this type of 1,2-benzdiyne scaffold in synthesizing a broad range of useful molecules containing functionalized arenes. Next, deprotection of the tosyl group on compound **27** with sodium-naphthalene solution in THF furnished compound **28** in 65% yield, which was followed by oxidation of **28** with manganese dioxide to feature compound **29** in 40% yield. Subsequent N-methylation and conversion of the enol methyl ether to aldehyde could be realized in one-pot fashion to afford compound **30** in 90% overall yield. It has been reported that compound **30** would then be transformed to (±)-esermetole and (±)-physostigmine according to literature precedents^57–59^.

In summary, a class of 1,2-benzdiyne precursors were successfully developed by employing *ortho*-deprotonative elimination strategy as the generation method under mild conditions. A broad range of precursors with various substituents on the parent arene ring, including benzofused scaffolds as well as heavily functionalized analogues, could be conveniently prepared. Distinctively, weak bases, such as carbonate and even fluoride salts, were found to serve as efficient activating reagents, which are the weakest bases used in *ortho*-deprotonative elimination strategies so far. As an exhibition, a broad spectrum of intensely functionalized arenes as well as polycyclic ring systems were achieved, those of which contain potential biological properties. This protocol represents a breakthrough in 1,2-benzdiyne chemistry with high functional group compatibility as well as excellent chemoselective control upon activation. In a broader context, our study refreshes the traditional understanding that *ortho*-deprotonative elimination strategies normally require strong bases to realize deprotonation task and, hence, have been limited in only a few types of aryne transformations. It can be envisioned that this array of 1,2-benzdiyne precursors not only provide a convenient maneuver to access various substituted analogues, but also could be used in the exploration of unexplored aryne/domino aryne transformations.

## Methods

### General procedure for the transformation of aryl triflate to aryl tosylate or aryl 2,6-difluorophenylsulfonate

To a solution of 3-iodoaryl triflate (1.0 mmol, 1.0 equiv) in 1,4-dioxane (20 mL) at room temperature was added 40% aq. Bu_4_NOH (1.33 mL, 2.0 mmol, 2.0 equiv). After stirred for three hours, water (20 mL) was added and it was extracted with DCM (20 mL x 3). The combined organic layers were washed with brine (20 mL), dried over Na_2_SO_4_, filtered, and concentrated. Crude oil was obtained, which was used directly in the next step without further purification.

To a solution of the above crude oil in THF (20 mL) at room temperature was added K_2_CO_3_ (276 mg, 2.0 mmol, 2.0 equiv) and *p*-TsCl (0.22 mL, 1.5 mmol, 1.5 equiv) or 2,6-difluorophenylsulfonyl chloride (0.20 mL, 1.5 mmol, 1.5 equiv). After stirring for three hours, water (20 mL) was added and it was extracted with DCM (20 mL x 3). The combined organic layers were washed with brine (20 mL), dried over Na_2_SO_4_, filtered, and concentrated. Flash column chromatography on silica gel afforded the corresponding 3-iodoaryl tosylate or 3-iodoaryl 2,6-difluorophenylsulfonate.

### General procedure for the oxidation of aryl iodides to form aryne precursors 1

(a) Oxidation with *m*-CPBA: To a solution of aryl iodide (1.0 mmol, 1.0 equiv) and TfOH (0.16 mL, 2.0 mmol, 2.0 equiv) in DCM (30 mL) at room temperature was added *m*-CPBA (190 mg, 1.1 mmol, 1.1 equiv). After stirred for 30 minutes, 1,3,5-trimethylbenzene (0.15 mL, 1.1 mmol, 1.1 equiv) was added. After three hours, all the volatiles were directly removed on a rotary evaporator. The resulting crude oil was triturated with diethyl ether and isolated by filtration to afford pure aryne precursor **1**. (b) Oxidation with Oxone: To a solution of aryl iodide (1.0 mmol, 1.0 equiv) and TfOH (0.16 mL, 2.0 mmol, 2.0 equiv) in MeCN (30 mL) at 0 °C was added Oxone (923 mg, 1.5 mmol, 1.5 equiv). After stirred for five minutes, 1,3,5-trimethylbenzene (0.15 mL, 1.1 mmol, 1.1 equiv) was added. After three hours, all the volatiles were directly removed on a rotary evaporator. The resulting crude oil was triturated with diethyl ether and isolated by filtration to afford pure aryne precursor **1**.

### General procedure for the synthesis of 1cc-1jj

To a solution of compound **4** (0.22 mmol, 1.1 equiv) in anhydrous 2,2,2-trifluoroethyl alcohol (2 mL) at room temperature was added Koser’s reagent **5** (78.4 mg, 0.2 mmol, 1.0 equiv). After eight hours, it was filtrated. All the volatiles were then removed on a rotary evaporator. The resulting crude oil was triturated with diethyl ether and isolated by filtration to afford pure aryne precursor **1**.

## Supplementary information

Supplementary Information

## Data Availability

The data generated in this study are provided within the article and in the Supplementary Information. Details about materials and methods, experimental procedures, characterization data, and NMR spectra are available in the Supplementary Information. The crystallographic data for compound **1b** can be obtained free of charge from the Cambridge Crystallographic Data Centre (CCDC) under reference number 2052067.

## References

[1] Hoffmann, R. W. *Dehydrobenzene and Cycloalkynes*; Academic Press, New York, 1967.

[2] Wenk HH, Winkler M, Sander W (2003). One century of Aryne chemistry. Angew. Chem. Int. Ed..

[3] Biju, A. T. *Modern Aryne Chemistry*; Wiley-VCH Verlag GmbH & Co. KGaA, Boschstr.: Weinheim, Germany, 2021.

[4] Himeshima Y, Sonoda T, Kobayashi H (1983). Fluoride-Induced 1,2-Elimination of *o*-Trimethylsilylphenyl Triflate to Benzyne under mild conditions. Chem. Lett..

[5] Fluegel LL, Hoye TR (2021). Hexadehydro-Diels-Alder Reaction: Benzyne generation via cycloisomerization of tethered Triynes. Chem. Rev..

[6] Sanz R (2008). Recent applications of Aryne chemistry to organic synthesis. A review. Org. Prep. Proced. Int..

[7] Gampe CM, Carreira EM (2012). Arynes and Cyclohexyne in natural product synthesis. Angew. Chem. Int. Ed..

[8] Tadross PM, Stoltz BM (2012). A comprehensive history of Arynes in natural product total synthesis. Chem. Rev..

[9] Bhunia A, Yetra SR, Biju AT (2012). Recent advances in transition-metal-free carbon-carbon and carbon-heteroatom bond-forming reactions using Arynes. Chem. Soc. Rev..

[10] Dubrovskiy AV, Markina NA, Larock RC (2013). Use of Benzynes for the synthesis of heterocycles. Org. Biomol. Chem..

[11] Pérez D, Peña D, Guitián E (2013). Aryne Cycloaddition reactions in the synthesis of large polycyclic aromatic compounds. Eur. J. Org. Chem..

[12] Goetz AE, Shah TK, Garg NK (2015). Pyridynes and Indolynes as building blocks for functionalized heterocycles and natural products. Chem. Commun..

[13] García-López J-A, Greaney MF (2016). Synthesis of Biaryls using Aryne intermediates. Chem. Soc. Rev..

[14] Bhojgude SS, Bhunia A, Biju AT (2016). Employing Arynes in Diels-Alder reactions and transition-metal-free multicomponent coupling and arylation reactions. Acc. Chem. Res..

[15] Shi J, Li Y, Li Y (2017). Aryne multifunctionalization with Benzdiyne and Benztriyne equivalents. Chem. Soc. Rev..

[16] Takikawa H, Nishii A, Sakai T, Suzuki K (2018). Aryne-based strategy in the total synthesis of naturally occurring polycyclic compounds. Chem. Soc. Rev..

[17] Pozo I, Guitián E, Pérez D, Peña D (2019). Synthesis of nanographenes, starphenes, and sterically congested polyarenes by aryne cyclotrimerization. Acc. Chem. Res..

[18] He J, Qiu D, Li Y (2020). Strategies toward Aryne Multifunctionalization via 1,2-Benzdiyne and Benzyne. Acc. Chem. Res..

[19] Werz DB, Biju AT (2020). Uncovering the neglected similarities of arynes and donor-acceptor cyclopropanes. Angew. Chem., Int. Ed..

[20] Shi J, Li L, Li Y (2021). *o*-Silylaryl Triflates: A Journey of Kobayashi Aryne precursors. Chem. Rev..

[21] Wickham PP (1991). Benzyne generation from Aryl Triflates. J. Org. Chem..

[22] Olofson RA, Dougherty CM (1973). Lithium 2,2,6,6-Tetramethylpiperidide and related, strong, proton-specific bases. Evaluation in synthesis. J. Am. Chem. Soc..

[23] Truong T, Daugulis O (2011). Base-mediated intermolecular sp^2^ C-H bond Arylation via Benzyne intermediates. J. Am. Chem. Soc..

[24] Truong T, Mesgar M, Le KKA, Daugulis O (2014). General method for functionalized polyaryl synthesis via aryne intermediates. J. Am. Chem. Soc..

[25] Truong T, Daugulis O (2012). Direct intermolecular Aniline *ortho*-Arylation via Benzyne intermediates. Org. Lett..

[26] Truong T, Daugulis O (2013). Divergent reaction pathways for phenol arylation by arynes: Synthesis of Helicenes and 2-Arylphenols. Chem. Sci..

[27] Mesgar M, Nguyen-Le J, Daugulis O (2018). New hindered amide base for Aryne insertion into Si–P, Si–S, Si–N, and C–C bonds. J. Am. Chem. Soc..

[28] Uchiyama M (2002). Generation of functionalized asymmetric Benzynes with TMP-Zincates. Effects of ligands on selectivity and reactivity of Zincates. J. Am. Chem. Soc..

[29] Uchiyama M (2008). Generation and suppression of 3-/4-Functionalized Benzynes using Zinc Ate Base (TMP-Zn-ate): New approaches to multisubstituted Benzenes. J. Am. Chem. Soc..

[30] Akiyama T, Imasaki Y, Kawanisi M (1974). Arylation of Tetrazolide with Diaryliodonium Halides: Evidence for intermediacy of benzyne. Chem. Lett..

[31] Sundalam SK, Nilova A, Seidl TL, Stuart DR (2016). A selective C-H deprotonation strategy to access functionalized arynes by using hypervalent iodine. Angew. Chem., Int. Ed..

[32] Stuart DR (2017). Unsymmetrical Diaryliodonium salts as aryne synthons: Renaissance of a C-H deprotonative approach to Arynes. Synlett.

[33] Nilova A (2021). Regioselective synthesis of 1,2,3,4-Tetrasubstituted Arenes by vicinal functionalization of Arynes derived from Aryl(Mes)iodonium salts. Chem. Eur. J..

[34] Nilova A, Metze B, Stuart DR (2021). Aryl(Tmp)iodonium Tosylate reagents as a strategic entry point to diverse aryl intermediates: selective access to arynes. Org. Lett..

[35] Okuyama T, Takino T, Sueda T, Ochiai M (1995). Solvolysis of Cyclohexenyliodonium salt, a new precursor for the vinyl cation: remarkable Nucleofugality of the Phenyliodonio Group and evidence for internal return from an intimate ion-molecule pair. J. Am. Chem. Soc..

[36] Lanzi M, Dherbassy Q, Wencel-Delord J (2021). Cyclic Diaryl *λ*^3^-Bromanes as original Aryne precursors. Angew. Chem., Int. Ed..

[37] Lanzi M, Ali Abdine RA, De Abreu M, Wencel-Delord J (2021). Cyclic Diaryl *λ*^3^-Bromanes: A rapid access to molecular complexity via cycloaddition reactions. Org. Lett..

[38] Lanzi M, Rogge T, Truong TS, Houk KN, Wencel-Delord J (2023). Cyclic Diaryl *λ*^3^-Chloranes: Reagents and their C-C and C-O couplings with phenols via aryne intermediates. J. Am. Chem. Soc..

[39] Ochiai M (2009). Hypervalent Aryl-, Alkynyl-, and Alkenyl- *λ*^3^-Bromanes. Synlett.

[40] Shi J, Qiu D, Wang J, Xu H, Li Y (2015). Domino Aryne precursor: Efficient construction of 2,4-Disubstituted Benzothiazoles. J. Am. Chem. Soc..

[41] Qiu D, Shi J, Li Y (2015). Domino Aryne precursor: A step beyond the boundary of traditional aryne chemistry. Synlett.

[42] Qiu D, He J, Yue X, Shi J, Li Y (2016). Diamination of Domino Aryne precursor with sulfonamides. Org. Lett..

[43] Li L, Qiu D, Shi J, Li Y (2016). Vicinal diamination of Arenes with Domino Aryne precursors. Org. Lett..

[44] Shi J, Xu H, Qiu D, He J, Li Y (2017). Selective Aryne formation via Grob fragmentation from the [2+2] Cycloadducts of 3-Triflyloxyarynes. J. Am. Chem. Soc..

[45] Xu H (2018). Domino Aryne annulation via a Nucleophilic-Ene process. J. Am. Chem. Soc..

[46] Lv C, Wan C, Liu S, Lan Y, Li Y (2018). Aryne Trifunctionalization enabled by 3-Silylaryne as a 1,2-Benzdiyne equivalent. Org. Lett..

[47] He J (2019). Arene Trifunctionalization with highly fused ring systems through a Domino Aryne Nucleophilic and Diels-Alder Cascade. Angew. Chem., Int. Ed..

[48] Dong Y, Lipschutz MI, Tilley TD (2016). Regioselective, transition metal-free C-O coupling reactions involving aryne intermediates. Org. Lett..

[49] Shen K, Fu Y, Li J-N, Liu L, Guo Q-X (2007). What are the pK_a_ values of C-H bonds in aromatic heterocyclic compounds in DMSO?. Tetrahedron.

[50] Kitamura T, Gondo K, Oyamada J (2017). Hypervalent Iodine/Triflate Hybrid Benzdiyne equivalents: Access to controlled synthesis of polycyclic aromatic compounds. J. Am. Chem. Soc..

[51] Bielawski M, Zhu M, Olofsson B (2007). Efficient and general One-Pot synthesis of Diaryliodonium Triflates: Optimization, scope and limitations. Adv. Synth. Catal..

[52] Yoshimura A, Zhdankin VV (2016). Advances in synthetic applications of hypervalent iodine compounds. Chem. Rev..

[53] Ochiai M, Suefuji T, Miyamoto K, Shiro M (2005). Effects of complexation with 18-Crown-6 on the Hypernucleofugality of Phenyl-*λ*^3^-Iodanyl Groups. Synthesis of Vinyl-*λ*^3^-Iodane Center·18-Crown-6 Complex. Org. Lett..

[54] Soldatova N (2018). One-pot synthesis of diaryliodonium salts from arenes and aryl iodides with oxone-sulfuric acid. Beilstein J. Org. Chem..

[55] Dohi T (2007). Versatile direct dehydrative approach for Diaryliodonium(III) salts in Fluoroalcohol Media. Chem. Commun..

[56] Ohgiya T, Nishiyama S (2004). A simple deprotection of triflate esters of phenol derivatives. Tetrahedron Lett..

[57] Matsuura T, Overman LE, Poon DJ (1998). Catalytic asymmetric synthesis of either Enantiomer of the Calabar Alkaloids Physostigmine and Physovenine. J. Am. Chem. Soc..

[58] Trost BM, Zhang Y (2006). Molybdenum-catalyzed asymmetric allylation of 3-Alkyloxindoles: Application to the formal total synthesis of (-)-Physostigmine. J. Am. Chem. Soc..

[59] Wang T, Yao W, Zhong F, Pang GH, Lu Y (2014). Phosphine-catalyzed Enantioselective γ-Addition of 3-Substituted Oxindoles to 2,3-Butadienoates and 2-Butynoates: Use of Prochiral Nucleophiles. Angew. Chem., Int. Ed..

