# Supplementary material for: 3-sulfonyloxyaryl(mesityl)iodonium triflates as 1,2-benzdiyne precursors with activation via ortho-deprotonative elimination strategy
Source: Nat Commun. 2023 Apr 3;14:1841. doi: 10.1038/s41467-023-37196-3 (PMC10070408; doi:10.1038/s41467-023-37196-3)
Supplement: Supplementary file 1 — Supplementary Information [file 41467_2023_37196_MOESM1_ESM.pdf]

SUPPLEMENTARY INFORMATION

3-Sulfonyloxyaryl(mesityl)iodonium Triflates as 1,2-Benzdiyne Precursors with Activation via *ortho*-Deprotonative Elimination Strategy

Haoyin Yuan,[1,†] Wenhao Yin,[1,†] Jili Hu,[1] and Yang Li*[1,2]

[1]School of Chemistry and Chemical Engineering, Chongqing University, 174 Shazheng Street, Chongqing, P. R. China, 400030
[2]College of Chemistry, Jilin University, Changchun, P. R. China 130012
[†]These authors contributed equally: Haoyin Yuan, Wenhao Yin.
*Correspondence to: y.li@cqu.edu.cn

Table of Contents

1. Supplementary Notes
2. Supplementary Methods
    2.1. Preparation of 3-iodoaryl triflates
    2.2. General procedure for the transformation of aryl triflate to aryl tosylate or aryl 2,6-difluorophenylsulfonate (Procedure A)
    2.3. General procedure for the preparation of aryne precursors **1** (Procedures B and C)
    2.4. General procedure for the synthesis of **1cc-1ii** (Procedure D)
    2.5. Domino 1,2-benzdiyne reactions with **1b-1d**
    2.6. General procedure for the synthesis of **7b-7i** (Procedure E)
    2.7. General procedure for the synthesis of **15b-15i** (Procedure F)
    2.8. General procedure for the synthesis of **19b-19i** (Procedure G)
    2.9 General procedure for the synthesis of **20** (Procedure H)
    2.10. Study on 3,4-pyridyne precursor
    2.11. Study on [2+2]-cycloaddition, Grob-fragmentation approach
    2.12. Study toward the synthesis of (±)-esermethole and (±)-physostigmine
    2.13. Crystal data and structure refinement for compound **1b**
3. Supplementary Figures for ^{1}H and ^{13}C NMR Spectra
4. Supplementary References

1. Supplementary Notes

All reagents were obtained from Adamas, Aladin, or Accela and used without further purification unless otherwise noted. To prevent moisture, Cs_2CO_3, K_2CO_3, CsF, and 18-c-6 were stored and used under inert atmosphere in Glovebox. The products were purified by column chromatography with Huanghai Silica Gel 50-75 um, ultrapure silica gel. ^{1}H and ^{13}C spectra were recorded in $CDCl_3$ (with 0.03% Me_4Si), DMSO-*d*6, CD_3OD or acetone-*d*6 using a Agilent 400 spectrometer. Chemical shifts (δ) are reported in ppm downfield from Me_4Si (δ 0.00 for ^{1}H NMR in $CDCl_3$, DMSO-*d*6, CD_3OD or acetone-*d*6) or the solvent peak (δ 7.26 for ^{1}H NMR in $CDCl_3$, 2.50 for ^{1}H NMR in DMSO-*d*6, δ 3.31 for ^{1}H NMR in CD_3OD, δ 2.05 for ^{1}H NMR in acetone-*d*6, δ 77.23 for ^{13}C NMR in $CDCl_3$, δ 39.50 for ^{13}C NMR in DMSO-*d*6, δ 29.85 for ^{13}C NMR in acetone-*d*6) as an internal reference with coupling constants (J) in hertz (Hz). Data are reported as follows: s = singlet, d = doublet, t = triplet, q = quartet, m = multiplet, brs = broad singlet. IR spectra were measured on a Nicolet iS50 FT-IR spectrometer using KBr plates. The high-resolution mass spectra (HRMS) were recorded on Bruker Solarix 7.0 T (ESI), Agilent 6224 TOF LC/MS spectrometer (ESI). Single-crystal X-ray diffraction (XRD) data were collected on a Super Nova-CCD using graphite-monochromated Mo Kα radiation (λ = 0.71073 Å) at 153 K or 293 K.

2. Supplementary Methods

2.1 Preparation of 3-iodoaryl triflates:

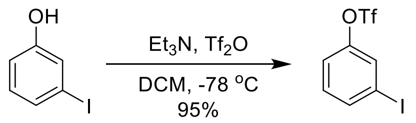

6a

3-Iodophenyl triflate: To a solution of commercially available 3-iodophenol (1.0 g, 4.57 mmol, 1.0 equiv) in anhydrous DCM (20 mL) at -78 ℃was added Et_3N (0.95 mL, 6.86 mmol, 1.5 equiv). After stirred for 5 minutes, trifluoromethanesulfonic anhydride (Tf_2O) (0.85 mL, 5.02 mmol, 1.1 equiv) was added slowly. After 10 minutes, all the volatiles were directly removed on a rotary evaporator. Flash column chromatography on silica gel afforded 1.5 g (95%) of 3-iodophenyl triflate as a colorless oil, the ^{1}H NMR of which is identical with that reported in literature.[1] ^{1}H NMR (400 MHz, $CDCl_3$) δ 7.73 (t, J = 7.9 Hz, 1H), 7.64 (t, J = 1.8 Hz, 1H), 7.27 (t, J_1 = 8.4 Hz, J_2 = 1.8 Hz, 1H), 7.17 (t, J = 8.4 Hz, 1H) ppm.

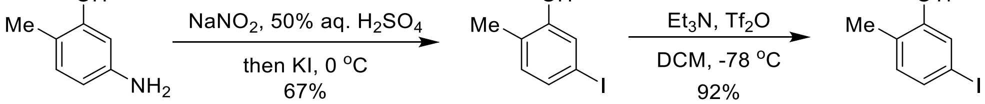

5-Iodo-2-methylphenyl triflate: To a solution of commercially available 5-amino-2-methylphenol (1.0 g, 8.13 mmol, 1.0 equiv) in 50% aq. H_2SO_4 (20 mL) at

0 ℃ was added $NaNO_2$ (617 mg, 8.94 mmol, 1.1 equiv) in portions. After stirred for 20 minutes, the resulting solution was slowly poured into a solution of KI (2.70 g, 16.26 mmol, 2.0 equiv) in water (30 mL) at 0 ℃. It was extracted with EtOAc (20 mL x 3). The combined organic layers were washed with brine (20 mL), dried over Na_2SO_4, filtered, and concentrated. Flash column chromatography with pet ether afforded 1.27 g (67%) of 5-iodo-2-methylphenol as a colorless oil. ^{1}H NMR (400 MHz, $CDCl_3$) δ 7.17 (dd, J_1 = 8.0 Hz, J_2 = 1.2 Hz, 1H), 7.11 (d, J = 1.2 Hz, 1H), 6.84 (d, J =8.0 Hz, 1H), 4.89 (s, 1H), 2.19 (s, 3H) ppm; ^{13}C NMR (100 MHz, $CDCl_3$) δ 154.7, 132.7, 130.0, 124.0, 90.5, 15.7 ppm; IR (thin film) 3371, 2917, 1578, 1490, 1405, 1334, 1251, 1225, 1181, 1117, 1033, 984, 799, 566, 436 (cm^{-1}); HRMS (ESI) m/z: calcd for C_7H_6IO [M - H]$^-$, 232.9469; found 232.9466.

To a solution of 5-iodo-2-methylphenol (1.0 g, 4.27 mmol, 1.0 equiv) in anhydrous DCM (20 mL) at -78 ℃ was added Et_3N (0.89 mL, 6.41 mmol, 1.5 equiv). After stirred for 5 minutes, Tf_2O (0.79 mL, 4.70 mmol, 1.1 equiv) was then added slowly. After 10 minutes, all the volatiles were directly removed on a rotary evaporator. Flash column chromatography on silica gel afforded 1.44 g (92%) of 5-iodo-2-methylphenyl triflate as a colorless oil. ^{1}H NMR (400 MHz, $CDCl_3$) δ 7.60 (dd, J_1 = 8.0 Hz, J_2 = 1.2 Hz, 1H), 7.56 (s, 1H), 7.04 (d, J = 8.0 Hz, 1H), 2.33 (s, 3H), ppm; ^{13}C NMR (100 MHz, $CDCl_3$) δ 148.4, 137.6, 133.7, 131.0, 130.4, 118.8 (q, J = 318.1 Hz), 90.1, 16.4 ppm; IR (thin film) 3445, 2367, 1629, 1476, 1424, 1383, 1245, 1218, 1143, 1100, 903, 810, 611 (cm^{-1}); HRMS (APCI) m/z: calcd for $C_8H_5F_3IO_3S$ [M - H]$^-$, 364.8962; found 364.8947.

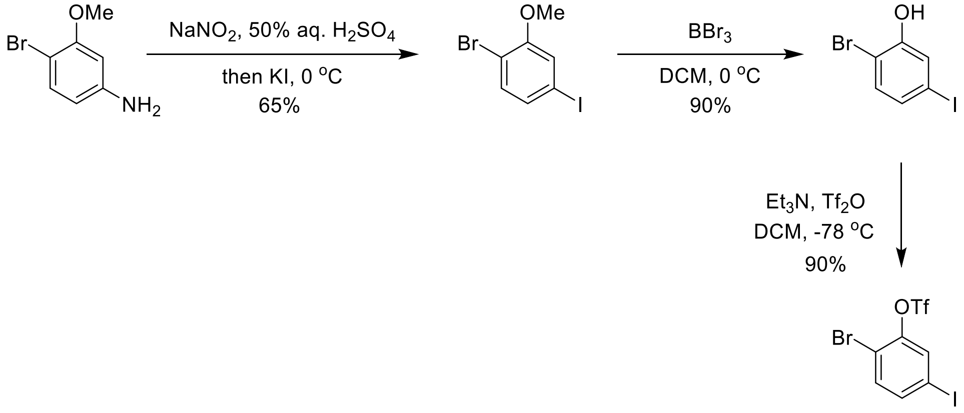

2-Bromo-5-iodophenyl triflate: To a solution of commercially available 4-bromo-3-methoxyaniline (1.0 g, 5.0 mmol, 1.0 equiv) in 50% aq. H_2SO_4 (20 mL) at 0 ℃ was added $NaNO_2$ (379.5 mg, 5.5 mmol, 1.1 equiv) in portions. After stirred for 20 minutes, the resulting solution was slowly poured into a solution of KI (1.66 g, 10.00 mmol, 2.0 equiv) in water (30 mL) at 0 ℃. It was extracted with EtOAc (20 mL x 3). The combined organic layers were washed with brine (20 mL), dried over Na_2SO_4, filtered, and concentrated. Flash column chromatography with pet ether afforded 1.0 g (65%) of 1-bromo-4-iodo-2-methoxybenzene as a colorless oil, the ^{1}H NMR of which is identical with that reported in literature.[2] ^{1}H NMR (400 MHz, $CDCl_3$) δ 7.28-7.22 (m, 1H), 7.18-7.12 (m, 2H), 3.88 (s, 3H) ppm.

To a solution of 1-bromo-4-iodo-2-methoxybenzene (1.0 g, 3.17 mmol, 1.0 equiv) in anhydrous DCM (20 mL) at 0 ℃ was added BBr_3 (1.19 mL, 15.87 mmol, 5.0 equiv) slowly. After stirred for three hours at 0 ℃, water was slowly added to quench the reaction. The resulting mixture was extracted with EtOAc (20 mL x 3). The combined organic layers were washed with brine (20 mL), dried over Na_2SO_4, filtered, and concentrated. Flash column chromatography with pet ether afforded 850 mg (90%) of 2-bromo-5-iodophenol as a colorless oil, the ^1H NMR of which is identical with that reported in literature.[3] ^1H NMR (400 MHz, CD_3OD) δ 7.24 (d, J = 2.0 Hz, 1H), 7.19 (d, J = 8.3 Hz, 1H), 7.03 (dd, J_1 = 8.3 Hz, J_2 = 2.0 Hz, 1H) ppm.
To a solution of 2-bromo-5-iodophenol (0.8 g, 2.68 mmol, 1.0 equiv) in anhydrous DCM (20 mL) at -78 ℃ was added Et_3N (0.56 mL, 4.03 mmol, 1.5 equiv). After stirred for 5 minutes, Tf_2O (0.50 mL, 2.95 mmol, 1.1 equiv) was added slowly. After 10 minutes, all the volatiles were directly removed on a rotary evaporator. Flash column chromatography on silica gel afforded 1.04 g (90%) of 2-bromo-5-iodophenyl triflate as a colorless oil. ^1H NMR (400 MHz, $CDCl_3$) δ 7.65 (d, J = 1.6 Hz, 1H), 7.57 (dd, J_1 = 8.0 Hz, J_2 = 1.6 Hz 1H), 7.39 (d, J = 8.4 Hz, 1H), ppm; ^{13}C NMR (100 MHz, $CDCl_3$) δ 147.1, 138.8, 135.7, 132.0, 118.7 (q, J = 319.0 Hz), 116.3, 92.0 ppm; IR (thin film) 3450, 3088, 1560, 1463, 1430, 1377, 1247, 1216, 1174, 1137, 1071, 1030, 899, 814, 788, 755, 677, 631, 598, 518, 486, 437 (cm^{-1}); HRMS (APCI) m/z: calcd for $C_7H_2BrF_3IO_3S$ $[M - H]^-$, 430.7890; found 430.7890.

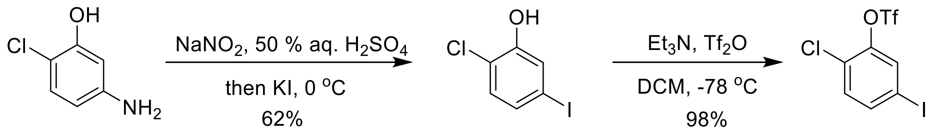

2-Chloro-5-iodophrnyl triflate: To a solution of commercially available 5-amino-2-chlorophenol (1.0 g, 6.99 mmol, 1.0 equiv) in 50% aq. H_2SO_4 (20 mL) at 0 ℃ was added $NaNO_2$ (530.5 mg, 7.69 mmol, 1.1 equiv) in portions. After stirred for 20 minutes, the resulting solution was slowly poured into a solution of KI (2.32 g, 13.98 mmol, 2.0 equiv) in water (30 mL) at 0 ℃. It was extracted with EtOAc (20 mL x 3). The combined organic layers were washed with brine (20 mL), dried over Na_2SO_4, filtered, and concentrated. Flash column chromatography with pet ether afforded 1.10 g (62%) of 2-chloro-5-iodophenol as a colorless oil, the ^1H NMR of which is identical with that reported in literature.[4] ^1H NMR (400 MHz, $CDCl_3$) δ 7.54 (d, J = 2.0 Hz, 1H), 7.37 (dd, J_1 = 8.4 Hz, J_2 = 2.0 Hz, 1H), 7.19 (d, J = 2.0 Hz, 1H), 5.68 (s, 1H) ppm.
To a solution of 2-chloro-5-iodophenol (1.0 g, 3.94 mmol, 1.0 equiv) in anhydrous DCM (20 mL) at -78 ℃ was added Et_3N (0.82 mL, 5.91 mmol, 1.5 equiv). After stirred for 5 minutes, Tf_2O (0.73 mL, 4.33 mmol, 1.1 equiv) was added slowly. After 10 minutes, all the volatiles were directly removed on a rotary evaporator. Flash column chromatography on silica gel afforded 1.49 g (98%) of 2-chloro-5-iodophenyl triflate as a colorless oil. ^1H NMR (400 MHz, $CDCl_3$) δ 7.66-7.64 (m, 2H), 7.24 (d, J = 8.0 Hz, 1H) ppm; ^{13}C NMR (100 MHz, $CDCl_3$) δ 145.7, 138.6, 132.6, 132.1 (d, J =

4.7 Hz), 127.7, 118.7 (q, J = 318.9 Hz), 91.0 ppm; IR (thin film) 3447, 3091, 1560, 1468, 1431, 1382, 1247, 1216, 1179, 1137, 1070, 1048, 900, 790, 758, 683, 634, 601, 495 (cm^{-1}); HRMS (APCI) m/z: calcd for $C_7H_2ClF_3IO_3S$ $[M - H]^-$, 384.8415; found 384.8416.

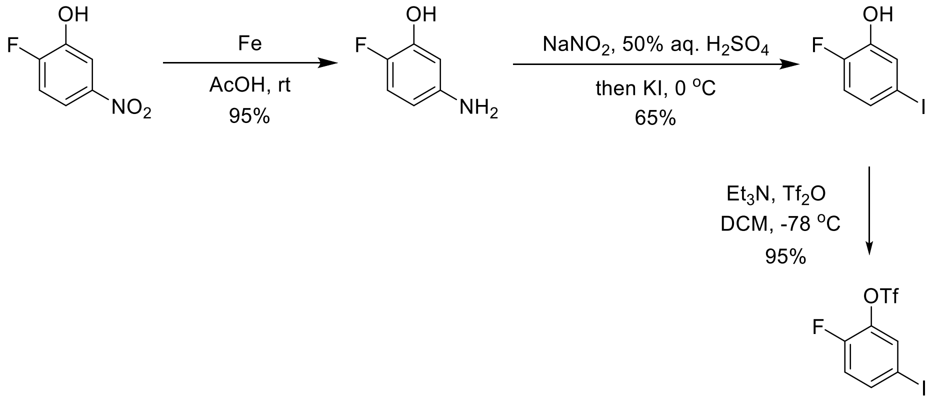

2-Fluoro-5-iodophenyl triflate: To a solution of commercially available 2-fluoro-5-nitrophenol (1.0 g, 6.37 mmol, 1.0 equiv) in acetic acid (20 mL) at room temperature was added iron powder (1.78 g, 31.85 mmol, 5.0 equiv). After stirred for three hours, the residue iron powder was filtered out. Water (20 mL) was added to the mother liquor and it was extracted with EtOAc (20 mL x 3). The combined organic layers were washed with brine (20 mL), dried over Na_2SO_4, filtered, and concentrated. Flash column chromatography with pet ether and EtOAc afforded 769 mg (95%) of 5-amino-2-fluorophenol as a colorless oil, the ^1H NMR of which is identical with that reported in literature.[5] ^1H NMR (400 MHz, CD_3OD) δ 6.78 (m, 1H), 6.34 (m, 1H), 6.13 (m, 1H) ppm.

To a solution of 5-amino-2-fluorophenol (700.0 mg, 5.51 mmol, 1.0 equiv) in 50% aq. H_2SO_4 (20 mL) at 0 ℃ was added $NaNO_2$ (418.3 mg, 6.06 mmol, 1.1 equiv) in portions. After stirred for 20 minutes, the resulting solution was slowly poured into a solution of KI (1.83 g, 11.02 mmol, 2.0 equiv) in water (30 mL) at 0 ℃. It was extracted with EtOAc (20 mL x 3). The combined organic layers were washed with brine (20 mL), dried over Na_2SO_4, filtered, and concentrated. Flash column chromatography with pet ether afforded 852.4 mg (65%) of 2-fluoro-5-iodophenol as a colorless oil, the ^1H NMR of which is identical with that reported in literature.[6] ^1H NMR (400 MHz, $CDCl_3$) δ 6.49 (dd, J_1 = 9.2 Hz, J_2 = 2.0 Hz, 1H), 6.37 (dd, J_1 = 12.8 Hz, J_2 = 8.0 Hz, 1H), 6.00 (m, 1H) ppm.

To a solution of 2-fluoro-5-iodophenol (650 mg, 2.74 mmol, 1.0 equiv) in anhydrous DCM (20 mL) at -78 ℃ was added Et_3N (0.57 mL, 4.11 mmol, 1.5 equiv). After stirred for 5 minutes, Tf_2O (0.51 mL, 3.0 mmol, 1.1 equiv) was then added slowly. After 10 minutes, all the volatiles were directly removed on a rotary evaporator. Flash column chromatography on silica gel afforded 0.96 g (95%) of 2-fluoro-5-iodophenyl triflate as a colorless oil. ^1H NMR (400 MHz, $CDCl_3$) δ 7.69-7.64 (m, 2H), 7.03 (t, J = 9.2 Hz, 1H) ppm; ^{13}C NMR (100 MHz, $CDCl_3$) δ 154.0 (d, J = 253.6 Hz), 139.0 (d, J = 6.7 Hz), 137.2 (d, J = 13.9 Hz), 132.6, 119.6 (d, J = 16.2 Hz), 118.8 (q, J = 318.8

Hz), 86.3 (d, J = 4.4 Hz) ppm; IR (thin film) 3444, 3102, 1595, 1490, 1432, 1273, 1217, 1163, 1137, 1110, 905, 868, 815, 753, 695, 608, 507, 478 (cm^{-1}); HRMS (APCI) m/z: calcd for $C_7H_2F_4IO_3S$ $[M - H]^-$, 368.8711; found 368.8711.

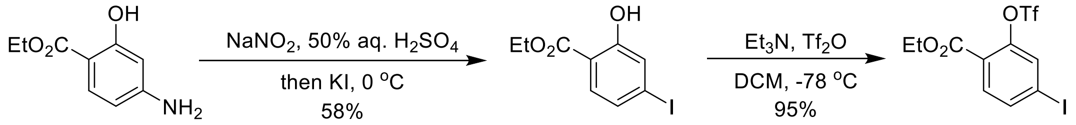

Ethyl 4-iodo-2-(((trifluoromethyl)sulfonyl)oxy)benzoate: To a solution of commercially available ethyl 4-amino-2-hydroxybenzoate (1.0 g, 5.52 mmol, 1.0 equiv) in 50% aq. H_2SO_4 (20 mL) at 0 ℃was added $NaNO_2$ (418.9 mg, 6.1 mmol, 1.1 equiv) in portions. After stirred for 20 minutes, the resulting solution was slowly poured into a solution of KI (1.82 g, 11.0 mmol, 2.0 equiv) in water (30 mL) at 0 ℃. It was extracted with EtOAc (20 mL x 3). The combined organic layers were washed with brine (20 mL), dried over Na_2SO_4, filtered, and concentrated. Flash column chromatography with pet ether afforded 934.9 mg (58%) of ethyl 2-hydroxy-4-iodobenzoate as a colorless oil, the ^1H NMR of which is identical with that reported in literature.[7] ^1H NMR (400 MHz, $CDCl_3$) δ 10.85 (s, 1H), 7.52 (d, J = 8.4 Hz, 1H), 7.40 (d, J = 1.6 Hz, 1H), 7.23 (dd, J_1 = 8.4 Hz, J_2 = 1.6 Hz 1H), 4.40 (q, J = 7.2 Hz, 2H), 1.41 (t, J = 7.2 Hz, 3H) ppm.

To a solution of ethyl 2-hydroxy-4-iodobenzoate (900 mg, 3.08 mmol, 1.0 equiv) in anhydrous DCM (20 mL) at -78 ℃was added Et_3N (0.64 mL, 4.62 mmol, 1.5 equiv). After stirred for 5 minutes, Tf_2O (0.57 mL, 3.39 mmol, 1.1 equiv) was then added slowly. After 10 minutes, all the volatiles were directly removed on a rotary evaporator. Flash column chromatography on silica gel afforded 1.20 g (95%) of ethyl 4-iodo-2-(((trifluoromethyl)sulfonyl)oxy)benzoate as a colorless oil. ^1H NMR (400 MHz, $CDCl_3$) δ 7.83-7.76 (m, 2H), 7.63 (s, 1H), 4.42 (q, J = 7.2 Hz, 2H), 1.39 (t, J = 7.2 Hz, 3H) ppm; ^{13}C NMR (100 MHz, $CDCl_3$) δ 163.5, 147.9, 138.0, 133.7, 131.9, 124.5, 118.8 (q, J = 318.9 Hz), 99.4, 62.6, 14.2 ppm; IR (thin film) 3438, 2990, 1722, 1594, 1473, 1422, 1386, 1284, 1243, 1211, 1155, 1136, 1088, 1016, 909, 868, 847, 767, 631, 598 (cm^{-1}); HRMS (APCI) m/z: calcd for $C_{10}H_9F_3IO_5S$ $[M + H]^+$ 424.9162; found 424.9156.

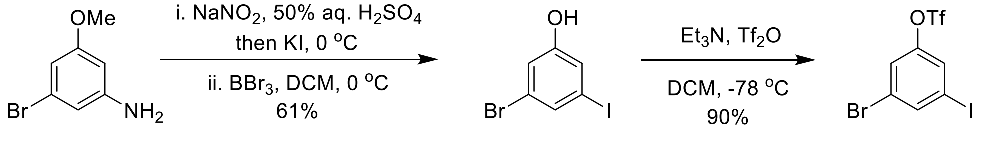

3-Bromo-5-iodophenyl triflate: To a solution of commercially available 3-bromo-5-methoxyaniline (1.0 g, 4.98 mmol, 1.0 equiv) in 50% aq. H_2SO_4 (20 mL) at 0 ℃was added $NaNO_2$ (378 mg, 5.48 mmol, 1.1 equiv) in portions. After stirred for 20 minutes, the resulting solution was slowly poured into a solution of KI (1.65 g, 9.96 mmol, 2.0 equiv) in water (30 mL) at 0 ℃. It was extracted with EtOAc (20 mL x

3). The combined organic layers were washed with brine (20 mL), dried over Na_2SO_4, filtered, and concentrated. A crude oil was obtained, which was used directly in the next step without further purification.

To the above crude oil in anhydrous DCM (20 mL) at 0 ℃ was added BBr_3 (1.20 mL, 16.08 mmol, 5.0 equiv) slowly. After stirred for three hours at 0 ℃, water was slowly added into the reaction. It was extracted with EtOAc (20 mL x 3). The combined organic layers were washed with brine (20 mL), dried over Na_2SO_4, filtered, and concentrated. Flash column chromatography with pet ether afforded 0.84 g (61%) of 3-bromo-5-iodophenol as a colorless oil, the ^{1}H NMR of which is identical with that reported in literature.[8] ^{1}H NMR (400 MHz, $CDCl_3$) δ 7.43 (d, J = 1.3 Hz, 1H), 7.17-7.12 (m, 1H), 6.99-6.95 (m, 1H) ppm.

To a solution of 3-bromo-5-iodophenol (800 mg, 2.68 mmol, 1.0 equiv) in anhydrous DCM (20 mL) at -78 ℃ was added Et_3N (0.56 mL, 4.03 mmol, 1.5 equiv). After stirred for 5 minutes, Tf_2O (0.50 mL, 2.95 mmol, 1.1 equiv) was added in portions. After 10 minutes, all the volatiles were directly removed on a rotary evaporator. Flash column chromatography on silica gel afforded 1.04 g (90%) of 3-bromo-5-iodophenyl triflate as a colorless oil. ^{1}H NMR (400 MHz, $CDCl_3$) δ 7.91 (s, 1H), 7.58 (s, 1H), 7.43 (s, 1H) ppm; ^{13}C NMR (100 MHz, $CDCl_3$) δ 149.1, 140.3, 129.5, 124.6, 123.8, 118.8 (q, J = 319.0 Hz), 94.2 ppm; IR (thin film) 3447, 3085, 1569, 1428, 1407, 1250, 1216, 1161, 1138, 915, 857, 796, 758, 727, 605 (cm^{-1}); HRMS (APCI) m/z: calcd for $C_7H_2BrF_3IO_3S$ [M - H]$^-$, 428.7910; found 428.7909.

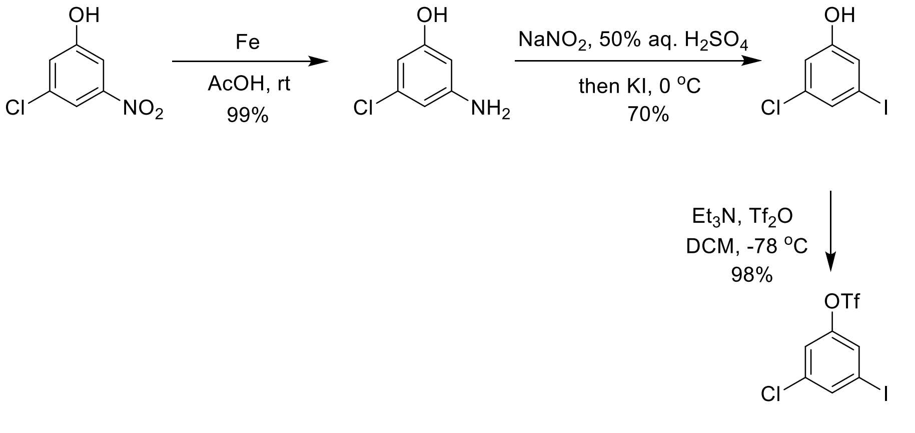

3-Chloro-5-iodophenyl triflate: To a solution of commercially available 3-chloro-5-nitrophenol (1.0 g, 5.78 mmol, 1.0 equiv) in acetic acid (20 mL) at room temperature was added iron powder (1.62 g, 28.90 mmol, 5.0 equiv). After stirred for three hours, the residue iron powder was filtered out. Water (20 mL) was added to the mother liquor and it was extracted with EtOAc (20 mL x 3). The combined organic layers were washed with brine (20 mL), dried over Na_2SO_4, filtered, and concentrated. Flash column chromatography (pet ether:EtOAc = 8:1) afforded 818 mg (99%) of 3-amino-5-chlorophenol as a colorless oil, the ^{1}H NMR of which is identical with that reported in literature.[9] ^{1}H NMR (400 MHz, DMSO-*d6*) δ 9.32 (s, 1H), 6.02 (m, 2H), 5.92 (m ,2H), 5.27 (s, 2H) ppm.

To a solution of 3-amino-5-chlorophenol (0.8 g, 5.59 mmol, 1.0 equiv) in 50% aq. H_2SO_4 (20 mL) at 0 ℃ was added $NaNO_2$ (424 mg, 6.15 mmol, 1.1 equiv) in portions. After stirred for 20 minutes, the resulting solution was slowly poured into a solution of KI (1.86 g, 11.18 mmol, 2.0 equiv) in water (30 mL) at 0 ℃. It was extracted with EtOAc (20 mL x 3). The combined organic layers were washed with brine (20 mL), dried over Na_2SO_4, filtered, and concentrated. Flash column chromatography with pet ether afforded 0.99 g (70%) of 3-chloro-5-iodophenol as a colorless oil, the ^{1}H NMR of which is identical with that reported in literature.[10] ^{1}H NMR (400 MHz, $CDCl_3$) δ 7.29 (s, 1H), 7.13 (s, 1H), 6.89-6.80 (m, 1H), 5.91 (s, 1H) ppm.

To a solution of 3-chloro-5-iodophenol (900 mg, 3.54 mmol, 1.0 equiv) in anhydrous DCM (20 mL) at -78 ℃ was added Et_3N (0.74 mL, 5.31 mmol, 1.5 equiv). After stirred for 5 minutes, Tf_2O (0.65 mL, 3.89 mmol, 1.1 equiv) was then added slowly. After 10 minutes, all the volatiles were directly removed on a rotary evaporator. Flash column chromatography on silica gel afforded 1.34 g (98%) of 3-chloro-5-iodophenyl triflate a colorless oil. ^{1}H NMR (400 MHz, $CDCl_3$) δ 7.76 (m, 1H), 7.54-7.53 (m, 1H), 7.29-7.28 (m, 1H) ppm; ^{13}C NMR (100 MHz, $CDCl_3$) δ 149.2, 137.7, 136.5, 129.1, 121.9, 118.8 (q, J = 319.2 Hz), 93.7 ppm; IR (thin film) 3432, 3094, 2937, 2851, 1573, 1430, 1409, 1251, 1218, 1166, 1139, 1091, 927, 857, 805, 761, 747, 672, 645, 605, 567, 512 (cm^{-1}); HRMS (APCI) m/z: calcd for $C_7H_2ClF_3IO_3S$ [M - H]$^-$, 384.8415; found 384.8418.

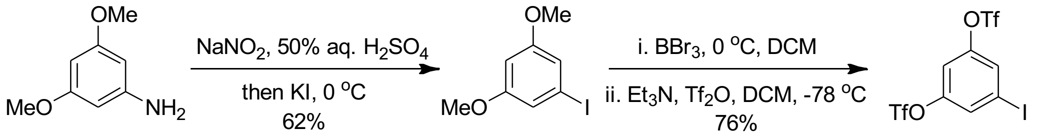

5-Iodo-1,3-phenylene bis(triflate): To a solution of commercially available 3,5-dimethoxyaniline (1.0 g, 6.53 mmol, 1.0 equiv) in 50% aq. H_2SO_4 (20 mL) at 0 ℃ was added $NaNO_2$ (496 mg, 7.19 mmol, 1.1 equiv) in portions. After stirred for 20 minutes, the resulting solution was slowly poured into a solution of KI (2.17 g, 13.06 mmol, 2.0 equiv) in water (30 mL) at 0 ℃. It was extracted with EtOAc (20 mL x 3). The combined organic layers were washed with brine (20 mL), dried over Na_2SO_4, filtered, and concentrated. Flash column chromatography with pet ether afforded 1.07 g (62%) of 1-iodo-3,5-dimethoxybenzene as a colorless oil, the ^{1}H NMR of which is identical with that reported in literature.[11] ^{1}H NMR (400 MHz, $CDCl_3$) δ 6.85 (d, J = 2.2 Hz, 2H), 6.40 (t, J = 2.2 Hz, 1H), 3.76 (s, 6H) ppm.

To a solution of 1-iodo-3,5-dimethoxybenzene (1.0 g, 3.79 mmol, 1.0 equiv) in anhydrous DCM (20 mL) at 0 ℃ was added BBr_3 (2.84 mL, 37.90 mmol, 10.0 equiv) slowly. After stirred for three hours at 0 ℃, water (10 mL) was slowly added into the reaction. It was extracted with EtOAc (20 mL x 3). The combined organic layers were washed with brine (20 mL), dried over Na_2SO_4, filtered, and concentrated. A crude oil was obtained, which was used directly in the next step without further purification.

To the above crude oil in anhydrous DCM (20 mL) at -78 ℃ was added Et_3N (0.62 mL, 4.45 mmol, 1.5 equiv). After stirred for 5 minutes, Tf_2O (0.55 mL, 3.27 mmol,

1.1 equiv) was then added slowly. After 10 minutes, all the volatiles were directly removed on a rotary evaporator. Flash column chromatography on silica gel afford 1.44 g (76%) of 5-iodo-1,3-phenylene bis(triflate) as a colorless oil, the ^{1}H NMR of which is identical with that reported in literature.[12] ^{1}H NMR (400 MHz, $CDCl_3$) δ 7.70 (d, *J* = 1.8 Hz, 2H), 7.25 (t, *J* = 1.8 Hz, 1H) ppm.

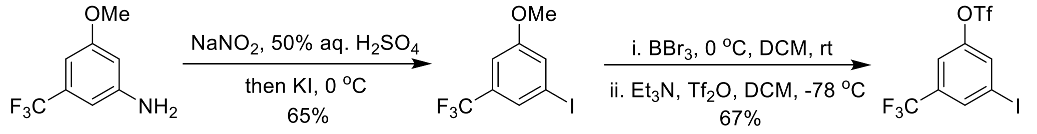

3-Iodo-5-(trifluoromethyl)phenyl triflate: To a solution of commercially available 3-methoxy-5-(trifluoromethyl)aniline (1.0 g, 5.24 mmol, 1.0 equiv) in 50% aq. H_2SO_4 (20 mL) at 0 ℃ was added $NaNO_2$ (397 mg, 5.76 mmol, 1.1 equiv) in portions. After stirred for 20 minutes, the resulting solution was slowly poured into a solution of KI (1.74 g, 10.48 mmol, 2.0 equiv) in water (30 mL) at 0 ℃. It was extracted with EtOAc (20 mL x 3). The combined organic layers were washed with brine (20 mL), dried over Na_2SO_4, filtered, and concentrated. Flash column chromatography with pet ether afforded 1.03 g (65%) of 1-iodo-3-methoxy-5-(trifluoromethyl)benzene as a colorless oil, the ^{1}H NMR of which is identical with that reported in literature.[13] ^{1}H NMR (400 MHz, CDCl3) δ 7.53 (s, 1H), 7.41 (s, 1H), 7.09 (s, 1H), 3.83 (s, 3H) ppm.

To a solution of 1-iodo-3-methoxy-5-(trifluoromethyl)benzene (1.0 g, 3.31 mmol, 1.0 equiv) in anhydrous DCM (20 mL) at 0 ℃ was added BBr_3 (1.24 mL, 16.56 mmol, 5.0 equiv) slowly. After stirred for three hours at 0 ℃, water (10 mL) was slowly added into the reaction. It was extracted with EtOAc (20 mL x 3). The combined organic layers were washed with brine (20 mL), dried over Na_2SO_4, filtered, and concentrated. A crude oil was obtained, which was used directly in the next step without further purification.

To the above crude oil in anhydrous DCM (20 mL) at -78 ℃ was added Et_3N (0.50 mL, 3.64 mmol, 1.5 equiv). After stirred for 5 minutes, Tf_2O (0.45 mL, 2.67 mmol, 1.1 equiv) was added slowly. After 10 minutes, all the volatiles were directly removed on a rotary evaporator. Flash column chromatography on silica gel afforded 0.93 g (67%) of 3-iodo-5-(trifluoromethyl)phenyl triflate as a colorless oil. ^{1}H NMR (400 MHz, $CDCl_3$) δ 8.01 (s, 1H), 7.83 (s, 1H), 7.52 (s, 1H) ppm; ^{13}C NMR (100 MHz, $CDCl_3$) δ 149.2, 134.8 (q, *J* = 3.6 Hz), 134.5, 134.2, 133.8, 122.0 (q, *J* = 271.9 Hz), 118.9 (q, *J* = 318.8 Hz), 118.6 (q, *J* = 3.7 Hz), 94.3 ppm; IR (thin film) 3441, 3085, 1774, 1580, 1453, 1431, 1315, 1220, 1139, 1089, 929, 880, 814, 776, 755, 690, 666, 608, 570, 522, 454 (cm^{-1}); HRMS (APCI) m/z: calcd for $C_8H_2F_6IO_3S$ $[M - H]^-$ 418.8679; found 418.8680.

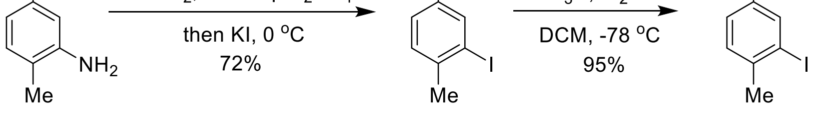

3-Iodo-4-methylphenyl triflate: To a solution of commercially available 3-amino-4-methylphenol (1.0 g, 8.13 mmol, 1.0 equiv) in 50% aq. H_2SO_4 (20 mL) at 0 ℃ was added $NaNO_2$ (903 mg, 8.94 mmol, 1.1 equiv) in portions. After stirred for 20 minutes, the resulting solution was slowly poured into a solution of KI (2.70 g, 16.26 mmol, 2.0 equiv) in water (30 mL) at 0 ℃. It was extracted with EtOAc (20 mL x 3). The combined organic layers were washed with brine (20 mL), dried over Na_2SO_4, filtered, and concentrated. Flash column chromatography with pet ether afforded 1.37 g (72%) of 3-iodo-4-methylphenol as a colorless oil, the ^{1}H NMR of which is identical with that reported in literature.[14] ^{1}H NMR (400 MHz, DMSO-*d*6) δ 9.50 (s, 1H), 7.23 (d, J = 2.0 Hz, 1H), 7.11 (d, J = 8.0 Hz, 1H), 6.71 (dd, J_1 = 8.0 Hz, J_2 = 2.0 Hz 1H), 2.25 (s, 3H) ppm.

To a solution of 3-iodo-4-methylphenol (1.0 g, 4.27 mmol, 1.0 equiv) in anhydrous DCM (20 mL) at -78 ℃ was added Et_3N (0.84 mL, 3.64 mmol, 1.5 equiv). After stirred for 5 minutes, Tf_2O (1.19 mL, 7.05 mmol, 1.1 equiv) was added slowly. After 10 minutes, all the volatiles were directly removed on a rotary evaporator. Flash column chromatography on silica gel afforded 1.48 g (95%) of 3-iodo-4-methylphenyl triflate as a colorless oil. ^{1}H NMR (400 MHz, $CDCl_3$) δ 7.71 (d, J = 2.4 Hz, 1H), 7.30 (d, J = 8.4 Hz, 1H), 7.19 (dd, J_1 = 8.4 Hz, J_2 = 2.4 Hz, 1H), 2.46 (s, 3H) ppm; ^{13}C NMR (100 MHz, $CDCl_3$) δ 147.0, 142.5, 131.5, 130.5, 121.1, 118.9 (q, J = 318.8 Hz), 100.5, 27.7 ppm; IR (thin film) 3458, 1583, 1475, 1426, 1378, 1248, 1215, 1141, 1027, 900, 822, 727, 614, 504 (cm^{-1}); HRMS (APCI) m/z: calcd for $C_8H_5F_3IO_3S$ [M - H]$^-$, 364.8962; found 364.8958.

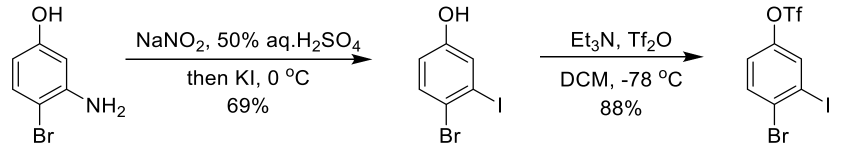

4-Bromo-3-iodophenyl triflate: To a solution of commercially available 3-amino-4-bromophenol (1.0 g, 5.32 mmol, 1.0 equiv) in 50% aq. H_2SO_4 (20 mL) at 0 ℃ was added $NaNO_2$ (403 mg, 5.85 mmol, 1.1 equiv) in portions. After stirred for 20 minutes, the resulting solution was slowly poured into a solution of KI (1.76 g, 10.64 mmol, 2.0 equiv) in water (30 mL) at 0 ℃. It was extracted with EtOAc (20 mL x 3). The combined organic layers were washed with brine (20 mL), dried over Na_2SO_4, filtered, and concentrated. Flash column chromatography with pet ether afforded 1.09 g (69%) of 4-bromo-3-iodophenol as a colorless oil, the ^{1}H NMR of which is identical with that reported in literature.[15] ^{1}H NMR (400 MHz, $CDCl_3$) δ 7.43 (d, J = 8.6 Hz, 1H), 7.37 (d, J = 2.7 Hz, 1H), 6.71 (dd, J_1 = 8.8 Hz, J_2 = 3.0 Hz, 1H), 4.91 (s, 1H) ppm.

To a solution of 4-bromo-3-iodophenol (1.0 g, 3.36 mmol, 1.0 equiv) in anhydrous DCM (20 mL) at -78 ℃ was added Et_3N (0.66 mL, 5.03 mmol, 1.5 equiv). After stirred for 5 minutes, Tf_2O (0.62 mL, 3.70 mmol, 1.1 equiv) was then added slowly. After 10 minutes, all the volatiles were directly removed on a rotary evaporator. Flash

column chromatography on silica gel afforded 1.27 g (88%) of 4-bromo-3-iodophenyl triflate as a colorless oil. ^{1}H NMR (400 MHz, $CDCl_3$) δ 7.76 (d, J = 2.4 Hz, 1H), 7.69 (d, J = 8.8 Hz, 1H), 7.16 (dd, J_1 = 8.8 Hz, J_2 = 2.8 Hz, 1H) ppm; ^{13}C NMR (100 MHz, $CDCl_3$) δ 147.7, 133.7, 133.1, 130.2, 122.6, 118.8 (q, J = 319.2 Hz), 101.8 ppm; IR (thin film) 3435, 3091, 1569, 1428, 1381, 1243, 1216, 1158, 1139, 1091, 1013, 898, 816, 787, 758, 657, 608, 576, 536, 504, 434 (cm^{-1}); HRMS (APCI) m/z: calcd for $C_7H_2BrF_3IO_3S$ [M - H]$^-$, 430.7890; found 430.7884.

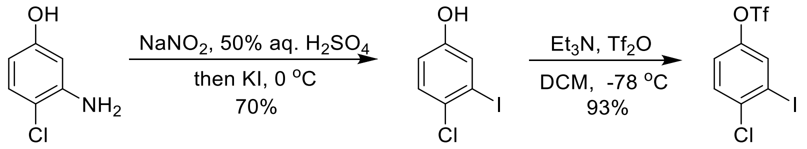

4-Chloro-3-iodophenyl triflate: To a solution of commercially available 3-amino-4-chlorophenol (1.0 g, 6.99 mmol, 1.0 equiv) in 50% aq. H_2SO_4 (20 mL) at 0 ℃ was added $NaNO_2$ (530 mg, 7.69 mmol, 1.1 equiv) in portions. After stirred for 20 minutes, the resulting solution was slowly poured into a solution of KI (2.32 g, 13.98 mmol, 2.0 equiv) in water (30 mL) at 0 ℃. It was extracted with EtOAc (20 mL x 3). The combined organic layers were washed with brine (20 mL), dried over Na_2SO_4, filtered, and concentrated. Flash column chromatography with pet ether afforded 1.24 g (70%) of 4-chloro-3-iodophenol as a colorless oil, the ^{1}H NMR of which is identical with that reported in literature.[16] ^{1}H NMR (400 MHz, $CDCl_3$) δ 7.35 (d, J = 3.0 Hz, 1H), 7.28 (d, J = 8.7 Hz, 1H), 6.78 (dd, J_1 = 8.7 Hz, J_2 = 3.0 Hz, 1H), 4.87 (s ,1H) ppm.

To a solution of 4-chloro-3-iodophenol (1.0 g, 3.93 mmol, 1.0 equiv) in anhydrous DCM (20 mL) at -78 ℃ was added Et_3N (0.78 mL, 5.91 mmol, 1.5 equiv). After stirred for 5 minutes, Tf_2O (0.73 mL, 4.32 mmol, 1.1 equiv) was added slowly. After 10 minutes, all the volatiles were directly removed on a rotary evaporator. Flash column chromatography on silica gel afforded 1.41 g (93%) of 4-chloro-3-iodophenyl triflate as a colorless oil. ^{1}H NMR (400 MHz, $CDCl_3$) δ 7.76 (d, J = 2.4 Hz, 1H), 7.52 (dd, J_1 = 9.2 Hz, J_2 = 2.0 Hz, 1H), 7.25 (dd, J_1 = 9.2 Hz, J_2 = 2.8 Hz, 1H) ppm; ^{13}C NMR (100 MHz, $CDCl_3$) δ 147.1, 139.3, 133.0 (d, J = 3.5 Hz), 130.3, 122.5, 118.8 (q, J = 319.1 Hz), 98.6 ppm; IR (thin film) 3458, 3094, 1574, 1453, 1429, 1381, 1215, 1139, 1109, 1023, 901, 818, 795, 761, 658, 613, 580, 475 (cm^{-1}); HRMS (APCI) m/z: calcd for $C_7H_2ClF_3IO_3S$ [M - H]$^-$, 386.8386; found 386.8388.

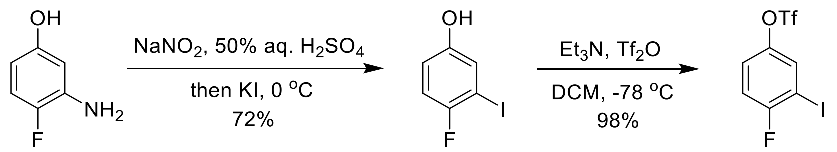

4-Fluoro-3-iodophenyl triflate: To a solution of commercially available 3-amino-4-fluorophenol (1.0 g, 7.87 mmol, 1.0 equiv) in 50% aq. H_2SO_4 (20 mL) at 0 ℃ was added $NaNO_2$ (597 mg, 8.66 mmol, 1.1 equiv) in portions. After stirred for 20 minutes, the resulting solution was slowly poured into a solution of KI (2.61 g,

15.74 mmol, 2.0 equiv) in water (30 mL) at 0 ℃. It was extracted with EtOAc (20 mL x 3). The combined organic layers were washed with brine (20 mL), dried over Na_2SO_4, filtered, and concentrated. Flash column chromatography with pet ether afforded 1.34 g (72%) of 4-fluoro-3-iodophenol as a colorless oil, the ^1H NMR of which is identical with that reported in literature.[17] ^1H NMR (400 MHz, $CDCl_3$) δ 9.65 (s, 1H), 7.18 (m, 1H), 7.05 (m, 1H), 6.80-6.70 (m, 1H) ppm.

To a solution of 4-fluoro-3-iodophenol (1.0 g, 4.22 mmol, 1.0 equiv) in anhydrous DCM (20 mL) at -78 ℃ was added Et_3N (0.83 mL, 6.33 mmol, 1.5 equiv). After stirred for 5 minutes, Tf_2O (0.78 mL, 4.64 mmol, 1.1 equiv) was added slowly. After 10 minutes, all the volatiles were directly removed on a rotary evaporator. Flash column chromatography on silica gel afforded 1.53 g (98%) of 4-fluoro-3-iodophenyl triflate as a colorless oil. ^1H NMR (400 MHz, $CDCl_3$) δ 7.68-7.66 (m, 1H), 7.29-7.25 (m, 1H), 7.15-7.11 (m, 1H) ppm; ^{13}C NMR (100 MHz, $CDCl_3$) δ 161.4 (d, J = 246.5 Hz), 145.2 (d, J = 3.5 Hz), 132.4, 123.3 (d, J = 8.1 Hz), 118.8 (q, J = 319.0 Hz), 116.7 (d, J = 26.7 Hz), 81.8 (d, J = 28.7 Hz) ppm; IR (thin film) 3429, 3100, 1629, 1598, 1479, 1428, 1252, 1215, 1142, 1039, 905, 838, 767, 721, 692, 617, 501, 434 (cm^{-1}); HRMS (APCI) m/z: calcd for $C_7H_2F_4IO_3S$ $[M - H]^-$, 368.8711; found 368.8711.

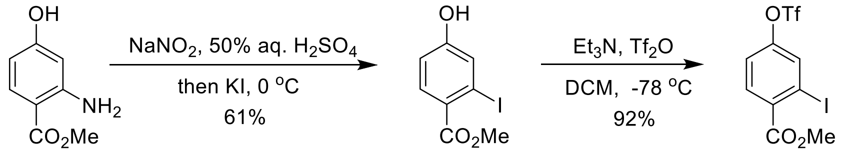

Methyl 2-iodo-4-(((trifluoromethyl)sulfonyl)oxy)benzoate: To a solution of commercially available methyl 2-amino-4-hydroxybenzoate (1.0 g, 5.99 mmol, 1.0 equiv) in 50% aq. H_2SO_4 (20 mL) at 0 ℃ was added $NaNO_2$ (454 mg, 6.59 mmol, 1.1 equiv) in portions. After stirred for 20 minutes, the resulting solution was slowly poured into a solution of KI (1.99 g, 11.98 mmol, 2.0 equiv) in water (30 mL) at 0 ℃. It was extracted with EtOAc (20 mL x 3). The combined organic layers were washed with brine (20 mL), dried over Na_2SO_4, filtered, and concentrated. Flash column chromatography with pet ether afforded 1.02 g (61%) of methyl 4-hydroxy-2-iodobenzoate as a colorless oil. ^1H NMR (400 MHz, DMSO-*d6*) δ 10.6 (s, 1H), 7.70 (d, J = 8.8 Hz, 1H), 7.42 (d, J = 2.0 Hz, 1H), 6.86 (dd, J_1 = 8.8 Hz, J_2 = 2.0 Hz, 1H), 3.77 (s, 3H) ppm; ^{13}C NMR (100 MHz, DMSO-*d6*) δ 165.7, 160.8, 132.7, 127.8, 124.3, 115.1, 96.3, 52.0 ppm; IR (thin film) 3406, 2984, 2923, 2854, 1690, 1614, 1447, 1392, 1351, 1323, 1263, 1209, 1174, 1103, 1030, 972, 918, 863, 805, 764, 643, 621, 593, 530, 426 (cm^{-1}); HRMS (ESI) m/z: calcd for $C_8H_7INaO_3$ $[M + Na]^+$, 300.9332; found 300.9334.

To a solution of methyl 4-hydroxy-2-iodobenzoate (1.0 g, 3.60 mmol, 1.0 equiv) in anhydrous DCM (20 mL) at -78 ℃ was added Et_3N (0.71 mL, 5.40 mmol, 1.5 equiv). After stirred for 5 minutes, Tf_2O (0.67 mL, 3.96 mmol, 1.1 equiv) was added slowly. After 10 minutes, all the volatiles were directly removed on a rotary evaporator. Flash column chromatography on silica gel afforded 1.36 g (92%) of methyl 2-iodo-4-(((trifluoromethyl)sulfonyl)oxy)benzoate as a colorless oil. ^1H NMR (400

MHz, $CDCl_3$) δ 7.91-7.89 (m, 2H), 7.35 (dd, J_1 = 8.8 Hz, J_2 = 2.4 Hz, 1H), 3.95 (s, 3H) ppm; ^{13}C NMR (100 MHz, $CDCl_3$) δ 165.7, 150.5, 135.4, 134.2, 132.5, 121.1, 118.8 (q, J = 318.6 Hz), 94.7, 53.1 ppm; IR (thin film) 3455, 3105, 2955, 2929, 2851, 1736, 1585, 1473, 1430, 1382, 1293, 1247, 1216, 1141, 1115, 1034, 964, 901, 836, 781, 754, 683, 657, 609, 585, 521, 443 (cm^{-1}); HRMS (APCI) m/z: calcd for $C_9H_5F_3IO_5S$ $[M + H]^+$, 410.9006; found 410.8998.

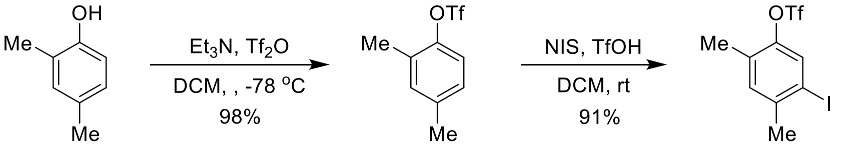

5-Iodo-2,4-dimethylphenyl triflate: To a solution of commercially available 2,4-dimethylphenol (2.0 g, 16.39 mmol, 1.0 equiv) in anhydrous DCM (30 mL) at -78 ℃ was added Et_3N (3.40 mL, 24.59 mmol, 1.5 equiv). After stirred for 5 minutes, Tf_2O (4.13 mL, 24.59 mmol, 1.1 equiv) was added slowly. After 10 minutes, all the volatiles were directly removed on a rotary evaporator. Flash column chromatography on silica gel afforded 4.08 g (98%) of 2,4-dimethylphenyl triflate as a colorless oil, the ^1H NMR of which is identical with that reported in literature.[18] ^1H NMR (400 MHz, $CDCl_3$) δ 7.12-7.10 (m, 2H), 7.05-7.03 (m, 1H), 2.34 (s, 3H), 2.33 (s, 3H) ppm. To a solution of 2,4-dimethylphenyl triflate (2.0 g, 7.87 mmol, 1.0 equiv) in anhydrous DCM (30 mL) at room temperature was added NIS (2.66 g, 11.81 mmol, 1.5 equiv). Triflic acid (TfOH) (1.26 mL, 15.74 mmol, 2.0 equiv) was then added slowly. After three hours, all the volatiles were directly removed on a rotary evaporator. Flash column chromatography on silica gel afforded 2.73 g (91%) of 5-iodo-2,4-dimethylphenyl triflate as a colorless oil. ^1H NMR (400 MHz, $CDCl_3$) δ 7.62 (s, 1H), 7.17 (s, 1H), 2.41 (s, 3H), 2.29 (s, 3H) ppm; ^{13}C NMR (100 MHz, $CDCl_3$) δ 146.1, 142.1, 132.6, 131.3, 131.0, 118.8 (q, J = 318.0 Hz), 96.6, 27.5, 16.3 ppm; IR (thin film) 3446, 2932, 1641, 1473, 1423, 1378, 1215, 1142, 1104, 957, 882, 640 (cm^{-1}); HRMS (APCI) m/z: calcd for $C_9H_7F_3IO_3S$ $[M - H]^-$, 378.9118; found 378.9114.

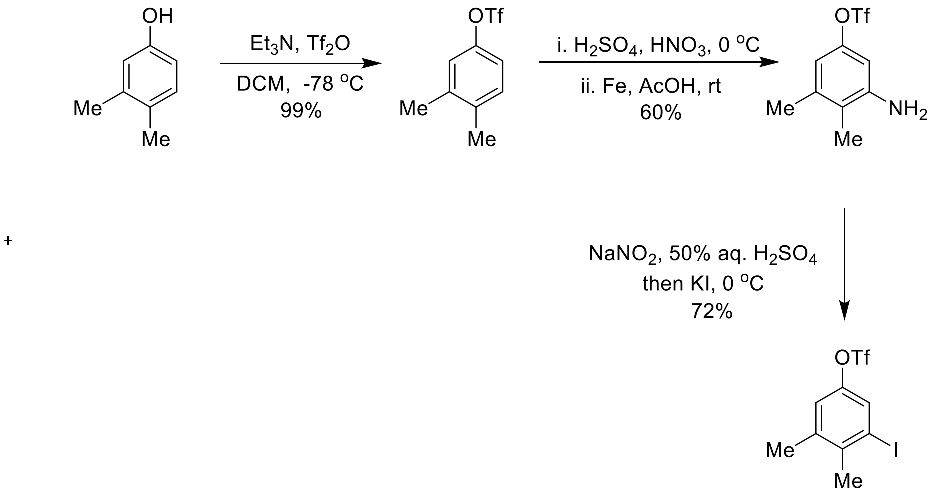

3-Iodo-4,5-dimethylphenyl triflate: To a solution of commercially available 3,4-dimethylphenol (2.0 g, 16.39 mmol, 1.0 equiv) in anhydrous DCM (30 mL) at -78 ℃ was added Et_3N (3.40 mL, 24.59 mmol, 1.5 equiv). After stirred for 5 minutes, Tf_2O (4.13 mL, 24.59 mmol, 1.1 equiv) was added slowly. After 10 minutes, all the volatiles were directly removed on a rotary evaporator. Flash column chromatography on silica gel afforded 4.12 g (99%) of 3,4-dimethylphenyl triflate as a colorless oil, the ^1H NMR of which is identical with that reported in literature.[18] ^1H NMR (400 MHz, $CDCl_3$) δ 7.17 (d, J = 8.4 Hz, 1H), 7.04 (d, J = 2.4 Hz, 1H), 6.99 (dd, J_1= 8.0 Hz, J_2= 2.4 Hz, 1H), 2.29 (s, 3H), 2.27 (s, 3H) ppm.
To a solution of 3,4-dimethylphenyl triflate (3.0 g, 11.81 mmol, 1.0 equiv) in 98% H_2SO_4 (30 mL) at 0 ℃ was added 68% aq. HNO_3 (0.5 mL, 11.81 mmol, 1.0 equiv). After stirred for 20 minutes, it was poured into water (20 mL). The resulting mixture was extracted with DCM (20 mL x 3). The combined organic layers were washed with brine (20 mL), dried over Na_2SO_4, filtered, and concentrated. A crude oil was obtained, which was used directly in the next step without further purification.
To the above crude oil in acetic acid (30 mL) at room temperature was added iron powder (3.31 g, 59.05 mmol, 5.0 equiv). After stirred for six hours, the residue iron powder was filtered out. Water (30 mL) was added to the mother liquor and it was extracted with DCM (30 mL x 3). The combined organic layers were washed with brine (20 mL), dried over Na_2SO_4, filtered, and concentrated. Flash column chromatography on silica gel afforded 1.91 g (60%) of 3-amino-4,5-dimethylphenyl triflate as a colorless oil. ^1H NMR (400 MHz, $CDCl_3$) δ 6.51 (d, J = 2.4 Hz, 1H), 6.44 (d, J = 2.4 Hz, 1H), 3.77 (s, 2H), 2.27 (s, 3H), 2.04 (s, 3H) ppm; ^{13}C NMR (100 MHz, $CDCl_3$) δ 147.9, 146.2, 139.3, 120.9, 118.9 (q, J = 318.8 Hz), 112.4, 105.3, 20.8, 12.7 ppm; IR (thin film) 3479, 2926, 1629, 1486, 1417, 1317, 1244, 1212, 1142, 1065, 1010, 964, 923, 864, 727, 666, 613, 541, 495 (cm^{-1}); HRMS (ESI) m/z: calcd for $C_9H_{11}F_3NO_3S$ $[M + H]^+$, 270.0406; found 270.0396.
To a solution of 3-amino-4,5-dimethylphenyl triflate (1.2 g, 4.46 mmol, 1.0 equiv) in 50% aq. H_2SO_4 (20 mL) at 0 ℃ was added $NaNO_2$ (339 mg, 4.91 mmol, 1.1 equiv) in portions. After stirred for 20 minutes, the reaction solution was slowly poured into a solution of KI (1.48 g, 8.92 mmol, 2.0 equiv) in water (30 mL) at 0 ℃. It was extracted with EtOAc (20 mL x 3). The combined organic layers were washed with brine (20 mL), dried over Na_2SO_4, filtered, and concentrated. Flash column chromatography with pet ether afforded 1.21 g (72%) of methyl 3-iodo-4,5-dimethylphenyl triflate. ^1H NMR (400 MHz, $CDCl_3$) δ 7.58 (d, J = 2.4 Hz, 1H), 7.07 (d, J = 2.0 Hz, 1H), 2.42 (s, 3H), 2.39 (s, 3H) ppm; ^{13}C NMR (100 MHz, $CDCl_3$) δ 146.8, 140.6, 139.3, 129.4, 122.6, 118.9 (q, J = 318.7 Hz), 101.4, 24.9, 22.4 ppm; IR (thin film) 3424, 2862, 1586, 1566, 1463, 1426, 1214, 1175, 1141, 1114, 996, 952, 874, 844, 787, 712, 695, 637, 602, 579, 489 (cm^{-1}); HRMS (ESI) m/z: calcd for $C_9H_7F_3IO_3S$ $[M - H]^-$, 378.9118; found 378.9118.

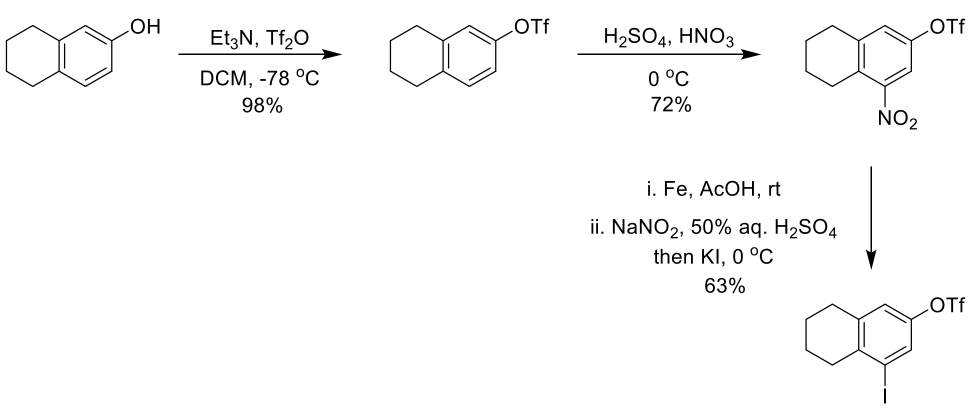

4-Iodo-5,6,7,8-tetrahydronaphthalen-2-yl triflate: To a solution of commercially available 5,6,7,8-tetrahydronaphthalen-2-ol (5.0 g, 33.78 mmol, 1.0 equiv) in anhydrous DCM (50 mL) at -78 ℃ was added Et_3N (7.03 mL, 50.68 mmol, 1.5 equiv). After stirred for 5 minutes, Tf_2O (6.25 mL, 37.16 mmol, 1.1 equiv) was added slowly. After 10 minutes, all the volatiles were directly removed on a rotary evaporator. Flash column chromatography on silica gel afforded 9.27 g (98%) of 5,6,7,8-tetrahydronaphthalen-2-yl triflate as a colorless oil, the ^{1}H NMR of which is identical with that reported in literature.[19] ^{1}H NMR (400 MHz, $CDCl_3$) δ 7.12 (d, J = 8.0 Hz, 1H), 7.00-6.98 (m, 2H), 2.81-2.77 (m, 4H), 1.84-1.78 (m, 4H) ppm.

To a solution of 5,6,7,8-tetrahydronaphthalen-2-yl triflate (9.0 g, 32.14 mmol, 1.0 equiv) in 98% H_2SO_4 (20 mL) at 0 ℃ was added conc. HNO_3 (68%, 2.38 mL, 48.21 mmol, 1.5 equiv). After stirred for 5 minutes, it was poured into water (20 mL). The resulting mixture was extracted with DCM (20 mL x 3). The combined organic layers were washed with brine (20 mL), dried over Na_2SO_4, filtered, and concentrated. Flash column chromatography on silica gel afforded 7.52 g (72%) of 4-nitro-5,6,7,8-tetrahydronaphthalen-2-yl triflate as a colorless oil. ^{1}H NMR (400 MHz, $CDCl_3$) δ 7.61 (d, J = 2.4 Hz, 1H), 7.26 (d, J = 2.4 Hz, 1H), 2.99-2.97 (m, 2H), 2.91-2.89 (m, 2H), 1.85-1.83 (m, 4H) ppm; ^{13}C NMR (100 MHz, $CDCl_3$) δ 150.4, 146.4, 143.3, 132.9, 126.3, 118.9 (q, J = 318.8 Hz), 115.4, 30.4, 26.2, 22.1, 21.7 ppm; IR (thin film) 3427, 2935, 2868, 1612, 1537, 1467, 1428, 1358, 1334, 1224, 1139, 1111, 1010, 973, 952, 874, 832, 770, 715, 651, 602, 536, 504, 472 (cm^{-1}); HRMS (APCI) m/z: calcd for $C_{11}H_9F_3NO_5S$ [M - H]$^-$, 324.0159; found 324.0150.

To a solution of 4-nitro-5,6,7,8-tetrahydronaphthalen-2-yl triflate (7.0 g, 21.53 mmol, 1.0 equiv) in HOAc (50 mL) at room temperature was added iron powder (5.57 g, 99.43 mmol, 5.0 equiv). After stirred for three hours, the residue iron powder was filtered out. Water (50 mL) was added to the mother liquor and it was extracted with DCM (30 mL x 3). The combined organic layers were washed with brine (20 mL), dried over Na_2SO_4, filtered, and concentrated. A crude oil was obtained, which was used directly in the next step without further purification.

To the above crude oil in conc. 50% H_2SO_4 (40 mL) at 0 ℃ was added $NaNO_2$ (1.51 g, 21.88 mmol, 1.1 equiv) in portions. After stirred for 15 minutes, the reaction solution was poured into a solution of KI (6.60 g, 39.78 mmol, 2.0 equiv) in water (30 mL) at 0 ℃. After stirred for 30 minutes at 0 ℃, it was poured into water (20 mL). The

resulting mixture was extracted with DCM (20 mL x 3). The combined organic layers were washed with brine (20 mL), dried over Na_2SO_4, filtered, and concentrated. Flash column chromatography on silica gel afforded 5.51 g (63%) of 4-iodo-5,6,7,8-tetrahydronaphthalen-2-yl triflate as a colorless oil. ^{1}H NMR (400 MHz, $CDCl_3$) δ 7.58 (d, J = 2.4 Hz, 1H), 7.02 (d, J = 2.0 Hz, 1H), 2.78 (t, J = 6.4 Hz, 2H), 2.66 (t, J = 6.4 Hz, 2H), 1.85-1.80 (m, 2H), 1.77-1.73 (m, 2H) ppm; ^{13}C NMR (100 MHz, $CDCl_3$) δ 146.7, 141.1, 140.3, 129.0, 121.8, 118.9 (q, J = 318.4 Hz), 102.6, 35.6, 30.7, 23.9, 22.4 ppm; IR (thin film) 3432, 2937, 2862, 2668, 1590, 1572, 1449, 1425, 1323, 1250, 1215, 1140, 1109, 997, 936, 872, 834, 776, 712, 677, 640, 603, 573, 518, 504, 466 (cm^{-1}); HRMS (APCI) m/z: calcd for $C_{11}H_9F_3IO_3S$ [M - H]$^-$, 404.9275; found 404.9285.

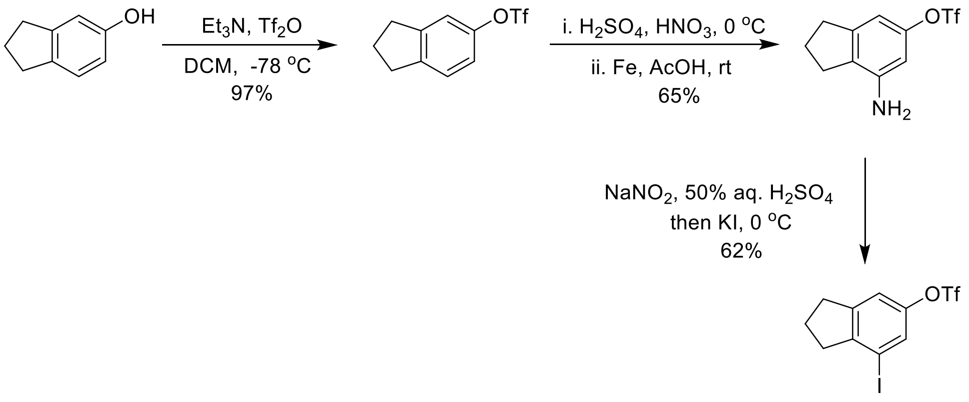

7-Iodo-2,3-dihydro-1*H*-inden-5-yl triflate: To a solution of commercially available 2,3-dihydro-1*H*-inden-5-ol (5.0 g, 37.31 mmol, 1.0 equiv) in anhydrous DCM (50 mL) at -78 ℃ was added Et_3N (7.76 mL, 55.97 mmol, 1.5 equiv). After stirred for 5 minutes, Tf_2O (6.90 mL, 41.04 mmol, 1.1 equiv) was added slowly. After 10 minutes, all the volatiles were directly removed on a rotary evaporator. Flash column chromatography on silica gel afforded 9.62 g (97%) of 2,3-dihydro-1*H*-inden-5-yl triflate as a colorless oil, the ^{1}H NMR of which is identical with that reported in literature. [20] ^{1}H NMR (400 MHz, $CDCl_3$) δ 7.25 (d, J = 7.2 Hz, 1H), 7.12 (d, J = 1.8 Hz, 1H), 7.02 (m, 1H), 2.96 (t, J = 7.4 Hz, 2H), 2.93 (t, J = 7.4 Hz, 2H), 2.45 (q, J = 7.4 Hz, 2H) ppm.

To a solution of 2,3-dihydro-1*H*-inden-5-yl triflate (9.0 g, 33.83 mmol, 1.0 equiv) in 98% H_2SO_4 (20 mL) at 0 ℃ was added conc. HNO_3 (68%, 1.84 mL, 37.22 mmol, 1.1 equiv). After stirred for 5 minutes, it was poured into water (20 mL). The resulting mixture was extracted with DCM (20 mL x 3). The combined organic layers were washed with brine (20 mL), dried over Na_2SO_4, filtered, and concentrated. A crude oil was obtained, which was used directly in the next step without further purification.

To the above crude oil in HOAc (100 mL) at room temperature was added iron powder (9.47 g, 169.15 mmol, 5.0 equiv). After stirred for three hours, the residue iron powder was filtered out. Water (100 mL) was added to the mother liquor and it was extracted with DCM (50 mL x 3). The combined organic layers were washed with brine (100 mL), dried over Na_2SO_4, filtered, and concentrated. Flash column

chromatography on silica gel afforded 6.18 g (65%) of 7-amino-2,3-dihydro-1*H*-inden-5-yl triflate as a colorless oil. ^{1}H NMR (400 MHz, $CDCl_3$) δ 6.56 (s, 1H), 6.37 (s, 1H), 3.72 (s, 2H), 2.91 (t, *J* = 7.6 Hz, 2H), 2.68 (t, *J* = 7.6 Hz, 2H), 2.17-2.09 (m, 2H) ppm; ^{13}C NMR (100 MHz, $CDCl_3$) δ 149.4, 147.3, 143.7, 128.5, 118.9 (q, *J* = 318.6 Hz), 107.0, 104.8, 33.4, 29.0, 24.8 ppm; IR (thin film) 3488, 3400, 3224, 2959, 2842, 1628, 1481, 1453, 1416, 1327, 1211, 1141, 1111, 1085, 1020, 949, 906, 861, 743, 674 (cm^{-1}); HRMS (ESI) m/z: calcd for $C_{10}H_{11}F_3NO_3S$ $[M + H]^+$, 282.0406; found 282.0407.

To a solution of 7-amino-2,3-dihydro-1*H*-inden-5-yl triflate (6.0 g, 21.35 mmol, 1.0 equiv) in 50% aq. H_2SO_4 (40 mL) at 0 ℃ was added $NaNO_2$ (1.62 g, 23.49 mmol, 1.1 equiv) in portions. After stirred for 20 minutes, the reaction solution was slowly poured into a solution of KI (7.09 g, 42.7 mmol, 2.0 equiv) in water (30 mL) at 0 ℃. It was extracted with EtOAc (20 mL x 3). The combined organic layers were washed with brine (20 mL), dried over Na_2SO_4, filtered, and concentrated. Flash column chromatography with pet ether afforded 5.19 g (62%) of methyl 7-iodo-2,3-dihydro-1*H*-inden-5-yl triflate. ^{1}H NMR (400 MHz, $CDCl_3$) δ 7.42 (d, *J* = 2.4 Hz, 1H), 7.10 (s, 1H), 3.11 (t, *J* = 7.6 Hz, 2H), 2.90 (t, *J* = 7.2 Hz, 2H), 2.18-2.10 (m, 2H) ppm; ^{13}C NMR (100 MHz, $CDCl_3$) δ 149.7, 147.8, 146.6, 128.2, 118.9 (q, *J* = 319.0 Hz), 117.4, 92.8, 38.0, 35.0, 24.3 ppm; IR (thin film) 3424, 2949, 2842, 1572, 1450, 1426, 1311, 1251, 1213, 1140, 1085, 1007, 953, 912, 880, 849, 802, 727, 692, 643, 604, 498 (cm^{-1}); HRMS (APCI) m/z: calcd for $C_{10}H_7F_3IO_3S$ $[M - H]^-$, 390.9118; found 390.9123.

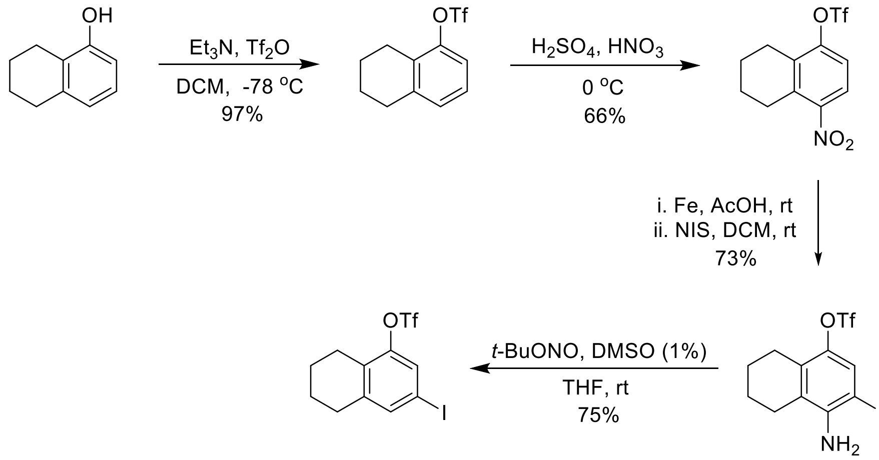

3-Iodo-5,6,7,8-tetrahydronaphthalen-1-yl triflate: To a solution of commercially available 5,6,7,8-tetrahydronaphthalen-1-ol (5.0 g, 33.78 mmol, 1.0 equiv) in anhydrous DCM (50 mL) at -78 ℃ was added Et_3N (7.03 mL, 50.68 mmol, 1.5 equiv). After stirred for 5 minutes, Tf_2O (6.25 mL, 37.16 mmol, 1.1 equiv) was added slowly. After 10 minutes, all the volatiles were directly removed on a rotary evaporator. Flash column chromatography on silica gel afforded 9.17 g (97%) of 5,6,7,8-tetrahydronaphthalen-1-yl triflate as a colorless oil, the ^{1}H NMR of which is

identical with that reported in literature.[21] ^{1}H NMR (400 MHz, $CDCl_3$) δ 7.19-7.07 (m, 3H), 2.85-2.80 (m, 2H), 1.88-1.79 (m, 2H) ppm.

To a solution of 5,6,7,8-tetrahydronaphthalen-1-yl triflate (9.0 g, 32.14 mmol, 1.0 equiv) in 98% H_2SO_4 (20 mL) at 0 ℃ was added conc. HNO_3 (68%, 2.17 mL, 48.21 mmol, 1.5 equiv). After stirred for 5 minutes, it was poured into water (20 mL). The resulting mixture was extracted with DCM (20 mL x 3). The combined organic layers were washed with brine (20 mL), dried over Na_2SO_4, filtered, and concentrated. Flash column chromatography on silica gel afforded 6.89 g (66%) of 4-nitro-5,6,7,8-tetrahydronaphthalen-1-yl triflate as a colorless oil. ^{1}H NMR (400 MHz, $CDCl_3$) δ 7.74 (d, J = 8.8 Hz, 1H), 7.24 (d, J = 8.8 Hz, 1H), 2.98 (t, J = 6.4 Hz, 2H), 2.86 (t, J = 6.4 Hz, 2H), 1.88-1.80 (m, 4H) ppm; ^{13}C NMR (100 MHz, $CDCl_3$) δ 150.2, 149.3, 135.9, 133.7, 123.3, 119.1, 118.7 (q, J = 318.2 Hz), 26.6, 24.4, 21.6, 21.1 ppm; IR (thin film) 3444, 2949, 2874, 1603, 1566, 1533, 1455, 1425, 1356, 1288, 1218, 1176, 1138, 1074, 1020, 976, 955, 875, 833, 810, 791, 764, 680 (cm^{-1}); HRMS (APCI) m/z: calcd for $C_{11}H_9F_3NO_5S$ $[M - H]^-$, 324.0159; found 324.0161.

To a solution of 4-nitro-5,6,7,8-tetrahydronaphthalen-1-yl triflate (6.5 g, 20.00 mmol, 1.0 equiv) in HOAc (15 mL) at room temperature was added iron powder (5.6 g, 100.00 mmol, 5.0 equiv). After stirred for three hours, the residue iron powder was filtered out. Water (20 mL) was added to the mother liquor and it was extracted with DCM (20 mL x 3). The combined organic layers were washed with brine (20 mL), dried over Na_2SO_4, filtered, and concentrated. A crude oil was obtained, which was used directly in the next step without further purification.

To the above crude oil in anhydrous DCM (50 mL) at room temperature was added NIS (5.4 g, 24.0 mmol, 1.2 equiv). After stirred for three hours, all the volatiles were directly removed on rotary evaporator. Flash column chromatography on silica gel afforded 6.15 g (73%) of 4-amino-3-iodo-5,6,7,8-tetrahydronaphthalen-1-yl triflate as a colorless oil. ^{1}H NMR (400 MHz, $CDCl_3$) δ 7.38 (s, 1H), 4.17 (s, 2H), 2.70 (t, J = 6.4 Hz, 2H), 2.46 (t, J = 5.6 Hz, 2H), 1.88-1.82 (m, 2H), 1.79-1.73 (m, 2H) ppm; ^{13}C NMR (100 MHz, $CDCl_3$) δ 144.8, 139.9, 131.6, 128.0, 123.0, 118.8 (q, J = 318.3 Hz), 78.8, 26.0, 24.3, 22.4, 21.4 ppm; IR (thin film) 3488, 3392, 2943, 2856, 1708, 1621, 1415, 1334, 1246, 1216, 1141, 1074, 1035, 970, 920, 883, 831, 808, 770, 744, 669, 642, 600, 567, 502 (cm^{-1}); HRMS (ESI) m/z: calcd for $C_{11}H_{12}F_3INO_3S$ $[M + H]^+$, 421.9529; found 421.9533.

To a solution of 4-amino-3-iodo-5,6,7,8-tetrahydronaphthalen-1-yl triflate (6.0 g, 14.25 mmol, 1.0 equiv) in THF (50 mL) at room temperature was added DMSO (11 mg, 0.14 mmol, 0.01 equiv), and *t*-BuONO (3.41 mL, 28.5 mmol, 2.0 equiv). After stirred for one hour, all the volatiles were directly removed on a rotary evaporator. Flash column chromatography on silica gel afforded 4.34 g (75%) of 3-iodo-5,6,7,8-tetrahydronaphthalen-1-yl triflate as a colorless oil. ^{1}H NMR (400 MHz, $CDCl_3$) δ 7.46 (s, 1H), 7.36 (s, 1H), 2.77 (t, J = 6.0 Hz, 2H), 2.72 (t, J = 5.6 Hz, 2H), 1.83-1.76 (m, 4H) ppm; ^{13}C NMR (100 MHz, $CDCl_3$) δ 148.1, 142.8, 138.2, 130.5, 127.3, 118.7 (q, J = 319.8 Hz), 89.3, 29.2, 23.6, 22.2, 22.0 ppm; IR (thin film) 3431, 2924, 2856, 1632, 1586, 1557, 1491, 1459, 1378, 1346, 1282, 1225, 1188, 1173,

1095, 1020, 970, 912, 860, 818, 770, 742, 677 (cm^{-1}); HRMS (APCI) m/z: calcd for $C_{11}H_9F_3IO_3S$ [M - H]$^-$, 404.9275; found 404.9266.

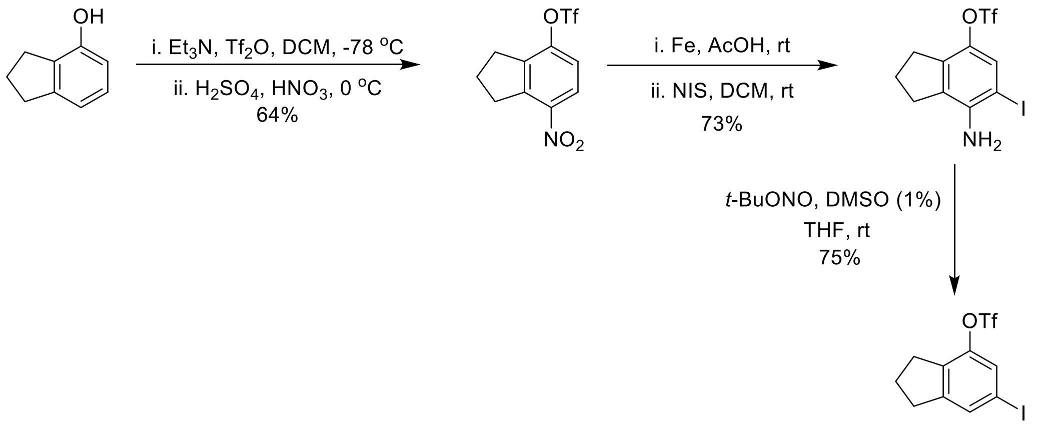

6-Iodo-2,3-dihydro-1*H*-inden-4-yl triflate: To a solution of commercially available 2,3-dihydro-1*H*-inden-4-ol (5.0 g, 37.31 mmol, 1.0 equiv) in anhydrous DCM (50 mL) at -78 ℃ was added Et_3N (7.76 mL, 55.97 mmol, 1.5 equiv). After stirred for 5 minutes, Tf_2O (6.90 mL, 41.04 mmol, 1.1 equiv) was added slowly. After 10 minutes, all the volatiles were directly removed on a rotary evaporator. A crude oil was obtained, which was used directly in the next step without further purification.

To the above crude oil in 98% H_2SO_4 (20 mL) at 0 ℃ was added conc. HNO_3 (68%, 2.28 mL, 50.75 mmol, 1.5 equiv). After stirred for 5 minutes, it was poured into water (20 mL). The resulting mixture was extracted with DCM (20 mL x 3). The combined organic layers were washed with brine (20 mL), dried over Na_2SO_4, filtered, and concentrated. Flash column chromatography on silica gel afforded 7.36 g (64%) of 7-nitro-2,3-dihydro-1*H*-inden-4-yl triflate as a colorless oil. ^{1}H NMR (400 MHz, $CDCl_3$) δ 8.10 (d, *J* = 9.2 Hz, 1H), 7.26 (d, *J* = 8.8 Hz, 1H), 3.50 (t, *J* = 7.6 Hz, 2H), 3.13 (t, *J* = 7.6 Hz, 2H), 2.30-2.22 (m, 2H) ppm; ^{13}C NMR (100 MHz, $CDCl_3$) δ 149.2, 145.4, 144.9, 140.8, 125.0, 120.4, 118.8 (q, *J* = 318.4 Hz), 35.1, 30.5, 24.9 ppm; IR (thin film) 3427, 3102, 2992, 1615, 1574, 1529, 1459, 1426, 1351, 1288, 1239, 1220, 1136, 990, 923, 848, 795, 761, 680, 613, 486, 434 (cm^{-1}); HRMS (APCI) m/z: calcd for $C_{10}H_7F_3NO_5S$ [M - H]$^-$, 310.0003; found 310.0004.

To a solution of 7-nitro-2,3-dihydro-1*H*-inden-4-yl triflate (7.0 g, 22.51 mmol, 1.0 equiv) in HOAc (15 mL) at room temperature was added iron powder (6.30 g, 112.54 mmol, 5.0 equiv). After stirred for three hours, the residue iron powder was filtered out. Waster (20 mL) was added to the mother liquor and it was extracted with DCM (50 mL x 3). The combined organic layers were washed with brine (20 mL), dried over Na_2SO_4, filtered, and concentrated. A crude oil was obtained, which was used directly in the next step without further purification.

To the above crude oil in anhydrous DCM (20 mL) at room temperature was added NIS (6.07 g, 27.01 mmol, 1.2 equiv). After stirred for three hours, all the volatiles were directly removed on a rotary evaporator. Flash column chromatography on silica gel afforded 6.50 g (71%) of 7-amino-6-iodo-2,3-dihydro-1*H*-inden-4-yl triflate as a

colorless oil. ^{1}H NMR (400 MHz, $CDCl_3$) δ 7.33 (s, 1H), 4.11 (s, 2H), 2.99 (t, *J* = 7.6 Hz, 2H), 2.79 (t, *J* = 7.6 Hz, 2H), 2.21-2.14 (m, 2H) ppm; ^{13}C NMR (100 MHz, $CDCl_3$) δ 143.2, 138.1, 137.9, 130.3, 129.1, 118.8 (q, *J* = 318.6 Hz), 79.3, 31.6, 31.0, 24.8 ppm; IR (thin film) 3476, 3385, 2952, 2845, 1616, 1458, 1417, 1246, 1213, 1139, 1001, 957, 847, 732, 709, 654, 605, 573, 541, 498 (cm^{-1}); HRMS (ESI) m/z: calcd for $C_{10}H_{10}F_3INO_3S$ $[M + H]^+$, 407.9373; found 407.9375.

To a solution of 7-amino-6-iodo-2,3-dihydro-1*H*-inden-4-yl triflate (6.0 g, 14.74 mmol, 1.0 equiv) in THF (50 mL) at room temperature was added DMSO (11.7 mg, 0.15 mmol, 0.01 equiv) and *t*-BuONO (3.53 mL, 29.48 mmol, 2.0 equiv). After stirred for one hour, all the volatiles were directly removed on rotary evaporator. Flash column chromatography on silica gel afforded 4.39 g (76%) of 6-iodo-2,3-dihydro-1*H*-inden-4-yl triflate as a colorless oil. ^{1}H NMR (400 MHz, $CDCl_3$) δ 7.57 (s, 1H), 7.36 (s, 1H), 2.98 (t, *J* = 7.6 Hz, 2H), 2.13 (m, 2H) ppm; ^{13}C NMR (100 MHz, $CDCl_3$) δ 150.6, 146.1, 136.9, 133.8, 127.6, 118.8 (q, *J* = 318.3 Hz), 90.7, 33.3, 30.1, 25.2 ppm; IR (thin film) 3432, 2966, 2848, 1607, 1564, 1460, 1424, 1311, 1249, 1215, 1141, 979, 856, 805, 767, 686, 651, 604, 567, 518, 492 (cm^{-1}); HRMS (APCI) m/z: calcd for $C_{10}H_7F_3IO_3S$ $[M - H]^-$, 390.9118; found 390.9120.

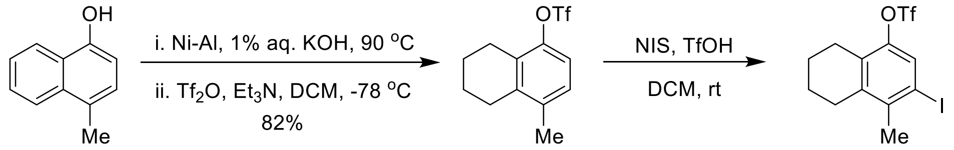

3-Iodo-4-methyl-5,6,7,8-tetrahydronaphthalen-1-yl triflate: To a solution of commercially available 4-methyl-1-naphthol (5.0 g, 31.65 mmol, 1.0 equiv) in 1% aq. KOH (100 mL) at 90 ℃ was added Ni-Al (27.09 g, 316.50 mmol, 10.0 equiv). After stirred for three days, the residue Ni-Al powder was filtered out. Water (100 mL) was added to the mother liquor and it was extracted with EtOAc (50 mL x 3). The combined organic layers were washed with brine (100 mL), dried over Na_2SO_4, filtered, and concentrated. A crude oil was obtained, which was used directly in the next step without further purification.

To the above crude oil in anhydrous DCM (50 mL) at -78 ℃ was added Et_3N (6.58 mL, 47.48 mmol, 1.5 equiv). After stirred for 5 minutes, Tf_2O (5.85 mL, 34.82 mmol, 1.1 equiv) was added slowly. After 10 minutes, all the volatiles were directly removed on a rotary evaporator. Flash column chromatography on silica gel afforded 7.63 g (82%) of 4-methyl-5,6,7,8-tetrahydronaphthalen-1-yl triflate as a colorless oil. ^{1}H NMR (400 MHz, $CDCl_3$) δ 7.02 (d, *J* = 8.4 Hz, 1H), 6.97 (d, *J* = 8.4 Hz, 1H), 2.78 (t, *J* = 6.4 Hz, 2H), 2.63 (t, *J* = 6.0 Hz, 2H), 2.22 (s, 3H), 1.87-1.75 (m, 4H) ppm; ^{13}C NMR (100 MHz, $CDCl_3$) δ 146.9, 138.8, 137.1, 130.2, 128.0, 118.9 (q, *J* = 318.1 Hz), 117.8, 27.3, 24.3, 22.6, 21.9, 19.5 ppm; IR (thin film) 3441, 2943, 1629, 1467, 1419, 1213, 1142, 1016, 975, 935, 872, 816, 695, 622, 585, 495 (cm^{-1}); HRMS (APCI) m/z: calcd for $C_{12}H_{12}F_3O_3S$ $[M - H]^-$, 293.0465; found 293.0463.

To a solution of 4-methyl-5,6,7,8-tetrahydronaphthalen-1-yl triflate (3.5 g, 11.90 mmol, 1.0 equiv) in anhydrous DCM (50 mL) at room temperature was added NIS

(3.21 g, 14.28 mmol, 1.2 equiv). Triflic acid (TfOH) (2.13 mL, 23.80 mmol, 2.0 equiv) was then added slowly. After stirred for three hours, water (50 mL) was added to the mother liquor and it was extracted with DCM (50 mL x 3). The combined organic layers were washed with brine (20 mL), dried over Na_2SO_4, filtered, and concentrated. A crude oil was obtained, which was used directly in the next step without further purification.

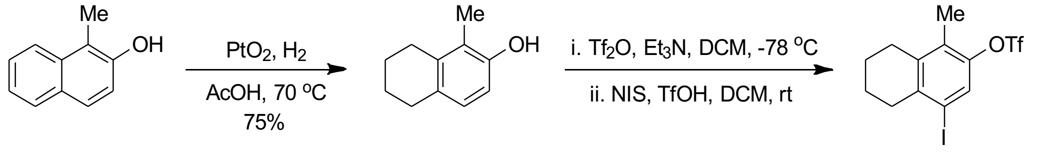

4-Iodo-1-methyl-5,6,7,8-tetrahydronaphthalen-2-yl triflate: To a solution of commercially available 1-methylnaphthalen-2-ol (5.0 g, 31.65 mmol, 1.0 equiv) in AcOH (50 mL) at 70 ℃ was added PtO_2 (7.18 g, 31.65 mmol, 1.0 equiv) under H_2 atmosphere. After stirred for three days, water (100 mL) was added to the mother liquor and it was extracted with EtOAc (50 mL x 3). The combined organic layers were washed with brine (100 mL), dried over Na_2SO_4, filtered, and concentrated. Flash column chromatography on silica gel afforded 3.85 g (75%) of 1-methyl-5,6,7,8-tetrahydronaphthalen-2-ol as a colorless oil, the ^{1}H NMR of which is identical with that reported in literature.[22] ^{1}H NMR (400 MHz, $CDCl_3$) δ 6.84 (d, J = 8.4 Hz, 1H), 6.59 (d, J = 8.4 Hz, 1H), 4.59 (s, 1H), 2.70 (t, J = 6.2 Hz, 2H), 2.64 (t, J = 6.2 Hz, 2H), 2.13 (s, 3H), 1.83-1.74 (m, 4H) ppm.

To a solution of 1-methyl-5,6,7,8-tetrahydronaphthalen-2-ol (3.0 g, 18.52 mmol, 1.0 equiv) in anhydrous DCM (50 mL) at -78 ℃ was added Et_3N (3.85 mL, 27.78 mmol, 1.5 equiv). After stirred for 5 minutes, Tf_2O (3.43 mL, 20.37 mmol, 1.1 equiv) was added slowly. After 10 minutes, waster (50 mL) was added to the mother liquor and it was extracted with DCM (50 mL x 3). The combined organic layers were washed with brine (20 mL), dried over Na_2SO_4, filtered, and concentrated. A crude oil was obtained, which was used directly in the next step without further purification.

To the above crude oil in anhydrous DCM (40 mL) at room temperature was added NIS (5.00 g, 22.22 mmol, 1.2 equiv). Triflic acid (TfOH) (2.96 mL, 37.04 mmol, 2.0 equiv) was then added slowly. After stirred for three hours, all the volatiles were directly removed on a rotary evaporator. The combined organic layers were washed with brine (20 mL), dried over Na_2SO_4, filtered, and concentrated. A crude oil was obtained, which was used directly in the next step without further purification.

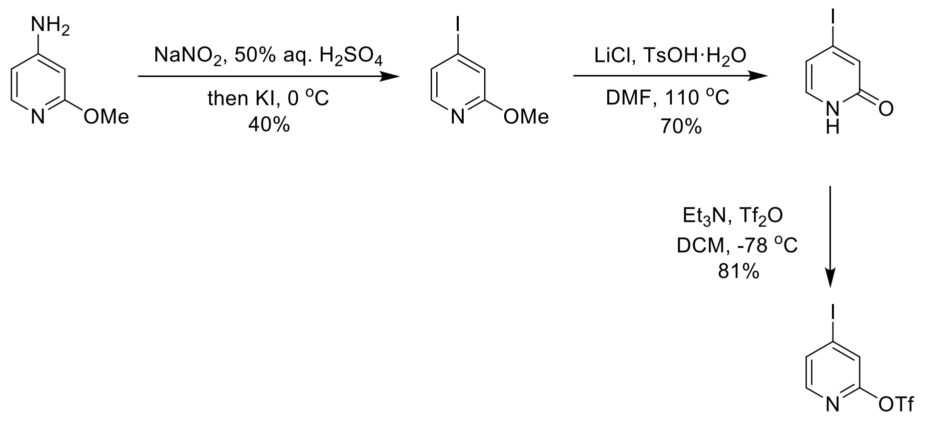

4-Iodopyridin-2-yl triflate: To a solution of commercially available methyl 4-amino-2-methoxypyridine (1.0 g, 8.06 mmol, 1.0 equiv) in 50% aq. H_2SO_4 (20 mL) at 0 ℃was added $NaNO_2$ (612 mg, 8.87 mmol, 1.1 equiv) in portions. After stirred for 20 minutes, the resulting solution was slowly poured into a solution of KI (2.68 g, 16.12 mmol, 2.0 equiv) in water (30 mL) at 0 ℃. It was extracted with EtOAc (20 mL x 3). The combined organic layers were washed with brine (20 mL), dried over Na_2SO_4, filtered, and concentrated. Flash column chromatography with pet ether afforded 0.76 g (40%) of 4-iodo-2-methoxypyridine as a yellow oil, the ^{1}H NMR of which is identical with that reported in literature.[23] ^{1}H NMR (400 MHz, $CDCl_3$) δ 7.83 (d, J = 5.6 Hz, 1H), 7.16-7.10 (m, 2H), 3.90 (s, 3H) ppm.

To a solution of methyl 4-iodo-2-methoxypyridine (700 mg, 2.98 mmol, 1.0 equiv) in DMF (20 mL) at 110 ℃ was added LiCl (379 mg, 8.94 mmol, 3.0 equiv) and TsOH·H_2O (2.83 mg, 14.90 mmol, 5.0 equiv). After stirred for 8 hours, the mother liquor was poured into H_2O (50 mL). It was extracted with EtOAc (20 mL x 3). The combined organic layers were washed with brine (20 mL), dried over Na_2SO_4, filtered, and concentrated. Flash column chromatography with pet ether afforded 461 mg (70%) of 4-iodopyridin-2(1*H*)-one as a yellow oil, the ^{1}H NMR of which is identical with that reported in literature.[24] ^{1}H NMR (400 MHz, DMSO-*d*6) δ 11.8 (s, 1H), 7.15 (d, J = 6.5 Hz, 1H), 6.89 (s, 1H), 6.48 (d, J = 6.5 Hz, 1H) ppm.

To a solution of 4-iodopyridin-2(1*H*)-one (400 mg, 1.81 mmol, 1.0 equiv) in anhydrous DCM (20 mL) at -78 ℃was added Et_3N (0.38 mL, 2.72 mmol, 1.5 equiv). After stirred for 5 minutes, Tf_2O (0.33 mL, 1.99 mmol, 1.1 equiv) was added slowly. After 10 minutes, all the volatiles were directly removed on a rotary evaporator. Flash column chromatography on silica gel afforded 516 mg (81%) of 4-iodopyridin-2-yl triflate as a colorless oil. ^{1}H NMR (400 MHz, $CDCl_3$) δ 8.33 (d, J = 5.2 Hz, 1H), 7.40 (dd, J_1 = 5.6 Hz, J_2 = 2.0 Hz, 1H), 7.22 (d, J = 1.6 Hz, 1H) ppm; ^{13}C NMR (100 MHz, $CDCl_3$) δ 156.4, 149.3, 148.1, 125.0, 118.8 (q, J = 320.3 Hz), 116.0 ppm; IR (thin film) 3450, 2929, 1585, 1558, 1428, 1219, 1138, 1086, 923, 810, 761, 609, 550, 507 (cm^{-1}); HRMS (APCI) m/z: calcd for $C_6H_4F_3INO_3S$ [M + H]$^+$, 353.8903; found 353.8896.

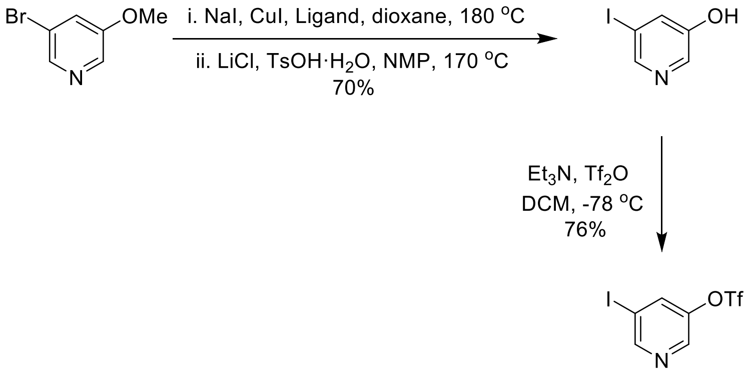

5-Iodopyridin-3-yl triflate: To a solution of commercially available methyl 3-bromo-5-methoxypyridine (1.0 g, 5.35 mmol, 1.0 equiv) in dioxane (20 mL) at 180 ℃ was added NaI (2.41 g, 16.05 mmol, 3.0 equiv) and CuI (510 mg, 2.68 mmol, 0.5 equiv). After stirred for 5 minutes, (1*R*,2*R*)-*N*,*N*'-dimethyl-1,2-cyclohexanediamine (152 mg, 1.07 mmol, 0.2 equiv) was added in portions. After stirred for 16 hours, water (50 mL) was added to the mother liquor and it was extracted with DCM (50 mL x 3). The combined organic layers were washed with brine (20 mL), dried over Na_2SO_4, filtered, and concentrated. A crude oil was obtained, which was used directly in the next step without further purification.

To the above crude oil in anhydrous NMP (20 mL) at 170 ℃ was added LiCl (681 mg, 16.05 mmol, 3.0 equiv) and $TsOH{\cdot}H_2O$ (5.08 g, 26.75 mmol, 5.0 equiv). After stirred for 8 hours, the precipitate was filtered out, the mother liquor was poured into water (400 mL) and it was extracted with EtOAc (50 mL x 3). The combined organic layers were washed with brine (20 mL), dried over Na_2SO_4, filtered, and concentrated. Flash column chromatography on silica gel afforded 828 mg (70%) of 5-iodopyridin-3-ol as a colorless oil, the ^{1}H NMR of which is identical with that reported in literature.[25] ^{1}H NMR (400 MHz, acetone-*d*6) δ 8.33 (d, J = 1.6 Hz, 1H), 8.20 (d, J = 2.7 Hz, 1H), 7.63 (dd, J_1 = 2.7 Hz, J_2 = 1.6 Hz,1H) ppm.

To a solution of 5-iodopyridin-3-ol (800 mg, 3.62 mmol, 1.0 equiv) in anhydrous DCM (20 mL) at -78 ℃ was added Et_3N (0.76 mL, 5.43 mmol, 1.5 equiv). After stirred for 5 minutes, Tf_2O (0.66 mL, 3.98 mmol, 1.1 equiv) was added slowly. After 10 minutes, all the volatiles were directly removed on a rotary evaporator. Flash column chromatography on silica gel afforded 971 mg (76%) of 5-iodopyridin-3-yl triflate as a colorless oil. ^{1}H NMR (400 MHz, $CDCl_3$) δ 8.87 (s, 1H), 8.57 (d, J = 2.4 Hz, 1H), 7.99 (dd, J_1 = 2.4 Hz, J_2 = 2.0 Hz, 1H) ppm; ^{13}C NMR (100 MHz, $CDCl_3$) δ 155.9, 146.2, 141.6, 137.5, 118.8 (q, J = 319.2 Hz), 92.6 ppm; IR (thin film) 3450, 3062, 1563, 1432, 1297, 1251, 1219, 1142, 1007, 899, 799, 758, 692, 631, 599, 518 (cm^{-1}); HRMS (APCI) m/z: calcd for $C_6H_4F_3INO_3S$ $[M + H]^+$, 353.8903; found 353.8893.

2.2. General procedure for the transformation of aryl triflate to aryl tosylate or aryl 2,6-difluorophenylsulfonate (Procedures A)

To a solution of 3-iodoaryl triflate (1.0 mmol, 1.0 equiv) in 1,4-dioxane (20 mL) at room temperature was added 40% aq. Bu_4NOH (1.33 mL, 2.0 mmol, 2.0 equiv). After stirred for three hours, water (20 mL) was added and it was extracted with DCM (20 mL x 3). The combined organic layers were washed with brine (20 mL), dried over Na_2SO_4, filtered, and concentrated. A crude oil was obtained, which was used directly in the next step without further purification.

To a solution of the above crude oil in THF (20 mL) at room temperature was added K_2CO_3 (276 mg, 2.0 mmol, 2.0 equiv) and *p*-TsCl (0.22 mL, 1.5 mmol, 1.5 equiv) or 2,6-difluorophenylsulfonyl chloride (0.20 mL, 1.5 mmol, 1.5 equiv). After stirred for three hours. Water (20 mL) was added and it was extracted with DCM (20 mL x 3). The combined organic layers were washed with brine (20 mL), dried over Na_2SO_4, filtered, and concentrated. Flash column chromatography on silica gel afforded the corresponding 3-iodoaryl tosylate or 3-iodoaryl 2,6-difluorophenylsulfonate.

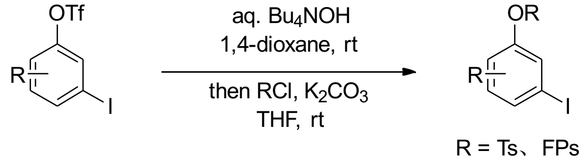

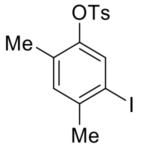

3s-Ts

Compound 3s-Ts: The synthesis of compound **3s-Ts** was followed by Procedure A. 341 mg (85%) of compound **3s-Ts** was isolated as a colorless oil. ^{1}H NMR (400 MHz, $CDCl_3$) δ 7.74 (d, *J* = 8.4 Hz, 2H), 7.35 (d, *J* = 8.4 Hz, 2H), 7.32 (s, 1H), 7.03 (s, 1H), 2.47 (s, 3H), 2.34 (s, 3H), 1.99 (s, 3H) ppm; ^{13}C NMR (100 MHz, $CDCl_3$) δ 146.2, 145.8, 140.4, 132.9, 132.4, 132.2, 131.8, 130.1, 128.7, 96.2, 27.4, 22.0, 16.2 ppm; IR (thin film) 3439, 2914, 1638, 1595, 1569, 1472, 1430, 1373, 1302, 1247, 1192, 1181, 1123, 1088, 1027, 949, 886, 831, 805, 743, 715, 651, 596, 548, 449 (cm^{-1}); HRMS (ESI) m/z: calcd for $C_{15}H_{15}INaO_3S$ $[M + Na]^+$, 424.9679; found 424.9674.

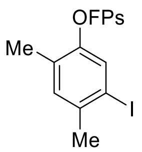

3s-FPs

Compound 3s-FPs: The synthesis of compound **3s-FPs** was followed by Procedure A. 339 mg (80%) of compound **3s-FPs** was isolated as a colorless oil. ^{1}H NMR (400 MHz, $CDCl_3$) δ 7.71-7.63 (m, 1H), 7.44 (s, 1H), 7.14-7.10 (m, 3H), 2.36 (s, 3H), 2.22 (s, 3H) ppm; ^{13}C NMR (100 MHz, $CDCl_3$) δ 160.3 (dd, J_1 = 260.0 Hz, J_2 = 2.9 Hz), 145.8, 141.0, 136.8 (t, *J* = 10.8 Hz), 132.5, 131.9, 131.8, 113.6 (m), 96.3, 27.4, 16.4 ppm; IR (thin film) 3440, 2917, 1614, 1473, 1369, 1285, 1239, 1190, 1094, 1013, 952,

877, 842, 787, 686, 669, 602, 544 (cm^{-1}); HRMS (ESI) m/z: calcd for $C_{14}H_{11}F_2IKO_3S$ $[M + K]^+$, 462.9073; found 462.9078.

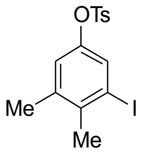

3t-Ts

Compound 3t-Ts: The synthesis of compound **3t-Ts** was followed by Procedure A. 318 mg (79%) of compound **3t-Ts** was isolated as a colorless oil. ^1H NMR (400 MHz, $CDCl_3$) δ 7.72 (d, J = 8.4 Hz, 2H), 7.34 (d, J = 8.0 Hz, 2H), 7.21 (d, J = 2.4 Hz, 1H), 6.84 (d, J = 2.4 Hz, 1H), 2.47 (s, 3H), 2.35 (s, 3H), 2.28 (s, 3H) ppm; ^{13}C NMR (100 MHz, $CDCl_3$) δ 147.1, 145.7, 138.9, 138.5, 132.4, 130.5, 130.0, 128.7, 123.9, 101.1, 24.8, 22.3, 22.0 ppm; IR (thin film) 3438, 2926, 1583, 1466, 1371, 1195, 1171, 1085, 952, 880, 820, 721, 657, 617, 549 (cm^{-1}); HRMS (ESI) m/z: calcd for $C_{15}H_{15}INaO_3S$ $[M + Na]^+$, 424.9679; found 424.9669.

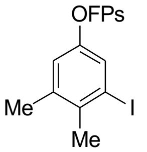

3t-FPs

Compound 3t-FPs: The synthesis of compound **3t-FPs** was followed by Procedure A. 339 mg (80%) of compound **3t-FPs** was isolated as a colorless oil. ^1H NMR (400 MHz, $CDCl_3$) δ 7.68-7.61 (m, 1H), 7.43 (d, J = 2.8 Hz, 1H), 7.09 (t, J = 8.4 Hz, 2H), 7.02 (d, J = 2.0 Hz, 1H), 2.36 (s,3 H), 2.31 (s, 3H) ppm; ^{13}C NMR (100 MHz, $CDCl_3$) δ 160.2 (dd, J_1 = 260.0 Hz, J_2 = 2.9 Hz), 146.5, 139.5, 138.9, 136.8 (t, J = 10.8 Hz), 129.9, 123.4, 113.5 (m), 101.3, 24.8, 22.4 ppm; IR (thin film) 3425, 2929, 1614, 1589, 1471, 1389, 1197, 1174, 1108, 956, 883, 828, 797, 722, 637, 539 (cm^{-1}); HRMS (ESI) m/z: calcd for $C_{14}H_{11}F_2INaO_3S$ $[M + Na]^+$, 446.9334; found 446.9330.

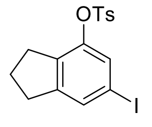

3u-Ts

Compound 3u-Ts: The synthesis of compound **3u-Ts** was followed by Procedure A. 360 mg (87%) of compound **3u-Ts** was isolated as a colorless oil. ^1H NMR (400 MHz, $CDCl_3$) δ 7.73 (d, J = 8.4 Hz, 2H), 7.44 (s, 1H), 7.34 (d, J = 8.0 Hz, 2H), 7.06 (s, 1H), 2.85 (t, J = 7.6 Hz, 2H), 2.59 (t, J = 7.6 Hz, 2H), 2.47 (s, 3H), 1.96-1.89 (m, 2H) ppm; ^{13}C NMR (100 MHz, $CDCl_3$) δ 149.7, 146.3, 145.8, 137.9, 132.9, 132.6, 130.1, 129.0, 128.7, 90.3, 33.2, 30.0, 25.2, 22.0 ppm; IR (thin film) 3430, 2924, 2839, 1598, 1447, 1378, 1115, 989, 865, 822, 761, 660, 541 (cm^{-1}); HRMS (ESI) m/z: calcd for $C_{16}H_{15}INaO_3S$ $[M + Na]^+$, 436.9679; found 436.9679.

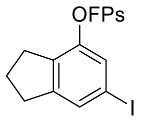

3u-FPs

Compound 3u-FPs: The synthesis of compound **3u-FPs** was followed by Procedure A. 336 mg (77%) of compound **3u-FPs** was isolated as a colorless oil. ^{1}H NMR (400 MHz, $CDCl_3$) δ 7.71-7.64 (m, 1H), 7.46 (s, 1H), 7.20 (s, 1H), 7.10 (t, *J* = 8.4 Hz, 1H), 2.89 (t, *J* = 7.6 Hz, 2H), 2.84 (t, *J* = 7.6 Hz, 2H), 2.05-1.98 (m, 2H) ppm; ^{13}C NMR (100 MHz, $CDCl_3$) δ 160.1 (dd, J_1 = 260.0 Hz, J_2 = 2.9 Hz), 150.1, 145.7, 137.6, 136.9 (t, *J* = 11.0 Hz), 132.9, 128.2, 113.6-113.4 (m), 90.4, 33.2, 30.0, 25.1 ppm; IR (thin film) 3430, 2958, 2362, 1612, 1563, 1402, 1294, 1216, 1140, 982, 859, 796, 761, 602, 538 (cm^{-1}); HRMS (ESI) m/z: calcd for $C_{15}H_{11}F_2INaO_3S$ $[M + Na]^+$, 458.9334; found 458.9333.

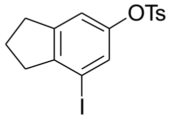

3v-Ts

Compound 3v-Ts: The synthesis of compound **3v-Ts** was followed by Procedure A. 356 mg (86%) of compound **3v-Ts** was isolated as a colorless oil. ^{1}H NMR (400 MHz, $CDCl_3$) δ 7.72 (d, *J* = 8.4 Hz, 2H), 7.34 (d, *J* = 8.0 Hz, 2H), 7.04 (s, 1H), 6.86 (s, 1H), 3.00 (t, *J* = 7.6 Hz, 2H), 2.83 (t, *J* = 7.6 Hz, 2H), 2.46 (s, 3H), 2.11-2.03 (m, 2H) ppm; ^{13}C NMR (100 MHz, $CDCl_3$) δ 148.2, 148.0, 145.9, 145.7, 132.5, 130.0, 129.2, 128.7, 118.6, 92.5, 37.9, 34.9, 24.2, 21.9 ppm; IR (thin film) 3401, 2949, 2839, 1629, 1612, 1485, 1453, 1414, 1375, 1212, 1141, 1087, 1016, 955, 866, 818, 738, 654, 546 (cm^{-1}); HRMS (ESI) m/z: calcd for $C_{16}H_{15}INaO_3S$ $[M + Na]^+$, 436.9679; found 436.9685.

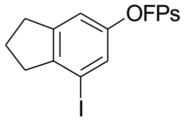

3v-FPs

Compound 3v-FPs: The synthesis of compound **3v-FPs** was followed by Procedure A. 336 mg (77%) of compound **3v-FPs** was isolated as a colorless oil. ^{1}H NMR (400 MHz, $CDCl_3$) δ 7.69-7.62 (m, 1H), 7.26 (s, 1H), 7.09 (t, *J* = 8.4 Hz, 2H), 7.02 (s, 1H), 3.02 (d, *J* = 7.6 Hz, 2H), 2.82 (d, *J* = 7.2 Hz, 2H), 2.11-2.03 (m, 2H) ppm; ^{13}C NMR (100 MHz, $CDCl_3$) δ 160.1 (dd, J_1 = 260.0 Hz, J_2 = 2.9 Hz), 148.6, 147.4, 146.2, 136.9 (t, *J* = 10.8 Hz), 128.5, 118.0, 113.6-113.3 (m), 92.7, 37.9, 34.9, 24.1 ppm; IR (thin film) 3441, 2911, 2845, 1613, 1589, 1566, 1473, 1446, 1374, 1285, 1236, 1192, 1143, 1107, 1085, 1005, 873, 793, 537 (cm^{-1}); HRMS (ESI) m/z: calcd for $C_{15}H_{11}F_2INaO_3S$ $[M + Na]^+$, 458.9334; found 458.9337.

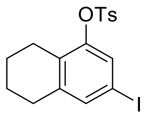

3w-Ts

Compound 3w-Ts: The synthesis of compound **3w-Ts** was followed by Procedure A. 321 mg (75%) of compound **3w-Ts** was isolated as a colorless oil. ^{1}H NMR (400 MHz, $CDCl_3$) δ 7.77 (d, J = 8.0 Hz, 2H), 7.36 (d, J = 8.0 Hz, 2H), 7.32 (s, 1H), 7.06 (s, 1H), 2.68 (t, J = 5.6 Hz, 2H), 2.50 (t, J = 6.0 Hz, 2H), 2.48 (s, 3H), 1.68-1.64 (m ,4H) ppm; ^{13}C NMR (100 MHz, $CDCl_3$) δ 148.1, 145.6, 141.8, 136.7, 133.0, 131.1, 129.9, 128.4, 128.0, 88.8, 29.0, 23.4, 22.2, 22.0, 21.8 ppm; IR (thin film) 3431, 2924, 2856, 1629, 1598, 1551, 1459, 1401, 1346, 1225, 1173, 1095, 1020, 958, 912, 818, 770, 718, 677, 559 (cm^{-1}); HRMS (ESI) m/z: calcd for $C_{17}H_{17}INaO_3S$ [M + Na]$^+$, 450.9835; found 450.9842.

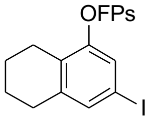

3w-FPs

Compound 3w-FPs: The synthesis of compound **3w-FPs** was followed by Procedure A. 315 mg (70%) of compound **3w-FPs** was isolated as a colorless oil. ^{1}H NMR (400 MHz, $CDCl_3$) δ 7.71-7.64 (m, 1H), 7.36 (s, 1H), 7.18 (s, 1H), 7.12 (t, J = 8.8 Hz, 2H), 2.72-2.70 (m, 4H), 1.73-1.72 (m, 4H) ppm; ^{13}C NMR (100 MHz, $CDCl_3$) δ 160.2 (dd, J_1 = 260.0 Hz, J_2 = 3.0 Hz), 147.9, 142.4, 137.3, 136.8 (t, J = 10.9 Hz), 131.2, 127.6, 113.7-113.5 (m), 89.1, 29.2, 23.6, 22.4, 22.1 ppm; IR (thin film) 3441, 2938, 2859, 1612, 1589, 1473, 1400, 1323, 1294, 1241, 1192, 1108, 1009, 969, 911, 864, 792 (cm^{-1}); HRMS (ESI) m/z: calcd for $C_{16}H_{13}F_2INaO_3S$ [M + Na]$^+$, 472.9490; found 472.9496.

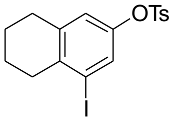

3x-Ts

Compound 3x-Ts: The synthesis of compound **3x-Ts** was followed by Procedure A. 385 mg (90%) of compound **3x-Ts** was isolated as a colorless oil. ^{1}H NMR (400 MHz, $CDCl_3$) δ 7.73 (d, J = 8.4 Hz, 2H), 7.34 (d, J = 8.4 Hz, 2H), 7.20 (d, J = 2.4 Hz, 1H), 6.78 (s, 1H), 2.66 (t, J = 6.4 Hz, 2H), 2.59 (t, J = 6.4 Hz, 2H), 2.47 (s, 3H), 1.82-1.74 (m, 2H), 1.71-1.65 (m, 2H) ppm; ^{13}C NMR (100 MHz, $CDCl_3$) δ 147.0, 145.7, 140.4, 138.7, 132.5, 130.2, 130.0, 128.7, 123.1, 102.1, 35.5, 30.7, 24.0, 22.5, 22.0 ppm; IR (thin film) 3427, 2920, 2851, 1627, 1592, 1401, 1369, 1173, 1112, 996, 932, 863, 802, 773, 724, 660, 602, 550 (cm^{-1}); HRMS (ESI) m/z: calcd for $C_{17}H_{17}INaO_3S$ [M + Na]$^+$, 450.9835; found 450.9828.

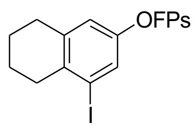

3x-FPs

Compound 3x-FPs: The synthesis of compound **3x-FPs** was followed by Procedure A. 315 mg (70%) of compound **3x-FPs** was isolated as a colorless oil. ^{1}H NMR (400 MHz, $CDCl_3$) δ 7.67-7.61 (m, 1H), 7.43 (d, J = 2.4 Hz, 1H), 7.09 (t, J = 8.4 Hz, 2H), 6.94 (s, 1H), 2.69 (t, J = 6.0 Hz, 2H), 2.59 (t, J = 6.4 Hz, 2H), 1.81-1.76 (m ,2H), 1.71-1.67 (m, 2H) ppm; ^{13}C NMR (100 MHz, $CDCl_3$) δ 160.2 (dd, J_1 = 260.0 Hz, J_2 = 2.9 Hz), 146.4, 140.7, 139.3, 136.8 (t, J = 11.0 Hz), 129.5, 122.5, 113.6-113.3 (m), 102.4, 35.5, 30.7, 23.9, 22.4 ppm; IR (thin film) 3424, 2936, 2851, 2301, 1614, 1590, 1562, 1471, 1449, 1396, 1298, 1240, 1175, 1106, 1009, 993, 935, 801, 636, 541, 447 (cm^{-1}); HRMS (ESI) m/z: calcd for $C_{16}H_{13}F_2INaO_3S$ $[M + Na]^+$, 472.9490; found 472.9492.

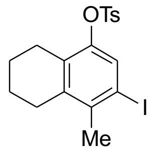

3y-Ts

Compound 3y-Ts: The synthesis of compound **3y-Ts** was followed by Procedure A. 407 mg (92%) of compound **3y-Ts** was isolated as a colorless oil. ^{1}H NMR (400 MHz, $CDCl_3$) δ 7.78 (d, J = 8.4 Hz, 2H), 7.36 (d, J = 8.0 Hz, 2H), 7.19 (s, 1H), 2.63 (t, J = 6.4 Hz, 2H), 2.55 (t, J = 6.4 Hz, 2H), 2.48 (s, 3H), 2.33 (s, 3H), 1.76-1.70 (m, 2H), 1.65-1.59 (m, 2H) ppm; ^{13}C NMR (100 MHz, $CDCl_3$) δ 146.2, 145.7, 138.5, 133.4, 131.7, 130.0, 129.5, 128.7, 97.3, 29.3, 24.6, 24.4, 23.1, 22.0, 21.5 ppm; IR (thin film) 3415, 2940, 1569, 1440, 1373, 1220, 1176, 1093, 1019, 975, 873, 810, 739, 703, 663, 563, 544 (cm^{-1}); HRMS (ESI) m/z: calcd for $C_{18}H_{19}INaO_3S$ $[M + Na]^+$, 464.9992; found 464.9995.

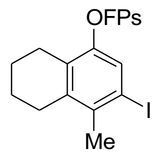

3y-FPs

Compound 3y-FPs: The synthesis of compound **3y-FPs** was followed by Procedure A. 353 mg (76%) of compound **3y-FPs** was isolated as a colorless oil. ^{1}H NMR (400 MHz, $CDCl_3$) δ 7.69-7.65 (m, 1H), 7.33 (s, 1H), 7.12 (t, J = 8.4 Hz, 2H), 2.73 (t, J = 6.0 Hz, 2H), 2.66 (t, J = 4.8 Hz, 2H), 2.34 (s ,3H), 1.79-1.76 (m, 2H), 1.71-1.69 (m, 2H) ppm; ^{13}C NMR (100 MHz, $CDCl_3$) δ 160.2 (dd, J_1 = 260.0 Hz, J_2 = 3.0 Hz), 145.8, 139.0, 138.8, 136.7 (t, J = 10.7 Hz), 131.5, 128.8, 113.7-113.4 (m), 97.4, 29.3, 24.7, 24.4, 23.0, 21.5 ppm; IR (thin film) 3445, 2935, 1612, 1473, 1390, 1367, 1294, 1236, 1185, 1107, 1011, 973, 812, 737, 704, 674 (cm^{-1}); HRMS (ESI) m/z: calcd for $C_{17}H_{15}F_2INaO_3S$ $[M + Na]^+$, 486.9647; found 486.9655.

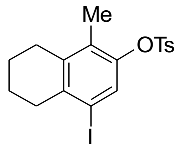

3z-Ts

Compound 3z-Ts: The synthesis of compound **3z-Ts** was followed by Procedure A. 407 mg (92%) of compound **3z-Ts** was isolated as a colorless oil. ^{1}H NMR (400 MHz, $CDCl_3$) δ 7.74 (dt, J_1 = 8.4 Hz, J_2 = 2.0 Hz, 2H), 7.35 (d, J = 8.0 Hz, 2H), 7.22 (s, 1H), 2.60 (t, J = 3.6 Hz, 2H), 2.53 (t, J = 6.0 Hz, 2H), 2.47 (s, 3H), 1.93 (s, 3H), 1.74-1.71 (m, 4H) ppm; ^{13}C NMR (100 MHz, $CDCl_3$) δ 145.9, 145.6, 139.5, 138.3, 133.1, 130.6, 130.0, 128.6, 98.6, 36.4, 28.2, 23.4, 22.7, 21.9, 12.7 ppm; IR (thin film) 3441, 2928, 1570, 1436, 1356, 1196, 1174, 1054, 920, 863, 809, 683, 659, 554 (cm^{-1}); HRMS (ESI) m/z: calcd for $C_{18}H_{19}INaO_3S$ $[M + Na]^+$, 464.9992; found 464.9993.

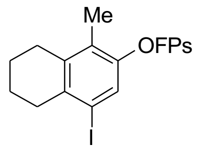

3z-FPs

Compound 3z-FPs: The synthesis of compound **3z-FPs** was followed by Procedure A. 418 mg (90%) of compound **3z-FPs** was isolated as a colorless oil. ^{1}H NMR (400 MHz, $CDCl_3$) δ 7.71-7.64 (m, 1H), 7.34 (s, 1H), 7.12 (t, J = 8.0 Hz, 2H), 2.62-2.60 (m, 4H), 2.13 (s, 3H), 1.77-1.73 (m, 4H) ppm; ^{13}C NMR (100 MHz, $CDCl_3$) δ 160.2 (dd, J_1 = 260.0 Hz, J_2 = 3.1 Hz), 145.5, 139.9, 138.9, 136.7 (t, J = 11.0 Hz), 130.6, 129.3, 113.7-113.4 (m), 98.8, 36.5, 28.3, 23.5, 22.7, 12.9 ppm; IR (thin film) 3444, 2926, 1611, 1474, 1433, 1373, 1305, 1199, 1173, 1047, 1010, 916, 802, 703, 669, 538 (cm^{-1}); HRMS (ESI) m/z: calcd for $C_{17}H_{15}F_2INaO_3S$ $[M + Na]^+$, 486.9647; found 486.9646.

2.3. General procedure for the preparation of aryne precursors 1 (Procedures B and C)

Oxidation with *m*-CPBA (Procedure B): To a solution of aryl iodide (1.0 mmol, 1.0 equiv) and TfOH (0.16 mL, 2.0 mmol, 2.0 equiv) in DCM (30 mL) at room temperature was added *m*-CPBA (190 mg, 1.1 mmol, 1.1 equiv). After stirred for 30 minutes, 1,3,5-trimethylbenzene (0.15 mL, 1.1 mmol, 1.1 equiv) was added. After three hours, all the volatiles were directly removed on a rotary evaporator. The resulting crude oil was triturated with diethyl ether and isolated by filtration to afford pure aryne precursor **1**.

Oxidation with Oxone (Procedure C): To a solution of aryl iodide (1.0 mmol, 1.0 equiv) and TfOH (0.16 mL, 2.0 mmol, 2.0 equiv) in MeCN (30 mL) at 0 ℃was added Oxone (923 mg, 1.5 mmol, 1.5 equiv). After stirred for five minutes, 1,3,5-trimethylbenzene (0.15 mL, 1.1 mmol, 1.1 equiv) was added. After three hours, all the volatiles were directly removed on a rotary evaporator. The resulting crude oil

was triturated with diethyl ether and isolated by filtration to afford pure aryne precursor **1**.

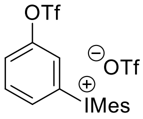

1b

Compound 1b: The synthesis of compound **1b** was followed by Procedure B. 533 mg (86%) of compound **1b** was isolated as a white solid. Mp: 127-128 °C; ^{1}H NMR (400 MHz, $CDCl_3$) δ 7.80 (d, J = 8.0 Hz, 1H), 7.52-7.48 (m, 2H), 7.41 (dd, J_1 = 8.4 Hz, J_2 = 1.6 Hz, 1H), 7.12 (s, 2H), 2.60 (s, 6H), 2.35 (s, 3H) ppm; ^{13}C NMR (100 MHz, $CDCl_3$) δ 150.4, 145.0, 142.7, 133.4, 133.1, 130.6, 125.7, 124.8, 121.1, 120.2 (q, J = 317.8 Hz), 118.7 (q, J = 319.2 Hz), 111.7, 27.1, 21.3 ppm; IR (thin film) 3450, 3102, 3071, 2987, 2914, 1580, 1465, 1427, 1378, 1220, 1168, 1138, 1024, 987, 892, 793, 750, 674, 635, 606, 572, 518, 428 (cm^{-1}); HRMS (ESI) m/z: calcd for $C_{16}H_{15}F_3IO_3S$ $[M - OTf]^+$ 470.9733; found 470.9733.

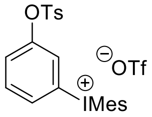

1c

Compound 1c: The synthesis of compound **1c** was followed by Procedure B. 448 mg (73%) of compound **1c** was isolated as a white solid. Mp: 149-150 °C; ^{1}H NMR (400 MHz, $CDCl_3$) δ 7.70 (d, J = 8.0 Hz, 1H), 7.55 (d, J = 8.0 Hz, 2H), 7.31 (t, J = 8.0 Hz, 1H), 7.29 (d, J = 8.0 Hz, 2H), 7.23 (s, 1H), 7.06 (m, 3H), 2.51 (s, 6H), 2.40 (s, 3H), 2.30 (s, 3H) ppm; ^{13}C NMR (100 MHz, $CDCl_3$) δ 150.8, 146.4, 144.7, 142.4, 132.9, 132.0, 131.1, 130.4, 130.2, 128.4, 126.6, 125.8, 120.1 (q, J = 318.2 Hz), 120.8, 110.8, 26.9, 21.8, 21.1 ppm; IR (thin film) 3441, 3091, 2966, 2917, 1734, 1638, 1580, 1563, 1465, 1418, 1379, 1274, 1199, 1174, 1085, 990, 944, 877, 813, 744, 683, 660, 638, 557, 509, 446 (cm^{-1}); HRMS (ESI) m/z: calcd for $C_{22}H_{22}IO_3S$ $[M - OTf]^+$493.0329; found 493.0334.

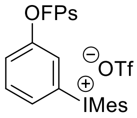

1d

Compound 1d: The synthesis of compound **1d** was followed by Procedure B. 517 mg (78%) of compound **1d** was isolated as a white solid. Mp: 136-137 °C; ^{1}H NMR (400 MHz, $CDCl_3$) δ 7.76 (d, J = 8.0 Hz, 1H), 7.70-7.63 (m, 1H), 7.42-7.37 (m, 2H), 7.32 (d, J = 8.4 Hz, 1H), 7.11 (s, 2H), 7.06 (t, J = 8.4 Hz, 2H), 2.56 (s, 6H), 2.36 (s, 3H) ppm; ^{13}C NMR (100 MHz, $CDCl_3$) δ 159.9 (dd, J_1 = 260.0 Hz, J_2 = 2.5 Hz), 150.4, 144.9, 142.6, 113.8-113.6 (m), 133.2, 132.4, 130.6, 126.0, 125.3, 120.8, 120.2 (q, J = 318.4 Hz), 113.7 (d, J = 25.7 Hz), 111.2, 27.1, 21.3 ppm; IR (thin film) 3450, 3094,

2920, 1612, 1585, 1473, 1407, 1276, 1246, 1203, 1170, 1108, 1029, 1009, 879, 801, 748, 680, 637, 567, 541, 516, 481, 446 (cm^{-1}); HRMS (ESI) m/z: calcd for $C_{21}H_{18}F_2IO_3S$ [M - OTf]$^+$, 514.9984; found 515.0009.

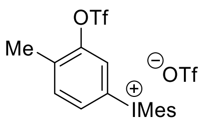

1e

Compound 1e: The synthesis of compound **1e** was followed by Procedure B. 507 mg (80%) of compound **1e** was isolated as a white solid. Mp: 190-191 °C; ^{1}H NMR (400 MHz, DMSO-*d6*) δ 8.08 (s, 1H), 7.89 (d, *J* = 8.4 Hz, 1H), 7.59 (d, *J* = 8.4 Hz, 1H), 7.24 (s, 2H), 2.60 (s, 6H), 2.35 (s, 3H), 2.30 (s, 3H) ppm; ^{13}C NMR (100 MHz, DMSO-*d6*) δ 148.0, 143.4, 141.7, 135.3, 135.0, 134.3, 129.9, 127.2, 123.0, 120.7 (q, *J* = 320.3 Hz), 118.0 (q, *J* = 318.4 Hz), 111.0, 26.2, 20.5, 15.7 ppm; IR (thin film) 3481, 3377, 2958, 2842, 1615, 1458, 1417, 1246, 1213, 1139, 999, 957, 847, 741, 648, 605, 567, 498 (cm^{-1}); HRMS (ESI) m/z: calcd for $C_{17}H_{17}F_3IO_3S$ [M - OTf]$^+$, 484.9890; found 484.9888.

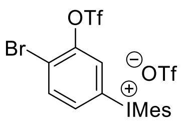

1f

Compound 1f: The synthesis of compound **1f** was followed by Procedure B. 557 mg (80%) of compound **1f** was isolated as a white solid. Mp: 175-176 °C; ^{1}H NMR (400 MHz, DMSO-*d6*) δ 8.23 (d, *J* = 1.6 Hz, 1H), 8.01 (d, *J* = 8.8 Hz, 1H), 7.85 (dd, J_1 = 8.8 Hz, J_2 = 1.6 Hz, 1H), 7.26 (s, 2H), 2.60 (s, 6H), 2.30 (s, 3H) ppm; ^{13}C NMR (100 MHz, DMSO-*d6*) δ 146.8, 143.8, 141.9, 137.2, 135.5, 130.0, 128.7, 123.0, 120.7 (q, *J* = 320.4 Hz), 119.8, 118.1 (q, *J* = 321.0 Hz), 113.1, 26.3, 20.5 ppm; IR (thin film) 3441, 3094, 1589, 1557, 1461, 1420, 1383, 1278, 1238, 1169, 1130, 1026, 889, 864, 819, 784, 753, 663, 634, 591, 515, 495, 437 (cm^{-1}); HRMS (ESI) m/z: calcd for $C_{16}H_{14}BrF_3IO_3S$ [M - OTf]$^+$, 548.8838; found 548.8833.

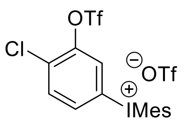

1g

Compound 1g: The synthesis of compound **1g** was followed by Procedure B. 562 mg (86%) of compound **1g** was isolated as a white solid. Mp: 174-175 °C; ^{1}H NMR (400 MHz, DMSO-*d6*) δ 8.33 (d, *J* = 1.6 Hz, 1H), 7.96 (dd, J_1 = 8.8 Hz, J_2 = 1.6 Hz, 1H), 7.88 (d, *J* = 8.4 Hz, 1H), 7.25 (s, 2H), 2.60 (s, 6H), 2.30 (s, 3H) ppm; ^{13}C NMR (100 MHz, DMSO-*d6*) δ 145.3, 143.8, 141.9, 135.7, 134.2, 130.0, 129.2, 123.1, 120.8 (q, *J* = 320.1 Hz), 119.7, 118.1 (q, *J* = 319.1 Hz), 112.5, 26.3, 20.6 ppm; IR (thin film) 3453, 3097, 1589, 1563, 1469, 1420, 1386, 1279, 1238, 1169, 1130, 1048, 1026, 891,

810, 787, 755, 680, 635, 594, 512, 434 (cm^{-1}); HRMS (ESI) m/z: calcd for $C_{16}H_{14}ClF_3IO_3S$ [M - OTf]$^+$, 504.9344; found 504.9324.

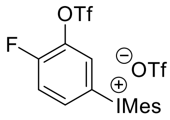

1h

Compound 1h: The synthesis of compound **1h** was followed by Procedure B. 567 mg (89%) of compound **1h** was isolated as a white solid. Mp: 148-149 °C; ^{1}H NMR (400 MHz, DMSO-*d6*) δ 8.56 (d, *J* = 8.0 Hz, 1H), 8.12-8.10 (m, 1H), 7.74 (t, *J* = 8.0 Hz, 1H), 7.24 (s, 2H), 2.62 (s, 6H), 2.29 (s, 3H) ppm; ^{13}C NMR (100 MHz, DMSO-*d6*) δ 154.9 (d, *J* = 255.9 Hz), 143.6, 141.8, 137.3 c, 136.7 (d, *J* = 14.0 Hz), 130.8, 129.9, 123.3, 121.2 (d, *J* = 19.4 Hz), 120.7 (q, *J* = 320.4 Hz), 118.1 (q, *J* = 319.0 Hz), 108.5 (d, *J* = 3.8 Hz), 26.3, 20.5 ppm; IR (thin film) 3444, 3105, 3062, 2917, 1592, 1491, 1436, 1428, 1278, 1248, 1221, 1168, 1134, 1114, 1024, 899, 815, 744, 700, 635, 608, 570, 509 (cm^{-1}); HRMS (ESI) m/z: calcd for $C_{16}H_{14}F_4IO_3S$ [M - OTf]$^+$, 488.9639; found 488.9640.

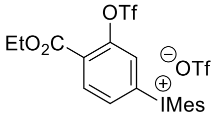

1i

Compound 1i: The synthesis of compound **1i** was followed by Procedure B. 678 mg (98%) of compound **1i** was isolated as a white solid. Mp: 175-176 °C; ^{1}H NMR (400 MHz, DMSO-*d6*) δ 8.15 (s, 1H), 8.10 (d, *J* = 8.4 Hz, 1H), 8.07 (d, *J* = 8.4 Hz, 1H), 7.27 (s, 2H), 4.34 (q, *J* = 8.0 Hz, 2H), 2.59 (s, 6H), 2.31 (s, 3H), 1.29 (t, *J* = 8.0 Hz, 3H) ppm; ^{13}C NMR (100 MHz, DMSO-*d6*) δ 162.4, 147.2, 143.9, 142.0, 134.8, 134.6, 130.1, 128.3, 126.9, 123.0, 120.8 (q, *J* = 319.9 Hz), 118.8, 118.0 (q, *J* = 319.0 Hz), 62.6, 26.3 (d, *J* = 3.0 Hz), 20.6 (d, *J* = 4.0 Hz), 13.8 ppm; IR (thin film) 3450, 3088, 2992, 1732, 1589, 1436, 1386, 1279, 1234, 1170, 1139, 1085, 1027, 905, 848, 781, 637, 602, 512 (cm^{-1}); HRMS (ESI) m/z: calcd for $C_{19}H_{19}F_3IO_5S$ [M - OTf]$^+$, 542.9945; found 542.9946.

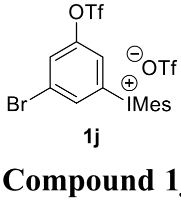

1j

Compound 1j: The synthesis of compound **1j** was followed by Procedure B. 656 mg (94%) of compound **1j** was isolated as a white solid. Mp: 102-103 °C; ^{1}H NMR (400 MHz, DMSO-*d6*) δ 8.33 (s, 1H), 8.20 (s, 1H), 8.16 (s, 1H), 7.25 (s, 2H), 2.60 (s, 6H), 2.30 (s, 3H) ppm; ^{13}C NMR (100 MHz, DMSO-*d6*) δ 149.2, 143.7, 141.9, 136.8, 130.0, 128.5, 126.6, 124.2, 123.1, 120.7 (q, *J* = 320.3 Hz), 118.1 (q, *J* = 319.3 Hz), 115.4, 26.3, 20.5 ppm; IR (thin film) 3470, 3068, 1569, 1426, 1289, 1217, 1167, 1135,

1024, 921, 857, 796, 753, 721, 660, 635, 601, 564, 517 (cm^{-1}); HRMS (ESI) m/z: calcd for $C_{16}H_{14}BrF_3IO_3S$ [M - OTf]$^+$, 548.8838; found 548.8840.

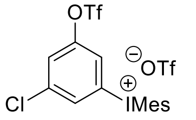

1k

Compound 1k: The synthesis of compound **1k** was followed by Procedure B. 569 mg (87%) of compound **1k** was isolated as a white solid. Mp: 103-104 °C; ^{1}H NMR (400 MHz, $CDCl_3$) δ 7.77 (s, 1H), 7.39 (d, *J* = 1.2 Hz, 2H), 7.13 (s, 2H), 2.62 (s, 6H), 2.36 (s, 3H) ppm; ^{13}C NMR (100 MHz, $CDCl_3$) δ 150.2, 145.0, 142.5, 138.4, 132.5, 130.7, 125.1, 123.7, 122.4, 120.2 (q, *J* = 317.5 Hz), 118.7 (q, *J* = 319.2 Hz), 114.4, 27.2, 21.4 ppm; IR (thin film) 3444, 2917, 2845, 1634, 1575, 1430, 1383,1222, 1168, 1137, 1027, 925, 857, 802, 760, 637, 564, 518 (cm^{-1}); HRMS (ESI) m/z: calcd for $C_{16}H_{14}ClF_3IO_3S$ [M - OTf]$^+$ 504.9344; found 504.9337.

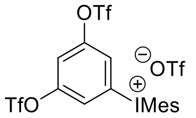

1l

Compound 1l: The synthesis of compound **1l** was followed by Procedure B. 392 mg (51%) of compound **1l** was isolated as a white solid. Mp: 131-132 °C; ^{1}H NMR (400 MHz, DMSO-*d*6) δ 8.38 (d, *J* = 2.0 Hz, 2H), 8.35 (m, 1H), 7.26 (s, 2H), 2.62 (s, 6H), 2.30 (s, 3H) ppm; ^{13}C NMR (100 MHz, DMSO-*d*6) δ 149.3, 143.8, 142.0, 130.0, 128.5, 123.4, 120.7 (q, *J* = 320.4 Hz), 120.4, 119.8, 118.2 (q, *J* = 319.5 Hz), 116.6 114.9, 26.3 (d, *J* = 2.5 Hz), 20.5 (d, *J* = 3.6 Hz) ppm; IR (thin film) 3444, 3082, 1588, 1430, 1283, 1218, 1166, 1136, 1111, 1028, 977, 875, 797, 761, 637, 606, 573, 515 (cm^{-1}); HRMS (ESI) m/z: calcd for $C_{17}H_{14}F_6IO_6S_2$ [M - OTf]$^+$, 618.9175; found 618.9163.

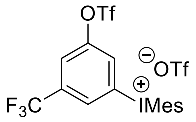

1m

Compound 1m: The synthesis of compound **1m** was followed by Procedure B. 365 mg (53%) of compound **1m** was isolated as a viscous oil. ^{1}H NMR (400 MHz, DMSO-*d*6) δ 8.55 (s, 1H), 8.37 (s, 2H), 7.26 (s, 2H), 2.62 (s, 6H), 2.30 (s, 3H) ppm; ^{13}C NMR (100 MHz, DMSO-*d*6) δ 149.4, 143.9, 142.0, 133.0 (q, *J* = 34.0 Hz), 131.5 (m), 131.3, 130.0, 123.0, 122.9, 121.9 (q, *J* = 272.2 Hz), 120.9 (q, *J* = 320.2 Hz), 118.1 (q, *J* = 319.3 Hz), 115.4, 26.3, 20.5 ppm; IR (thin film) 3424, 3062, 2269, 2130, 1653, 1577, 1431, 1312, 1249, 1222, 1181, 1140, 1091, 1028, 1003, 931, 880, 823, 765, 685, 603, 567, 521 (cm^{-1}); HRMS (ESI) m/z: calcd for $C_{17}H_{14}F_6IO_3S$ [M - OTf]$^+$, 538.9607; found 538.9605.

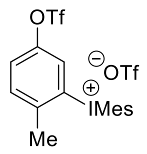

1n

Compound 1n: The synthesis of compound **1n** was followed by Procedure B. 520 mg (82%) of compound **1n** was isolated as a white solid. Mp: 169-170 °C; ^{1}H NMR (400 MHz, DMSO-*d6*) δ 8.29 (s, 1H), 7.74 (s, 2H), 7.22 (s, 2H), 2.58 (s, 6H), 2.55 (s, 3H), 2.28 (s, 3H) ppm; ^{13}C NMR (100 MHz, DMSO-*d6*) δ 147.1, 143.3, 142.1, 141.9, 133.2, 130.1, 129.9, 125.5, 121.8, 120.8 (q, J = 319.9 Hz), 118.2 (q, J = 318.2 Hz), 118.0, 26.0, 23.8, 20.4 ppm; IR (thin film) 3432, 3102, 1586, 1482, 1421, 1378, 1275, 1109, 1166, 1134, 1026, 903, 874, 724, 635, 588, 509, 440 (cm^{-1}); HRMS (ESI) m/z: calcd for $C_{17}H_{17}F_3IO_3S$ [M - OTf]$^+$, 484.9890; found 484.9886.

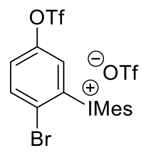

1o

Compound 1o: The synthesis of compound **1o** was followed by Procedure B. 621 mg (89%) of compound **1o** was isolated as a white solid. Mp: 170-171 °C; ^{1}H NMR (400 MHz, DMSO-*d6*) δ 8.57 (d, J = 2.8 Hz, 1H), 8.13 (d, J = 8.8 Hz, 1H), 7.78 (dd, J_1 = 8.8 Hz, J_2 = 2.4 Hz, 1H), 7.22 (s, 2H), 2.62 (s, 6H), 2.28 (s, 3H) ppm; ^{13}C NMR (100 MHz, DMSO-*d6*) δ 147.9, 143.5, 142.3, 135.6, 132.5, 130.2, 127.5, 127.4, 122.8, 120.8 (q, J = 320.0 Hz), 120.2, 118.2 (q, J = 319.3 Hz), 26.2, 20.4 ppm; IR (thin film) 3447, 3088, 1566, 1453, 1420, 1276, 1237, 1166, 1132, 1025, 886, 784, 758, 634, 608, 573, 515 (cm^{-1}); HRMS (ESI) m/z: calcd for $C_{16}H_{14}BrF_3IO_3S$ [M - OTf]$^+$, 548.8838; found 548.8845.

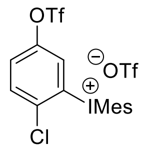

1p

Compound 1p: The synthesis of compound **1p** was followed by Procedure B. 595 mg (91%) of compound **1p** was isolated as a white solid. Mp: 184-185 °C; ^{1}H NMR (400 MHz, DMSO-*d6*) δ 8.58 (d, J = 2.8 Hz, 1H), 8.13 (d, J = 8.8 Hz, 1H), 7.79 (dd, J_1 = 8.8 Hz, J_2 = 2.8 Hz, 1H), 7.22 (s, 2H), 2.62 (s, 6H), 2.28 (s, 3H) ppm; ^{13}C NMR (100 MHz, DMSO-*d6*) δ 147.4, 143.5, 142.2, 136.8, 132.4, 132.3, 130.1, 127.5, 122.6, 120.7 (q, J = 320.4 Hz), 118.2 (q, J = 319.5 Hz), 117.1, 26.1, 20.5 ppm; IR (thin film) 3453, 3088, 1572, 1458, 1421, 1378, 1277, 1237, 1169, 1132, 1025, 879, 794, 761, 636, 615, 582, 512 (cm^{-1}); HRMS (ESI) m/z: calcd for $C_{16}H_{14}ClF_3IO_3S$ [M - OTf]$^+$, 504.9344; found 504.9347.

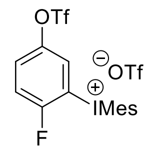

1q

Compound 1q: The synthesis of compound **1q** was followed by Procedure B. 510 mg (80%) of compound **1q** was isolated as a white solid. Mp: 194-195 °C; ^{1}H NMR (400 MHz, DMSO-*d6*) δ 8.69 (t, J = 4.0 Hz, 1H), 7.93 (dt, J_1 = 8.4 Hz, J_2 = 4.0 Hz, 1H), 7.76 (t, J = 8.4 Hz, 1H), 7.21 (s, 2H), 2.63 (s, 6H), 2.27 (s, 3H) ppm; ^{13}C NMR (100 MHz, DMSO-*d6*) δ 159.3 (d, J = 249.4 Hz), 145.2 (d, J = 2.4 Hz), 143.6, 141.9, 130.9, 130.0, 128.7 (d, J = 8.9 Hz), 123.0, 120.8 (q, J = 320.5 Hz), 118.8 (d, J = 25.8 Hz), 118.1 (q, J = 319.0 Hz), 102.4 (d, J = 27.2 Hz), 26.1, 20.5 (d, J = 3.8 Hz) ppm; IR (thin film) 3453, 3100, 1589, 1480, 1425, 1275, 1234, 1172, 1134, 1026, 881, 834, 689, 635, 582, 512 (cm^{-1}); HRMS (ESI) m/z: calcd for $C_{16}H_{14}F_4IO_3S$ [M - OTf]$^+$, 488.9639; found 488.9639.

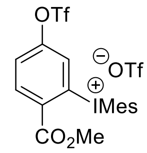

1r

Compound 1r: The synthesis of compound **1r** was followed by Procedure B. 509 mg (75%) of compound **1r** was isolated as a viscous oil. ^{1}H NMR (400 MHz, DMSO-*d6*) δ 8.46 (d, J = 8.4 Hz, 1H), 8.01 (dd, J_1 = 8.4 Hz, J_2 = 2.4 Hz, 1H), 7.44 (s, 2H), 6.62 (d, J = 2.4 Hz, 1H), 4.08 (s, 3H), 2.52 (s, 6H), 2.43 (s, 3H) ppm; ^{13}C NMR (100 MHz, DMSO-*d6*) δ 166.9, 153.3, 145.0, 143.3, 134.6, 130.2, 128.5, 124.8, 121.6, 120.7 (q, J = 320.2 Hz), 118.8, 117.9 (q, J = 319.3 Hz), 115.9, 54.8, 26.0, 20.7 ppm; IR (thin film) 3427, 2252, 2118, 1655, 1421, 1386, 1308, 1271, 1213, 1135, 1027, 1003, 891, 826, 764, 637 (cm^{-1}); HRMS (ESI) m/z: calcd for $C_{18}H_{17}F_3IO_5S$ [M - OTf]$^+$, 528.9788; found 528.9795.

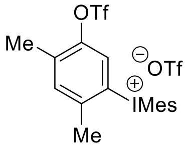

1s

Compound 1s: The synthesis of compound **1s** was followed by Procedure B. 473 mg (73%) of compound **1s** was isolated as a white solid. Mp: 162-163 °C; ^{1}H NMR (400 MHz, DMSO-*d6*) δ 8.06 (s, 1H), 7.68 (s, 1H), 7.23 (s, 2H), 2.58 (s, 6H), 2.51 (s, 3H), 2.34 (s, 3H), 2.30 (s, 3H) ppm; ^{13}C NMR (100 MHz, DMSO-*d6*) δ 145.9, 143.2, 141.7, 141.6, 135.1, 134.7, 130.0, 129.2, 121.9, 120.7 (q, J = 320.2 Hz), 118.0 (q, J = 318.7 Hz), 114.6, 25.9, 23.6, 20.4, 15.7 ppm; IR (thin film) 3444, 2917, 1629, 1470, 1417, 1375, 1275, 1241, 1167, 1135, 1094, 1025, 949, 868, 738, 680, 636, 591, 573,

507, 443 (cm^{-1}); HRMS (ESI) m/z: calcd for $C_{18}H_{19}F_3IO_3S$ [M - OTf]$^+$, 499.0046; found 499.0046.

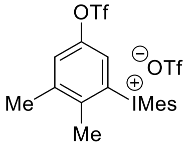

1t

Compound 1t: The synthesis of compound **1t** was followed by Procedure B. 473 mg (73%) of compound **1t** was isolated as a white solid. Mp: 134-135 °C; ^{1}H NMR (400 MHz, DMSO-*d6*) δ 8.08 (d, *J* = 2.4 Hz, 1H), 7.69 (d, *J* = 2.0 Hz, 1H), 7.23 (s, 2H), 2.58 (s, 6H), 2.51 (s, 3H), 2.41 (s, 3H), 2.29 (s, 3H) ppm; ^{13}C NMR (100 MHz, DMSO-*d6*) δ 146.9, 143.2, 142.1, 141.8, 140.5, 130.0, 128.2, 127.2, 126.2, 122.4, 120.7 (q, *J* = 320.3 Hz), 119.2, 118.1 (q, *J* = 319.2 Hz), 26.1, 22.3, 21.3, 20.4 ppm; IR (thin film) 3423, 2254, 2121, 1650, 1424, 1279, 1204, 1168, 1137, 1028, 825, 764, 634 (cm^{-1}); HRMS (ESI) m/z: calcd for $C_{18}H_{19}F_3IO_3S$ [M - OTf]$^+$, 499.0046; found 499.0045.

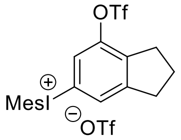

1u

Compound 1u: The synthesis of compound **1u** was followed by Procedure C. 475 mg (72%) of compound **1u** was isolated as a white solid. Mp: 152-153 °C; ^{1}H NMR (400 MHz, DMSO-*d6*) δ 8.01 (s, 1H), 7.94 (s, 1H), 7.23 (s, 2H), 2.98 (t, *J* = 7.6 Hz, 4H), 2.62 (s, 6H), 2.30 (s, 3H), 2.10-2.03 (m ,2H) ppm; ^{13}C NMR (100 MHz, DMSO-*d6*) δ 152.2, 145.6, 143.4, 141.7, 140.7, 130.6, 129.9, 125.3, 122.9, 120.0 (q, *J* = 320.0 Hz), 118.1 (q, *J* = 318.5 Hz), 111.6, 33.0, 29.7, 26.3, 24.7, 20.5 ppm; IR (thin film) 3427, 2972, 1606, 1574, 1432, 1401, 1280, 1231, 1167, 1138, 1028, 985, 853, 796, 758, 712, 637, 605, 570, 544, 512 (cm^{-1}); HRMS (ESI) m/z: calcd for $C_{19}H_{19}F_3IO_3S$ [M - OTf]$^+$ 511.0046; found 511.0046.

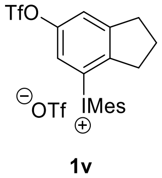

1v

Compound 1v: The synthesis of compound **1v** was followed by Procedure C. 548 mg (83%) of compound **1v** was isolated as a white solid. Mp: 142-143 °C; ^{1}H NMR (400 MHz, DMSO-*d6*) δ 8.11 (d, *J* = 2.0 Hz, 1H), 7.70 (d, *J* = 1.6 Hz, 1H), 7.22 (s, 2H), 3.07 (t, *J* = 7.6 Hz, 2H), 2.94 (t, *J* = 7.6 Hz, 2H), 2.58 (s, 6H), 2.29 (s, 3H), 2.13-2.05 (m, 2H) ppm; ^{13}C NMR (100 MHz, DMSO-*d6*) δ 149.5, 148.6, 147.6, 143.2, 141.9, 130.0, 126.7, 121.6, 121.5, 120.1 (q, *J* = 320.1 Hz), 118.2 (q, *J* = 319.3 Hz), 111.2,

34.9, 34.0, 26.2, 24.4, 20.5 ppm; IR (thin film) 3420, 2963, 2926, 2854, 1725, 1632, 1586, 1560, 1426, 1381, 1222, 1138, 1084, 1032, 984, 953, 915, 877, 851, 764, 735, 680, 639, 604, 579, 541, 515 (cm^{-1}); HRMS (ESI) m/z: calcd for $C_{19}H_{19}F_3IO_3S$ [M - OTf]$^+$ 511.0046; found 511.0046.

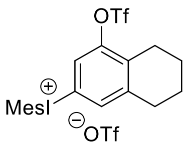

1w

Compound 1w: The synthesis of compound **1w** was followed by Procedure C. 492 mg (73%) of compound **1w** was isolated as a white solid. Mp: 150-151 °C; ^{1}H NMR (400 MHz, DMSO-*d6*) δ 7.77 (s, 1H), 7.75 (s, 1H), 7.22 (s, 2H), 2.76 (t, *J* = 5.6 Hz, 2H), 2.68 (t, *J* = 4.8 Hz, 1H), 2.58 (s, 6H), 2.28 (s, 3H), 1.70 (m, 4H) ppm; ^{13}C NMR (100 MHz, DMSO-*d6*) δ 147.7, 144.5, 143.4, 141.7, 134.7, 134.2, 129.9, 123.9, 122.8, 120.7 (q, *J* = 320.4 Hz), 118.0 (q, *J* = 320.9 Hz), 110.2, 28.6, 26.3, 23.2, 21.2, 20.8, 20.5 ppm; IR (thin film) 3426, 2946, 2854, 1728, 1577, 1464, 1430, 1279, 1219, 1167, 1137, 1026, 961, 911, 865, 825, 793, 755, 636, 602, 588, 518 (cm^{-1}); HRMS (ESI) m/z: calcd for $C_{20}H_{21}F_3IO_3S$ [M - OTf]$^+$, 525.0203; found 525.0201.

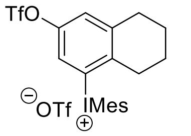

1x

Compound 1x: The synthesis of compound **1x** was followed by Procedure C. 479 mg (71%) of compound **1x** was isolated as a white solid. Mp: 151-152 °C; ^{1}H NMR (400 MHz, DMSO-*d6*) δ 8.08 (d, *J* = 2.4 Hz, 1H), 7.60 (d, *J* = 2.4 Hz, 1H), 7.22 (s, 2H), 2.80 (m, 4H), 2.57 (s, 6H), 2.29 (s, 3H), 1.80-1.77 (m, 2H), 1.67-1.64 (m, 2H) ppm; ^{13}C NMR (100 MHz, DMSO-*d6*) δ 146.8, 143.3, 143.2, 142.0, 139.9, 130.1, 127.2, 125.8, 121.6, 120.7 (q, *J* = 320.0 Hz), 119.3, 118.1 (q, *J* = 320.0 Hz), 31.6, 29.6, 26.1, 22.3, 21.2, 20.5 ppm; IR (thin film) 3426, 2946, 2871, 1593, 1557, 1450, 1425, 1281, 1220, 1137, 1022, 993, 936, 860, 829, 767, 712, 672, 636, 601, 576, 544, 516 (cm^{-1}); HRMS (ESI) m/z: calcd for $C_{20}H_{21}F_3IO_3S$ [M - OTf]$^+$ 525.0203; found 525.0202.

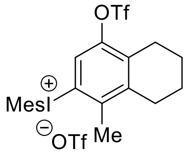

1y

Compound 1y: The synthesis of compound **1y** was followed by Procedure B. 488 mg (71%) of compound **1x** was isolated as a white solid. Mp: 152-153 °C; ^{1}H NMR (400 MHz, DMSO-*d6*) δ 7.80 (s, 1H), 7.22 (s, 2H), 2.71 (t, *J* = 6.0 Hz, 4H), 2.57 (s, 6H), 2.48 (s, 3H), 2.29 (s, 3H), 1.76-1.69 (m, 4H) ppm; ^{13}C NMR (100 MHz, DMSO-*d6*) δ

145.9, 143.2, 141.6, 141.4, 140.0, 134.4, 130.0, 125.6, 122.7, 120.7 (q, *J* = 320.3 Hz), 118.0 (q, *J* = 318.6 Hz), 116.1, 28.1, 26.1, 23.9, 22.3, 21.7, 20.4, 20.3 ppm; IR (thin film) 3438, 2949, 2871, 1574, 1433, 1277, 1244, 1166, 1138, 1027, 981, 865, 816, 729, 638, 608, 515 (cm^{-1}); HRMS (ESI) m/z: calcd for $C_{21}H_{23}F_3IO_3S$ [M - OTf]$^+$, 539.0359; found 539.0359.

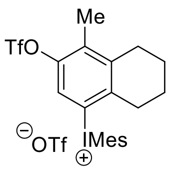

1z

Compound 1z: The synthesis of compound **1z** was followed by Procedure B. 599 mg (87%) of compound **1z** was isolated as a white solid. Mp: 133-134 °C; ^1H NMR (400 MHz, DMSO-d_6) δ 7.88 (s, 1H), 7.22 (s, 2H), 2.82-2.81 (m, 2H), 2.68 (m, 2H), 2.57 (s, 6H), 2.29 (s, 3H), 2.22 (s, 3H), 1.73-1.72 (m, 4H) ppm; ^{13}C NMR (100 MHz, DMSO-d_6) δ 145.8, 143.3, 142.2, 141.8, 139.3, 134.2, 130.1, 126.4, 121.9, 120.7 (q, *J* = 320.4 Hz), 118.0 (q, *J* = 318.6 Hz), 115.9, 32.5, 27.4, 26.0, 21.9, 21.5, 20.4, 12.7 ppm; IR (thin film) 3458, 3076, 2926, 1580, 1430, 1276, 1252, 1167, 1139, 1028, 923, 862, 826, 698, 635, 600, 564, 518 (cm^{-1}); HRMS (ESI) m/z: calcd for $C_{21}H_{23}F_3IO_3S$ [M - OTf]$^+$ 539.0359; found 539.0364.

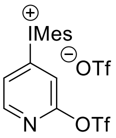

1aa

Compound 1aa: The synthesis of compound **1aa** was followed by Procedure B. 528 mg (85%) of compound **1aa** was isolated as a white solid. Mp: 151-152 °C; ^1H NMR (400 MHz, $CDCl_3$) δ 8.34 (d, *J* = 5.2 Hz, 1H), 7.73 (d, *J* = 5.2 Hz, 1H), 7.32 (s, 1H), 7.17 (s, 2H), 2.61 (s, 6H), 2.39 (s, 3H) ppm; ^{13}C NMR (100 MHz, $CDCl_3$) δ 156.8, 150.6, 145.5, 142.6, 130.9, 128.1, 126.9, 122.1, 118.7 (q, *J* = 318.9 Hz), 118.1, 27.3, 21.4 ppm; IR (thin film) 3449, 2943, 2561, 1698, 1629, 1574, 1421, 1257, 1179, 1035, 920, 753, 709, 643, 518 (cm^{-1}); HRMS (ESI) m/z: calcd for $C_{15}H_{14}F_3IO_3S$ [M - OTf]$^+$ 471.9686; found 471.9683.

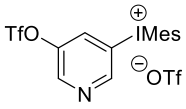

1bb

Compound 1bb: The synthesis of compound **1bb** was followed by Procedure B. 398 mg (64%) of compound **1bb** was isolated as a brown oil. ^1H NMR (400 MHz, $CDCl_3$) δ 8.68 (d, *J* = 2.4 Hz, 1H), 8.62 (d, *J* = 1.6 Hz, 1H), 8.24 (t, *J* = 2.0 Hz, 1H), 7.13 (s, 2H), 2.65 (s, 6H), 2.37 (s, 3H) ppm; ^{13}C NMR (100 MHz, $CDCl_3$) δ 150.8, 146.8, 145.2, 145.1, 142.5, 134.1, 130.8, 121.8, 118.8 (q, *J* = 319.5 Hz), 111.2, 27.3, 21.3

ppm; IR (thin film) 3444, 2923, 1632, 1561, 1429, 1384, 1222, 1140, 1029, 893, 784, 755, 692, 638, 599, 515 (cm^{-1}); HRMS (ESI) m/z: calcd for $C_{15}H_{14}F_3IO_3S$ $[M - OTf]^+$ 471.9686; found 471.9684.

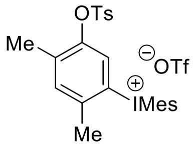

1s-Ts

Compound 1s-Ts: The synthesis of compound **1s-Ts** was followed by Procedure B. 369 mg (55%) of compound **1s-Ts** was isolated as a white solid. Mp: 157-158 °C; ^1H NMR (400 MHz, DMSO-d_6) δ 7.58 (s, 1H), 7.52 (d, J = 8.0 Hz, 2H), 7.43-7.41 (m, 3H), 7.22 (s, 2H), 2.49 (s, 6H), 2.46 (s, 3H), 2.41 (s, 3H), 2.27 (s, 3H), 1.96 (s, 3H) ppm; ^{13}C NMR (100 MHz, DMSO-d_6) δ 146.6, 146.2, 143.3, 141.6, 139.9, 135.8, 134.2, 131.3, 130.5, 130.1, 129.1, 128.1, 122.6, 120.8 (q, J = 320.1 Hz), 114.4, 26.1, 23.6, 21.3, 20.5, 15.7 ppm; IR (thin film) 3434, 2249, 2118, 1652, 1459, 1378, 1277, 1216, 1166, 1029, 1003, 819, 761, 641 (cm^{-1}); HRMS (ESI) m/z: calcd for $C_{24}H_{26}IO_3S$ $[M - OTf]^+$ 521.0642; found 521.0636.

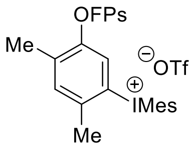

1s-FPs

Compound 1s-FPs: The synthesis of compound **1s-FPs** was followed by Procedure B. 430 mg (62%) of compound **1s-FPs** was isolated as a white solid. Mp: 162-163 °C; ^1H NMR (400 MHz, DMSO-d_6) δ 8.04-7.96 (m, 1H), 7.63 (s, 1H), 7.58 (s, 1H), 7.44 (t, J = 8.8 Hz, 2H), 7.22 (s, 2H), 2.51 (s, 3H), 2.49 (s, 6H), 2.33 (s, 3H), 2.17 (s, 3H) ppm; ^{13}C NMR (100 MHz, DMSO-d_6) δ 158.9 (d, J = 250.0 Hz), 145.8, 143.2, 141.6, 140.6, 139.1 (d, J = 11.7 Hz), 135.9, 134.5, 130.1, 129.0, 121.8, 114.3 (d, J = 9.9 Hz), 114.1, 26.0, 23.6, 20.5, 15.7 ppm; IR (thin film) 3425, 2246, 2130, 1650, 1473, 1386, 1271, 1198, 1169, 1027, 1003, 828, 767, 628, 553 (cm^{-1}); HRMS (ESI) m/z: calcd for $C_{23}H_{22}F_2IO_3S$ $[M - OTf]^+$, 543.0297; found 543.0298.

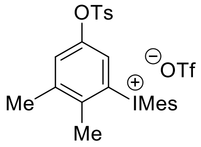

1t-Ts

Compound 1t-Ts: The synthesis of compound **1t-Ts** was followed by Procedure B. 449 mg (67%) of compound **1t-Ts** was isolated as a white solid. Mp: 104-105 °C; ^1H NMR (400 MHz, DMSO-d_6) δ 7.62 (d, J = 8.0 Hz, 2H), 7.48 (s, 1H), 7.43 (d, J = 8.0 Hz, 2H), 7.22 (s, 3H), 2.47 (s, 6H), 2.44 (s, 6H), 2.31 (s, 3H), 2.28 (s, 3H) ppm; ^{13}C NMR (100 MHz, DMSO-d_6) δ 147.3, 146.3, 143.2, 141.6, 141.3, 138.6, 130.8, 130.4,

130.0, 126.9, 126.8, 122.4, 118.8, 26.1, 22.2, 21.3, 20.5 ppm; IR (thin film) 3431, 2253, 2127, 1655, 1456, 1372, 1279, 1169, 1028, 825, 764, 634 (cm^{-1}); HRMS (ESI) m/z: calcd for $C_{24}H_{26}IO_3S$ $[M - OTf]^+$ 521.0642; found 521.0635.

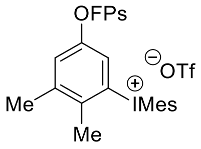

1t-FPs

Compound 1t-FPs: The synthesis of compound **1t-FPs** was followed by Procedure B. 616 mg (89%) of compound **1t-FPs** was isolated as a white solid. Mp: 127-128 °C; ^{1}H NMR (400 MHz, DMSO-d_6) δ 7.97-7.90 (m, 1H), 7.54 (d, J = 2.0 Hz, 1H), 7.39-7.32 (m, 3H), 7.21 (s, 2H), 2.47 (s, 9H), 2.32 (s, 6H) ppm; ^{13}C NMR (100 MHz, DMSO-d_6) δ 158.8 (dd, J_1 = 260.0 Hz, J_2 = 2.7 Hz), 146.7, 143.2, 141.7, 141.5, 139.4, 139.0 (t, J = 11.4 Hz), 130.1, 126.5 (d, J = 16.3 Hz), 122.2, 120.7 (q, J = 320.4 Hz), 119.0, 114.0 (d, J = 25.0 Hz), 111.5 (t, J = 15.3 Hz), 26.0, 22.1, 21.2, 20.5 ppm; IR (thin film) 3426, 2266, 2124, 1654, 1459, 1386, 1256, 1198, 1169, 1027, 826, 765, 636 (cm^{-1}); HRMS (ESI) m/z: calcd for $C_{23}H_{22}F_2IO_3S$ $[M - OTf]^+$ 543.0297; found 543.0301.

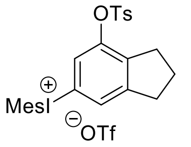

1u-Ts

Compound 1u-Ts: The synthesis of compound **1u-Ts** was followed by Procedure C. 409 mg (60%) of compound **1u-Ts** was isolated as a white solid. Mp: 152-153 °C; ^{1}H NMR (400 MHz, $CDCl_3$) δ 7.63 (d, J = 8.0 Hz, 3H), 7.33 (d, J = 8.0 Hz, 2H), 7.11 (s, 2H), 7.04 (s, 1H), 2.90 (t, J = 7.6 Hz, 2H), 2.62 (t, J = 7.6 Hz, 2H), 2.60 (s, 6H), 2.48 (s, 3H), 2.37 (s, 3H), 1.99-1.91 (m, 2H) ppm; ^{13}C NMR (100 MHz, $CDCl_3$) δ 151.9, 147.4, 146.3, 144.8, 142.9, 142.6, 132.2, 130.7, 130.3, 128.5, 128.4, 120.9, 107.9, 33.6, 30.1, 27.3, 25.1, 22.0, 21.4 ppm; IR (thin film) 3442, 2943, 2856, 1635, 1438, 1407, 1379, 1279, 1173, 1088, 999, 854, 822, 767, 637, 561 (cm^{-1}); HRMS (ESI) m/z: calcd for $C_{25}H_{26}IO_3S$ $[M - OTf]^+$, 533.0642; found 533.0638.

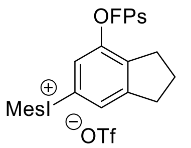

1u-FPs

Compound 1u-FPs: The synthesis of compound **1u-FPs** was followed by Procedure C. 612 mg (87%) of compound **1u-FPs** was isolated as a white solid. Mp: 157-158 °C; ^{1}H NMR (400 MHz, DMSO-*d*6) δ 8.01-7.97 (m, 1H), 7.86 (s, 1H), 7.51 (s, 1H), 7.44 (t, J = 8.8 Hz, 2H), 7.23 (s, 2H), 2.92 (t, J = 7.6 Hz, 2H), 2.77 (t, J = 7.2 Hz, 2H), 2.54

(s, 6H), 2.32 (s, 3H), 2.0-1.93 (m, 2H) ppm; ^{13}C NMR (100 MHz, DMSO-d_6) δ 158.9 (dd, J_1 = 260.0 Hz, J_2 = 2.4 Hz), 151.6, 145.5, 143.3, 141.6, 141.4, 139.1 (t, J = 11.6 Hz), 129.9, 129.8, 125.2, 122.7, 114.2 (d, J = 24.9 Hz), 111.2, 32.9, 29.7, 26.3, 24.7, 20.6 ppm; IR (thin film) 3430, 2981, 1614, 1586, 1563, 1473, 1396, 1280, 1240, 1195, 1141, 1026, 987, 855, 828, 793, 767, 692, 636, 536 (cm^{-1}); HRMS (ESI) m/z: calcd for $C_{24}H_{22}F_2IO_3S$ [M - OTf]$^+$, 555.0297; found 555.0296.

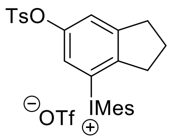

1v-Ts

Compound 1v-Ts: The synthesis of compound **1v-Ts** was followed by Procedure C. 464 mg (68%) of compound **1v-Ts** was isolated as a white solid. Mp: 152-153 °C; ^{1}H NMR (400 MHz, DMSO-*d6*) δ 7.69 (d, J = 8.0 Hz, 2H), 7.48-7.45 (m, 3H), 7.26 (s, 1H), 7.21 (s, 2H), 2.96 (t, J = 6.8 Hz, 2H), 2.91 (t, J = 7.2 Hz, 2H), 2.47 (s, 6H), 2.45 (s, 3H), 2.30 (s, 3H), 2.05-2.02 (m, 2H) ppm; ^{13}C NMR (100 MHz, DMSO-d_6) δ 148.9, 148.0, 146.9, 146.3, 143.2, 141.8, 130.9, 130.4, 130.0, 128.3, 126.5, 122.2, 121.5, 110.8, 34.9, 34.0, 26.2, 24.4, 21.3, 20.5 ppm; IR (thin film) 3430, 2949, 1644, 1606, 1566, 1467, 1384, 1278, 1243, 1187, 1152, 1025, 981, 952, 860, 813, 724, 674, 634, 573, 543, 434 (cm^{-1}); HRMS (ESI) m/z: calcd for $C_{25}H_{26}IO_3S$ [M - OTf]$^+$, 533.0642; found 533.0644.

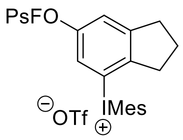

1v-FPs

Compound 1v-FPs: The synthesis of compound **1v-FPs** was followed by Procedure C. 437mg (62%) of compound **1v-FPs** was isolated as a white solid. Mp: 129-130 °C; ^{1}H NMR (400 MHz, DMSO-*d6*) δ 7.96-7.91 (m, 1H), 7.57 (d, J = 1.6 Hz, 1H), 7.42-7.37 (m, 3H), 7.22 (s, 2H), 3.01 (t, J = 7.6 Hz, 2H), 2.95 (t, J = 7.6 Hz, 2H), 2.50 (s, 6H), 2.33 (s, 3H), 2.11-2.03 (m, 2H) ppm; ^{13}C NMR (100 MHz, DMSO-d_6) δ 158.9 (dd, J_1 = 260.0 Hz, J_2 = 2.7 Hz), 149.2, 147.5 (d, J = 9.3 Hz), 143.2, 141.7, 138.9 (t, J = 11.2 Hz), 130.0, 126.0, 121.9, 121.3, 120.7 (q, J = 320.1 Hz), 114.1 (d, J = 25.0 Hz), 111.6 (t, J = 15.3 Hz), 110.9, 34.9, 33.9, 26.1, 24.3, 20.5 ppm; IR (thin film) 3430, 3085, 2972, 1613, 1589, 1557, 1473, 1456, 1397, 1282, 1248, 1187, 1143, 1106, 1031, 1003, 964, 880, 854, 829, 787, 721, 680, 635, 538 (cm^{-1}); HRMS (ESI) m/z: calcd for $C_{24}H_{22}F_2IO_3S$ [M - OTf]$^+$, 555.0297; found 555.0296.

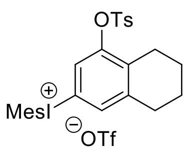

1w-Ts

Compound 1w-Ts: The synthesis of compound **1w-Ts** was followed by Procedure C. 452 mg (65%) of compound **1w-Ts** was isolated as a white solid. Mp: 150-153 °C; ^{1}H NMR (400 MHz,CDCl$_3$) δ 7.64 (d, *J* = 8.0 Hz, 2H), 7.55 (s, 1H), 7.33 (d, *J* = 8.0 Hz, 2H), 7.10 (s, 2H), 7.04 (d, *J* = 9.2 Hz, 1H), 2.72 (t, *J* = 5.6 Hz, 2H), 2.60 (s, 6H), 2.47 (s, 3H), 2.45 (t, *J* = 6.0 Hz, 2H), 2.36 (s, 3H), 1.65-1.57 (m, 4H) ppm; ^{13}C NMR (100 MHz, CDCl$_3$) δ 149.4, 146.3, 144.7, 144.4, 142.6, 136.1, 132.8, 132.4, 131.0, 130.6, 130.3, 128.4, 123.1, 120.9, 106.9, 29.6, 27.3, 23.8, 22.0, 21.6, 21.4 ppm; IR (thin film) 3403, 3100, 2932, 2859, 1644, 1595, 1554, 1467, 1401, 1379, 1328, 1282, 1246, 1167, 1094, 1025, 969, 910, 868, 816, 754, 668, 633, 547, 517 (cm^{-1}); HRMS (ESI) m/z: calcd for $C_{26}H_{28}IO_3S$ [M - OTf]$^+$, 547.0798; found 547.0799.

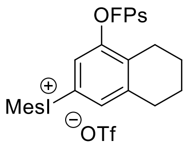

1w-FPs

Compound 1w-FPs: The synthesis of compound **1w-FPs** was followed by Procedure C. 617 mg (86%) of compound **1y-FPs** was isolated as a white solid. Mp: 140-141 °C; ^{1}H NMR (400 MHz, DMSO-d_6) δ 8.04-7.97 (m, 1H), 7.76 (d, *J* = 1.2 Hz, 1H), 7.45 (t, *J* = 8.8 Hz, 2H), 7.34 (d, *J* = 1.6 Hz, 1H), 7.23 (s, 2H), 2.76 (m, 2H), 2.62 (m, 2H), 2.52 (s, 6H), 2.33 (s, 3H), 1.65 (m, 4H) ppm; ^{13}C NMR (100 MHz, DMSO-d_6) δ 158.8 (dd, J_1 = 260.0 Hz, J_2 = 2.6 Hz), 147.8, 144.0, 143.4, 141.6, 139.1 (t, *J* = 11.4 Hz), 134.9, 133.9, 129.9, 123.5, 122.4, 120.7 (q, *J* = 320.0 Hz), 114.3 (d, *J* = 25.2 Hz), 112.7 (t, *J* = 15.3 Hz), 109.7, 28.7, 26.3, 23.3, 21.4, 21.0, 20.6 ppm; IR (thin film) 3427, 2952, 2851, 1611, 1588, 1560, 1474, 1447, 1401, 1323, 1275, 1236, 1193, 1164, 1107, 1024, 1011, 969, 912, 860, 822, 798, 780, 753, 677, 636, 579, 539, 516, 472 (cm^{-1}); HRMS (ESI) m/z: calcd for $C_{25}H_{24}F_2IO_3S$ [M - OTf]$^+$, 569.0453; found 569.0454.

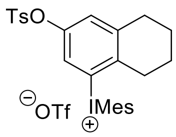

1x-Ts

Compound 1x-Ts: The synthesis of compound **1x-Ts** was followed by Procedure C. 452 mg (65%) of compound **1x-Ts** was isolated as a white solid. Mp: 168-169 °C; ^{1}H NMR (400 MHz, CDCl$_3$) δ 7.60 (d, *J* = 8.0 Hz, 2H), 7.33 (d, *J* = 8.0 Hz, 2H), 7.15 (s, 2H), 6.91 (s, 1H), 6.59 (s, 1H), 2.79 (t, *J* = 5.6 Hz, 2H), 2.74 (t, *J* = 6.0 Hz, 2H), 2.51 (s, 6H), 2.49 (s, 3H), 2.42 (s, 3H), 1.92 (m, 2H), 1.78 (m, 2H) ppm; ^{13}C NMR (100 MHz, CDCl$_3$) δ 149.0, 146.3, 145.5, 143.7, 143.0, 136.8, 131.2, 130.2, 128.6, 126.7, 122.7, 120.7, 115.7, 32.2, 30.2, 27.2, 23.1, 22.0, 21.9, 21.4 ppm; IR (thin film) 3435, 3053, 2930, 2862, 1594, 1551, 1451, 1382, 1278, 1244, 1188, 1155, 1114, 1024, 994, 933, 857, 818, 782, 761, 709, 674, 657, 632, 551, 517 (cm^{-1}); HRMS (ESI) m/z: calcd for $C_{26}H_{28}IO_3S$ [M - OTf]$^+$, 547.0798; found 547.0802.

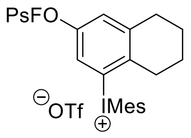

1x-FPs

Compound 1x-FPs: The synthesis of compound **1x-FPs** was followed by Procedure C. 603 mg (84%) of compound **1x-FPs** was isolated as a white solid. Mp: 146-147 °C; ^{1}H NMR (400 MHz, $CDCl_3$) δ 7.72-7.65 (m, 1H), 7.16 (s, 2H), 7.08 (s, 1H), 7.06 (t, *J* = 8.0 Hz, 2H), 6.84 (d, *J* = 2.4 Hz, 1H), 2.80 (t, *J* = 6.4 Hz, 2H), 2.76 (t, *J* = 6.4 Hz, 2H), 2.54 (s, 6H), 2.40 (s, 3H), 1.94-1.88 (m, 2H), 1.80-1.74 (m, 2H) ppm; ^{13}C NMR (100 MHz, $CDCl_3$) δ 160.0 (dd, J_1 = 270.0 Hz, J_2 = 2.7 Hz), 148.2, 145.4, 143.9, 143.0, 137.6, 137.6 (t, *J* = 10.9 Hz), 131.1, 122.5, 120.4 (q, *J* = 318.0 Hz), 119.3, 116.1, 113.7 (d, *J* = 25.8 Hz), 32.2, 30.2, 27.1, 22.9, 21.8, 21.3 ppm; IR (thin film) 3426, 3094, 2951, 1613, 1592, 1560, 1475, 1454, 1397, 1280, 1244, 1201, 1157, 1108, 1026, 1005, 941, 880, 861, 808, 709, 660, 634, 539, 509 (cm^{-1}); HRMS (ESI) m/z: calcd for $C_{25}H_{24}F_2IO_3S$ [M - OTf]$^+$, 569.0453; found 569.0453.

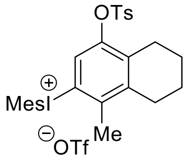

1y-Ts

Compound 1y-Ts: The synthesis of compound **1y-Ts** was followed by Procedure B. 462 mg (65%) of compound **1y-Ts** was isolated as a white solid. Mp: 143-144 °C; ^{1}H NMR (400 MHz, DMSO-*d6*) δ 7.58 (d, *J* = 8.0 Hz, 2H), 7.45 (d, *J* = 8.0 Hz, 2H), 7.42 (s, 1H), 7.23 (s, 2H), 2.63 (t, *J* = 6.0 Hz, 2H), 2.52 (s, 6H), 2.46-2.43 (m, 8H), 2.32 (s, 3H), 1.64-1.63 (m, 2H), 1.53-1.51 (m, 2H) ppm; ^{13}C NMR (100 MHz, $CDCl_3$) δ 146.4, 146.1, 143.1, 141.4, 140.5, 138.1, 134.9, 131.5, 130.3, 130.0, 127.9, 125.3, 123.0, 115.8, 28.1, 26.1, 23.8, 22.2, 21.9, 21.2, 20.5, 20.4 ppm; IR (thin film) 3455, 2937, 1589, 1447, 1372, 1278, 1250, 1191, 1169, 1091, 1029, 975, 851, 786, 727, 635, 567, 538, 504 (cm^{-1}); HRMS (ESI) m/z: calcd for $C_{27}H_{30}IO_3S$ [M - OTf]$^+$, 561.0955; found 561.0958.

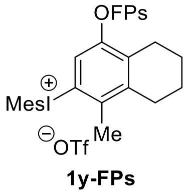

1y-FPs

Compound 1y-FPs: The synthesis of compound **1y-FPs** was followed by Procedure B. 410 mg (56%) of compound **1y-FPs** was isolated as a white solid. Mp: 166-167 °C; ^{1}H NMR (400 MHz, DMSO-*d6*) δ 8.04-7.97 (m, 1H), 7.45 (t, *J* = 8.8 Hz, 2H), 7.39 (s, 1H), 7.21 (s, 2H), 2.69 (t, *J* = 5.6 Hz, 2H), 2.63 (t, *J* = 5.6 Hz, 2H), 2.48 (s, 9H), 2.33 (s, 3H), 1.73-1.7 (m, 2H), 1.64-1.61 (m, 2H) ppm; ^{13}C NMR (100 MHz, DMSO-*d6*) δ

158.8 (dd, J_1 = 260.0 Hz, J_2 = 2.8 Hz), 145.9, 143.2, 141.4, 141.0, 139.0 (t, J = 11.4 Hz), 135.1, 130.0, 125.1, 122.5, 120.8 (q, J = 319.8 Hz), 115.7, 114.3-114.1 (m), 112.7 (t, J = 15.6 Hz), 28.1, 26.1, 23.9, 22.3, 21.9, 20.5, 20.4 ppm; IR (thin film) 3435, 3085, 2932, 2871, 1612, 1580, 1473, 1447, 1398, 1270, 1193, 1161, 1027, 1004, 978, 862, 804, 770, 638, 662, 541, 518 (cm^{-1}); HRMS (ESI) m/z: calcd for $C_{26}H_{26}F_2IO_3S$ $[M - OTf]^+$, 583.0610; found 583.0607.

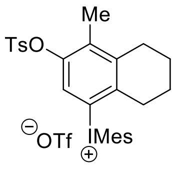

1z-Ts

Compound 1z-Ts: The synthesis of compound **1z-Ts** was followed by Procedure B. 376 mg (53%) of compound **1z-Ts** was isolated as a white solid. Mp: 153-154 °C; ^{1}H NMR (400 MHz, DMSO-*d*6) δ 7.63 (d, J = 8.4 Hz, 2H), 7.48 (d, J = 8.4 Hz, 2H), 7.43 (s, 1H), 7.24 (s, 2H), 2.82 (t, J = 5.2 Hz, 2H), 2.56 (t, J = 6.0 Hz, 2H), 2.51 (s, 6H), 2.46 (s, 3H), 2.32 (s, 3H), 1.92 (s, 3H), 1.73-1.67 (m, 4H) ppm; ^{13}C NMR (100 MHz, DMSO-*d*6) δ 146.4, 145.9, 143.2, 141.6, 141.4, 137.6, 134.8, 131.5, 130.4, 130.1, 128.0, 126.5, 122.2, 116.0, 32.4, 27.3, 26.1, 22.0, 21.6, 21.3, 20.5, 12.5 ppm; IR (thin film) 3415, 2932, 1592, 1444, 1382, 1281, 1246, 1199, 1158, 1027, 909, 863, 776, 727, 636, 544, 507 (cm^{-1}); HRMS (ESI) m/z: calcd for $C_{27}H_{30}IO_3S$ $[M - OTf]^+$, 561.0955; found 561.0960.

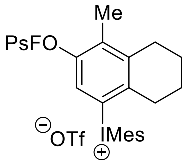

1z-FPs

Compound 1z-FPs: The synthesis of compound **1z-FPs** was followed by Procedure B. 527 mg (72%) of compound **1z-FPs** was isolated as a white solid. Mp: 138-139 °C; ^{1}H NMR (400 MHz, DMSO-*d*6) δ 8.02-7.95 (m, 1H), 7.45-7.41 (m, 3H), 7.20 (s, 2H), 2.82 (t, J = 5.6 Hz, 2H), 2.61 (t, J = 5.6 Hz, 2H), 2.47 (s, 6H), 2.31 (s, 3H), 2.07 (s, 3H), 1.74-1.68 (m, 4H) ppm; ^{13}C NMR (100 MHz, DMSO-*d*6) δ 158.9 (dd, J_1 = 260.0 Hz, J_2 = 2.7 Hz), 145.7, 143.4, 141.9, 141.7, 139.1 (t, J = 11.3 Hz), 138.3, 134.9, 130.2, 126.0, 121.8, 120.7 (q, J = 320.1 Hz), 116.0, 114.4-114.1 (m), 112.6 (t, J = 15.4 Hz), 32.5, 27.5, 26.1, 22.0, 21.7, 20.6, 12.6 ppm; IR (thin film) 3415, 3082, 2946, 1613, 1586, 1473, 1381, 1285, 1242, 1201, 1173, 1025, 1006, 906, 807, 698, 634, 530, 509 (cm^{-1}); HRMS (ESI) m/z: calcd for $C_{26}H_{26}F_2IO_3S$ $[M - OTf]^+$, 583.0610; found 583.0615.

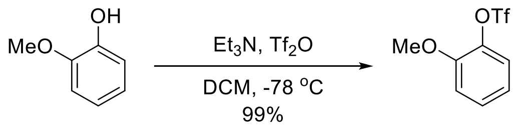

2-Methoxyphenyl triflate: To a solution of commercially available 2-methoxyphenol (1.0 g, 8.06 mmol, 1.0 equiv) in anhydrous DCM (20 mL) at -78 ℃ was added Et_3N (1.67 mL, 12.09 mmol, 1.5 equiv). After stirred for 5 minutes, Tf_2O (1.50 mL, 8.87 mmol, 1.1 equiv) was added slowly. After 10 minutes, all the volatiles were directly removed on a rotary evaporator. Flash column chromatography on silica gel afforded 2.04 g (99%) of 2-methoxyphenyl triflate as a colorless oil, the ^1H NMR of which is identical with that reported in literature.[26] ^1H NMR (400 MHz, $CDCl_3$) δ 7.73 (dd, J_1 = 8.2 Hz, J_2 = 1.6 Hz, 1H), 7.22 (dd, J_1 = 8.3 Hz, J_2 = 1.4 Hz, 1H), 6.98 (m, 1H), 3.90 (s, 3H) ppm.

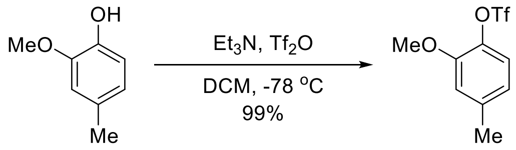

2-Methoxy-4-methylphenyl triflate: To a solution of commercially available 2-methoxy-4-methylphenol (1.0 g, 7.25 mmol, 1.0 equiv) in anhydrous DCM (20 mL) at -78 ℃ was added Et_3N (1.50 mL, 10.88 mmol, 1.5 equiv). After stirred for 5 minutes, Tf_2O (1.35 mL, 7.98 mmol, 1.1 equiv) was added slowly. After 10 minutes, all the volatiles were directly removed on a rotary evaporator. Flash column chromatography on silica gel afforded 1.94 g (99%) of 2-methoxy-4-methylphenyl triflate as a colorless oil, the ^1H NMR of which is identical with that reported in literature.[27] ^1H NMR (400 MHz, $CDCl_3$) δ 7.08 (d, J = 8.3 Hz, 1H), 6.84 (d, J = 1.4 Hz, 1H), 6.76 (m, 1H), 3.90 (s, 3H), 2.37 (s, 3H) ppm.

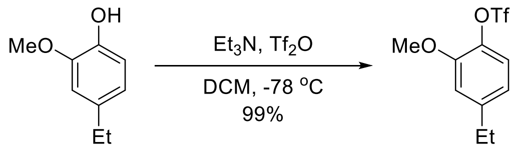

4-Ethyl-2-methoxyphenyl triflate: To a solution of commercially available 4-ethyl-2-methoxyphenol (1.0 g, 6.58 mmol, 1.0 equiv) in anhydrous DCM (20 mL) at -78 ℃ was added Et_3N (1.36 mL, 9.87 mmol, 1.5 equiv). After stirred for 5 minutes, Tf_2O (1.22 mL, 7.24 mmol, 1.1 equiv) was added slowly. After 10 minutes, all the volatiles were directly removed on a rotary evaporator. Flash column chromatography on silica gel afforded 1.85 g (99%) of 4-ethyl-2-methoxyphenyl triflate as a colorless oil, the ^1H NMR of which is identical with that reported in literature.[28] ^1H NMR (400 MHz, $CDCl_3$) δ 7.10 (d, J = 8.3 Hz, 1H), 6.86 (d, J = 2.0 Hz, 1H), 6.80 (m, 1H), 3.89 (s, 3H), 2.66 (q, J = 7.6 Hz, 2H), 1.26 (t, J = 7.5 Hz, 3H) ppm.

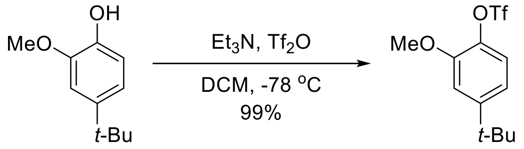

4-(*tert*-Butyl)-2-methoxyphenyl triflate: To a solution of commercially available 4-(*tert*-butyl)-2-methoxyphenol (1.0 g, 5.56 mmol, 1.0 equiv) in anhydrous DCM (20 mL) at -78 ℃ was added Et_3N (1.15 mL, 8.34 mmol, 1.5 equiv). After stirred for 5 minutes, Tf_2O (1.03 mL, 6.12 mmol, 1.1 equiv) was added slowly. After 10 minutes, all the volatiles were directly removed on a rotary evaporator. Flash column chromatography on silica gel afforded 1.72 g (99%) of 4-(*tert*-butyl)-2-methoxyphenyl triflate as a colorless oil. ^{1}H NMR (400 MHz, $CDCl_3$) δ 7.26 (d, J = 8.8 Hz, 1H), 6.99 (d, J = 3.2 Hz, 1H), 6.75 (dd, J_1 = 9.2 Hz, J_2 = 3.2 Hz, 1H), 3.81 (s, 3H), 1.42 (s, 9H) ppm; ^{13}C NMR (100 MHz, $CDCl_3$) δ 158.4, 143.4, 142.9, 122.3, 118.6 (q, J = 317.8 Hz), 115.3, 111.2, 55.8, 35.1, 30.4 ppm; IR (thin film) 3458, 2963, 1584, 1483, 1417, 1301, 1214, 1142, 1054, 876, 805, 622, 588, 495 (cm^{-1}); HRMS (ESI) m/z: calcd for $C_{12}H_{15}F_3KO_4S$ $[M + K]^+$, 351.0275; found 351.0276.

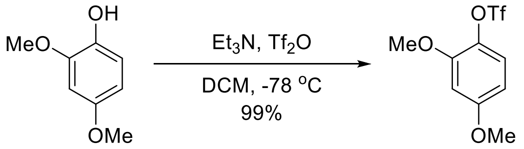

2,4-Dimethoxyphenyl triflate: To a solution of commercially available 2,4-dimethoxyphenol (1.0 g, 6.49 mmol, 1.0 equiv) in anhydrous DCM (20 mL) at -78 ℃ was added Et_3N (1.34 mL, 9.74 mmol, 1.5 equiv). After stirred for 5 minutes, Tf_2O (1.20 mL, 6.12 mmol, 1.1 equiv) was added slowly. After 10 minutes, all the volatiles were directly removed on a rotary evaporator. Flash column chromatography on silica gel afforded 1.72 g (99%) of 2,4-dimethoxyphenyl triflate as a colorless oil. ^{1}H NMR (400 MHz, $CDCl_3$) δ 7.12 (d, J = 9.2 Hz, 1H), 6.56 (d, J = 2.8 Hz, 1H), 6.44 (dd, J_1 = 9.2 Hz, J_2 = 2.8 Hz, 1H), 3.87 (s, 3H), 3.80 (s, 3H) ppm; ^{13}C NMR (100 MHz, $CDCl_3$) δ 160.3, 152.4, 132.8, 122.9, 119.0 (q, J = 318.5 Hz), 104.3, 100.5, 56.2, 55.8 ppm; IR (thin film) 3438, 3013, 2961, 2839, 1612, 1508, 1421, 1248, 1213, 1170, 1142, 1110, 1035, 877, 822, 619, 582, 492 (cm^{-1}); HRMS (ESI) m/z: calcd for $C_9H_9F_3NaO_5S$ $[M + Na]^+$, 309.0015; found 309.0005.

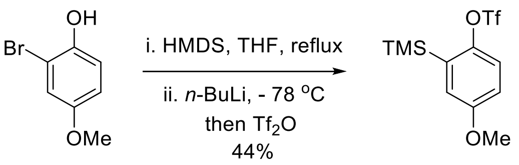

4-Methoxy-2-(trimethylsilyl)phenyl triflate: To a solution of commercially available 2-bromo-4-methoxyphenol (1.0 g, 4.95 mmol, 1.0 equiv) in anhydrous THF (30 mL) was added HMDS (2.06 mL, 9.90 mmol, 2.0 equiv) under inert atmosphere. The reaction mixture was refluxed for two hours. After cooling to room temperature, the resulting solution was concentrated. A crude oil was obtained, which was used directly in the next step without further purification.
To the above crude oil in anhydrous THF (30 mL) under inert atmosphere at -78 ℃ was added *n*-BuLi (2.5 M in hexane, 2.18 mL, 5.45 mmol, 1.1 equiv) dropwise. The resulting solution was stirred at -78 ℃ for 40 min. Tf_2O (0.92 mL, 5.45 mmol, 1.1 equiv) was added to the mixture dropwise and the reaction was stirred for another 40 min. It was then poured into water (20 mL). The resulting mixture was extracted with DCM (20 mL x 3). The combined organic layers were washed with brine (20 mL), dried over Na_2SO_4, filtered, and concentrated. Flash column chromatography on silica gel afforded 0.71 g (44%) of 4-methoxy-2-(trimethylsilyl)phenyl triflate as a colorless oil, the ^{1}H NMR of which is identical with that reported in literature.[29] ^{1}H NMR (400 MHz, $CDCl_3$) δ 7.15 (d, J = 8.8 Hz, 1H), 6.92 (d, J = 3.2 Hz, 1H), 6.77-6.80 (m, 1H), 3.70 (s, 3H), 0.27 (s, 9H) ppm.

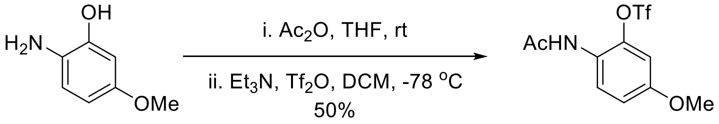

2-Acetamido-5-methoxyphenyl triflate: To a solution of commercially available 2-amino-5-methoxyphenol (1.0 g, 7.19 mmol, 1.0 equiv) in anhydrous THF (30 mL) at room temperature was added acetic anhydride (0.74 mL, 9.91 mmol, 1.1 equiv). After stirred for three hours, it was poured into water (20 mL). The resulting mixture was extracted with DCM (20 mL x 3). The combined organic layers were washed with brine (20 mL), dried over Na_2SO_4, filtered, and concentrated. A crude oil was obtained, which was used directly in the next step without further purification.
To the above crude oil in anhydrous DCM (20 mL) at -78 ℃ was added Et_3N (1.48 mL, 10.79 mmol, 1.5 equiv). After stirred for 5 minutes, Tf_2O (1.55 mL, 7.91 mmol, 1.1 equiv) was added slowly. After 10 minutes, all the volatiles were directly removed on a rotary evaporator. Flash column chromatography on silica gel afforded 1.13 g (50%) of 2-acetamido-5-methoxyphenyl triflate as a colorless oil. ^{1}H NMR (400 MHz, $CDCl_3$) δ 7.87 (s, 1H), 7.32 (s, 1H), 7.18 (d, J = 9.2 Hz, 1H), 6.67 (dd, J_1 = 9.2 Hz, J_2 = 2.8 Hz, 1H), 3.82 (s, 3H), 2.22 (s, 3H) ppm; ^{13}C NMR (100 MHz, $CDCl_3$) δ 168.7, 159.6, 132.8, 131.5, 122.3, 118.8 (q, J = 318.4 Hz), 111.1, 108.7, 56.0, 24.7 ppm; IR (thin film) 3439, 3250, 3065, 3007, 1669, 1611, 1556, 1489, 1449, 1422, 1381, 1321, 1214, 1134, 1036, 886, 842, 805, 724, 618, 509, 486 (cm^{-1}); HRMS (ESI) m/z: calcd for $C_{10}H_{10}F_3NNaO_5S$ $[M + Na]^+$, 336.0124; found 336.0122.

2.4. General procedure for the synthesis of 1cc-1jj (Procedure D):
To a solution of compound **4** (0.22 mmol, 1.1 equiv) in anhydrous 2,2,2-trifluoroethyl

alcohol (2 mL) at room temperature was added Koser's reagent **5** (78.4 mg, 0.2 mmol, 1.0 equiv). After eight hours, it was filtrated. All the volatiles were then removed on a rotary evaporator. The resulting crude oil was triturated with diethyl ether and isolated by filtration to afford pure aryne precursor **1**.

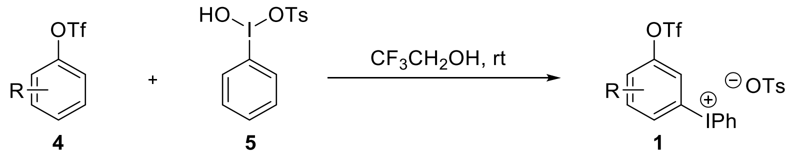

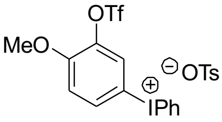

1cc

Compound 1cc: The synthesis of compound **1cc** was followed by Procedure D. 103 mg (82%) of compound **1cc** was isolated as a white solid. Mp: 175-176 °C; ^{1}H NMR (400 MHz, DMSO-*d6*) δ 8.53 (d, J = 1.6 Hz, 1H), 8.35 (dd, J_1 = 8.8 Hz, J_2 = 2.0 Hz, 1H), 8.25 (d, J = 7.6 Hz, 2H), 7.66 (t, J = 7.6 Hz, 1H), 7.54-7.45 (m, 5H), 7.11 (d, J = 7.6 Hz, 2H), 3.93 (s, 3H), 2.28 (s, 3H) ppm; ^{13}C NMR (100 MHz, DMSO-*d6*) δ 153.8, 145.7, 137.9, 137.7, 137.6, 134.9, 132.0, 131.7, 129.4, 128.0, 125.5, 118.8 (q, J = 320.1 Hz), 117.5, 117.0, 104.5, 57.1, 20.7 ppm; IR (thin film) 3447, 2961, 1592, 1495, 1434, 1276, 1245, 1172, 1140, 1121, 1026, 900, 851, 744, 637, 570, 515 (cm^{-1}); HRMS (ESI) m/z: calcd for $C_{14}H_{11}F_3IO_4S$ [M - OTs]$^+$, 458.9369; found 458.9370.

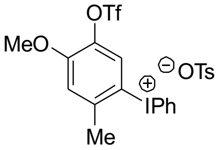

1dd

Compound 1dd: The synthesis of compound **1dd** was followed by Procedure D. 109 mg (85%) of compound **1dd** was isolated as a white solid. Mp: 152-153 °C; ^{1}H NMR (400 MHz, DMSO-*d6*) δ 8.66 (s, 1H), 8.22 (d, J = 7.6 Hz, 2H), 7.65 (t, J = 7.2 Hz, 1H), 7.54-7.50 (m, 3H), 7.47 (d, J = 8.0 Hz, 2H), 7.10 (d, J = 8.0 Hz, 2H), 3.92 (s, 3H), 2.64 (s, 3H), 2.28 (s, 3H) ppm; ^{13}C NMR (100 MHz, DMSO-*d6*) δ 153.6, 145.4, 144.1, 137.9, 136.0, 135.0, 132.1, 131.8, 130.6, 128.1, 125.5, 118.2 (q, J = 318.9 Hz), 116.9, 116.1, 109.1, 57.1, 25.0, 20.8 ppm; IR (thin film) 3444, 3045, 1592, 1494, 1421, 1376, 1308, 1218, 1159, 1126, 1008, 878, 818, 751, 679, 641, 608, 563, 498 (cm^{-1}); HRMS (ESI) m/z: calcd for $C_{15}H_{13}F_3IO_4S$ [M - OTs]$^+$, 427.9526; found 472.9522.

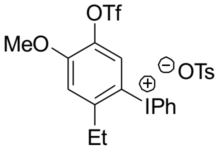

1ee

Compound 1ee: The synthesis of compound **1ee** was followed by Procedure D. 105 mg (80%) of compound **1ee** was isolated as a white solid. Mp: 174-175 °C; ^{1}H NMR (400 MHz, DMSO-*d6*) δ 8.72 (s, 1H), 8.21 (d, *J* = 7.6 Hz, 2H), 7.65 (t, *J* = 7.6 Hz, 1H), 7.52 (t, *J* = 8.0 Hz, 2H), 7.48-7.46 (m, 3H), 7.11 (d, *J* = 8.0 Hz, 2H), 3.95 (s, 3H), 2.93 (q, *J* = 7.2 Hz, 2H), 2.28 (s, 3H), 1.19 (t, *J* = 7.6 Hz, 3H) ppm; ^{13}C NMR (100 MHz, DMSO-*d6*) δ 154.0, 149.2, 145.4, 137.9, 136.2, 134.8, 132.1, 131.8, 131.0, 128.1, 125.4, 118.2 (q, *J* = 318.7 Hz), 117.0, 114.9, 108.6, 57.1, 31.6, 20.8, 15.1 ppm; IR (thin film) 3438, 2981, 2935, 1593, 1489, 1425, 1381, 1283, 1245, 1215, 1160, 1124, 1004, 872, 810, 741, 680, 566, 492, 449 (cm^{-1}); HRMS (ESI) m/z: calcd for $C_{16}H_{15}F_{3}IO_{4}S$ $[M - OTs]^{+}$, 486.9682; found 486.9672.

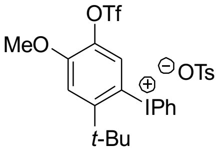

1ff

Compound 1ff: The synthesis of compound **1ff** was followed by Procedure D. 103 mg (75%) of compound **1ff** was isolated as a white solid. Mp: 106-107 °C; ^{1}H NMR (400 MHz, DMSO-*d6*) δ 8.20-8.18 (m, 3H), 7.65 (t, *J* = 7.6 Hz, 1H), 7.53-7.46 (m, 4H), 7.24 (s, 1H), 7.11 (d, *J* = 8.0 Hz, 2H), 3.99 (s, 3H), 2.28 (s, 3H), 1.37 (s, 9H) ppm; ^{13}C NMR (100 MHz, DMSO-*d6*) δ 155.9, 148.3, 145.4, 141.1, 137.9, 135.5, 132.2, 131.7, 129.1, 128.2, 125.5, 117.9 (q, *J* = 318.3 Hz), 116.3, 112.5, 103.4, 57.6, 38.6, 29.7, 20.8 ppm; IR (thin film) 3443, 3062, 2958, 1586, 1477, 1406, 1365, 1231, 1167, 1124, 1072, 1033, 1007, 932, 873, 813, 737, 678, 566, 501 (cm^{-1}); HRMS (ESI) m/z: calcd for $C_{18}H_{19}F_{3}IO_{4}S$ $[M - OTs]^{+}$, 514.9995; found 514.9991.

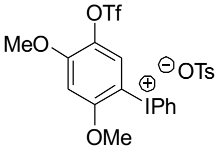

1gg

Compound 1gg: The synthesis of compound **1gg** was followed by Procedure D. 107 mg (81%) of compound **1gg** was isolated as a white solid. Mp: 182-183 °C; ^{1}H NMR (400 MHz, DMSO-*d6*) δ 8.57 (s, 1H), 8.12 (d, *J* = 7.2 Hz, 2H), 7.64 (t, *J* = 7.6 Hz, 1H), 7.52-7.46 (m, 4H), 7.12-7.09 (m, 3H), 4.02 (s, 3H), 3.99 (s, 3H), 2.28 (s, 3H) ppm; ^{13}C NMR (100 MHz, DMSO-*d6*) δ 158.2, 155.8, 145.7, 137.7, 134.9, 132.0, 131.6, 130.3, 128.1, 125.5, 118.2 (q, *J* = 318.8 Hz), 116.7, 98.8, 94.4, 58.1, 57.4, 20.8 ppm; IR (thin film) 3421, 3056, 2961, 1593, 1494, 1471, 1415, 1391, 1317, 1238, 1210, 1171, 1123, 1008, 889, 819, 734, 681, 604, 565, 507, 449 (cm^{-1}); HRMS (ESI) m/z: calcd for $C_{15}H_{13}F_{3}IO_{5}S$ $[M - OTs]^{+}$, 488.9475; found 488.9480.

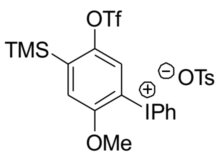

1hh

Compound 1hh: The synthesis of compound **1hh** was followed by Procedure D. 84 mg (60%) of compound **1hh** was isolated as a white solid. Mp: 133-134 °C; ^{1}H NMR (400 MHz, DMSO-*d*6) δ 8.30 (s, 1H), 8.20 (d, *J* = 7.2 Hz, 2H), 7.65 (t, *J* = 7.2 Hz, 1H), 7.51 (d, *J* = 8.0 Hz, 2H), 7.47 (d, *J* = 8.0 Hz, 2H), 7.27 (s, 1H), 7.10 (d, *J* = 8.0 Hz, 2H), 4.00 (s, 3H), 2.28 (s, 3H), 0.35 (s, 9H) ppm; ^{13}C NMR (100 MHz, DMSO-*d*6) δ 155.3, 146.5, 145.6, 140.2, 137.6, 135.4, 132.1, 131.6, 128.1, 128.0, 125.5, 118.6, 117.9 (q, *J* = 318.5 Hz), 116.3, 107.8, 57.6, 20.7, -1.15 ppm; IR (thin film) 3417, 2914, 2859, 1636, 1459, 1404, 1341, 1228, 1165, 1134, 1065, 1034, 1004, 918, 844, 810, 739, 681, 605, 556 (cm^{-1}); HRMS (ESI) m/z: calcd for $C_{17}H_{19}F_3IO_4SSi$ [M - OTs]$^+$, 530.9765; found 530.9768.

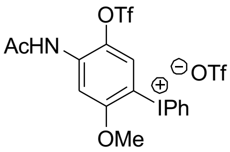

1ii

Compound 1ii: To a suspension of 2-acetamido-5-methoxyphenyl triflate (68.9 mg, 0.22 mmol, 1.1 equiv) and NaOTf (172 mg, 1.0 mmol, 5.0 equiv) in anhydrous DCM (2 mL) at room temperature was added Koser's reagent **5** (78.4 mg, 0.2 mmol, 1.0 equiv). After eight hours, it was filtrated. All the volatiles were then removed on a rotary evaporator. The resulting crude oil was triturated with diethyl ether and isolated by filtration to afford 39.9 mg (30%) of compound **1ii** as a viscous oil. ^{1}H NMR (400 MHz, $CDCl_3$) δ 8.37 (s, 1H), 7.97 (d, *J* = 7.6 Hz, 2H), 7.67 (t, *J* = 6.4 Hz, 1H), 7.58 (s, 1H), 7.55 (s, 1H), 7.50 (t, *J* = 7.6 Hz, 2H), 3.99 (s, 3H), 2.27 (s, 3H) ppm; ^{13}C NMR (100 MHz, acetone-*d*6) δ 169.9, 158.3, 138.5, 136.5, 134.9, 133.6 (d, *J* = 2.4 Hz), 133.0 (d, *J* = 1.8 Hz), 131.3, 119.5 (q, *J* = 318.3 Hz), 115.5 (d, *J* = 3.3 Hz), 108.1, 108.0, 58.4, 24.1 ppm; IR (thin film) 3417, 2940, 2859, 1620, 1424, 1384, 1215, 1135, 1034, 880, 819, 735, 689, 634, 559 (cm^{-1}); HRMS (ESI) m/z: calcd for $C_{19}H_{20}F_3INO_5S$ [M - OTf]$^+$, 558.0054; found 558.0052.

2.5. Domino 1,2-benzdiyne reactions with 1b-1d:

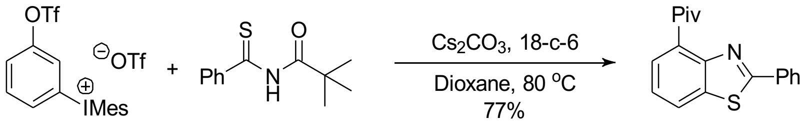

To a suspension of benzothioamide **6** (44.2 mg, 0.2 mmol, 1.0 equiv), Cs_2CO_3 (520 mg, 1.6 mmol, 8.0 equiv), and 18-c-6 (105.6 mg, 0.4 mmol, 2.0 equiv) in 1,4-dioxane (3 mL) at 80 °C was added a solution of **1b** (248 mg, 0.4 mmol, 2.0 equiv) in

1,4-dioxane (3 mL) over 8 hours via a syringe pump. All the volatiles were removed directly on a rotary evaporator. Flash column chromatography on silica gel afforded 45.4 mg (77%) of **7a** as a white solid, the ^{1}H NMR of which is identical with that reported in literature.[30] ^{1}H NMR (400 MHz, $CDCl_3$) δ 8.08-8.06 (m, 2H), 7.92 (d, *J* = 8.0 Hz, 1H), 7.49-7.48 (m, 3H), 7.38 (t, *J* = 7.6 Hz, 1H), 7.28 (d, *J* = 6.5 Hz, 1H), 1.37 (s, 9H) ppm.

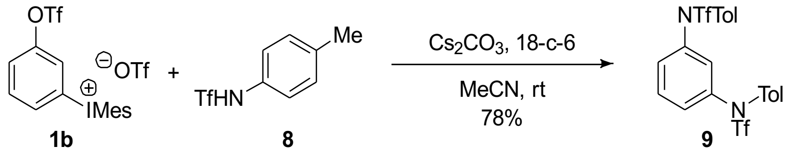

To a suspension of *N*-triflated aniline **8** (95.6 mg, 0.4 mmol, 1.0 equiv), Cs_2CO_3 (520 mg, 1.6 mmol, 8.0 equiv), and 18-c-6 (105.6 mg, 0.4 mmol, 2.0 equiv) in MeCN (10 mL) at room temperature was added a solution of **1b** (248 mg, 0.4 mmol, 1.0 equiv) in MeCN (10 mL) over 8 hours via a syringe pump. All the volatiles were removed directly on a rotary evaporator. Flash column chromatography on silica gel afforded 34.8 mg (78%) of **9** as a white solid, the ^{1}H NMR of which is identical with that reported in literature.[31] ^{1}H NMR (400 MHz, $CDCl_3$) δ 7.58 (s, 1H), 7.37 (m, 3H), 7.30 (d, *J* = 8.0 Hz, 4H), 7.22 (d, *J* = 7.6 Hz, 4H), 2.36 (s, 6H) ppm.

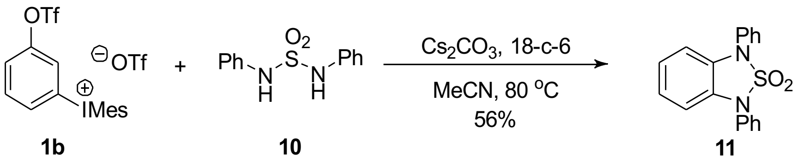

To a suspension of sulfonamide **10** (49.6 mg, 0.2 mmol, 1.0 equiv), Cs_2CO_3 (520 mg, 1.6 mmol, 8.0 equiv), and 18-c-6 (105.6 mg, 0.4 mmol, 2.0 equiv) in MeCN (10 mL) at 80 ℃was added a solution of **1b** (248 mg, 0.4 mmol, 1.0 equiv) in MeCN (10 mL) over 8 hours via a syringe pump. All the volatiles were removed directly on a rotary evaporator. Flash column chromatography on silica gel afforded 36.1 mg (56%) of **11** as a white solid, the ^{1}H NMR of which is identical with that reported in literature.[32] ^{1}H NMR (400 MHz, $CDCl_3$) δ 7.66 (d, *J* = 7.5 Hz, 4H), 7.57 (t, *J* = 7.5 Hz, 4H), 7.51 (t, *J* = 7.5 Hz, 2H), 6.94 (m, 2H), 6.68 (m, 2H) ppm.

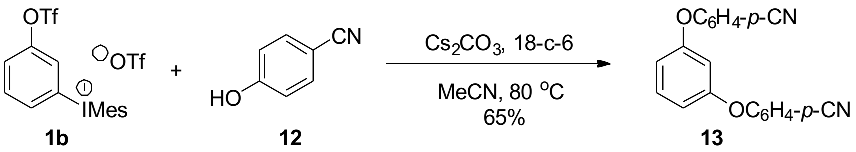

To a suspension of *p*-cyanophenol **12** (47.6 mg, 0.4 mmol, 1.0 equiv), Cs_2CO_3 (520 mg, 1.6 mmol, 8.0 equiv), and 18-c-6 (105.6 mg, 0.4 mmol, 2.0 equiv) in MeCN (10 mL) at room temperature was added a solution of **1b** (248 mg, 0.4 mmol, 1.0 equiv) in MeCN (10 mL) over 8 hours via a syringe pump. All the volatiles were removed

directly on a rotary evaporator. Flash column chromatography on silica gel afforded 40.6 mg (65%) of **13** as a colorless oil. ^1H NMR (400 MHz, $CDCl_3$) δ 7.63 (d, J = 8.4 Hz, 4H), 7.42 (t, J = 8.4 Hz, 1H), 7.05 (d, J = 8.4 Hz, 4H), 6.91 (dd, J_1 = 8.0 Hz, J_2 = 2.0 Hz, 2H), 6.79 (s, 1H) ppm; ^{13}C NMR (100 MHz, $CDCl_3$) δ 160.9, 156.7, 134.5, 131.6, 118.8, 118.6, 116.6, 112.4, 106.9 ppm; IR (thin film) 3444, 2226, 1592, 1501, 1478, 1420, 1384, 1265, 1234, 1169, 1124, 966, 842, 779, 686, 651, 544 (cm^{-1}); HRMS (ESI) m/z: calcd for $C_{20}H_{12}N_2NaO_2$ $[M + Na]^+$ 335.0791; found 335.0798.

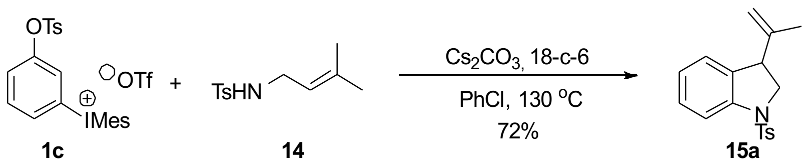

1c **14** **15a**

A suspension of tosylamide **14** (47.8 mg, 0.2 mmol, 1.0 equiv), Cs_2CO_3 (520 mg, 1.6 mmol, 8.0 equiv), and 18-c-6 (105.6 mg, 0.4 mmol, 2.0 equiv) in anhydrous chlorobenzene (3 mL) under inert atmosphere was heated at 130 ℃ for 30 minutes. A solution of **1c** (256.8 mg, 0.4 mmol, 2.0 equiv) in chlorobenzene (3 mL) was then added to this suspension over 8 hours via a syringe pump. After addition, it was heated for additional 4 hours. All the volatiles were removed directly on a rotary evaporator. Flash column chromatography on silica gel afforded 45.1 mg (72%) of **15a** as a white solid, the ^1H NMR of which is identical with that reported in literature.[33] ^1H NMR (400 MHz, $CDCl_3$) δ 7.70-7.66 (m, 3H), 7.26-7.21 (m, 3H), 6.98-6.97 (m, 2H), 4.77 (s, 1H), 4.72 (s, 1H), 4.04 (t, J = 10.4 Hz, 1H), 3.87-3.83 (m, 1H), 3.69 (dd, J_1 = 10.4 Hz, J_2 = 6.8 Hz, 1H), 2.37 (s, 3H), 1.39 (s, 3H) ppm.

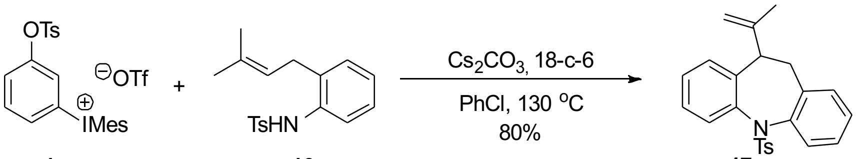

1c **16** **17**

A suspension of tosylamide **16** (63 mg, 0.2 mmol, 1.0 equiv), Cs_2CO_3 (520 mg, 1.6 mmol, 8.0 equiv), and 18-c-6 (105.6 mg, 0.4 mmol, 2.0 equiv) in anhydrous chlorobenzene (10 mL) under inert atmosphere was heated at 130 ℃ for 30 minutes. A solution of **1c** (256.8 mg, 0.4 mmol, 2.0 equiv) in chlorobenzene (10 mL) was then added to this suspension over 8 hours via a syringe pump. After addition, it was heated for additional 4 hours. All the volatiles were removed directly on a rotary evaporator. Flash column chromatography on silica gel afforded 62.2 mg (80%) of **17** as a white solid, the ^1H NMR of which is identical with that reported in literature.[33] ^1H NMR (500 MHz, $CDCl_3$) δ 7.63 (d, J = 8.5 Hz, 2H), 7.58-7.51 (m, 2.7H), 7.41 (d, J = 7.0 Hz, 1H), 7.25-7.19 (m, 7H), 7.18-7.11 (m, 3.7H), 7.05 (d, J = 7.0 Hz, 1H), 4.92 (s, 0.43H), 4.88 (s, 1H), 4.83 (s, 1H), 4.58 (s, 0.42H), 3.72-3.67 (m, 0.42H), 3.54-3.50 (m, 1H), 3.26 (t, J = 13.5 Hz, 1H), 3.18-3.11 (m, 0.43H), 2.70-2.61 (m, 0.42H), 2.42-2.37 (m, 5.3H), 1.45 (s, 3H), 1.36 (s, 1.2H) ppm.

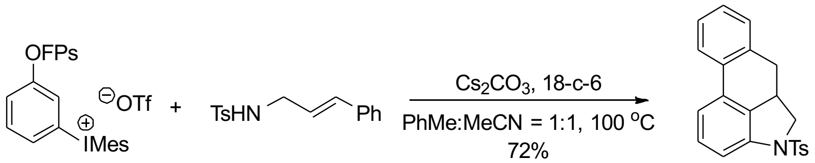

1d **18** **19a**

A suspension of cinnamyl amide **18** (57.4 mg, 0.2 mmol, 1.0 equiv), Cs_2CO_3 (520 mg, 1.6 mmol, 8.0 equiv), and 18-c-6 (26.4 mg, 0.4 mmol, 0.5 equiv) in anhydrous toluene (7 mL) under inert atmosphere was heated at 100 °C for 30 minutes. A solution of **1d** (265.6 mg, 0.4 mmol, 2.0 equiv) in MeCN (7 mL) was then added to this suspension over 4 hours via a syringe pump. After addition, it was heated for additional one hours. All the volatiles were removed directly on a rotary evaporator. Flash column chromatography on silica gel afforded 52 mg (72%) of **19a** as a white solid, the ^{1}H NMR of which is identical with that reported in literature.[34] ^{1}H NMR (400 MHz, $CDCl_3$) δ 7.73 (d, J = 8.4 Hz, 2H), 7.71 (d, J = 8.4 Hz, 1H), 7.49 (d, J = 8.0 Hz, 1H), 7.34 (d, J = 8.0 Hz, 1H), 7.29 (t, J = 7.6 Hz, 2H), 7.23 (d, J = 8.4 Hz, 2H), 7.22-7.17 (m, 2H), 4.41 (t, J = 9.6 Hz, 1H), 3.49 (t, J = 10.8 Hz, 1H), 3.36-3.28 (m, 1H), 2.99 (dd, J_1 = 14.8 Hz, J_2 = 6.0 Hz,1H), 2.61 (t, J = 14.8 Hz, 1H), 2.35 (s, 3H) ppm.

2.6. General procedure for the synthesis of 7b-7i (Procedure E): To a solution of benzothioamide **6** (44.2 mg, 0.2 mmol, 1.0 equiv), Cs_2CO_3 (520 mg, 1.6 mmol, 8.0 equiv), and 18-c-6 (105.6 mg, 0.4 mmol, 2.0 equiv) in anhydrous dioxane (3 mL) at 80 ℃ was added slowly a solution of **1s-1z** (0.4 mmol, 2.0 equiv) in anhydrous dioxane (3 mL) over 8 hours via a syringe pump. All the volatiles were removed directly on a rotary evaporator. Flash column chromatography with pet ether and EtOAc afforded products **7b-7i**.

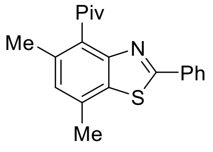

7b

Compound 7b: The synthesis of compound **7b** was followed by Procedure E. 33 mg (51%) of compound **7b** was isolated as a colorless oil. ^{1}H NMR (400 MHz, $CDCl_3$) δ 8.07-8.05 (m, 2H), 7.48-7.46 (m, 3H), 7.03 (s, 1H), 2.56 (s, 3H), 2.34 (s, 3H), 1.35 (s, 9H) ppm; ^{13}C NMR (100 MHz, $CDCl_3$) δ 216.3, 167.5, 150.3, 134.2, 133.9, 131.6, 131.5, 131.1, 129.2, 128.2, 127.7, 45.2, 28.0, 21.5, 19.8 ppm; IR (thin film) 3446, 2925, 1687, 1476, 1443, 1383, 1258, 1177, 1091, 1052, 948, 861, 805, 766, 691, 636 (cm^{-1}); HRMS (ESI) m/z: calcd for $C_{20}H_{22}NOS$ $[M + H]^+$, 324.1417; found 324.1417.

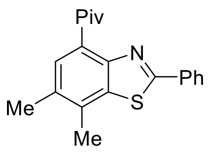

7c

Compound 7c: The synthesis of compound **7c** was followed by Procedure E. 38 mg (59%) of compound **7c** was isolated as a colorless oil. ^{1}H NMR (400 MHz, $CDCl_3$) δ 8.08-8.05 (m, 2H), 7.48-7.46 (m, 3H), 7.09 (s, 1H), 2.53 (s, 3H), 2.42 (s, 3H), 1.36 (s, 9H) ppm; ^{13}C NMR (100 MHz, $CDCl_3$) δ 213.7, 166.8, 148.3, 137.2, 134.1, 134.0, 132.8, 131.1, 130.6, 129.1, 127.7, 126.2, 45.2, 27.5, 19.8, 19.6 ppm; IR (thin film) 3447, 2920, 1739, 1635, 1409, 1382, 1163, 1109, 831, 773 (cm^{-1}); HRMS (ESI) m/z: calcd for $C_{20}H_{21}NNaOS$ $[M + Na]^+$, 346.1236; found 346.1238.

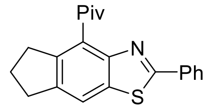

7d

Compound 7d: The synthesis of compound **7d** was followed by Procedure E. 41 mg (61%) of compound **7d** was isolated as a colorless oil. ^{1}H NMR (400 MHz, $CDCl_3$) δ 8.04-8.02 (m, 2H), 7.70 (s, 1H), 7.48-7.44 (m, 3H), 3.03 (t, J = 7.2 Hz, 2H), 2.92 (t, J = 7.6 Hz, 2H), 2.20-2.13 (m, 2H), 1.36 (s, 9H) ppm; ^{13}C NMR (100 MHz, $CDCl_3$) δ 215.2, 166.8, 149.1, 143.2, 139.3, 134.0, 133.7, 132.9, 130.9, 129.1, 127.5, 117.1, 45.1, 32.9, 31.8, 27.8, 26.4 ppm; IR (thin film) 3446, 2926, 1687, 1636, 1475, 1383, 1325, 1262, 1217, 1059, 1013, 953, 877, 848, 796, 761, 688, 637 (cm^{-1}); HRMS (ESI) m/z: calcd for $C_{21}H_{21}NNaOS$ $[M + Na]^+$, 358.1236; found 358.1234.

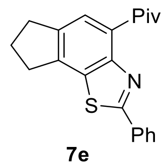

7e

Compound 7e: The synthesis of compound **7e** was followed by Procedure E. 36 mg (54%) of compound **7e** was isolated as a colorless oil. ^{1}H NMR (400 MHz, $CDCl_3$) δ 8.08-8.05 (m, 2H), 7.48-7.46 (m, 3H), 7.16 (s, 1H), 3.09 (q, J = 7.2 Hz, 4H), 2.32-2.24 (m, 2H), 1.37 (s, 9H) ppm; ^{13}C NMR (100 MHz, $CDCl_3$) δ 214.2, 166.9, 141.1, 137.8, 134.8, 133.9, 131.8, 131.1, 129.1, 127.7, 120.1, 45.2, 33.4, 33.4, 29.9, 27.5, 25.6 ppm; IR (thin film) 3449, 2923, 1684, 1633, 1474, 1384, 1262, 1183, 1033, 949, 868, 836, 810 (cm^{-1}); HRMS (ESI) m/z: calcd for $C_{21}H_{21}NNaOS$ $[M + Na]^+$, 358.1236; found 358.1241.

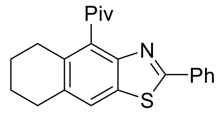

7f

Compound 7f: The synthesis of compound **7f** was followed by Procedure E. 52 mg (74%) of compound **7f** was isolated as a colorless oil. ^{1}H NMR (400 MHz, $CDCl_3$) δ 8.04-8.02 (m, 2H), 7.58 (s, 1H), 7.48-7.44 (m, 3H), 2.93 (m, 2H), 2.83-2.61 (m, 2H), 1.85-1.82 (m, 4H), 1.37 (s, 9H) ppm; ^{13}C NMR (100 MHz, $CDCl_3$) δ 216.8, 166.9, 148.5, 136.3, 136.0, 133.9, 132.7, 131.3, 131.0, 129.1, 127.6, 121.4, 45.0, 30.4, 28.1, 27.7, 23.0, 22.9 ppm; IR (thin film) 3447, 2929, 1688, 1479, 1442, 1404, 1359, 1229, 1178, 1090, 1027, 1004, 971, 942, 841, 763, 688 (cm^{-1}); HRMS (ESI) m/z: calcd for $C_{22}H_{23}NNaOS$ $[M + Na]^+$, 372.1393; found 372.1390.

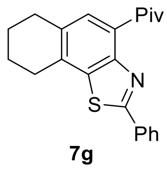

7g

Compound 7g: The synthesis of compound **7g** was followed by Procedure E. 32 mg (46%) of compound **7g** was isolated as a colorless oil. ^{1}H NMR (400 MHz, $CDCl_3$) δ 8.08-8.05 (m, 2H), 7.47-7.46 (m, 3H), 7.00 (s, 1H), 2.89 (m, 4H), 1.96-1.89 (m, 4H), 1.36 (s, 9H) ppm; ^{13}C NMR (100 MHz, $CDCl_3$) δ 213.9, 166.7, 148.0, 136.0, 134.1, 134.0, 133.9, 131.5, 131.0, 129.1, 127.7, 125.3, 45.1, 29.6, 29.5, 27.5, 23.3, 22.9 ppm; IR (thin film) 3449, 2923, 1684, 1633, 1474, 1384, 1262, 1183, 1033, 949, 868, 836, 810 (cm^{-1}); HRMS (ESI) m/z: calcd for $C_{22}H_{23}NNaOS$ $[M + Na]^+$, 372.1393; found 372.1391.

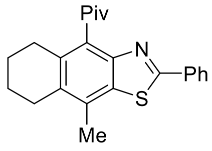

7h

Compound 7h: The synthesis of compound **7h** was followed by Procedure E. 40 mg (55%) of compound **7h** was isolated as a colorless oil. ^{1}H NMR (400 MHz, $CDCl_3$) δ 8.06-8.03 (m, 2H), 7.46-7.45 (m, 3H), 2.81-2.67 (m, 4H), 2.48 (s, 3H), 1.91-1.85 (m, 2H), 1.82-.77 (m, 2H), 1.36 (s, 9H) ppm; ^{13}C NMR (100 MHz, $CDCl_3$) δ 217.0, 166.4, 147.5, 134.4, 134.3, 134.1, 133.2, 131.5, 130.9, 129.4, 129.1, 127.6, 45.0, 28.5, 28.2, 27.6, 23.2, 22.7, 19.6 ppm; IR (thin film) 3443, 2237, 1646, 1381, 1265, 1169, 1027, 819, 764, 674, 637, 562 (cm^{-1}); HRMS (ESI) m/z: calcd for $C_{23}H_{25}NNaOS$ $[M + Na]^+$, 386.1549; found 386.1546.

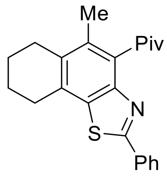

7i

Compound 7i: The synthesis of compound **7i** was followed by Procedure E. 36 mg (50%) of compound **7i** was isolated as a colorless oil. ^{1}H NMR (400 MHz, $CDCl_3$) δ 8.06-8.04 (m, 2H), 7.46-7.45 (m, 3H), 2.90 (t, J = 6.0 Hz, 2H), 2.72 (t, J = 5.6 Hz, 2H), 2.20 (s, 3H), 1.93-1.92 (m, 4H), 1.35 (s, 9H) ppm; ^{13}C NMR (100 MHz, $CDCl_3$) δ 216.8, 166.3, 147.8, 134.5, 134.1, 133.8, 133.4, 130.8, 130.5, 130.4, 129.1, 127.6, 45.3, 30.3, 28.0, 27.3, 23.5, 22.5, 17.4 ppm; IR (thin film) 3457, 2928, 2861, 1687, 1638, 1560, 1475, 1385, 1258, 1118, 989, 958, 934, 809, 764, 692 (cm^{-1}); HRMS (ESI) m/z: calcd for $C_{23}H_{25}NNaOS$ [M + Na]$^+$, 386.1549; found 386.1557.

2.7. General procedure for the synthesis of 15b-15i (Procedure F): A suspension of tosylamide **14** (47.8 mg, 0.2 mmol, 1.0 equiv), Cs_2CO_3 (520 mg, 1.6 mmol, 8.0 equiv), and 18-c-6 (105.6 mg, 0.4 mmol, 2.0 equiv) in anhydrous chlorobenzene (3 mL) under inert atmosphere was heated at 130 ℃ for 30 minutes. A solution of **1** (0.4 mmol, 2.0 equiv) in chlorobenzene (3 mL) was then added to this suspension over 8 hours via a syringe pump. After addition, it was heated for additional 4 hours. The resulting mixture was concentrated directly on rotary evaporator. Flash column chromatography with pet ether and EtOAc afforded product **15b-15i**.

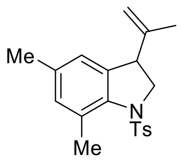

15b

Compound 15b: The synthesis of compound **15b** was followed by Procedure F. 35.5 mg (52%) of compound **15b** was isolated as a colorless oil. ^{1}H NMR (400 MHz, $CDCl_3$) δ 7.42 (d, J = 8.0 Hz, 2H), 7.17 (d, J = 8.0 Hz, 2H), 6.93 (s, 1H), 6.51 (s, 1H), 4.81 (s, 1H), 4.60 (s, 1H), 4.09 (dd, J_1 = 12.8 Hz, J_2 = 7.6 Hz, 1H), 3.64 (t, J = 11.6 Hz, 1H), 2.95 (dd, J_1 = 10.8 Hz, J_2 = 8.0 Hz, 1H), 2.53 (s, 3H), 2.40 (s, 3H), 2.27 (s, 3H), 1.42 (s, 3H) ppm; ^{13}C NMR (100 MHz, $CDCl_3$) δ 144.1, 142.9, 139.9, 139.2, 136.6, 135.1, 131.9, 131.5, 129.6, 128.0, 122.6, 115.3, 57.2, 49.4, 21.8, 21.3, 19.9, 18.6 ppm; IR (thin film) 3446, 2929, 1638, 1458, 1383, 1355, 1165, 1088, 1014, 906, 813, 712, 678, 585, 536 (cm^{-1}); HRMS (ESI) m/z: calcd for $C_{20}H_{23}NNaO_2S$ [M + Na]$^+$, 364.1342; found 364.1339.

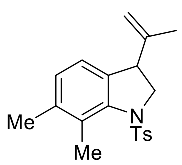

15c

Compound 15c: The synthesis of compound **15c** was followed by Procedure F. 32.1 mg (47%) of compound **15c** was isolated as a colorless oil. ^{1}H NMR (400 MHz, $CDCl_3$) δ 7.37 (d, J = 8.0 Hz, 2H), 7.16 (d, J = 8.0 Hz, 2H), 6.97 (d, J = 7.6 Hz, 1H), 6.62 (d, J = 7.6 Hz, 1H), 4.80 (s, 1H), 4.60 (s, 1H), 4.09 (dd, J_1 = 13.2 Hz, J_2 = 8.0 Hz, 1H), 3.62 (dd, J_1 = 12.4 Hz, J_2 = 11.2 Hz, 1H), 2.91 (dd, J_1 = 10.4 Hz, J_2 = 8.4 Hz,

1H), 2.46 (s, 3H), 2.40 (s, 3H), 2.31 (s, 3H), 1.41 (s, 3H) ppm; ^{13}C NMR (100 MHz, $CDCl_3$) δ 144.2, 142.9, 142.7, 137.7, 136.6, 134.9, 131.4, 129.6, 128.4, 128.0, 121.4, 115.1, 57.3, 49.4, 21.8, 20.3, 18.7, 17.5 ppm; IR (thin film) 3442, 2920, 1642, 1451, 1382, 1355, 1165, 1088, 1007, 898, 814, 676, 588 (cm^{-1}); HRMS (ESI) m/z: calcd for $C_{20}H_{23}NNaO_2S$ [M + Na]$^+$, 364.1342; found 364.1347.

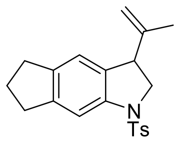

15d

Compound 15d: The synthesis of compound **15d** was followed by Procedure F. 56.5 mg (80%) of compound **15d** was isolated as a colorless oil. ^{1}H NMR (400 MHz, $CDCl_3$) δ 7.41 (d, J = 8.4 Hz, 2H), 6.99 (s, 1H), 6.96 (d, J = 8.0 Hz, 2H), 6.53 (s, 1H), 4.48 (m, 1H), 4.44 (s, 1H), 3.75 (t, J = 9.6 Hz, 1H), 3.50 (t, J = 7.2 Hz, 1H), 3.41 (dd, J_1 = 10.4 Hz, J_2 = 6.8 Hz, 1H), 2.63 (t, J = 7.2 Hz, 2H), 2.52 (t, J = 7.2 Hz, 2H), 2.1 (s, 3H), 1.84-1.75 (m, 2H), 1.13 (s, 3H) ppm; ^{13}C NMR (100 MHz, $CDCl_3$) δ 144.9, 144.5, 144.1, 140.9, 140.0, 134.2, 131.6, 129.8, 127.6, 121.0, 113.7, 111.1, 54.9, 48.0, 33.2, 32.4, 26.0, 21.7, 18.5 (cm^{-1}); IR (thin film) 3453, 2937, 2848, 1638, 1586, 1467, 1351, 1166, 1090, 1044, 952, 894, 871, 828, 799, 664, 593, 546 (cm^{-1}); HRMS (ESI) m/z: calcd for $C_{21}H_{23}NNaO_2S$ [M + Na]$^+$, 376.1342; found 376.1338.

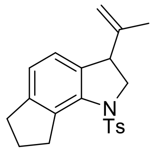

15e

Compound 15e: The synthesis of compound **15e** was followed by Procedure F. 47.3 mg (67%) of compound **15e** was isolated as a colorless oil. ^{1}H NMR (400 MHz, $CDCl_3$) δ 7.46 (d, J = 8.4 Hz, 2H), 7.17 (d, J = 8.0 Hz, 2H), 6.99 (d, J = 7.2 Hz, 1H), 6.69 (d, J = 7.6 Hz, 1H), 4.78 (s, 1H), 4.60 (s, 1H), 4.14 (dd, J_1 = 12.4 Hz, J_2 = 8.4 Hz, 1H), 3.75 (dd, J_1 = 12.8 Hz, J_2 = 10.4 Hz, 1H), 3.84-3.30 (m, 1H), 3.22 (t, J = 9.2 Hz, 1H), 3.13-3.06 (ddd, J_1 = 16.8 Hz, J_2 = 8.4 Hz, J_3 = 4.0 Hz, 1H), 2.99-2.83 (m, 2H), 2.38 (s, 3H), 2.18-2.09 (m, 1H), 2.02-1.94 (m, 1H), 1.47 (s, 3H) ppm; ^{13}C NMR (100 MHz, $CDCl_3$) δ 146.6, 144.0, 143.3, 139.3, 135.9, 135.4, 135.2, 129.7, 127.6, 122.7, 121.9, 114.9, 56.7, 49.0, 33.4, 33.3, 26.4, 21.8, 18.8 (cm^{-1}); IR (thin film) 3435, 2943, 2839, 1638, 1589, 1436, 1357, 1286, 1165, 1091, 1022, 897, 814, 674, 594, 541 (cm^{-1}); HRMS (ESI) m/z: calcd for $C_{21}H_{23}NNaO_2S$ [M + Na]$^+$, 376.1342; found 376.1336.

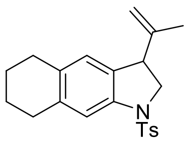

15f

Compound 15f: The synthesis of compound **15f** was followed by Procedure F. 47.0 mg (64%) of compound **15f** was isolated as a colorless oil. ^{1}H NMR (400 MHz, $CDCl_3$) δ 7.69 (dt, J_1 = 8.0 Hz, J_2 = 2.0 Hz, 2H), 7.36 (s, 1H), 7.23 (d, J = 8.0 Hz, 2H), 6.66 (s, 1H), 4.75 (m, 1H), 4.72 (m, 1H), 3.98 (dd, J_1 = 10.4 Hz, J_2 = 10.0 Hz, 1H), 3.77 (t, J = 7.2 Hz, 1H), 3.65 (dd, J_1 = 10.8 Hz, J_2 = 7.2 Hz, 1H), 2.79 (t, J = 4.8 Hz, 2H), 2.65 (d, J = 6.4 Hz, 2H), 2.37 (s, 3H), 1.78-1.75 (m, 4H), 1.40 (s, 3H) ppm; ^{13}C NMR (100 MHz, $CDCl_3$) δ 144.5, 144.1, 140.0, 137.5, 134.3, 132.7, 130.8, 129.8, 127.6, 125.7, 115.0, 113.7, 54.6, 48.1, 30.1, 29.4, 23.4, 23.4, 21.7, 18.6 ppm; IR (thin film) 3447, 2926, 1644, 1598, 1484, 1441, 1355, 1242, 1164, 1090, 1056, 900, 808, 664, 594, 544 (cm^{-1}); HRMS (ESI) m/z: calcd for $C_{22}H_{25}NNaO_2S$ [M + Na]$^+$, 390.1498; found 390.1496.

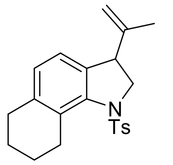

15g

Compound 15g: The synthesis of compound **15g** was followed by Procedure F. 44.8 mg (61%) of compound **15g** was isolated as a colorless oil. ^{1}H NMR (400 MHz, $CDCl_3$) δ 7.45 (d, J = 8.0 Hz, 2H), 7.18 (d, J = 8.0 Hz, 2H), 6.90 (d, J = 7.6 Hz, 1H), 6.64 (d, J = 7.6 Hz, 1H), 4.80 (s, 1H), 4.60 (s, 1H), 4.10 (dd, J_1 = 12.8 Hz, J_2 = 7.6 Hz, 1H), 3.62 (t, J = 11.6 Hz, 1H), 3.27-3.19 (m, 1H), 3.00-2.73 (m, 4H), 2.40 (s, 3H), 2.03-1.76 (m, 4H), 1.42 (s, 3H) ppm; ^{13}C NMR (100 MHz, $CDCl_3$) δ 144.2, 142.9, 142.0, 138.0, 136.1, 135.2, 132.8, 129.6, 128.0, 128.0, 121.4, 115.1, 57.6, 49.2, 29.6, 27.6, 23.0, 23.0, 21.8, 18.8 ppm; IR (thin film) 3435, 2929, 1644, 1595, 1442, 1357, 1247, 1165, 1090, 1046, 900, 813, 674, 576, 538 (cm^{-1}); HRMS (ESI) m/z: calcd for $C_{22}H_{25}NNaO_2S$ [M + Na]$^+$, 390.1498; found 390.1498.

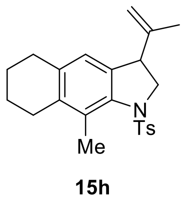

15h

Compound 15h: The synthesis of compound **15h** was followed by Procedure F. 42.7 mg (56%) of compound **15h** was isolated as a colorless oil. ^{1}H NMR (400 MHz, $CDCl_3$) δ 7.41 (d, J = 8.0 Hz, 2H), 7.18 (d, J = 8.0 Hz, 2H), 6.45 (s, 1H), 4.80 (s, 1H), 4.60 (s, 1H), 4.04 (dd, J_1 = 13.2 Hz, J_2 = 7.6 Hz, 1H), 3.59 (t, J = 11.6 Hz, 1H), 2.91 (dd, J_1 = 10.0 Hz, J_2 = 8.4 Hz, 1H), 2.75-2.57 (m, 4H), 2.41 (s, 3H), 2.40 (s, 3H), 1.91-1.68 (m, 4H), 1.42 (s, 3H) ppm; ^{13}C NMR (100 MHz, $CDCl_3$) δ 144.1, 143.0, 140.2, 136.1, 136.1, 135.7, 135.0, 130.9, 129.6, 128.1, 122.3, 115.0, 57.1, 49.3, 30.6, 27.3, 23.5, 23.0, 21.8, 18.7, 17.1 ppm; IR (thin film) 3445, 2926, 2854, 1639, 1458, 1382, 1355, 1260, 1216, 1164, 1090, 1026, 906, 865, 810 (cm^{-1}); HRMS (ESI) m/z: calcd for $C_{23}H_{27}NNaO_2S$ [M + Na]$^+$, 404.1655; found 404.1645.

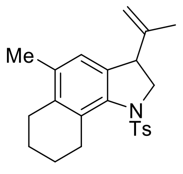

15i

Compound 15i: The synthesis of compound **15i** was followed by Procedure F. 38.1 mg (50%) of compound **15i** was isolated as a colorless oil. ^{1}H NMR (400 MHz, $CDCl_3$) δ 7.46 (dt, J_1 = 8.4 Hz, J_2 = 1.6 Hz, 2H), 7.18 (d, J = 8.0 Hz, 2H), 6.53 (s, 1H), 4.80 (m, 1H), 4.60 (m, 1H), 4.07 (dd, J_1 = 12.8 Hz, J_2 = 7.6 Hz, 1H), 3.60 (dd, J_1 = 12.8 Hz, J_2 = 11.2 Hz, 1H), 3.26-3.18 (m, 1H), 3.00-2.92 (m, 2H), 2.74-2.69 (m, 1H), 2.59-2.51 (m, 1H), 2.41 (s, 3H), 2.16 (s, 3H), 2.04-1.92 (m, 2H), 1.84-1.73 (m, 1H), 1.49-1.39 (m, 1H), 1.43 (s, 3H) ppm; ^{13}C NMR (100 MHz, $CDCl_3$) δ 144.0, 143.1, 140.1, 135.8, 135.6, 135.6, 135.3, 132.5, 129.6, 128.0, 122.9, 115.0, 57.6, 49.3, 28.3, 27.6, 23.3, 22.8, 21.8, 20.1, 18.8 ppm; IR (thin film) 3449, 2931, 1645, 1598, 1440, 1356, 1323, 1233, 1165, 1089, 1037, 968, 899, 872, 815, 677, 573, 541 (cm^{-1}); HRMS (ESI) m/z: calcd for $C_{23}H_{27}NNaO_2S$ $[M + Na]^+$, 404.1655; found 404.1655.

2.8. General procedure for the synthesis of 19b-19i (Procedure G): A suspension of cinnamyl amide **18** (57.4 mg, 0.2 mmol, 1.0 equiv), Cs_2CO_3 (520 mg, 1.6 mmol, 8.0 equiv), and 18-c-6 (26.4 mg, 0.4 mmol, 0.5 equiv) in anhydrous toluene (7 mL) under inert atmosphere was reflux for 30 minutes. A solution of **1** (0.4 mmol, 2.0 equiv) in MeCN (7 mL) was then added to this suspension over 4 hours via a syringe pump. After addition, it was heated for additional one hours. All the volatiles were removed directly on a rotary evaporator. Flash column chromatography with pet ether and EtOAc afforded product **19b-19i**.

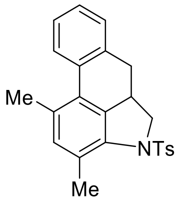

19b

Compound 19b: The synthesis of compound **19b** was followed by Procedure G. 54 mg (69%) of compound **19b** was isolated as a colorless oil; ^{1}H NMR (400 MHz, $CDCl_3$) δ 7.70 (d, J = 7.6 Hz, 1H), 7.52 (d, J = 8.4 Hz, 2H), 7.30-7.26 (m, 1H), 7.18-7.10 (m ,4H), 7.02 (s, 1H), 4.38 (dd, J_1 = 12.4 Hz, J_2 = 6.8 Hz, 1H), 3.60 (dd, J_1 = 12.4 Hz, J_2 = 10.4 Hz, 1H), 2.70 (dd, J_1 = 13.2 Hz, J_2 = 4.8 Hz, 1H), 2.57 (s, 6H), 2.47-2.39 (m, 1H), 2.35-2.28 (m, 4H) ppm; ^{13}C NMR (100 MHz, $CDCl_3$) δ 144.1, 140.6, 138.3, 137.3, 135.2, 135.0, 134.7, 132.8, 131.8, 129.8, 129.2, 128.9, 127.8, 127.4, 127.1, 126.8, 60.7, 36.9, 35.3, 22.2, 21.8, 19.7 ppm; IR (thin film) 3447, 2925, 1742, 1638, 1453, 1382, 1259, 1164, 1025, 926, 860, 808, 680 (cm^{-1}); HRMS (ESI) m/z: calcd for $C_{24}H_{23}NNaO_2S$ $[M + Na]^+$, 412.1342; found 412.1335.

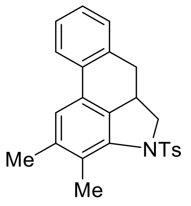

19c

Compound 19c: The synthesis of compound **19b** was followed by Procedure G. 49.0 mg (63%) of compound **19c** was isolated as a colorless oil; ^{1}H NMR (400 MHz, $CDCl_3$) δ 7.67 (d, J = 7.6 Hz, 1H), 7.50 (d, J = 8.4 Hz, 2H), 7.31 (s, 1H), 7.27 (t, J = 7.6 Hz, 1H), 7.18-7.10 (m, 4H), 4.41 (dd, J_1 = 12.8 Hz, J_2 = 6.8 Hz, 1H), 3.66 (dd, J_1 = 12.4 Hz, J_2 = 10.4 Hz, 1H), 2.76 (dd, J_1 = 13.2 Hz, J_2 = 4.8 Hz, 1H), 2.49-2.34 (m, 8H), 2.32 (s, 3H) ppm; ^{13}C NMR (100 MHz, $CDCl_3$) δ 144.2, 141.0, 138.9, 136.0, 135.7, 135.4, 133.8, 130.7, 129.8, 129.5, 129.2, 127.8, 127.7, 127.5, 123.2, 122.5, 61.9, 35.5, 34.6, 21.8, 20.9, 17.0 ppm; IR (thin film) 3418, 2932, 2373, 1636, 1384, 1349, 1164, 1088, 808, 758, 724, 669, 608, 567 (cm^{-1}); HRMS (ESI) m/z: calcd for $C_{24}H_{23}NNaO_2S$ [M + Na]$^+$, 412.1342; found 412.1348.

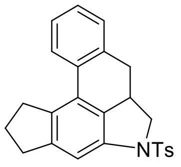

19d

Compound 19d: The synthesis of compound **19d** was followed by Procedure G. 66.6 mg (83%) of compound **19d** was isolated as a colorless oil; ^{1}H NMR (400 MHz, $CDCl_3$) δ 7.70 (d, J = 8.0 Hz, 3H), 7.42 (s, 1H), 7.31-7.26 (m, 1H), 7.22-7.18 (m, 4H), 4.37 (t, J = 10.4 Hz, 1H), 3.53 (t, J = 10.8 Hz, 1H), 3.24-2.88 (m, 6H), 2.55 (t, J = 14.4 Hz, 1H), 2.35 (s, 3H), 2.31-2.24 (m, 1H), 2.08-1.95 (m, 1H) ppm; ^{13}C NMR (100 MHz, $CDCl_3$) δ 147.0, 144.1, 139.3, 136.8, 135.6, 134.6, 134.3, 132.1, 129.9, 129.3, 127.7, 127.5, 127.3, 126.5, 111.6, 58.6, 35.7, 35.4, 33.3, 33.3, 26.4, 21.8 ppm; IR (thin film) 3439, 2937, 1650, 1425, 1352, 1265, 1164, 1096, 1039, 906, 796, 776, 736, 666, 600, 553 (cm^{-1}); HRMS (ESI) m/z: calcd for $C_{25}H_{23}NNaO_2S$ [M + Na], 424.1342; found 424.1336.

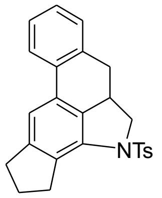

19e

Compound 19e: The synthesis of compound **19e** was followed by Procedure G. 61.0 mg (76%) of compound **19e** was isolated as a colorless oil; ^{1}H NMR (400 MHz, $CDCl_3$) δ 7.66 (d, J = 7.6 Hz, 1H), 7.54 (d, J = 8.4 Hz, 2H), 7.33 (s, 1H), 7.29-7.26 (m, 1H), 7.18-7.12 (m, 4H), 4.44 (dd, J_1 = 12.4 Hz, J_2 = 7.6 Hz, 1H), 3.71 (d, J = 11.6 Hz,

1H), 3.44-3.35 (m, 1H), 3.14-2.8 (m, 5H), 2.73-2.63 (m, 1H), 2.47 (d, J = 14.0 Hz, 1H), 2.32 (s, 3H), 2.26-2.19 (m, 1H), 2.11-1.97 (m, 1H) ppm; ^{13}C NMR (100 MHz, $CDCl_3$) δ 147.8, 144.1, 137.5, 135.7, 135.6, 135.5, 134.8, 134.1, 130.6, 129.9, 129.2, 127.7, 127.6, 127.4, 123.4, 116.2, 60.8, 35.6, 34.6, 33.7, 32.7, 26.3, 21.8 ppm; IR (thin film) 3451, 2930, 1597, 1456, 1353, 1165, 1089, 1010, 978, 816, 772, 727, 674, 580, 552 (cm^{-1}); HRMS (ESI) m/z: calcd for $C_{25}H_{23}NNaO_2S$ [M + Na]$^+$, 424.1342; found 424.1341.

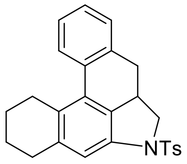

19f

Compound 19f: The synthesis of compound **19f** was followed by Procedure G. 68 mg (82%) of compound **19f** was isolated as a colorless oil; ^{1}H NMR (400 MHz, $CDCl_3$) δ 7.75 (d, J = 7.6 Hz, 1H), 7.70 (d, J = 8.4 Hz, 2H), 7.30 (s, 1H), 7.28-7.25 (m, 1H), 7.23-7.16 (m, 4H), 4.32 (dd, J_1 = 10.8 Hz, J_2 = 8.8 Hz, 1H), 3.48 (t, J = 10.4 Hz, 1H), 3.13-2.83 (m, 6H), 2.50 (t, J = 14.0 Hz, 1H), 2.35 (s, 3H), 2.07-1.96 (m, 2H), 1.78-1.67 (m, 1H), 1.49-1.39 (m, 1H) ppm; ^{13}C NMR (100 MHz, $CDCl_3$) δ 144.1, 138.8, 138.2, 138.0, 134.6, 134.4, 132.8, 131.4, 130.2, 129.9, 129.2, 128.2, 127.5, 127.4, 126.7, 116.0, 57.6, 36.4, 35.9, 30.9, 29.9, 23.8, 22.9, 21.7 ppm; IR (thin film) 3448, 2924, 1786, 1599, 1439, 1351, 1260, 1163, 1095, 1029, 873, 813, 764, 738, 668, 599, 536, 498 (cm^{-1}); HRMS (ESI) m/z: calcd for $[C_{26}H_{25}NNaO_2S]^+$, 438.1498; found 438.1501.

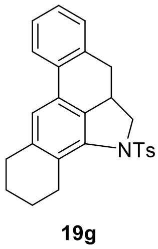

19g

Compound 19g: The synthesis of compound **19g** was followed by Procedure G. 51 mg (62%) of compound **19g** was isolated as a colorless oil; ^{1}H NMR (400 MHz, $CDCl_3$) δ 7.65 (d, J = 7.2 Hz, 1H), 7.54 (d, J = 8.0 Hz, 2H), 7.27 (t, J = 7.2 Hz, 1H), 7.23 (s, 1H), 7.16 (dt, J_1 = 7.6 Hz, J_2 = 1.2 Hz, 1H), 7.14-7.09 (m, 3H), 4.43 (dd, J_1 = 12.8 Hz, J_2 = 6.8 Hz, 1H), 3.65 (dd, J_1 = 12.4 Hz, J_2 = 10.8 Hz, 1H), 3.27-3.19 (m, 1H), 3.00-2.95 (m, 2H), 2.90-2.82 (m, 1H), 2.76 (dd, J_1 = 14.0 Hz, J_2 = 5.6 Hz, 1H), 2.51-2.44 (m, 1H), 2.39 (d, J = 14.0 Hz, 1H), 2.32 (s, 3H), 2.05-1.94 (m, 2H), 1.85-1.74 (m, 1H), 1.55-1.44 (m, 1H) ppm; ^{13}C NMR (100 MHz, $CDCl_3$) δ 144.2, 140.4, 139.2, 135.7, 135.5, 135.4, 133.9, 132.2, 129.8, 129.7, 129.2, 127.7, 127.7, 127.5, 123.3, 122.2, 62.1, 35.2, 34.5, 30.2, 27.6, 23.2, 23.0, 21.9 ppm; IR (thin film) 3447, 2836, 1616, 1507, 1420, 1311, 1245, 1213, 1171, 1143, 1109, 1035, 878, 621

(cm^{-1}); HRMS (ESI) m/z: calcd for $C_{26}H_{25}NNaO_2S$ [M + Na]$^+$, 438.1498; found 438.1497.

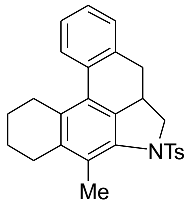

19h

Compound 19h: The synthesis of compound **19h** was followed by Procedure G. 72.1 mg (84%) of compound **19h** was isolated as a colorless oil; ^{1}H NMR (400 MHz, $CDCl_3$) δ 7.70 (d, *J* = 7.6 Hz, 1H), 7.51 (d, *J* = 8.0 Hz, 2H), 7.28-7.24 (m, 1H), 7.16-7.10 (m, 4H), 4.30 (dd, J_1 = 12.4 Hz, J_2 = 6.4 Hz, 1H), 3.55 (dd, J_1 = 12.4 Hz, J_2 = 10.0 Hz, 1H), 3.11-2.99 (m, 2H), 2.85 (dd, J_1 = 17.6 Hz, J_2 = 5.2 Hz, 1H), 2.71-2.64 (m, 2H), 2.45 (s, 3H), 2.34-2.24 (m, 5H), 2.17-2.05 (m, 2H), 1.78-1.68 (m, 1H), 1.45-1.39 (m, 1H) ppm; ^{13}C NMR (100 MHz, $CDCl_3$) δ 144.1, 138.4, 138.1, 138.0, 137.7, 135.2, 134.9, 133.0, 131.5, 129.7, 129.2, 128.8, 128.3, 128.0, 126.9, 126.4, 60.4, 37.3, 35.8, 30.9, 29.0, 23.6, 23.2, 21.8, 17.0 ppm; IR (thin film) 3446, 2926, 1636, 1433, 1383, 1351, 1218, 1164, 1092, 1010, 778, 724, 674, 637, 614, 553 (cm^{-1}); HRMS (ESI) m/z: calcd for $C_{27}H_{27}NNaO_2S$ [M + Na]$^+$, 452.1655; found 452.1662.

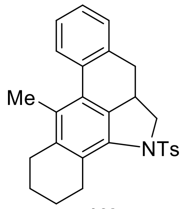

19i

Compound 19i: The synthesis of compound **19i** was followed by Procedure G. 58.3 mg (68%) of compound **19i** was isolated as a colorless oil; ^{1}H NMR (400 MHz, $CDCl_3$) δ 7.64 (d, *J* = 7.6 Hz, 1H), 7.56 (d, *J* = 8.0 Hz, 2H), 7.29-7.26 (m, 1H), 7.17-7.11 (m, 4H), 4.36 (dd, J_1 = 12.4 Hz, J_2 = 6.8 Hz, 1H), 3.54 (t, *J* = 11.6 Hz, 1H), 3.28-3.19 (m, 1H), 3.04 (d, *J* = 17.6 Hz, 1H), 2.82 (dd, J_1 = 17.2 Hz, J_2 = 4.4 Hz, 1H), 2.72-2.65 (m, 2H), 2.46-2.34 (m, 4H), 2.43 (s, 3H), 2.31 (s, 3H), 2.27 (t, *J* = 13.2 Hz, 1H), 2.14-2.04 (m, 2H), 1.81-1.70 (m, 1H), 1.50-1.42 (m, 1H) ppm; ^{13}C NMR (100 MHz, $CDCl_3$) δ 144.0, 138.2, 138.0, 137.9, 137.8, 135.5, 135.1, 132.5, 131.9, 129.9, 129.7, 128.8, 128.2, 127.9, 126.9, 126.5, 61.0, 37.4, 35.6, 29.5, 28.6, 23.7, 22.7, 21.8, 17.4 ppm; IR (thin film) 3439, 2935, 2854, 1634, 1454, 1352, 1164, 1089, 1027, 981, 808, 724, 676, 608, 553 (cm^{-1}); HRMS (ESI) m/z: calcd for $C_{27}H_{27}NNaO_2S$ [M + Na]$^+$, 452.1655; found 452.1660.

2.9. General procedure for the synthesis of 20 (Procedure H): A suspension of *N*-benzoylbenzamide (90.0 mg, 0.4 mmol, 1.0 equiv), Cs_2CO_3 (520 mg, 1.6 mmol, 4.0 equiv), H_2O (1.4 mg, 0.08 mmol, 0.2 equiv), and 18-c-6 (52.8 mg, 0.2 mmol, 0.5 equiv) in anhydrous MeCN (5 mL) under inert atmosphere at 80 °C was stirred for 30

minutes. A solution of **1** (0.8 mmol, 2.0 equiv) in MeCN (5 mL) was then added to this suspension over eight hours via a syringe pump. After addition, it was heated for additional four hours. The resulting mixture was concentrated directly on rotary evaporator. Flash column chromatography with pet ether and EtOAc afforded product **20**.

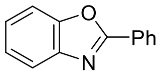

20a

Compound 20a: The synthesis of compound **20a** was followed by Procedure H from **1b**. 53.0 mg (68%) of compound **20a** was isolated as a colorless oil, the ^{1}H NMR of which is identical with that reported in literature.[35] ^{1}H NMR (400 MHz, $CDCl_3$) δ 8.20 (dd, J_1 = 5.6 Hz, J_2 = 2.2 Hz, 2H), 7.69 (m, 1H), 7.43-7.57 (m, 4H), 7.25-7.33 (m, 2H) ppm.

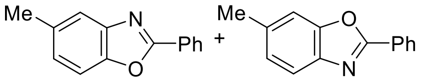

20b **20b'**

Compound 20b and 20b': The synthesis of compound **20b** and **20b'** was followed by Procedure H from **1e**. 41.0 mg (49%) of **20b** and **20b'** was isolated as a 1:3 mixture of regioisomer, the ^{1}H NMR of both compounds are identical with that reported in literature.[35] ^{1}H NMR (400 MHz, $CDCl_3$) δ 8.26-8.23 (m, 8H), 7.64 (d, J= 8.0 Hz, 1H), 7.56-7.51 (m, 17H), 7.46 (d, J= 8.0 Hz, 3H), 7.39 (s, 1H), 7.16 (m, 4H), 2.51 (s, 3H) (minor), 2.49 (s, 9H) (major) ppm.

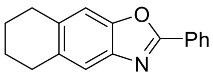

20c

Compound 20c: The synthesis of compound **20c** was followed by Procedure H from **1w**. 60.8 mg (61%) of compound **20c** was isolated as a colorless oil. ^{1}H NMR (400 MHz, $CDCl_3$) δ 8.23 (m, 2H), 7.51 (m, 3H), 7.44 (s, 1H), 7.26 (s, 1H), 2.91 (m, 4H), 1.84 (m, 4H) ppm; ^{13}C NMR (100 MHz, $CDCl_3$) δ 162.8, 149.5, 140.4, 135.2, 134.1, 131.4, 129.1, 127.7, 119.6, 110.2, 30.4, 30.0, 23.4, 23.3 ppm; IR (thin film) 3854, 3821, 3752, 3650, 3432, 2927, 2355, 1685, 1560, 1461, 1405, 1358, 1136, 1092, 1004, 835, 820, 723, 705, 545 (cm^{-1}); HRMS (ESI) m/z: Calcd for $C_{17}H_{16}NO$ $[M + H]^+$, 250.1226; Found, 250.1232.

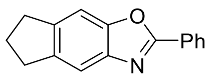

20d

Compound 20d: The synthesis of compound **20d** was followed by Procedure H from **1u**. 49.8 mg (53%) of compound **20d** was isolated as a colorless oil. ^{1}H NMR (400 MHz, $CDCl_3$) δ 8.23 (m, 2H), 7.56 (s, 1H), 7.51 (m, 3H), 7.40 (s, 1H), 3.01 (m, 4H), 2.17 (m, 2H) ppm; ^{13}C NMR (100 MHz, $CDCl_3$) δ 162.8, 150.3, 142.4, 141.2, 131.3,

129.1, 127.8, 127.6, 115.2, 106.4, 33.3, 32.9, 26.7 ppm; IR (thin film) 3432, 2961, 2922, 2843, 2341, 1629, 1401, 1384, 1095, 998, 859, 778, 684, 586, 543, 475 (cm^{-1}); HRMS (ESI) m/z: Calcd for $C_{16}H_{13}NNaO$ $[M + Na]^+$, 258.0889; Found, 258.0898.

2.10. Study on 3,4-pyridyne precursor:

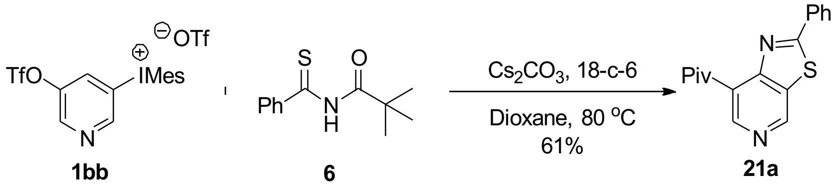

To a mixture of benzothioamide **6** (44.2 mg, 0.2 mmol, 1.0 equiv), Cs_2CO_3 (520 mg, 1.6 mmol), and 18-c-6 (105.6 mg, 0.4 mmol) in 1,4-dioxane (3 mL) at 80 °C was added a solution of **1bb** (268 mg, 0.4 mmol, 2.0 equiv) in 1,4-dioxane (3 mL) over 8 hours via a syringe pump. All the volatiles were removed directly on a rotary evaporator. Flash column chromatography on silica gel afforded 36.1 mg (61%) of **21a** as a colorless oil. ^1H NMR (400 MHz, $CDCl_3$) δ 9.22 (s, 1H), 8.49 (s, 1H), 8.13-8.11 (m, 2H), 7.58-7.51 (m, 3H), 1.38 (s, 9H) ppm; ^{13}C NMR (100 MHz, $CDCl_3$) δ 210.8, 173.8, 155.4, 148.2, 144.8, 142.7, 132.8, 132.7, 129.5, 128.4, 45.5, 29.9, 27.1 ppm; IR (thin film) 3473, 3418, 2917, 2364, 1636, 1385, 1218, 1126, 712, 628 (cm^{-1}); HRMS (ESI) m/z: calcd for $C_{17}H_{17}N_2O_2S$ $[M + H]^+$, 297.1056; found 297.1056.

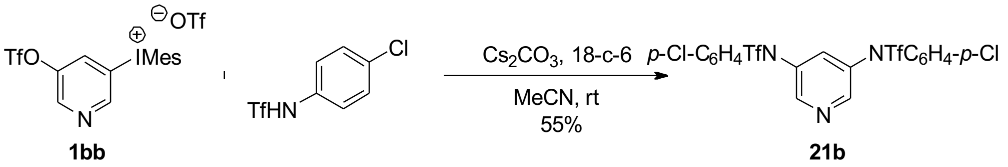

To a mixture of *N*-triflated *p*-chloroaniline (95.6 mg, 0.4 mmol, 1.0 equiv), Cs_2CO_3 (520 mg, 1.6 mmol, 4.0 equiv), and 18-c-6 (105.6 mg, 0.4 mmol, 1.0 equiv) in MeCN (10 mL) at room temperature was added a solution of **1bb** (268 mg, 0.4 mmol, 2.0 equiv) in MeCN (10 mL) over 8 hours via a syringe pump. All the volatiles were removed directly on a rotary evaporator. Flash column chromatography on silica gel afforded 65.2 mg (55%) of **21b** as a yellow oil. ^1H NMR (400 MHz, $CDCl_3$) δ 8.57 (d, J = 1.6 Hz, 2H), 7.90 (t, J = 2.4 Hz, 1H), 7.45 (d, J = 8.8 Hz, 4H), 7.36 (d, J = 8.8 Hz, 4H) ppm; ^{13}C NMR (100 MHz, $CDCl_3$) δ 147.9, 137.0, 136.8, 136.5, 133.3, 130.8, 130.0, 120.2 (q, J = 321.7 Hz) ppm; IR (thin film) 3419, 2929, 2851, 2364, 2344, 1636, 1404, 1385, 1224, 1201, 1123, 712, 611 (cm^{-1}); HRMS (ESI) m/z: calcd for $C_{19}H_{12}Cl_2F_6N_3O_4S_2$ $[M + H]^+$, 593.9545; found 593.9538.

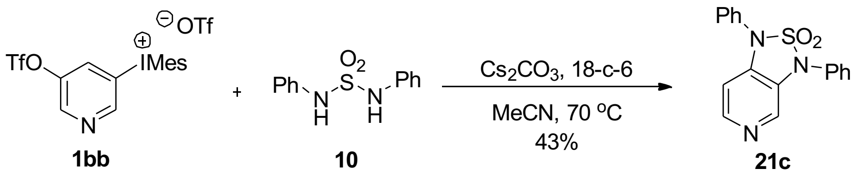

To a mixture of sulfonamide **10** (49.6 mg, 0.2 mmol, 1.0 equiv), Cs_2CO_3 (520 mg, 1.6 mmol), and 18-c-6 (105.6 mg, 0.4 mmol, 1.0 equiv) in MeCN (10 mL) at 70 ℃ was added a solution of **1bb** (268 mg, 0.4 mmol, 2.0 equiv) in MeCN (10 mL) over 8 hours via a syringe pump. All the volatiles were removed directly on a rotary evaporator. Flash column chromatography on silica gel afforded 27.8 mg (43%) of **21c** as a yellow oil. ^1H NMR (400 MHz, $CDCl_3$) δ 8.25 (m 1H), 7.96 (m, 1H), 7.63 (m ,10H), 6.80 (m, 1H) ppm; ^{13}C NMR (100 MHz, $CDCl_3$) δ 144.5, 136.4, 131.8, 131.1, 130.8, 130.6, 130.3, 129.9, 128.5, 128.2, 128.2, 120.0, 103.7 ppm; IR (thin film) 3424, 2928, 2851, 2364, 1728, 1633, 1386, 1161, 1120, 781, 709, 617 (cm^{-1}); HRMS (ESI) m/z: calcd for $C_{17}H_{14}N_3O_2S$ $[M + H]^+$, 324.0801; found 324.0804.

2.11. Study on [2+2]-cycloaddition, Grob-fragmentation approach:

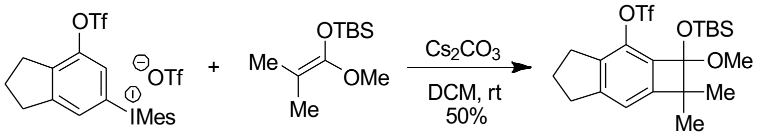

1u **23a**

Preparation of compound 23a: To a suspension of ketene silyl acetal (0.8 mmol, 2.0 equiv) and Cs_2CO_3 (520 mg, 1.6 mmol, 8.0 equiv) in anhydrous DCM (10 mL) under inert atmosphere at room temperature was added a solution of **1u** (264 mg, 0.4 mmol, 1.0 equiv) in DCM (10 mL) over 4 hours via a syringe pump. After addition, it was stirred for one additional hour. All the volatiles were removed directly on a rotary evaporator. Flash column chromatography with pet ether and EtOAc afforded 96 mg (50%) of compound **23a** as a colorless oil. ^1H NMR (400 MHz, $CDCl_3$) δ 7.03 (s, 1H), 3.47 (s, 3H), 2.96 (q, J = 7.2 Hz, 4H), 2.17-2.03 (m, 2H), 1.34 (s, 3H), 1.32 (s, 3H), 0.92 (s, 9H), 0.24 (s, 3H), 0.18 (s, 3H) ppm; ^{13}C NMR (100 MHz, $CDCl_3$) δ 154.1, 150.9, 137.8, 135.0, 133.7, 118.8 (q, J = 317.8 Hz), 117.6, 105.9, 56.9, 55.1, 33.9, 30.3, 26.5, 25.5, 25.1, 22.5, 18.6, -2.9, -2.9 ppm; IR (thin film) 3417, 2961, 2862, 2367, 1620, 1467, 1419, 1381, 1326, 1213, 1143, 1095, 989, 932, 898, 841, 773, 680, 602 (cm^{-1}); HRMS (ESI) m/z: calcd for $C_{20}H_{31}F_3NaO_5SSi$ $[M + Na]^+$, 503.1506; found 503.1500.

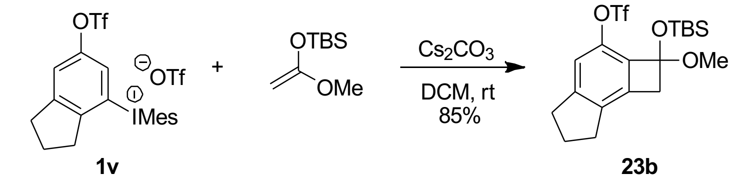

1v **23b**

Preparation of compound 23b: To a suspension of ketene silyl acetal (0.8 mmol, 2.0 equiv) and Cs_2CO_3 (520 mg, 1.6 mmol, 8.0 equiv) in anhydrous DCM (10 mL) under inert atmosphere at room temperature was added a solution of **1v** (264 mg, 0.4 mmol, 1.0 equiv) in DCM (10 mL) over 4 hours via a syringe pump. After addition, it was stirred for one additional hour. All the volatiles were removed directly on a rotary evaporator. Flash column chromatography with pet ether and EtOAc afforded 154 mg

(85%) of compound **23b** as a colorless oil. ^{1}H NMR (400 MHz, $CDCl_3$) δ 6.98 (s, 1H), 3.48 (s, 3H), 3.43 (d, J = 14.0 Hz, 1H), 3.38 (d, J = 14.0 Hz, 1H), 2.91 (td, J_1 = 7.6 Hz, J_2 = 2.8 Hz, 2H), 2.79 (t, J = 7.6 Hz, 2H), 2.18-2.10 (m, 2H), 0.92 (s, 9H), 0.20 (s, 3H), 0.18 (s, 3H) ppm; ^{13}C NMR (100 MHz, $CDCl_3$) δ 149.8, 140.4, 139.2, 138.8, 136.5, 118.8 (q, J = 318.4 Hz), 115.8, 102.9, 53.4, 48.3, 33.1, 29.7, 26.0, 25.8, 18.1, -3.2, -3.6 ppm; IR (thin film) 3418, 2935, 2854, 1621, 1464, 1425, 1349, 1213, 1146, 1068, 1007, 929, 839, 784, 614 (cm^{-1}); HRMS (ESI) m/z: calcd for $C_{19}H_{27}F_3NaO_5SSi$ $[M + Na]^+$, 475.1193; found 475.1200.

Synthesis of 24a-24d:

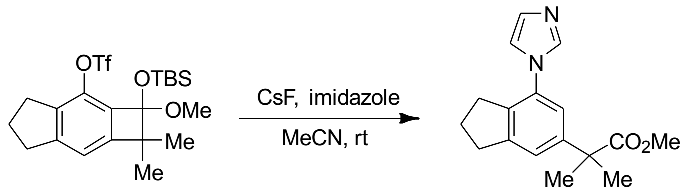

23a 24a

Compound 24a: To a suspension of imidazole (54.4 mg, 0.8 mmol, 2.0 equiv) and CsF (520 mg, 1.6 mmol, 8.0 equiv) in anhydrous MeCN (10 mL) under inert atmosphere at room temperature was added a solution of **23a** (192 mg, 0.4 mmol, 1.0 equiv) in MeCN (10 mL). After stirred for 16 hours, all the volatiles were removed directly on a rotary evaporator. Flash column chromatography with pet ether and EtOAc afforded 64.8 mg (57%) of **24a** as colorless oil. ^{1}H NMR (400 MHz, $CDCl_3$) δ 7.80 (s, 1H),7.26 (s, 1H), 7.20 (d, J = 18.4 Hz, 2H), 7.06 (s, 1H), 3.68 (s, 3H), 3.00 (t, J = 7.2 Hz, 2H), 2.87 (t, J = 7.6 Hz, 2H), 2.17-2.06 (m, 2H), 1.59 (s, 5H), ppm; ^{13}C NMR (100 MHz, $CDCl_3$) δ 177.1, 147.6, 145.1, 137.0, 136.7, 134.0, 129.7, 121.8, 119.7, 119.5, 52.6, 46.6, 33.6, 31.2, 26.9, 25.7 ppm; ^{13}C NMR (100 MHz, $CDCl_3$) δ 177.1, 147.6, 145.1, 137.0, 136.7, 134.0, 129.7, 121.8, 119.7, 119.5, 52.6, 46.6, 33.6, 31.2, 26.9, 25.7 ppm; IR (thin film) 3422, 2914, 2854, 2364, 1734, 1622, 1470, 1383, 1262, 1126, 868, 666, 628 (cm^{-1}); HRMS (ESI) m/z: calcd for $C_{17}H_{21}N_2O_2$ $[M + H]^+$, 285.1598; found 285.1592.

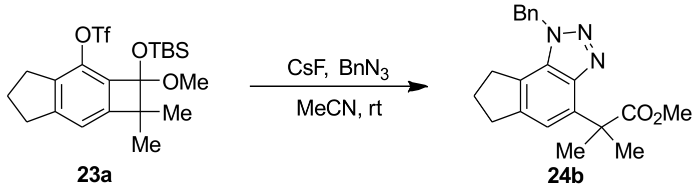

23a 24b

Compound 24b: To a suspension of BnN_3 (106.4 mg, 0.8 mmol, 2.0 equiv) and CsF (520 mg, 1.6 mmol, 8.0 equiv) in anhydrous MeCN (10 mL) under inert atmosphere at room temperature was added a solution of **23a** (192 mg, 0.4 mmol, 1.0 equiv) in MeCN (10 mL). After stirred for 16 hours, all the volatiles were removed directly on a rotary evaporator. Flash column chromatography with pet ether and EtOAc afforded

87.9 mg (63%) of **24b** as colorless oil. ^{1}H NMR (400 MHz, $CDCl_3$) δ 7.33-7.27 (m, 3H), 7.17-7.15 (m, 2H), 7.11 (s, 1H), 5.87 (s, 2H), 3.70 (s, 3H), 3.00 (t, J = 7.2 Hz, 4H), 2.18-2.11 (m, 2H), 1.80 (s, 6H) ppm; ^{13}C NMR (100 MHz, $CDCl_3$) δ 177.7, 144.5, 144.4, 136.7, 135.9, 131.3, 129.1, 128.3, 127.0, 123.2, 117.2, 52.6, 52.5, 46.0, 33.4, 30.8, 26.7, 25.2 ppm; IR (thin film) 3452, 2925, 2851, 1741, 1635, 1438, 1382, 1250, 1136, 1027, 865, 828, 738 (cm^{-1}); HRMS (ESI) m/z: calcd for $C_{21}H_{23}N_3NaO_2$ $[M + Na]^+$, 372.1683; found 372.1684.

The structure of **24b** was confirmed by NOESY experiment, as the following interaction was observed:

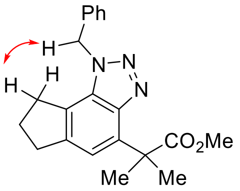

24b

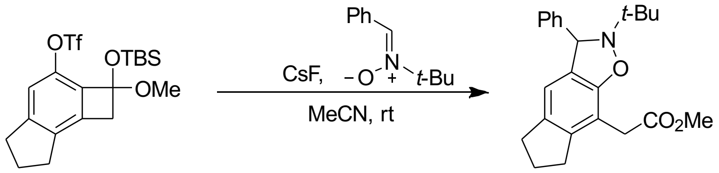

23b **24c**

Compound 24c: To a suspension of *N*-*t*-butyl-*a*-phenylnitrone (142 mg, 0.8 mmol, 10.0 equiv) and CsF (520 mg, 1.6 mmol, 8.0 equiv) in anhydrous MeCN (10 mL) under inert atmosphere at room temperature was added a solution of **23b** (181 mg, 0.4 mmol, 1.0 equiv) in MeCN (10 mL). After stirred for 16 hours, all the volatiles were removed directly on a rotary evaporator. Flash column chromatography with pet ether and EtOAc afforded 138.7 mg (95%) of **24c** as colorless oil. ^{1}H NMR (400 MHz, $CDCl_3$) δ 7.39 (d, J = 7.2 Hz, 1.65H), 7.33-7.27 (m, 3.3H), 7.25-7.23 (m, 3.3H), 6.67 (s, 1H), 6.65 (s, 0.65H), 5.64 (s, 0.65H), 5.55 (s, 1H), 3.72-3.68 (m, 4H), 3.58-3.53 (m, 2.95H), 3.29 (d, J = 15.2 Hz, 0.65H), 3.14 (d, J = 15.2 Hz, 0.65H), 2.89 (t, J = 7.6 Hz, 1.3H), 2.81-2.69 (m, 5.3H), 2.11-2.04 (m, 3.3H) 1.18 (s, 5.9H), 1.17 (s, 9H) ppm; ^{13}C NMR (100 MHz, $CDCl_3$) δ 171.6, 171.2, 157.2, 154.1, 146.1, 144.5, 144.4, 143.2, 137.1, 136.5, 128.9, 128.8, 128.3, 128.2, 127.7, 127.5, 127.4, 127.1, 125.2, 118.2, 109.8, 102.2, 67.6, 66.6, 61.7, 61.4, 52.2, 52.1, 35.7, 33.5, 33.3, 32.6, 31.2, 30.5, 25.8, 25.6, 25.5, 25.5 ppm; IR (thin film) 3422, 2923, 2842, 2367, 1738, 1633, 1450, 1385, 1262, 1210, 1160, 845, 758, 712, 617, 556 (cm^{-1}); HRMS (ESI) m/z: calcd for $C_{23}H_{27}NNaO_3$ $[M + Na]^+$, 388.1883; found 388.1885.

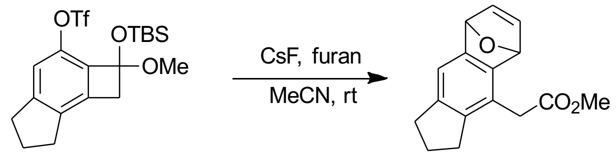

23b 24d

Compound 24d: To a suspension of furan (680 mg, 4.0 mmol, 10.0 equiv) and CsF (520 mg, 1.6 mmol, 8.0 equiv) in anhydrous MeCN (10 mL) under inert atmosphere at room temperature was added a solution of **23b** (181 mg, 0.4 mmol, 1.0 equiv) in MeCN (10 mL). After stirred for 16 hours, all the volatiles were removed directly on a rotary evaporator. Flash column chromatography with pet ether and EtOAc afforded 95.2 mg (93%) of **24d** as colorless oil. ^{1}H NMR (400 MHz, $CDCl_3$) δ 7.06 (dd, J_1 = 5.6 Hz, J_2 = 2.0 Hz, 1H), 7.05 (s, 1H), 7.01 (dd, J_1 = 5.6 Hz, J_2 = 2.0 Hz, 1H), 5.79 (s, 1H), 5.66 (s, 1H), 3.70-3.55 (m, 3H), 2.86-2.79 (m, 4H), 2.12-2.04 (m, 2H) ppm; ^{13}C NMR (100 MHz, $CDCl_3$) δ 171.6, 148.4, 147.5, 143.4, 143.1, 141.4, 140.3, 123.2, 116.4, 82.8, 81.5, 52.3, 36.3, 33.2, 31.1, 25.2 ppm; IR (thin film) 3420, 2961, 2859,2370, 1735, 1636, 1441, 1384, 1265, 1162, 1039, 848, 718, 611 (cm^{-1}); HRMS (ESI) m/z: calcd for $C_{16}H_{16}NaO_3$ [M + Na]$^+$, 279.0992; found 279.0998.

2.12. Study toward the synthesis of (±)-esermethole and (±)-physostigmine:

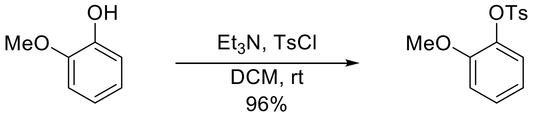

2-Methoxyphenyl 4-methylbenzenesulfonate: To a solution of commercially available 2-methoxyphenol (1.0 g, 8.06 mmol, 1.0 equiv) in anhydrous DCM (20 mL) at rt was added Et_3N (1.67 mL, 10.88 mmol, 1.5 equiv). After stirred for 5 minutes, TsCl (1.69 g, 8.87 mmol, 1.1 equiv) was added slowly. After 10 minutes, all the volatiles were directly removed on a rotary evaporator. Flash column chromatography on silica gel afforded 2.15 g (96%) of 2-methoxyphenyl 4-methylbenzenesulfonate as a colorless oil, the ^{1}H NMR of which is identical with that reported in literature.[36] ^{1}H NMR (400 MHz, $CDCl_3$) δ 7.77 (d, J = 8.3 Hz, 2H), 7.33 (d, J = 8.3 Hz, 2H), 7.23-7.18 (m, 1H), 7.16 (d, J = 8.2 Hz, 1H), 6.90 (m, 1H), 6.86 (m, 1H), 3.60 (s, 3H), 2.46 (s, 3H) ppm.

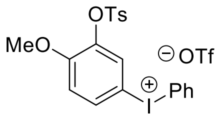

1cc-Ts

Aryne precursor 1cc-Ts: To a solution of 2-methoxyphenyl 4-methylbenzenesulfonate (153 mg, 0.55 mmol, 1.1 equiv) in anhydrous 2,2,2-trifluoroethyl alcohol (10 mL) at room temperature was added Koser's reagent **5** (196.0 mg, 0.5 mmol, 1.0 equiv). After eight hours, it was filtrated. All the volatiles

were then removed on a rotary evaporator. The resulting crude material was triturated with diethyl ether and isolated by filtration to afford a crude solid, which was used directly in the next step without further purification.

To the above crude solid in anhydrous MeCN (10 mL) at room temperature was added NaOTf (94.6 mg, 0.55 mmol, 1.1 equiv). After stirred for three hours, it was quenched with water and extracted with DCM (20 mL x 3). After concentration, the resulting crude material was triturated with diethyl ether and isolated by filtration to afford 230.0 mg (73%) of aryne precursor **1cc-Ts** as a white solid. Mp: 152-153 °C; ^{1}H NMR (400 MHz, DMSO-*d6*) δ 8.24-8.16 (m, 4H), 7.68 (t, *J* = 7.6 Hz, 1H), 7.62 (d, *J* = 8.4 Hz, 2H), 7.55 (t, *J* = 8.0 Hz, 2H), 7.42 (d, *J* = 8.4 Hz, 2H), 7.20 (d, *J* = 8.8 Hz, 1H) 3.52 (s, 3H), 2.42 (s, 3H) ppm; ^{13}C NMR (100 MHz, DMSO-*d6*) δ 154.5, 146.1, 138.1, 136.2, 134.9, 132.0, 131.7, 131.5, 130.5, 129.9, 128.2, 120.7 (q, *J* = 320.4 Hz), 117.4, 116.2, 104.1, 56.2, 21.2 ppm; IR (thin film) 3424, 2283, 1647, 1583, 1495, 1472, 1443, 1377, 1279, 1242, 1167, 1129, 1089, 1028, 990, 877, 814, 780, 713, 680, 637, 545, 453 (cm^{-1}); HRMS (ESI) m/z: Calcd for $C_{20}H_{18}IO_4S$ [M - OTf]$^+$, 480.9965; Found, 480.9970.

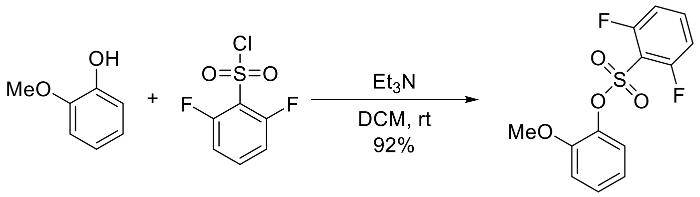

2-Methoxyphenyl 2,6-difluorobenzenesulfonate: To a solution of 2-methoxyphenol (1.0 g, 8.06 mmol, 1.0 equiv) in anhydrous DCM (20 mL) at room temperature was added Et_3N (1.59 mL, 12.09 mmol, 1.5 equiv). After stirred for five minutes, 2,6-difluorobenzenesulfonyl chloride (1.88 g, 8.87 mmol, 1.1 equiv) was added slowly. After 30 minutes, all the volatiles were directly removed on a rotary evaporator. Flash column chromatography on silica gel afforded 2.22 g (92%) of 2-methoxyphenyl 2,6-difluorobenzenesulfonate as a white solid. Mp: 67-68 °C; ^{1}H NMR (400 MHz, $CDCl_3$) δ 7.64-7.57 (m, 1H), 7.26-7.20 (m, 2H), 7.04 (t, *J* = 8.4 Hz, 2H), 6.93 (td, J_1 = 7.6 Hz, J_2 = 1.2 Hz, 1H), 6.87 (dd, J_1 = 8.4 Hz, J_2 = 1.2 Hz, 1H), 3.56 (s, 3H) ppm; ^{13}C NMR (100 MHz, $CDCl_3$) δ 160.4 (d, *J* = 258.6 Hz), 151.7, 138.4, 136.1 (t, *J* = 10.8 Hz), 128.7, 124.4, 121.1, 113.1, 113.1, 112.9, 55.7 ppm; IR (thin film) 3406, 3104, 3017, 2846, 2774, 2302, 1960, 1614, 1590, 1497, 1474, 1394, 1299, 1259, 1200, 1156, 1101, 1010, 869, 789, 761, 719, 707, 640 (cm^{-1}); HRMS (ESI) m/z: Calcd for $C_{13}H_{10}F_2NaO_4S$ [M + Na]$^+$, 323.0160; Found, 323.0169.

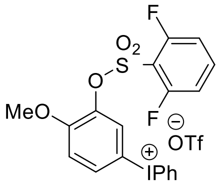

1cc-FPs

Aryne precursor 1cc-FPs: To a solution of 2-methoxyphenyl 2,6-difluorobenzenesulfonate (165 mg, 0.55 mmol, 1.1 equiv) in anhydrous 2,2,2-trifluoroethyl alcohol (10 mL) at room temperature was added Koser's reagent **5** (196.0 mg, 0.5 mmol, 1.0 equiv). After eight hours, it was filtrated. All the volatiles were then removed on a rotary evaporator. The resulting crude material was triturated with diethyl ether and isolated by filtration to afford a crude solid, which was used directly in the next step without further purification.

To the above crude solid in anhydrous MeCN (10 mL) at room temperature was added NaOTf (94.6 mg, 0.55 mmol, 1.1 equiv). After stirred for three hours, it was quenched with water and extracted with DCM (20 mL x 3). After concentration, the resulting crude material was triturated with diethyl ether and isolated by filtration to afford 228.2 mg (70%) of aryne precursor **1cc-FPs** as a white solid. Mp: 136-137 °C; ^{1}H NMR (400 MHz, DMSO-*d6*) δ 8.35 (d, *J* = 2.4 Hz, 1H), 8.25-8.19 (m, 3H), 7.95-7.91 (m, 1H), 7.66 (t, *J* = 7.2 Hz, 1H), 7.53 (t, *J* = 8.0 Hz, 2H), 7.40 (t, *J* = 8.8 Hz, 2H), 7.26 (d, *J* = 9.2 Hz, 1H), 3.52 (s, 3H) ppm; ^{13}C NMR (100 MHz, DMSO-*d6*) δ 159.1 (d, *J* = 256.3 Hz), 154.3, 138.4 (t, *J* = 11.1 Hz), 137.9, 136.7, 134.8, 132.0, 131.7, 131.0, 120.7 (q, *J* = 320.5 Hz), 117.3, 116.4, 113.8-113.6 (m), 113.3 (t, *J* = 15.4 Hz), 104.3, 56.3 ppm; IR (thin film) 3406, 3109, 2951, 2845, 2348, 1614, 1591, 1497, 1474, 1443, 1398, 1305, 1259, 1200, 1159, 1106, 1025, 1006, 990, 884, 873, 831, 789, 744, 718, 633 (cm^{-1}); HRMS (ESI) m/z: Calcd for $C_{19}H_{14}F_{2}IO_{4}S$ [M - OTf]$^{+}$, 502.9620; Found, 502.9617.

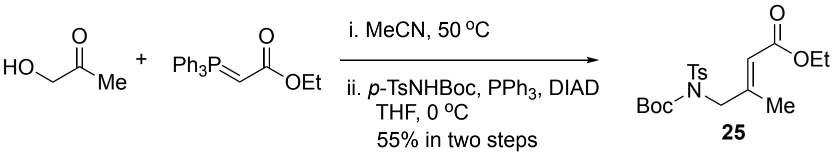

Compound 25: To a solution of 1-hydroxypropan-2-one (3.0 g, 40.54 mmol, 1.0 equiv) in anhydrous MeCN (50 mL) at 50 ℃ was added ethyl 2-(triphenyl-λ^{5}-phosphaneylidene)acetate (16.9 g, 48.65 mmol, 1.2 equiv). After two hours, all the volatiles were directly removed on a rotary evaporator. Petroleum ether (200 mL) and ethyl acetate (20 mL) was added to the resulting mixture. After stirred for two hours, the residue solid was filtered out. Water (200 mL) was added to the mother liquor and it was extracted with DCM (50 mL x 3). The combined organic layers were washed with brine (100 mL), dried over Na_2SO_4, filtered, and concentrated. A crude oil was obtained, which was used directly in the next step without further purification.

To the above crude oil in THF (100 mL) at 0 ℃ was added *tert*-butyl tosylcarbamate

(p-TsNHBoc) (12.1 g, 44.59 mmol, 1.1 equiv) and PPh_3 (21.2 g, 81.08 mmol, 2.0 equiv). After stirred for five minutes, DIAD (16.1 mL, 81.08 mmol, 2.0 equiv) was added slowly. After three hours, all the volatiles were directly removed on a rotary evaporator. Petroleum ether (200 mL) and ethyl acetate (20 mL) was added to the resulting mixture. After stirred for two hours, the residue solid was filtered out. Water (200 mL) was added to the mother liquor and it was extracted with DCM (50 mL x 3). Flash column chromatography (4:1) on silica gel afforded 8.85 g (55% in two steps) of compound **25** as a white solid. Mp: 74-75 °C; ^{1}H NMR (400 MHz, $CDCl_3$) δ 7.77 (d, J = 8.4 Hz, 2H), 7.28 (d, J = 8.4 Hz, 2H), 5.76 (s, 1H), 4.45 (s, 2H), 4.15-4.09 (m, 2H), 2.41 (s, 3H), 2.14 (s, 3H), 1.33 (d, J = 2.8 Hz, 9H), 1.26-1.22 (m, 3H) ppm; ^{13}C NMR (100 MHz, $CDCl_3$) δ 166.6, 153.3, 150.9, 144.7, 136.9, 129.5, 128.5, 116.0, 85.0, 60.0, 53.2, 28.0, 21.8, 16.5, 14.5 ppm; IR (thin film) 3421, 2983, 2934, 2346, 1723, 1663, 1397, 1368, 1220, 1148, 1091, 985, 859, 815, 767, 719, 677, 655, 586, 545, 469 (cm^{-1}); HRMS (ESI) m/z: Calcd for $C_{19}H_{27}NNaO_6S$ $[M + Na]^+$, 420.1451; Found, 420.1459.

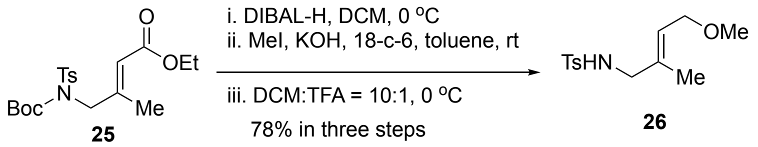

Compound 26: To a solution of compound **25** (2.0 g, 5.0 mmol, 1.0 equiv) in anhydrous DCM (30 mL) at 0 ℃ was slowly added DIBAL-H (1 M in hexanes, 10.5 mL, 10.5 mmol, 2.1 equiv). After three hours, water (20 mL) was added to the mother liquor and it was extracted with EtOAc (20 mL x 3). The combined organic layers were washed with brine (20 mL), dried over Na_2SO_4, filtered, and concentrated. A crude oil was obtained, which was used directly in the next step without further purification.

To the above crude oil in anhydrous toluene (20 mL) at room temperature was added KOH (560 mg, 10.0 mmol, 2.0 equiv) and 18-c-6 (661 mg, 2.5 mmol, 0.5 equiv). After stirred for five minutes, MeI (467 uL, 7.5 mmol, 1.5 equiv) was added slowly. After four hours, it was quenched with water and extracted with EtOAc (20 mL x 3). The combined organic layers were washed with brine (20 mL), dried over Na_2SO_4, filtered, and concentrated. A crude oil was obtained, which was used directly in the next step without further purification.

The above crude oil was treated with DCM-TFA (10:1, 20 mL) at 0 ℃. After stirred for three hours, it was quenched with water and extracted with EtOAc (20 mL x 3). The combined organic layers were washed with brine (20 mL), dried over Na_2SO_4, filtered, and concentrated. Flash column chromatography on silica gel afforded 1.05 g (78% in three steps) of compound **26** as a colorless oil. ^{1}H NMR (400 MHz, $CDCl_3$) δ 7.72 (d, J = 8.4 Hz, 2H), 7.28 (d, J = 8.4 Hz, 2H), 5.47-5.43 (m, 1H), 4.93-4.87 (m, 1H), 3.86 (d, J = 6.4 Hz, 2H), 3.45 (d, J = 6.0 Hz, 2H), 3.26 (d, J = 1.6 Hz, 3H), 2.41 (s, 3H), 1.60 (s, 3H) ppm; ^{13}C NMR (100 MHz, $CDCl_3$) δ 143.6, 137.2, 134.9, 129.8, 127.3, 124.5, 68.6, 58.2, 50.7, 21.7, 14.8 ppm; IR (thin film) 3286, 2924, 2826, 2355,

1598, 1446, 1384, 1330, 1160, 1093, 980, 860, 815, 707, 663, 591, 552, 475 (cm^{-1}); HRMS (ESI) m/z: Calcd for $C_{13}H_{19}NNaO_3S$ $[M + Na]^+$, 292.0978; Found, 292.0986.

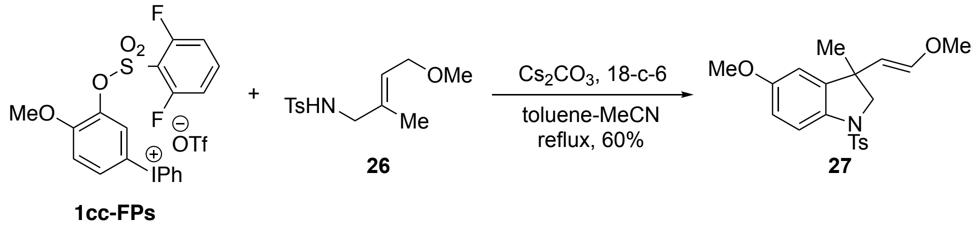

Compound 27: A suspension of compound **26** (134.5 mg, 0.5 mmol, 1.0 equiv), Cs_2CO_3 (650 mg, 2.0 mmol, 4.0 equiv), and 18-c-6 (79.2 mg, 0.3 mmol, 0.6 equiv) in anhydrous toluene (20 mL) under inert atmosphere was heated to reflux for 30 minutes. A solution of aryne precursor **1cc-FPs** (652.0 mg, 1.0 mmol, 2.0 equiv) in MeCN (10 mL) was then added to this suspension over two hours via a syringe pump. After addition, it was heated for additional 30 minutes. All the volatiles were removed directly on a rotary evaporator. Flash column chromatography with pet ether and EtOAc afforded 112.0 mg (60%) of compound **27** as a colorless oil. ^1H NMR (400 MHz, $CDCl_3$) δ 7.67 (d, J = 8.4 Hz, 2H), 7.57 (d, J = 8.8 Hz, 1H), 7.22 (d, J = 8.0 Hz, 2H), 6.75 (dd, J_1 = 8.8 Hz, J_2 = 2.4 Hz, 1H), 6.52 (d, J = 2.8 Hz, 1H), 6.03 (d, J = 12.8 Hz, 1H), 4.59 (d, J = 13.2 Hz, 1H), 3.75 (s, 3H), 3.68 (d, J = 1.2 Hz, 2H), 3.37 (s, 3H), 2.36 (s, 3H), 1.15 (s, 3H) ppm; ^{13}C NMR (100 MHz, $CDCl_3$) δ 157.0, 148.2, 144.2, 140.9, 134.6, 134.2, 129.8, 127.6, 116.0, 113.3, 109.9, 109.2, 64.6, 56.1, 55.9, 43.8, 26.2, 21.7 ppm; IR (thin film) 3395, 3233, 2822, 2354, 1651, 1482, 1400, 1352, 1275, 1217, 1164, 1091, 983, 868, 814, 737, 668 (cm^{-1}); HRMS (ESI) m/z: Calcd for $C_{20}H_{23}NNaO_4S$ $[M + Na]^+$, 396.1240; Found, 396.1245.

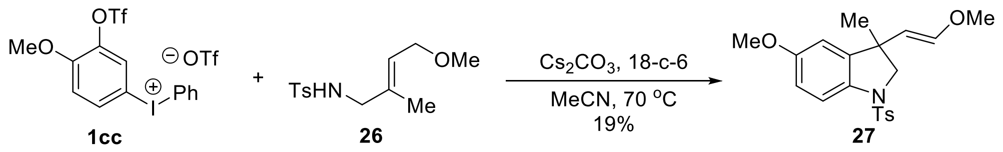

A suspension of compound **26** (134.5 mg, 0.5 mmol, 1.0 equiv), Cs_2CO_3 (650 mg, 2.0 mmol, 4.0 equiv), and 18-c-6 (79.2 mg, 0.3 mmol, 0.6 equiv) in anhydrous MeCN (20 mL) under inert atmosphere was heated to 70 ℃ for 30 minutes. A solution of aryne precursor **1cc** (607.9 mg, 1.0 mmol, 2.0 equiv) in MeCN (10 mL) was then added to this suspension over two hours via a syringe pump. After addition, it was heated for additional 30 minutes. All the volatiles were removed directly on a rotary evaporator. Flash column chromatography with pet ether and EtOAc afforded 35.4 mg (19%) of compound **27** as a colorless oil.

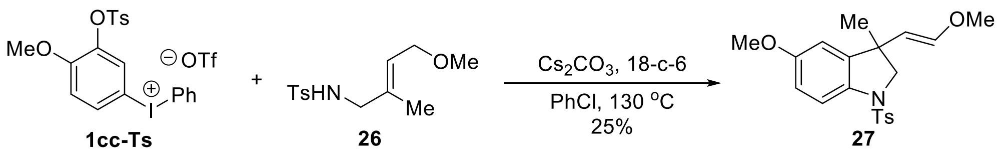

A suspension of compound **26** (134.5 mg, 0.5 mmol, 1.0 equiv), Cs_2CO_3 (650 mg, 2.0 mmol, 4.0 equiv), and 18-c-6 (79.2 mg, 0.3 mmol, 0.6 equiv) in anhydrous PhCl (20 mL) under inert atmosphere was heated 130 ℃ for 30 minutes. A solution of aryne precursor **1cc-Ts** (630.0 mg, 1.0 mmol, 2.0 equiv) in PhCl (10 mL) was then added to this suspension over two hours via a syringe pump. After addition, it was heated for additional 30 minutes. All the volatiles were removed directly on a rotary evaporator. Flash column chromatography with pet ether and EtOAc afforded 46.6 mg (25%) of compound **27** as a colorless oil.

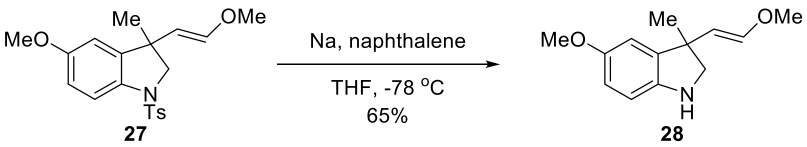

Compound 28: To a solution of compound **27** (89.5 mg, 0.24 mmol, 1.0 equiv) in anhydrous THF (3 mL) at -78 ℃ was slowly added a sodium-naphthalene solution (1.0 M in THF, 2.4 mL, 2.4 mmol, 10.0 equiv). After 10 minutes, it was quenched with water and extracted with EtOAc (10 mL x 3). The combined organic layers were washed with brine (10 mL), dried over Na_2SO_4, filtered, and concentrated. Flash column chromatography on silica gel afforded 34.2 mg (65%) of compound **28** as a colorless oil. ^1H NMR (400 MHz, $CDCl_3$) δ 7.65-7.63 (m, 3H), 6.30 (d, J = 12.8 Hz, 1H), 4.96 (d, J = 12.8 Hz, 1H), 3.75 (s, 3H), 3.51 (s, 3H), 3.41 (d, J = 8.8 Hz, 1H), 3.32 (d, J = 8.8 Hz, 1H), 1.37 (s, 3H) ppm; ^{13}C NMR (100 MHz, $CDCl_3$) δ 154.1, 147.5, 144.1, 138.9, 112.8, 111.2, 110.2, 110.1, 62.7, 56.2, 56.2, 45.6, 25.3 ppm; IR (thin film) 3406, 2361, 1648, 1492, 1400, 1386, 1303, 1265, 1213, 1091, 984, 933, 867 (cm^{-1}); HRMS (ESI) m/z: Calcd for $C_{13}H_{18}NO_2$ $[M + H]^+$, 220.1332; Found, 220.1335.

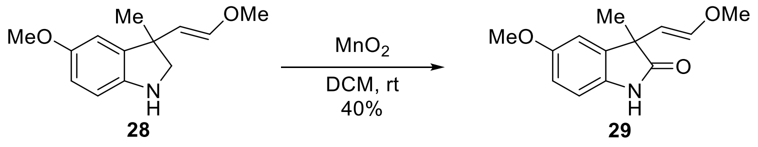

Compound 29: To a solution of compound **28** (15 mg, 0.068 mmol, 1.0 equiv) in anhydrous DCM (1 mL) at 25 °C was added MnO_2 (119.0 mg, 1.37 mmol, 20.0 equiv). After stirred for 30 hours, the residue MnO_2 powder was filtered out. The resulting mixture was concentrated directly on a rotary evaporator. Flash column chromatography afforded 6.4 mg (40%) of compound **29** as a colorless oil. ^1H NMR (400 MHz, $CDCl_3$) δ 6.81-6.74 (m, 3H), 6.35 (d, J = 12.8 Hz, 1H), 4.91 (d, J = 12.8

Hz, 1H), 3.80 (s, 3H), 3.52 (s, 3H), 1.48 (s, 3H) ppm; ^{13}C NMR (100 MHz, $CDCl_3$) δ 181.5, 156.1, 149.2, 135.6, 133.2, 112.7, 111.5, 110.3, 104.2, 56.2, 56.0, 48.5, 24.2 ppm; IR (thin film) 3405, 3223, 2571, 2362, 2327, 1654, 1400, 1282, 1091, 984, 868, 708, 673 (cm-1); HRMS (ESI) m/z: Calcd for $C_{13}H_{15}NNaO_3$ $[M + Na]^+$, 256.0944; Found, 256.0949.

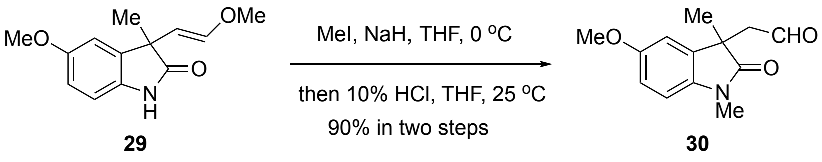

Compound 30: To a solution of compound **29** (10.0 mg, 0.043 mmol, 1.0 equiv) in anhydrous THF (1 mL) at 0 ℃ was added MeI (12.2 mg, 0.086 mmol, 2.0 equiv). After 5 minutes, NaH (60% in mineral oil, 3.4 mg, 0.086 mmol, 2.0 equiv) was added. After stirred for 10 minutes, aq. 10% HCl (0.3 mL) was directly added. The resulting solution was stirred at 25 °C overnight. It was quenched with water and extracted with EtOAc (5 mL x 3). The combined organic layers were washed with brine (3 mL), dried over Na_2SO_4, filtered, and concentrated. Flash column chromatography on silica gel afforded 9.0 mg (90% in two steps) of compound **30**, the ^{1}H NMR of which is identical with that reported in literature.[37] ^{1}H NMR (400 MHz, $CDCl_3$) δ 9.51 (s, 1H), 6.80-6.78 (m, 3H) 3.78 (s, 3H), 3.24 (s, 3H), 2.96 (dd, J_1 = 11.6 Hz, J_2 = 2.0 Hz, 2H), 1.41 (s, 3H) ppm.

2.13. Crystal data and structure refinement for compound 1b

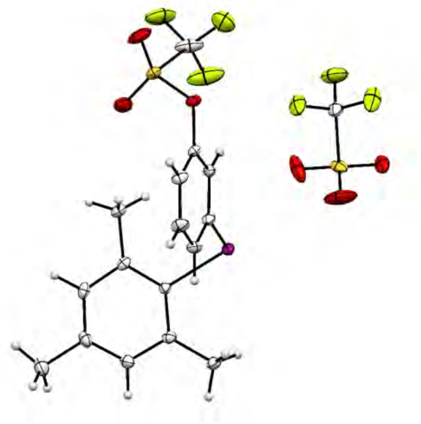

Supplementary Fig. 1. Thermal ellipsoid plot (30% probability) of X-ray structure of **1b**. Hydrogen, white; Carbon, gray; Iodine, purple; Sulfur, yellow; Oxygen, red; Fluorine, green. CCDC of **1b**: 2052067.

Supplementary Table 1. The crystal parameters of compound 1b

Empirical formula	$C_{17}H_{15}F_6IO_6S_2$
Formula weight	620.31
T/ K	150
Crystal system	triclinic
Crystal size(mm)	0.15×0.10×0.10
Space group	P-1
a / Å	12.6927(6)
b / Å	13.5150(6)
c / Å	15.5282(8)
α/ °	115.339(5)
β/ °	105.718(4)
γ/ °	96.119(4)
V / Å^3	2240.3(2)
Z	4
D_c /g cm^{-3}	1.839
μ /mm^{-1}	1.697
θ range/ °	6.726 to 58.2882
F(000)	1216.0
Reflections collected	17687
Independent reflections	10171
GOF(F^2)	1.048
Data/restraints/parameters	10171/1/583
R [I >2σ (I)]	R_1 = 0.0379
	wR_2 =0.0825
R (all data)	R_1 = 0.0513
	wR_2 = 0.0918

Supplementary Table 2. Bond lengths [Å] and angles [deg] for 1b

I(1)-C(5)	2.018(3)	C(9)-C(10)	1.388(5)
I(1)-C(13)	2.113(3)	C(10)-C(11)	1.394(6)
S(1)-O(1)	1.418(3)	C(10)-C(15)	1.518(5)
S(1)-O(2)	1.407(3)	C(11)-C(12)	1.397(5)
S(1)-O(3)	1.561(3)	C(12)-C(13)	1.510(5)
S(1)-C(7)	1.826(6)	C(12)-C(16)	1.510(5)
F(1)-C(7)	1.307(7)	I(2)-C(21)	2.105(4)
F(2)-C(7)	1.313(6)	I(2)-C(26)	2.118(4)
F(3)-C(7)	1.316(5)	S(4)-O(10)	1.575(3)
O(3)-C(1)	1.434(4)	S(4)-O(11)	1.416(4)
C(1)-C(2)	1.367(6)	S(4)-O(12)	1.398(4)
C(1)-C(6)	1.384(5)	S(4)-O(18)	1.848(6)
C(2)-C(3)	1.387(5)	F(7)-C(18)	1.340(6)
F(8)-C(18)	1.290(6)	C(27)-C(28)	1.398(5)
F(9)-C(18)	1.290(7)	C(27)-C(31)	1.497(5)
O(10)-C(19)	1.431(4)	C(28)-C(29)	1.377(6)
C(19)-C(20)	1.375(5)	C(29)-C(30)	1.392(6)
C(19)-C(24)	1.371(5)	C(29)-C(32)	1.507(6)
C(20)-C(21)	1.376(5)	S(2)-O(4)	1.427(3)
C(21)-C(22)	1.387(5)	S(2)-O(5)	1.443(3)
C(22)-C(23)	1.378(7)	S(2)-O(6)	1.446(3)
C(23)-C(24)	1.375(5)	S(2)-C(17)	1.825(4)
C(25)-C(26)	1.385(5)	F(4)-C(17)	1.322(5)
C(25)-C(30)	1.388(5)	F(5)-C(17)	1.325(5)
C(25)-C(33)	1.509(5)	F(6)-C(17)	1.333(5)
C(26)-C(27)	1.406(5)	S(3)-O(7)	1.434(3)
C(3)-C(4)	1.385(5)	S(3)-O(8)	1.423(4)
C(4)-C(5)	1.382(5)	S(3)-O(9)	1.421(4)
C(5)-C(6)	1.390(5)	S(3)-C(34)	1.805(4)
C(8)-C(9)	1.385(5)	F(10)-C(34)	1.325(5)
C(8)-C(13)	1.404(5)	F(11)-C(34)	1.331(6)
C(8)-C(14)	1.508(5)	F(12)-C(34)	1.303(5)
C(5)-I(1)-C(13)	96.19(13)	O(1)-S(1)-C(7)	105.9(3)
O(1)-S(1)-O(3)	111.18(17)	O(2)-S(1)-O(1)	122.9(2)

O(2)-S(1)-O(3)	106.16(17)	C(9)-C(8)-C(14)	119.9(3)
O(2)-S(1)-C(7)	107.2(2)	C(13)-C(8)-C(14)	123.4(3)
O(3)-S(1)-C(7)	101.5(2)	C(9)-C(10)-C(11)	118.7(3)
C(1)-O(3)-S(1)	120.9(2)	C(9)-C(10)-C(15)	121.0(4)
C(2)-C(1)-O(3)	119.8(3)	C(11)-C(10)-C(15)	120.2(4)
C(2)-C(1)-C(6)	123.8(3)	C(12)-C(11)-C(10)	122.1(3)
C(6)-C(1)-O(3)	116.3(3)	C(11)-C(12)-C(13)	116.7(3)
C(1)-C(2)-C(3)	118.4(4)	C(11)-C(12)-C(16)	119.3(3)
C(4)-C(3)-C(2)	120.5(4)	C(13)-C(12)-C(16)	124.0(3)
C(5)-C(4)-C(3)	118.7(3)	C(8)-C(13)-I(1)	117.5(2)
C(4)-C(5)-I(1)	118.3(2)	C(12)-C(13)-I(1)	118.9(3)
C(4)-C(5)-C(6)	122.7(3)	C(12)-C(13)-C(8)	123.6(3)
C(6)-C(5)-I(1)	118.9(3)	C(21)-I(2)-C(26)	95.03(14)
F(9)-C(18)-F(7)	108.9(4)	O(10)-S(4)-C(18)	99.7(2)
F(9)-C(18)-F(8)	109.8(6)	O(11)-S(4)-O(10)	110.28(19)
C(20)-C(19)-O(10)	118.9(3)	O(11)-S(4)-C(18)	105.3(3)
C(24)-C(19)-O(10)	118.3(3)	O(12)-S(4)-O(10)	107.6(2)
C(24)-C(19)-C(20)	122.7(3)	O(12)-S(4)-O(11)	125.6(3)
C(19)-C(20)-C(21)	117.7(3)	O(12)-S(4)-C(18)	105.1(2)
C(20)-C(21)-I(2)	117.0(3)	C(19)-O(10)-S(4)	118.0(2)
C(20)-C(21)-C(22)	122.5(4)	F(7)-C(18)-S(4)	106.1(5)
C(22)-C(21)-I(2)	120.5(3)	F(8)-C(18)-S(4)	112.6(4)
C(21)-C(22)-C(23)	117.9(3)	F(8)-C(18)-F(7)	108.9(4)
C(24)-C(23)-C(22)	121.0(4)	F(9)-C(18)-S(4)	110.4(4)
C(19)-C(24)-C(23)	118.8(4)		
C(26)-C(25)-C(30)	116.3(3)	C(27)-C(26)-I(2)	116.7(3)
C(26)-C(25)-C(33)	125.3(3)	C(26)-C(27)-C(31)	124.2(3)
C(30)-C(25)-C(33)	118.4(4)	C(28)-C(27)-C(26)	115.0(4)
C(25)-C(26)-I(2)	118.3(3)	C(28)-C(27)-C(31)	120.8(3)
C(25)-C(26)-C(27)	125.0(3)	C(29)-C(28)-C(27)	122.8(4)
C(1)-C(6)-C(5)	115.8(3)	C(28)-C(29)-C(30)	118.9(4)
F(1)-C(7)-S(1)	110.8(4)	C(28)-C(29)-C(32)	120.9(4)
F(1)-C(7)-F(2)	109.0(5)	C(30)-C(29)-C(32)	120.2(4)
F(1)-C(7)-F(3)	108.7(5)	C(25)-C(30)-C(29)	122.0(4)
F(2)-C(7)-S(1)	108.7(4)	O(4)-S(2)-O(5)	114.95(17)
F(2)-C(7)-F(3)	109.4(4)	O(4)-S(2)-O(6)	115.91(19)
F(3)-C(7)-S(1)	110.3(4)	O(4)-S(2)-C(17)	104.8(2)
C(9)-C(8)-C(13)	116.6(3)	O(5)-S(2)-O(6)	113.07(17)

O(5)-S(2)-C(17)	103.28(19)	O(8)-S(3)-C(34)	102.4(2)
O(6)-S(2)-C(17)	102.71(18)	O(9)-S(3)-O(7)	114.7(2)
F(4)-C(17)-S(2)	111.1(3)	O(9)-S(3)-O(8)	115.4(3)
F(4)-C(17)-F(5)	108.6(4)	O(9)-S(3)-C(34)	103.6(2)
F(4)-C(17)-F(6)	108.0(4)	F(10)-C(34)-F(11)	108.1(4)
F(5)-C(17)-S(2)	111.0(3)	F(11)-C(34)-S(3)	111.0(3)
F(5)-C(17)-F(6)	107.8(3)	F(12)-C(34)-S(3)	113.0(3)
F(6)-C(17)-S(2)	110.2(3)	F(12)-C(34)-F(10)	108.0(4)
O(7)-S(3)-C(34)	103.56(19)	F(12)-C(34)-F(11)	104.1(4)
O(8)-S(3)-O(7)	114.7(3)		

3. Supplementary ^{1}H and ^{13}C NMR spectra

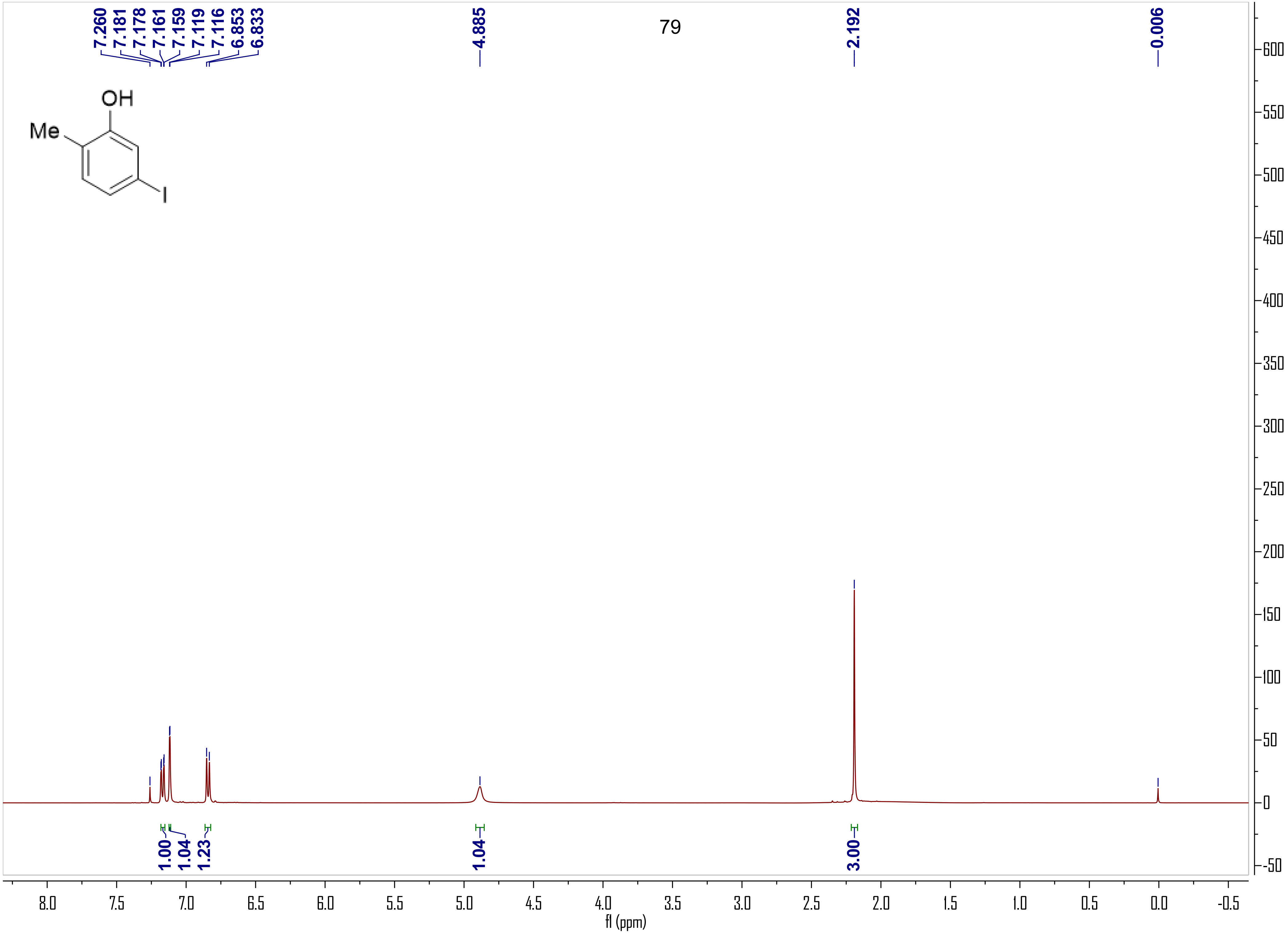

Supplementary Fig 2. ¹H NMR spectrum (400 MHz, $CDCl_3$, r.t.) of 5-iodo-2-methylphenol.

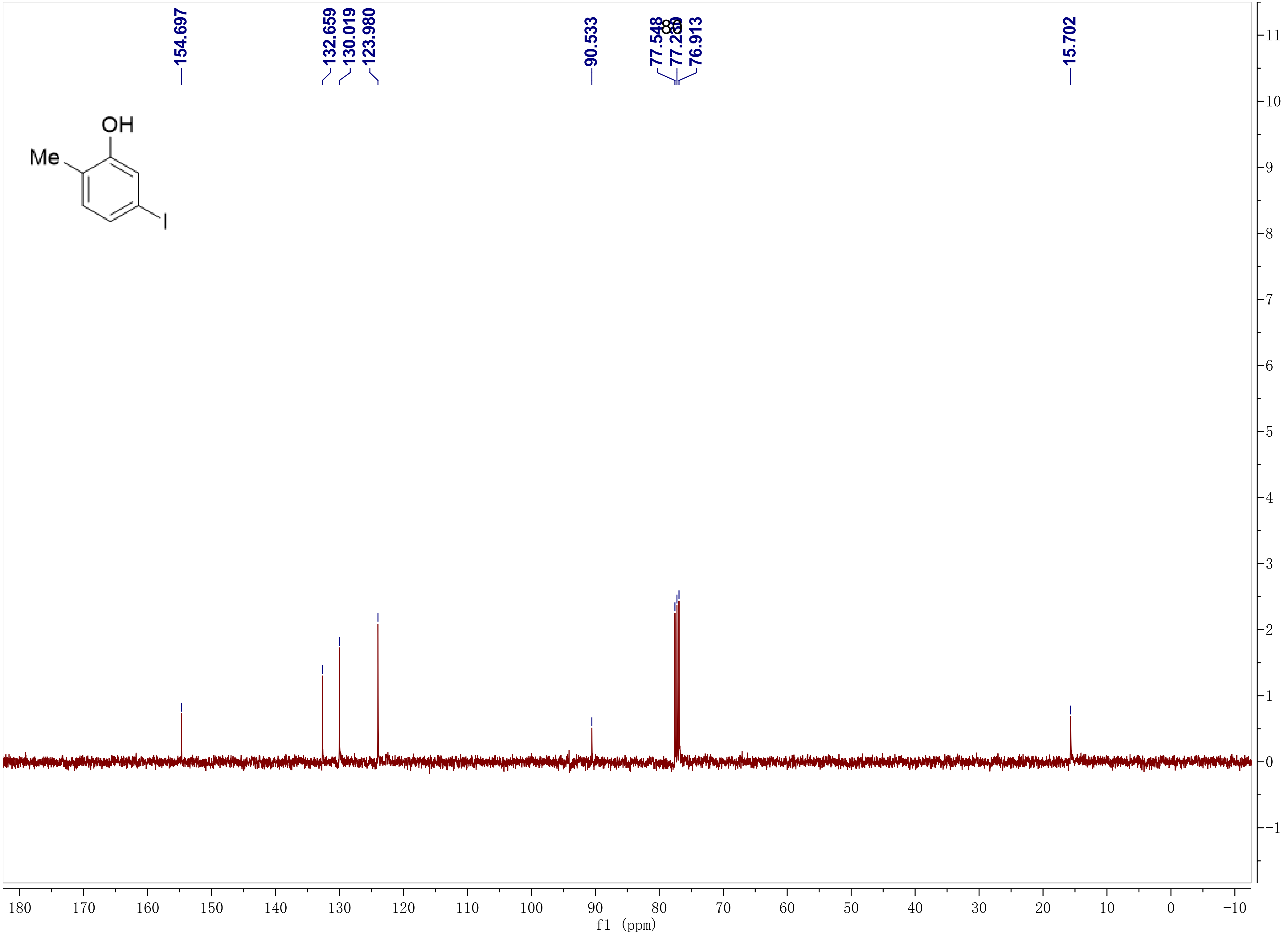

Supplementary Fig 3. ^{13}C NMR spectrum (100 MHz, $CDCl_3$, r.t.) of 5-iodo-2-methylphenol.

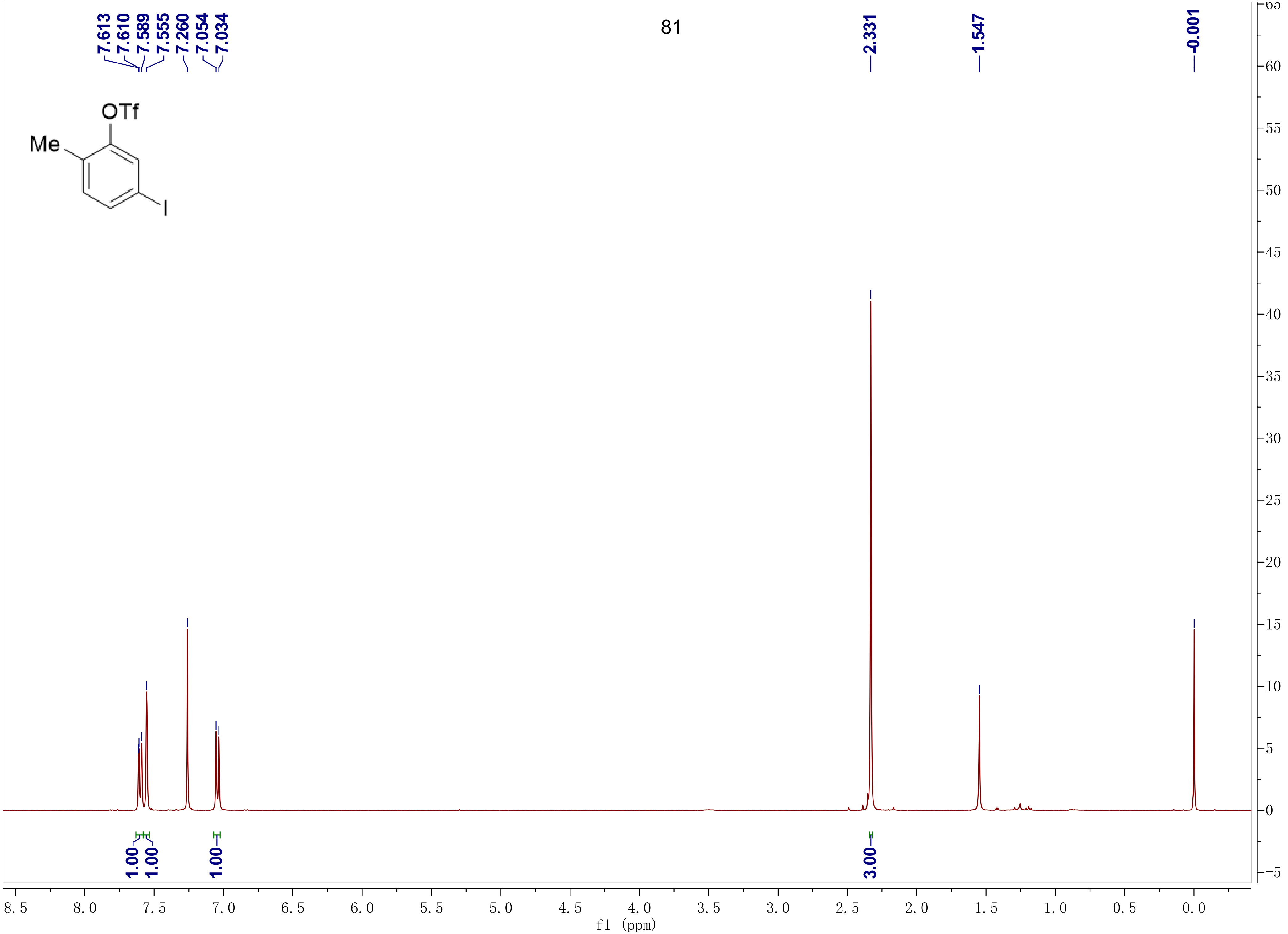

Supplementary Fig 4. ^{1}H NMR spectrum (400 MHz, $CDCl_3$, r.t.) of 5-iodo-2-methylphenyl triflate.

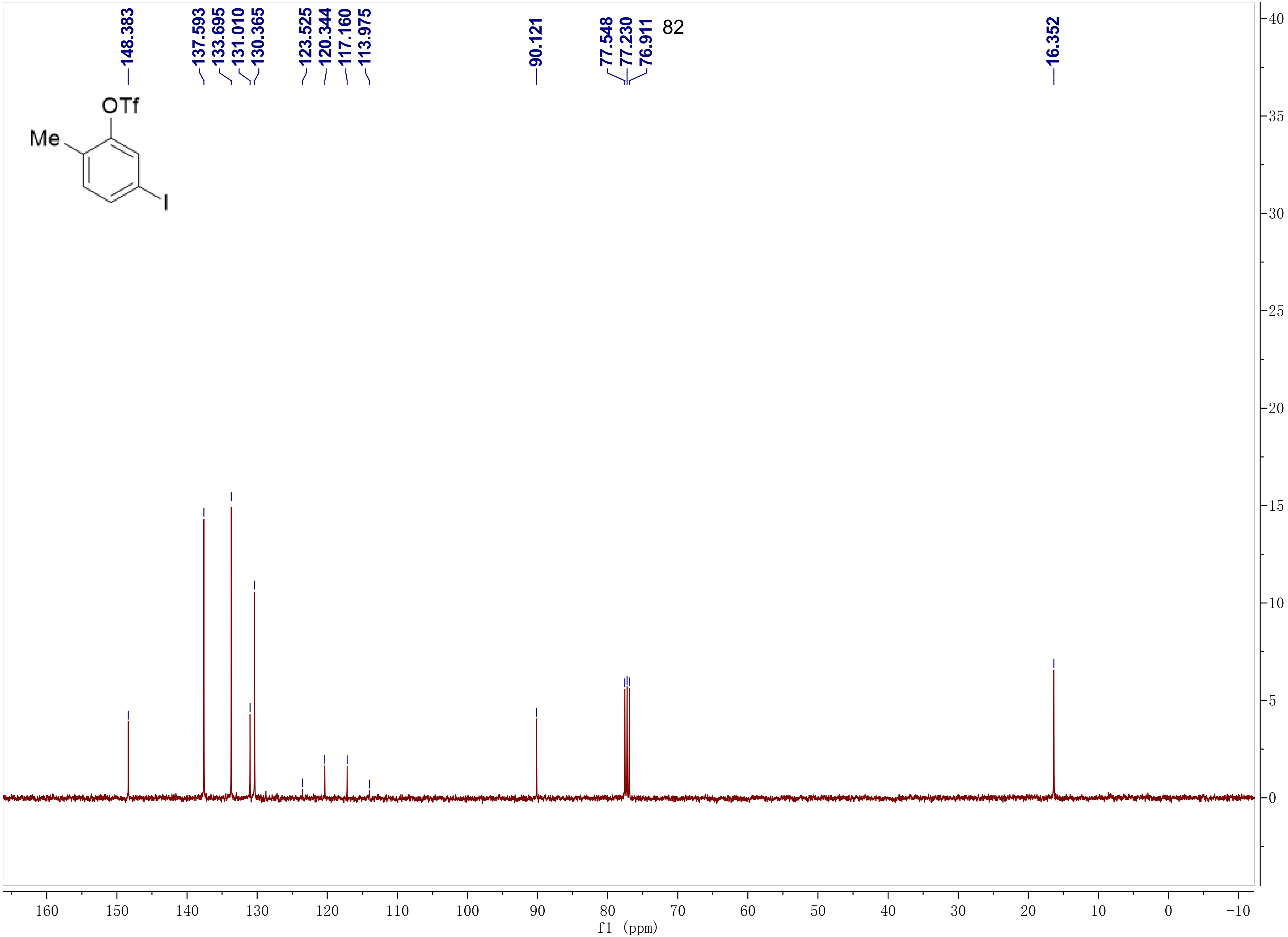

Supplementary Fig 5. ^{13}C NMR spectrum (100 MHz, $CDCl_3$, r.t.) of 5-iodo-2-methylphenyl triflate.

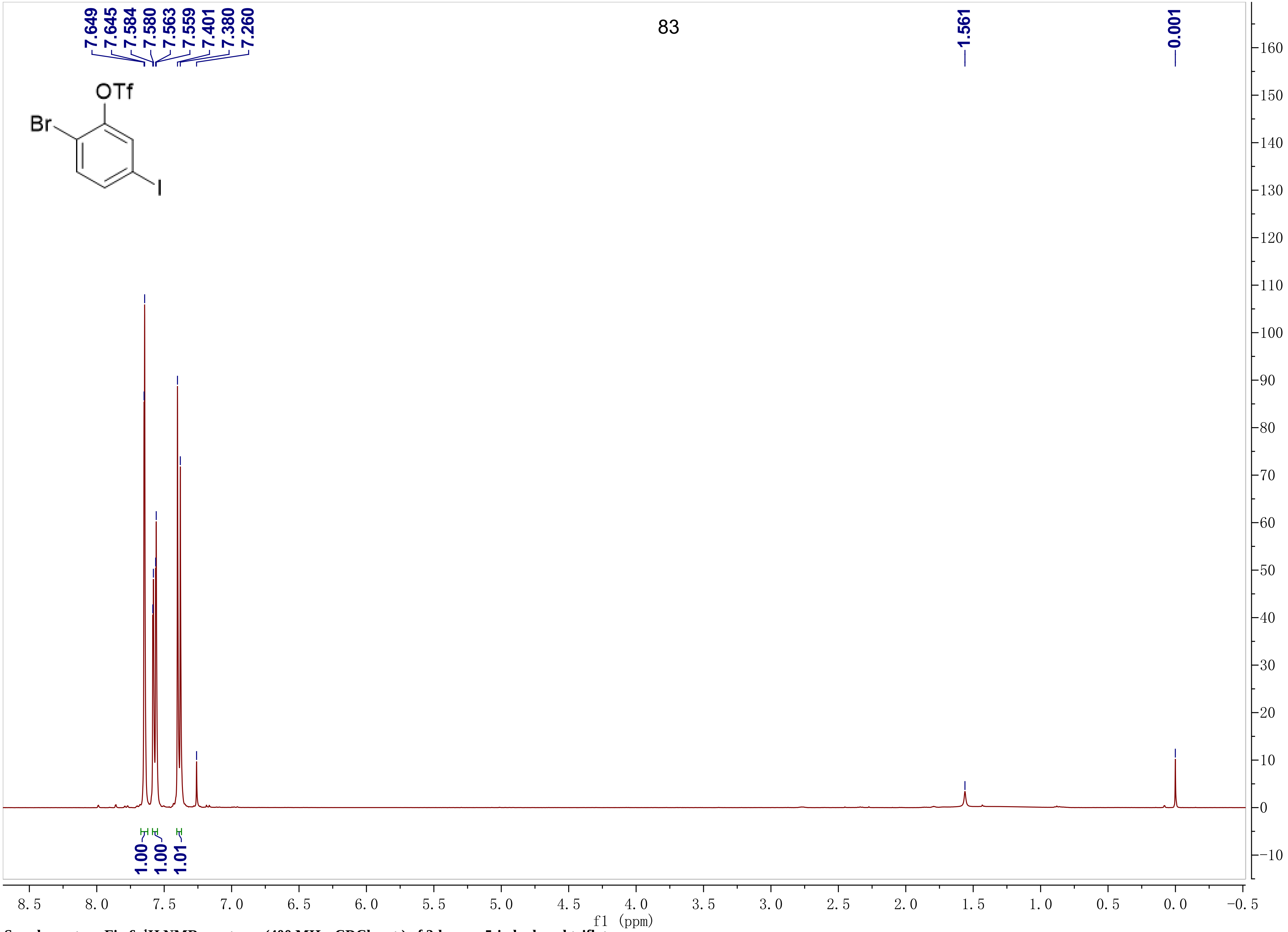

Supplementary Fig 6. ^{1}H NMR spectrum (400 MHz, $CDCl_3$, r.t.) of 2-bromo-5-iodophenyl triflate.

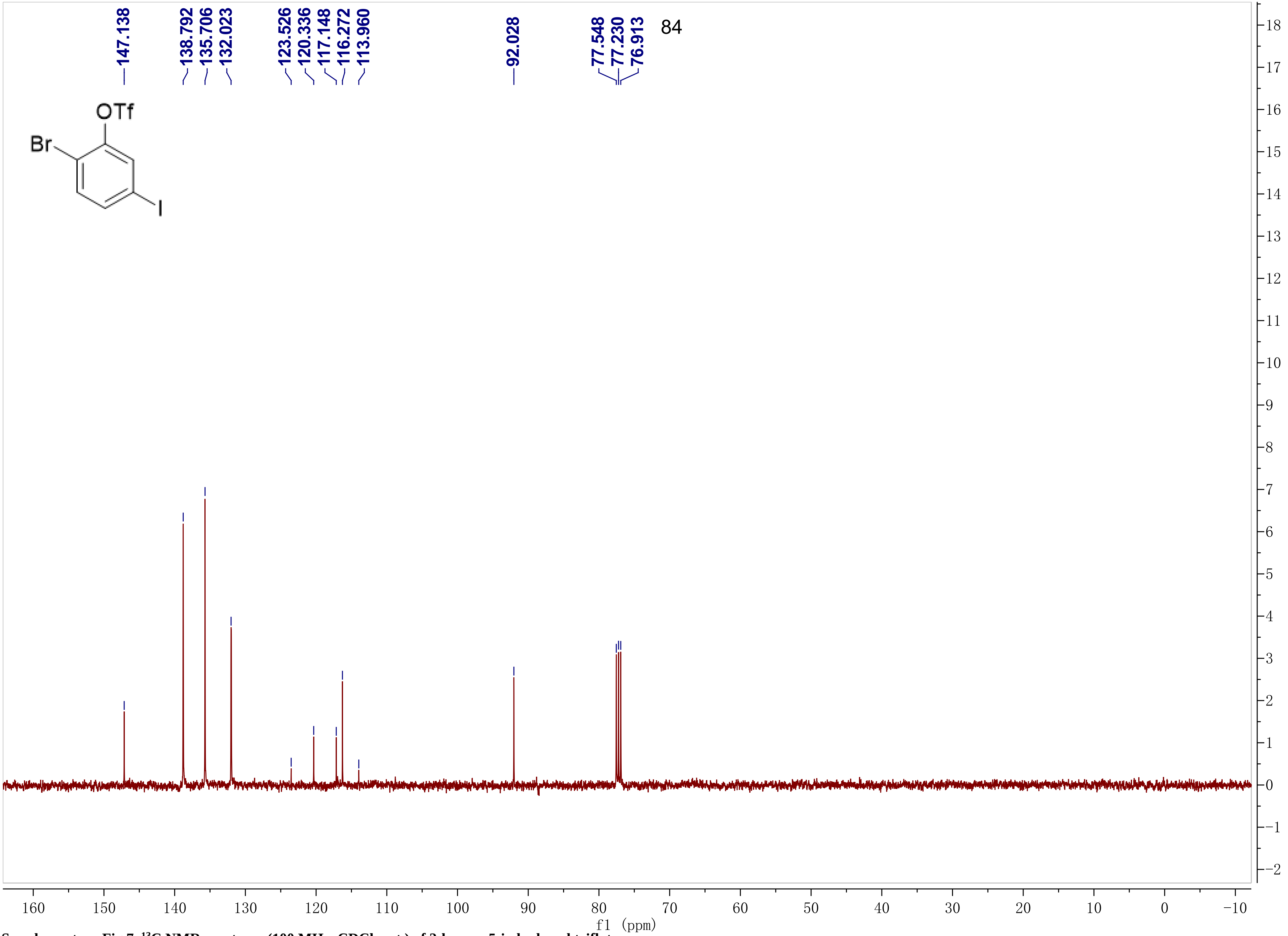

Supplementary Fig 7. ^{13}C NMR spectrum (100 MHz, $CDCl_3$, r.t.) of 2-bromo-5-iodophenyl triflate.

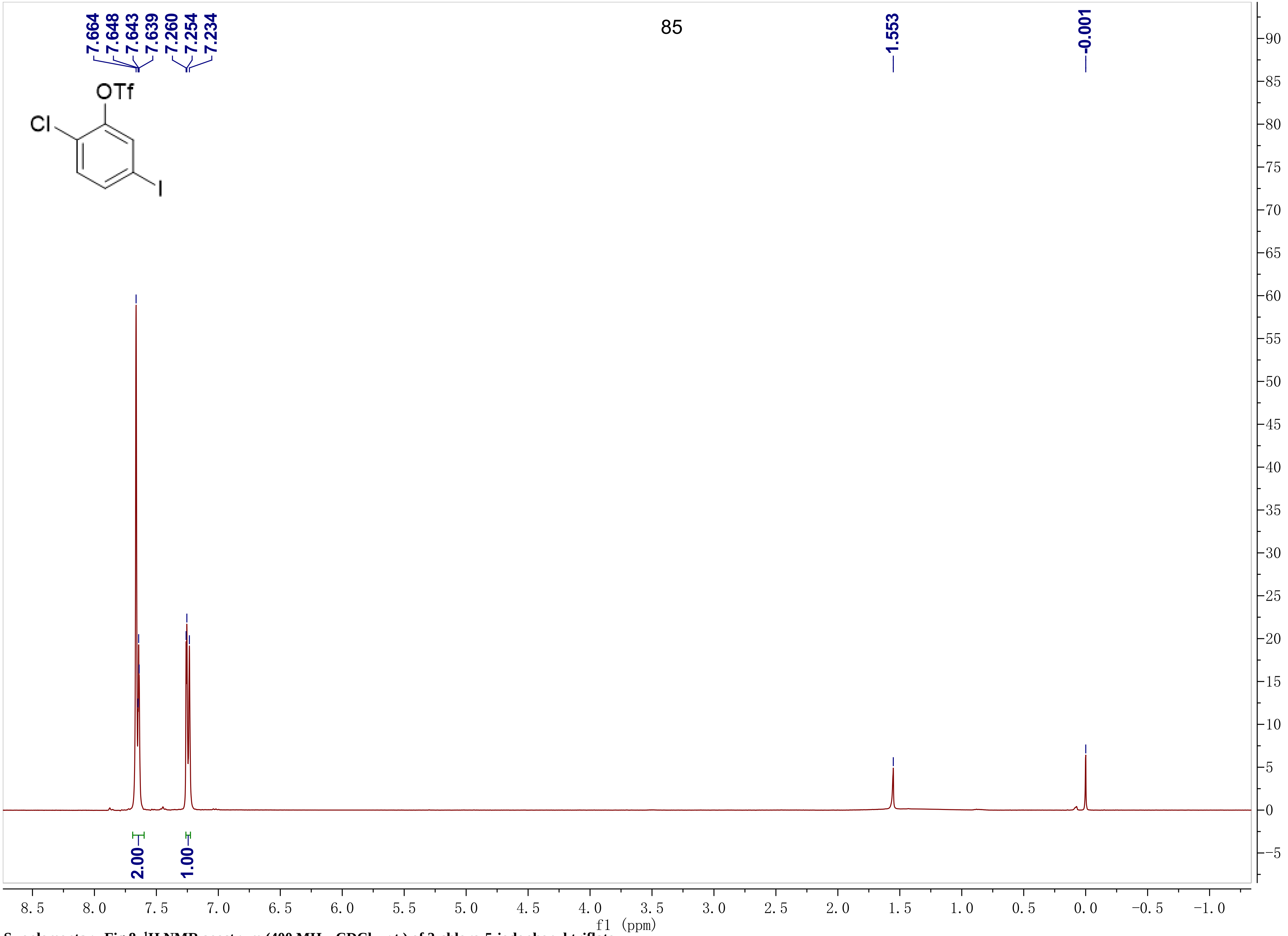

Supplementary Fig 8. **^{1}H NMR spectrum (400 MHz, $CDCl_3$, r.t.) of 2-chloro-5-iodophenyl triflate.**

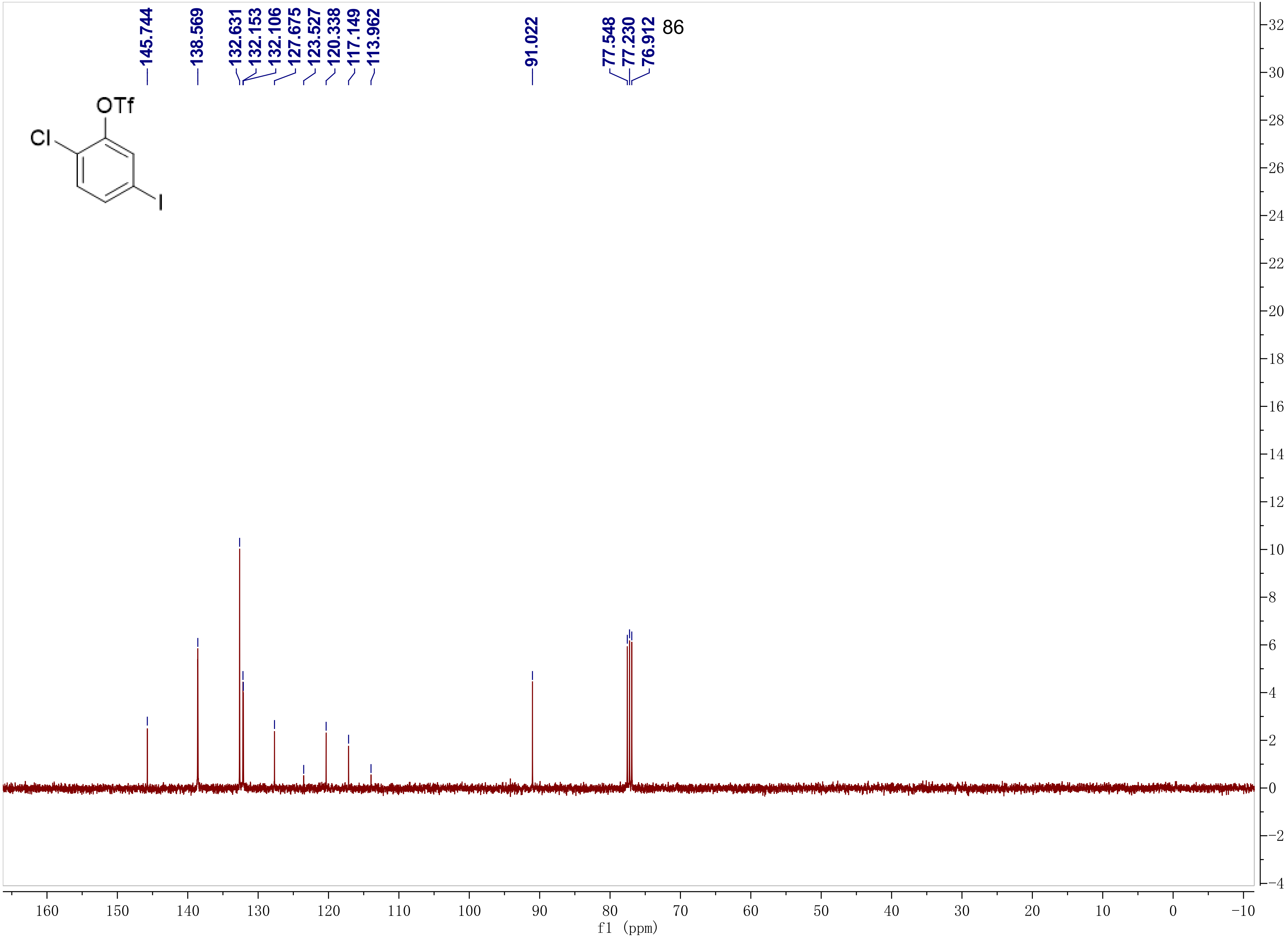

Supplementary Fig 9. ^{13}C NMR spectrum (100 MHz, $CDCl_3$, r.t.) of 2-chloro-5-iodophenyl triflate.

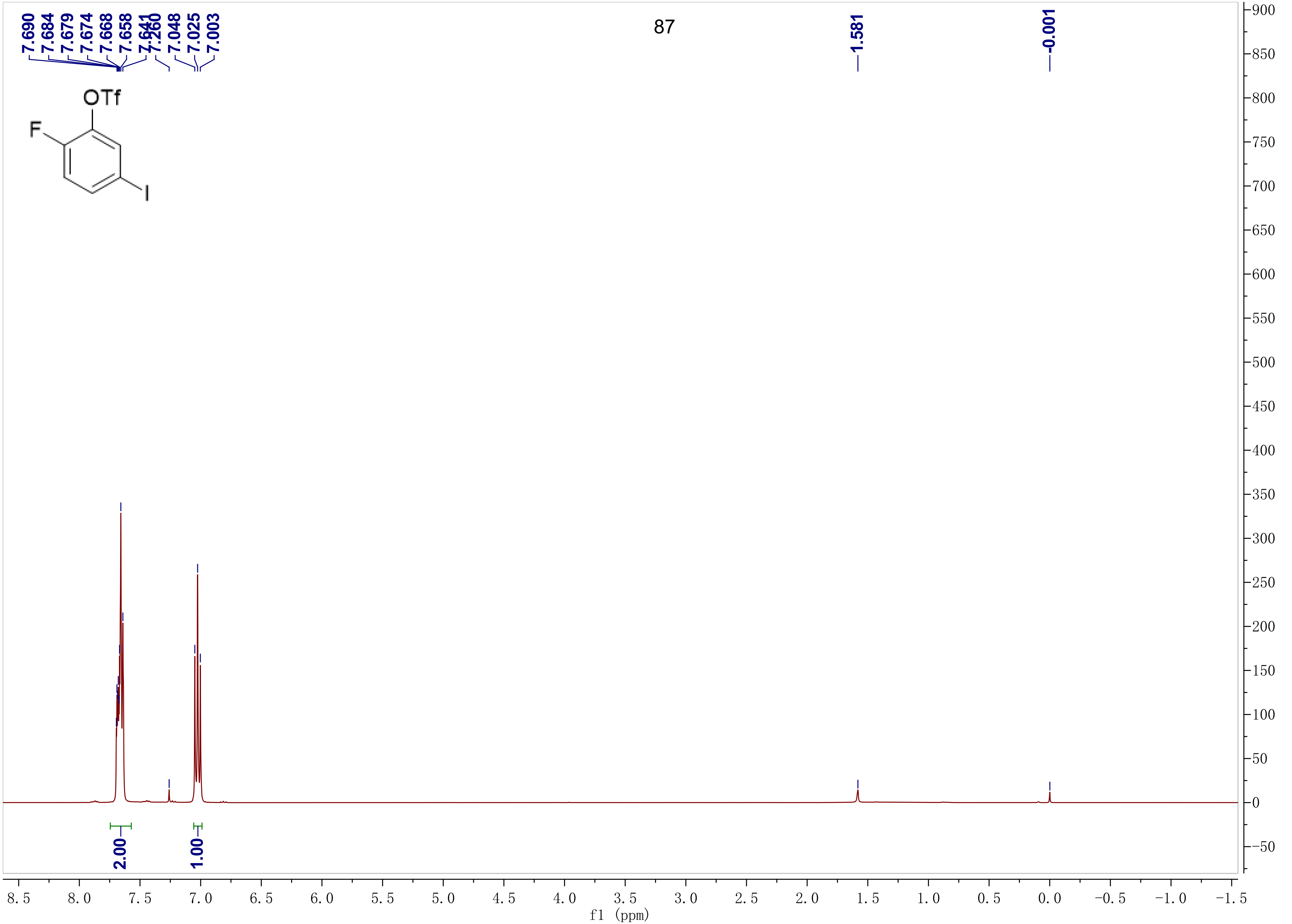

Supplementary Fig 10. ^{1}H NMR spectrum (400 MHz, $CDCl_3$, r.t.) of 2-fluoro-5-iodophenyl triflate.

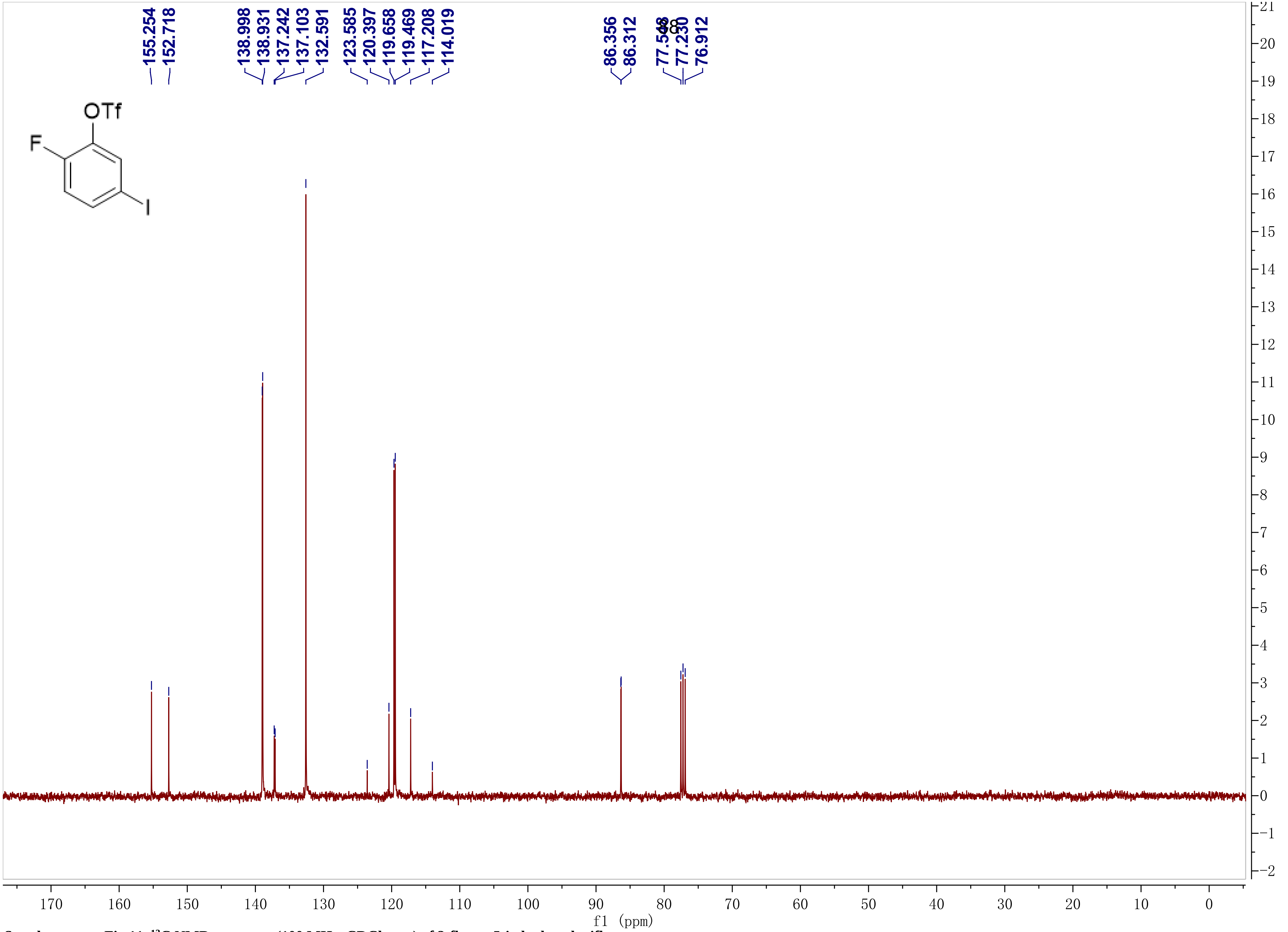

Supplementary Fig 11. ^{13}C NMR spectrum (100 MHz, $CDCl_3$, r.t.) of 2-fluoro-5-iodophenyl triflate.

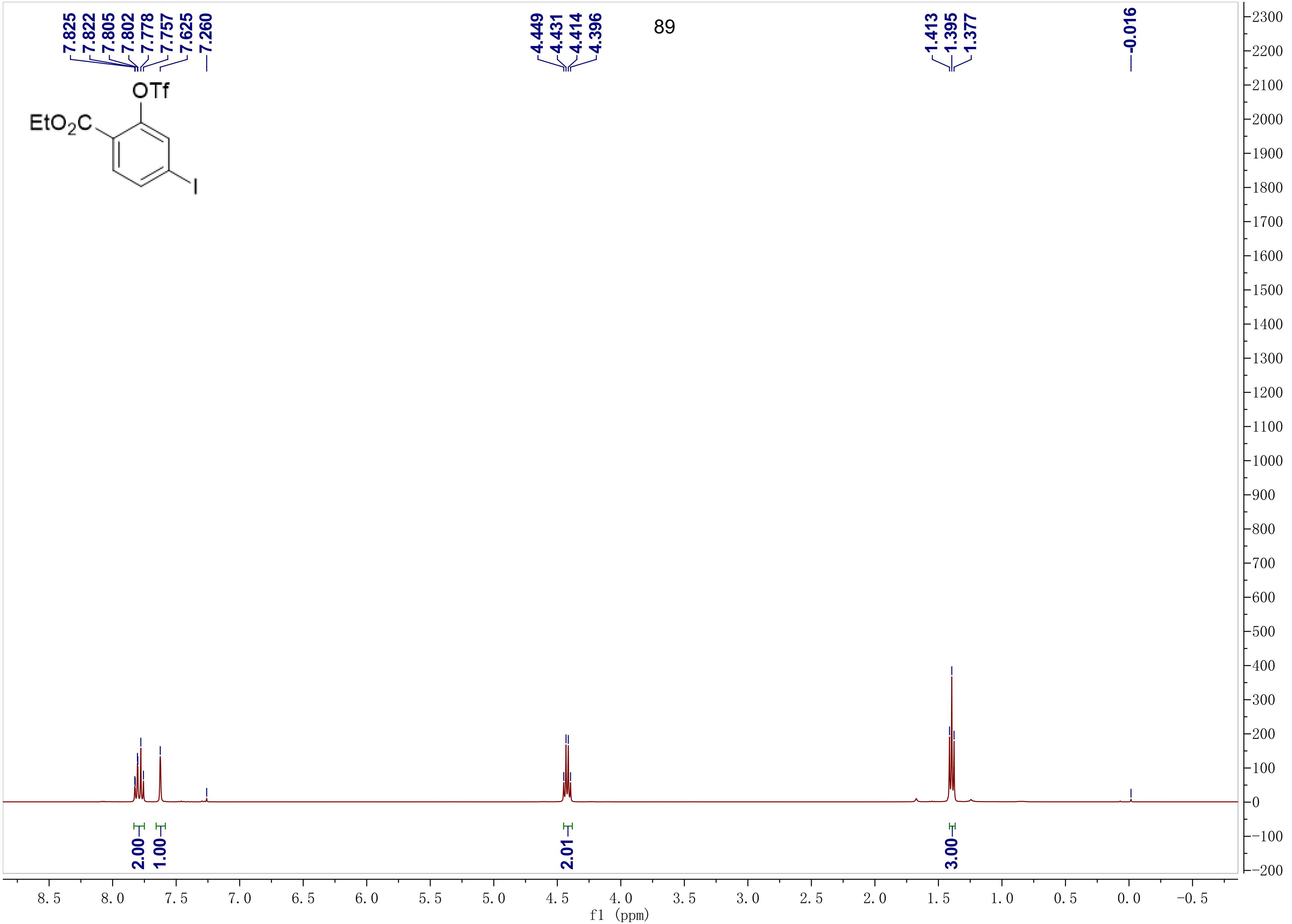

Supplementary Fig 12. ^{1}H NMR spectrum (400 MHz, $CDCl_3$, r.t.) of ethyl 4-iodo-2-(((trifluoromethyl)sulfonyl)oxy)benzoate.

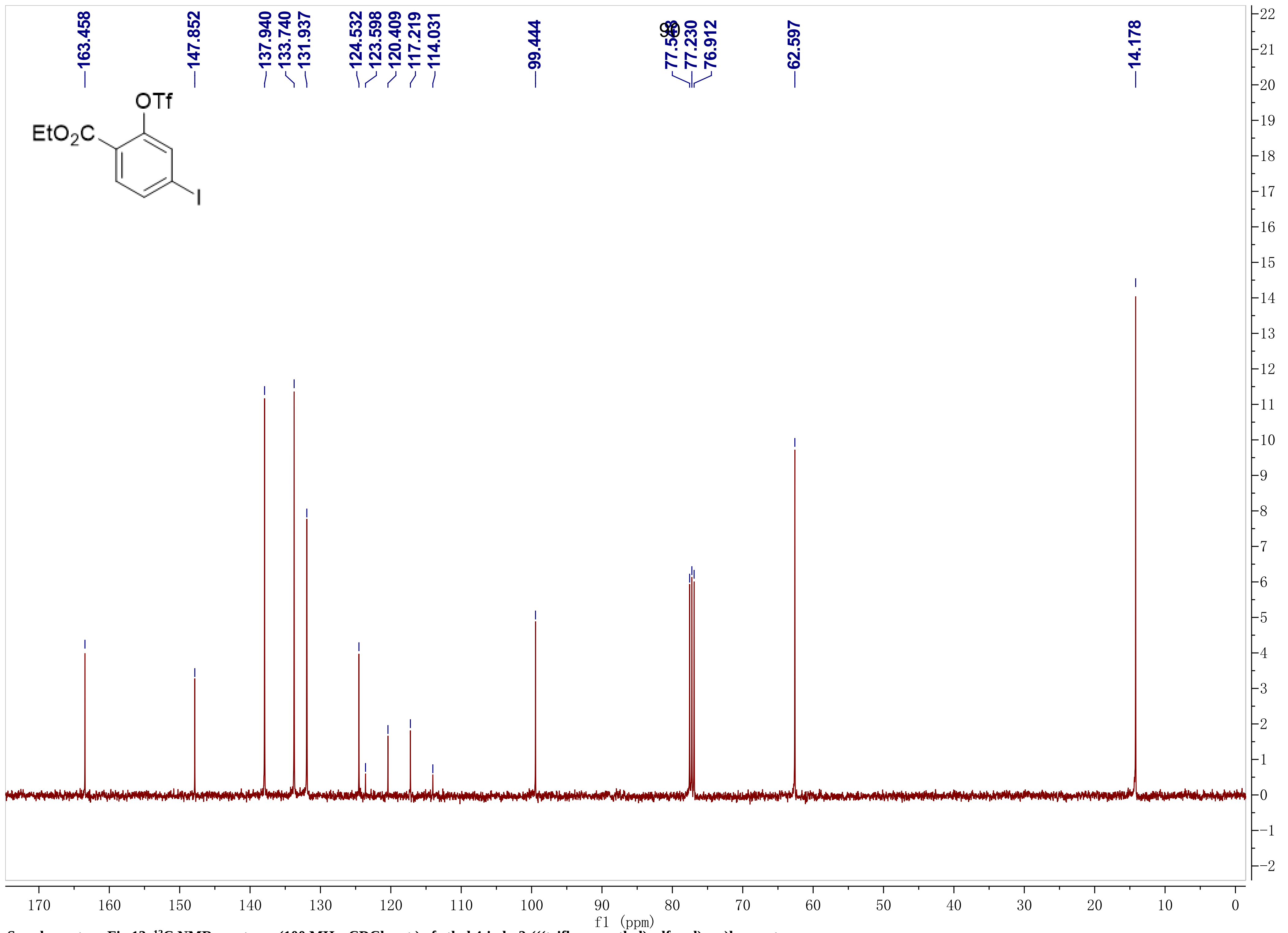

Supplementary Fig 13. **^{13}C NMR spectrum (100 MHz, $CDCl_3$, r.t.) of ethyl 4-iodo-2-(((trifluoromethyl)sulfonyl)oxy)benzoate.**

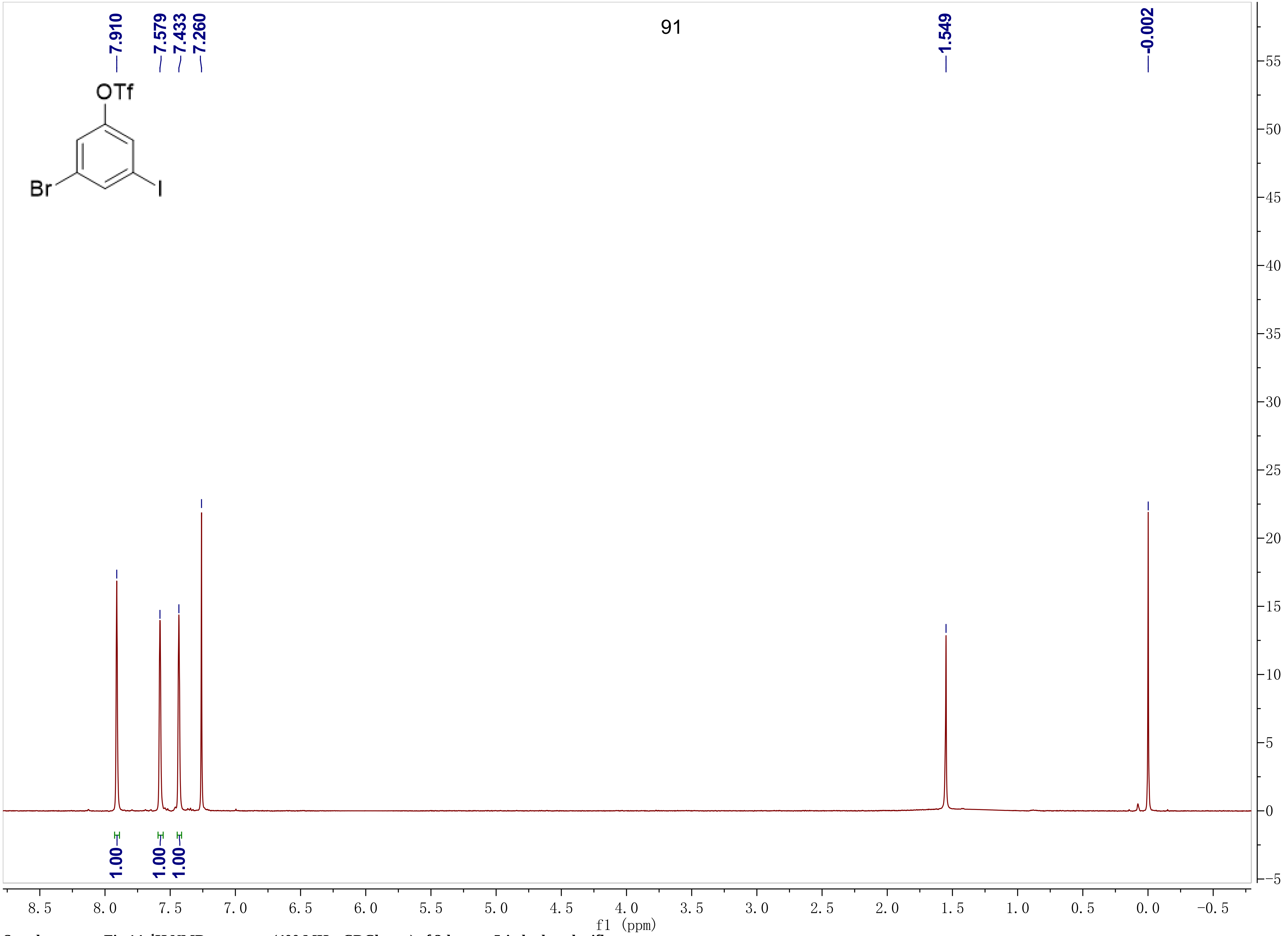

Supplementary Fig 14. ^{1}H NMR spectrum (400 MHz, $CDCl_3$, r.t.) of 3-bromo-5-iodophenyl triflate.

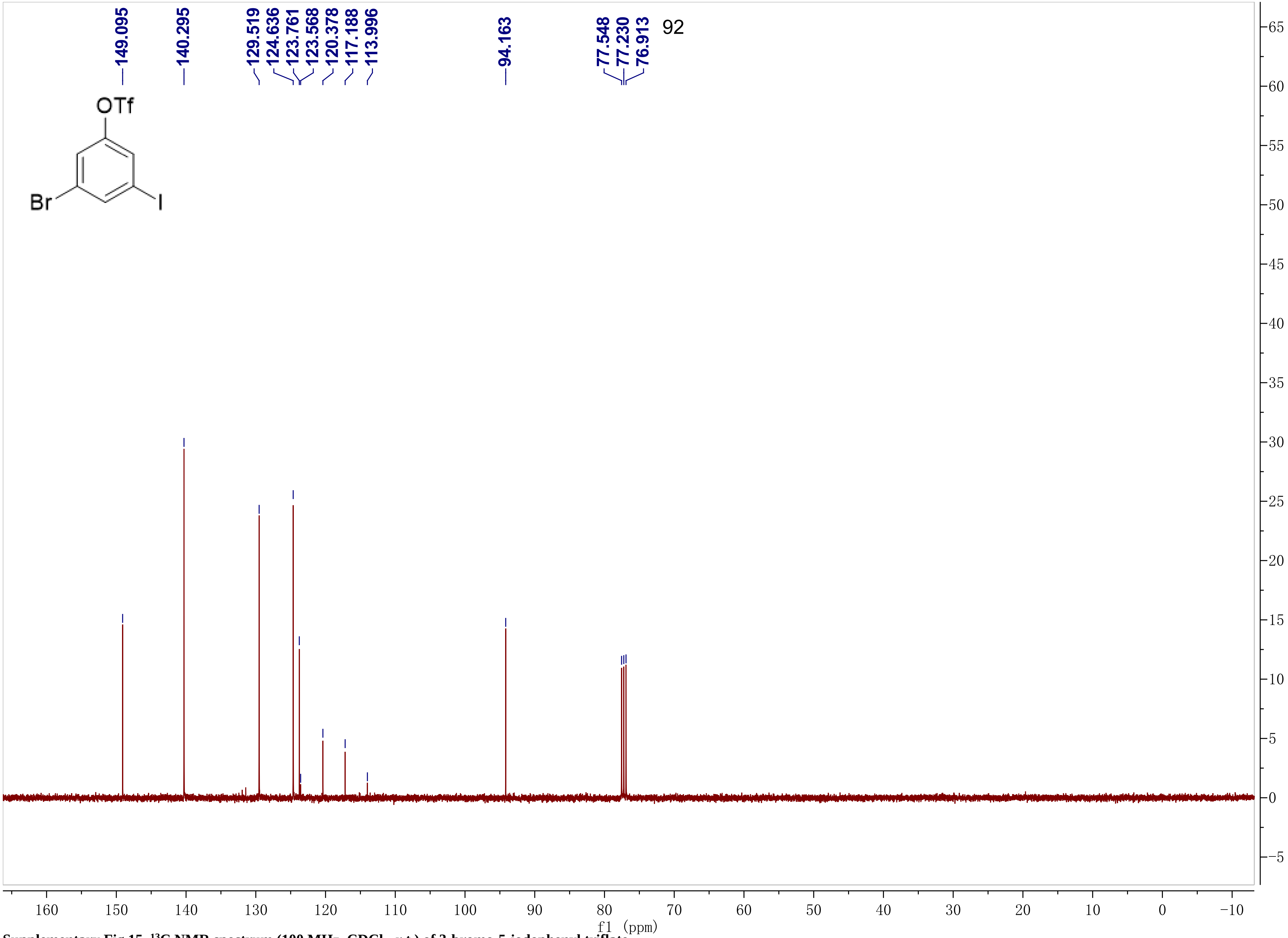

Supplementary Fig 15. ^{13}C NMR spectrum (100 MHz, $CDCl_3$, r.t.) of 3-bromo-5-iodophenyl triflate.

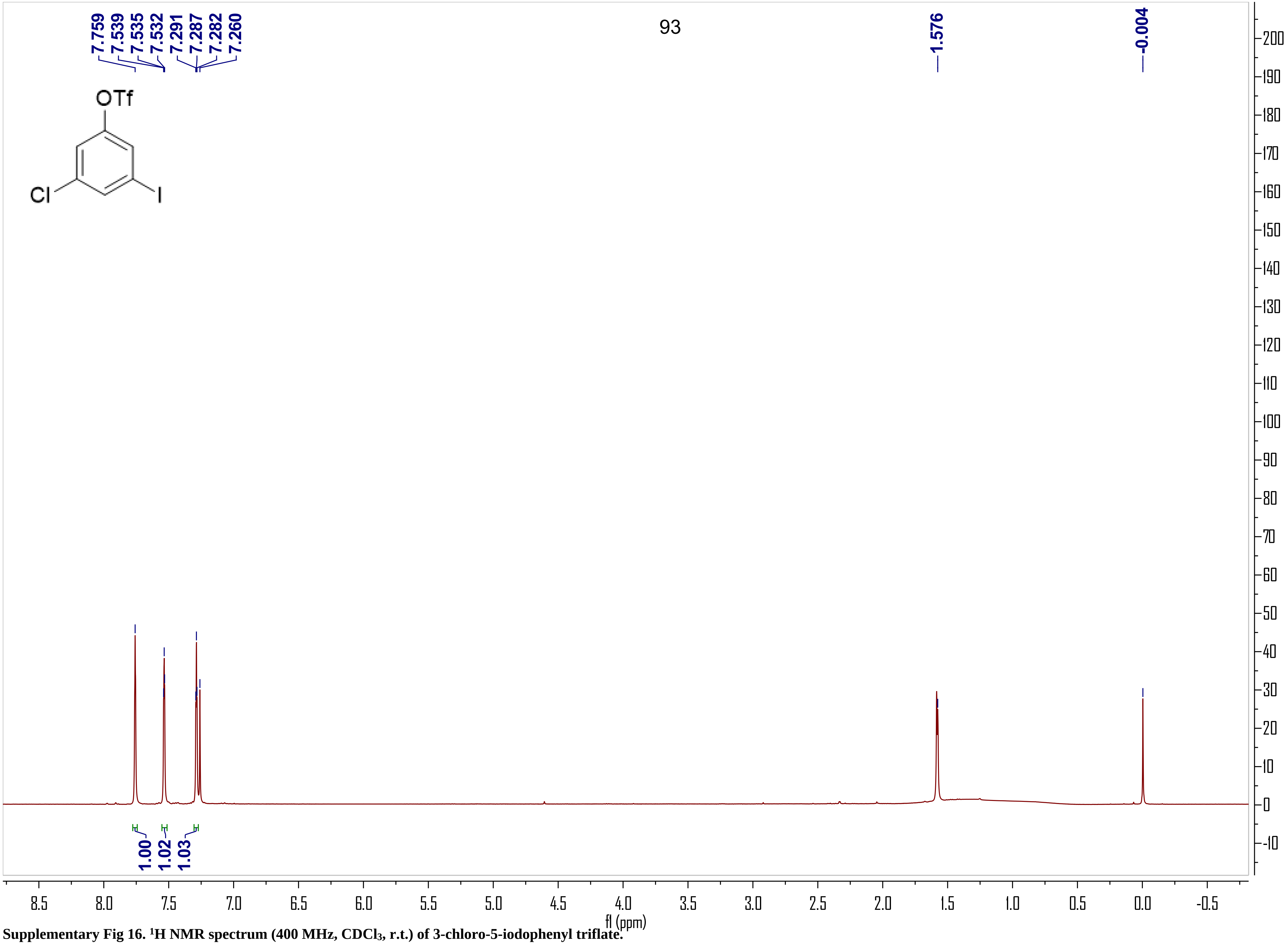

Supplementary Fig 16. **^{1}H NMR spectrum (400 MHz, $CDCl_3$, r.t.) of 3-chloro-5-iodophenyl triflate.**

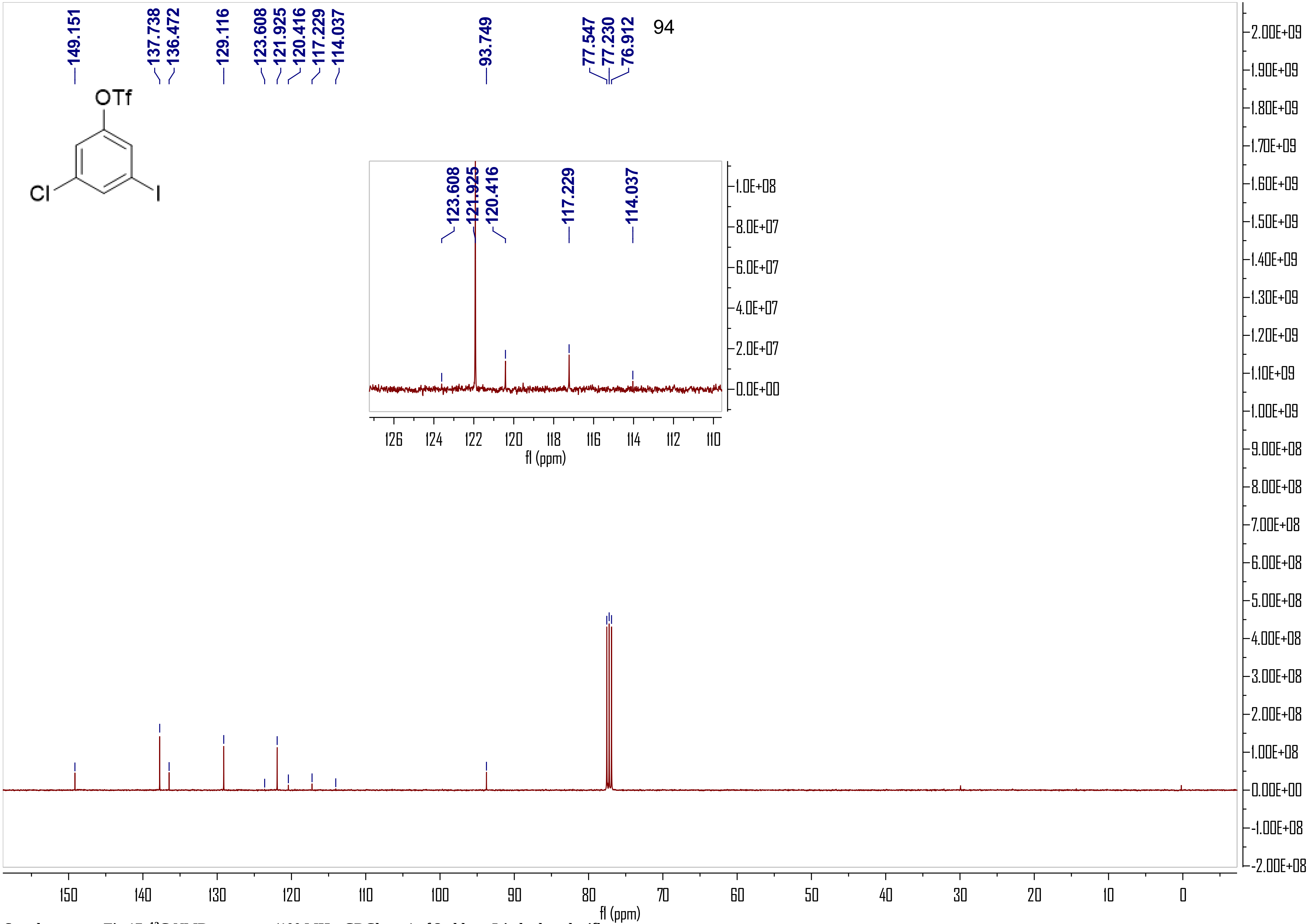

Supplementary Fig 17. ^{13}C **NMR spectrum (100 MHz,** $CDCl_3$**, r.t.) of 3-chloro-5-iodophenyl triflate.**

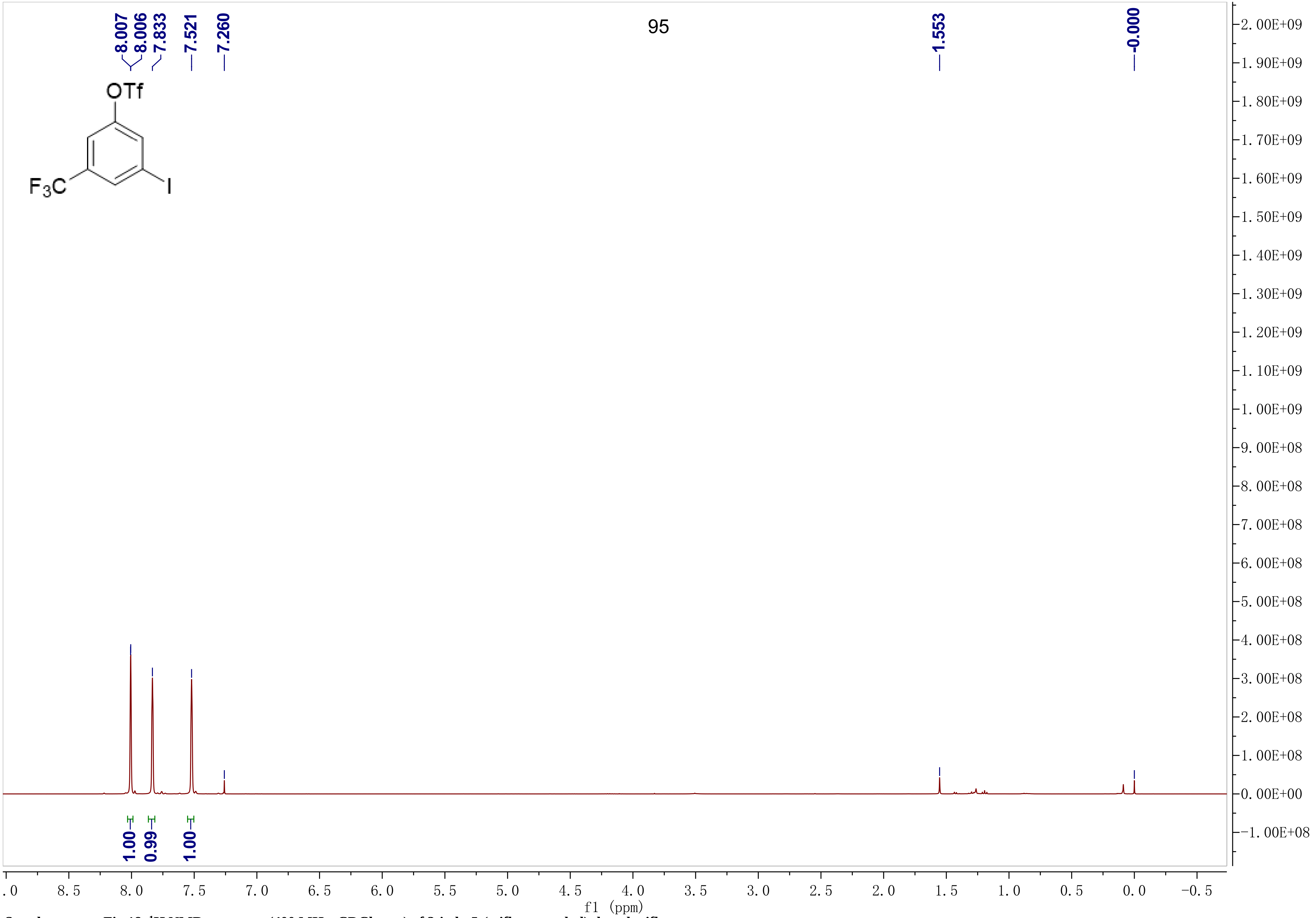

Supplementary Fig 18. ^{1}H NMR spectrum (400 MHz, $CDCl_3$, r.t.) of 3-iodo-5-(trifluoromethyl)phenyl triflate.

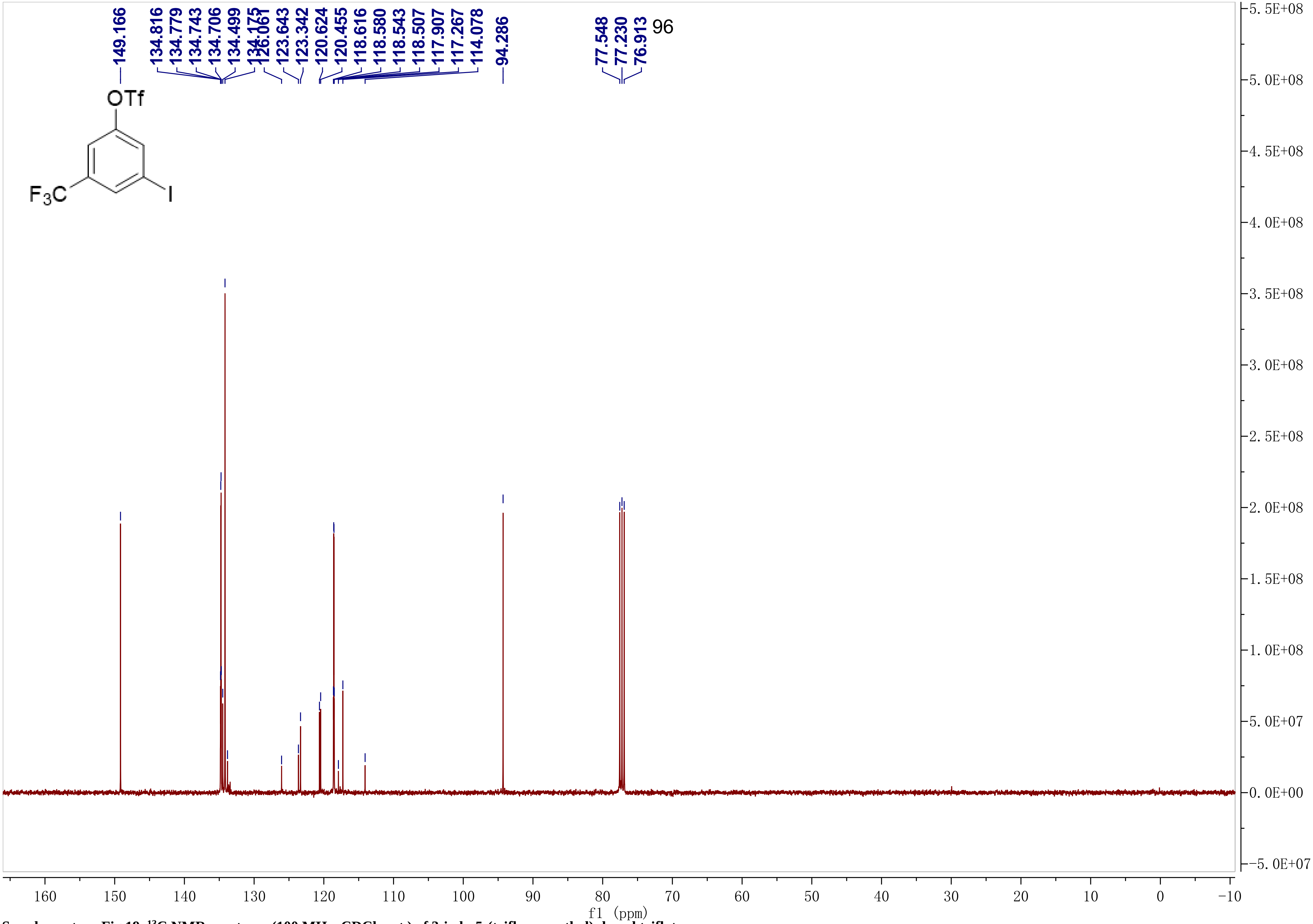

Supplementary Fig 19. ^{13}C NMR spectrum (100 MHz, $CDCl_3$, r.t.) of 3-iodo-5-(trifluoromethyl)phenyl triflate.

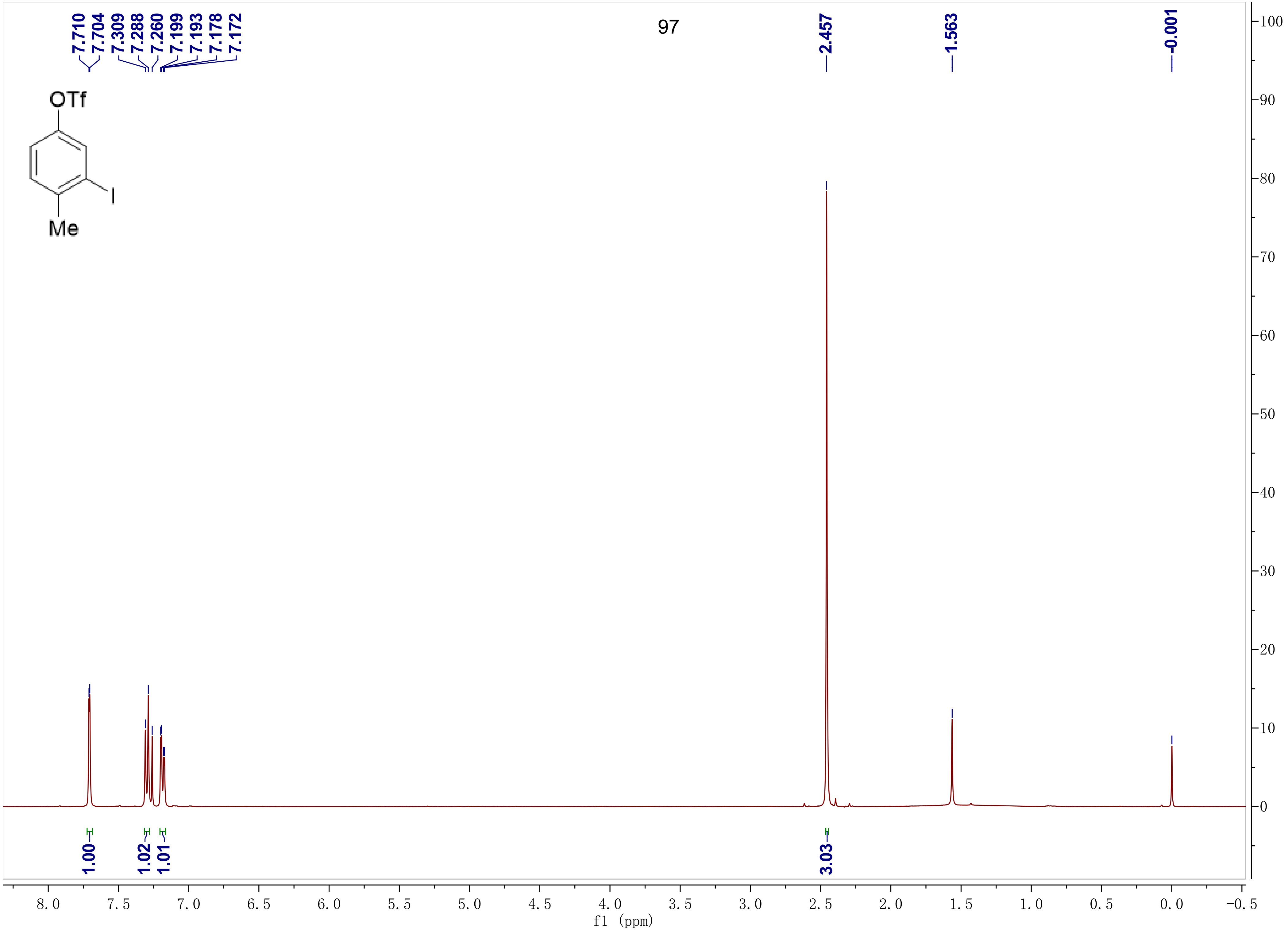

Supplementary Fig 20. ¹H NMR spectrum (400 MHz, $CDCl_3$, r.t.) of 3-iodo-4-methylphenyl triflate.

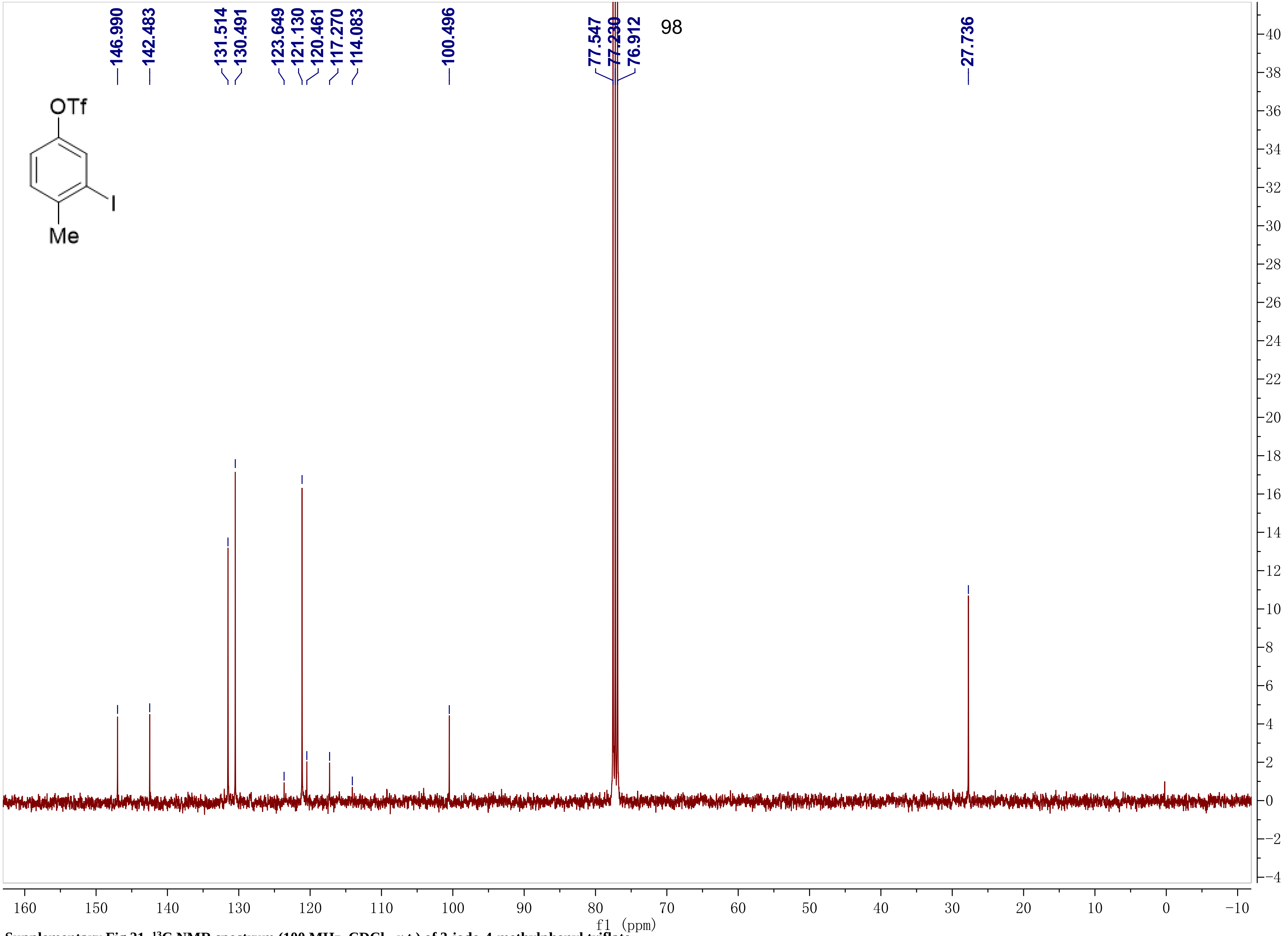

Supplementary Fig 21. ^{13}C NMR spectrum (100 MHz, CDCl$_3$, r.t.) of 3-iodo-4-methylphenyl triflate.

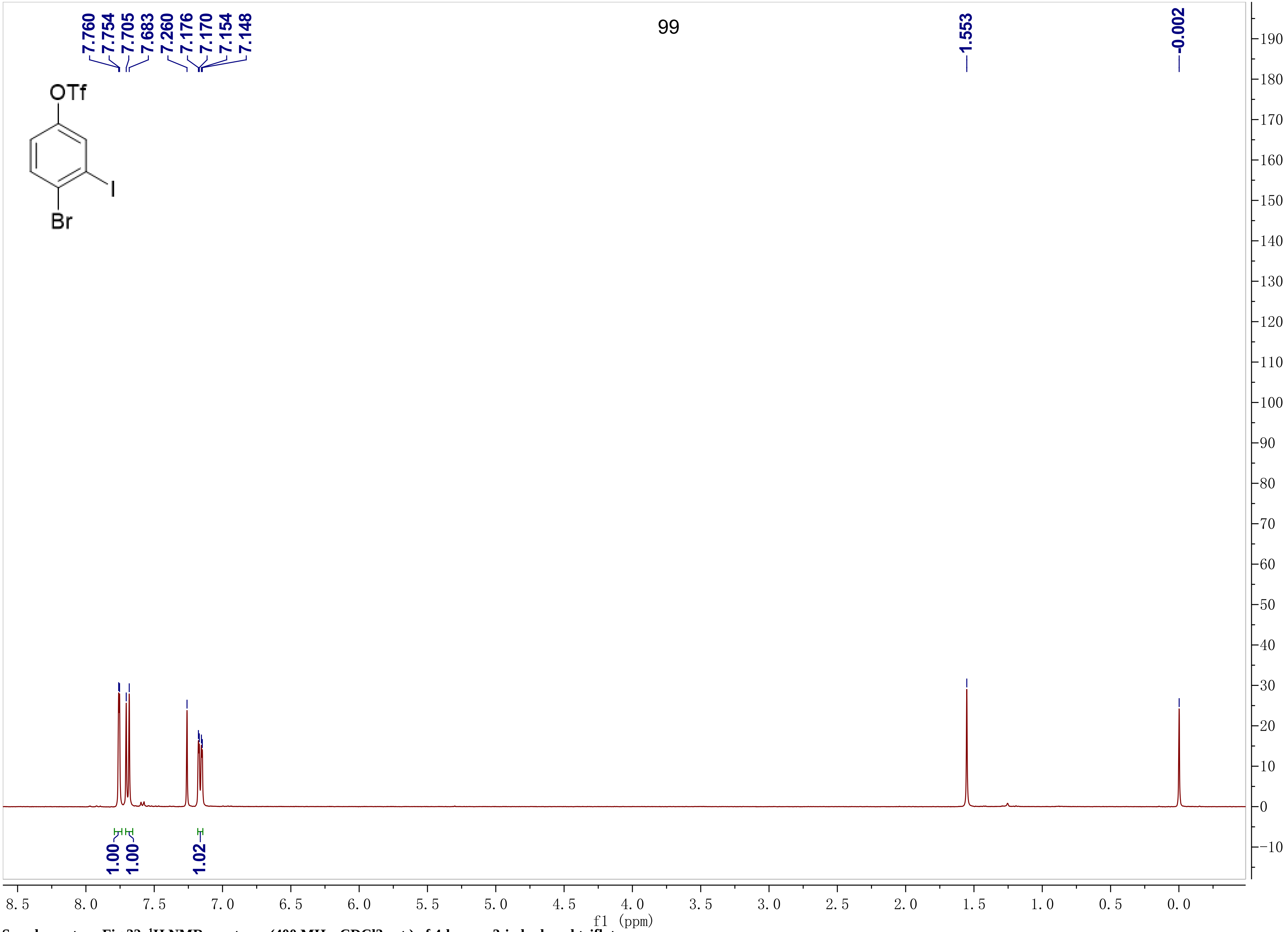

Supplementary Fig 22. ^{1}**H NMR spectrum (400 MHz, CDCl3, r.t.) of 4-bromo-3-iodophenyl triflate.**

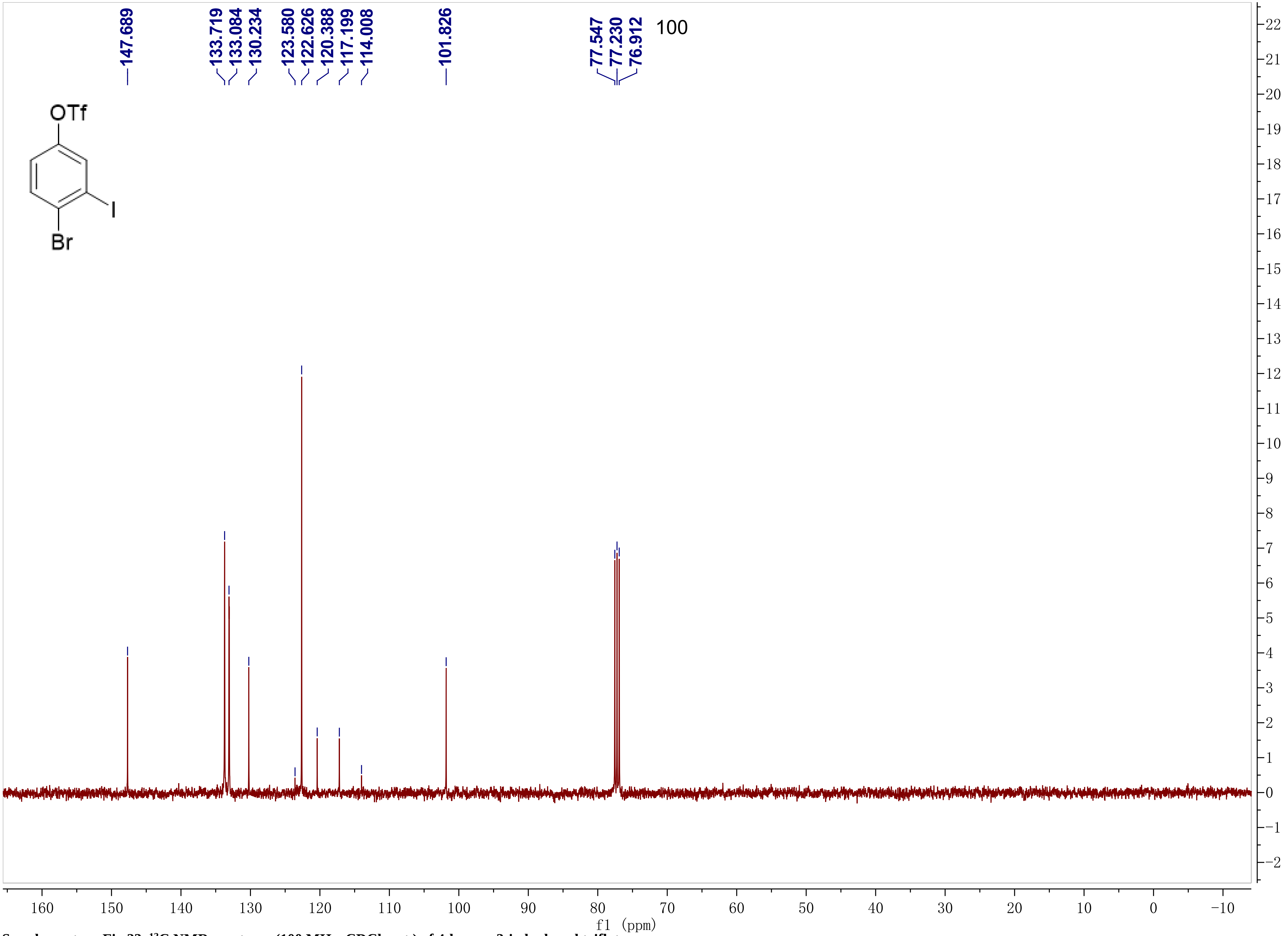

Supplementary Fig 23. ^{13}C NMR spectrum (100 MHz, $CDCl_3$, r.t.) of 4-bromo-3-iodophenyl triflate.

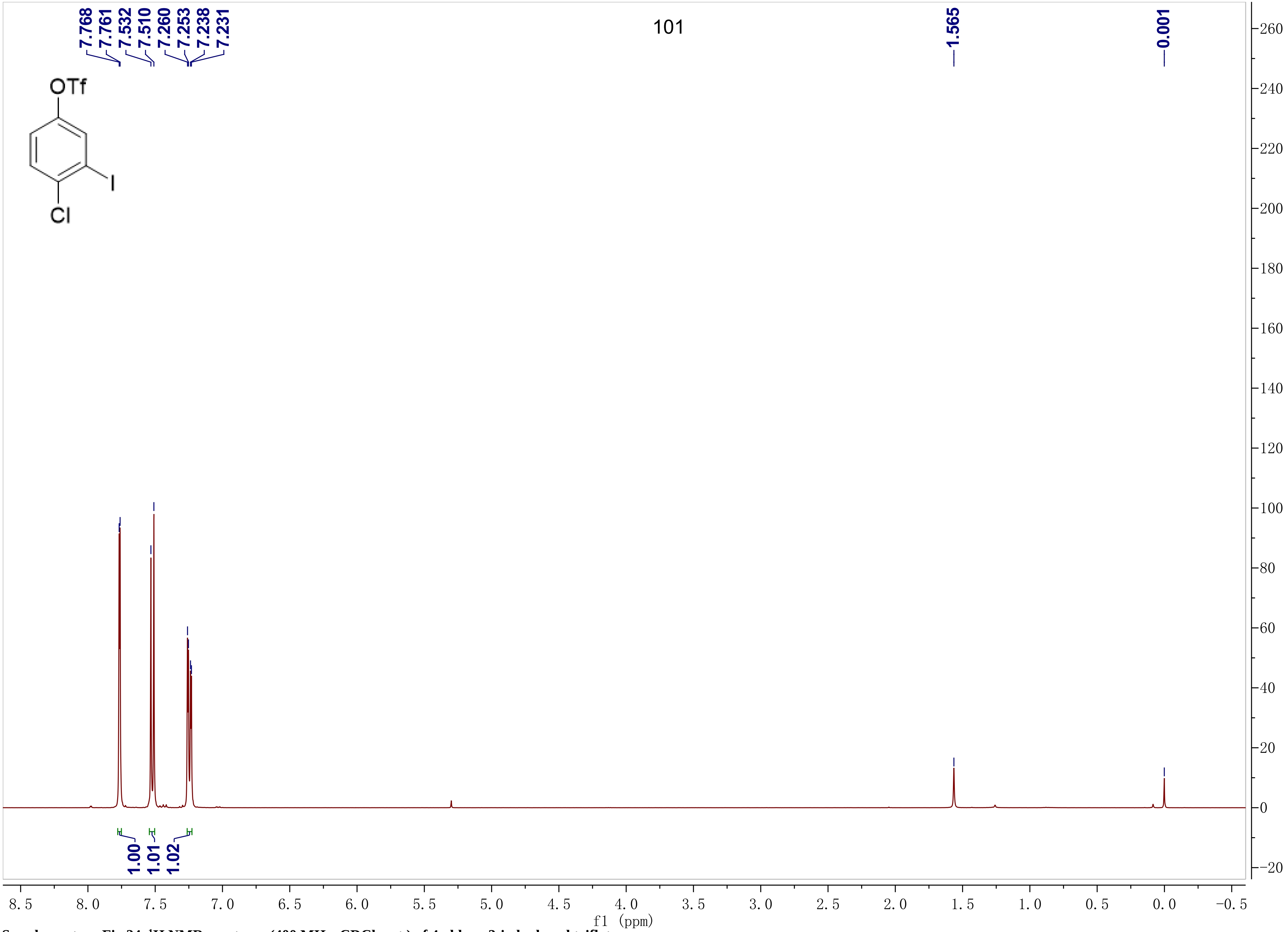

Supplementary Fig 24. ^{1}H NMR spectrum (400 MHz, $CDCl_3$, r.t.) of 4-chloro-3-iodophenyl triflate.

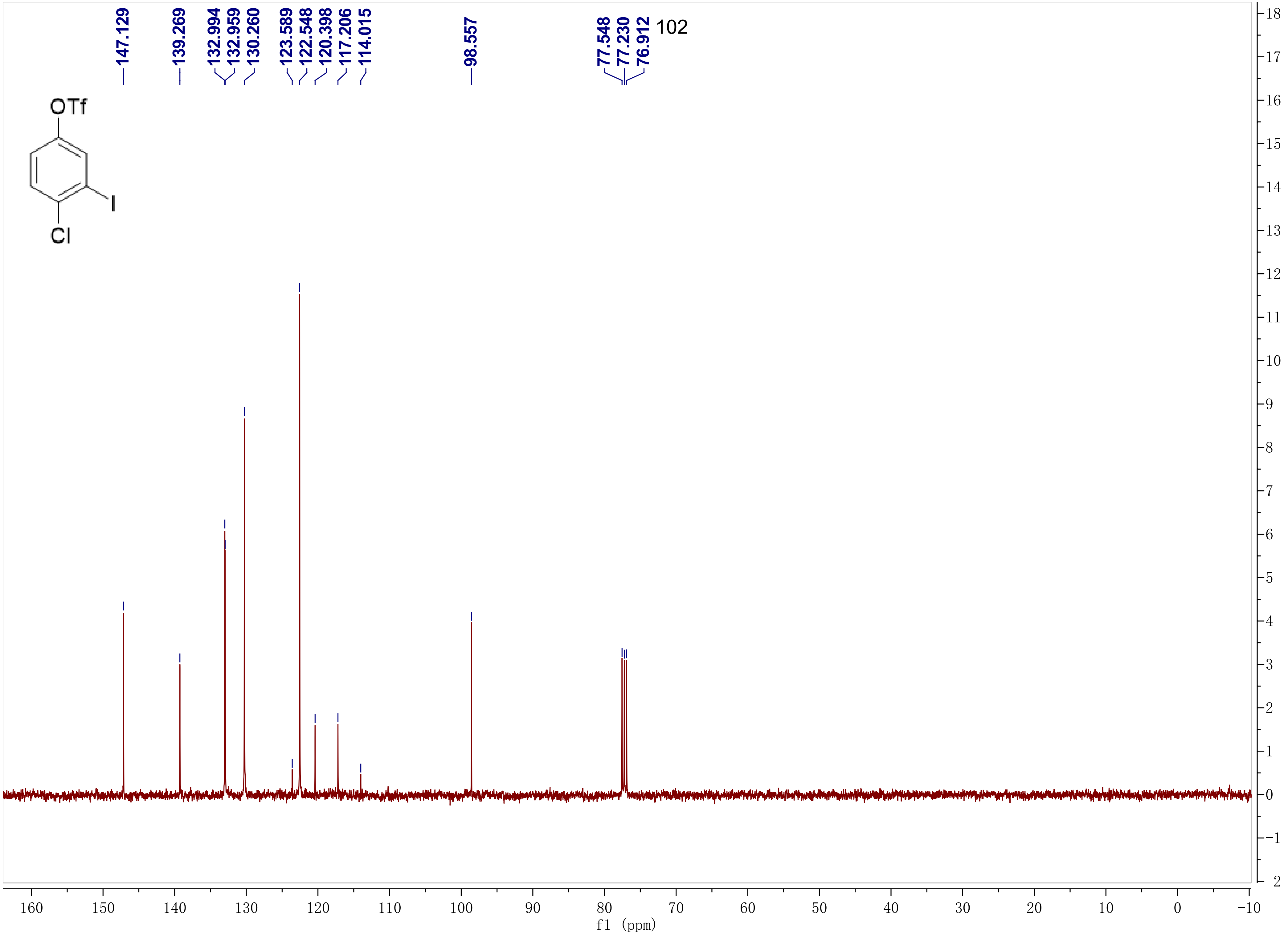

Supplementary Fig 25. ^{13}C NMR spectrum (100 MHz, $CDCl_3$, r.t.) of 4-chloro-3-iodophenyl triflate.

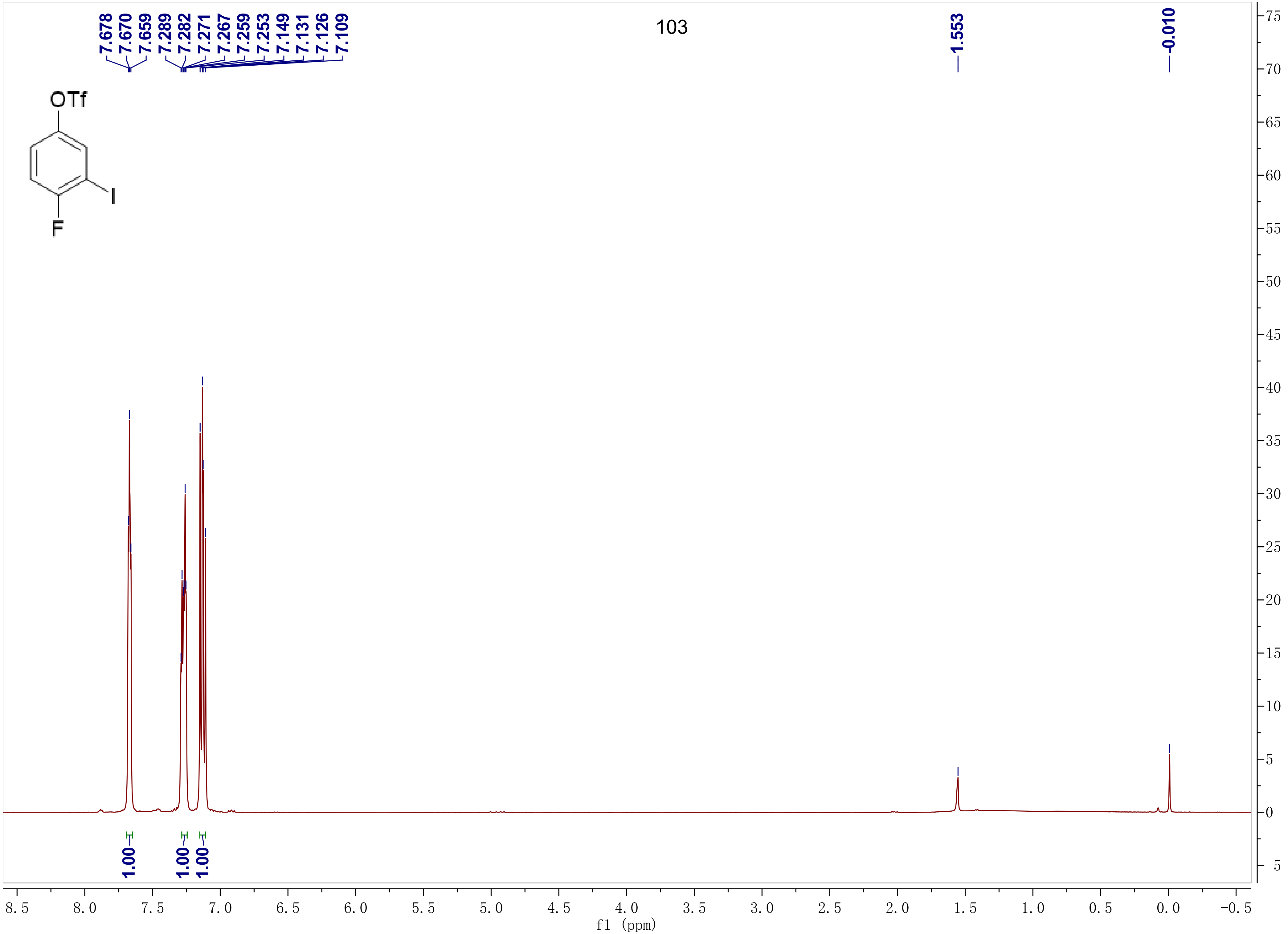

Supplementary Fig 26. ^{1}H **NMR spectrum (400 MHz,** $CDCl_3$**, r.t.) of 4-fluoro-3-iodophenyl triflate.**

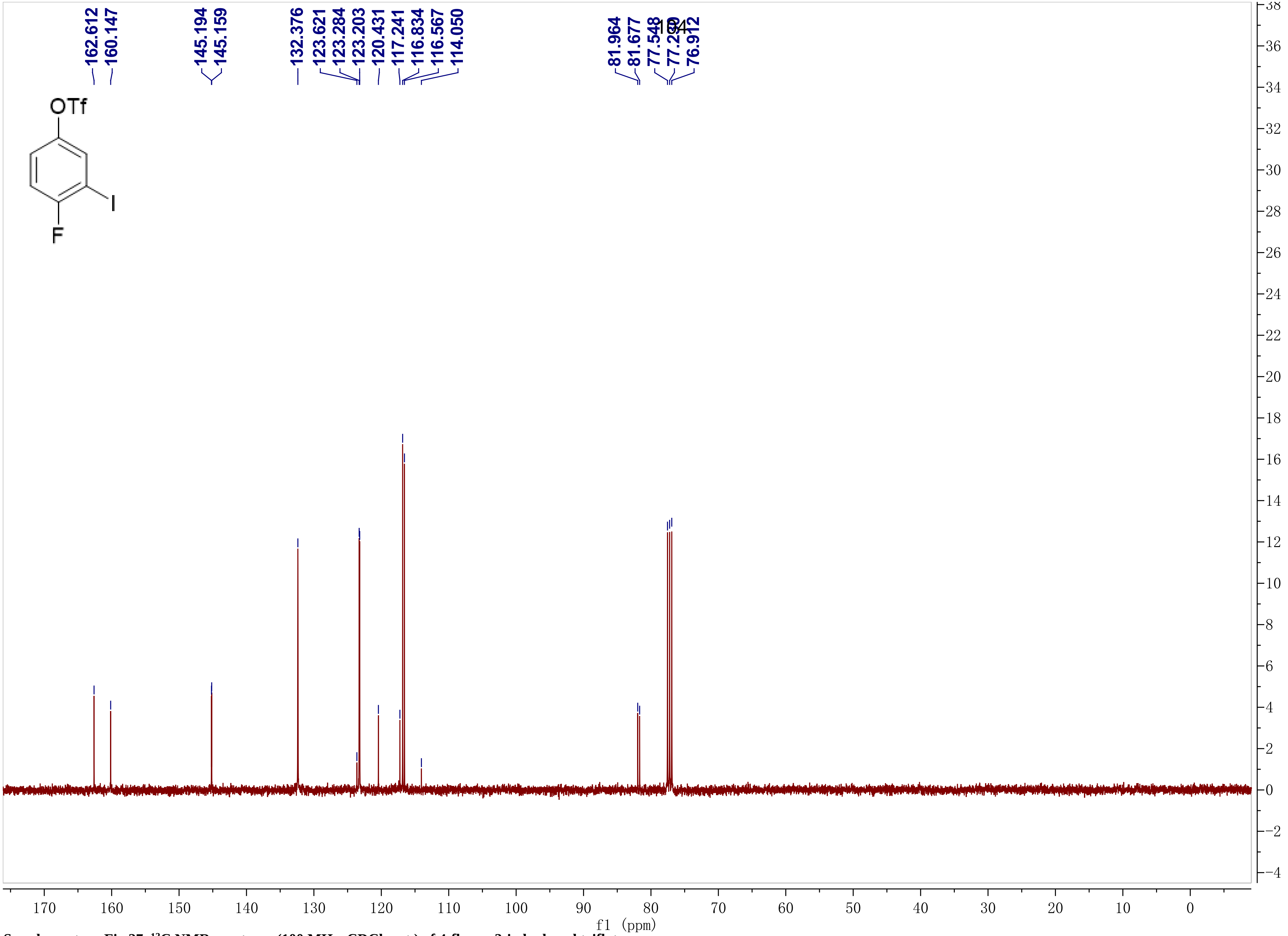

Supplementary Fig 27. ^{13}C NMR spectrum (100 MHz, $CDCl_3$, r.t.) of 4-fluoro-3-iodophenyl triflate.

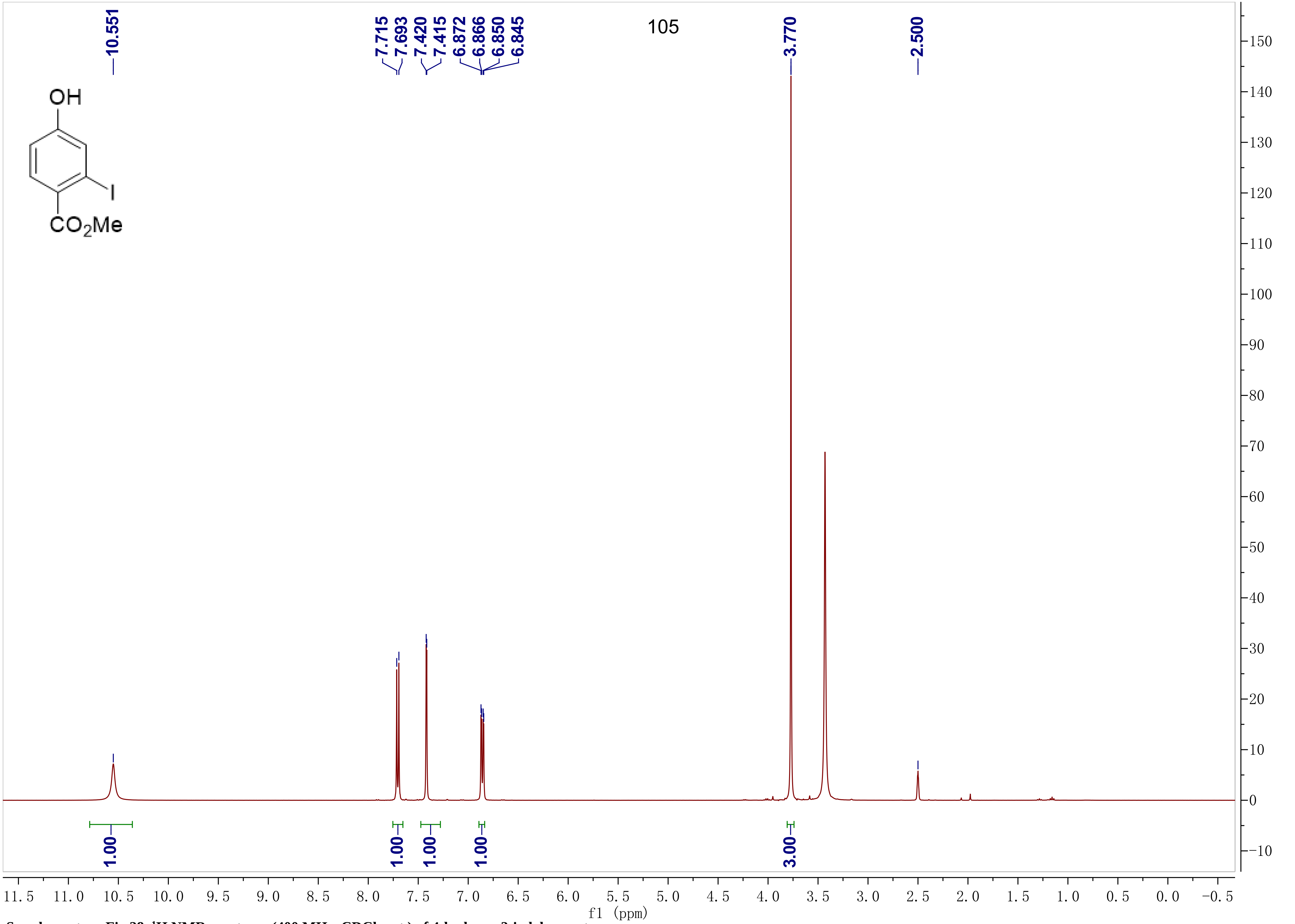

Supplementary Fig 28. ^1H NMR spectrum (400 MHz, $CDCl_3$, r.t.) of 4-hydroxy-2-iodobenzoate.

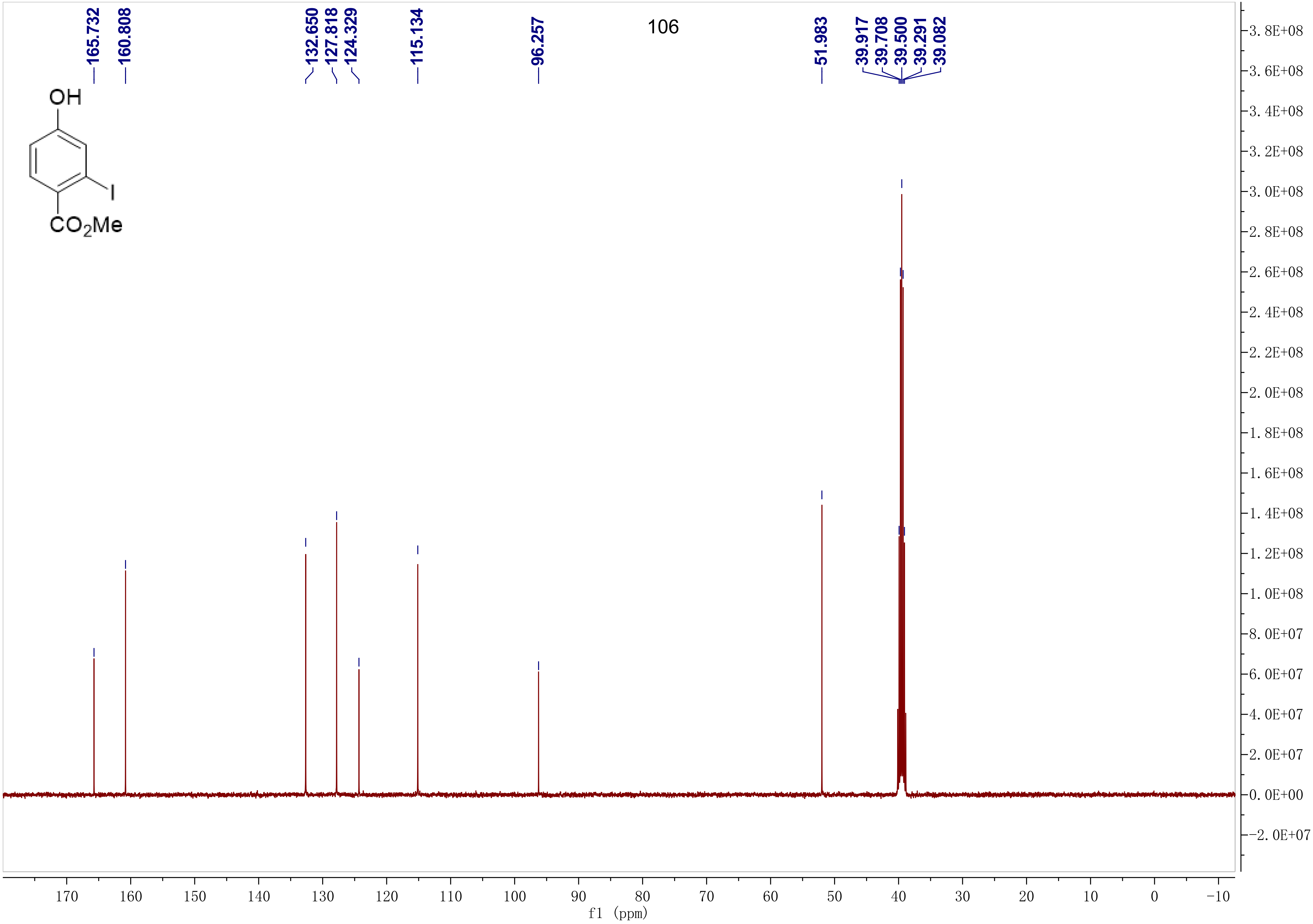

Supplementary Fig 29. **^{13}C NMR spectrum (100 MHz, $CDCl_3$, r.t.) of 4-hydroxy-2-iodobenzoate.**

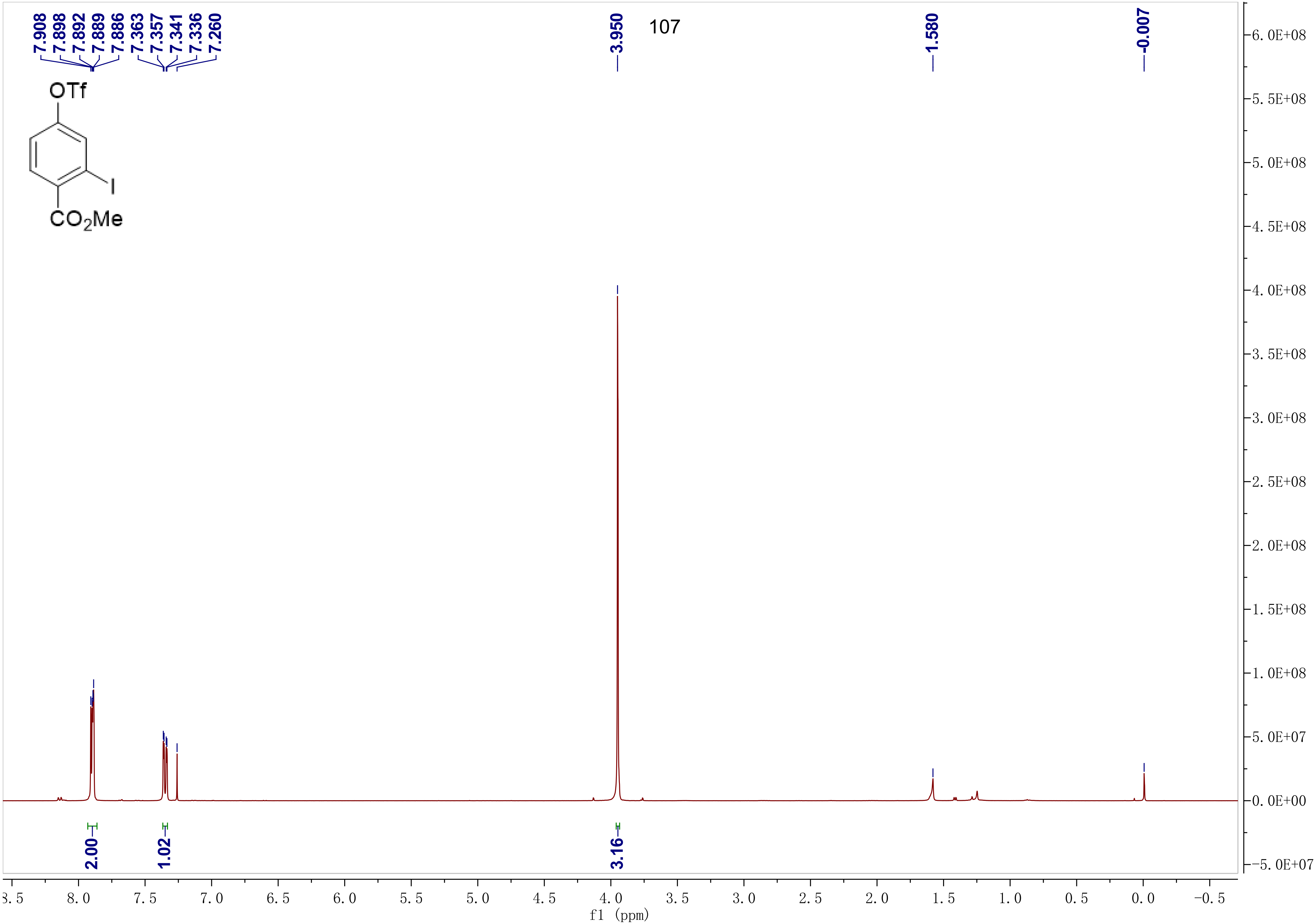

Supplementary Fig 30. ^{1}H NMR spectrum (400 MHz, $CDCl_3$, r.t.) of 2-iodo-4-(((trifluoromethyl)sulfonyl)oxy)benzoate.

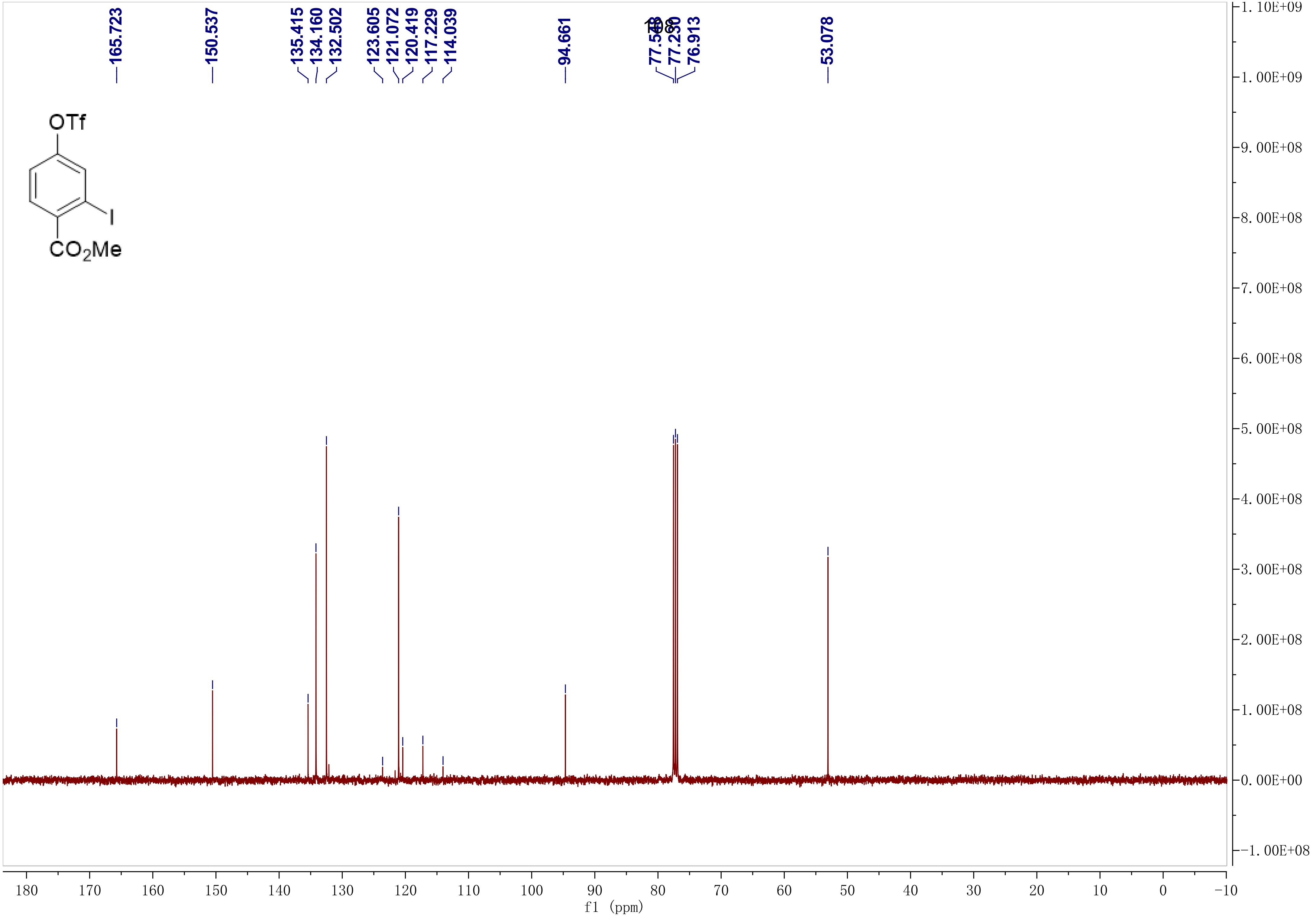

Supplementary Fig 31. ^{13}C NMR spectrum (100 MHz, $CDCl_3$, r.t.) of 2-iodo-4-(((trifluoromethyl)sulfonyl)oxy)benzoate.

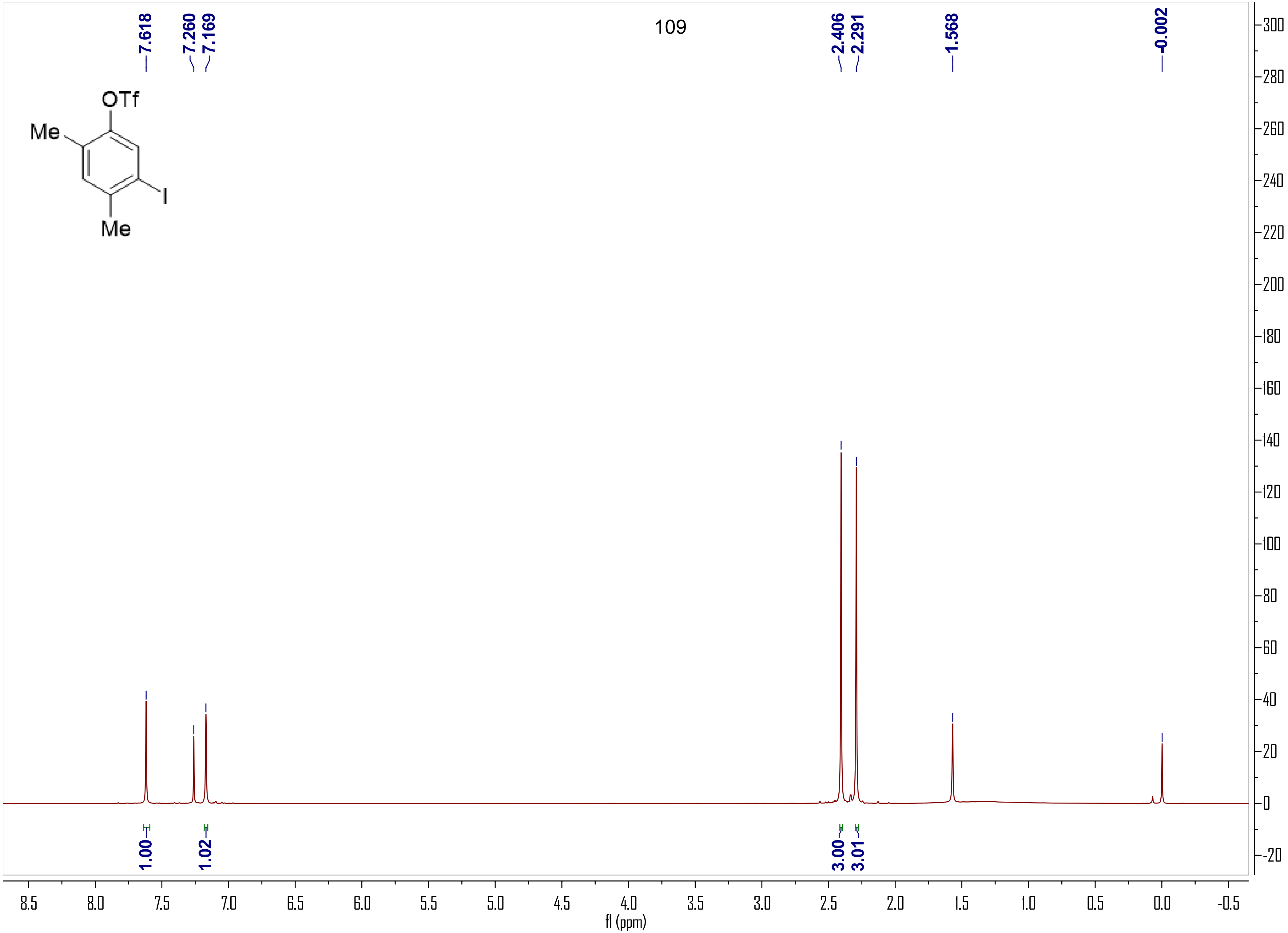

Supplementary Fig 32. ^{1}H NMR spectrum (400 MHz, CDCl3, r.t.) of 5-iodo-2,4-dimethylphenyl triflate.

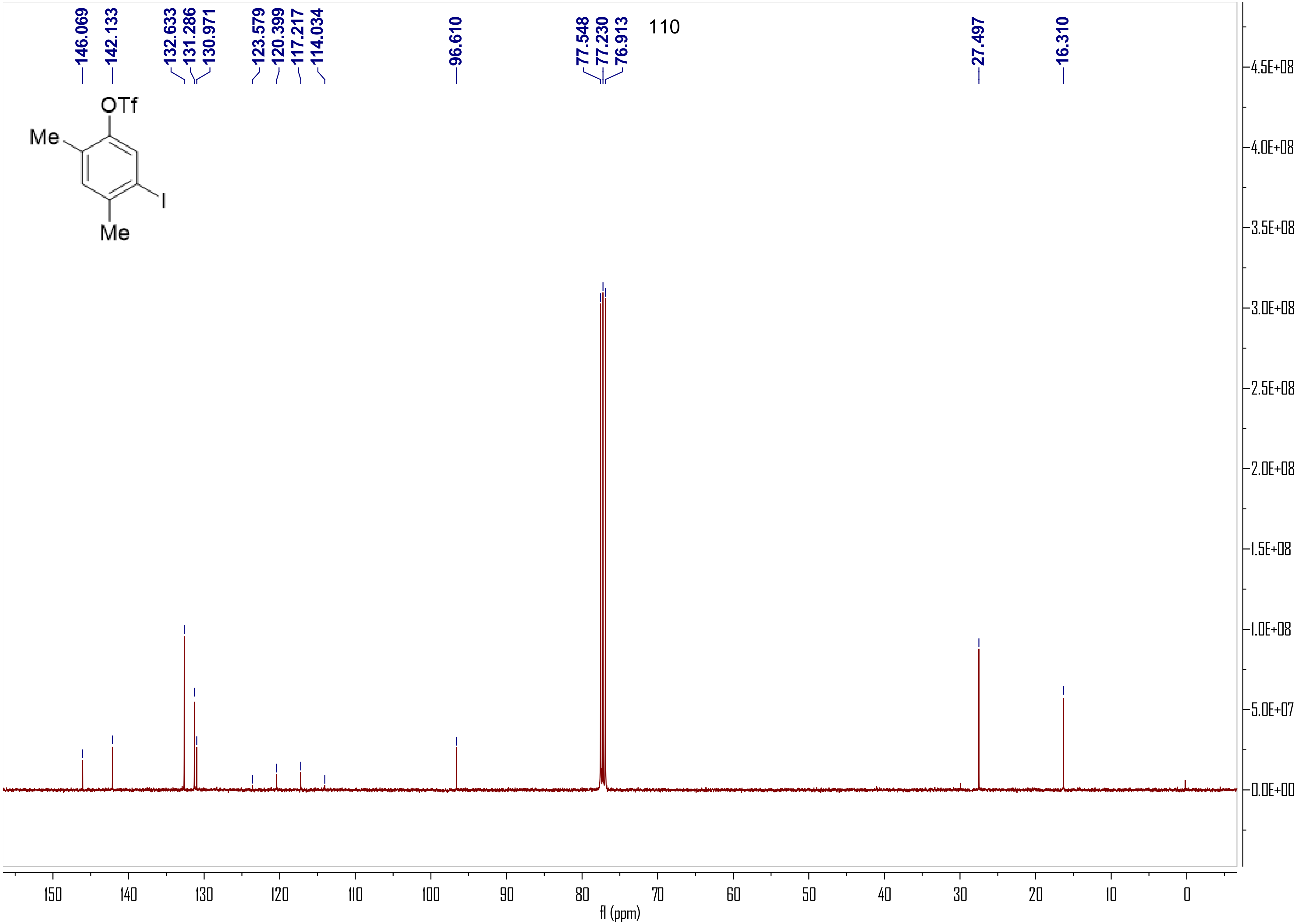

Supplementary Fig 33. ^{13}C NMR spectrum (100 MHz, $CDCl_3$, r.t.) of 5-iodo-2,4-dimethylphenyl triflate.

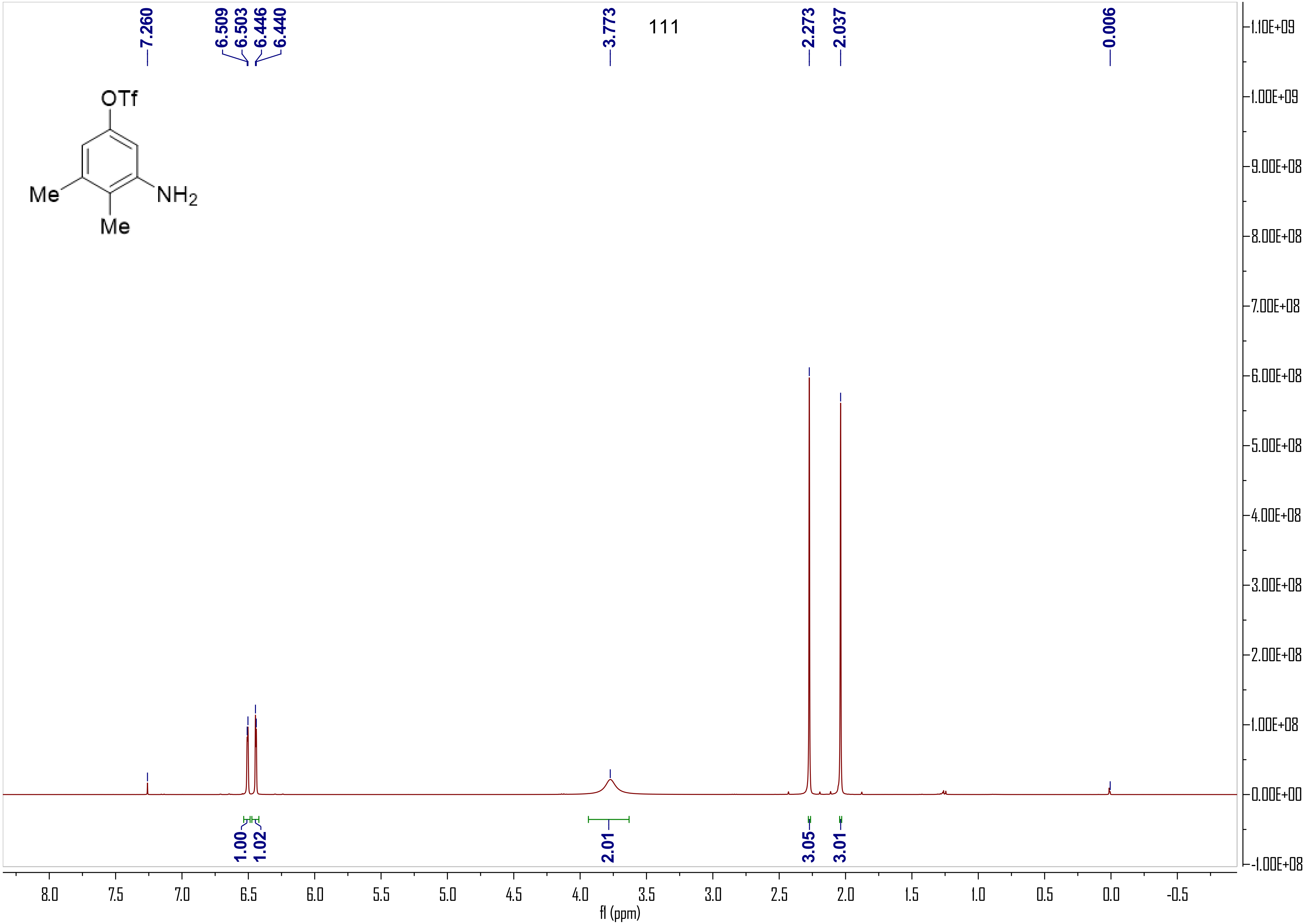

Supplementary Fig 34. **^{1}H NMR spectrum (400 MHz, $CDCl_3$, r.t.) of 3-amino-4,5-dimethylphenyl triflate.**

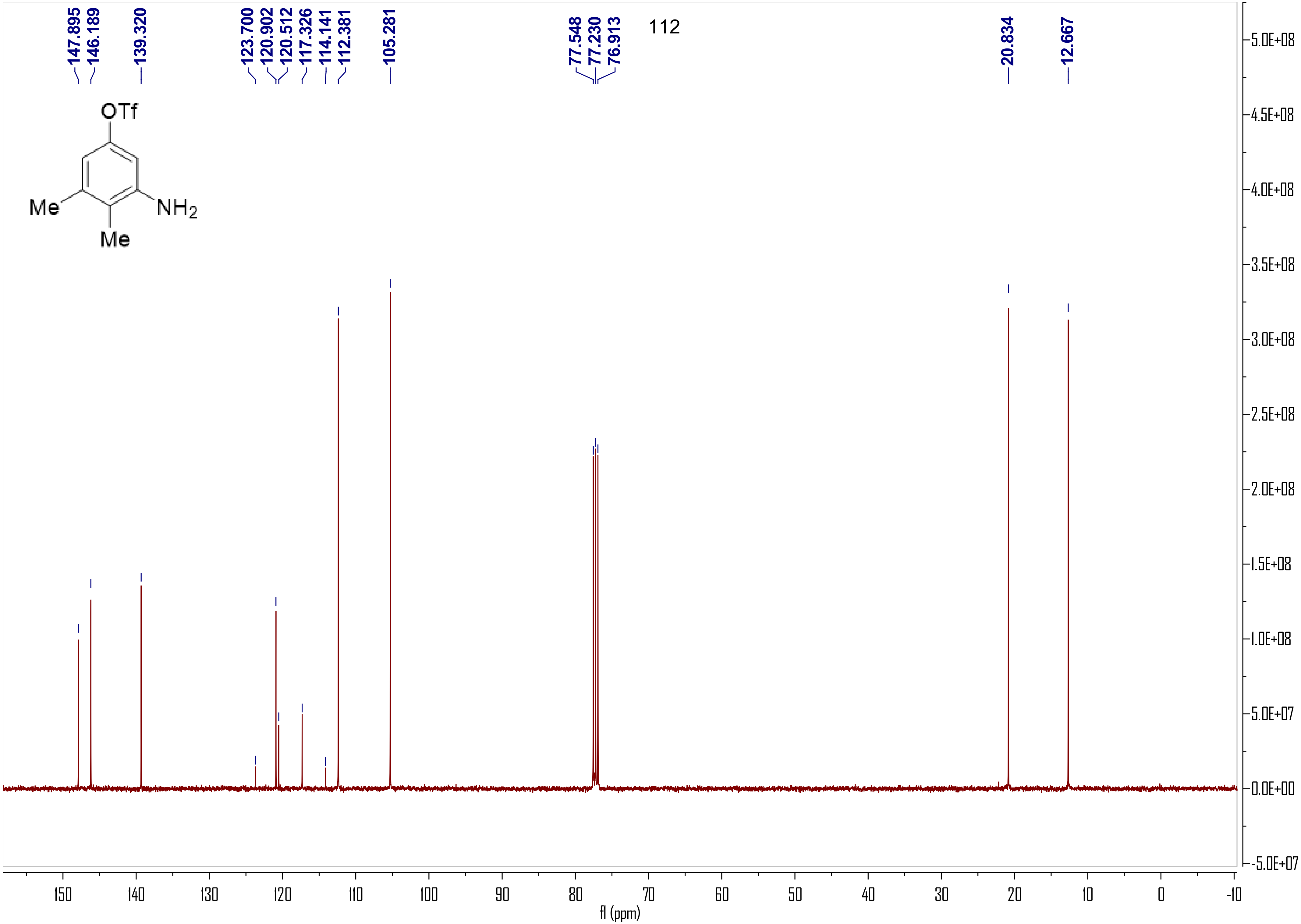

Supplementary Fig 35. ^{13}C **NMR spectrum (100 MHz, $CDCl_3$, r.t.) of 3-amino-4,5-dimethylphenyl triflate.**

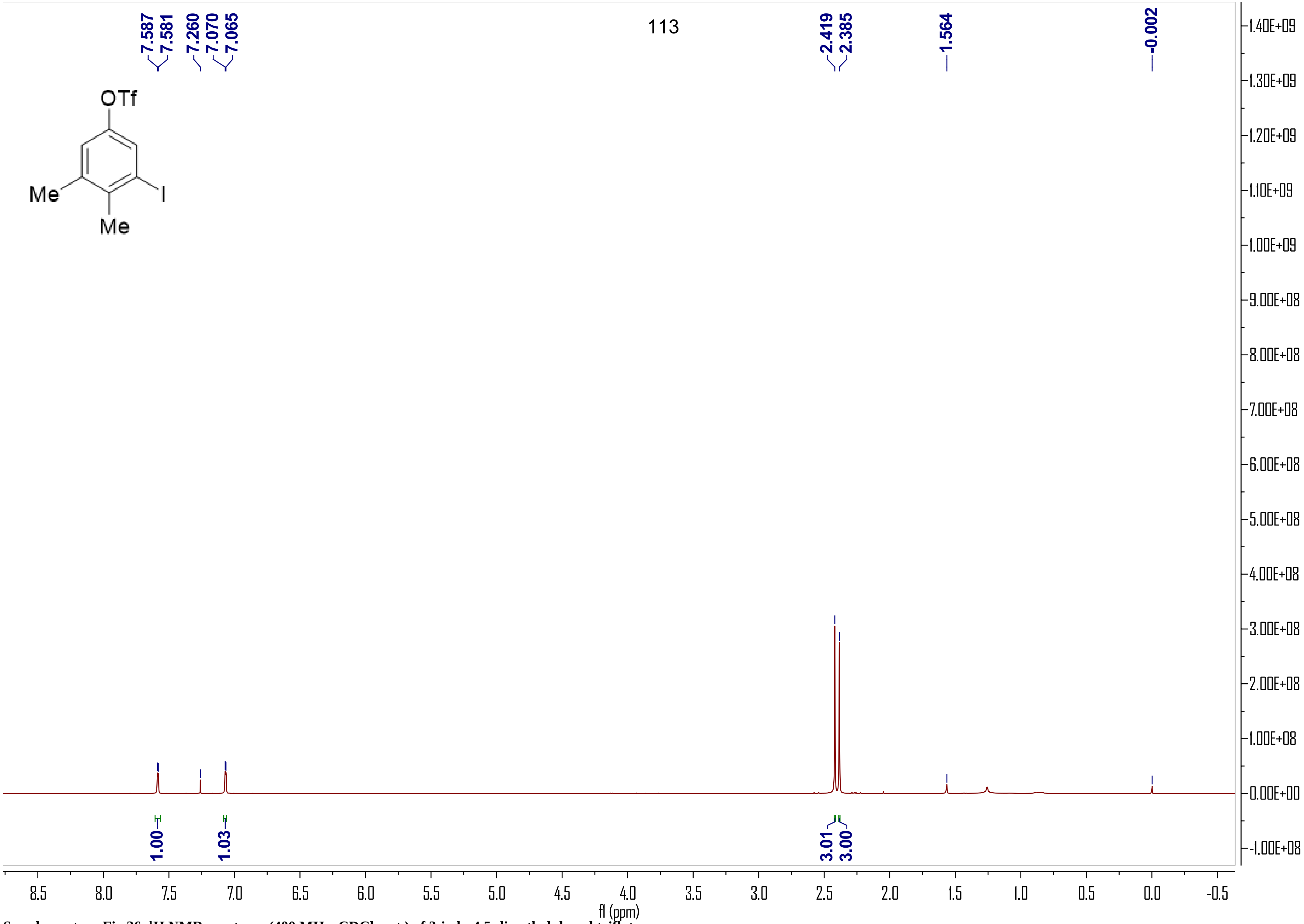

Supplementary Fig 36. ^{1}H NMR spectrum (400 MHz, $CDCl_3$, r.t.) of 3-iodo-4,5-dimethylphenyl triflate.

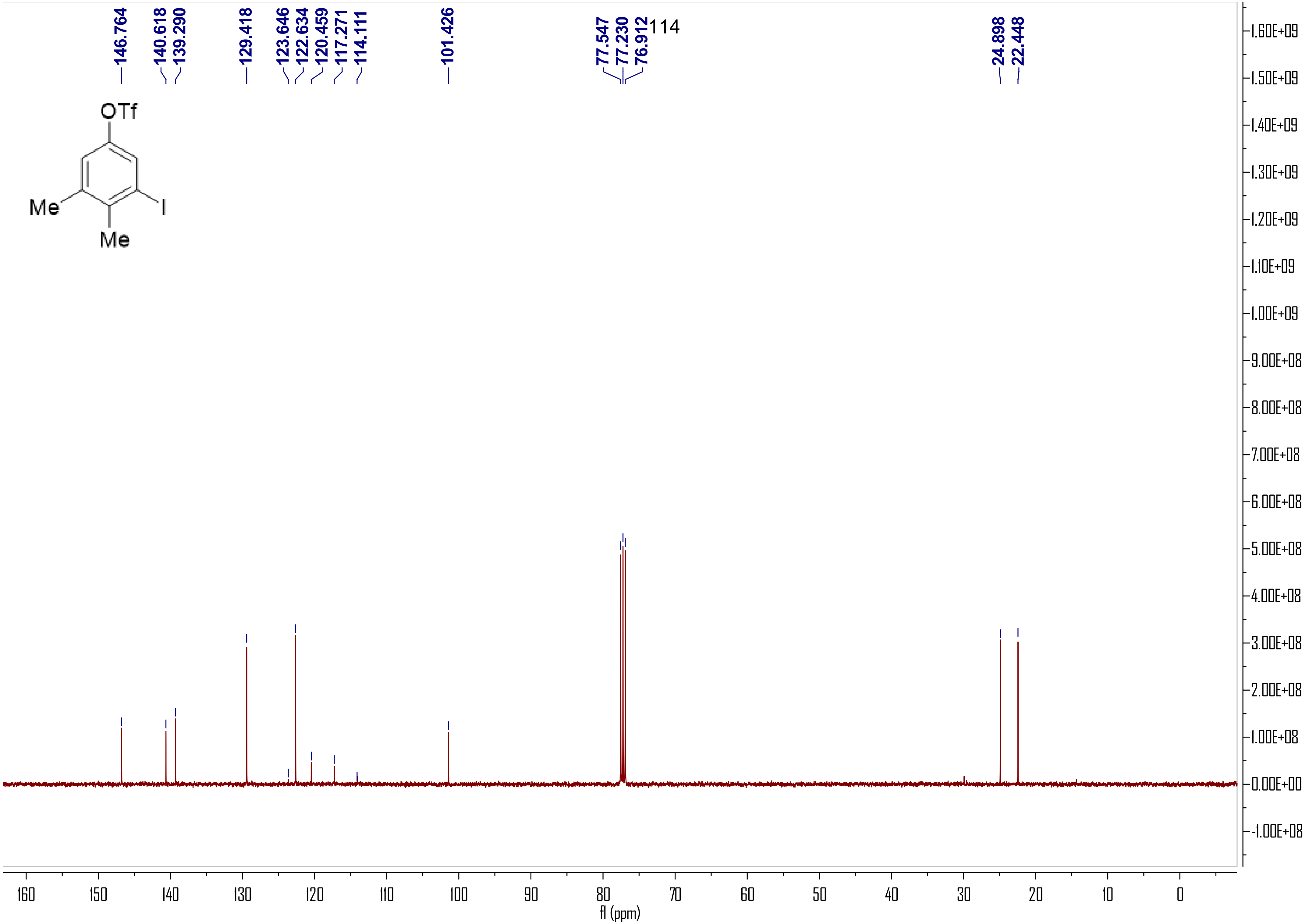

Supplementary Fig 37. ^{13}C **NMR spectrum (100 MHz,** $CDCl_3$**, r.t.) of 3-iodo-4,5-dimethylphenyl triflate.**

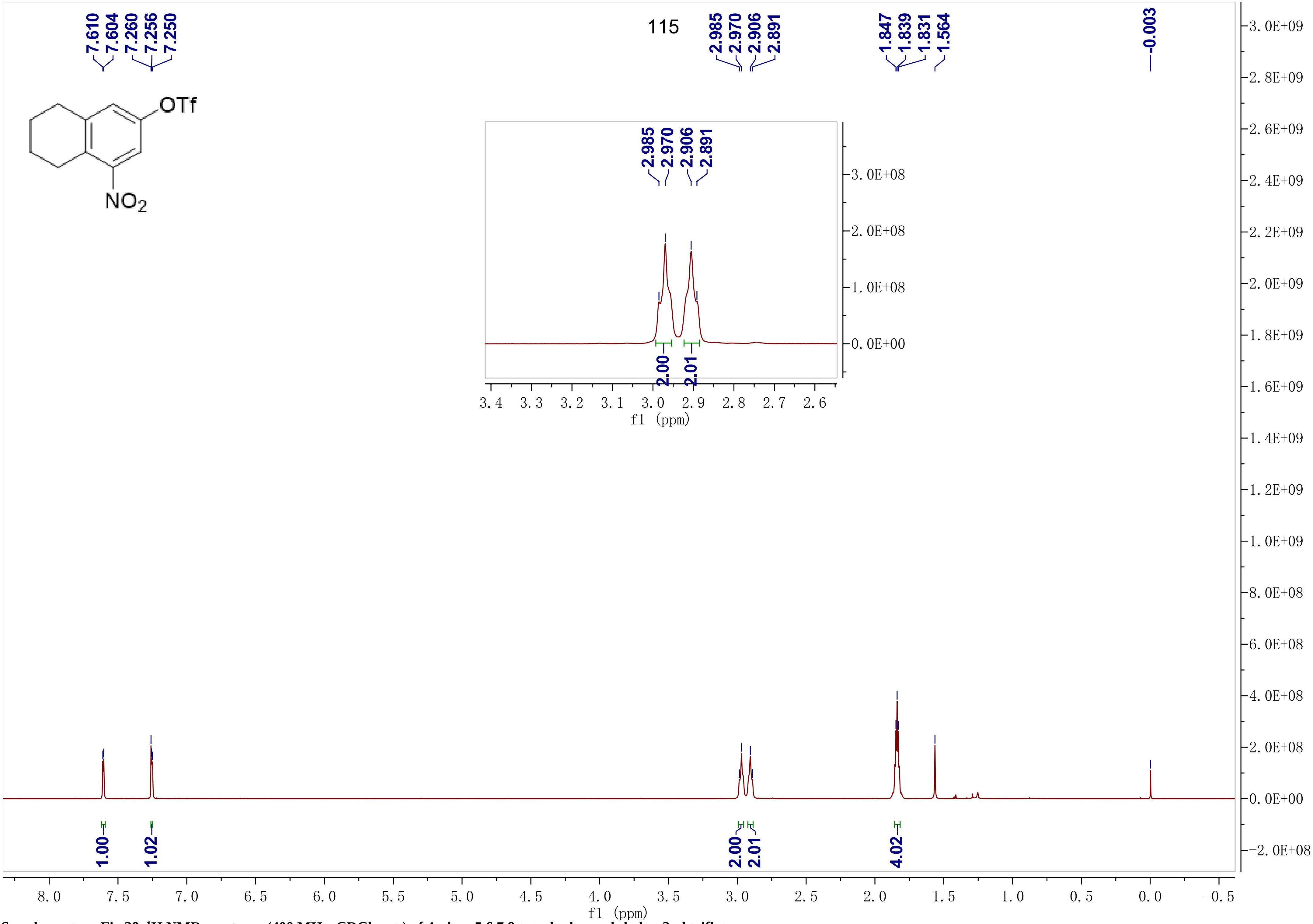

Supplementary Fig 38. **^{1}H NMR spectrum (400 MHz, $CDCl_3$, r.t.) of 4-nitro-5,6,7,8-tetrahydronaphthalen-2-yl triflate.**

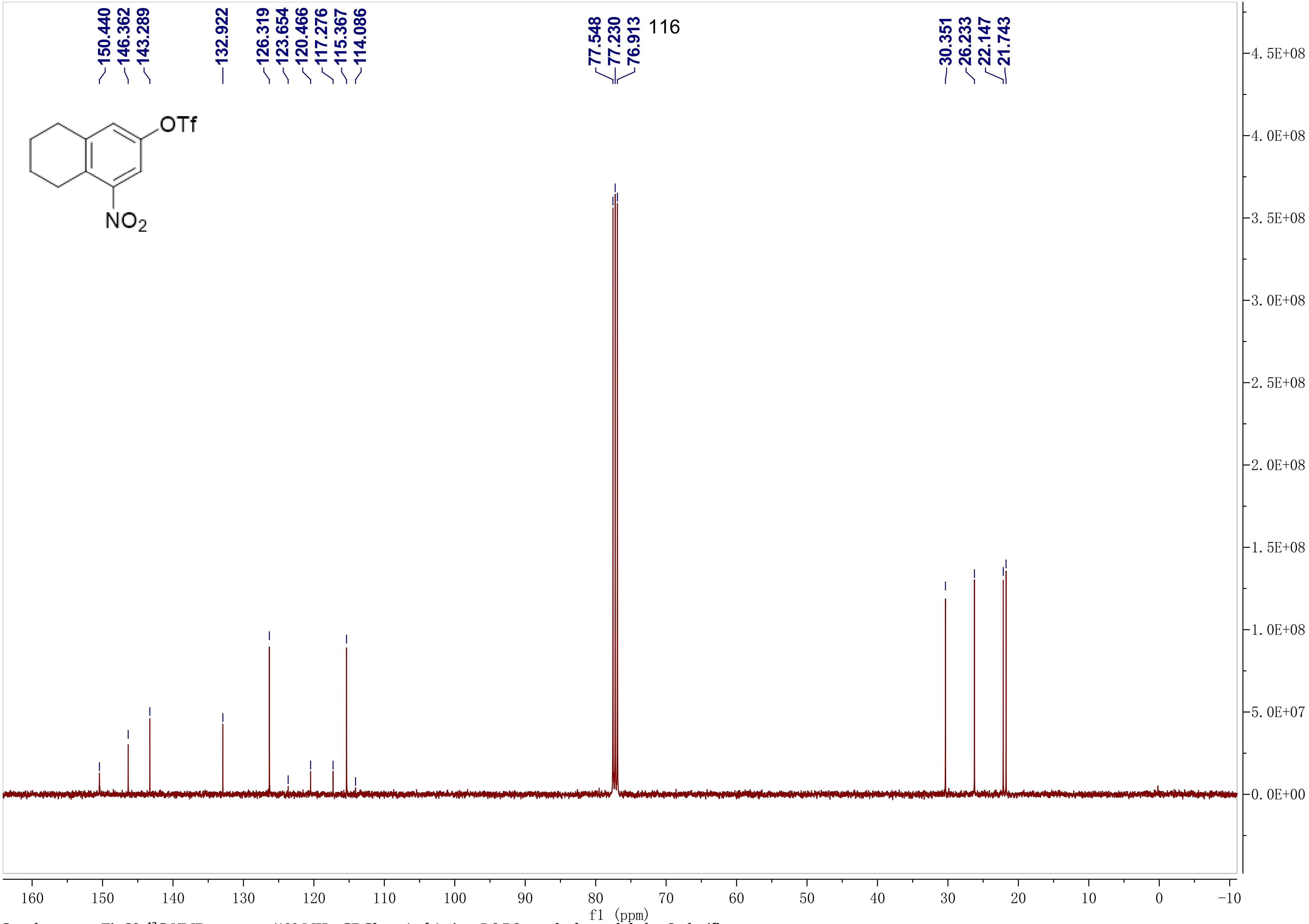

Supplementary Fig 39. ^{13}C NMR spectrum (100 MHz, $CDCl_3$, r.t.) of 4-nitro-5,6,7,8-tetrahydronaphthalen-2-yl triflate.

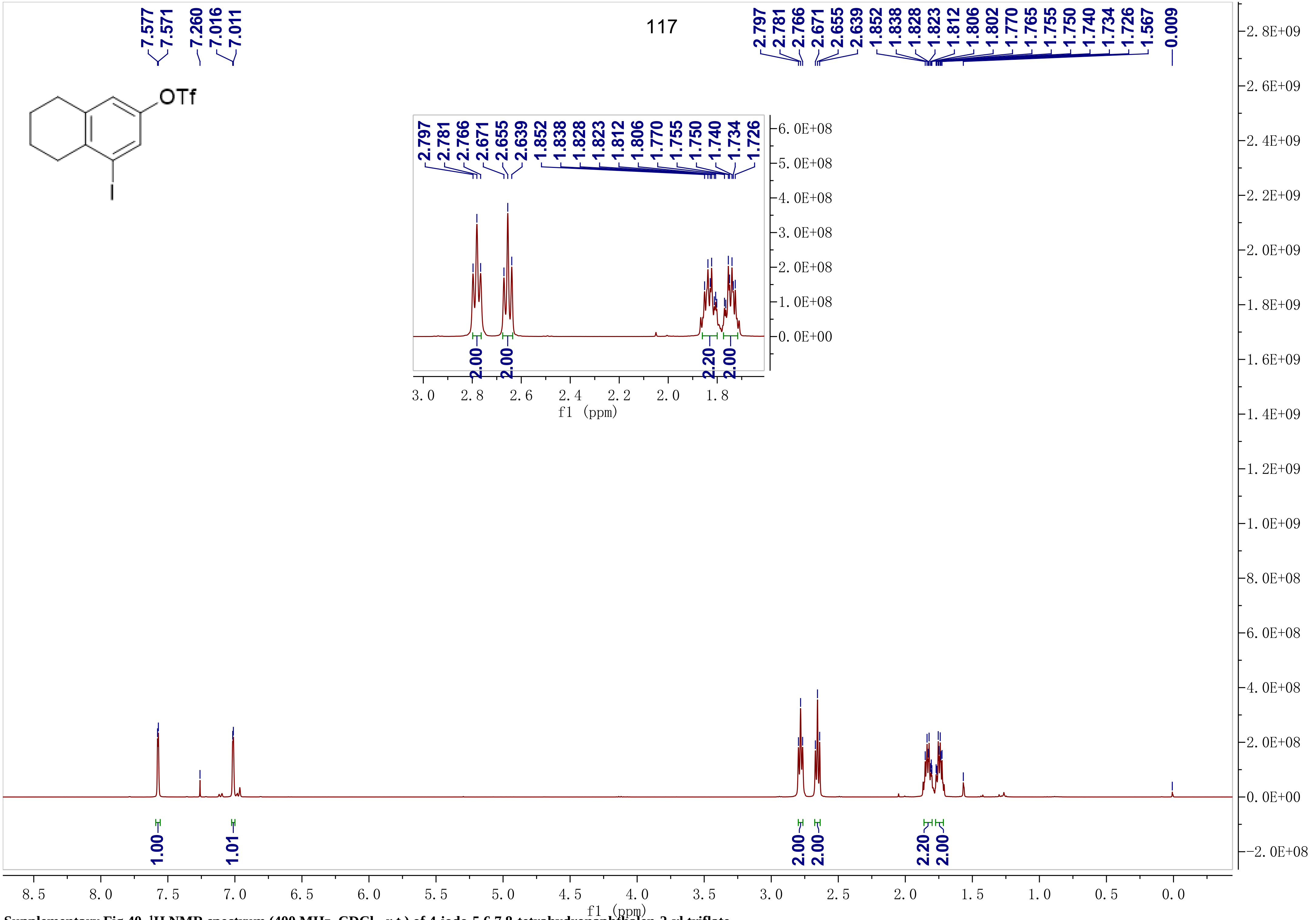

Supplementary Fig 40. **^{1}H NMR spectrum (400 MHz, $CDCl_3$, r.t.) of 4-iodo-5,6,7,8-tetrahydronaphthalen-2-yl triflate.**

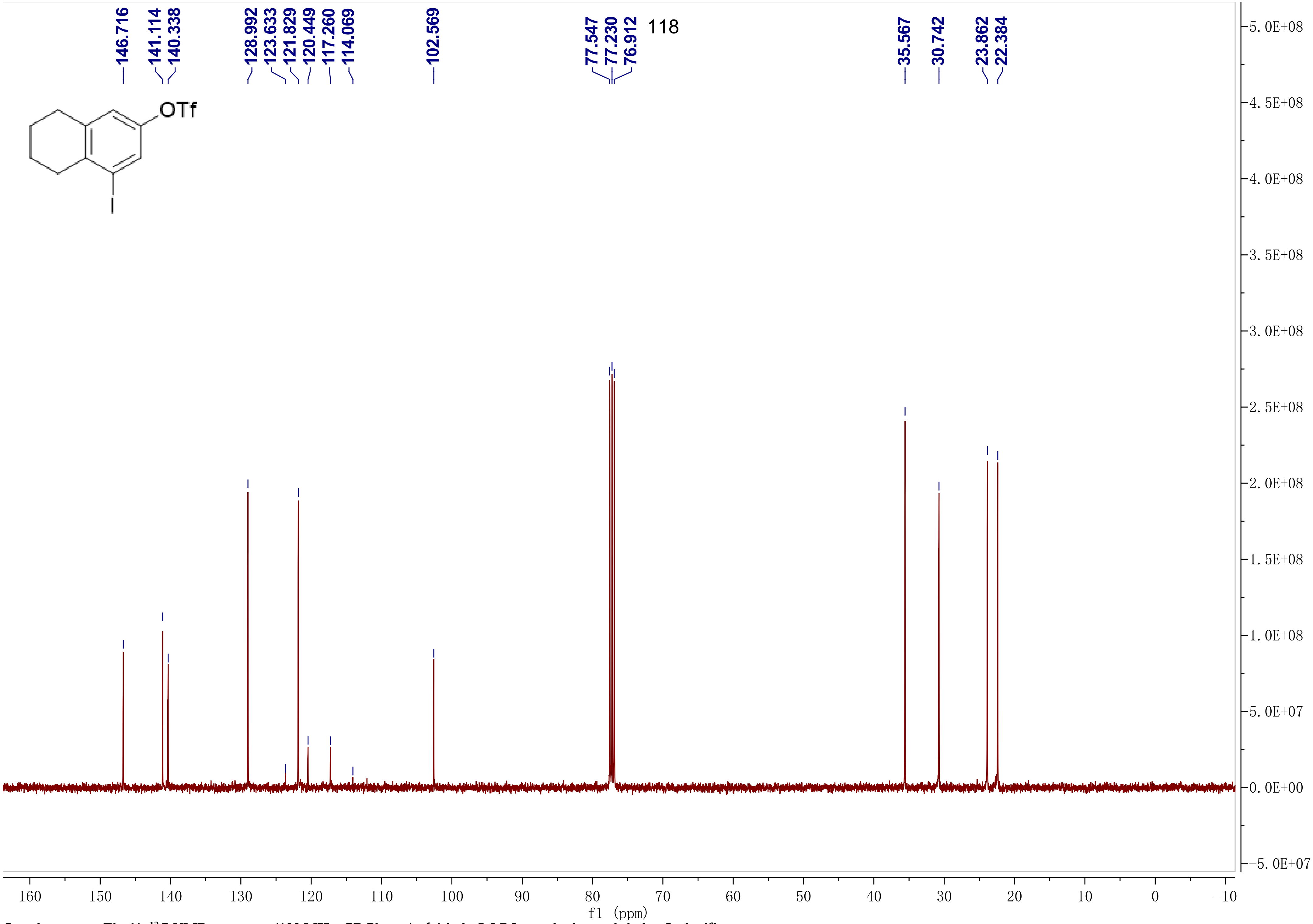

Supplementary Fig 41. ^{13}C NMR spectrum (100 MHz, $CDCl_3$, r.t.) of 4-iodo-5,6,7,8-tetrahydronaphthalen-2-yl triflate.

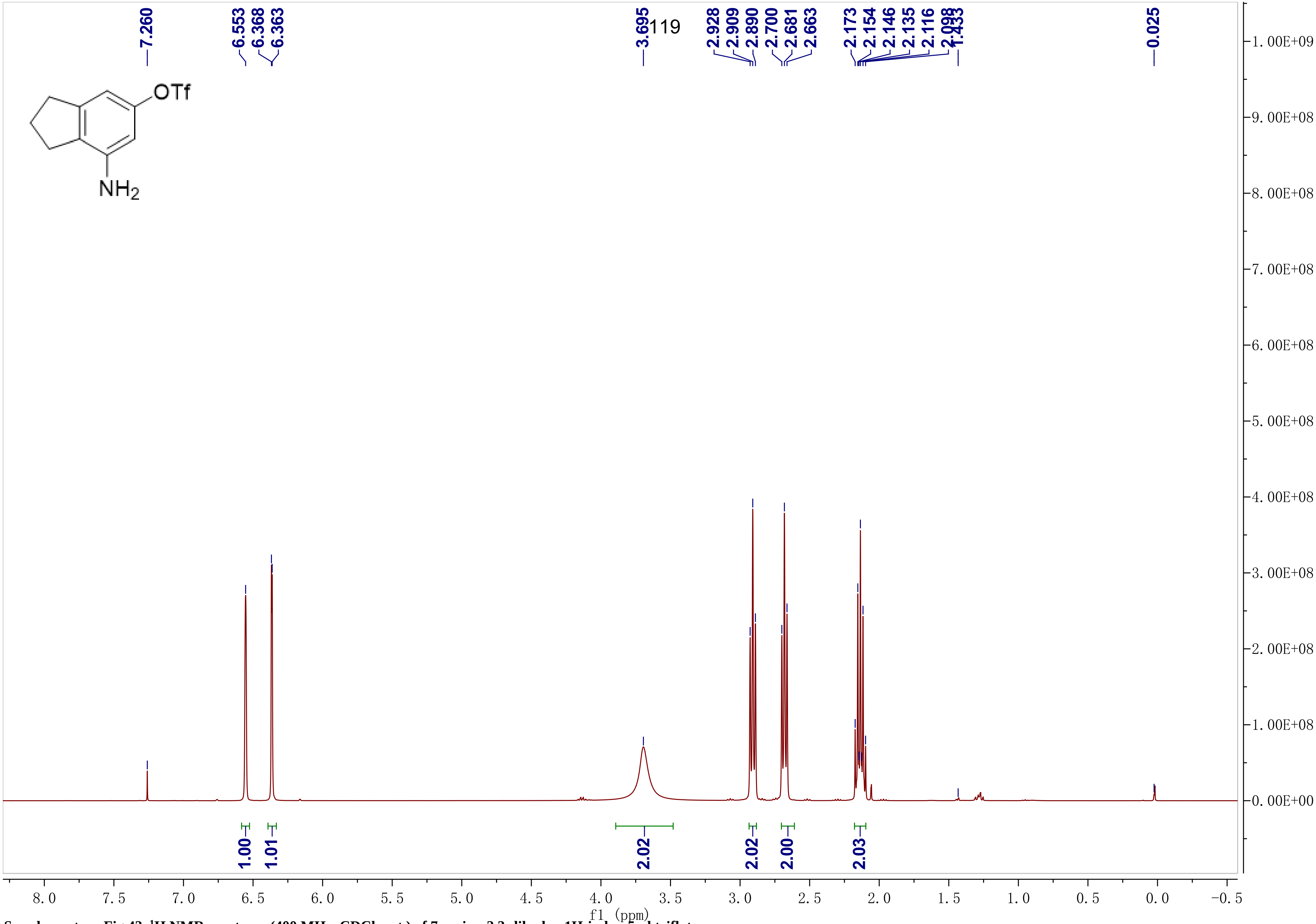

Supplementary Fig 42. ¹H NMR spectrum (400 MHz, $CDCl_3$, r.t.) of 7-amino-2,3-dihydro-1H-inden-5-yl triflate.

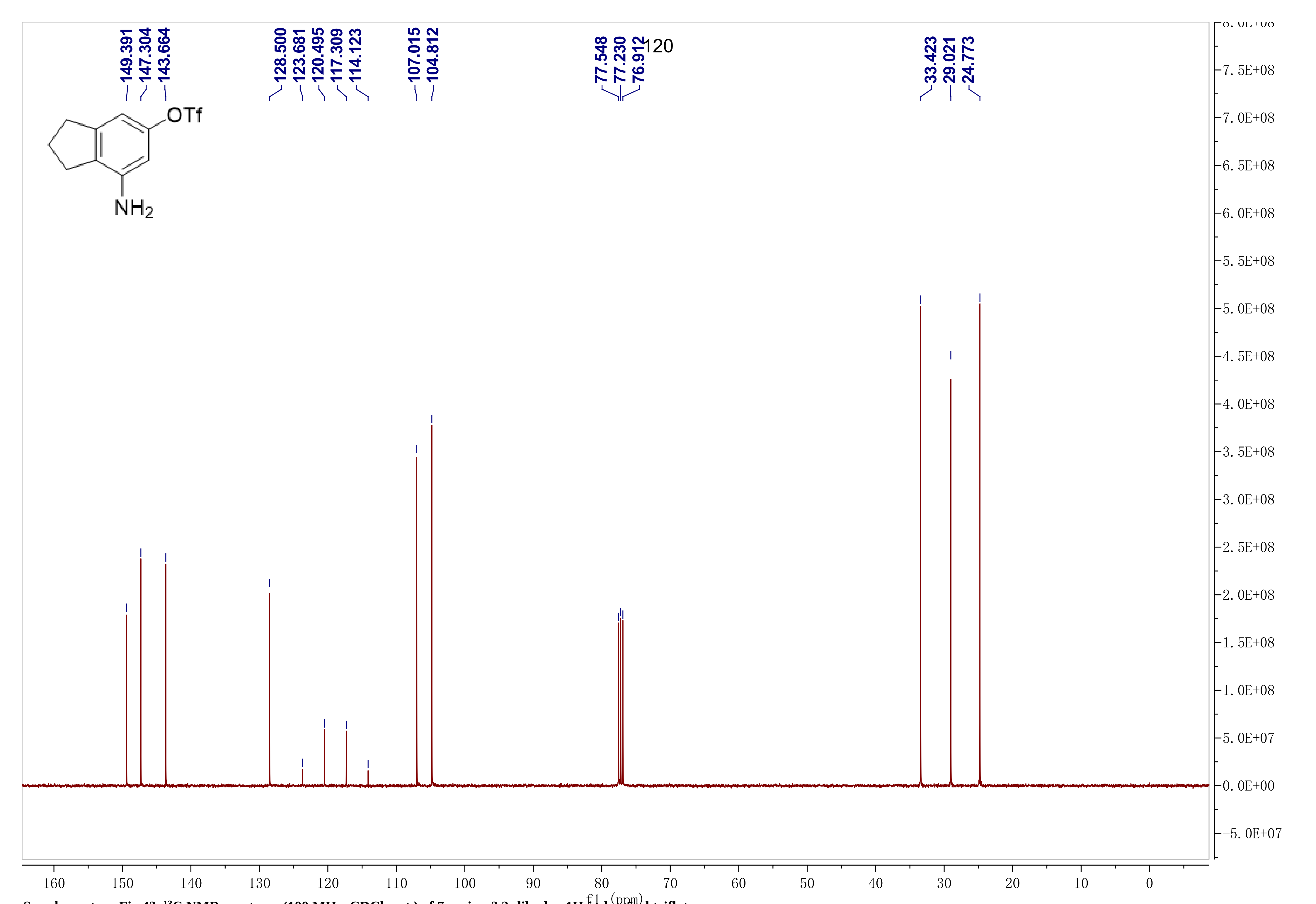

Supplementary Fig 43. ^{13}C NMR spectrum (100 MHz, $CDCl_3$, r.t.) of 7-amino-2,3-dihydro-1H-inden-5-yl triflate.

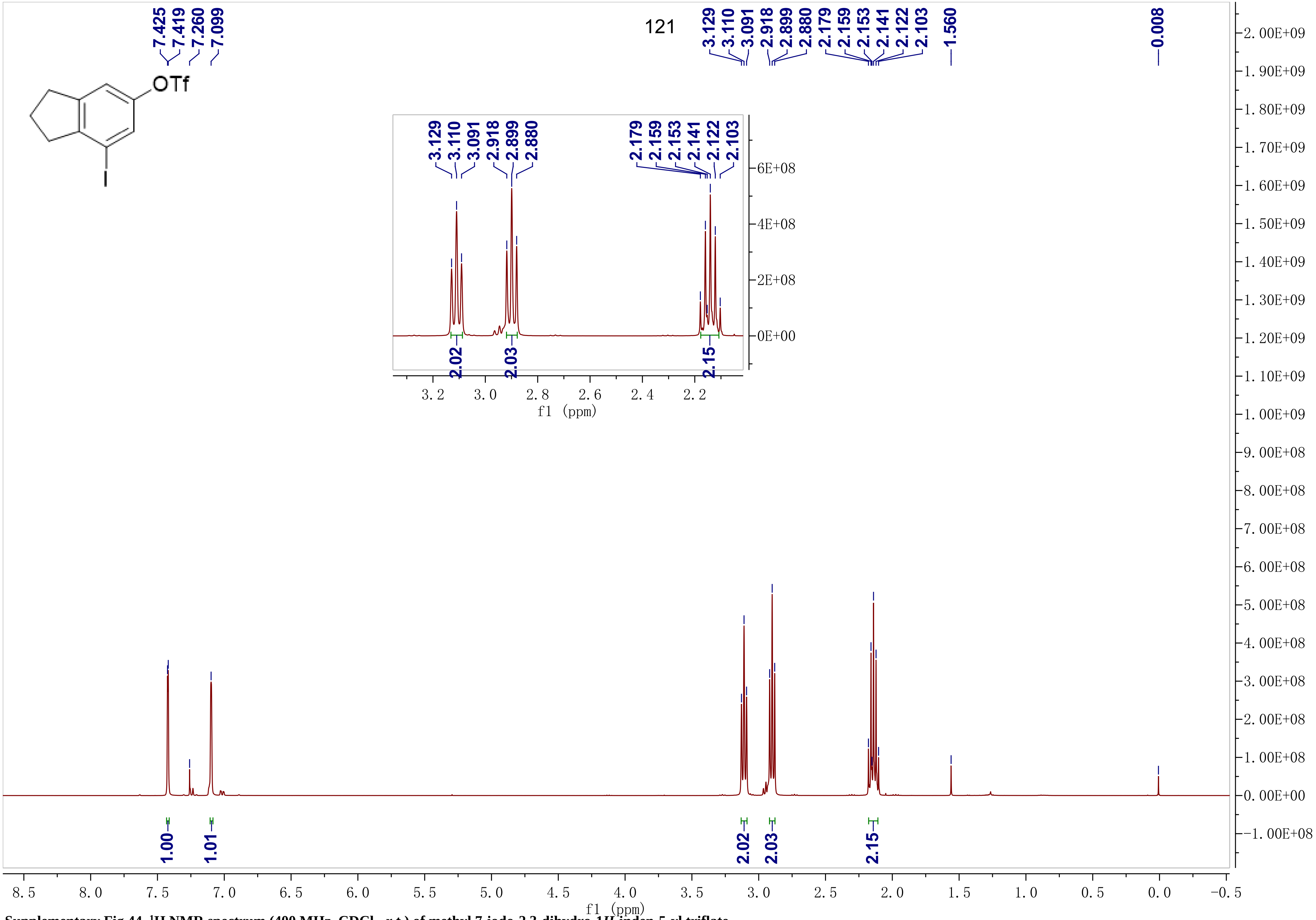

Supplementary Fig 44. **^{1}H NMR spectrum (400 MHz, $CDCl_3$, r.t.) of methyl 7-iodo-2,3-dihydro-1*H*-inden-5-yl triflate.**

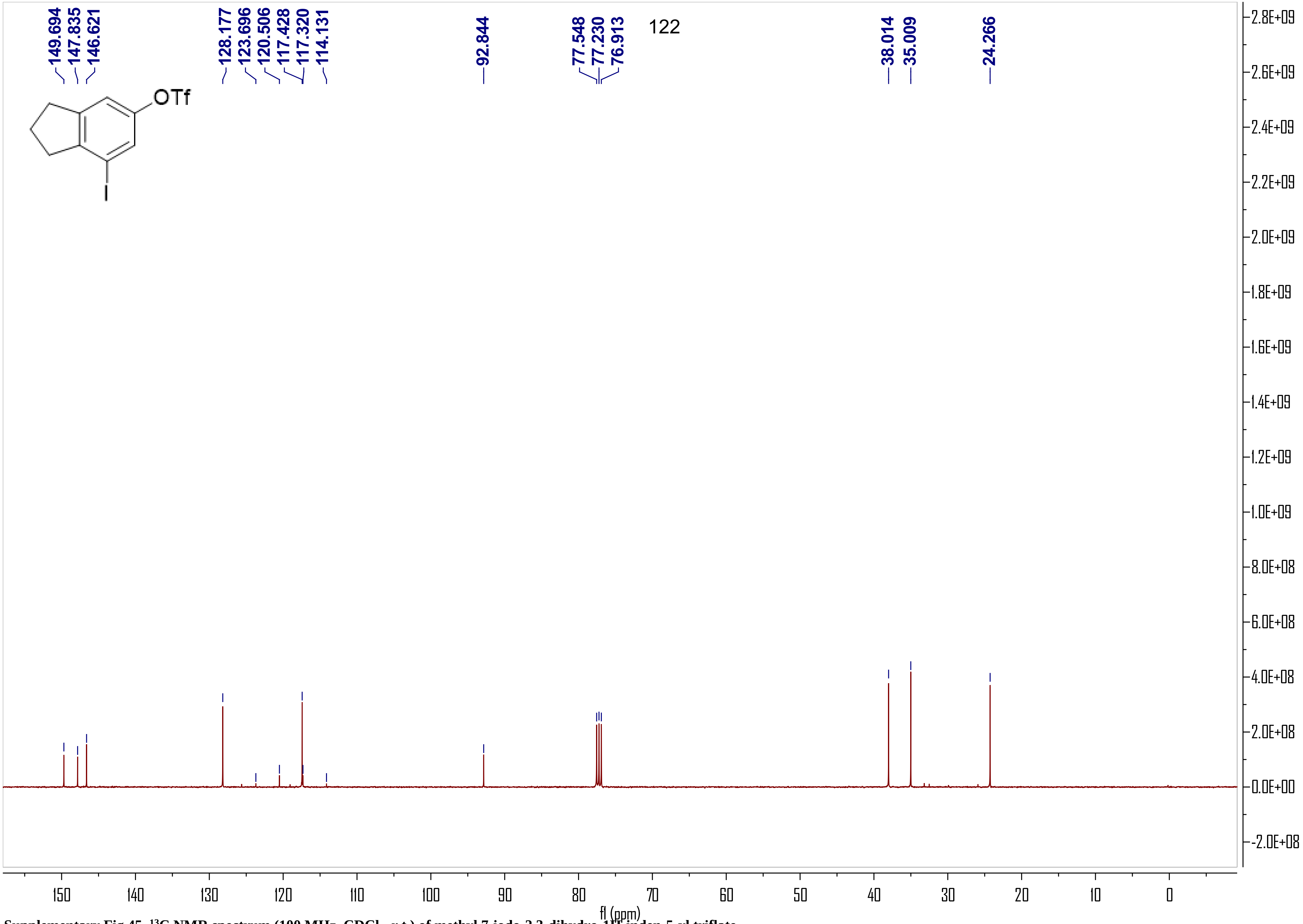

Supplementary Fig 45. ^{13}C **NMR spectrum (100 MHz, $CDCl_3$, r.t.) of methyl 7-iodo-2,3-dihydro-1H-inden-5-yl triflate.**

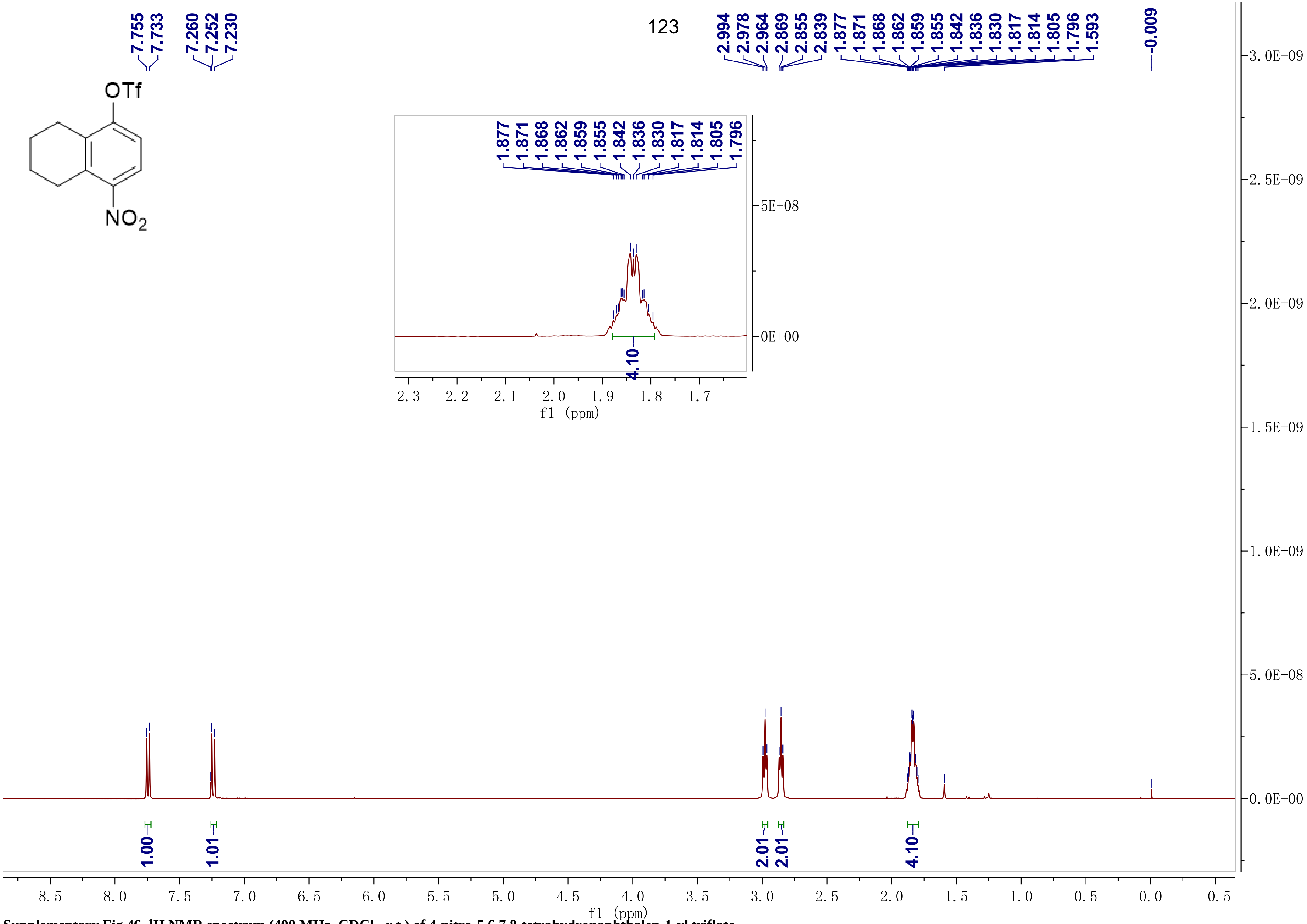

Supplementary Fig 46. **¹H NMR spectrum (400 MHz, $CDCl_3$, r.t.) of 4-nitro-5,6,7,8-tetrahydronaphthalen-1-yl triflate.**

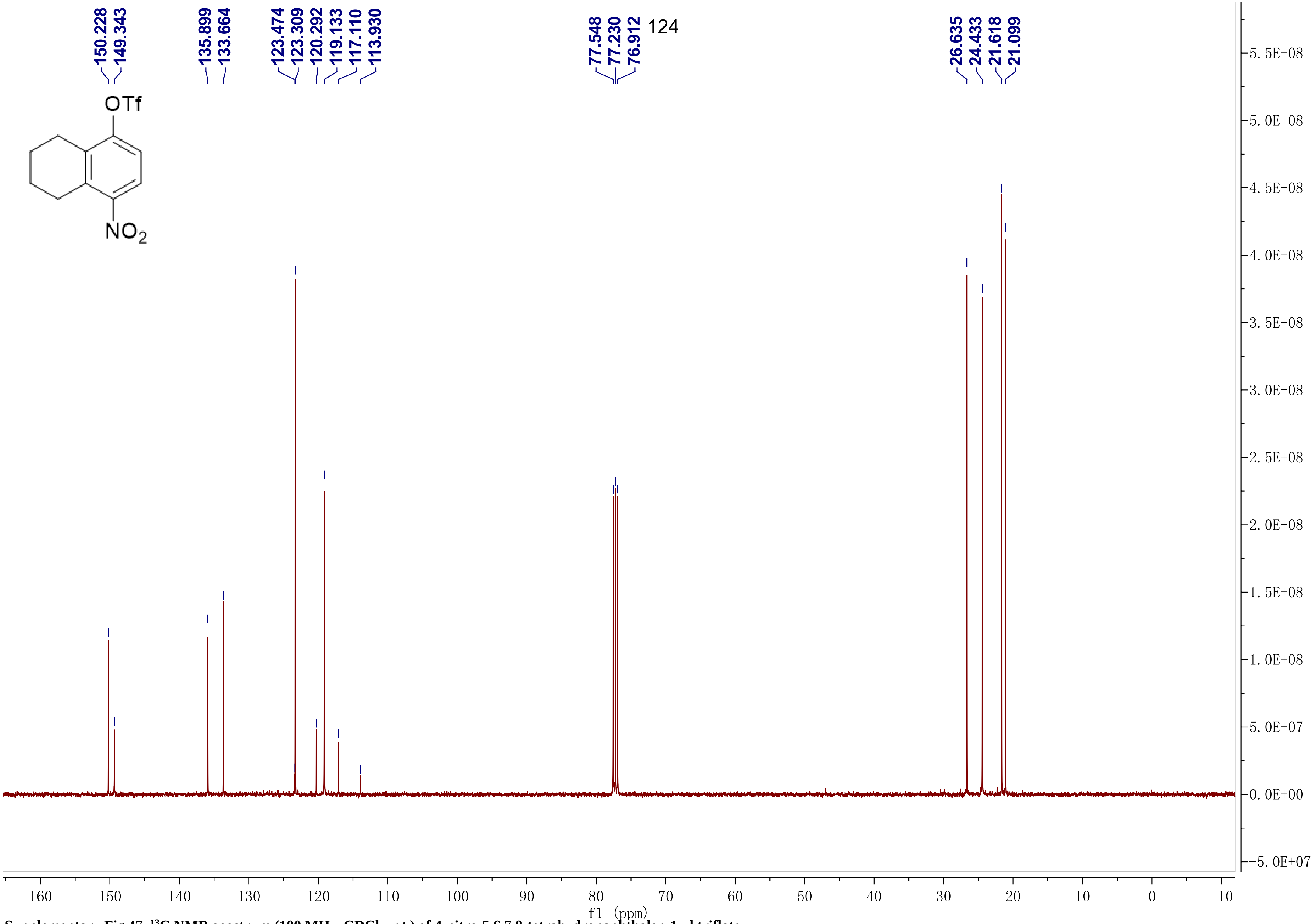

Supplementary Fig 47. ^{13}C NMR spectrum (100 MHz, $CDCl_3$, r.t.) of 4-nitro-5,6,7,8-tetrahydronaphthalen-1-yl triflate.

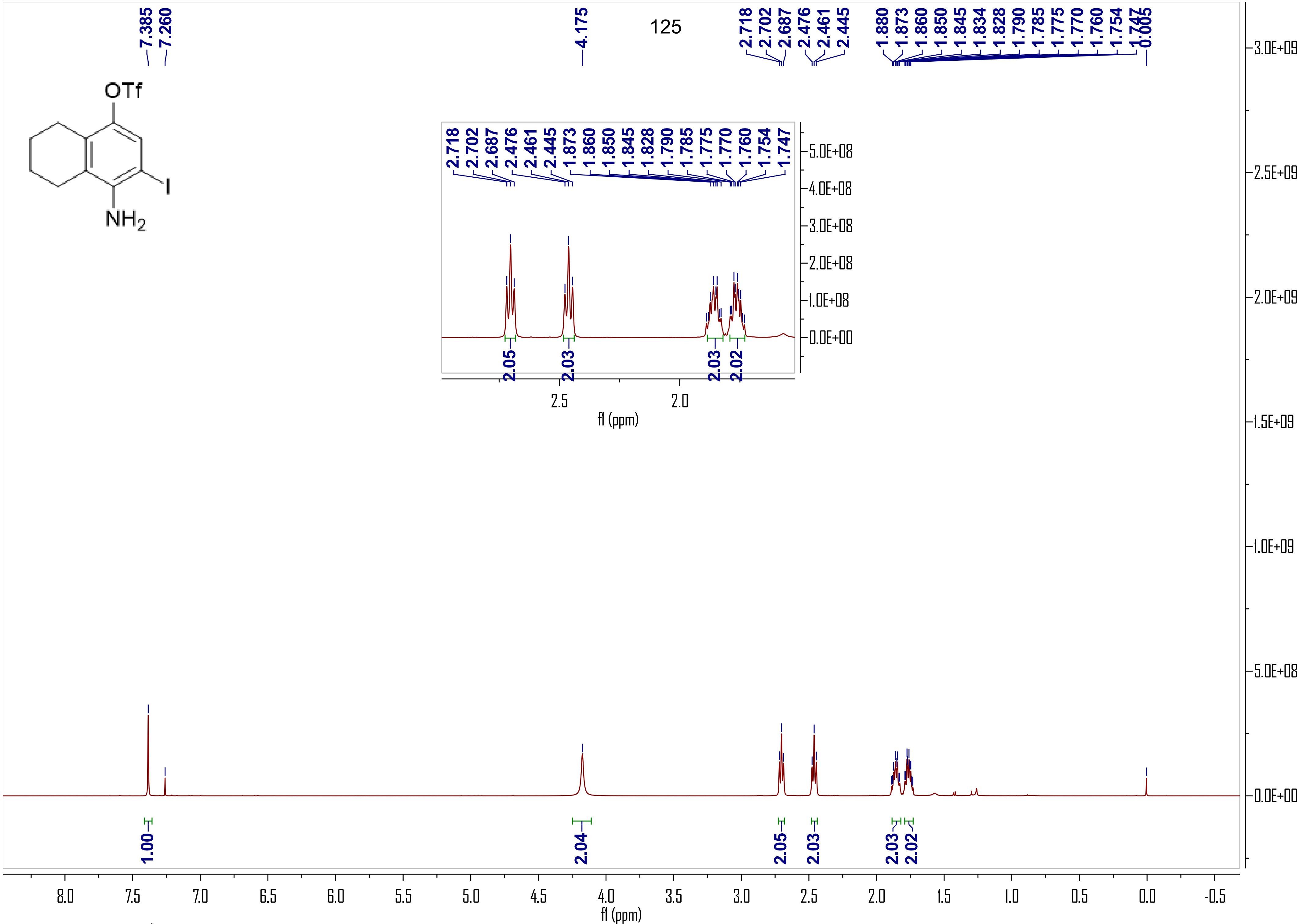

Supplementary Fig 48. **^{1}H NMR spectrum (400 MHz, $CDCl_3$, r.t.) of 4-amino-3-iodo-5,6,7,8-tetrahydronaphthalen-1-yl triflate.**

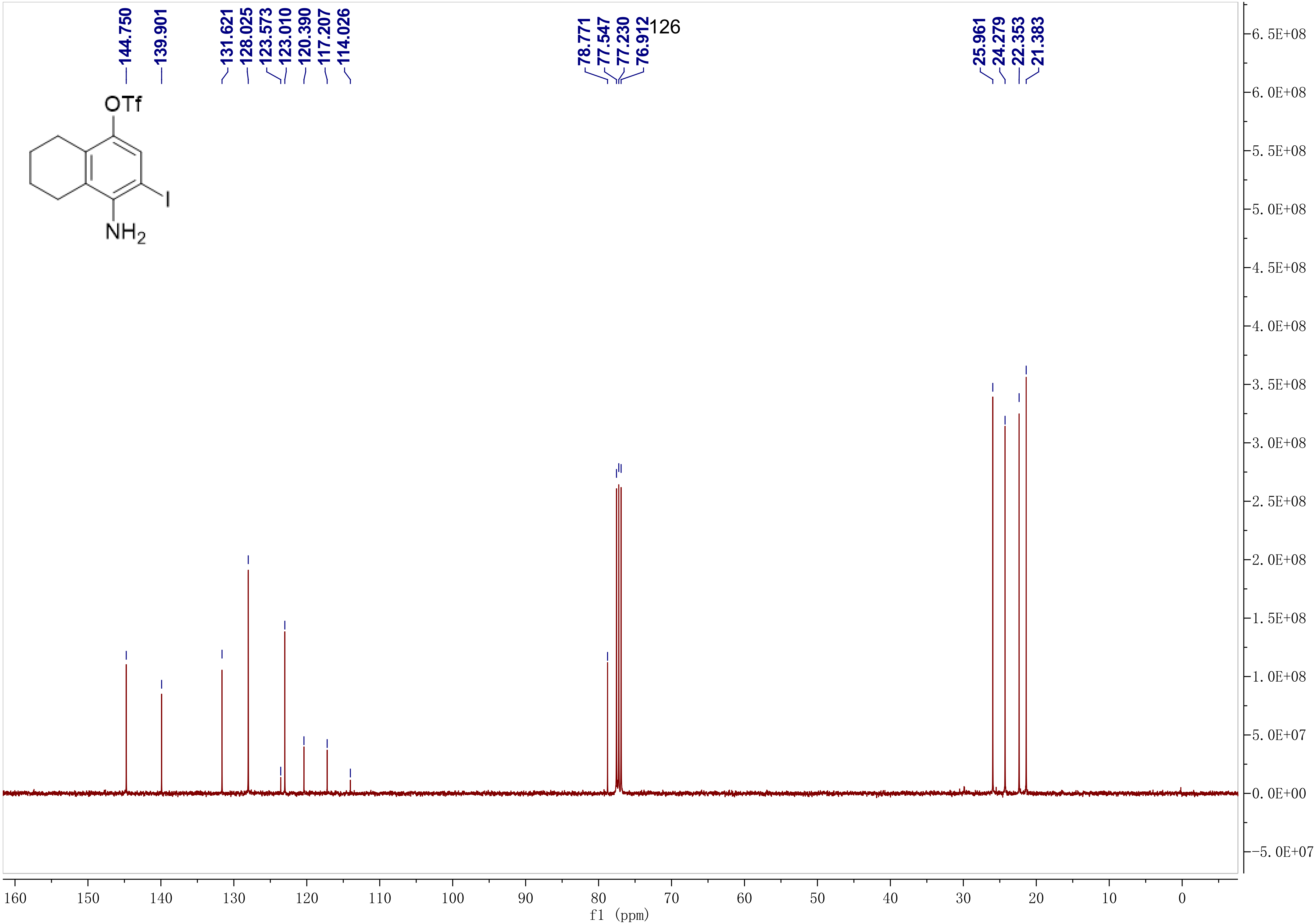

Supplementary Fig 49. ^{13}C NMR spectrum (100 MHz, $CDCl_3$, r.t.) of 4-amino-3-iodo-5,6,7,8-tetrahydronaphthalen-1-yl triflate.

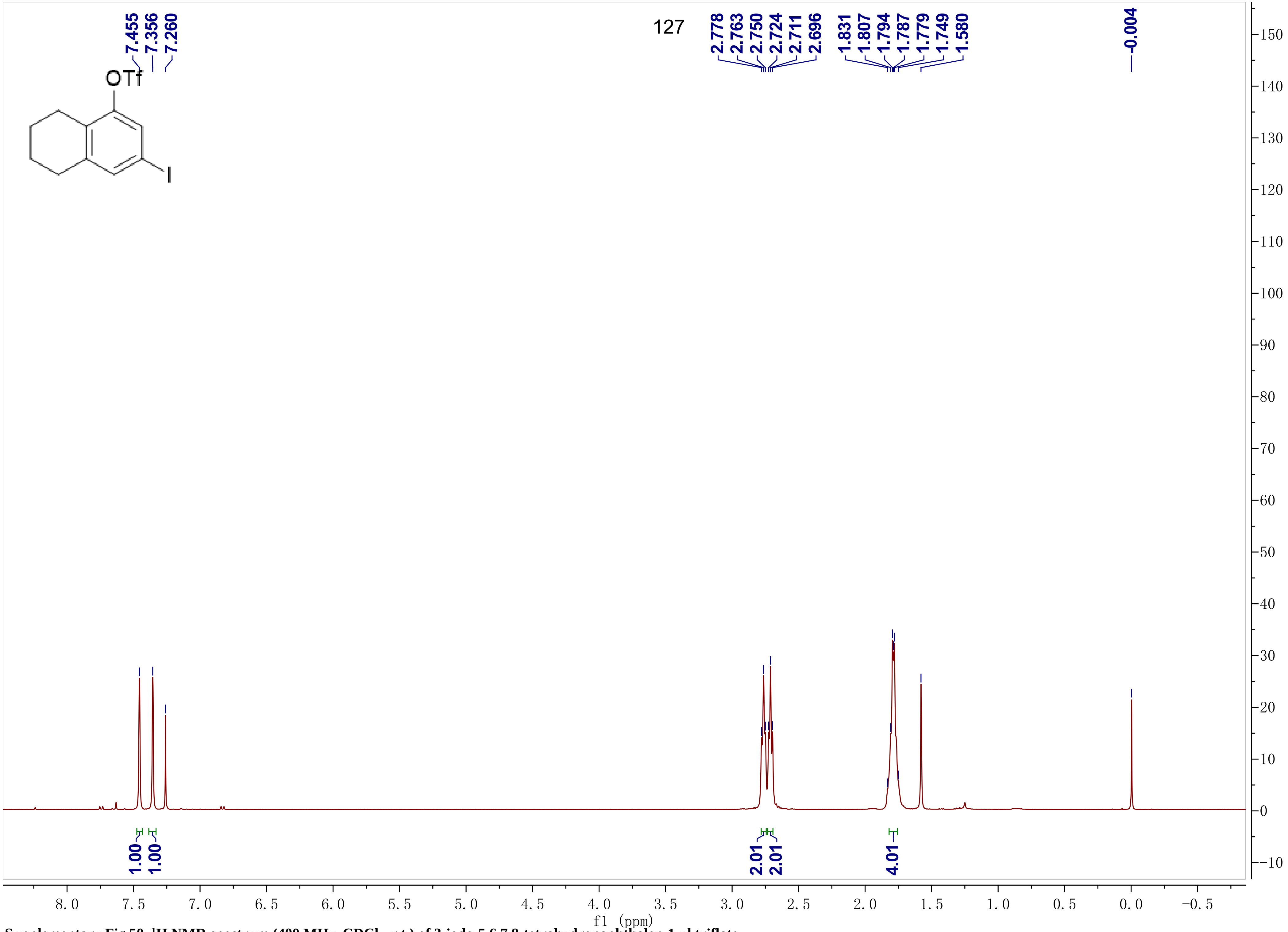

Supplementary Fig 50. ¹H NMR spectrum (400 MHz, $CDCl_3$, r.t.) of 3-iodo-5,6,7,8-tetrahydronaphthalen-1-yl triflate.

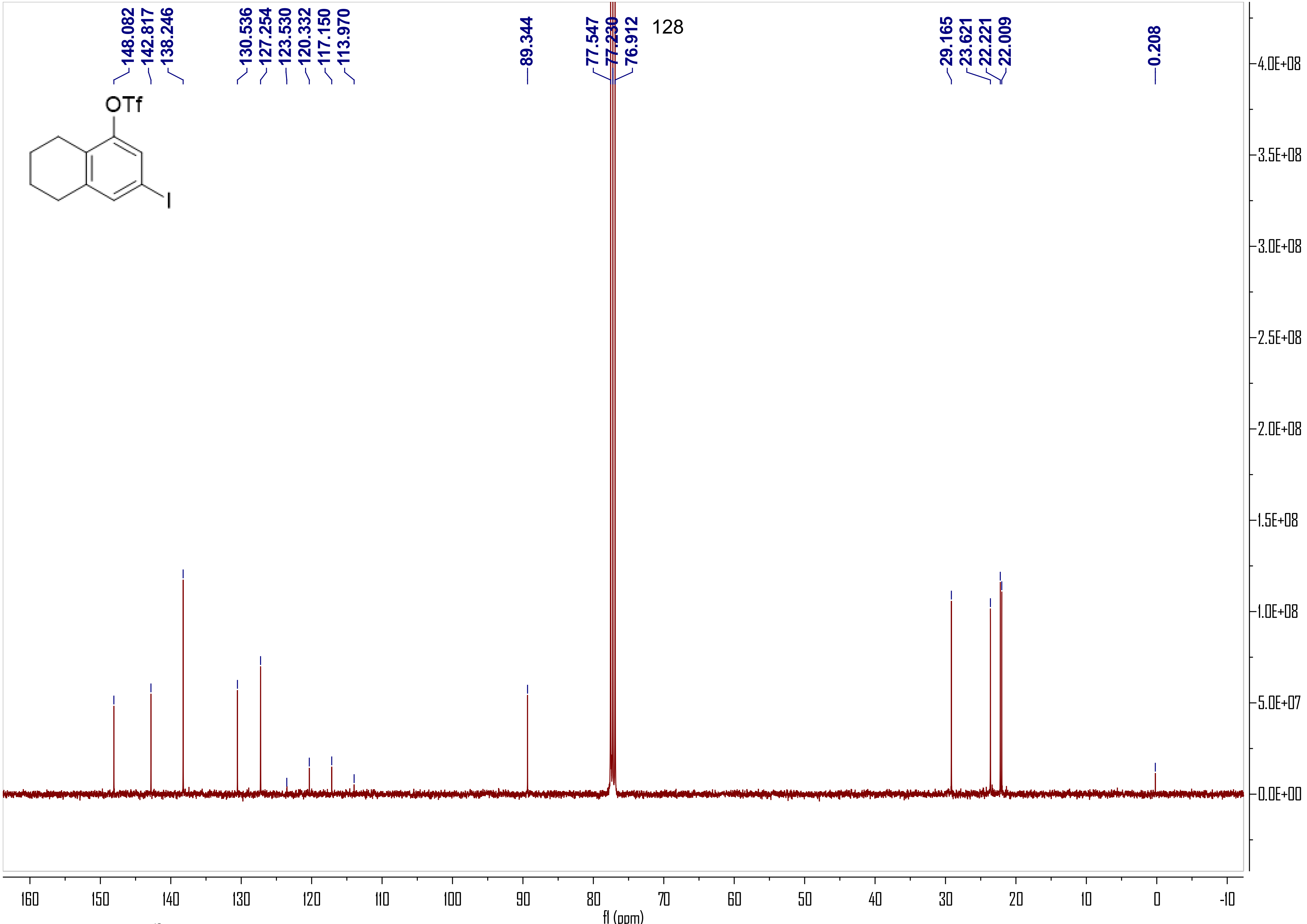

Supplementary Fig 51. ^{13}C **NMR spectrum (100 MHz,** $CDCl_3$**, r.t.) of 3-iodo-5,6,7,8-tetrahydronaphthalen-1-yl triflate.**

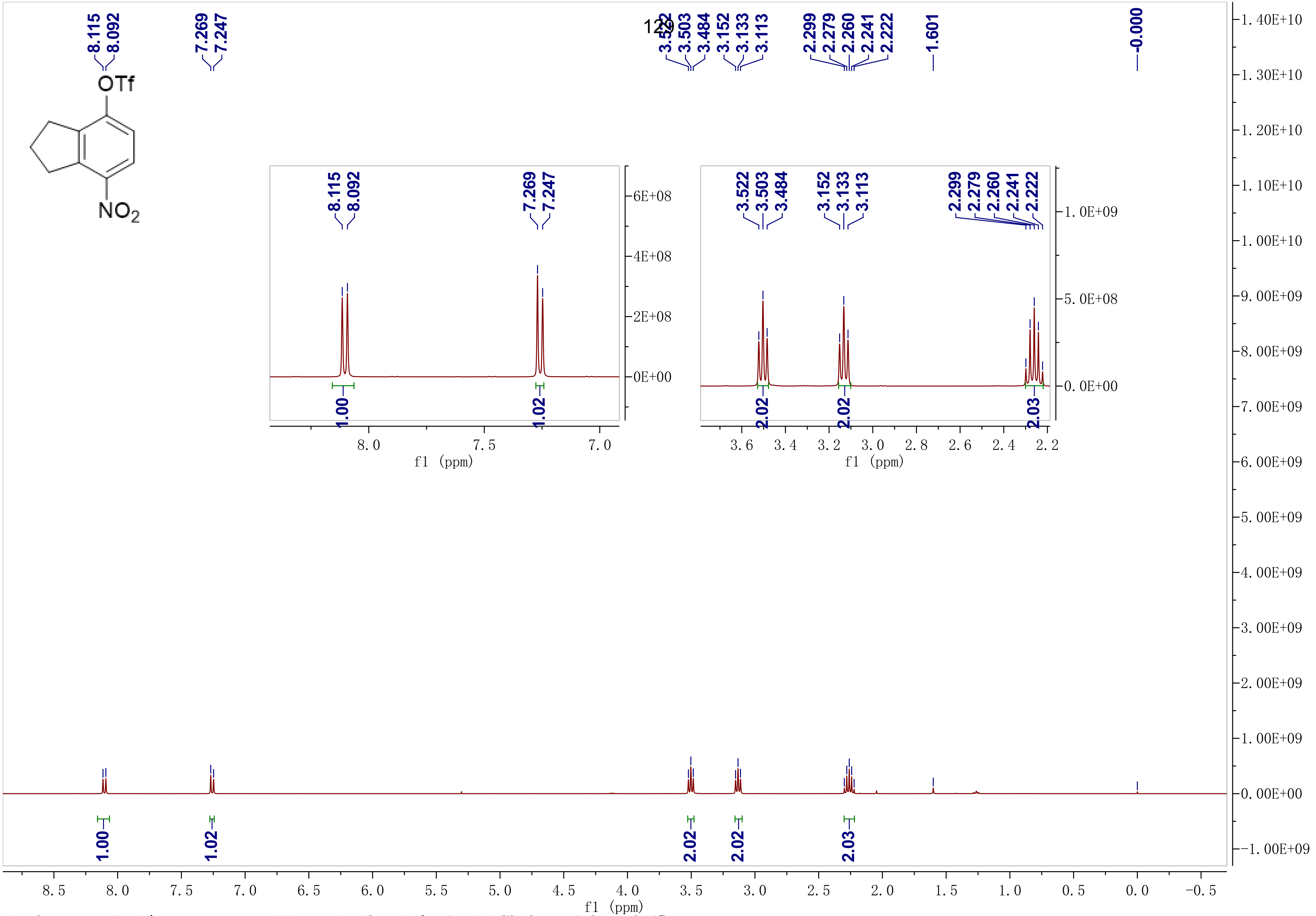

Supplementary Fig 52. **^{1}H NMR spectrum (400 MHz, $CDCl_3$, r.t.) of 7-nitro-2,3-dihydro-1*H*-inden-4-yl triflate.**

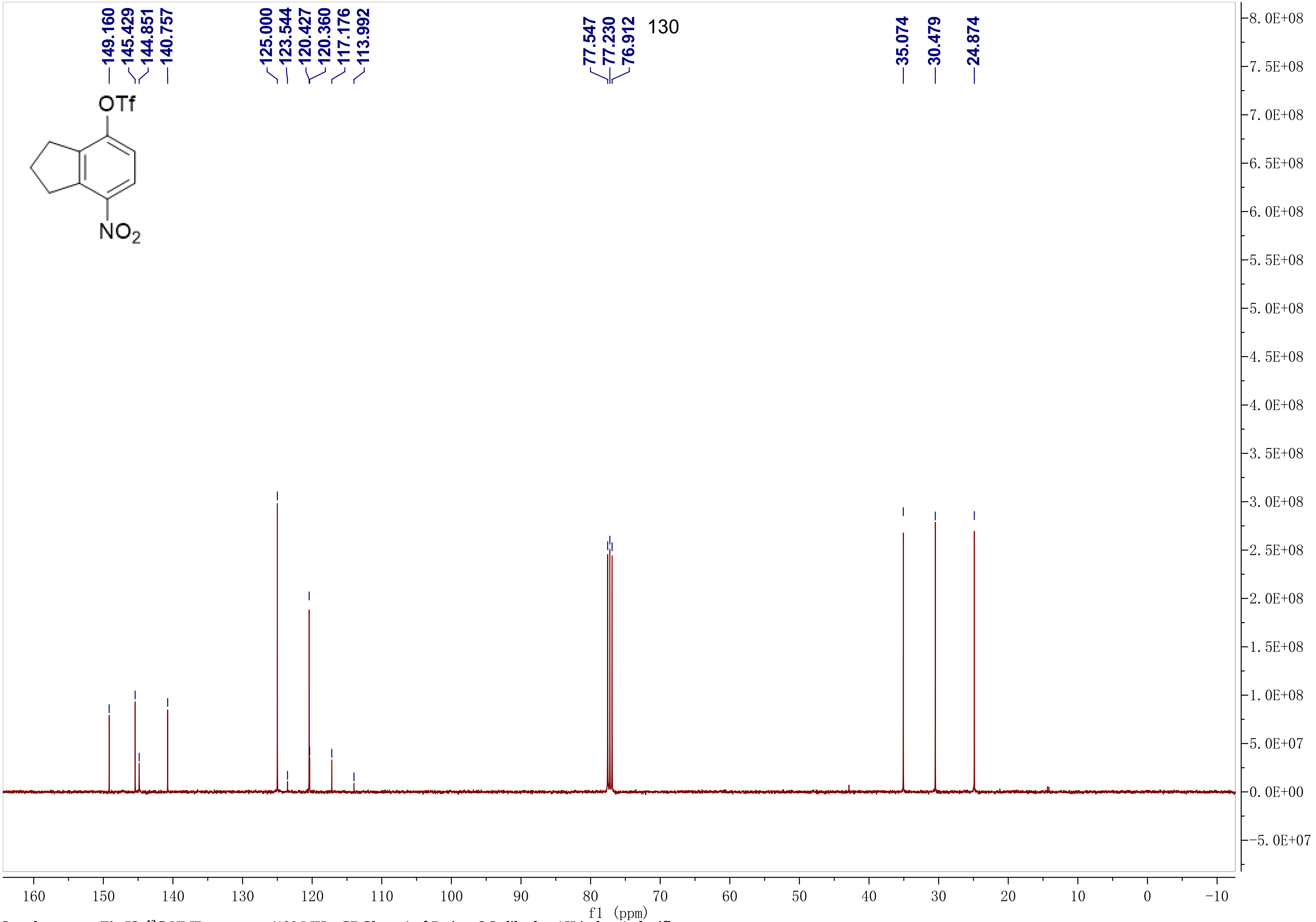

Supplementary Fig 53. **^{13}C NMR spectrum (100 MHz, $CDCl_3$, r.t.) of 7-nitro-2,3-dihydro-1*H*-inden-4-yl triflate.**

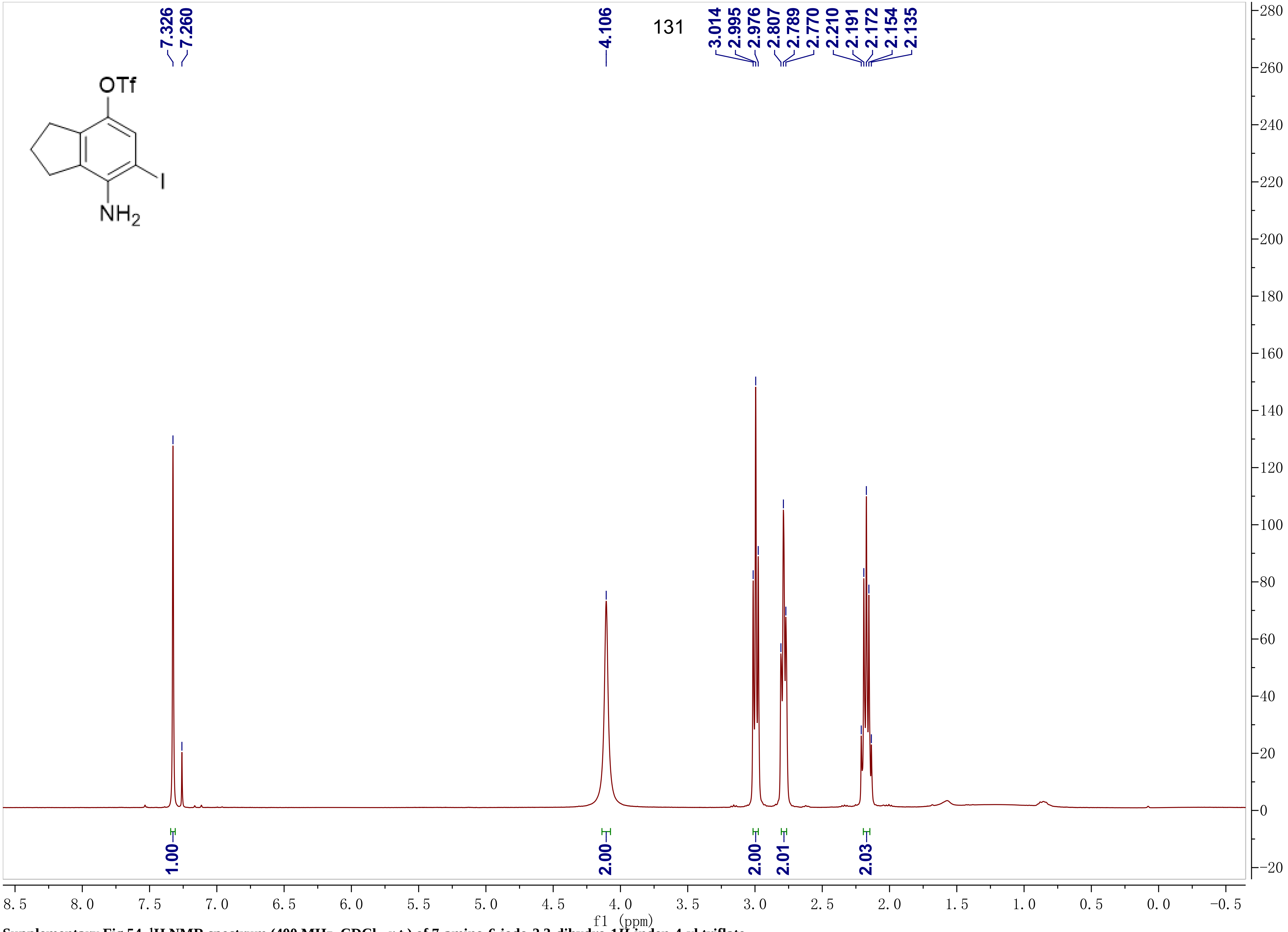

Supplementary Fig 54. **^{1}H NMR spectrum (400 MHz, $CDCl_3$, r.t.) of 7-amino-6-iodo-2,3-dihydro-1*H*-inden-4-yl triflate.**

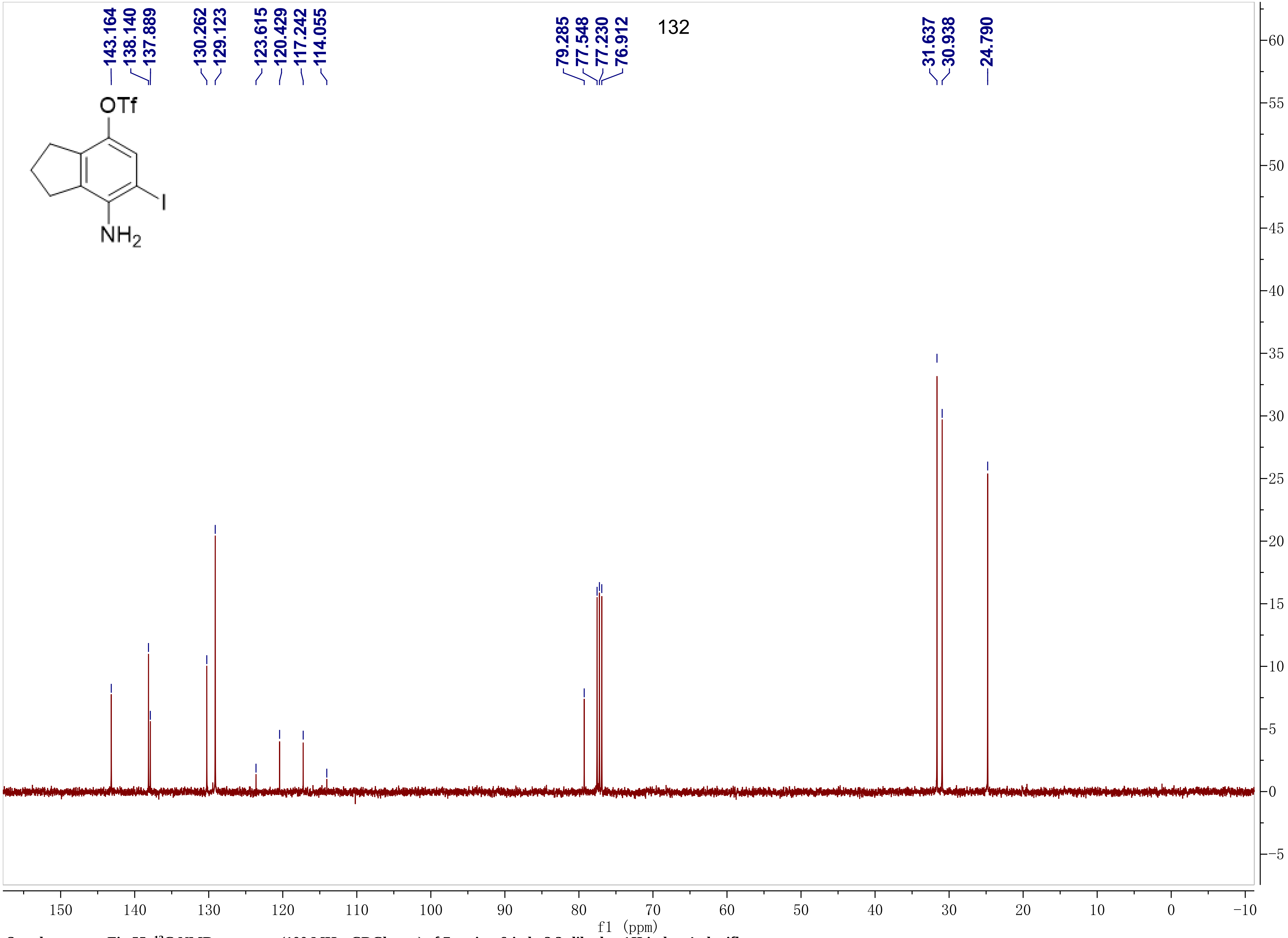

Supplementary Fig 55. **^{13}C NMR spectrum (100 MHz, $CDCl_3$, r.t.) of 7-amino-6-iodo-2,3-dihydro-1*H*-inden-4-yl triflate.**

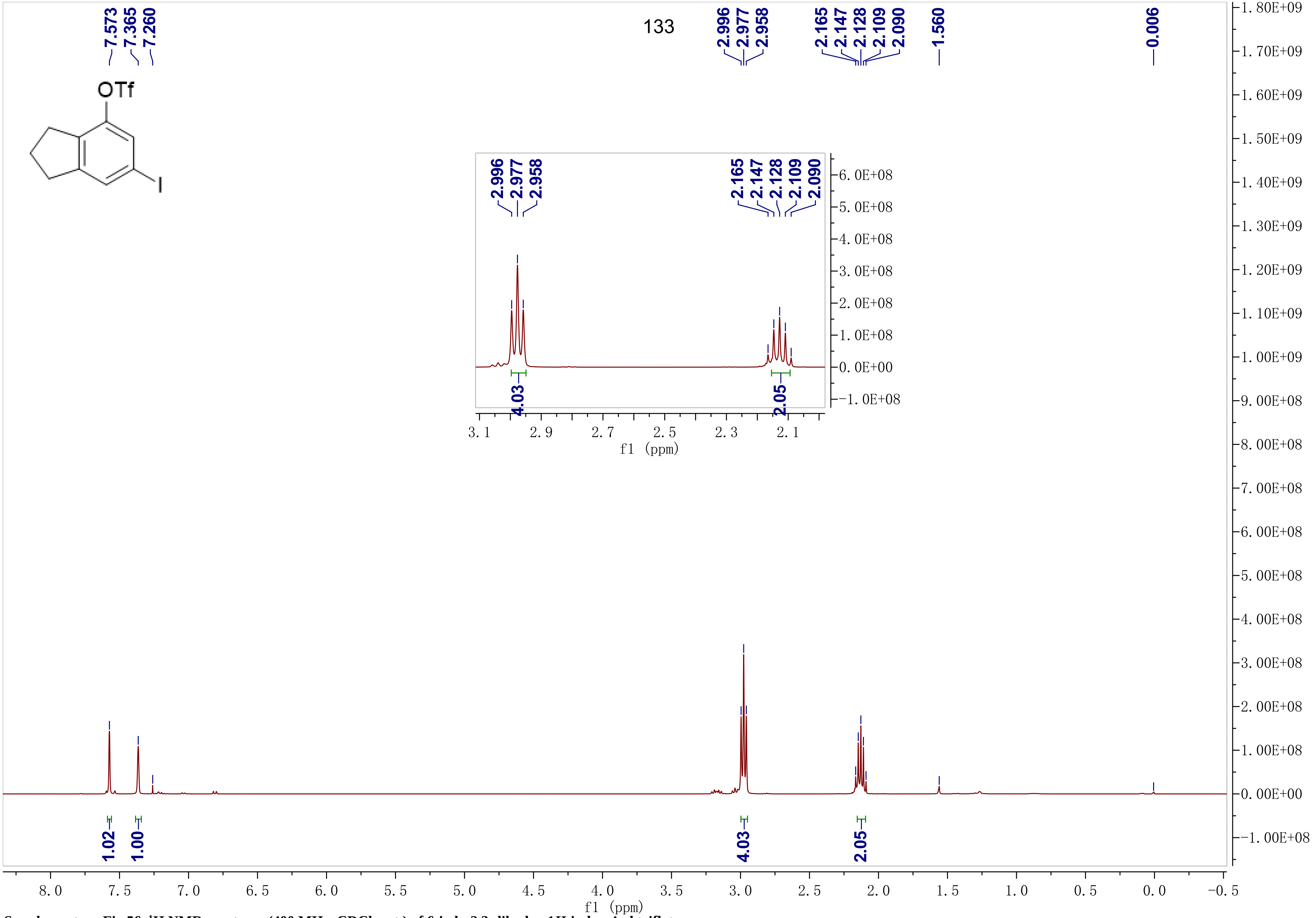

Supplementary Fig 56. ^{1}H NMR spectrum (400 MHz, $CDCl_3$, r.t.) of 6-iodo-2,3-dihydro-1*H*-inden-4-yl triflate.

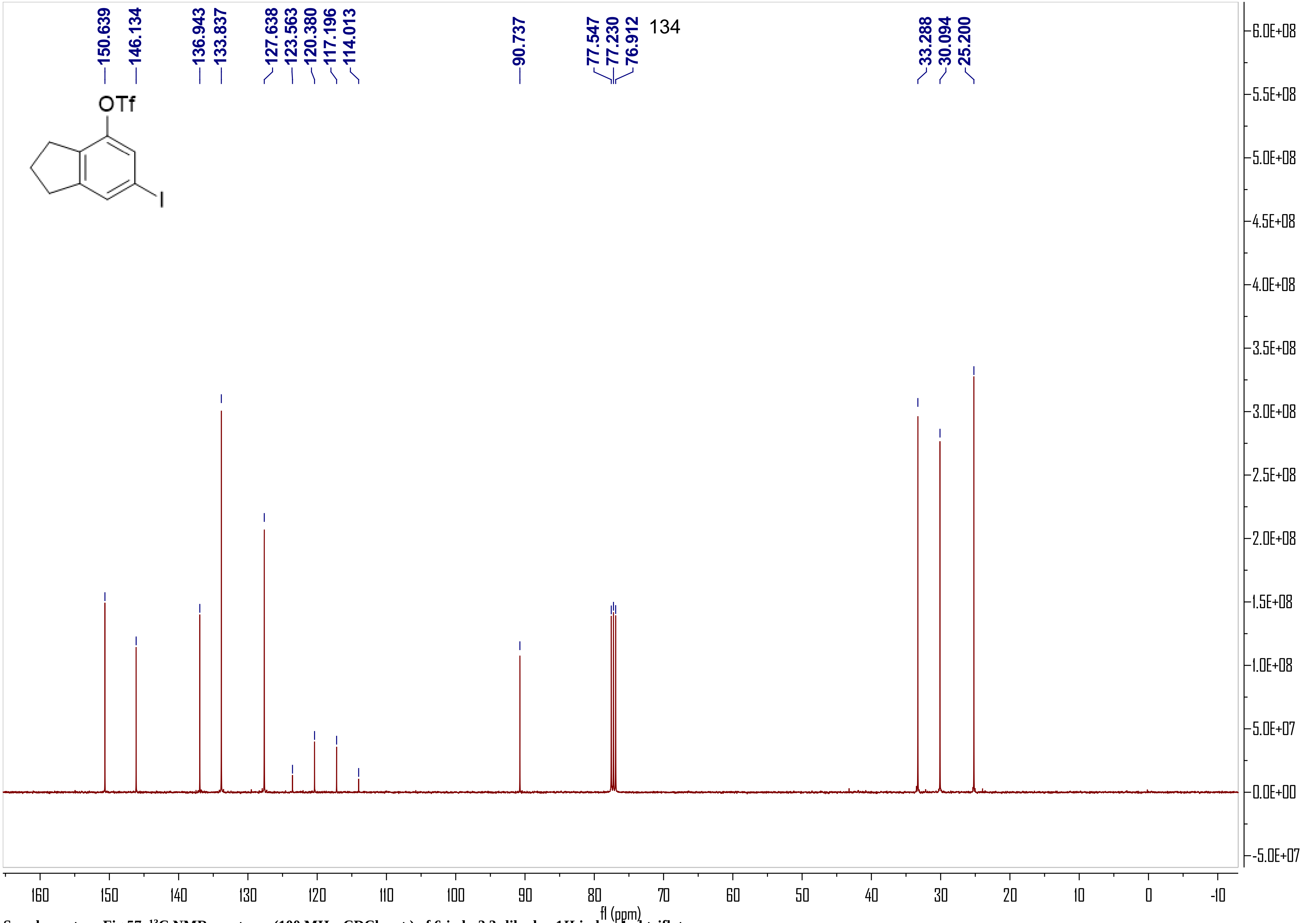

Supplementary Fig 57. ^{13}C NMR spectrum (100 MHz, $CDCl_3$, r.t.) of 6-iodo-2,3-dihydro-1*H*-inden-4-yl triflate.

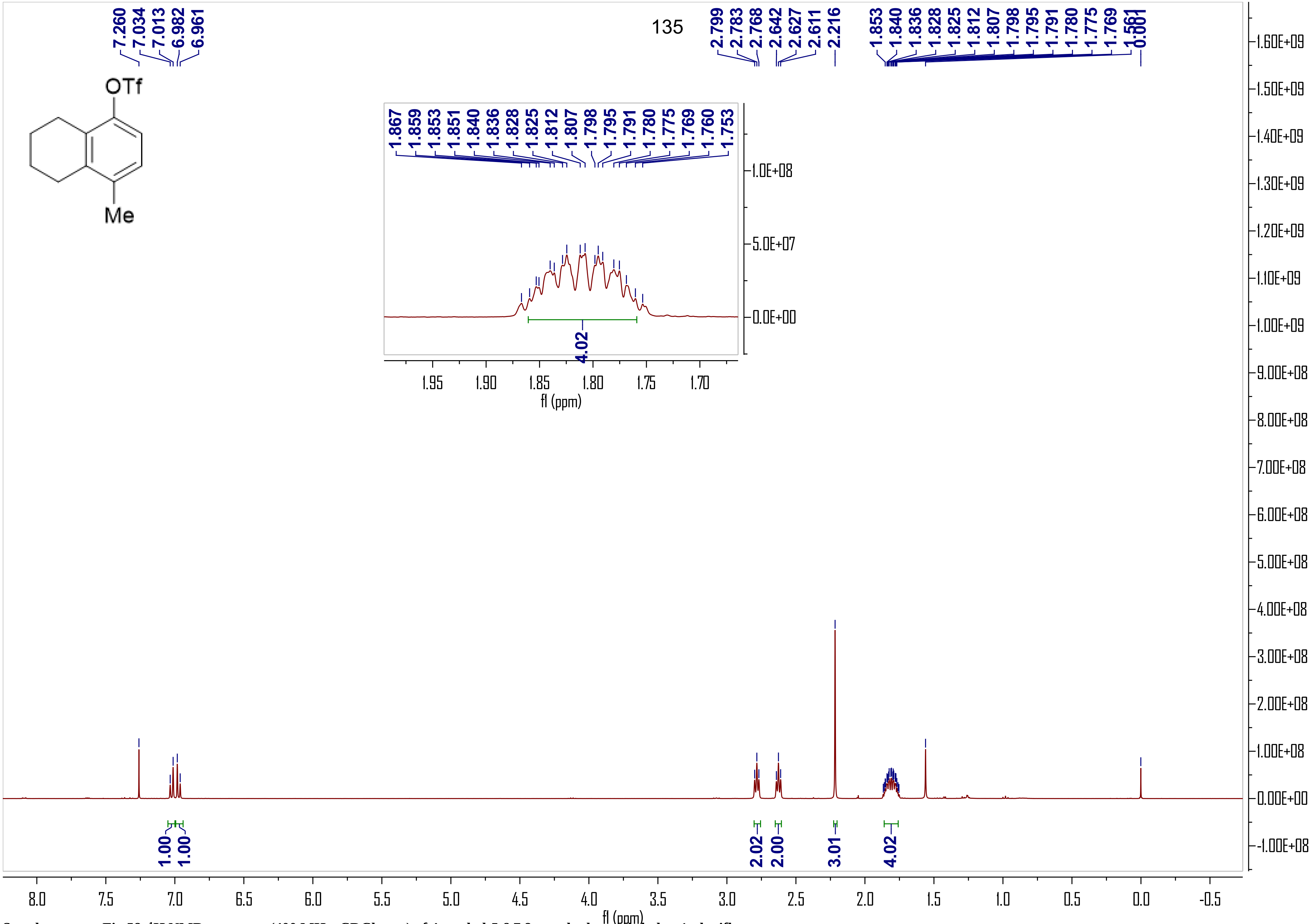

Supplementary Fig 58. **^{1}H NMR spectrum (400 MHz, $CDCl_3$, r.t.) of 4-methyl-5,6,7,8-tetrahydronaphthalen-1-yl triflate.**

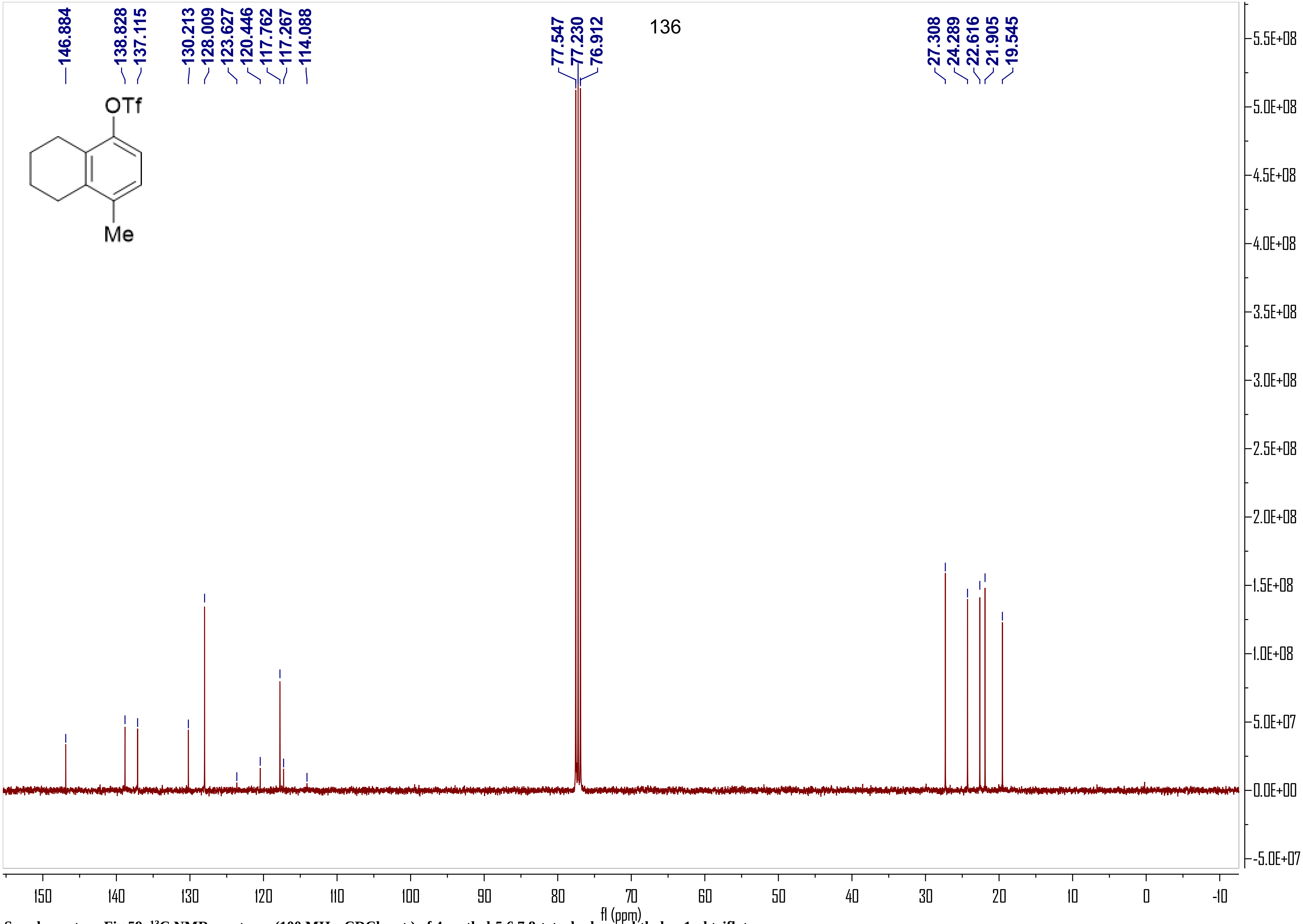

Supplementary Fig 59. ^{13}C **NMR spectrum (100 MHz,** $CDCl_3$**, r.t.) of 4-methyl-5,6,7,8-tetrahydronaphthalen-1-yl triflate.**

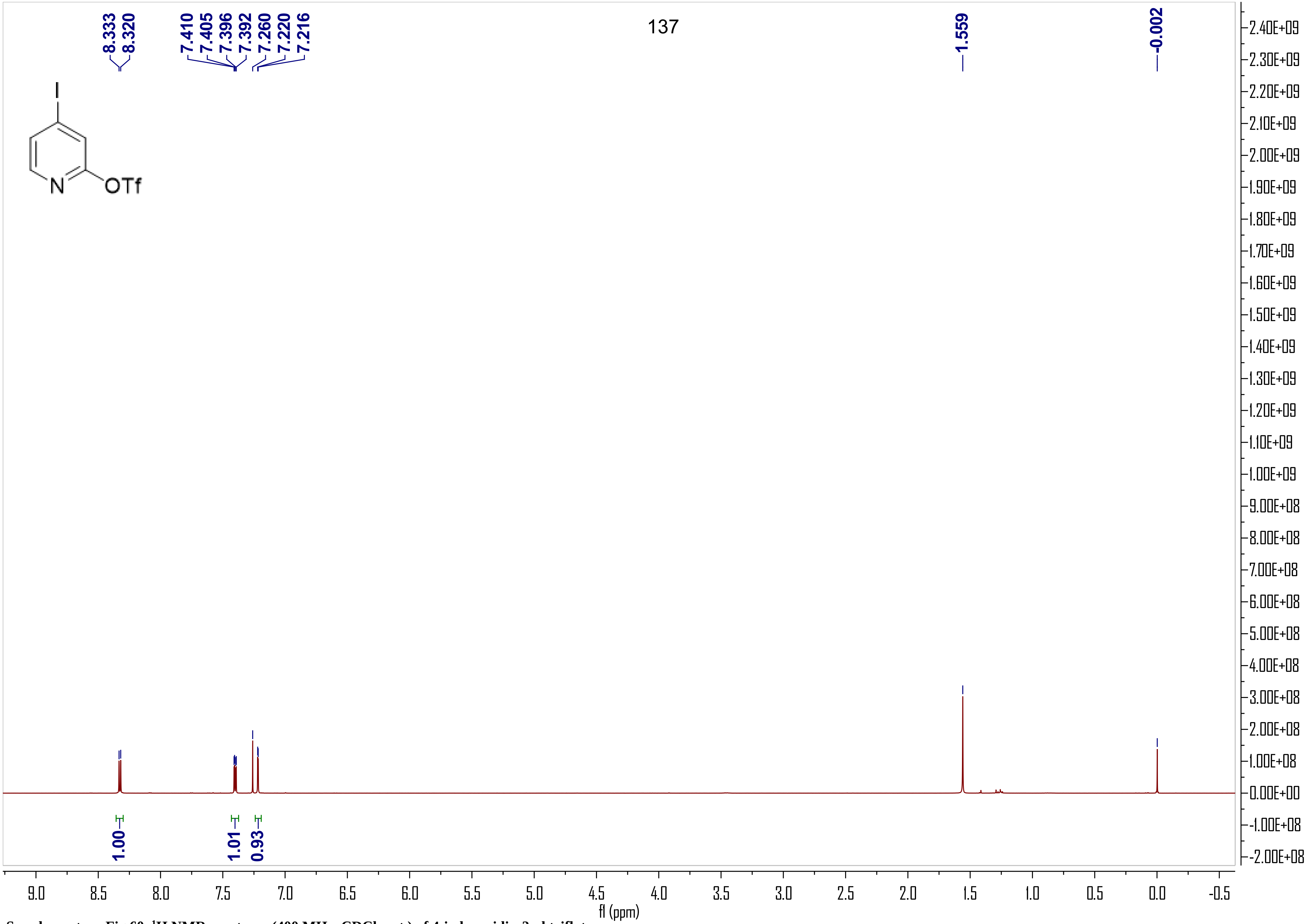

Supplementary Fig 60. ^{1}H NMR spectrum (400 MHz, $CDCl_3$, r.t.) of 4-iodopyridin-2-yl triflate.

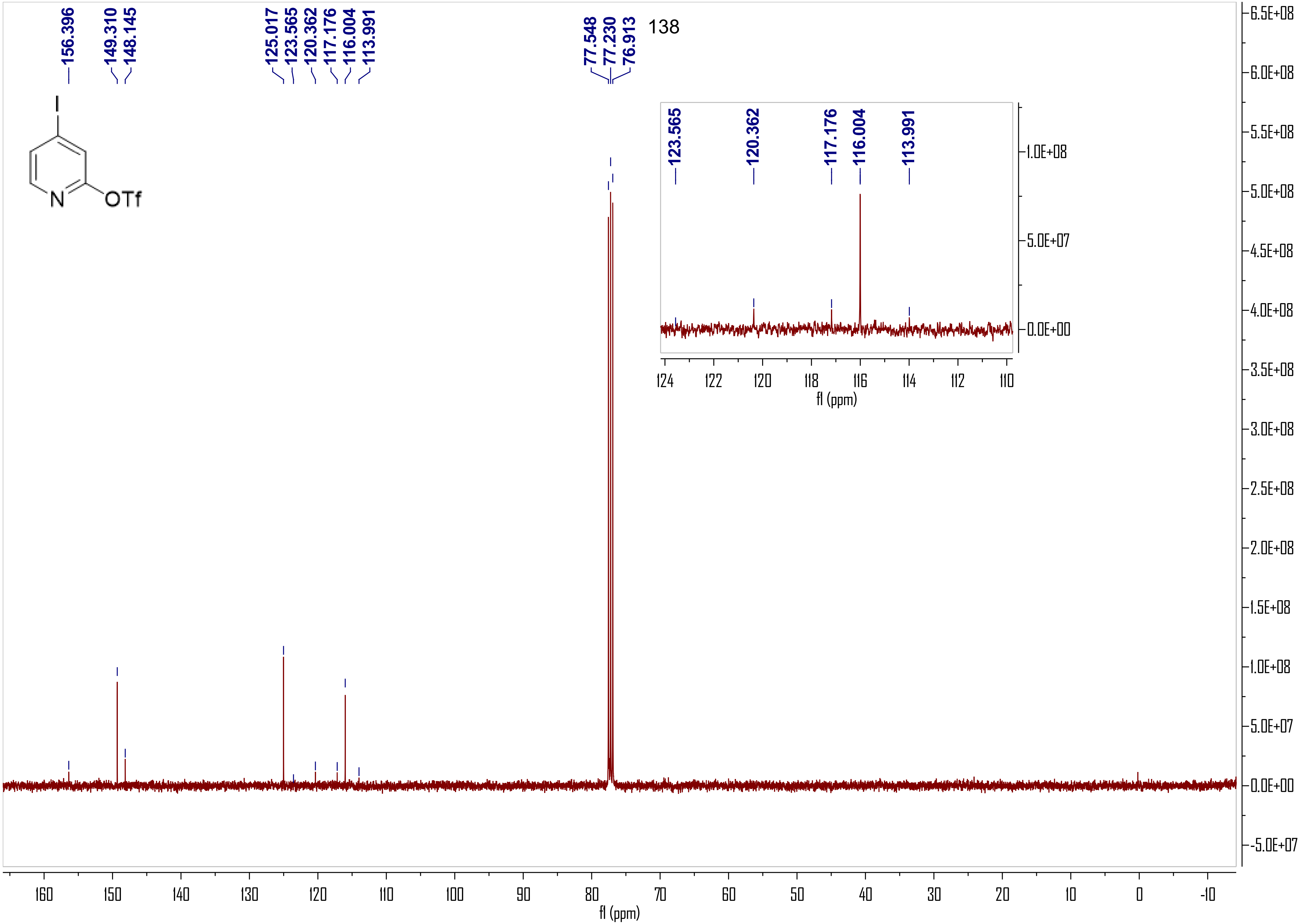

Supplementary Fig 61. ^{13}C **NMR spectrum (100 MHz,** $CDCl_3$**, r.t.) of 4-iodopyridin-2-yl triflate.**

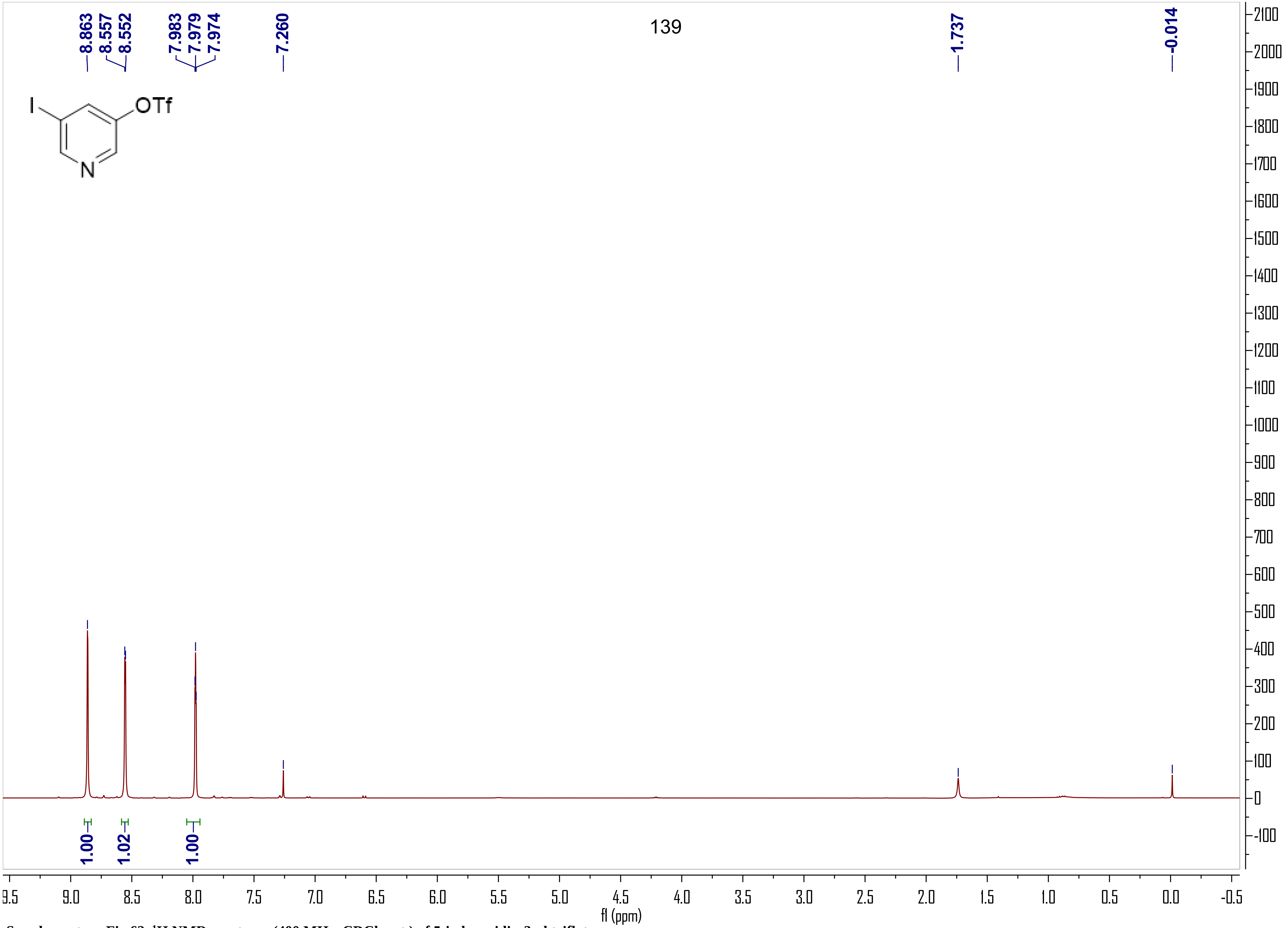

Supplementary Fig 62. ^{1}H NMR spectrum (400 MHz, $CDCl_3$, r.t.) of 5-iodopyridin-3-yl triflate.

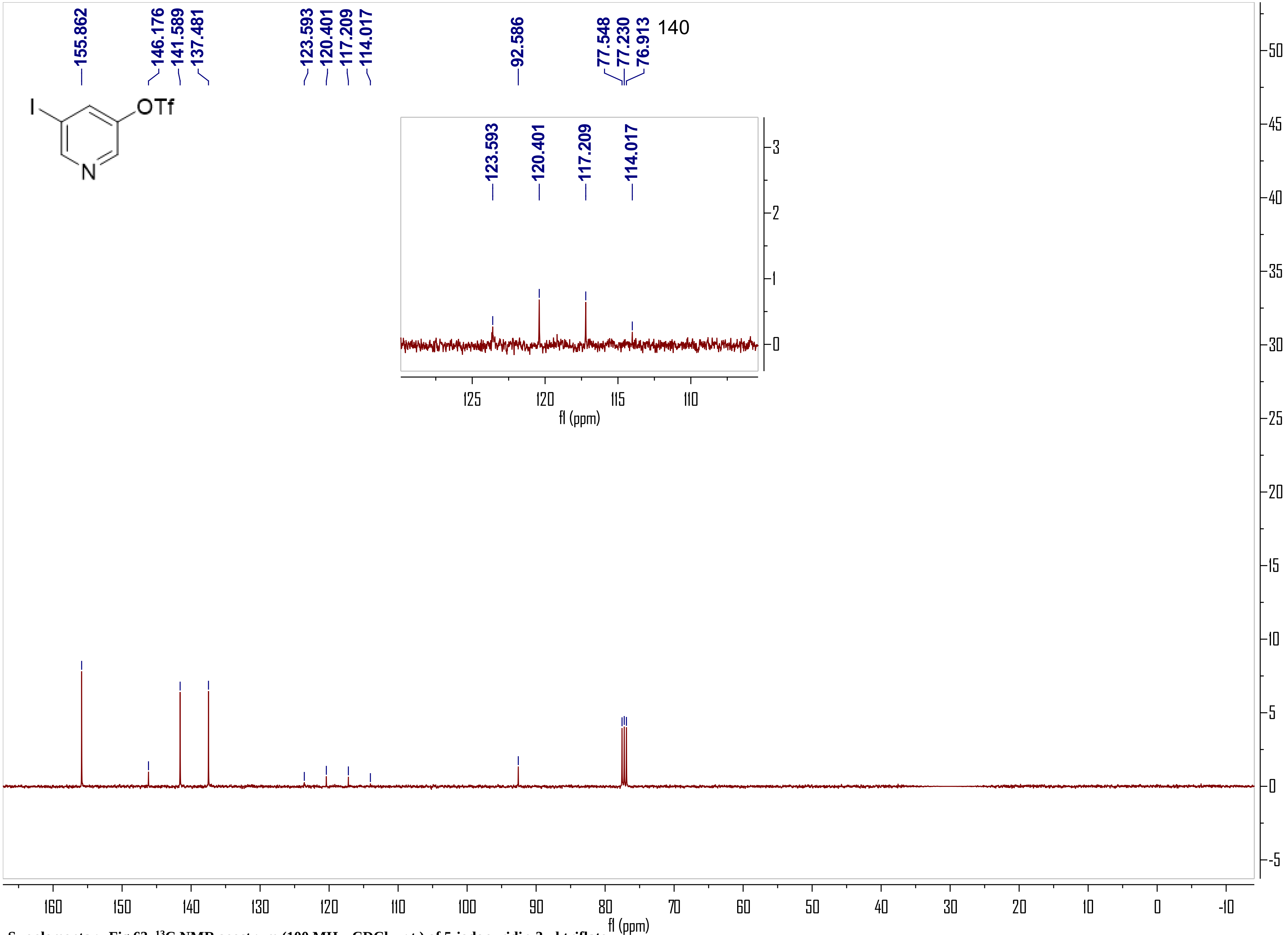

Supplementary Fig 63. **^{13}C NMR spectrum (100 MHz, $CDCl_3$, r.t.) of 5-iodopyridin-3-yl triflate.**

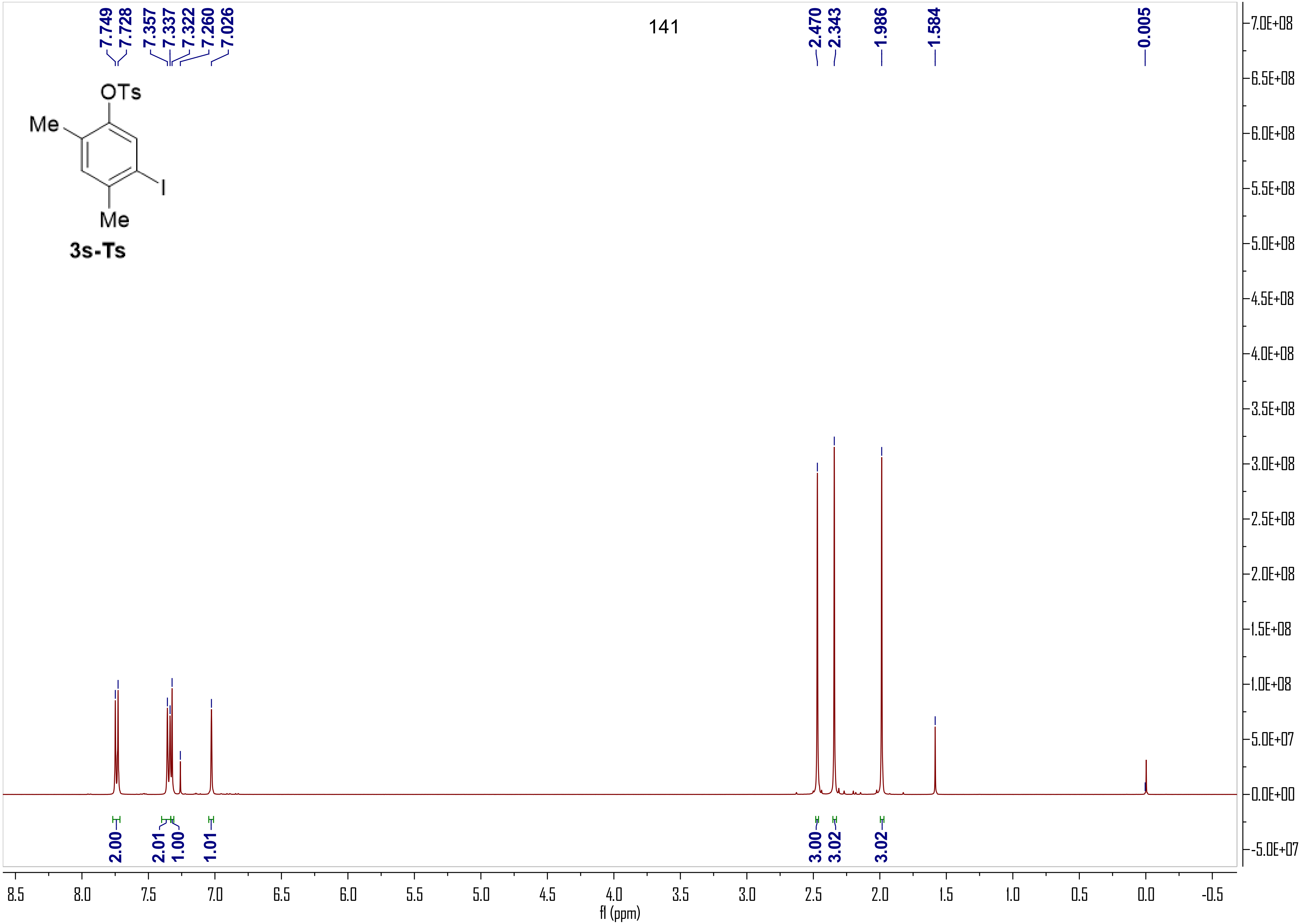

Supplementary Fig 64. **^{1}H NMR spectrum (400 MHz, $CDCl_3$, r.t.) of 3s-Ts.**

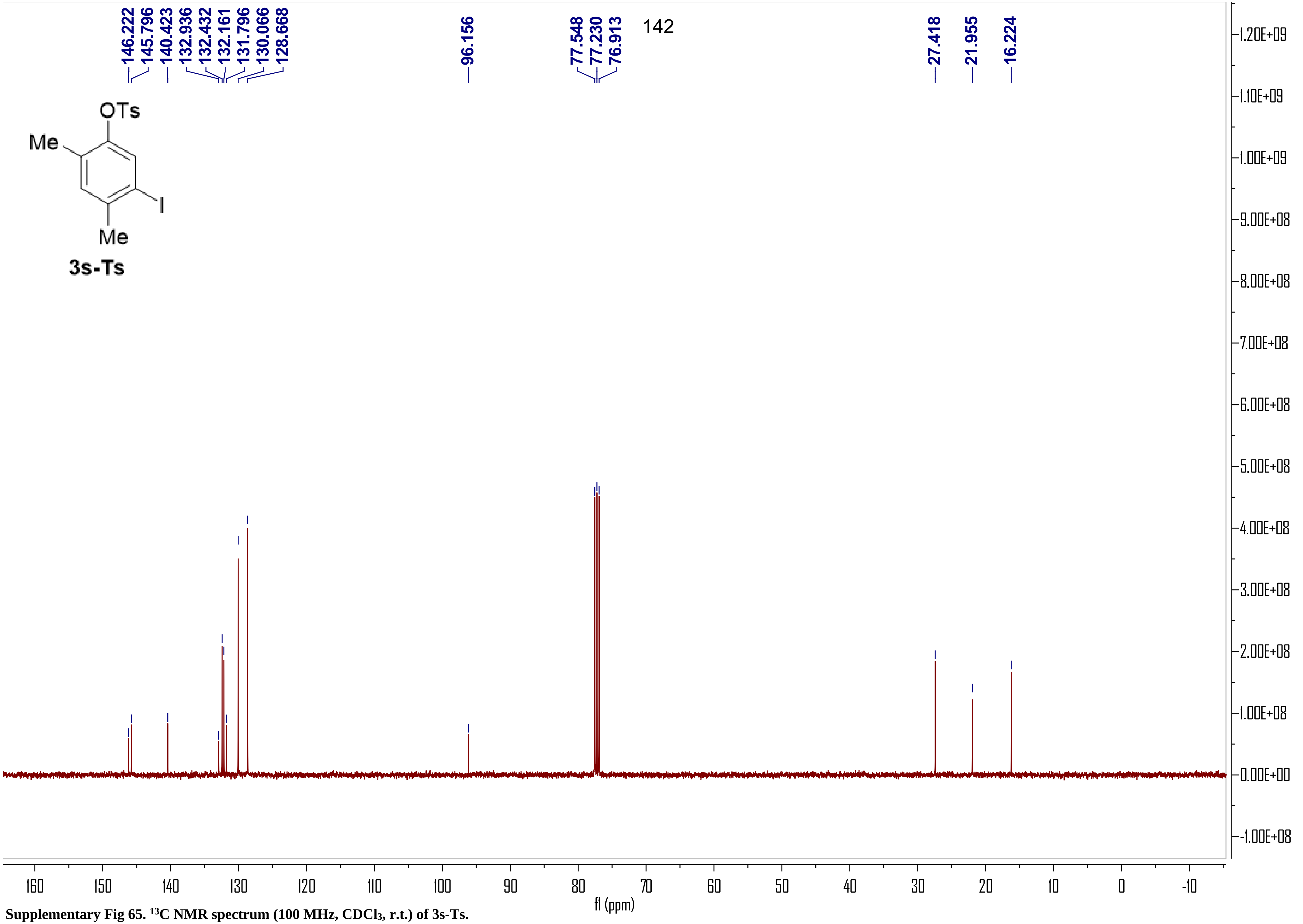

3s-Ts

Supplementary Fig 65. **^{13}C NMR spectrum (100 MHz, $CDCl_3$, r.t.) of 3s-Ts.**

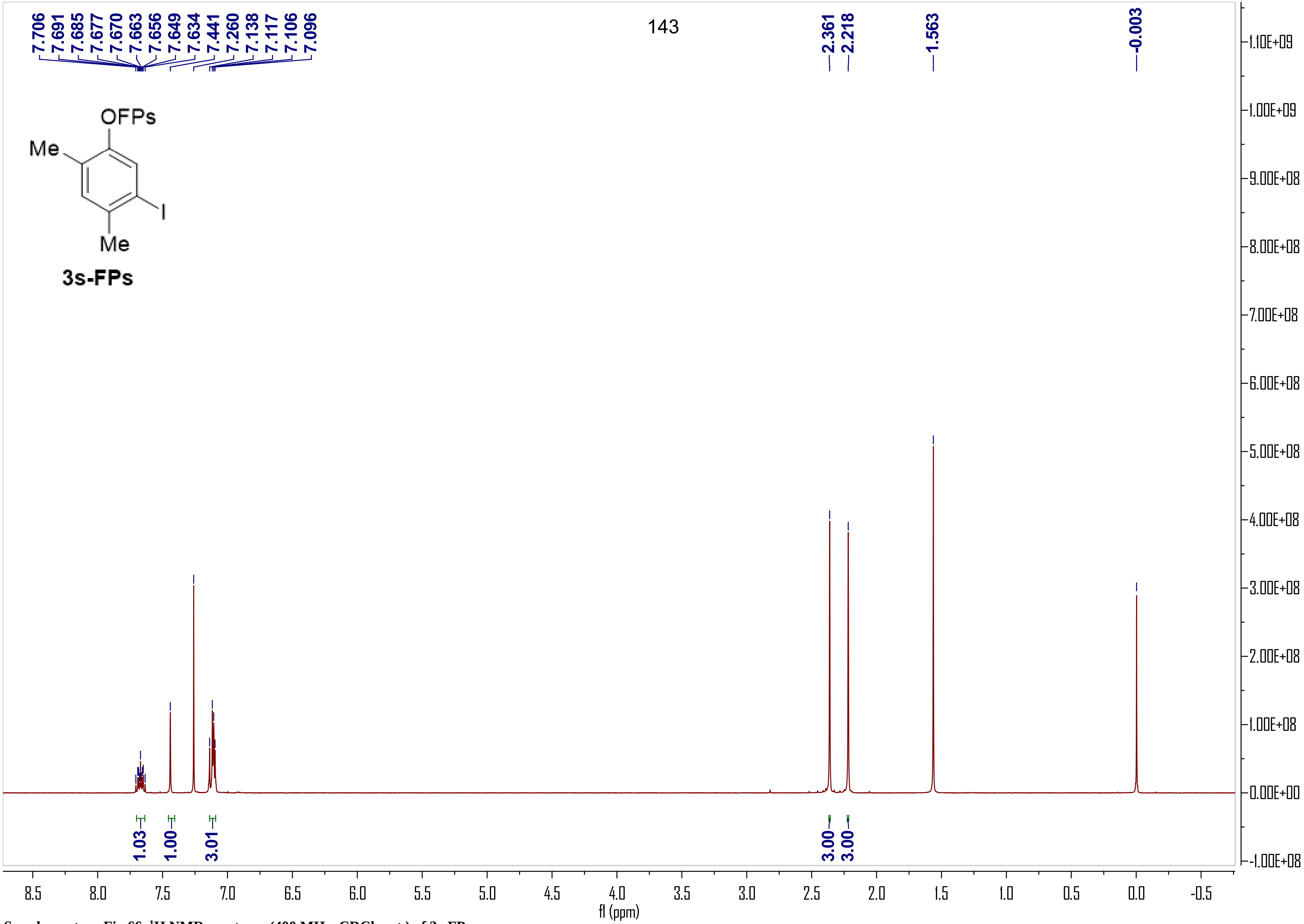

Supplementary Fig 66. **^{1}H NMR spectrum (400 MHz, $CDCl_3$, r.t.) of 3s-FPs.**

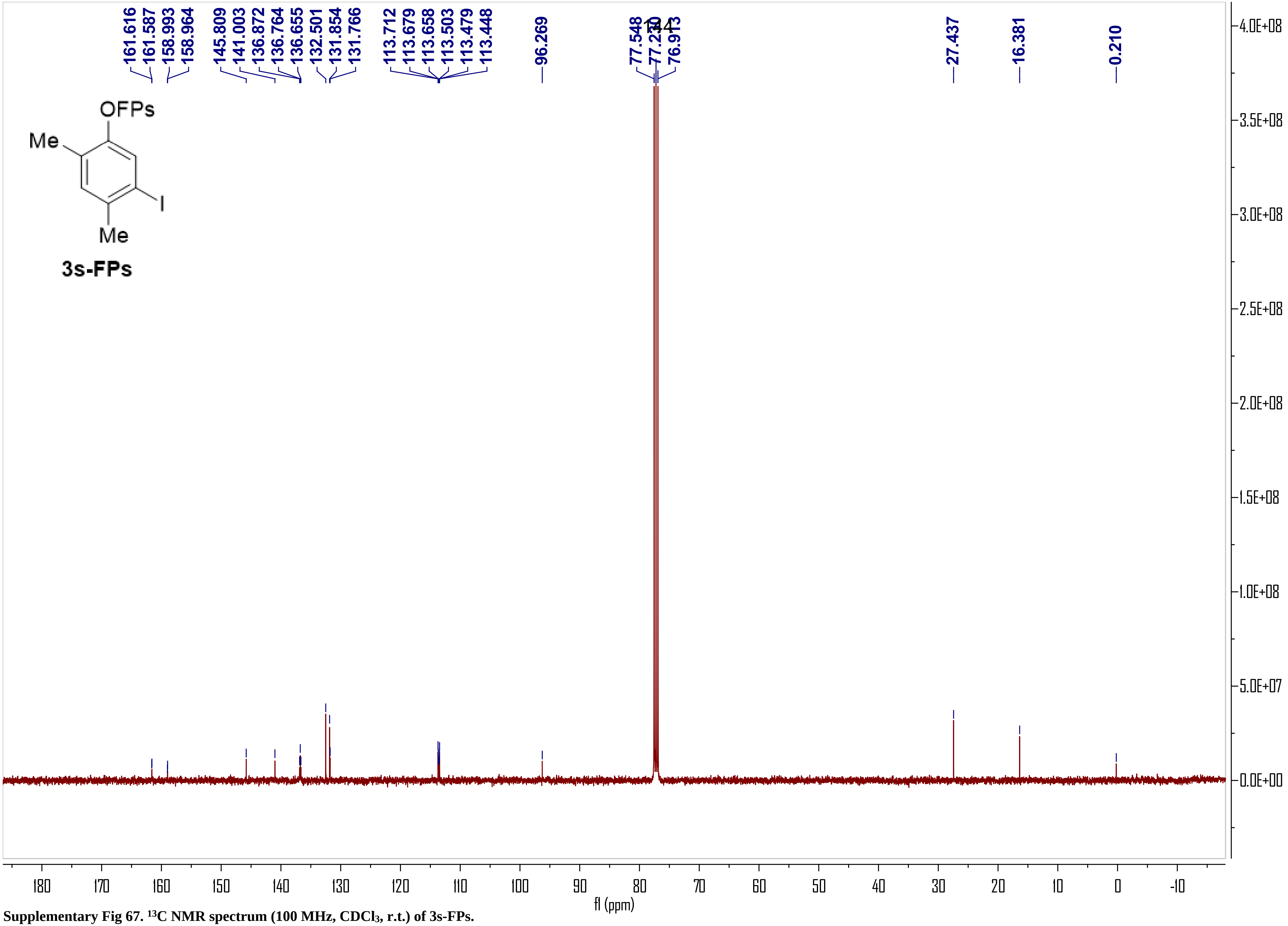

Supplementary Fig 67. ^{13}**C NMR spectrum (100 MHz, CDCl$_3$, r.t.) of 3s-FPs.**

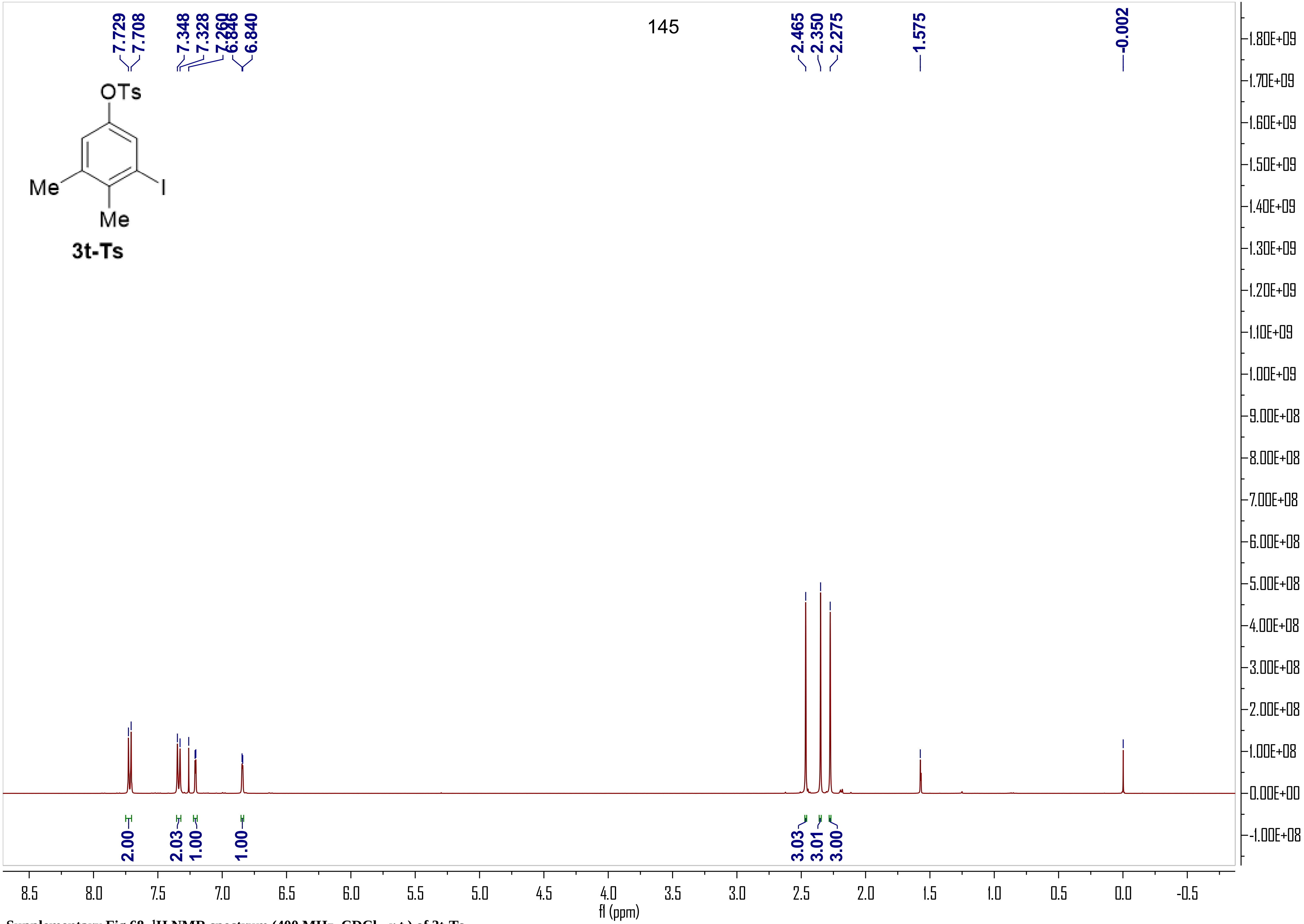

Supplementary Fig 68. ^{1}H NMR spectrum (400 MHz, $CDCl_3$, r.t.) of 3t-Ts.

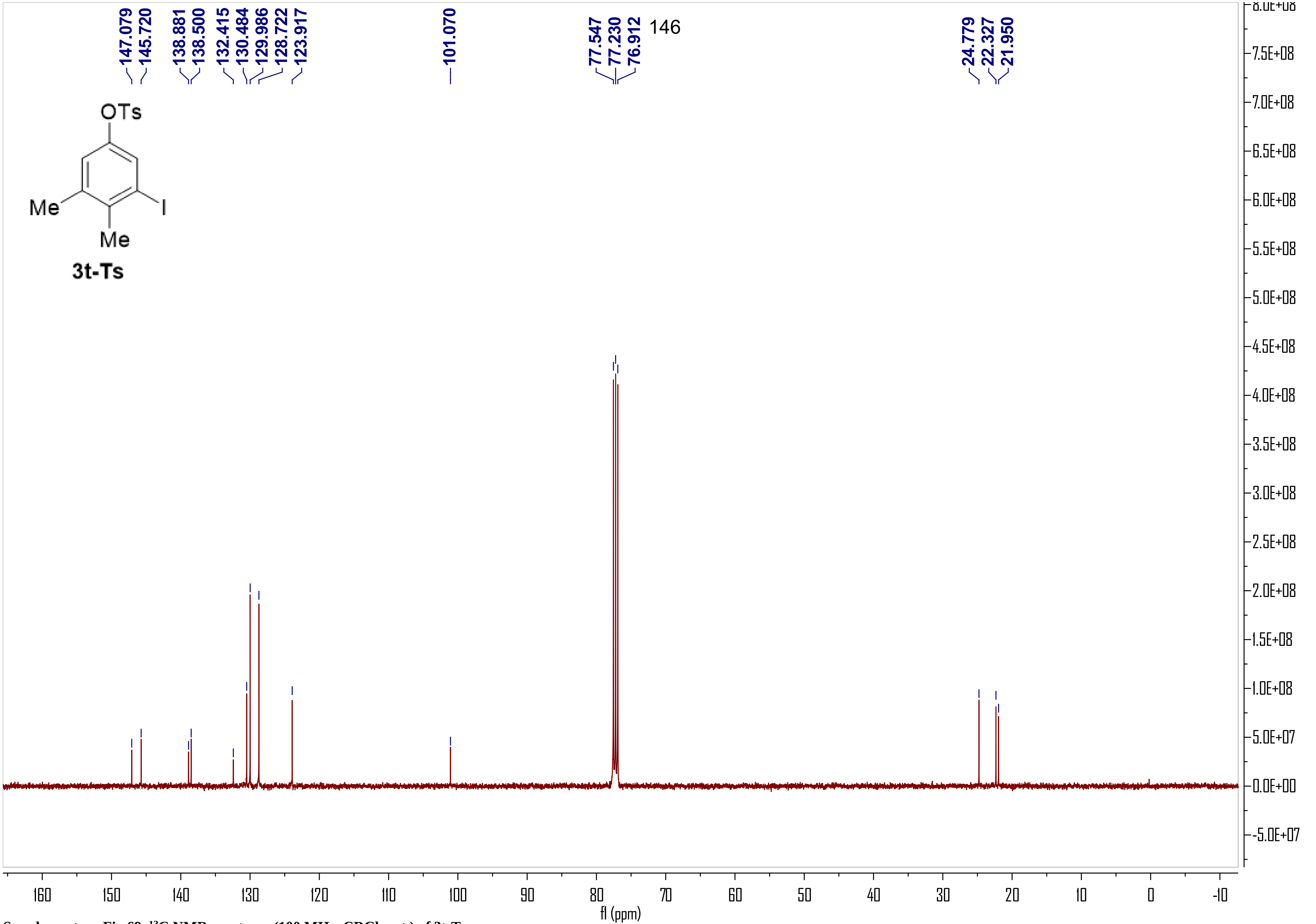

Supplementary Fig 69. ^{13}**C NMR spectrum (100 MHz, CDCl$_3$, r.t.) of 3t-Ts.**

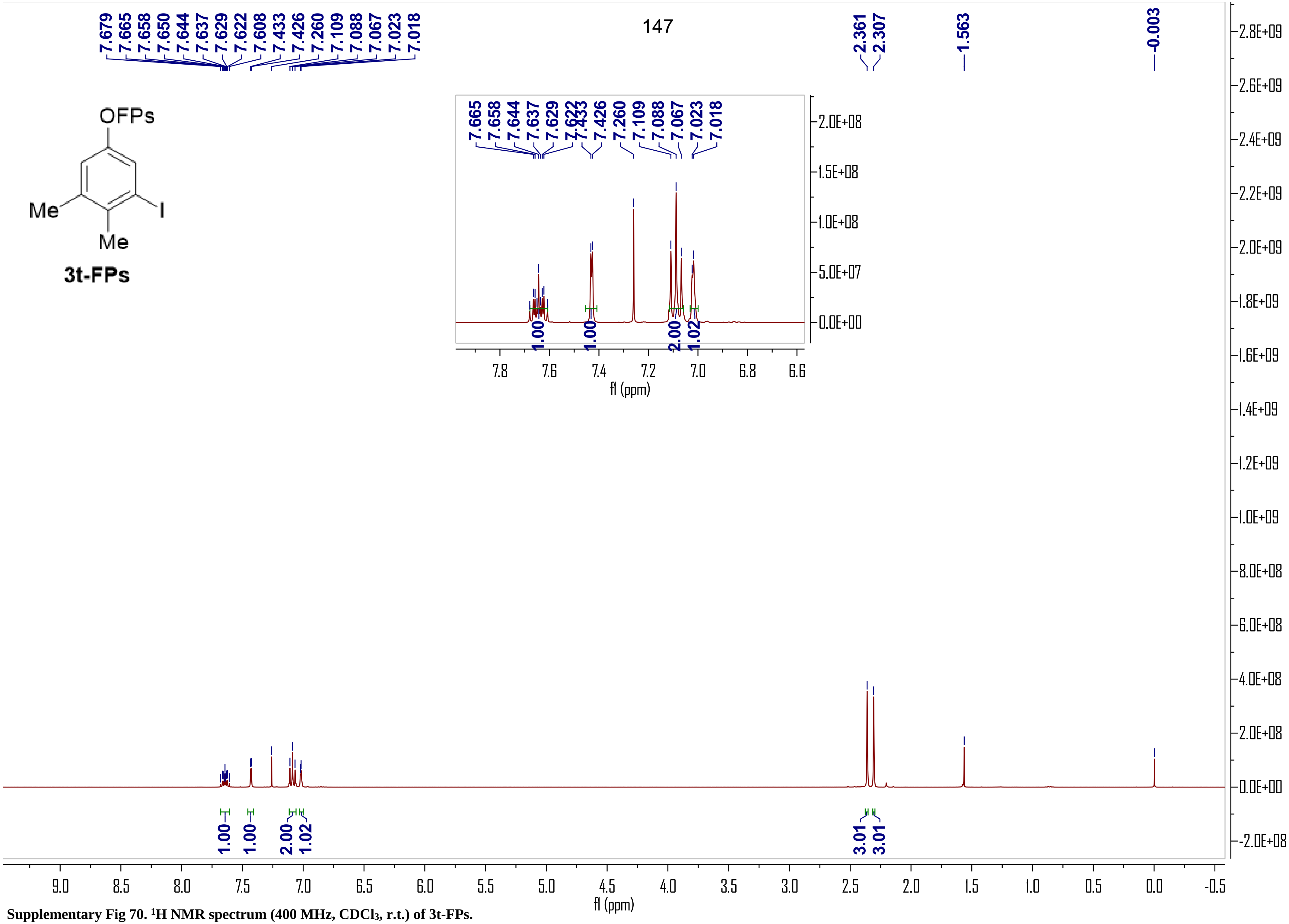

Supplementary Fig 70. ¹H NMR spectrum (400 MHz, $CDCl_3$, r.t.) of 3t-FPs.

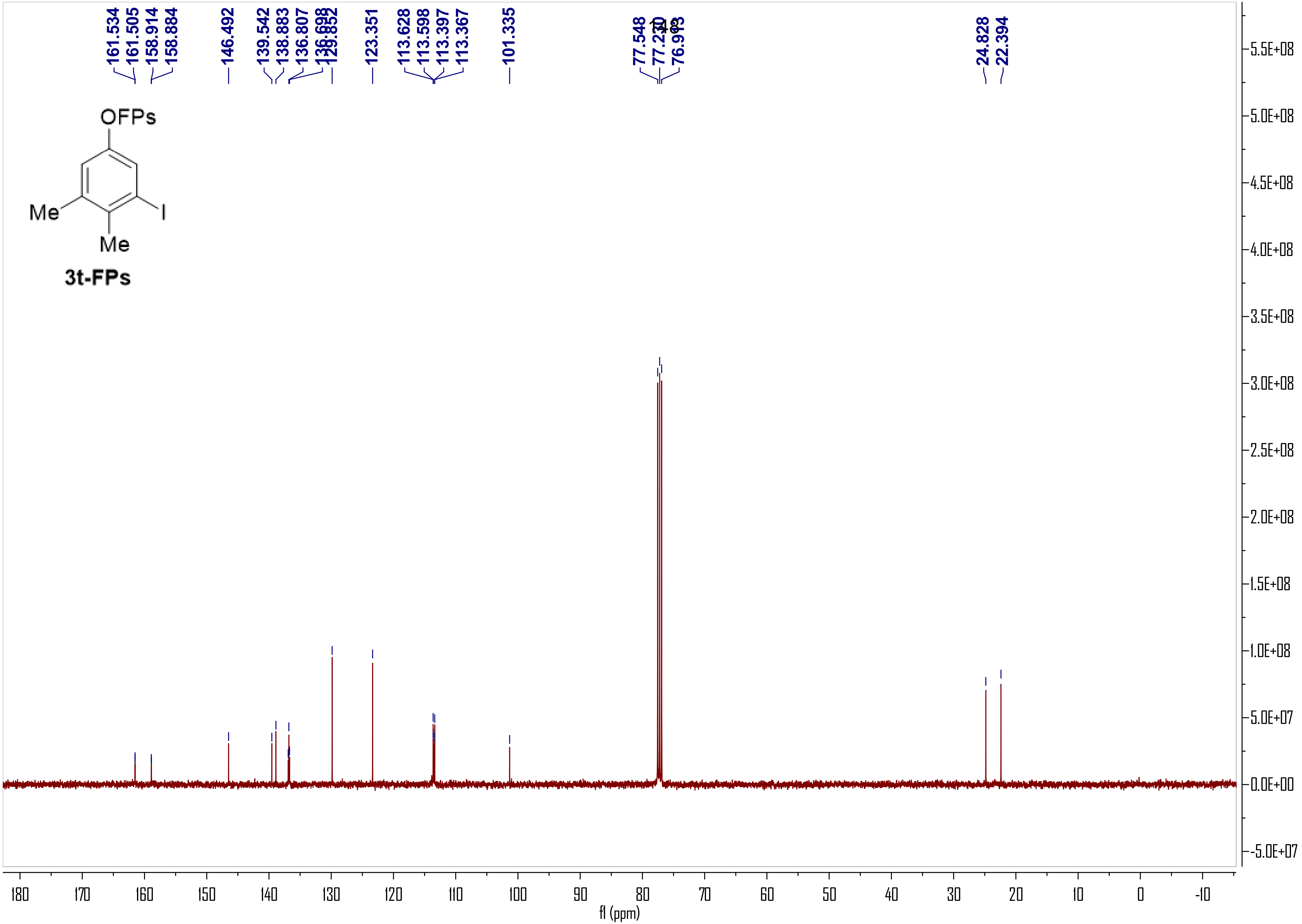

Supplementary Fig 71. ^{13}**C NMR spectrum (100 MHz, CDCl$_3$, r.t.) of 3t-FPs.**

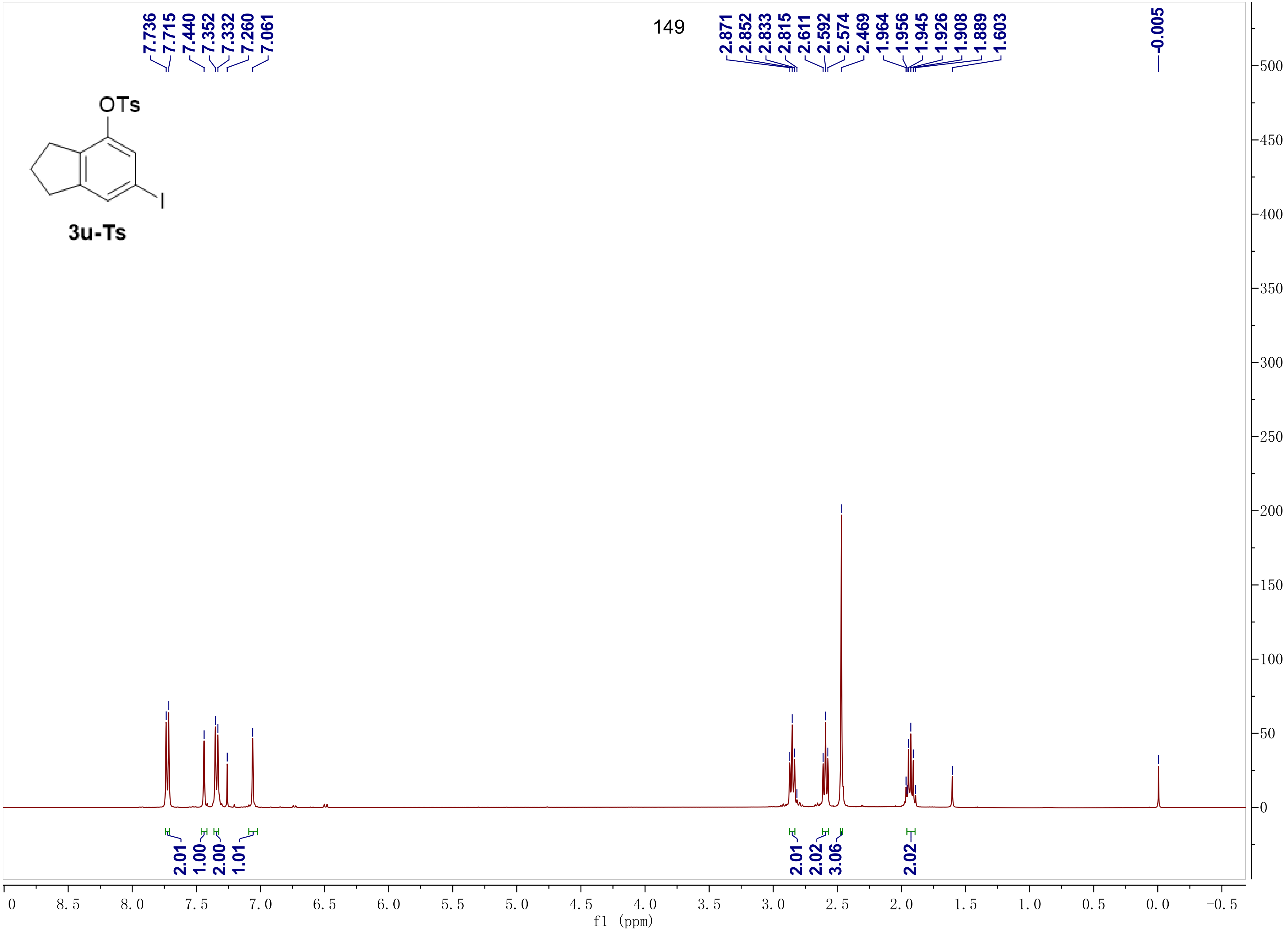

Supplementary Fig 72. ^{1}H NMR spectrum (400 MHz, $CDCl_3$, r.t.) of 3u-Ts.

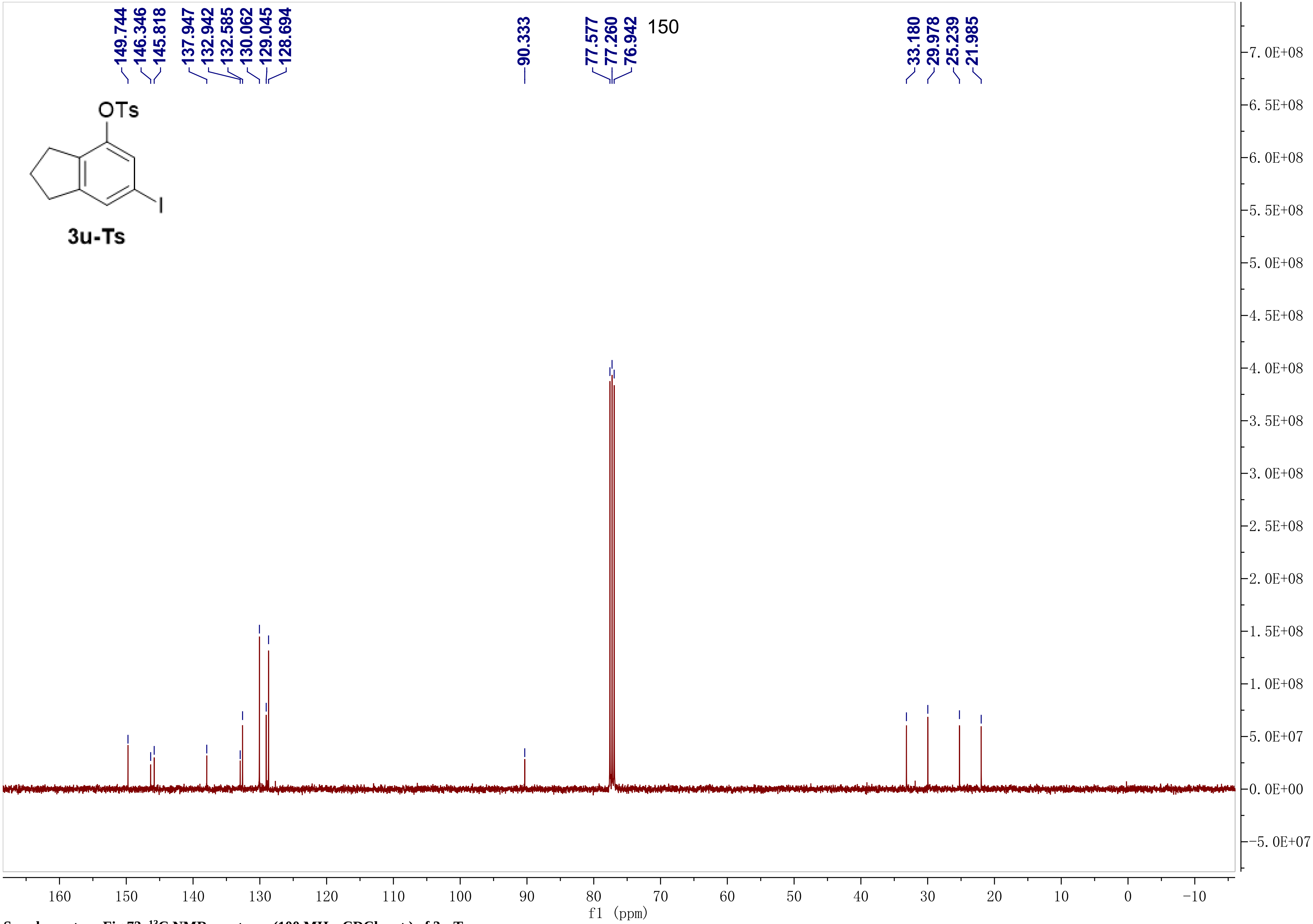

Supplementary Fig 73. ^{13}C NMR spectrum (100 MHz, $CDCl_3$, r.t.) of 3u-Ts.

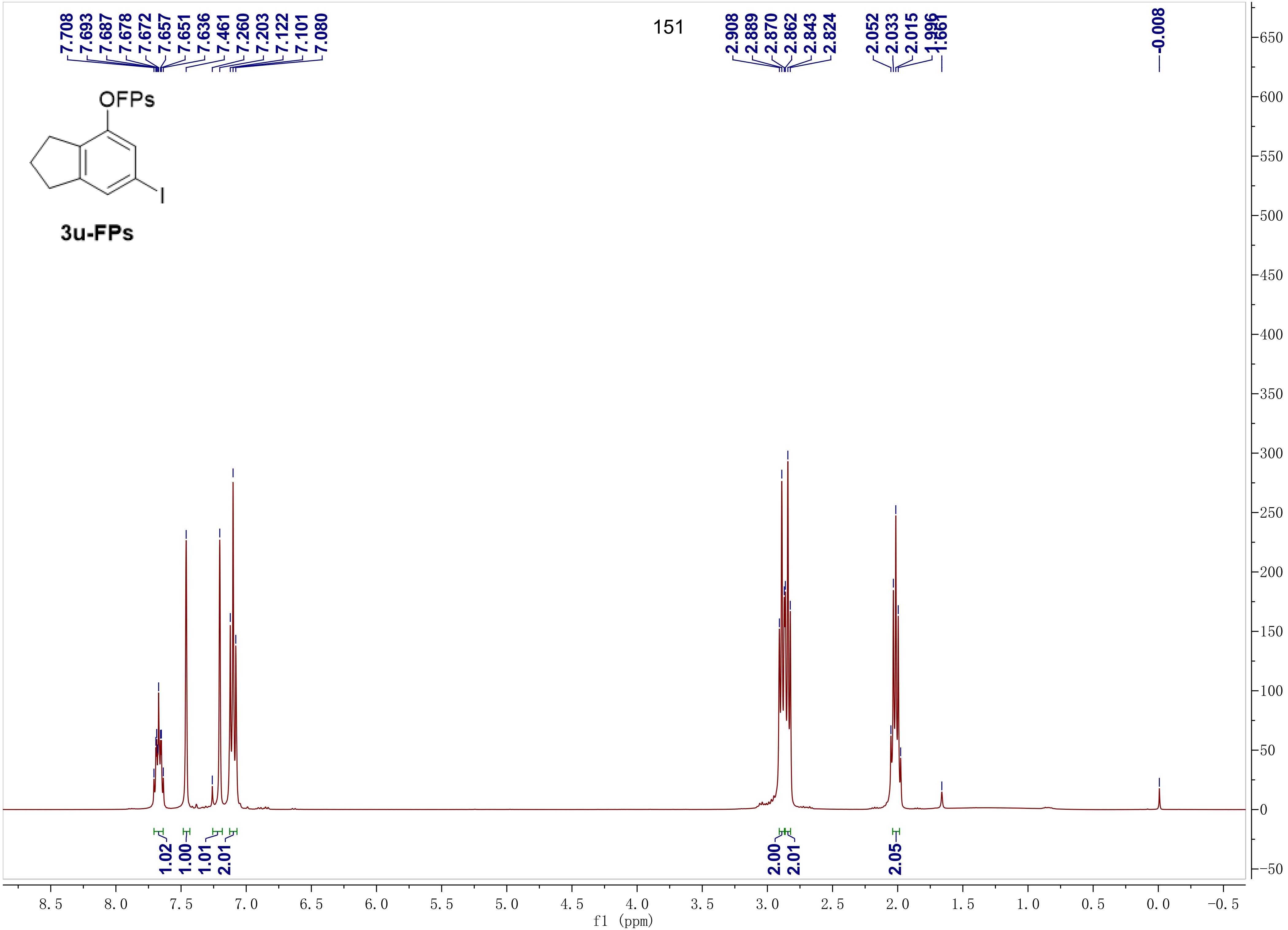

Supplementary Fig 74. ^{1}H NMR spectrum (400 MHz, $CDCl_3$, r.t.) of 3u-FPs.

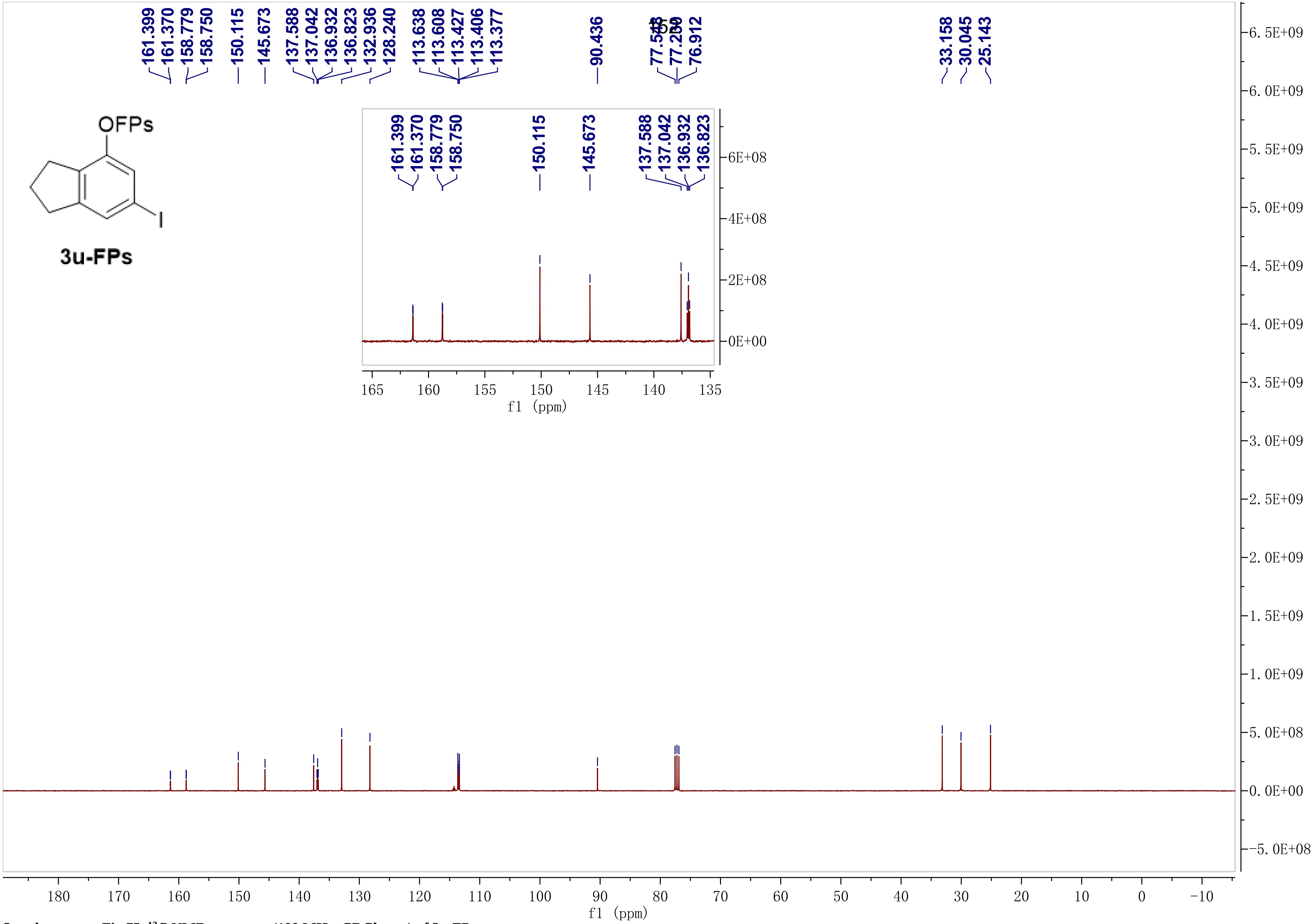

Supplementary Fig 75. ^{13}C NMR spectrum (100 MHz, $CDCl_3$, r.t.) of 3u-FPs.

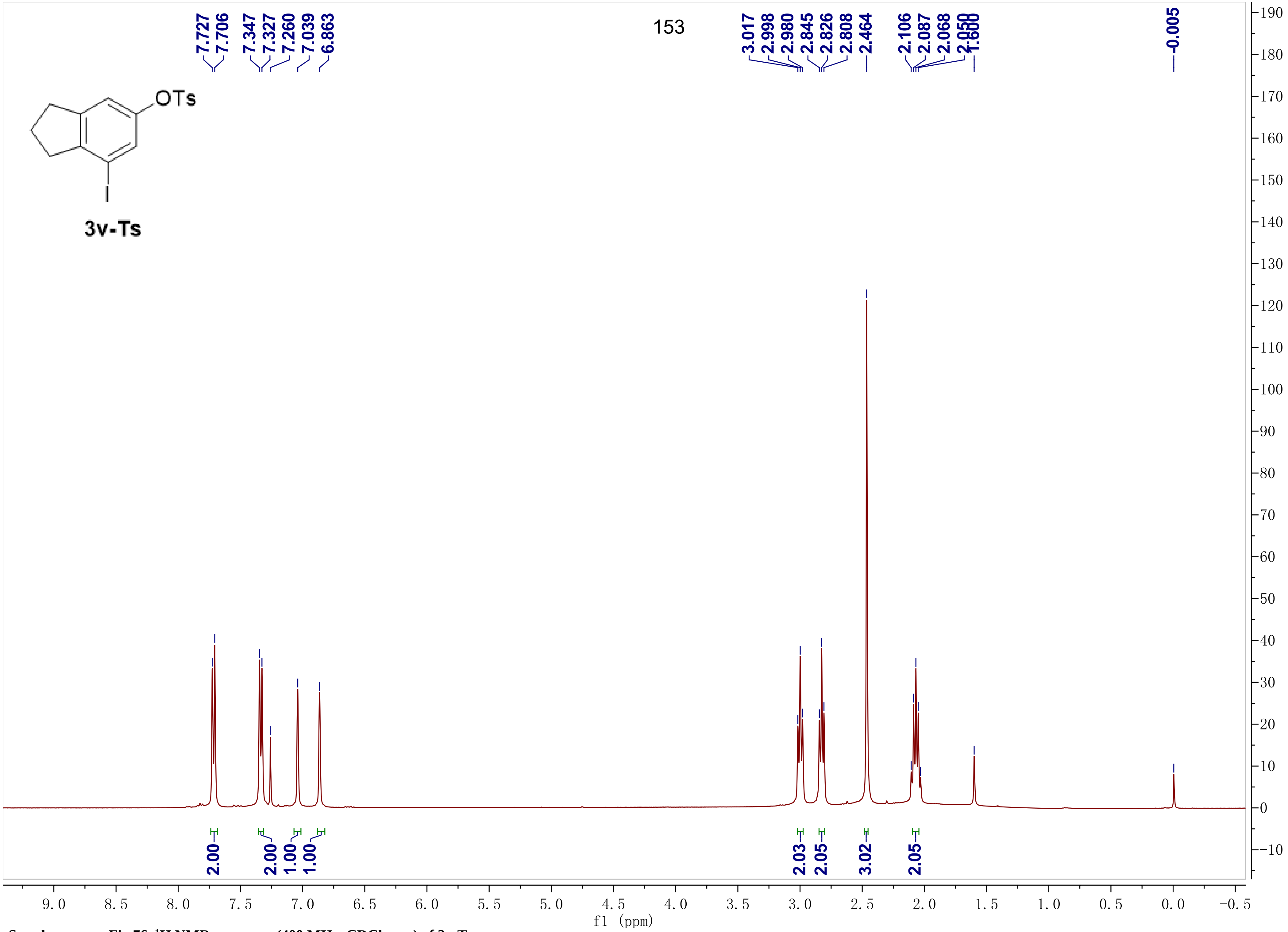

Supplementary Fig 76. **^{1}H NMR spectrum (400 MHz, $CDCl_3$, r.t.) of 3v-Ts.**

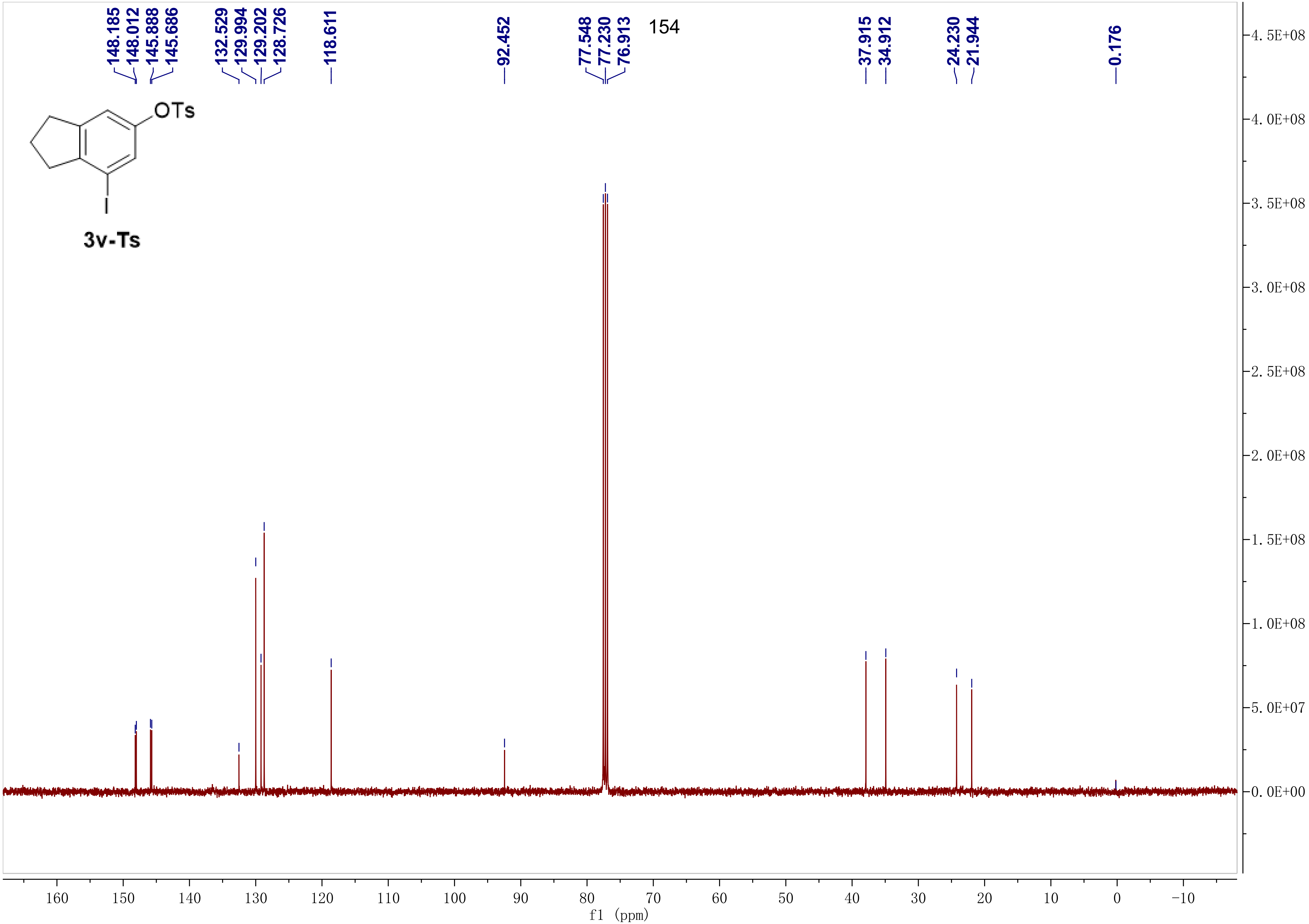

Supplementary Fig 77. ^{13}C NMR spectrum (100 MHz, $CDCl_3$, r.t.) of 3v-Ts.

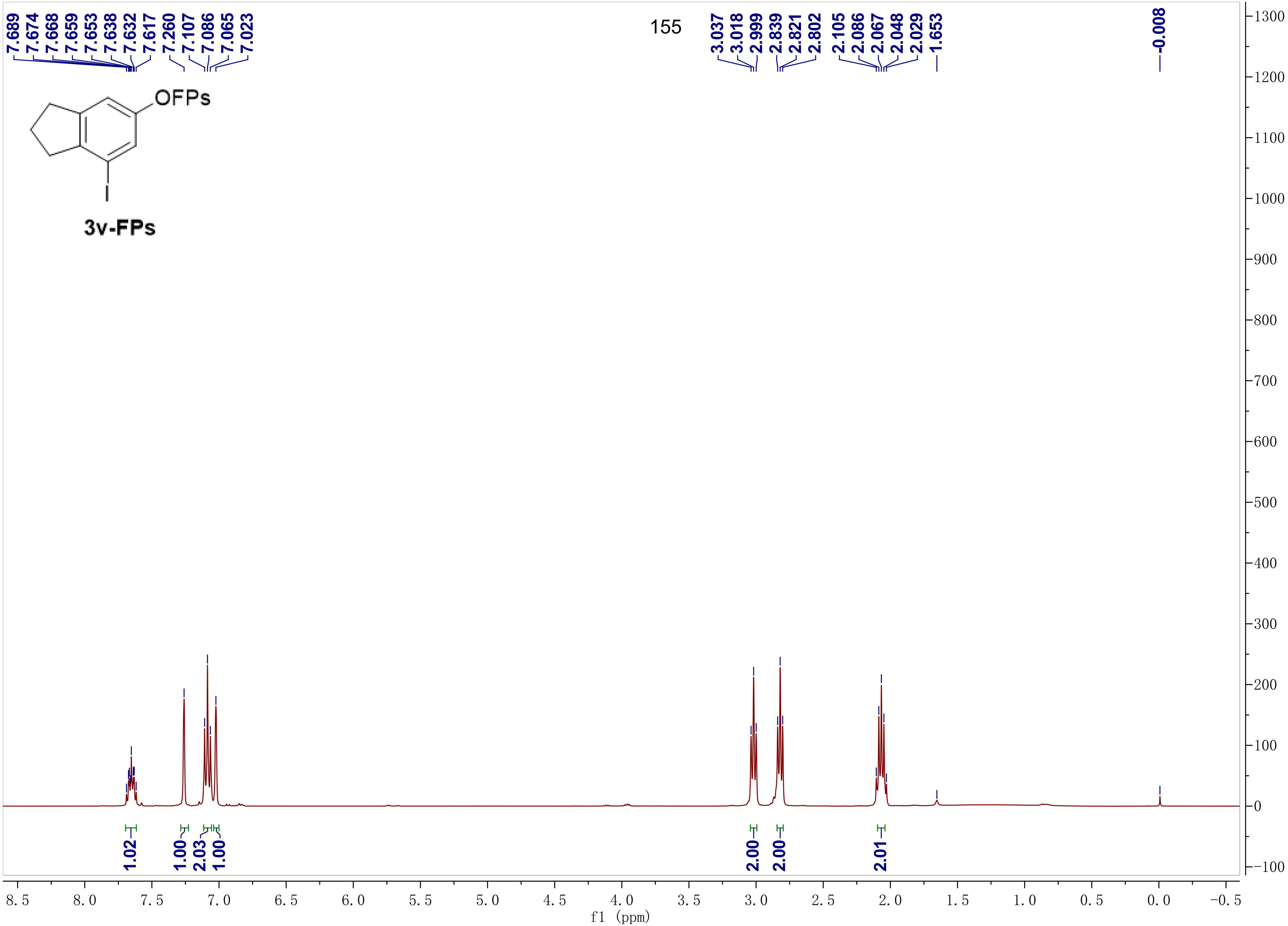

Supplementary Fig 78. **^{1}H NMR spectrum (400 MHz, $CDCl_3$, r.t.) of 3v-FPs.**

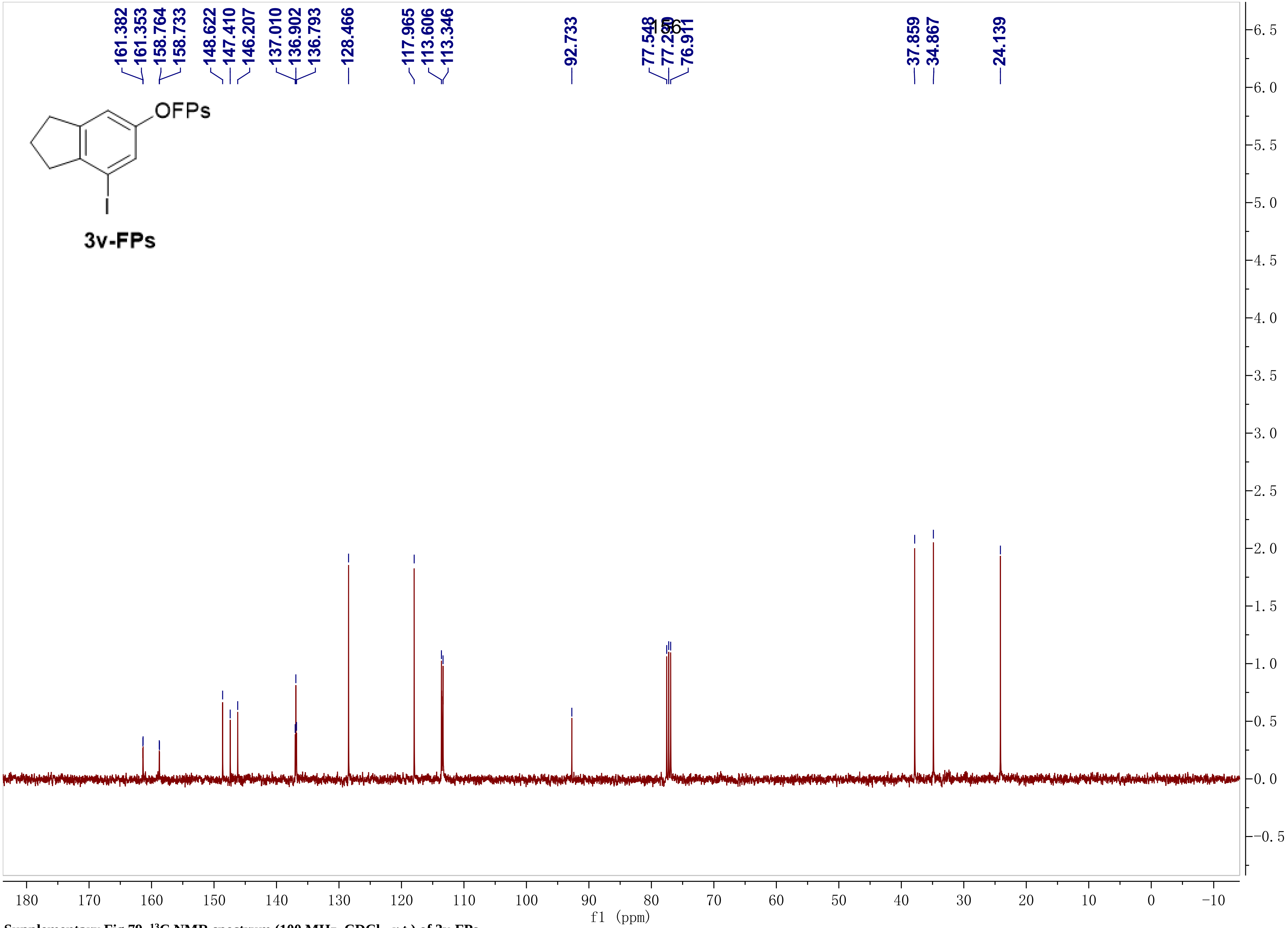

Supplementary Fig 79. ^{13}**C NMR spectrum (100 MHz, CDCl$_3$, r.t.) of 3v-FPs.**

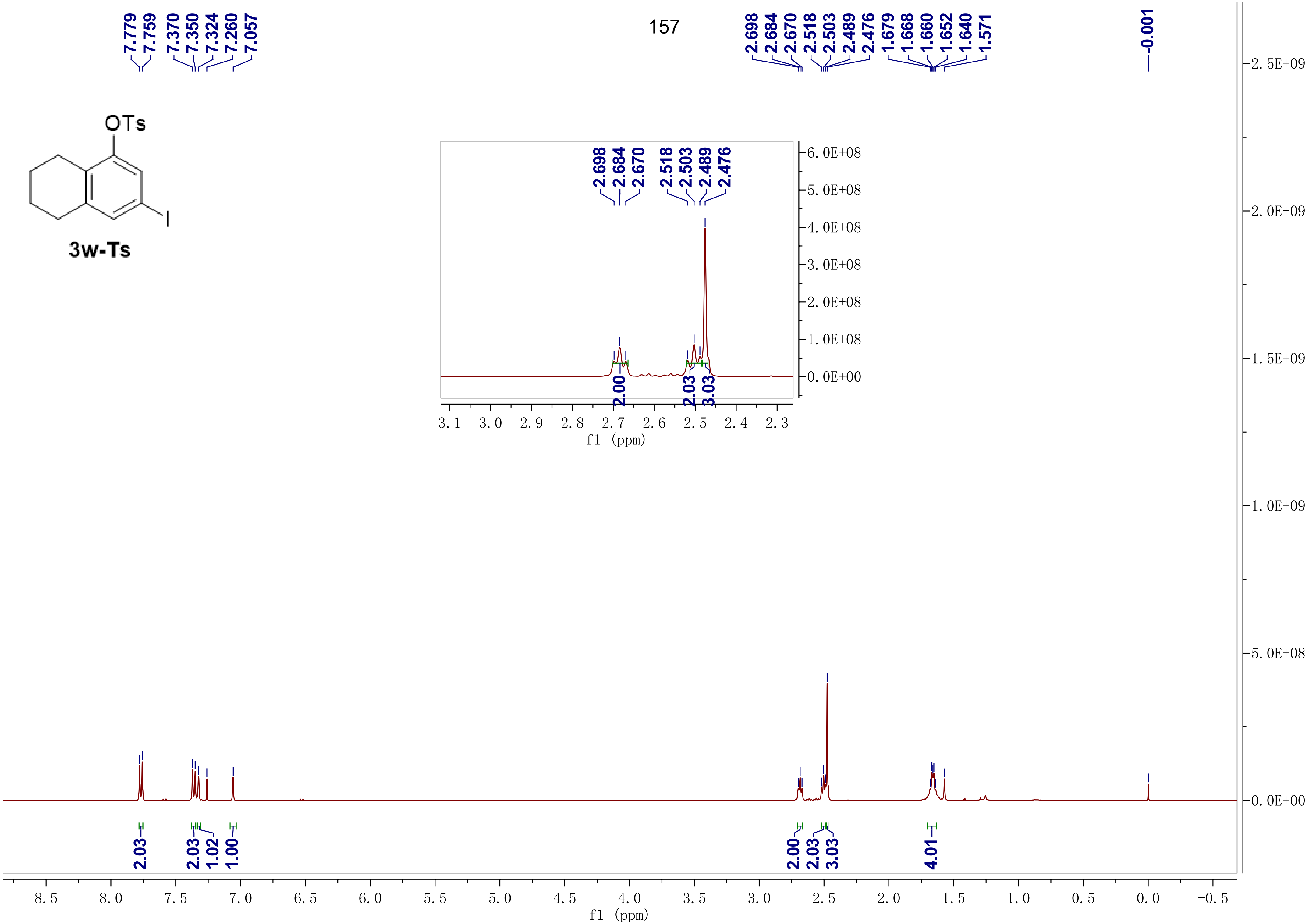

Supplementary Fig 80. ^{1}**H NMR spectrum (400 MHz, CDCl$_3$, r.t.) of 3w-Ts.**

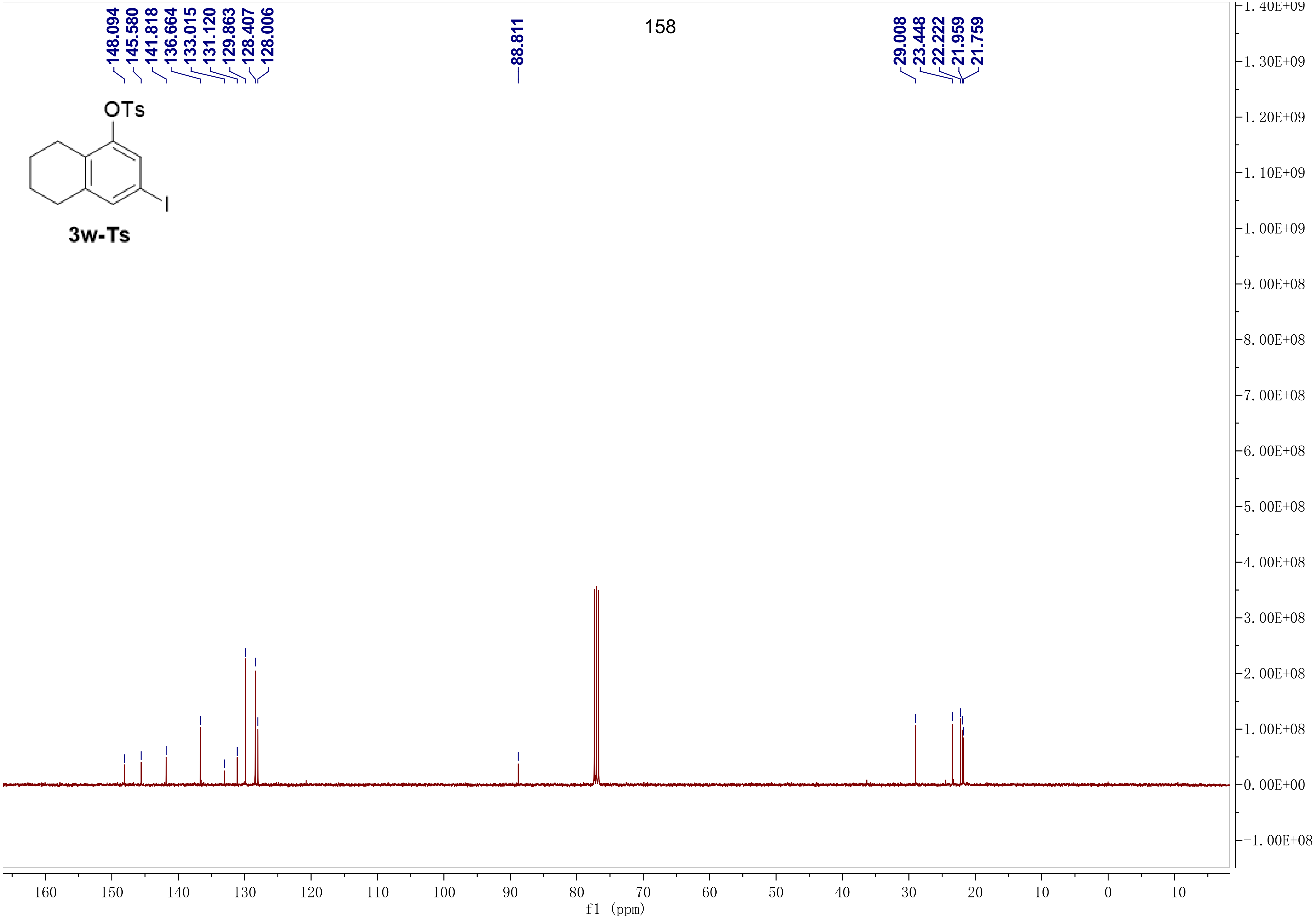

Supplementary Fig 81. ^{13}**C NMR spectrum (100 MHz, CDCl$_3$, r.t.) of 3w-Ts.**

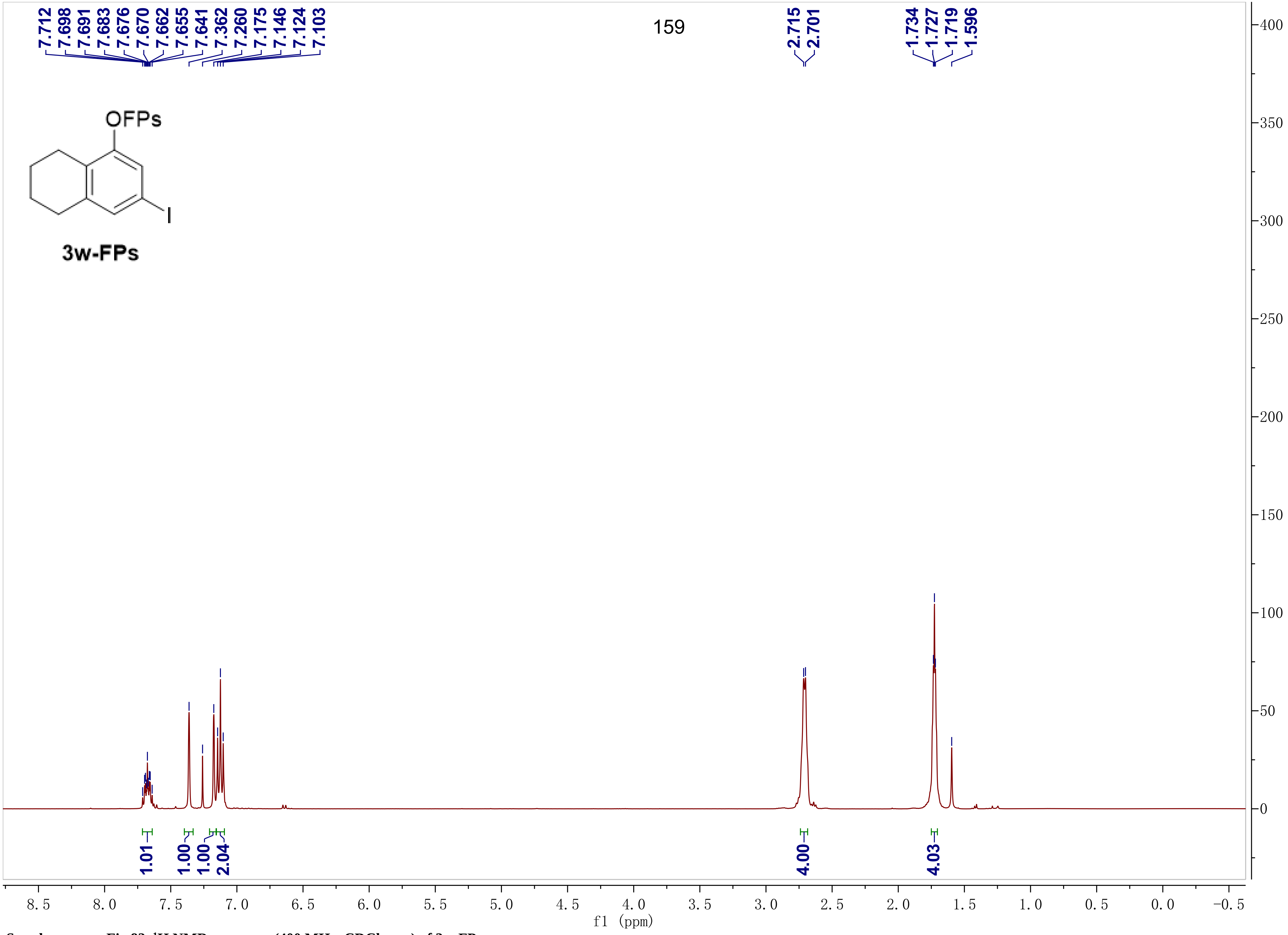

Supplementary Fig 82. ¹H NMR spectrum (400 MHz, $CDCl_3$, r.t.) of 3w-FPs.

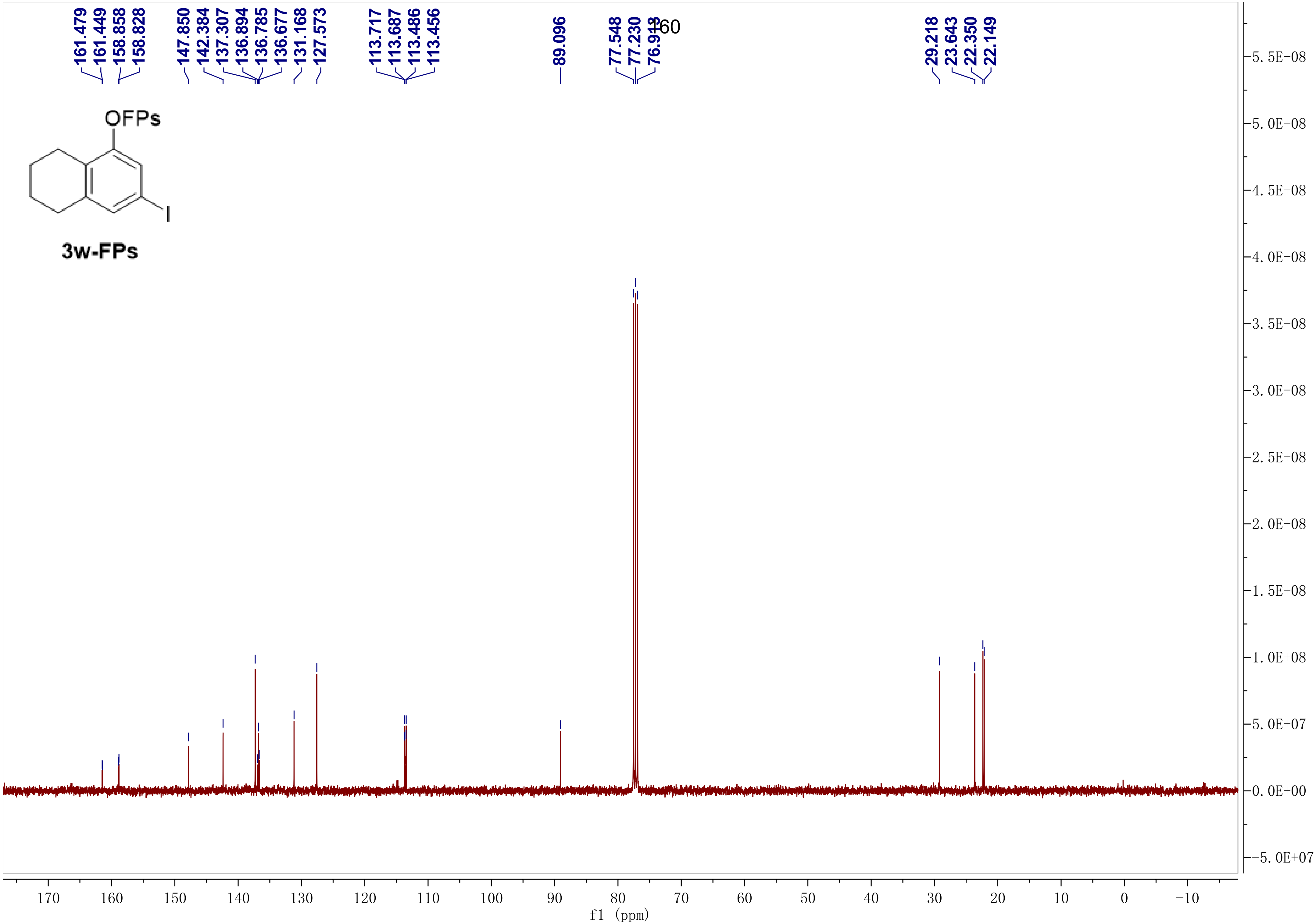

Supplementary Fig 83. ^{13}C NMR spectrum (100 MHz, $CDCl_3$, r.t.) of 3w-FPs.

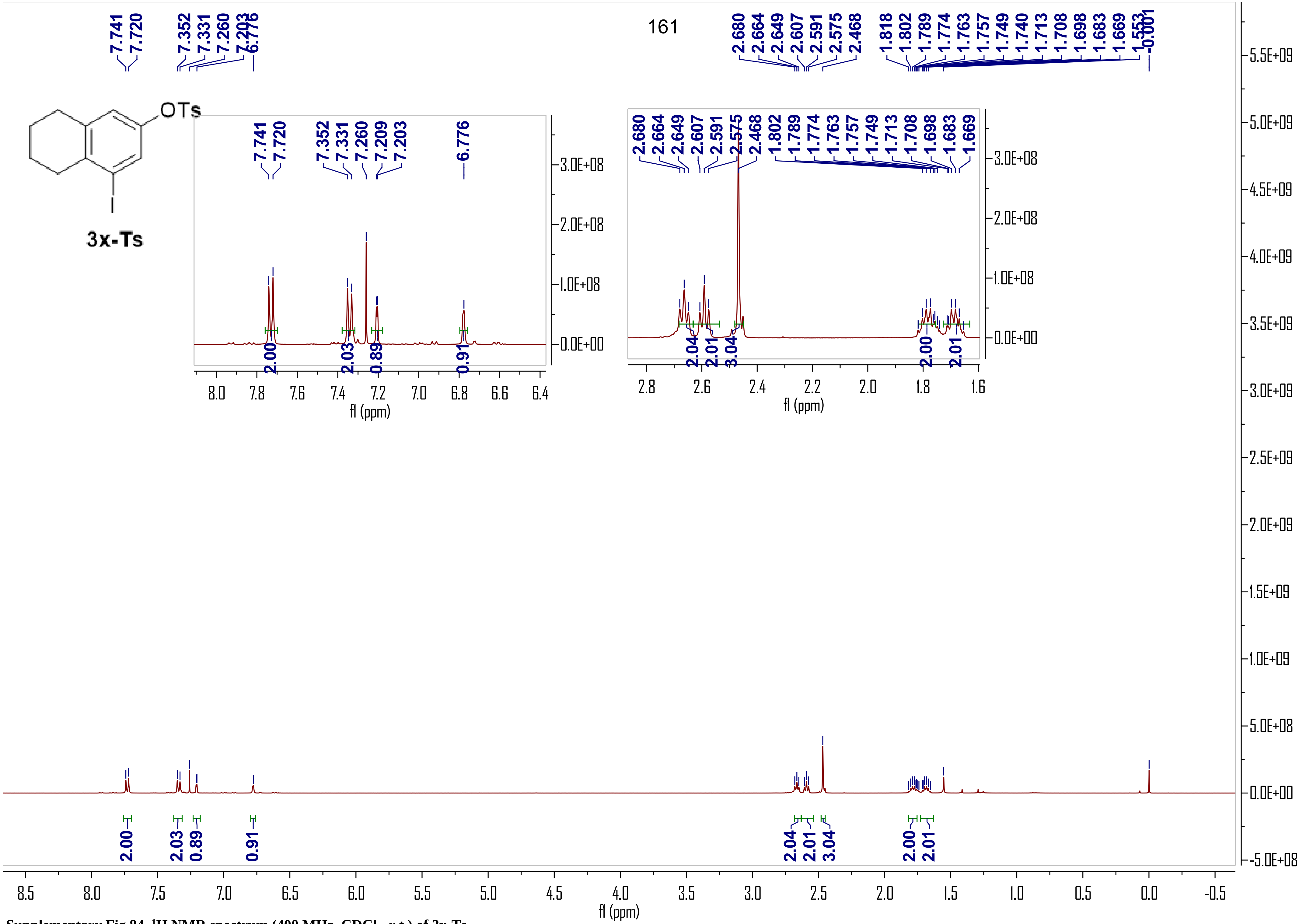

Supplementary Fig 84. ^{1}H NMR spectrum (400 MHz, $CDCl_3$, r.t.) of 3x-Ts.

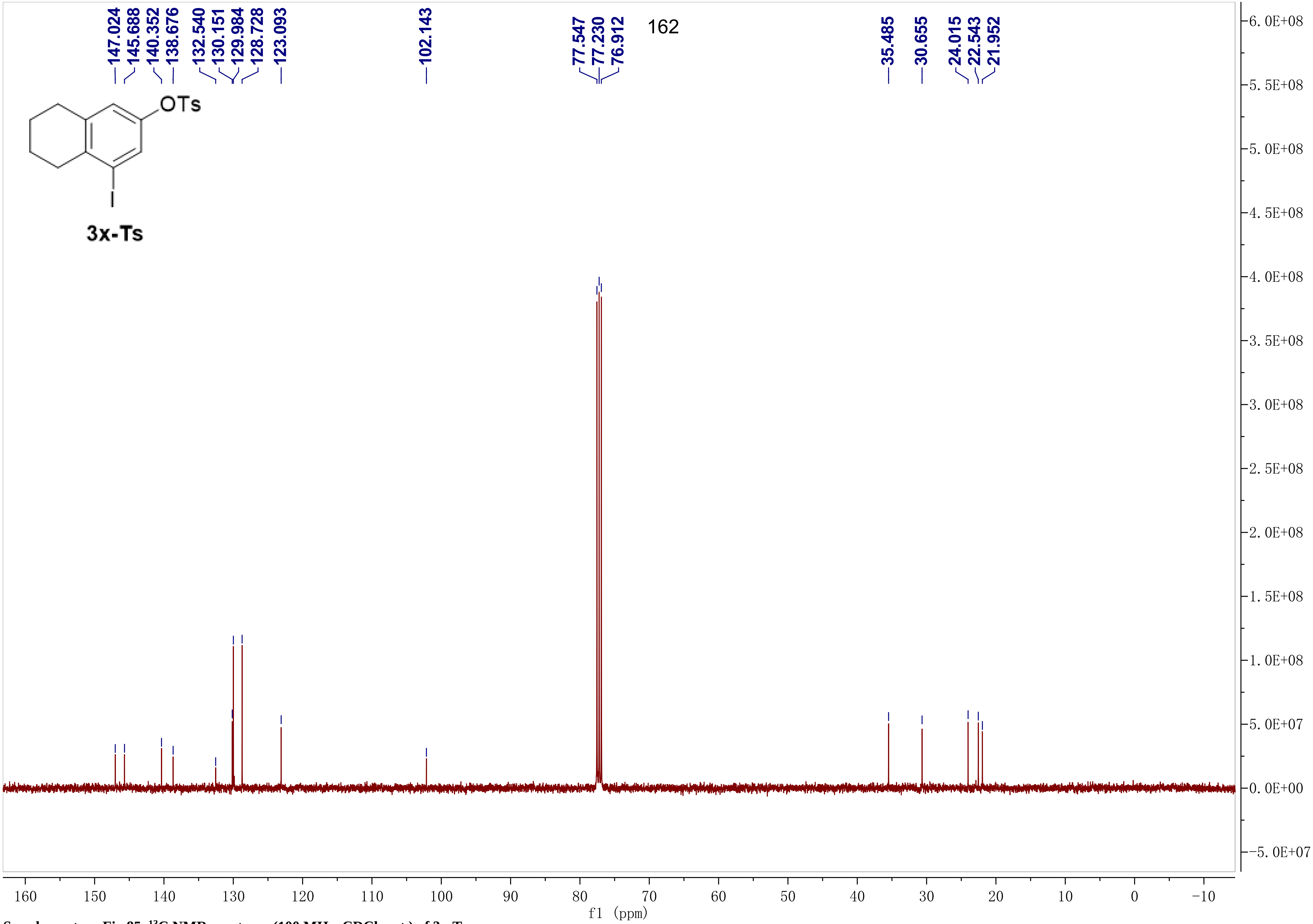

Supplementary Fig 85. ^{13}C NMR spectrum (100 MHz, $CDCl_3$, r.t.) of 3x-Ts.

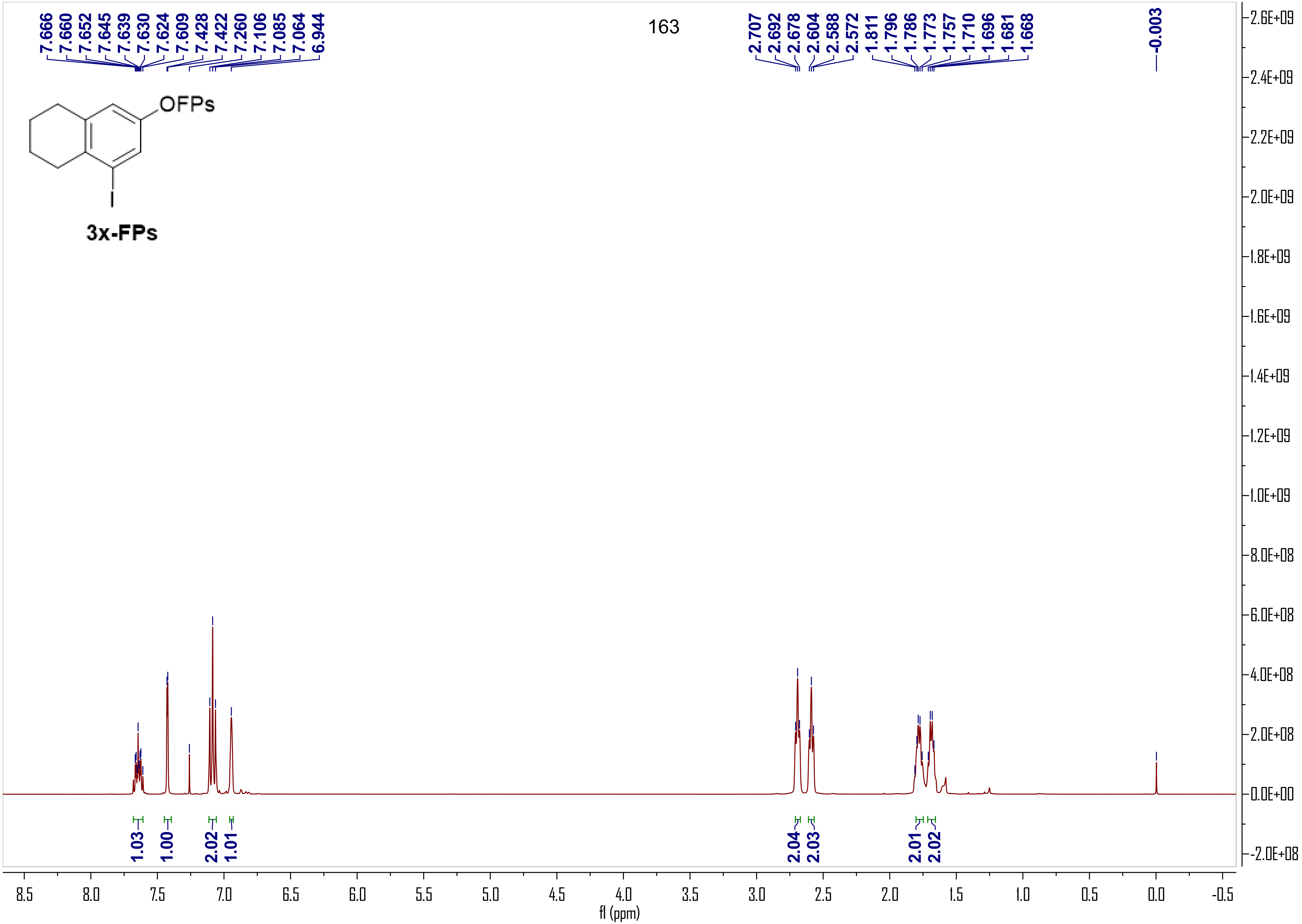

Supplementary Fig 86. **^{1}H NMR spectrum (400 MHz, $CDCl_3$, r.t.) of 3x-FPs.**

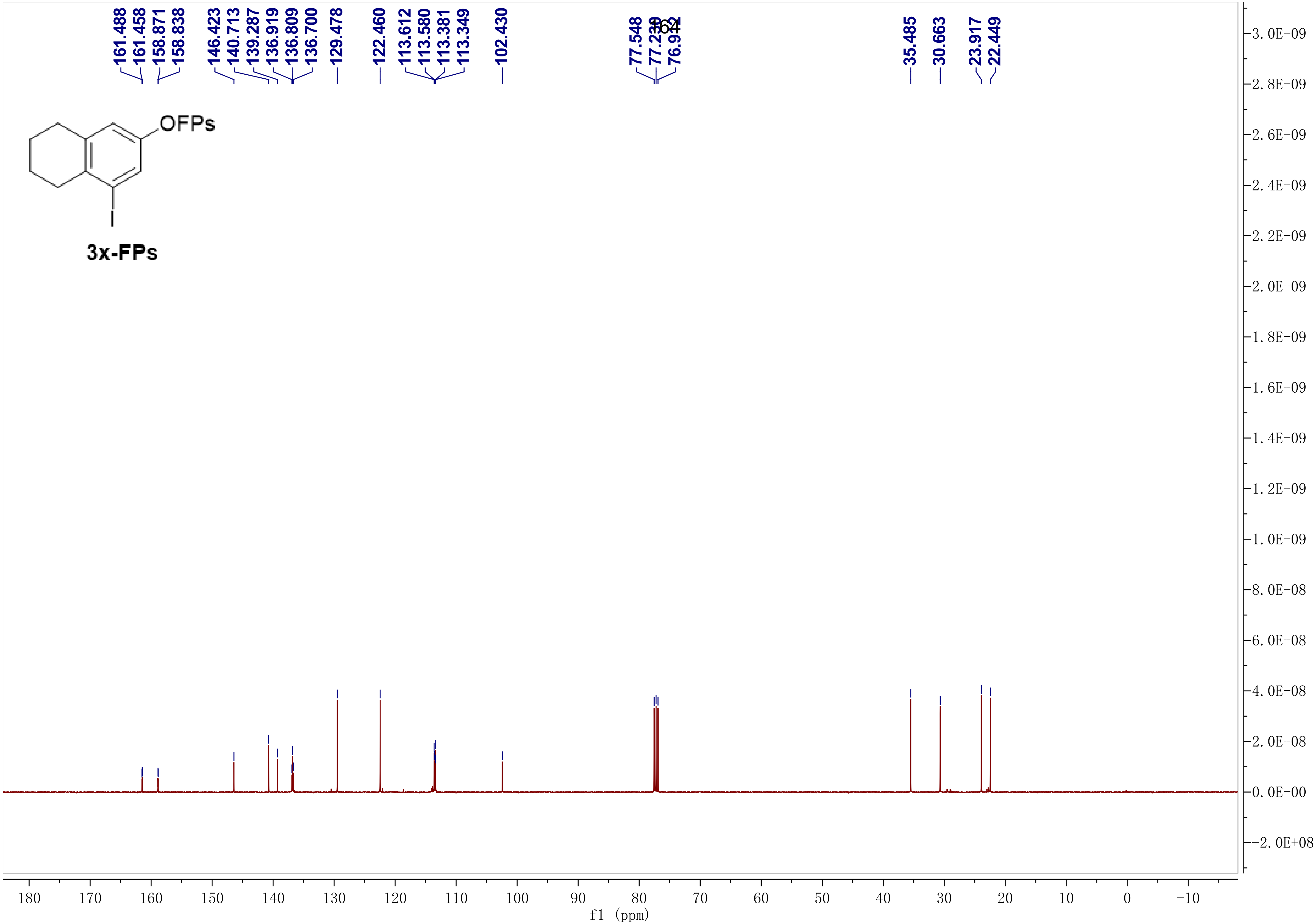

Supplementary Fig 87. ^{13}C NMR spectrum (100 MHz, $CDCl_3$, r.t.) of 3x-FPs.

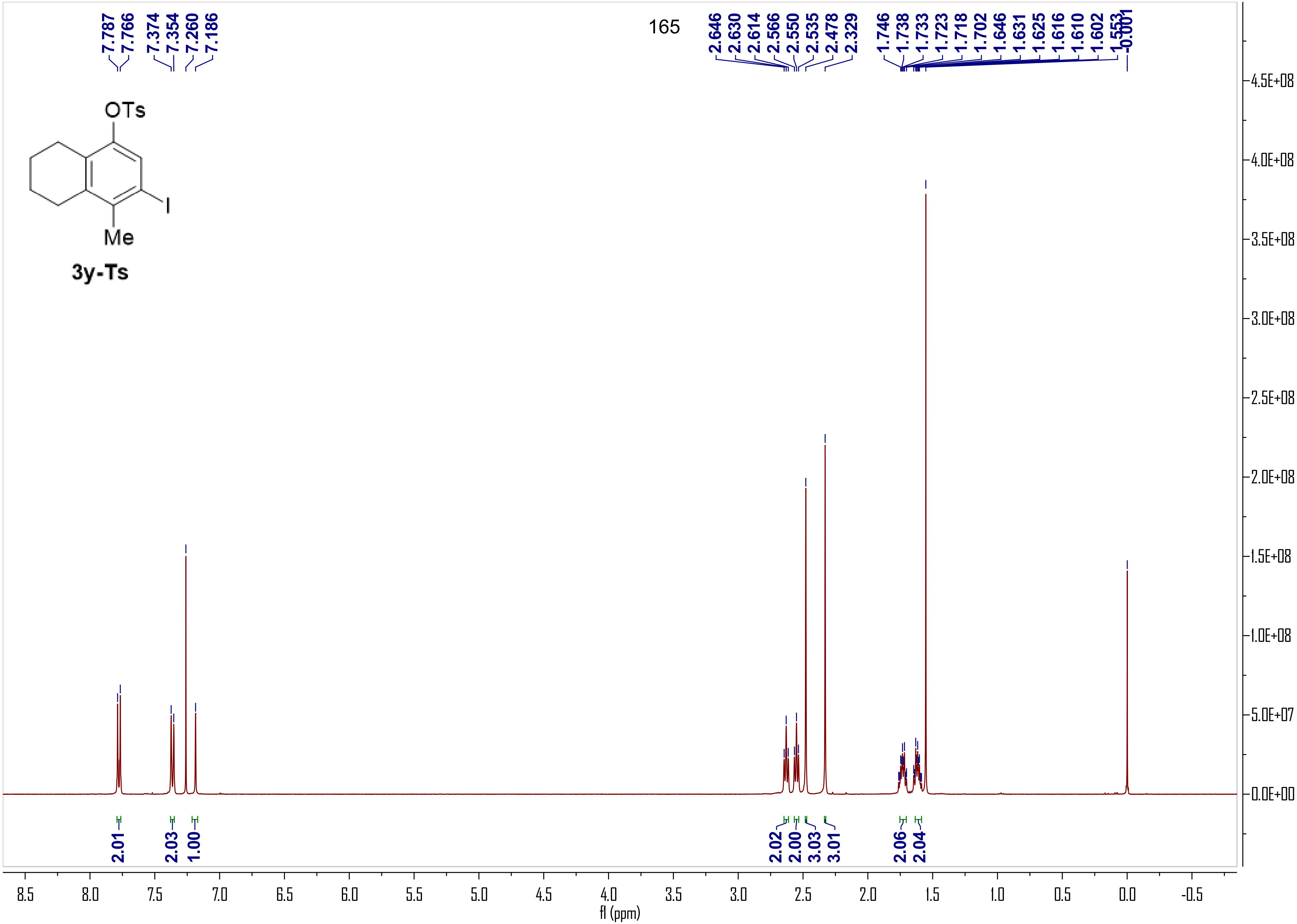

Supplementary Fig 88. **^{1}H NMR spectrum (400 MHz, $CDCl_3$, r.t.) of 3y-Ts.**

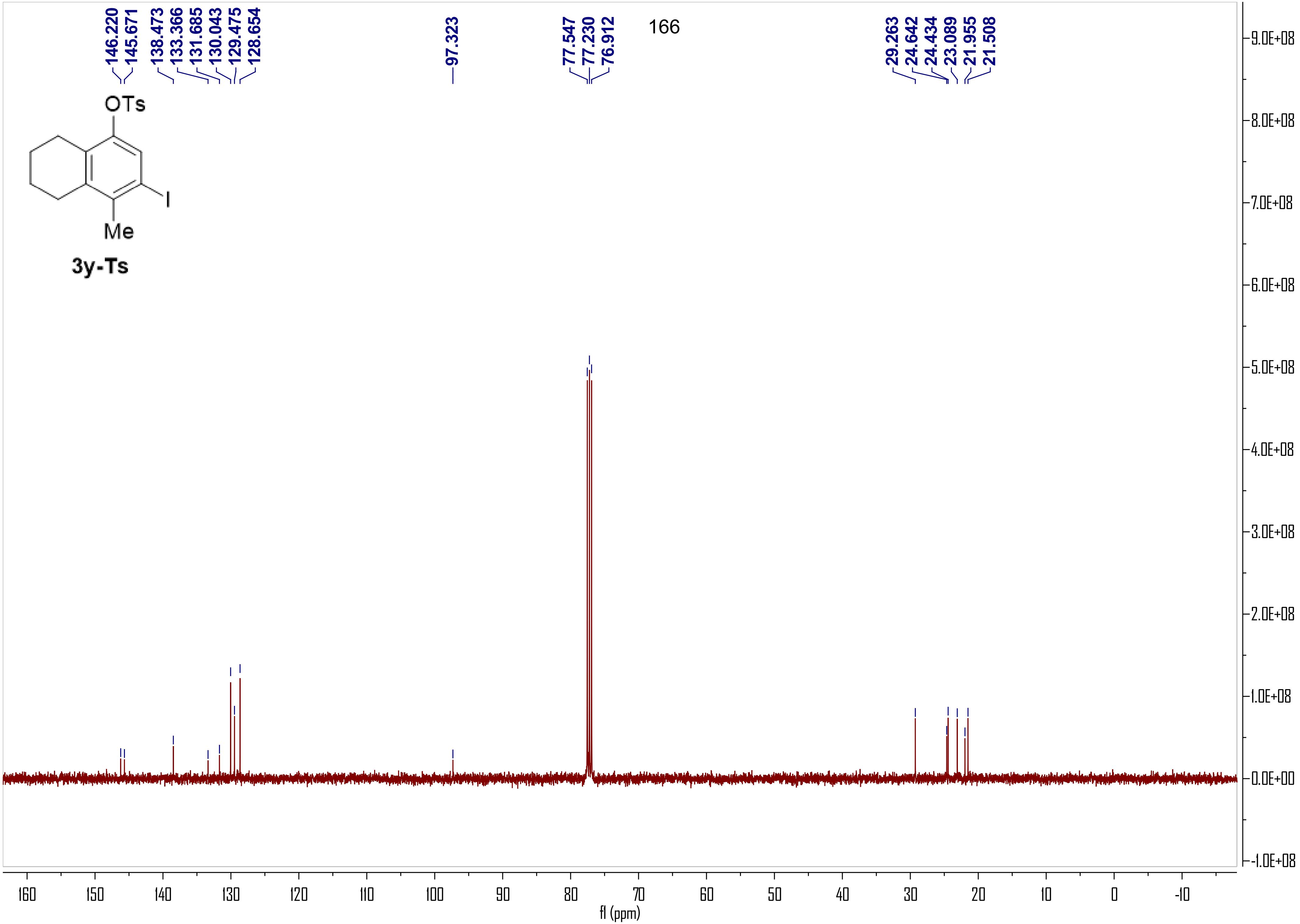

Supplementary Fig 89. ^{13}C **NMR spectrum (100 MHz, $CDCl_3$, r.t.) of 3y-Ts.**

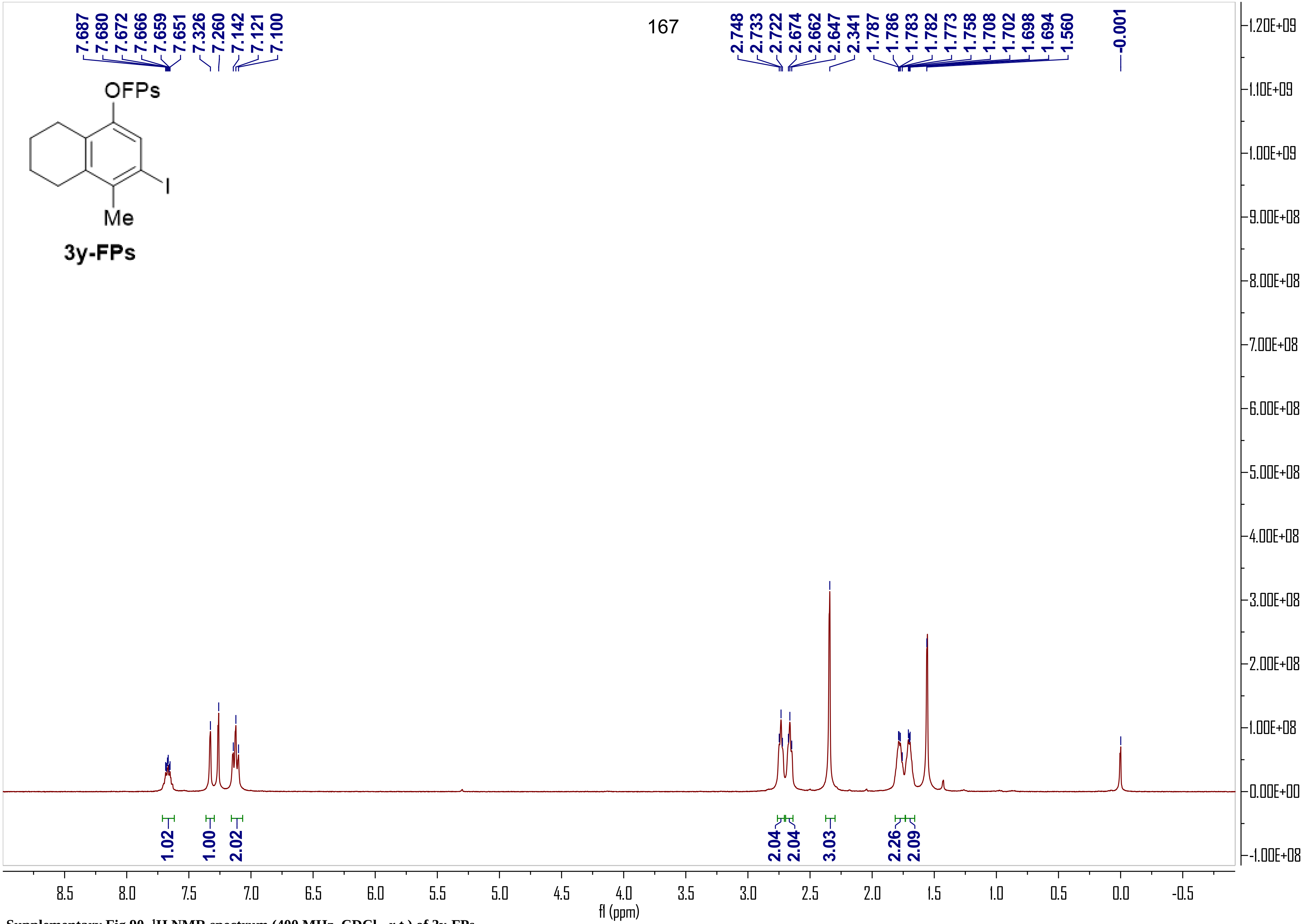

Supplementary Fig 90. ^{1}H NMR spectrum (400 MHz, $CDCl_3$, r.t.) of 3y-FPs.

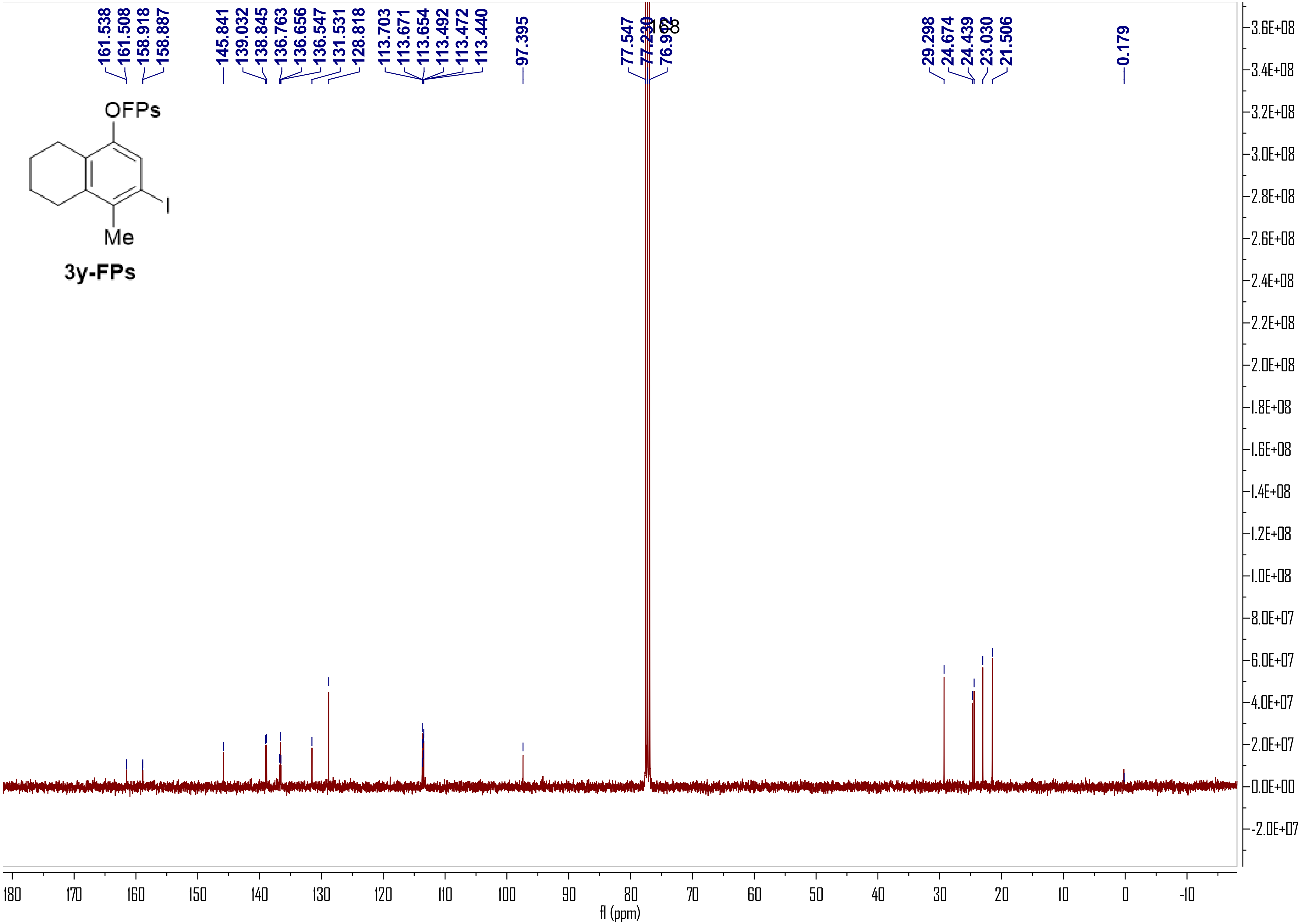

Supplementary Fig 91. ^{13}C **NMR spectrum (100 MHz, $CDCl_3$, r.t.) of 3y-FPs.**

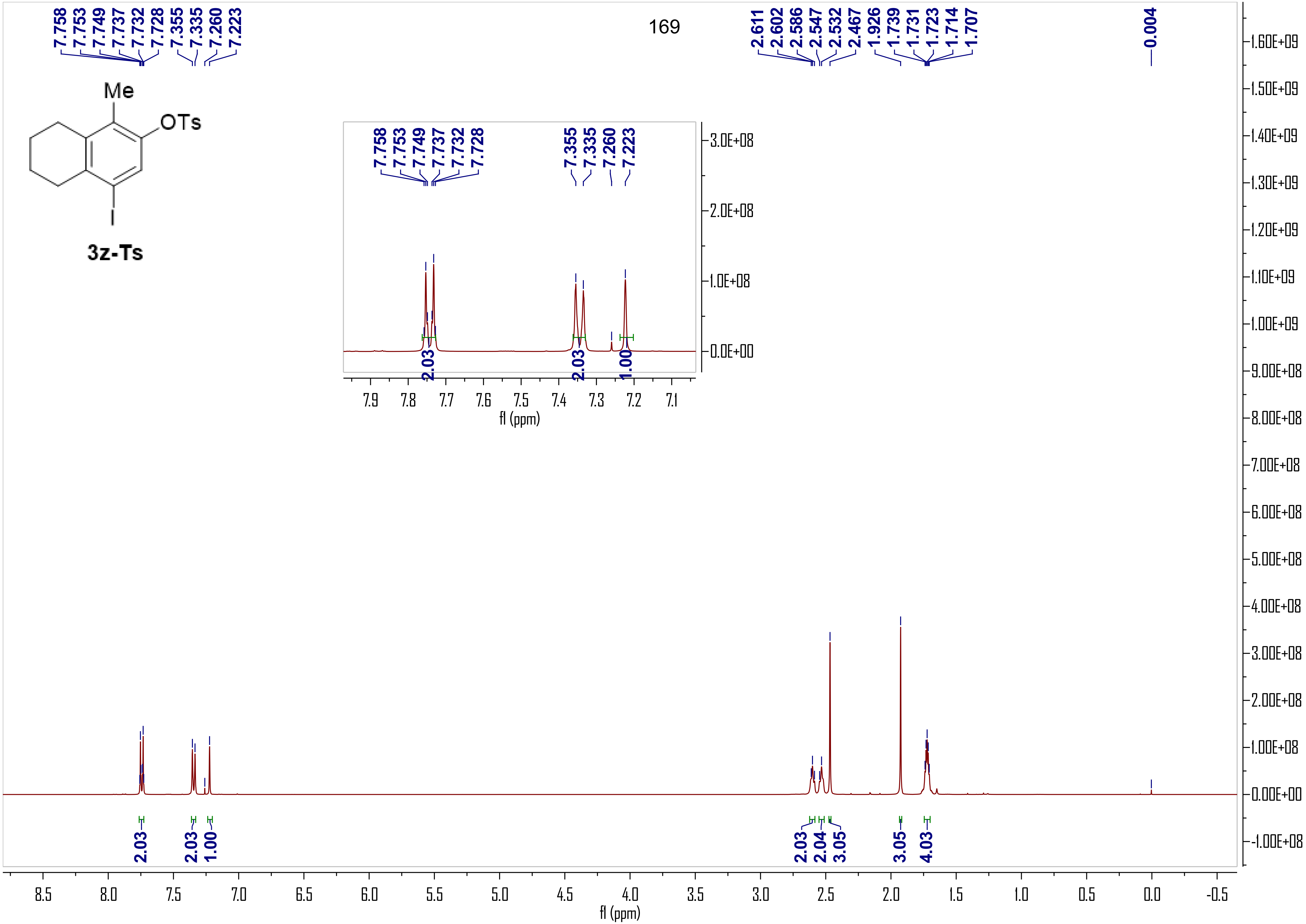

Supplementary Fig 92. ^{1}H NMR spectrum (400 MHz, $CDCl_3$, r.t.) of 3z-Ts.

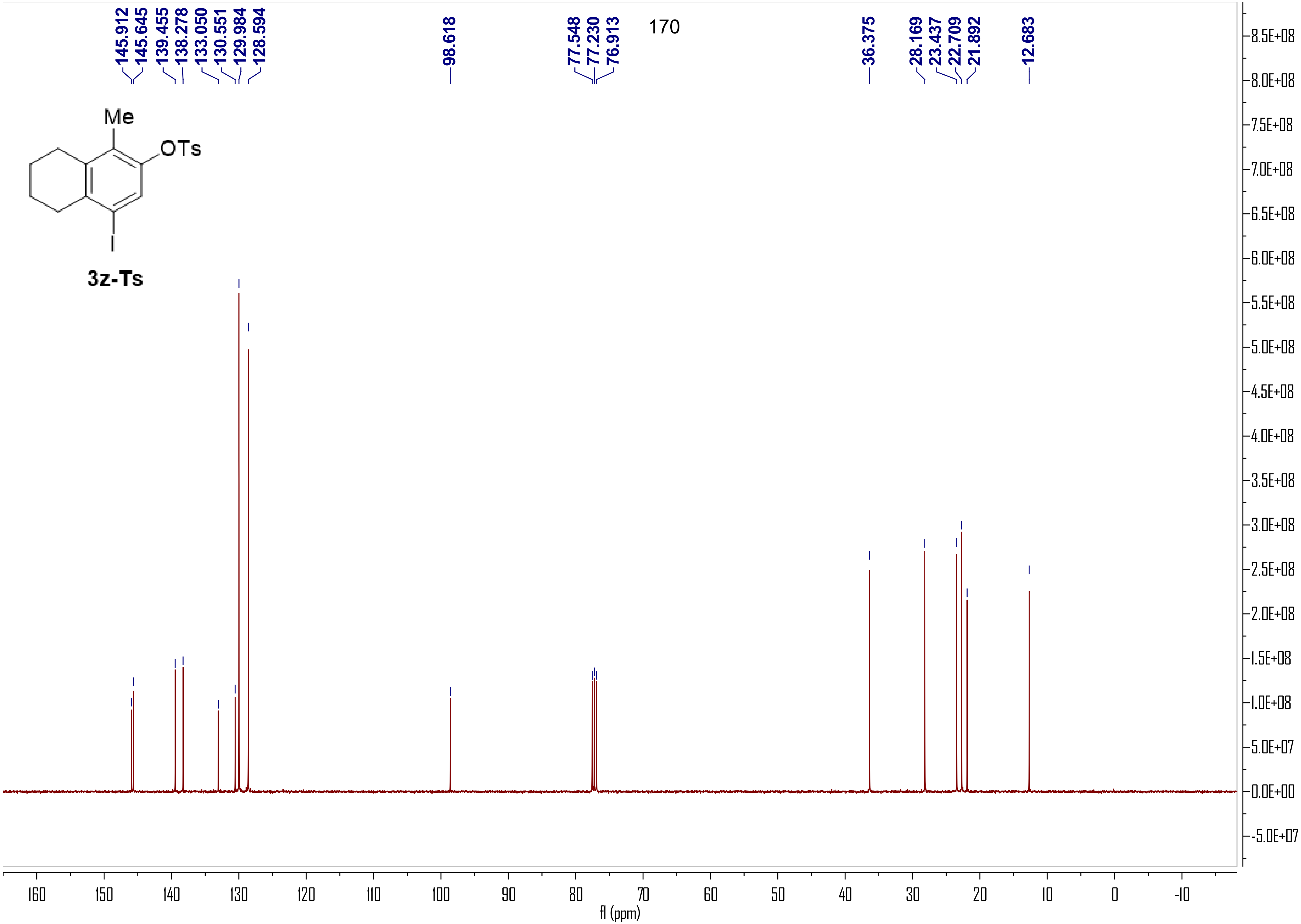

Supplementary Fig 93. ^{13}C NMR spectrum (100 MHz, $CDCl_3$, r.t.) of 3z-Ts.

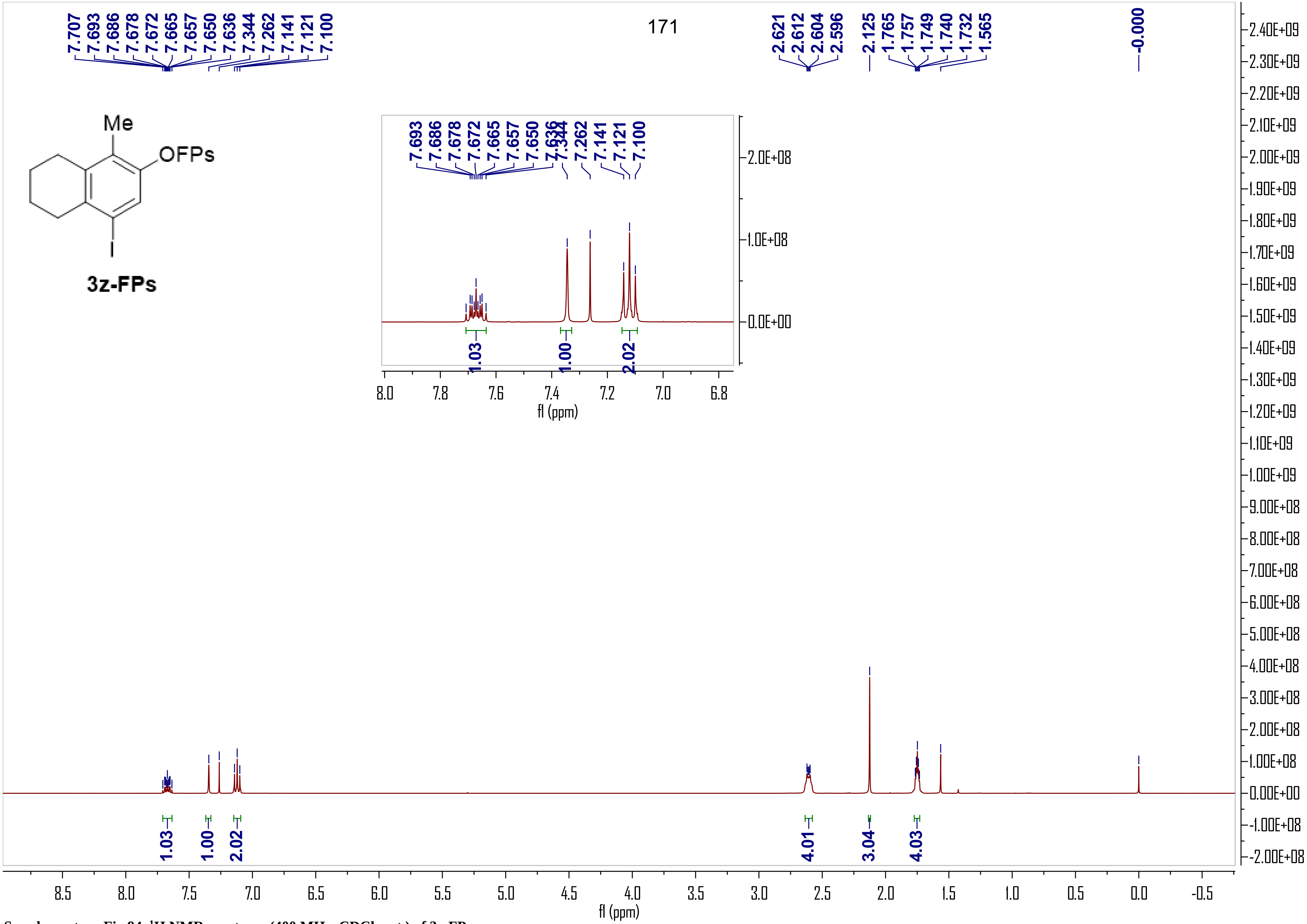

Supplementary Fig 94. **^{1}H NMR spectrum (400 MHz, $CDCl_3$, r.t.) of 3z-FPs.**

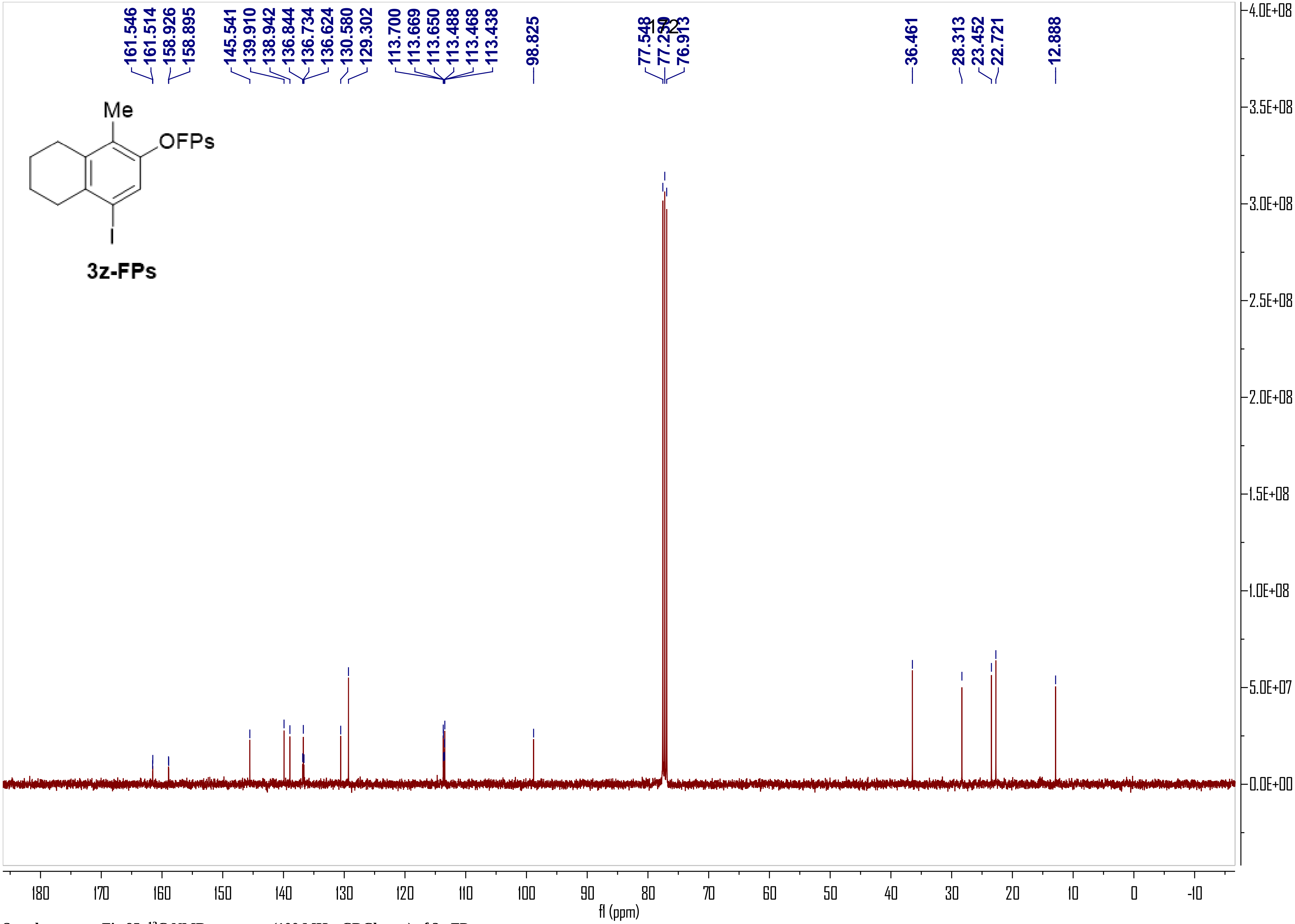

Supplementary Fig 95. ^{13}**C NMR spectrum (100 MHz, $CDCl_3$, r.t.) of 3z-FPs.**

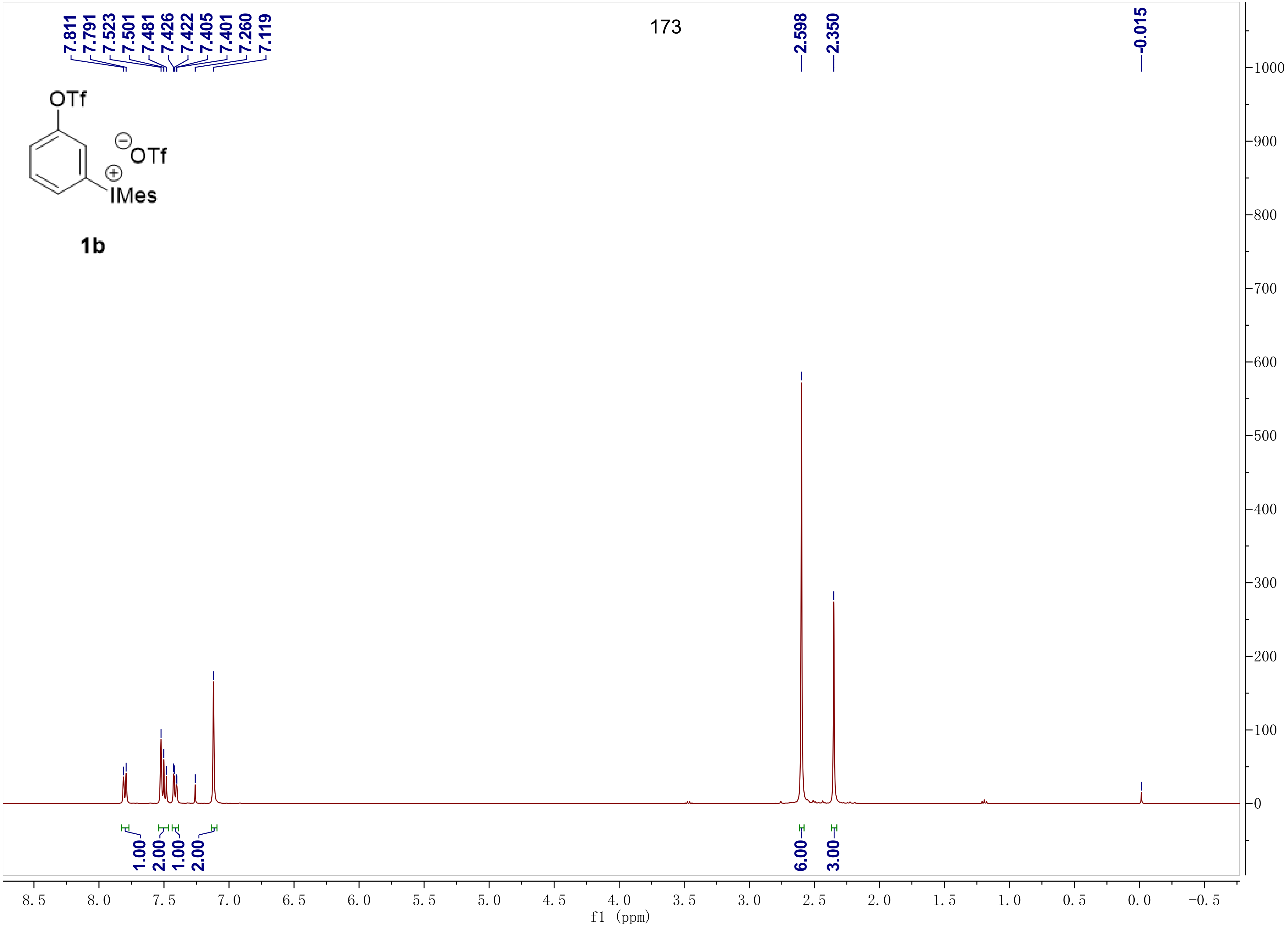

Supplementary Fig 96. **^{1}H NMR spectrum (400 MHz, $CDCl_3$, r.t.) of 1b.**

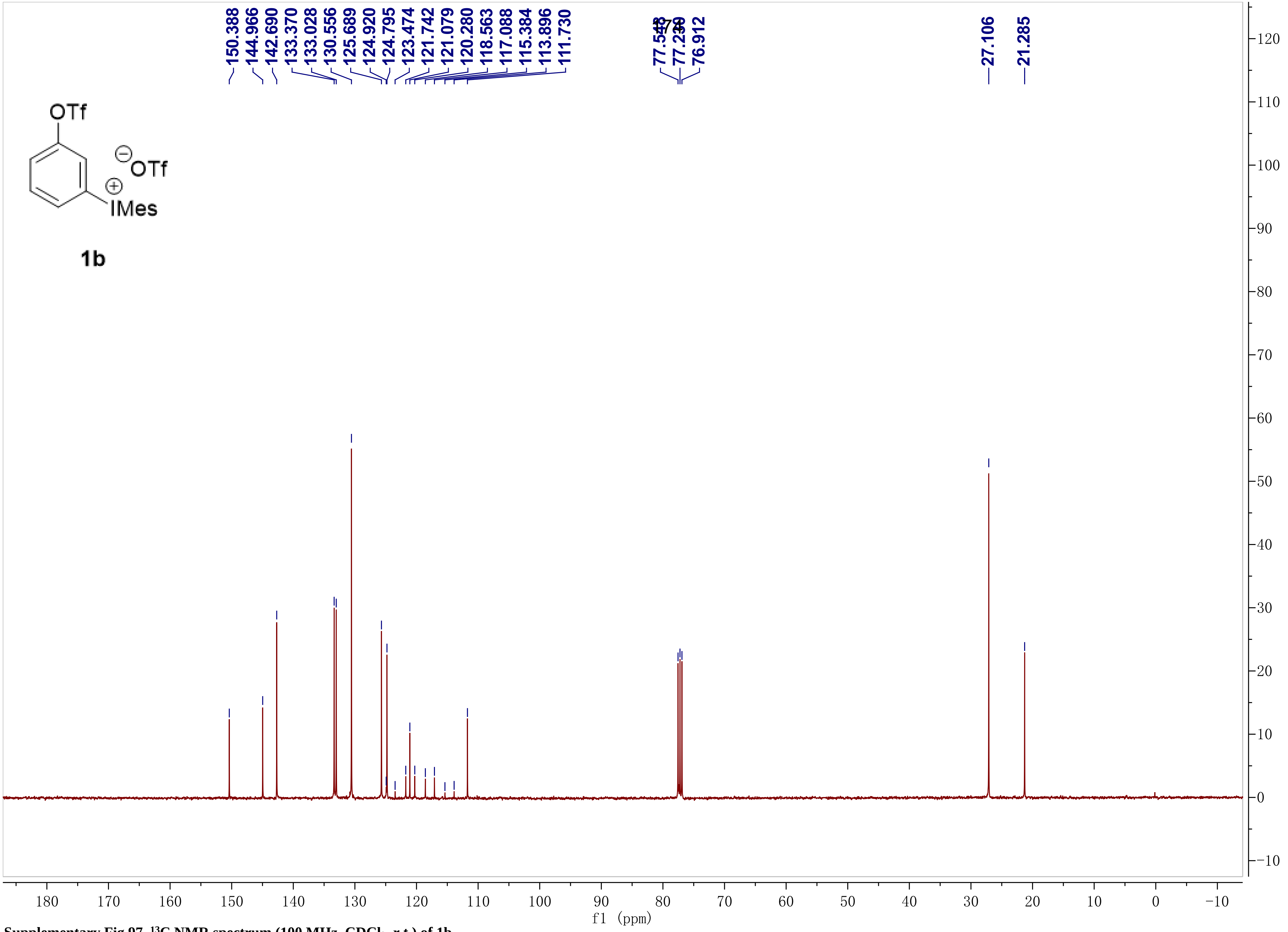

Supplementary Fig 97. **^{13}C NMR spectrum (100 MHz, $CDCl_3$, r.t.) of 1b.**

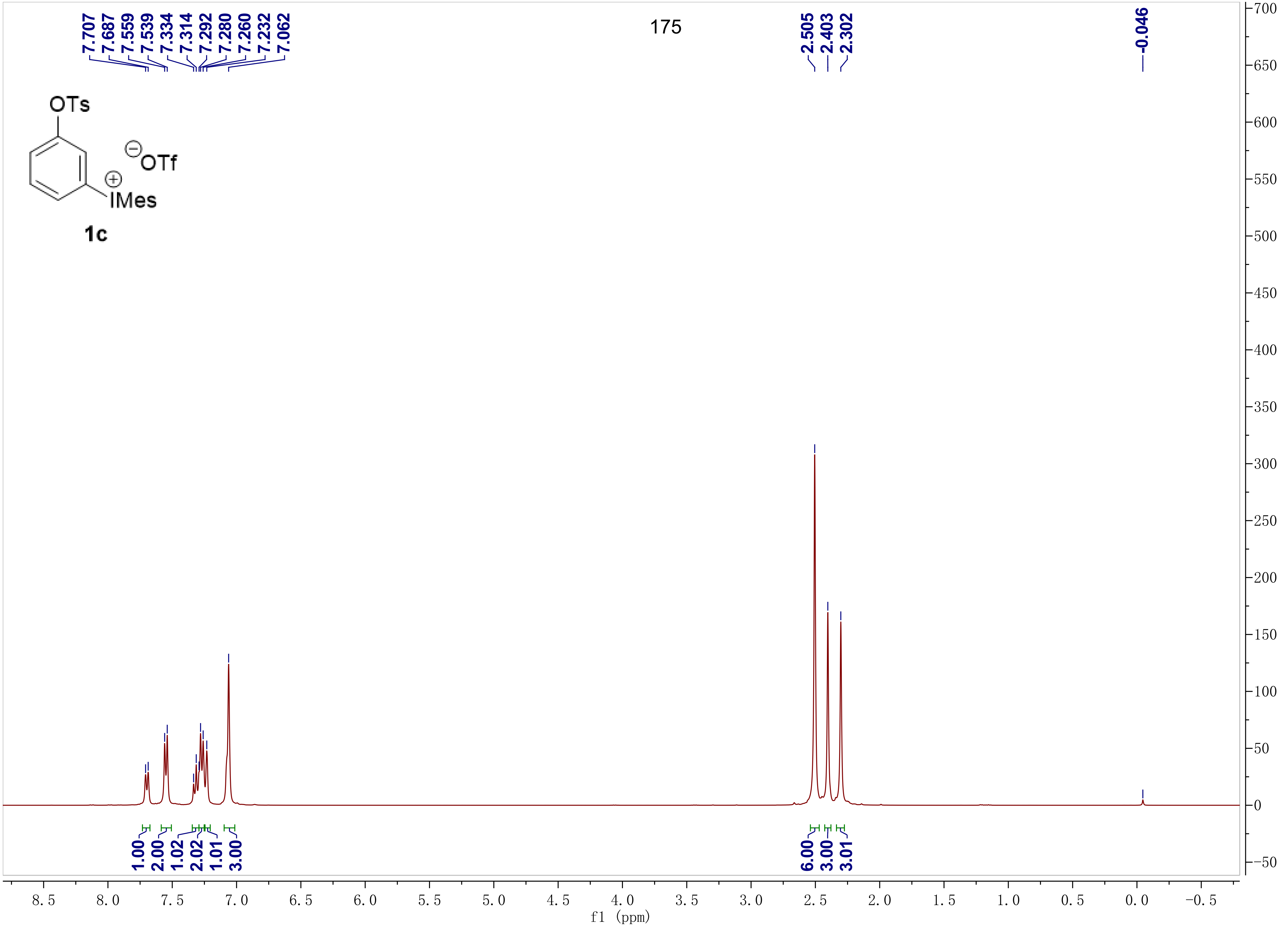

Supplementary Fig 98. ^{1}H NMR spectrum (400 MHz, $CDCl_3$, r.t.) of 1c.

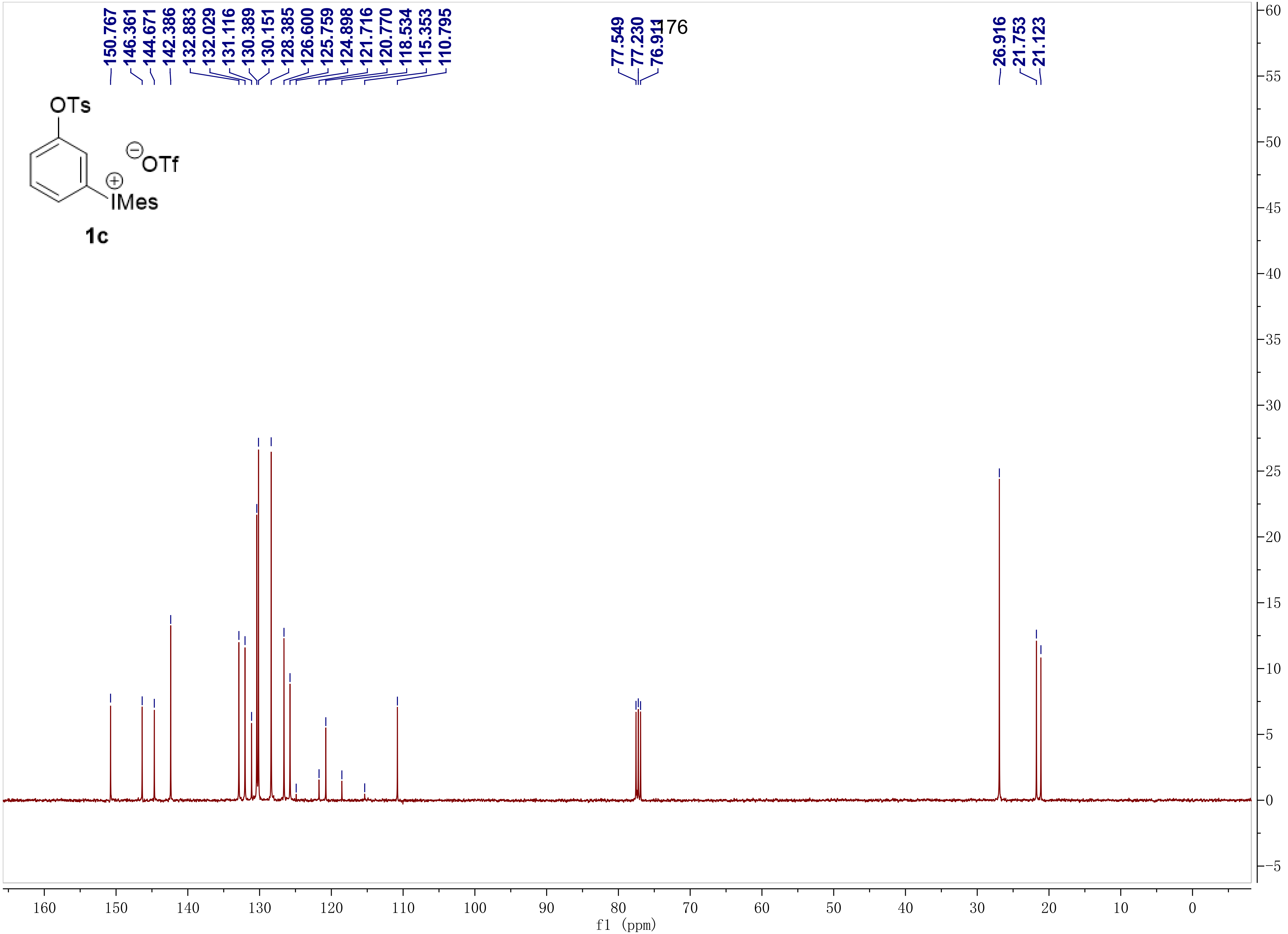

Supplementary Fig 99. **^{13}C NMR spectrum (100 MHz, $CDCl_3$, r.t.) of 1c.**

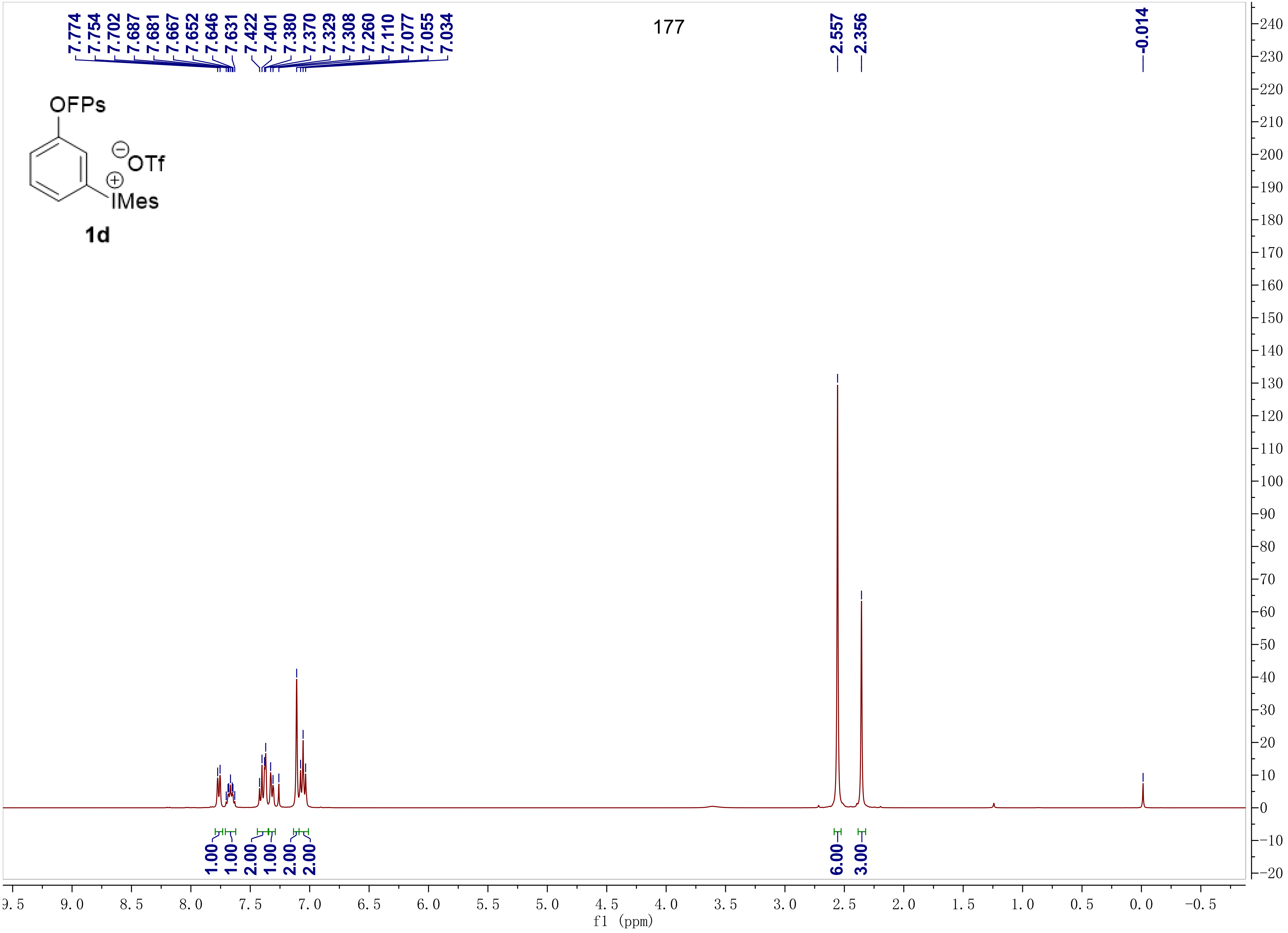

Supplementary Fig 100. ¹H NMR spectrum (400 MHz, $CDCl_3$, r.t.) of 1d.

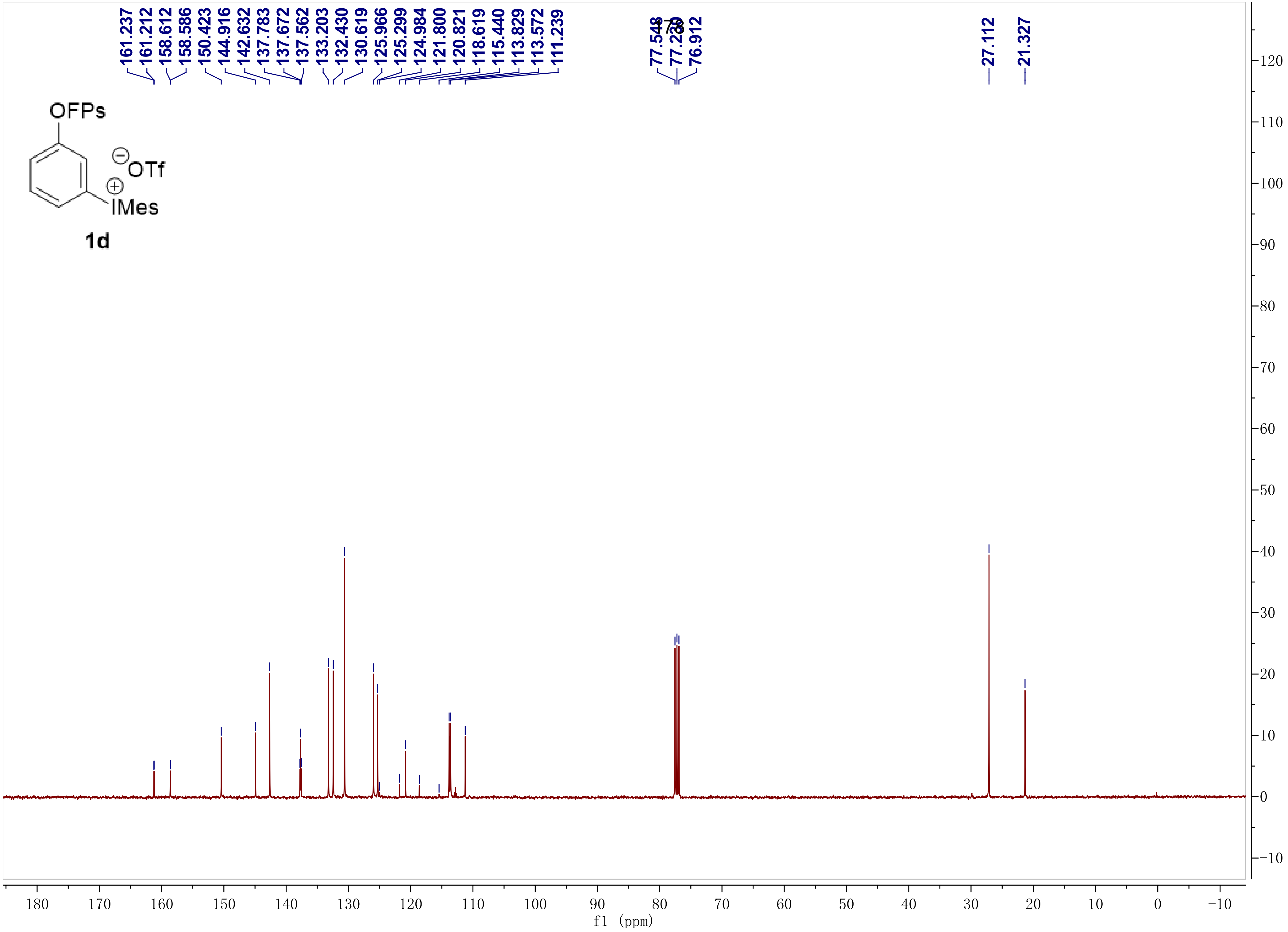

Supplementary Fig 101. **^{13}C NMR spectrum (100 MHz, $CDCl_3$, r.t.) of 1d.**

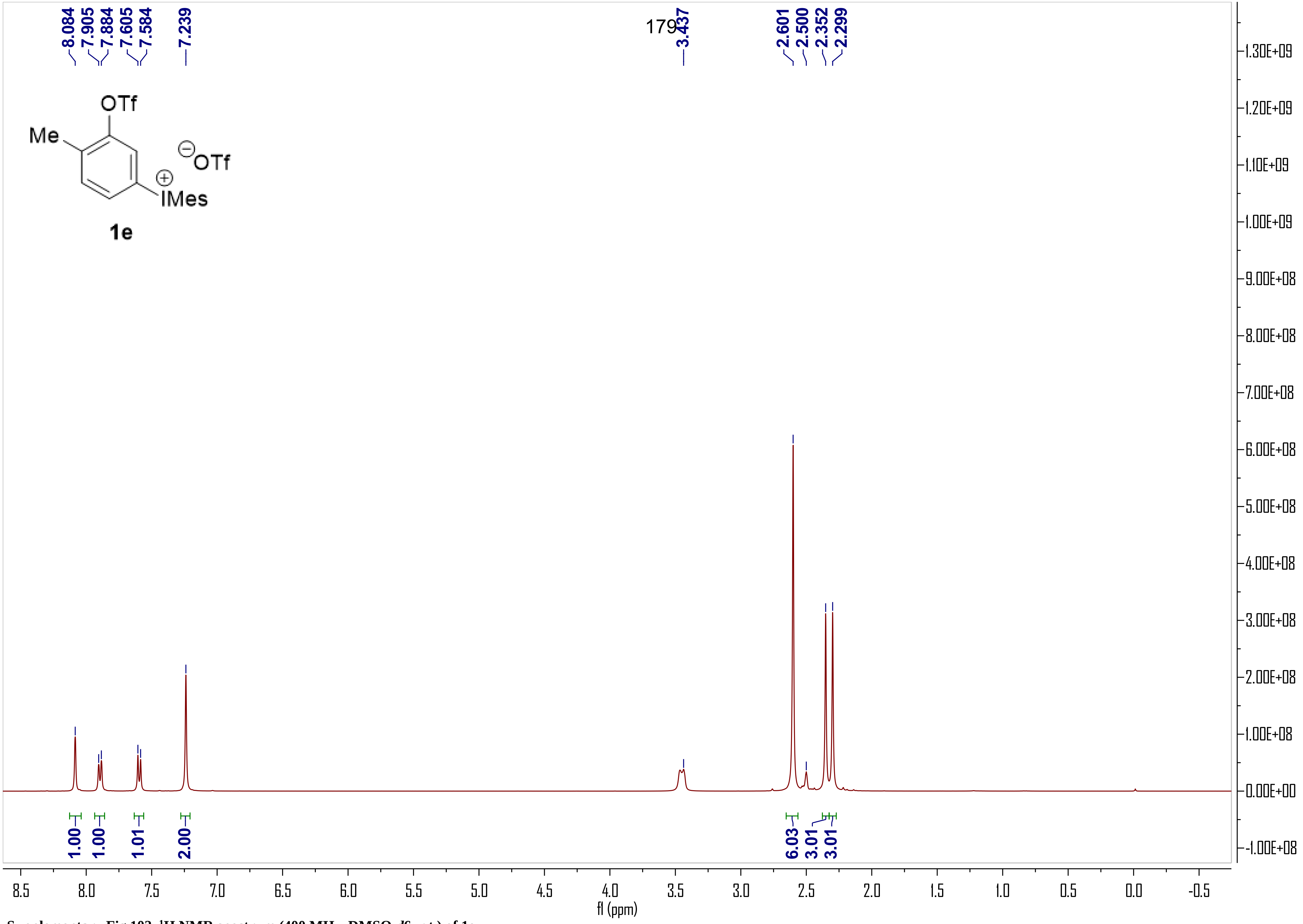

Supplementary Fig 102. ^{1}H NMR spectrum (400 MHz, DMSO-*d*6, r.t.) of 1e.

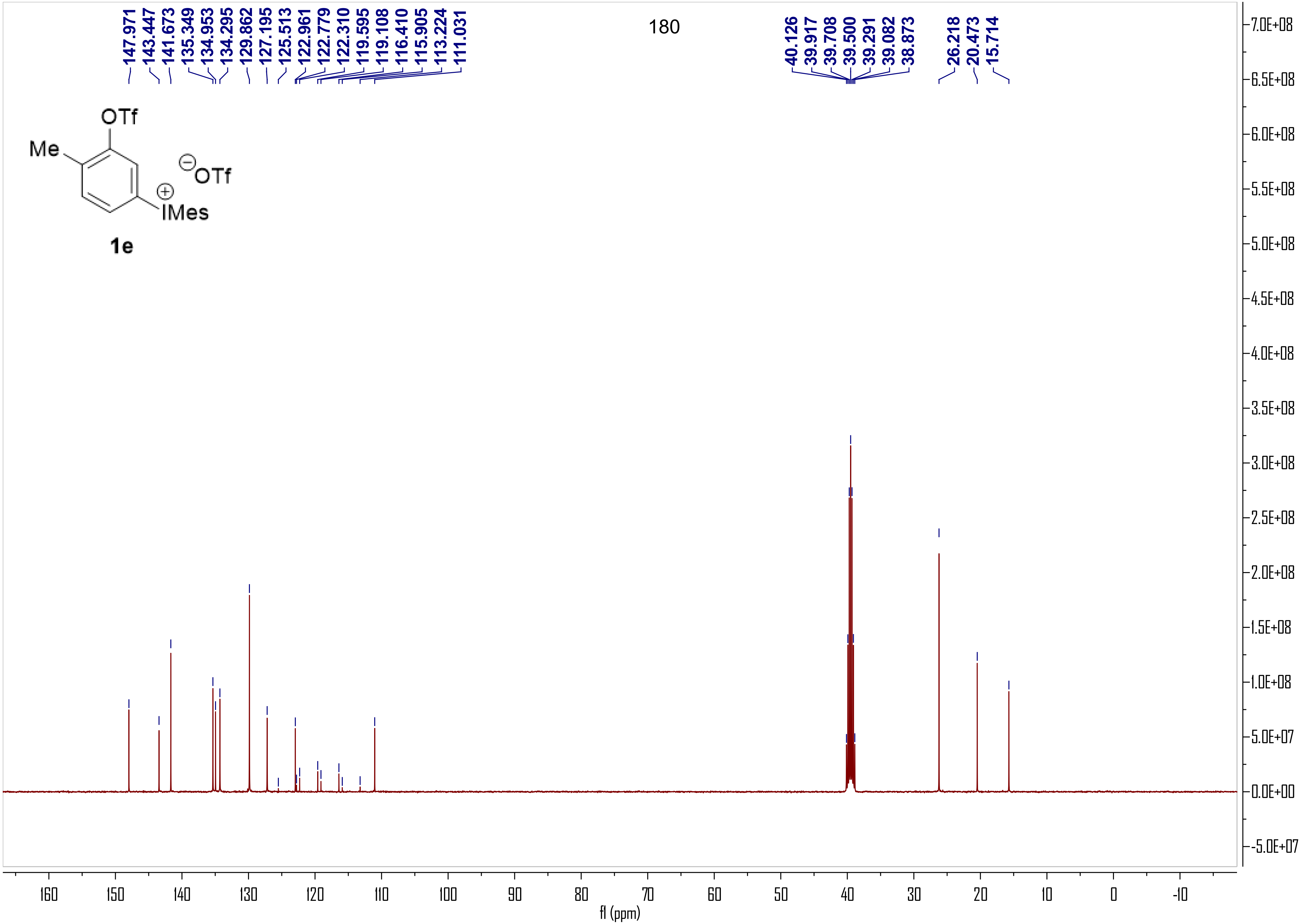

Supplementary Fig 103. ^{13}**C NMR spectrum (100 MHz, DMSO-*d*6, r.t.) of 1e.**

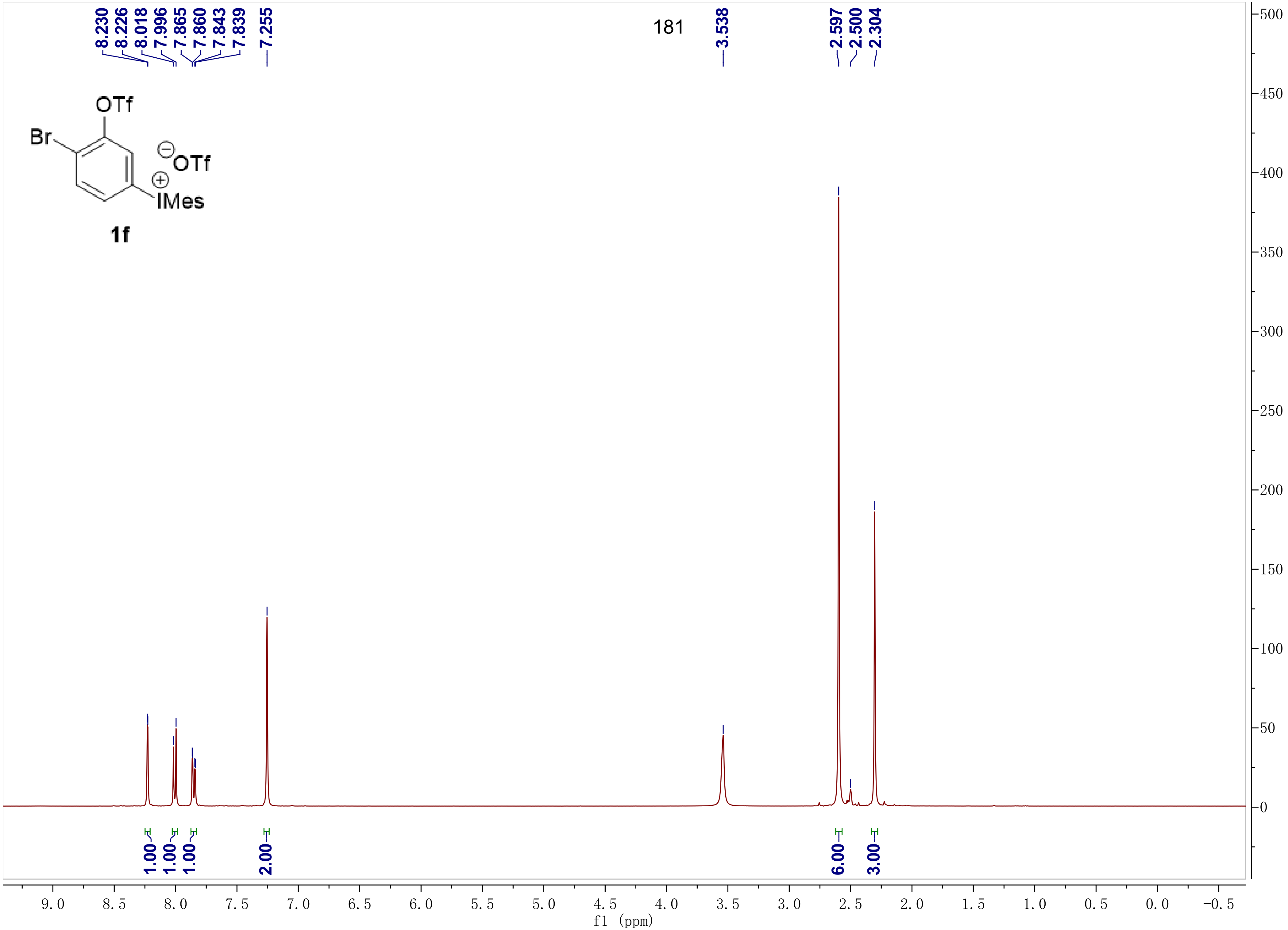

Supplementary Fig 104. **^{1}H NMR spectrum (400 MHz, DMSO-*d6*, r.t.) of 1f.**

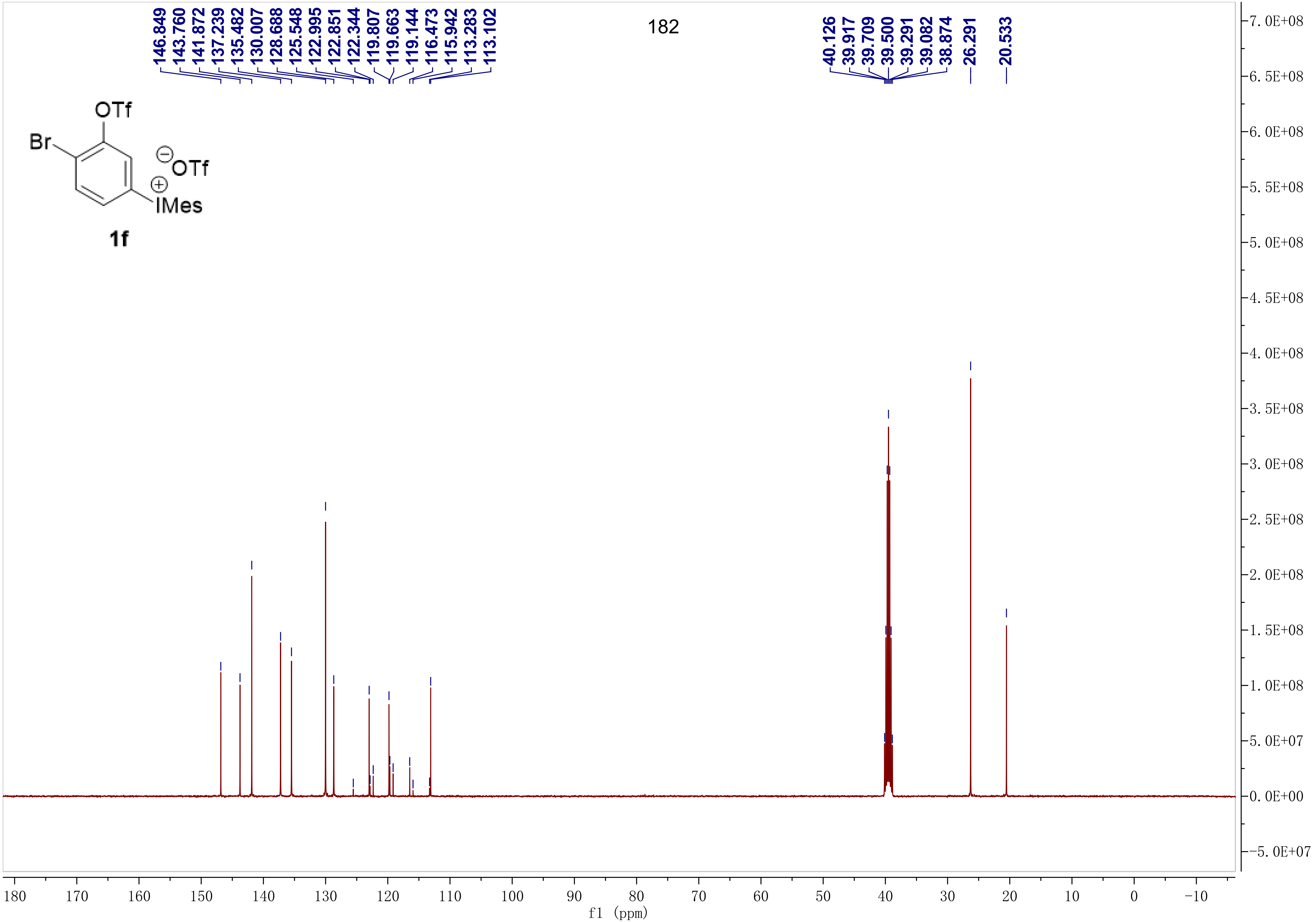

Supplementary Fig 105. ^{13}C **NMR spectrum (100 MHz, DMSO-*d*6, r.t.) of 1f.**

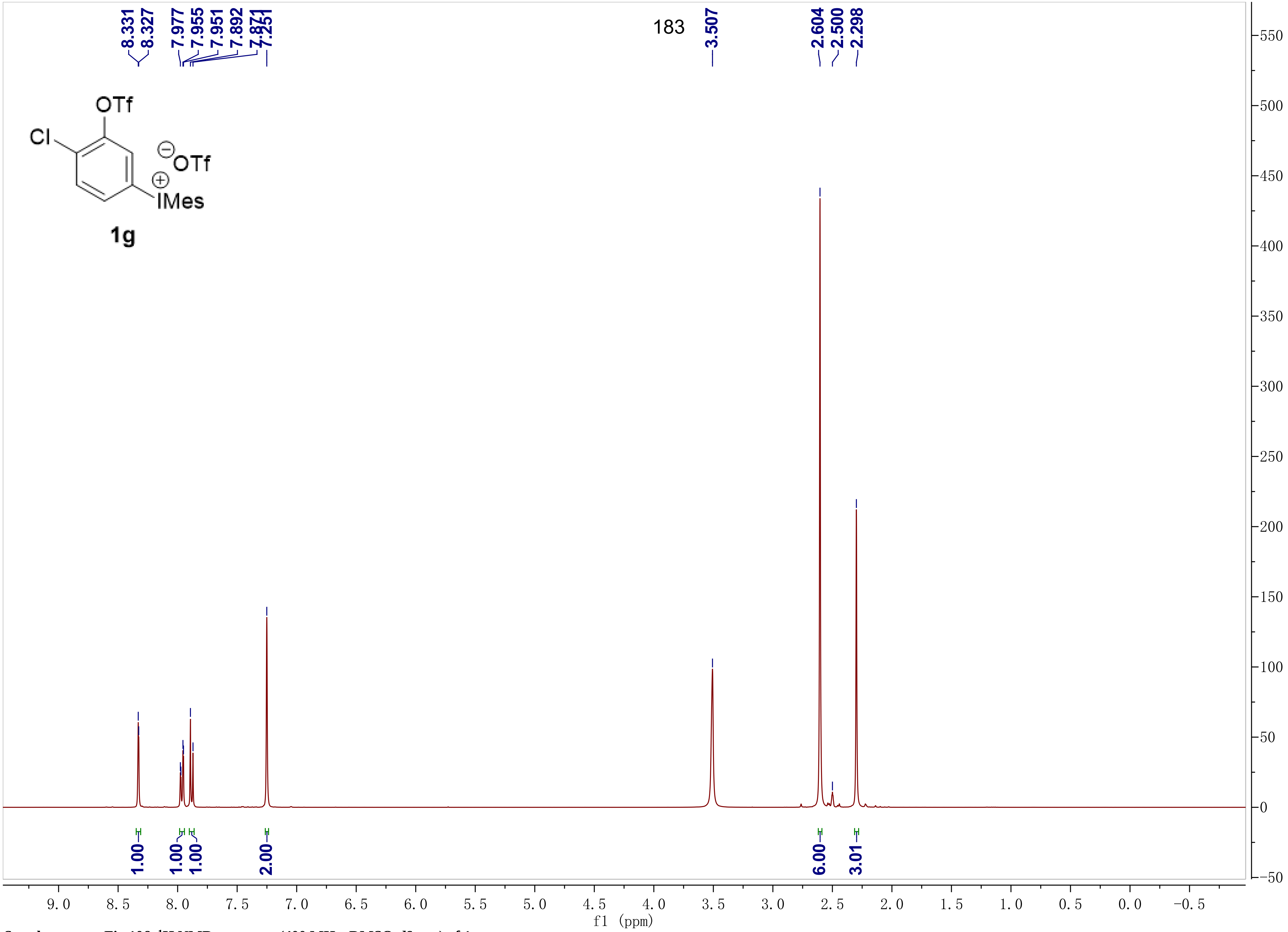

Supplementary Fig 106. ^{1}H NMR spectrum (400 MHz, DMSO-*d*6, r.t.) of 1g.

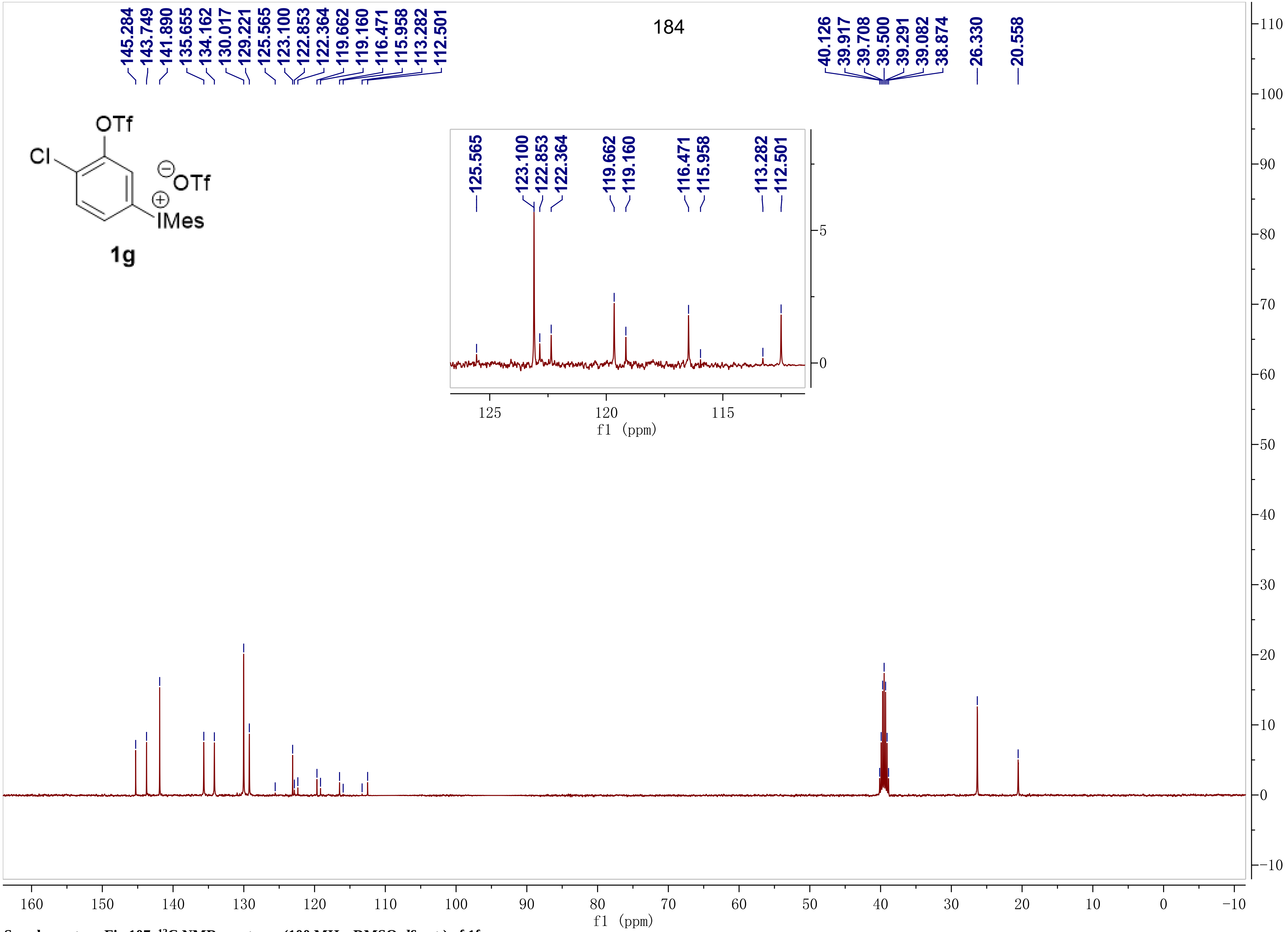

Supplementary Fig 107. **^{13}C NMR spectrum (100 MHz, DMSO-*d6*, r.t.) of 1f.**

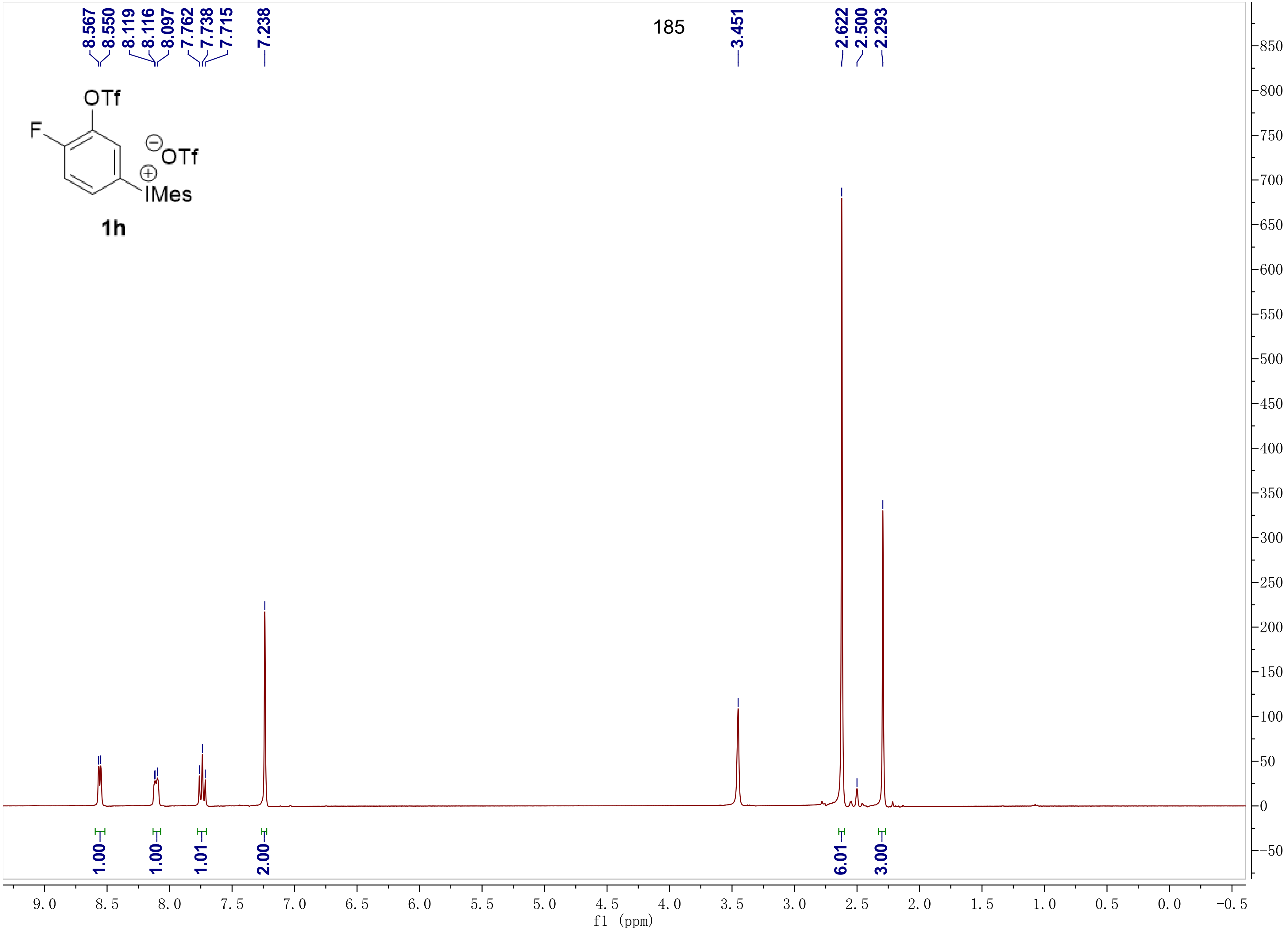

Supplementary Fig 108. ^{1}H **NMR spectrum (400 MHz, DMSO-*d*6, r.t.) of 1h.**

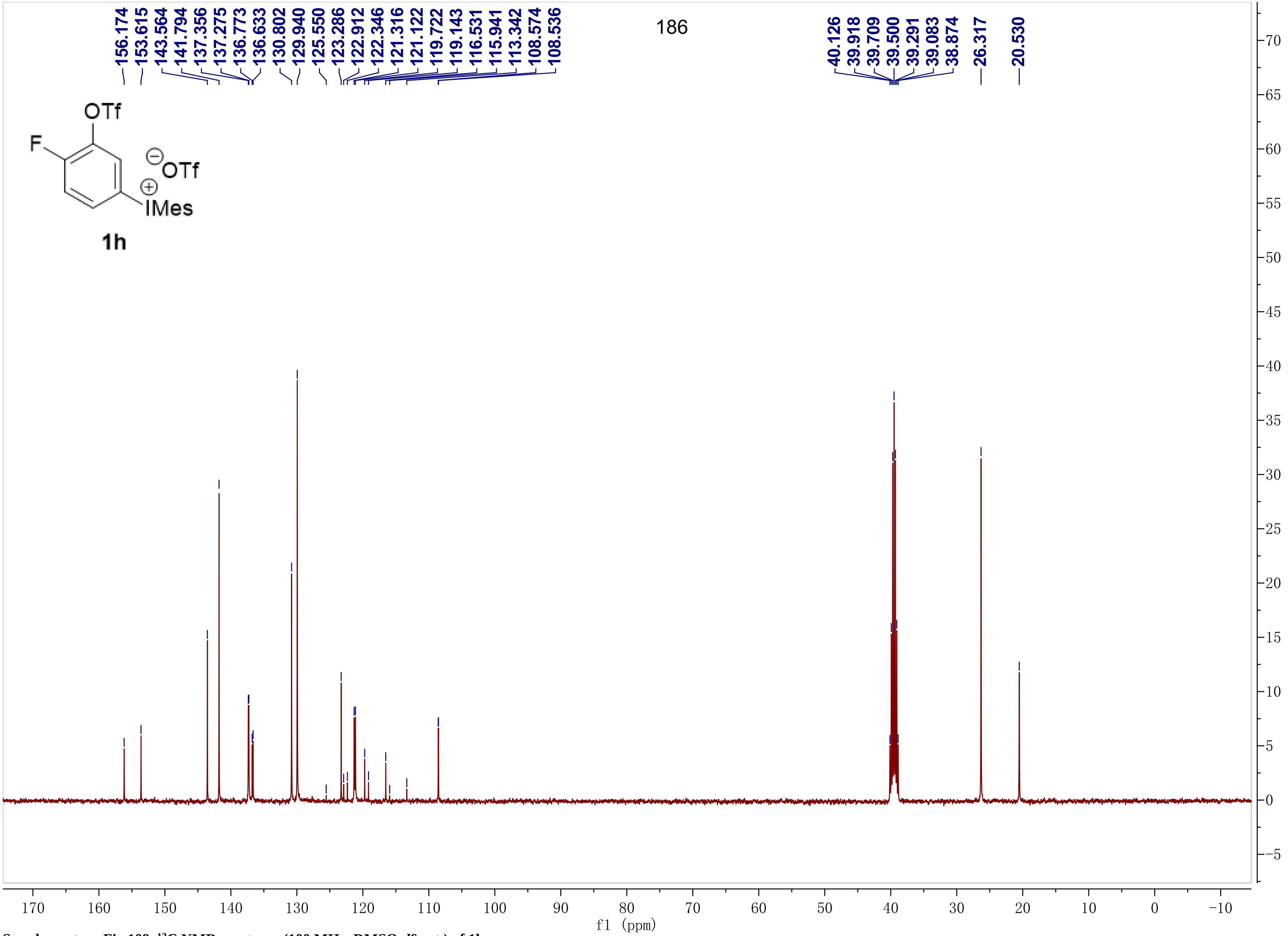

Supplementary Fig 109. ^{13}C NMR spectrum (100 MHz, DMSO-*d*6, r.t.) of 1h.

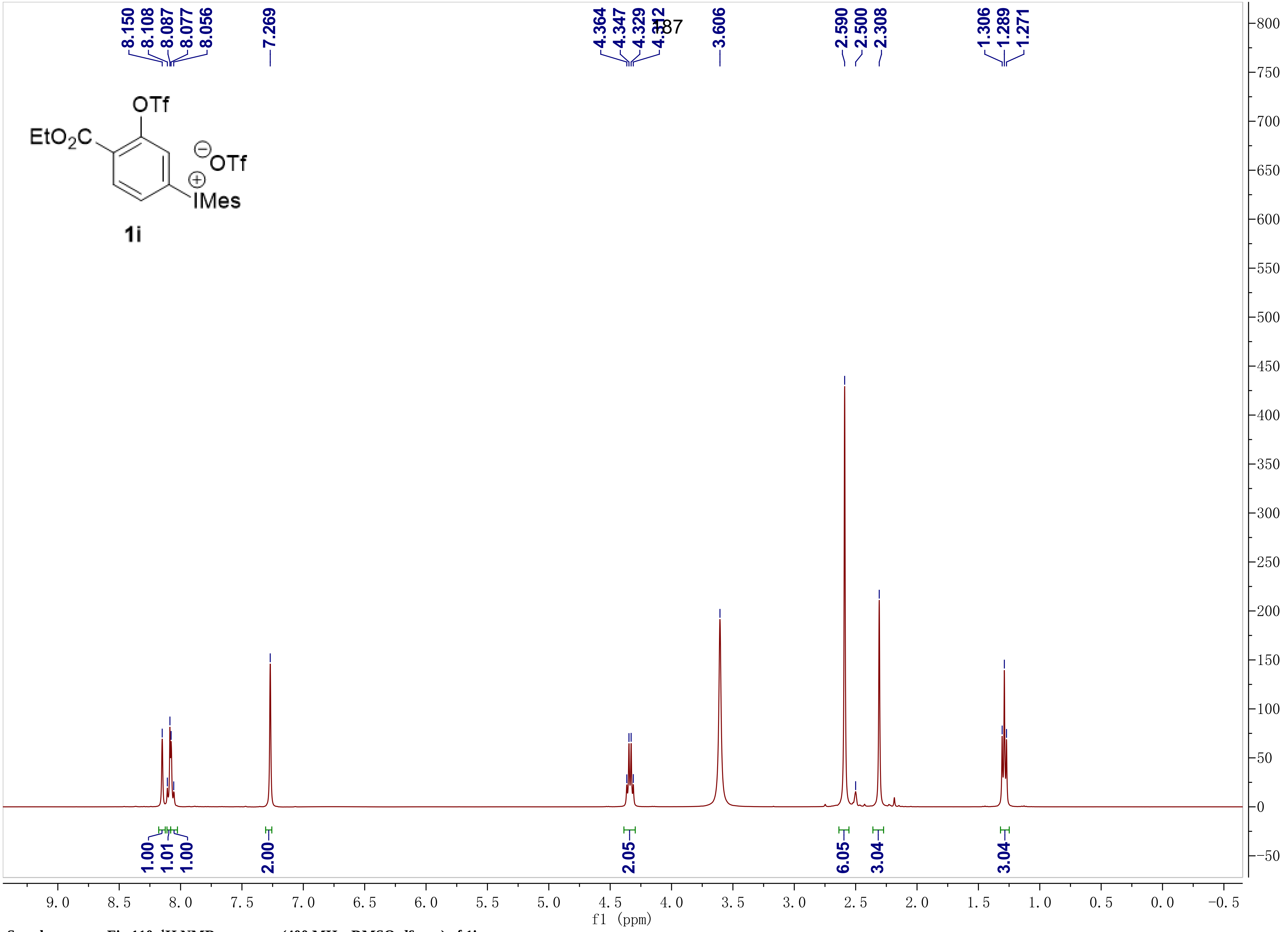

Supplementary Fig 110. ¹H NMR spectrum (400 MHz, DMSO-*d*6, r.t.) of 1i.

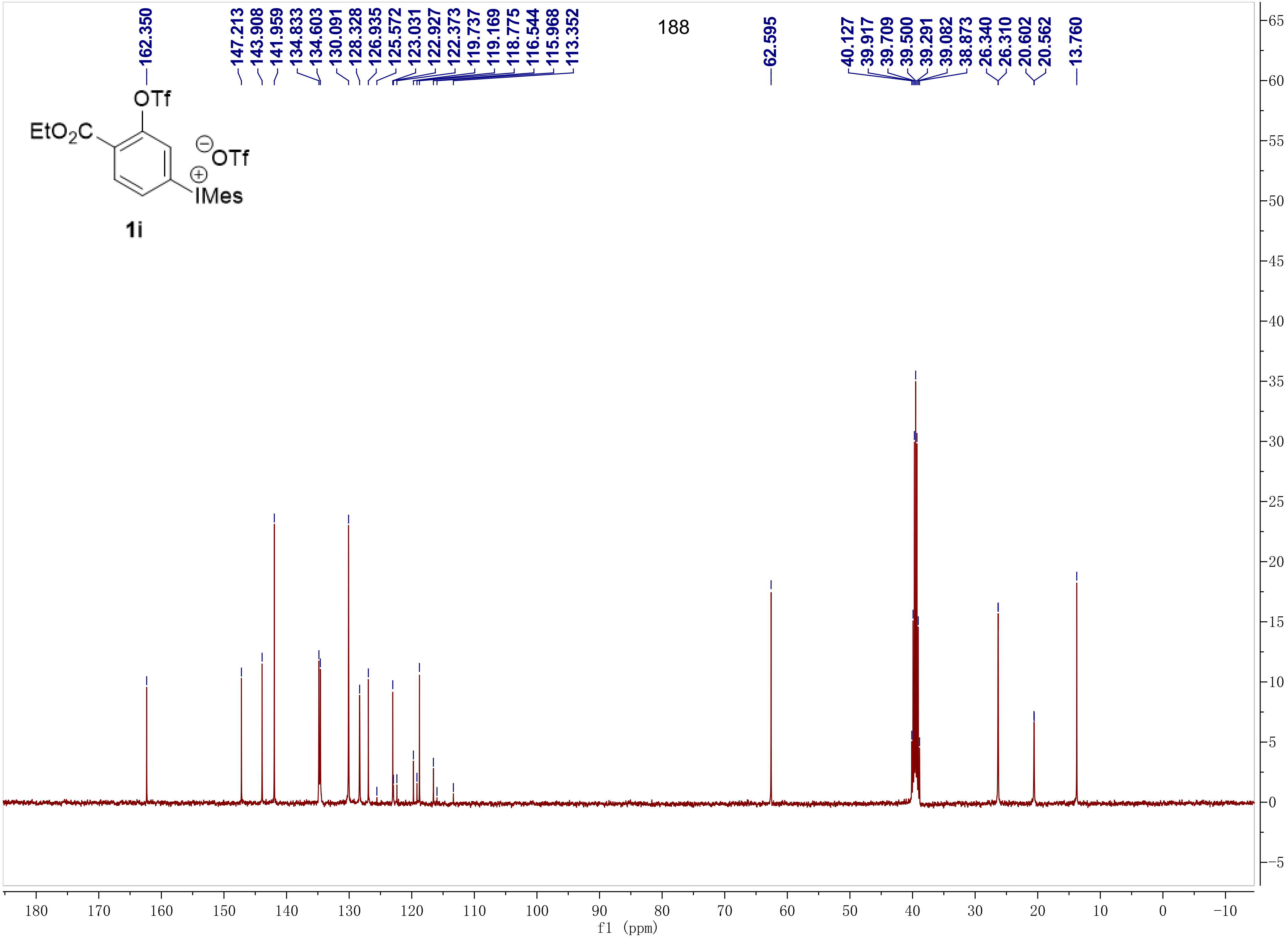

Supplementary Fig 111. **^{13}C NMR spectrum (100 MHz, DMSO-*d*6, r.t.) of 1i.**

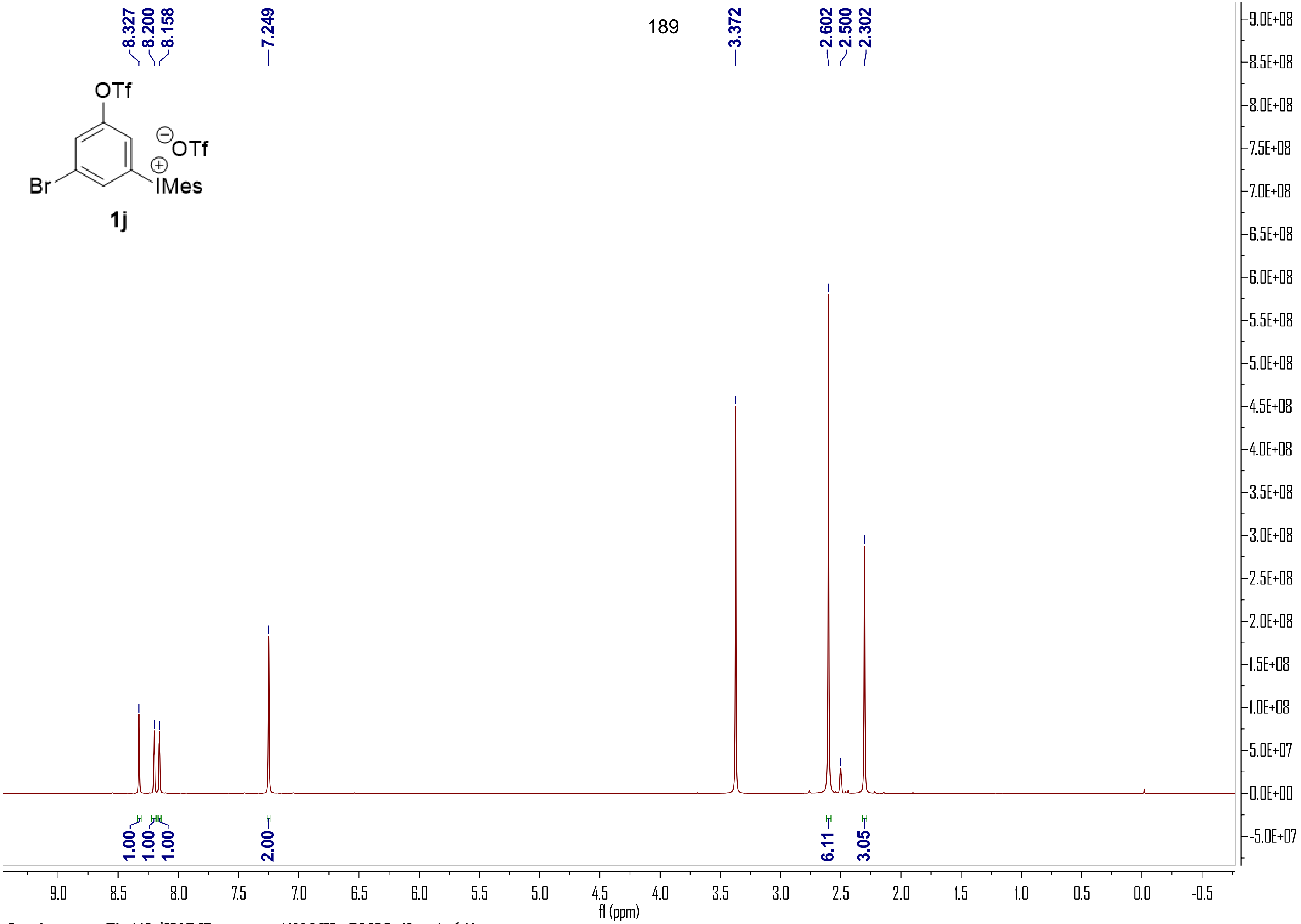

1j

Supplementary Fig 112. ^{1}H NMR spectrum (400 MHz, DMSO-*d*6, r.t.) of 1j.

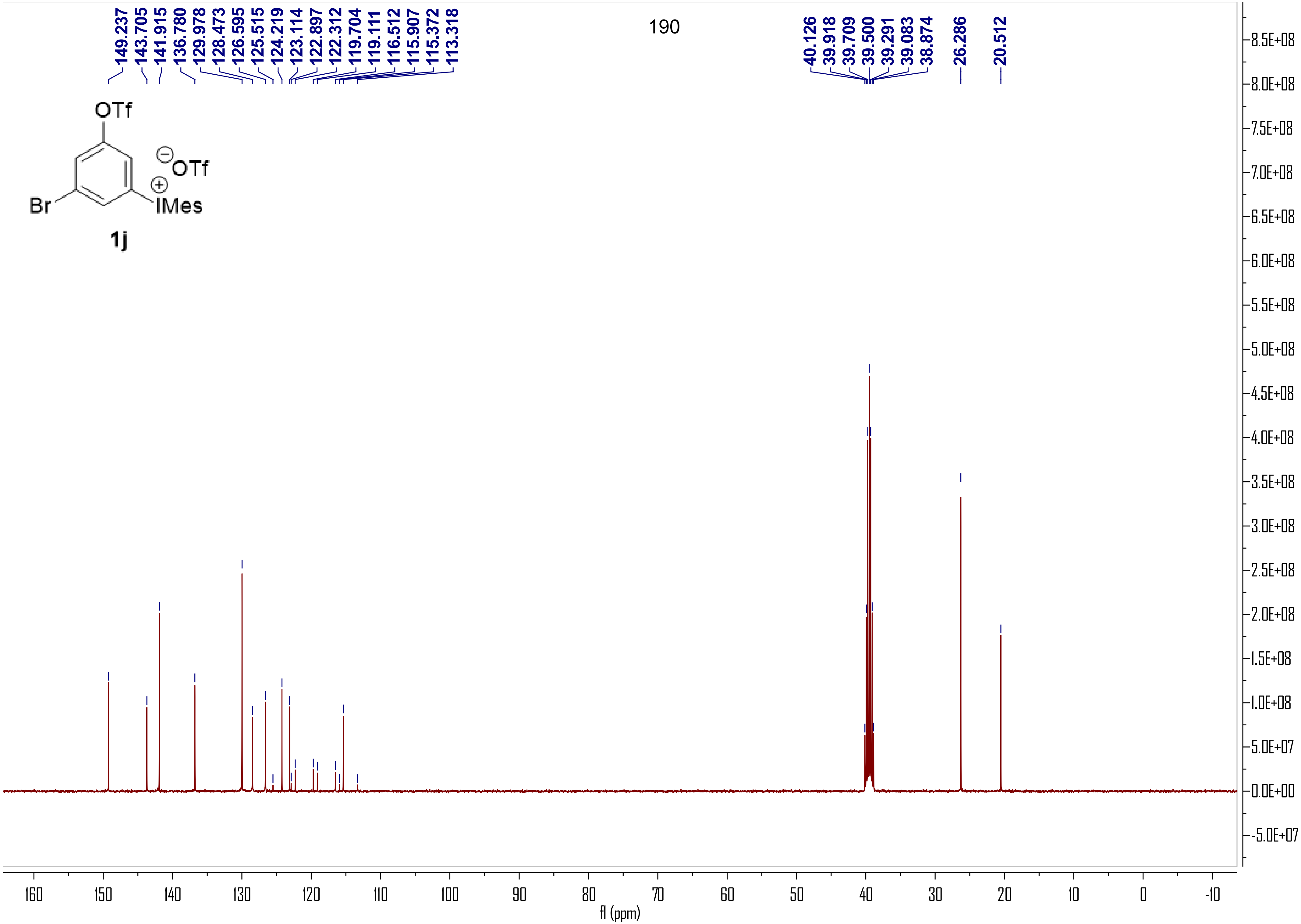

Supplementary Fig 113. **^{13}C NMR spectrum (100 MHz, DMSO-*d*6, r.t.) of 1j.**

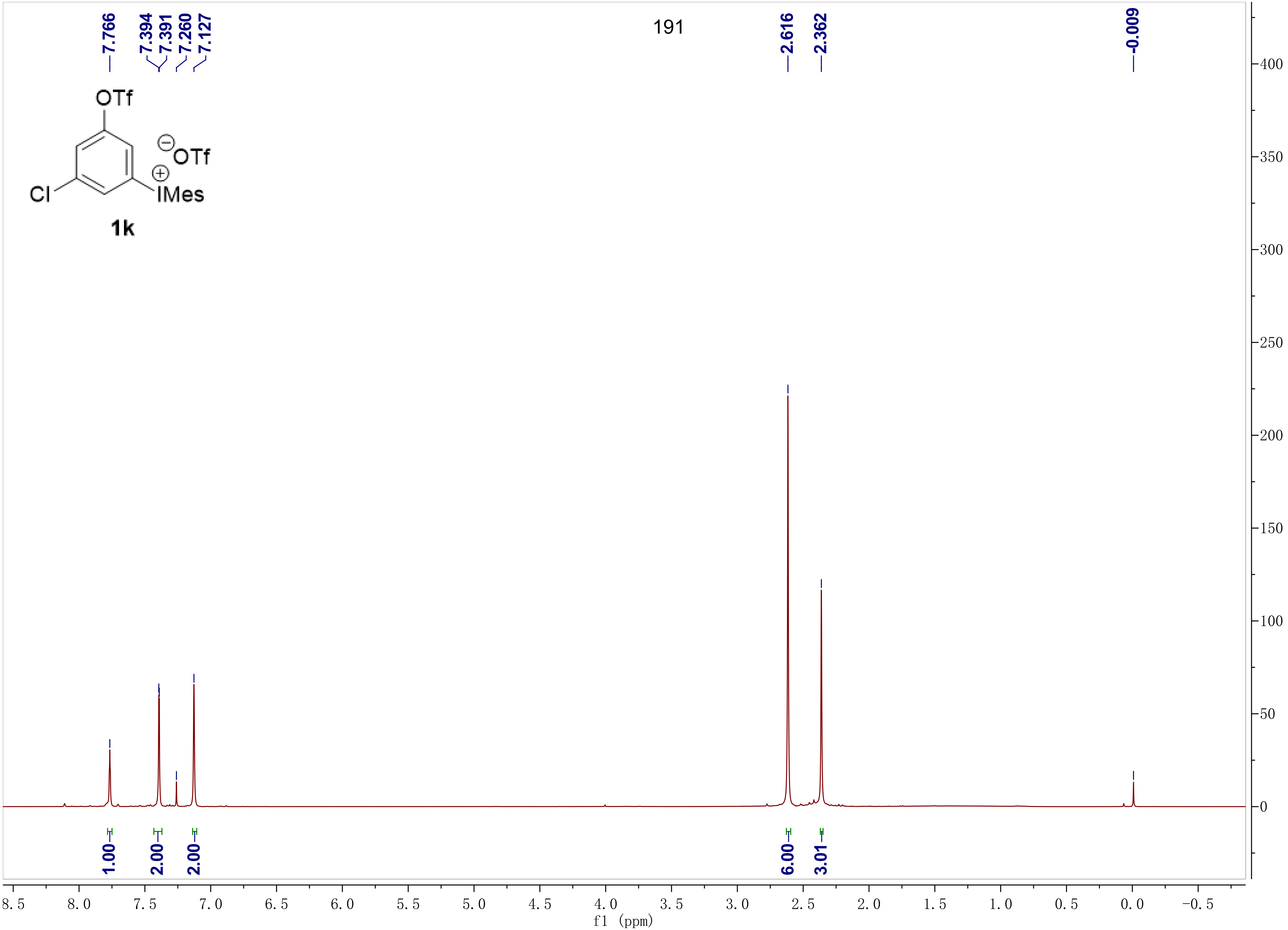

Supplementary Fig 114. ^{1}H NMR spectrum (400 MHz, DMSO-*d6*, r.t.) of 1k.

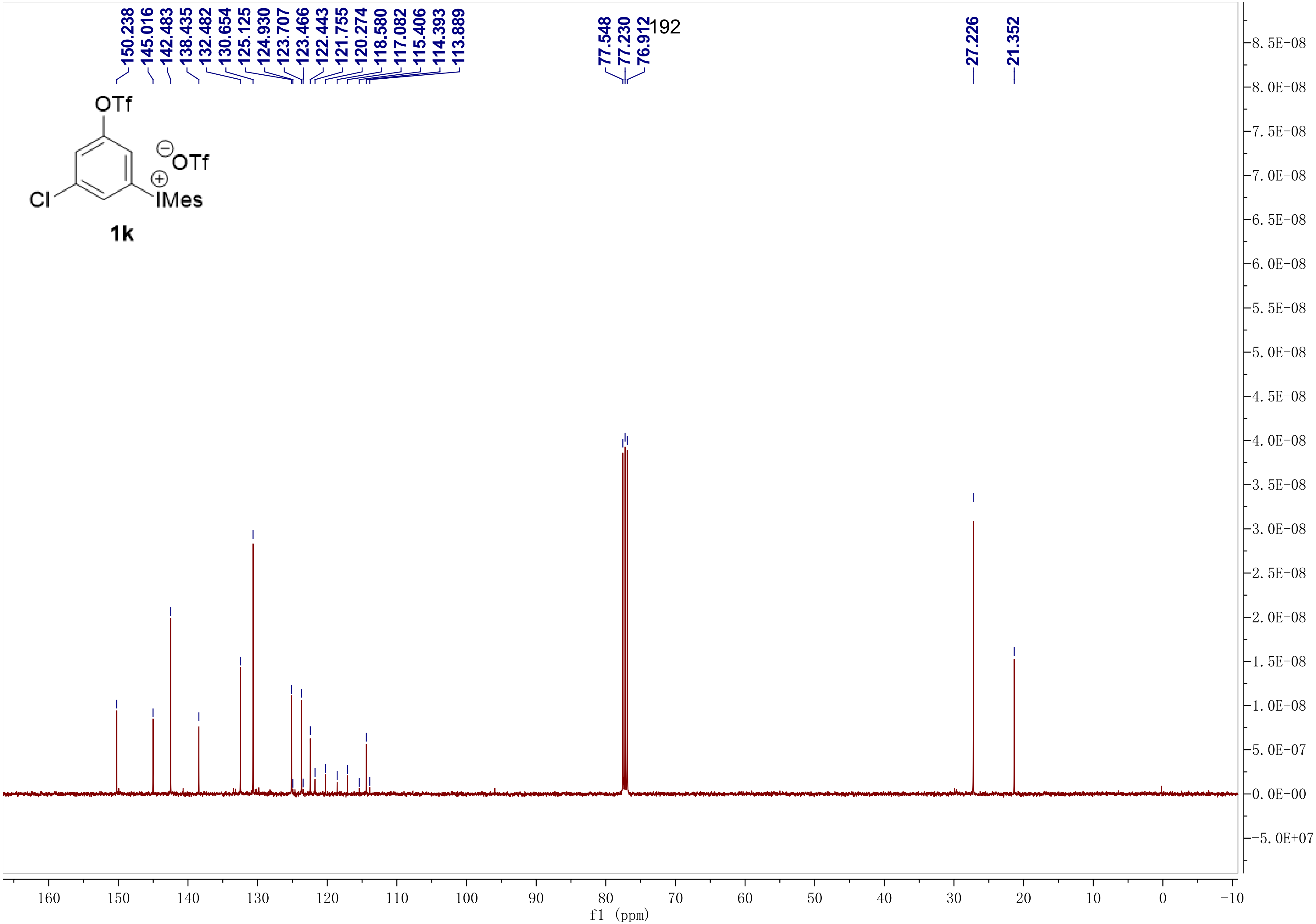

Supplementary Fig 115. ^{13}**C NMR spectrum (100 MHz, DMSO-*d*6, r.t.) of 1k.**

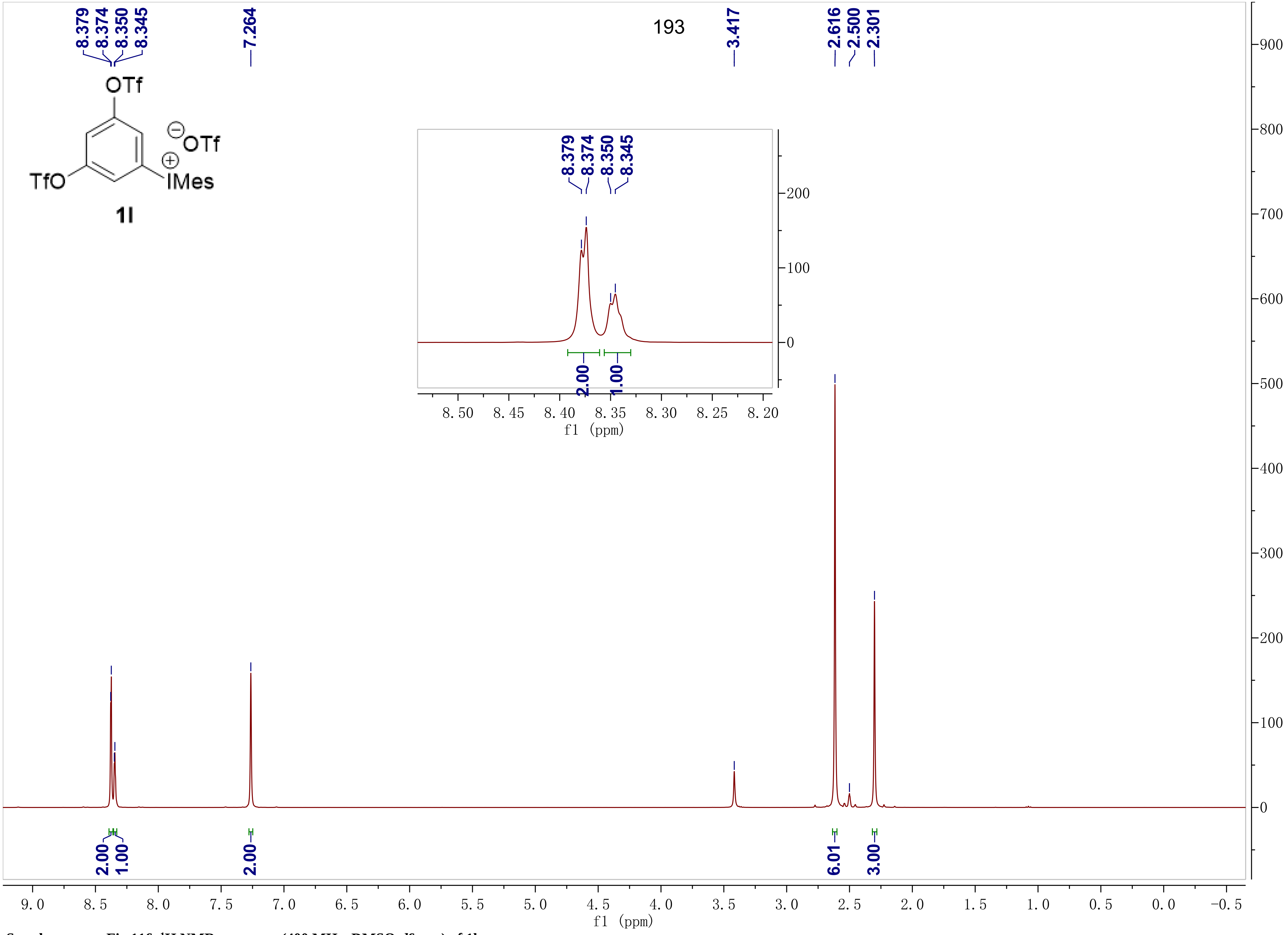

Supplementary Fig 116. **^{1}H NMR spectrum (400 MHz, DMSO-*d*6, r.t.) of 1l.**

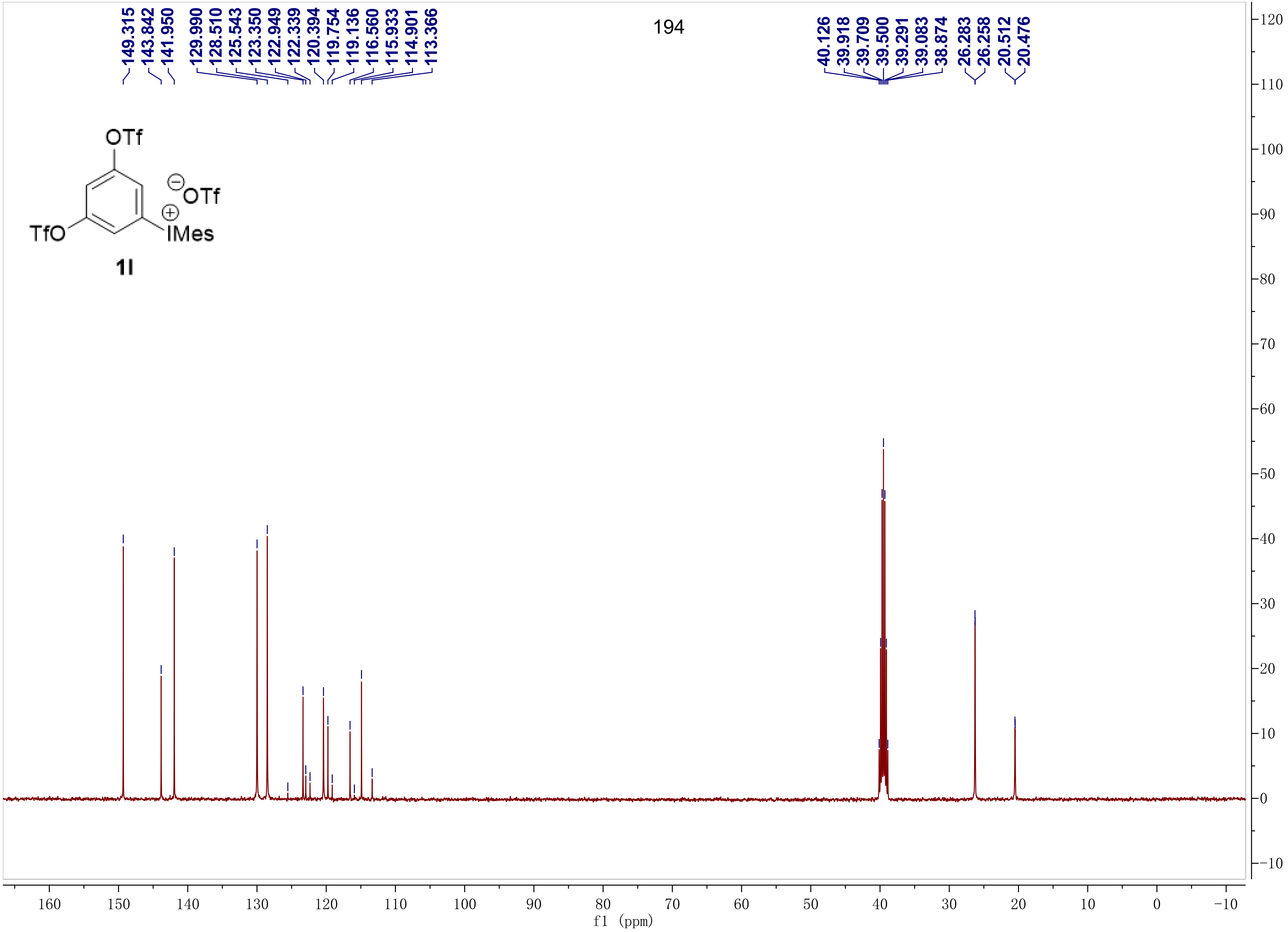

Supplementary Fig 117. ^{13}C NMR spectrum (100 MHz, DMSO-*d*6, r.t.) of 1l.

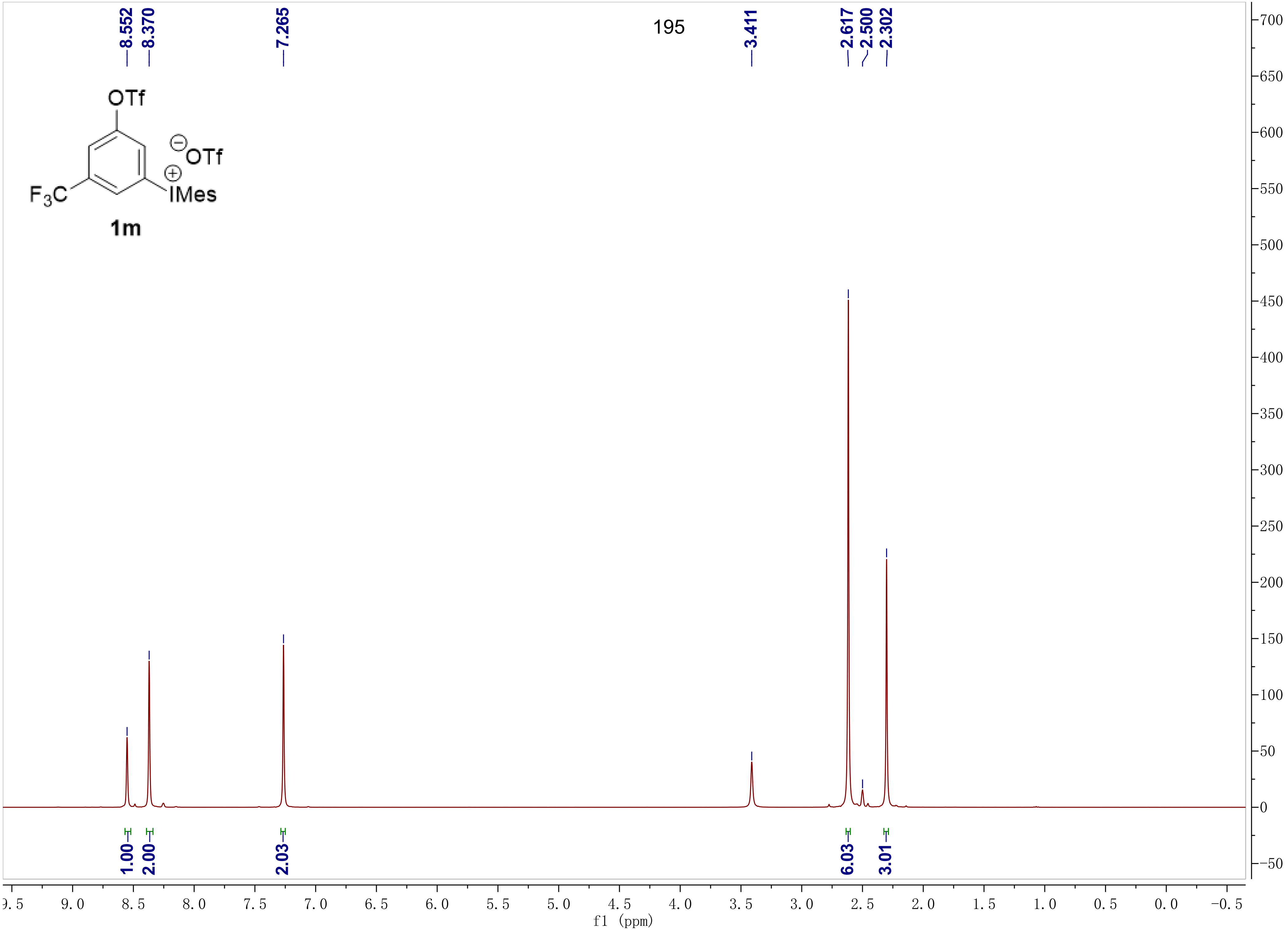

Supplementary Fig 118. ^{1}H **NMR spectrum (400 MHz, DMSO-*d*6, r.t.) of 1m.**

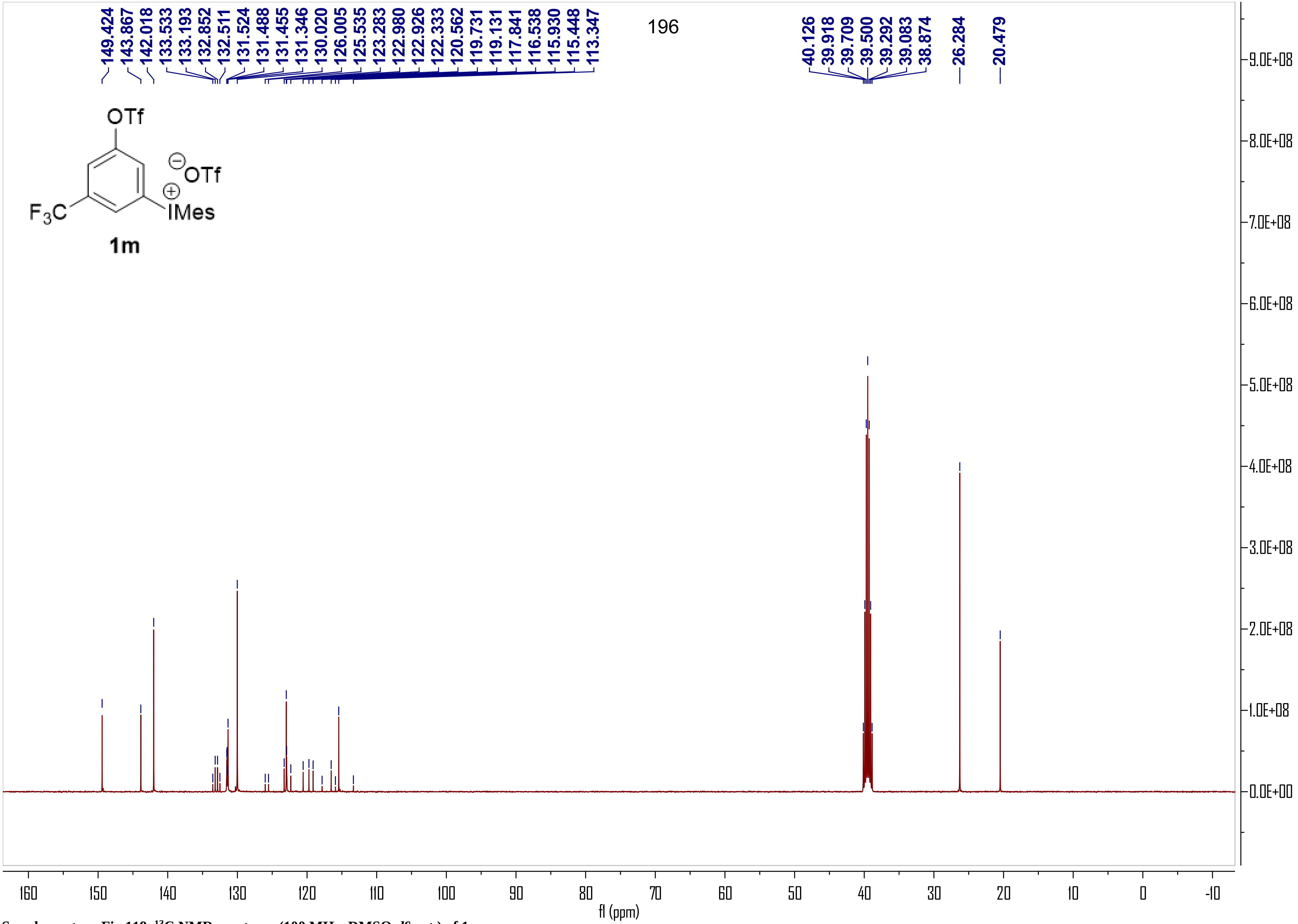

Supplementary Fig 119. ^{13}**C NMR spectrum (100 MHz, DMSO-*d*6, r.t.) of 1m.**

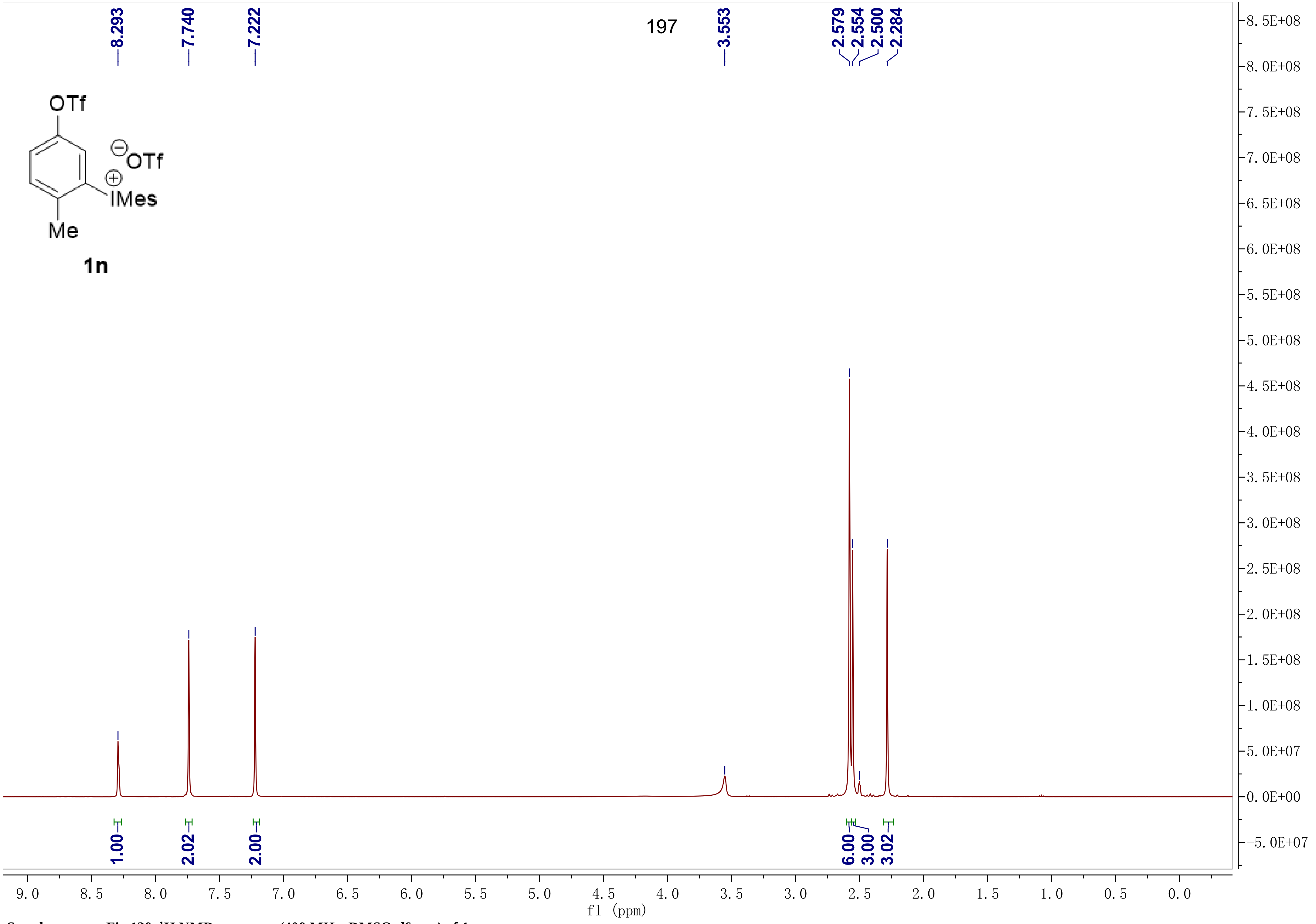

Supplementary Fig 120. **^{1}H NMR spectrum (400 MHz, DMSO-*d*6, r.t.) of 1n.**

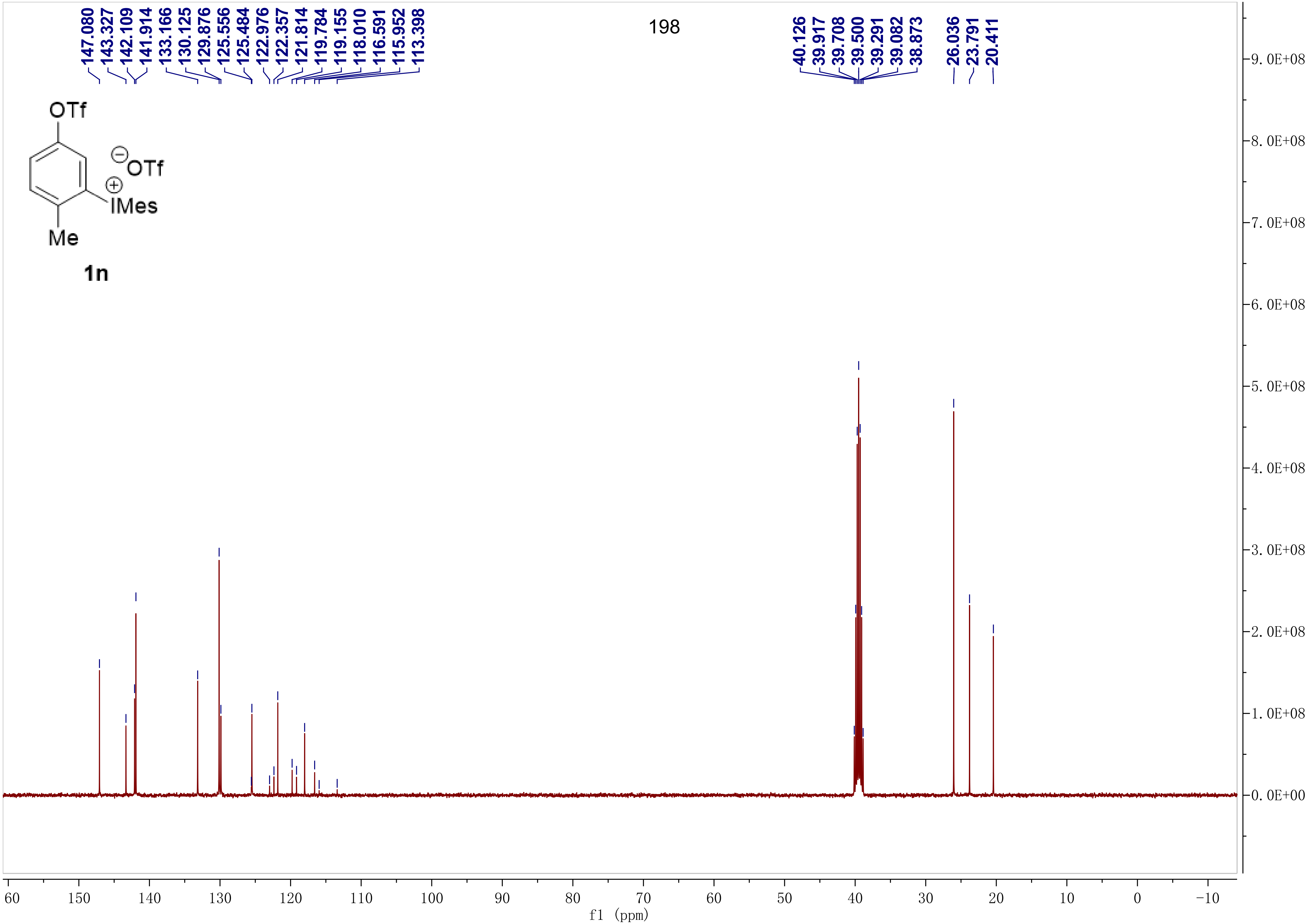

Supplementary Fig 121. **^{13}C NMR spectrum (100 MHz, DMSO-*d*6, r.t.) of 1n.**

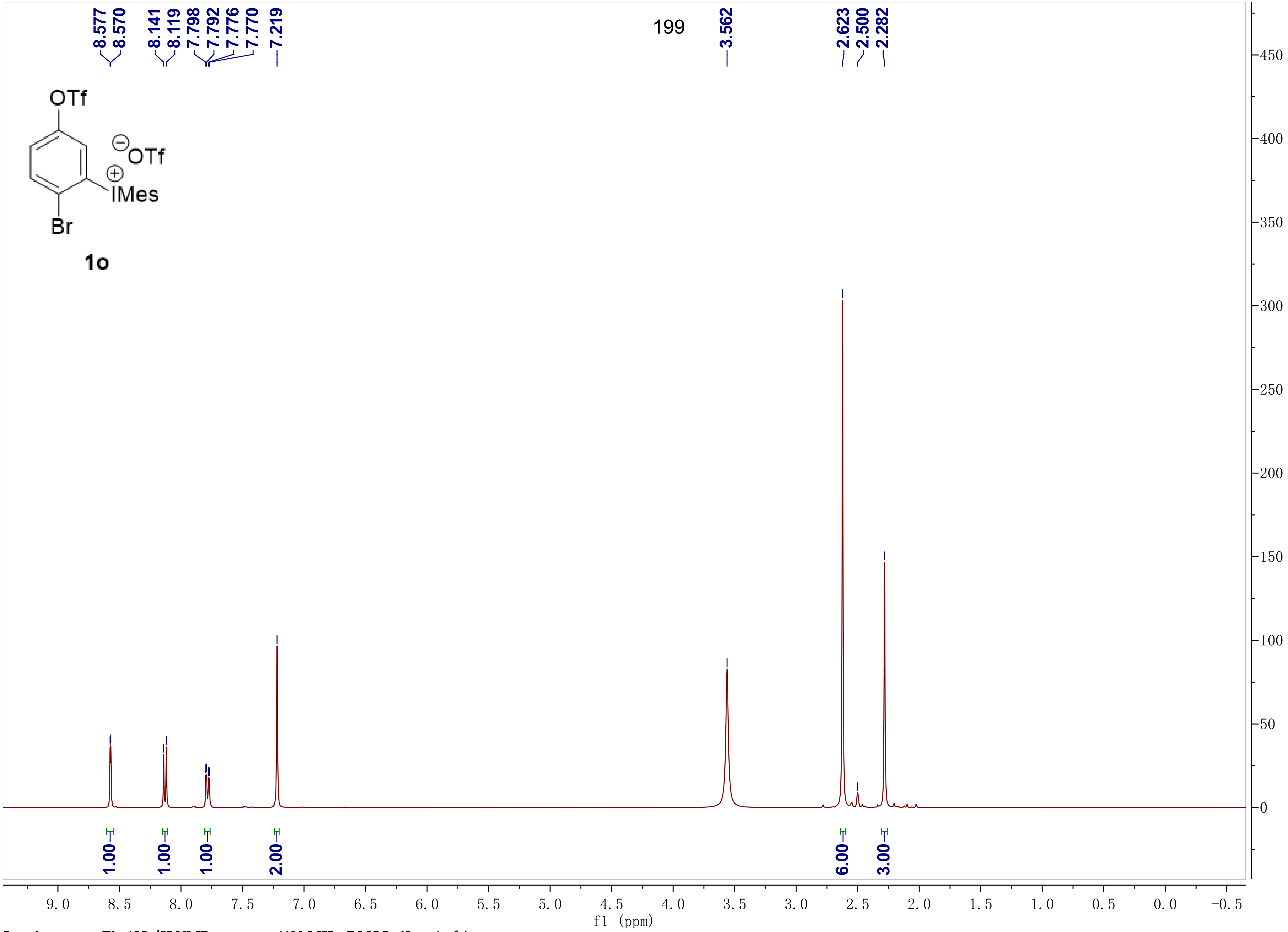

Supplementary Fig 122. ^{1}H NMR spectrum (400 MHz, DMSO-*d6*, r.t.) of 1o.

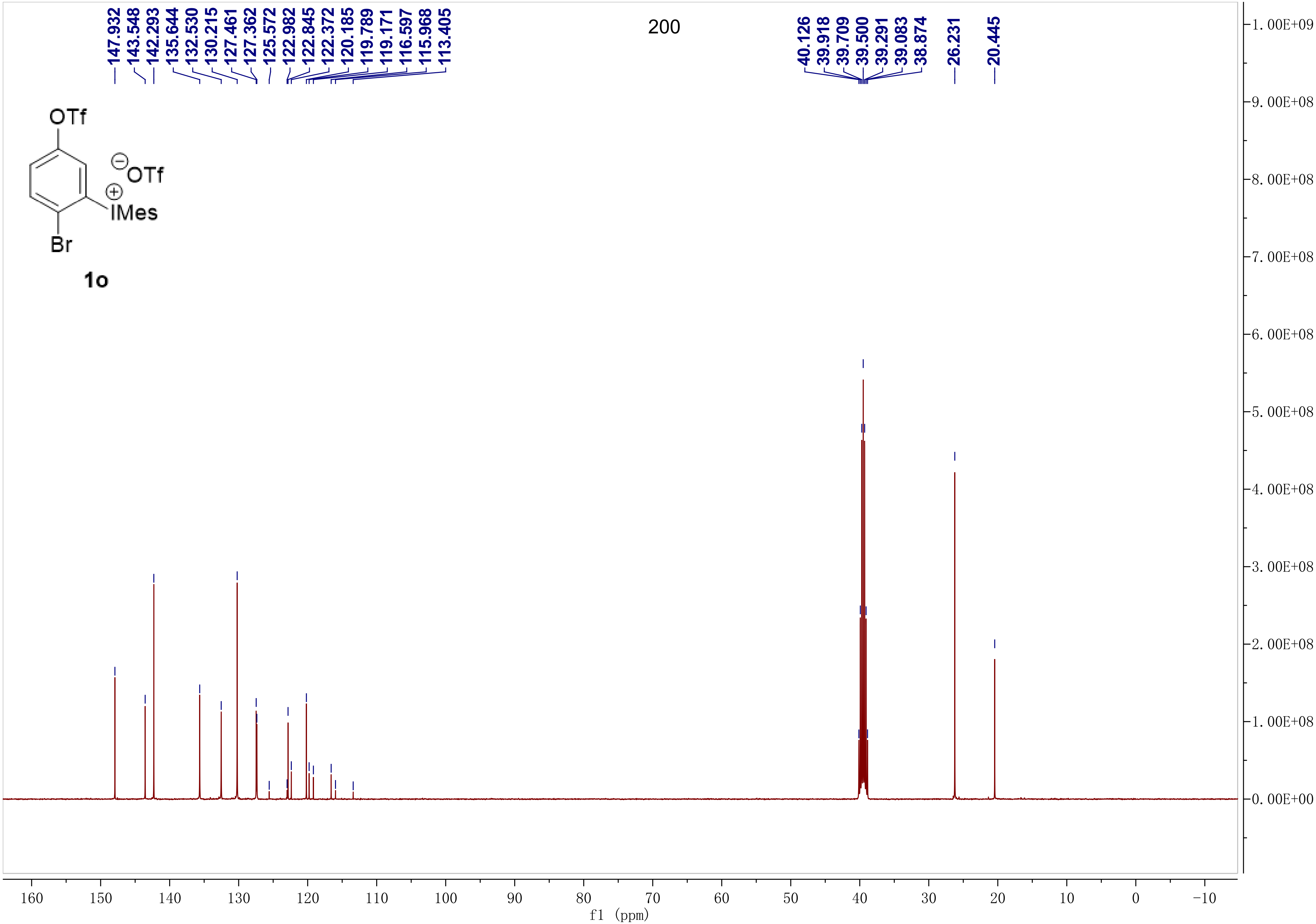

Supplementary Fig 123. ^{13}C NMR spectrum (100 MHz, DMSO-*d*6, r.t.) of 1o.

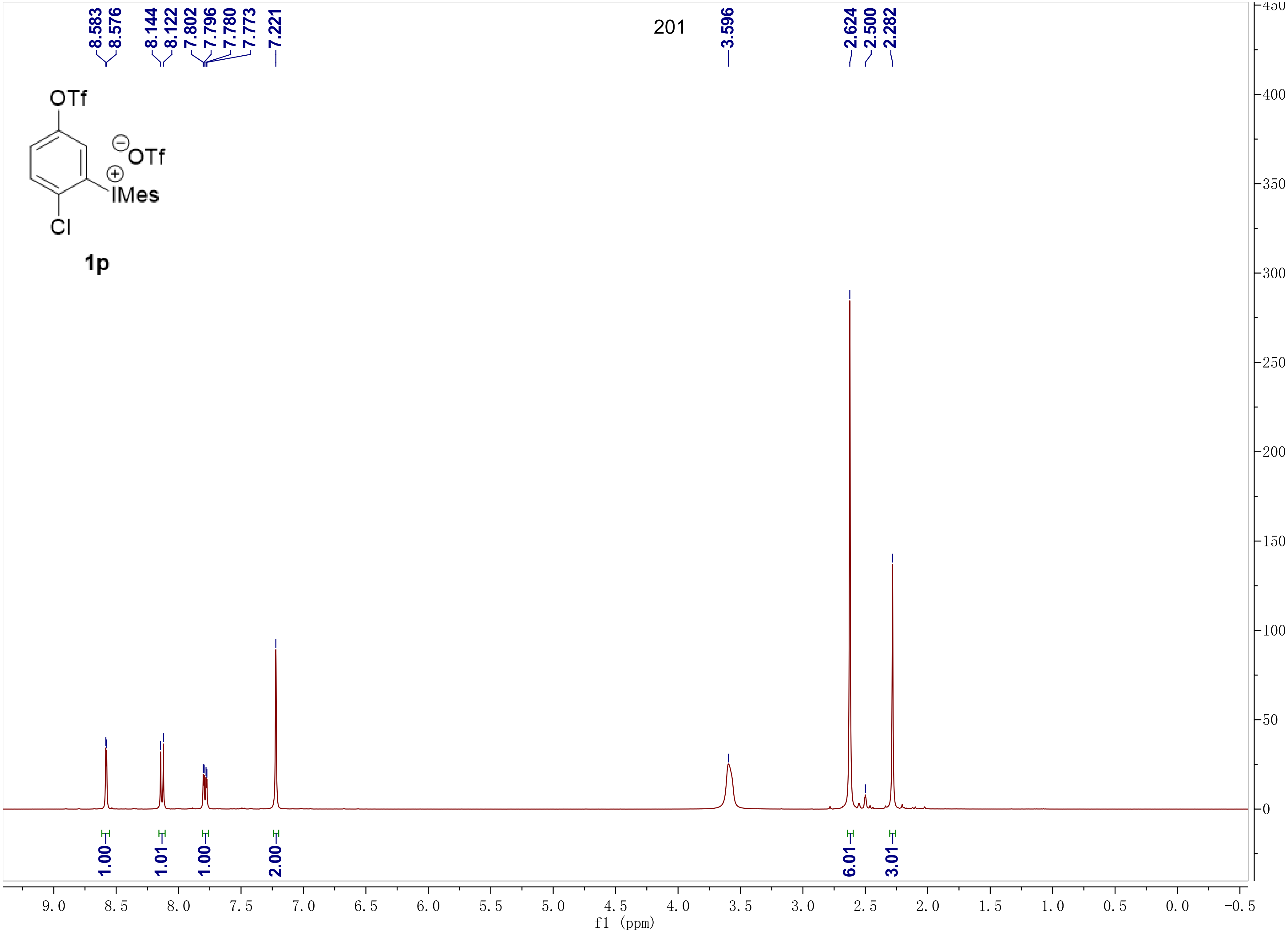

Supplementary Fig 124. **^{1}H NMR spectrum (400 MHz, DMSO-*d*6, r.t.) of 1p.**

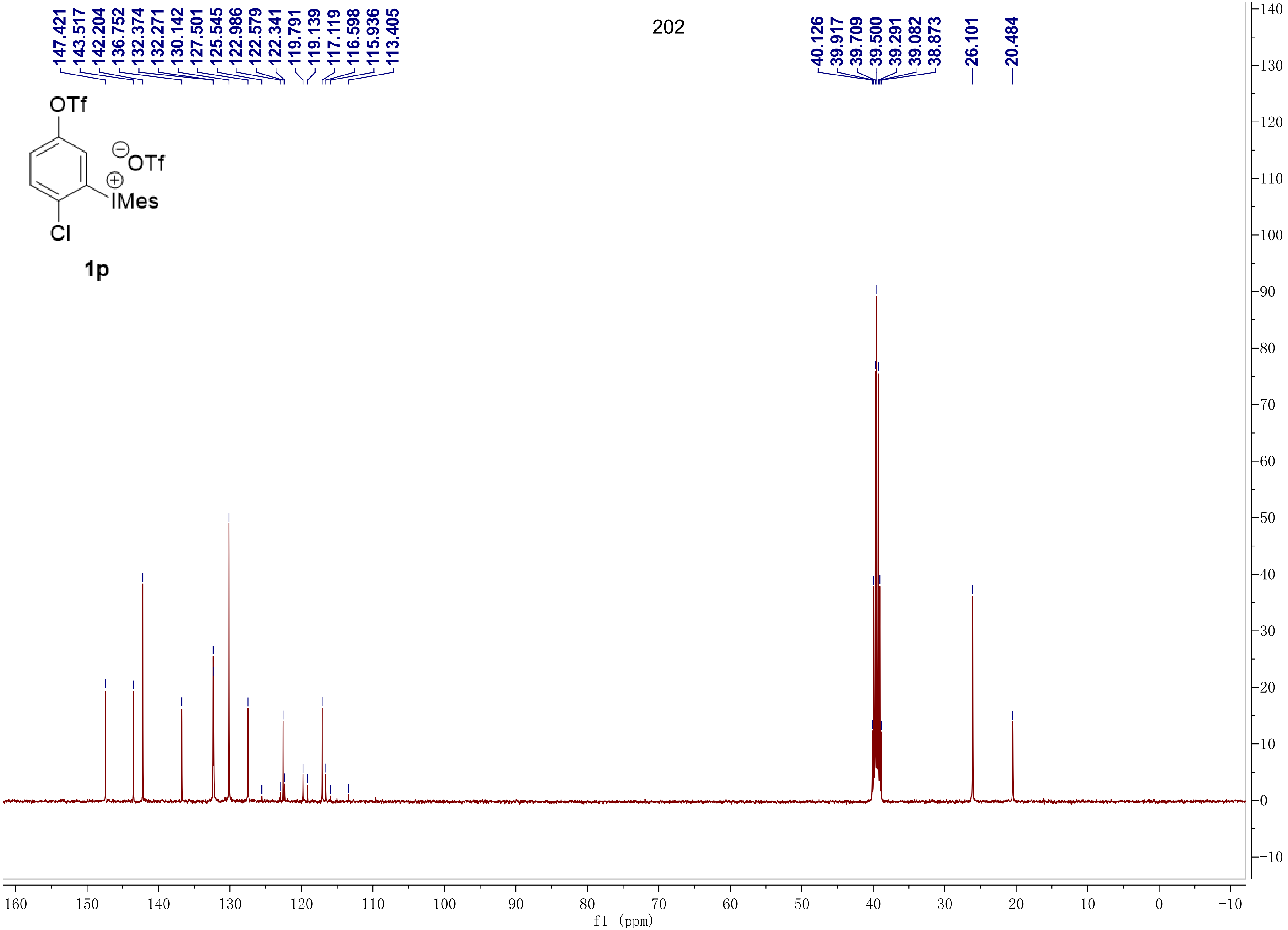

Supplementary Fig 125. ^{13}C NMR spectrum (100 MHz, DMSO-*d*6, r.t.) of 1p.

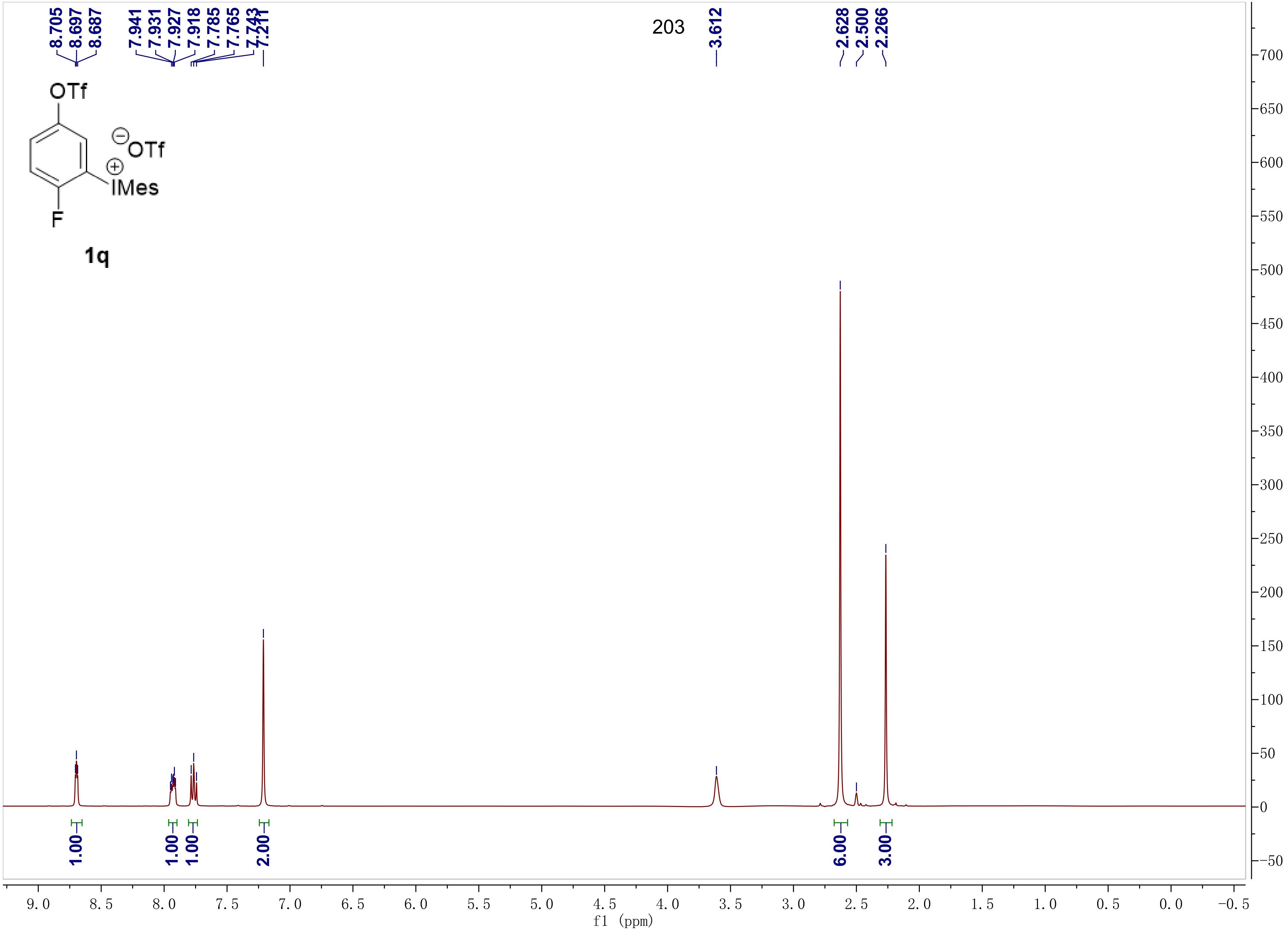

Supplementary Fig 126. **^{1}H NMR spectrum (400 MHz, DMSO-*d*6, r.t.) of 1q.**

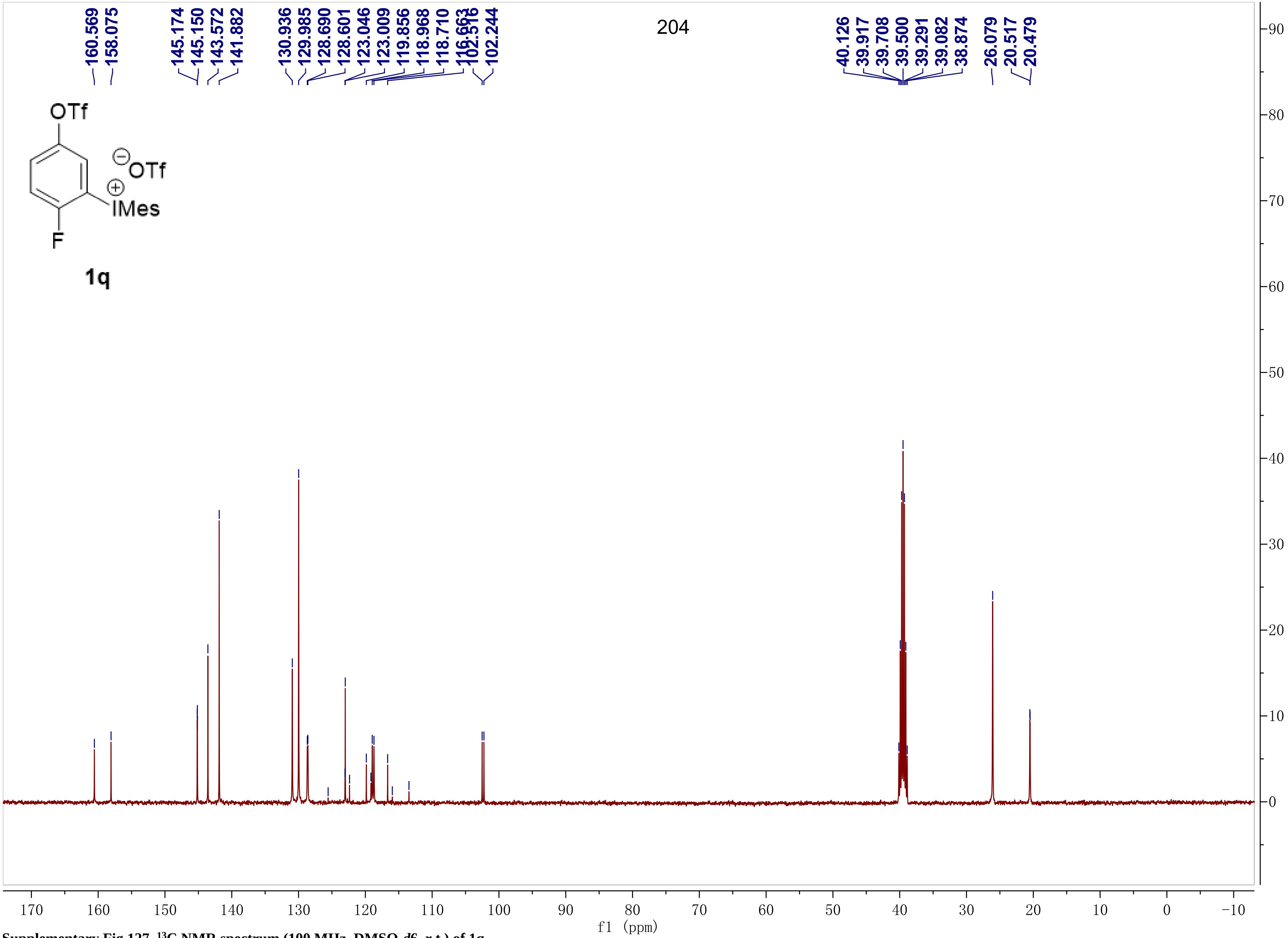

Supplementary Fig 127. ^{13}C NMR spectrum (100 MHz, DMSO-*d*6, r.t.) of 1q.

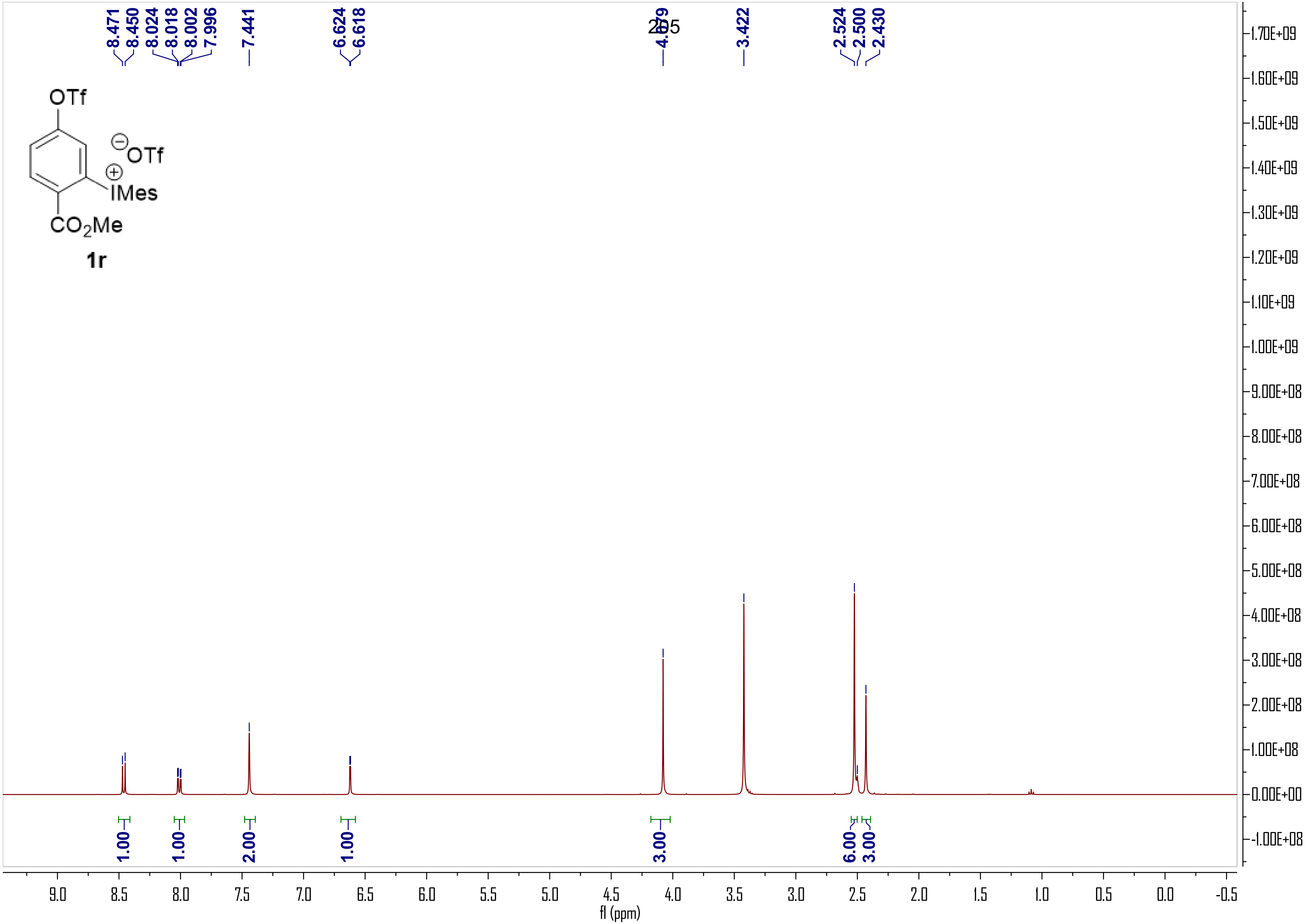

Supplementary Fig 128. ^{1}H NMR spectrum (400 MHz, DMSO-*d*6, r.t.) of 1r.

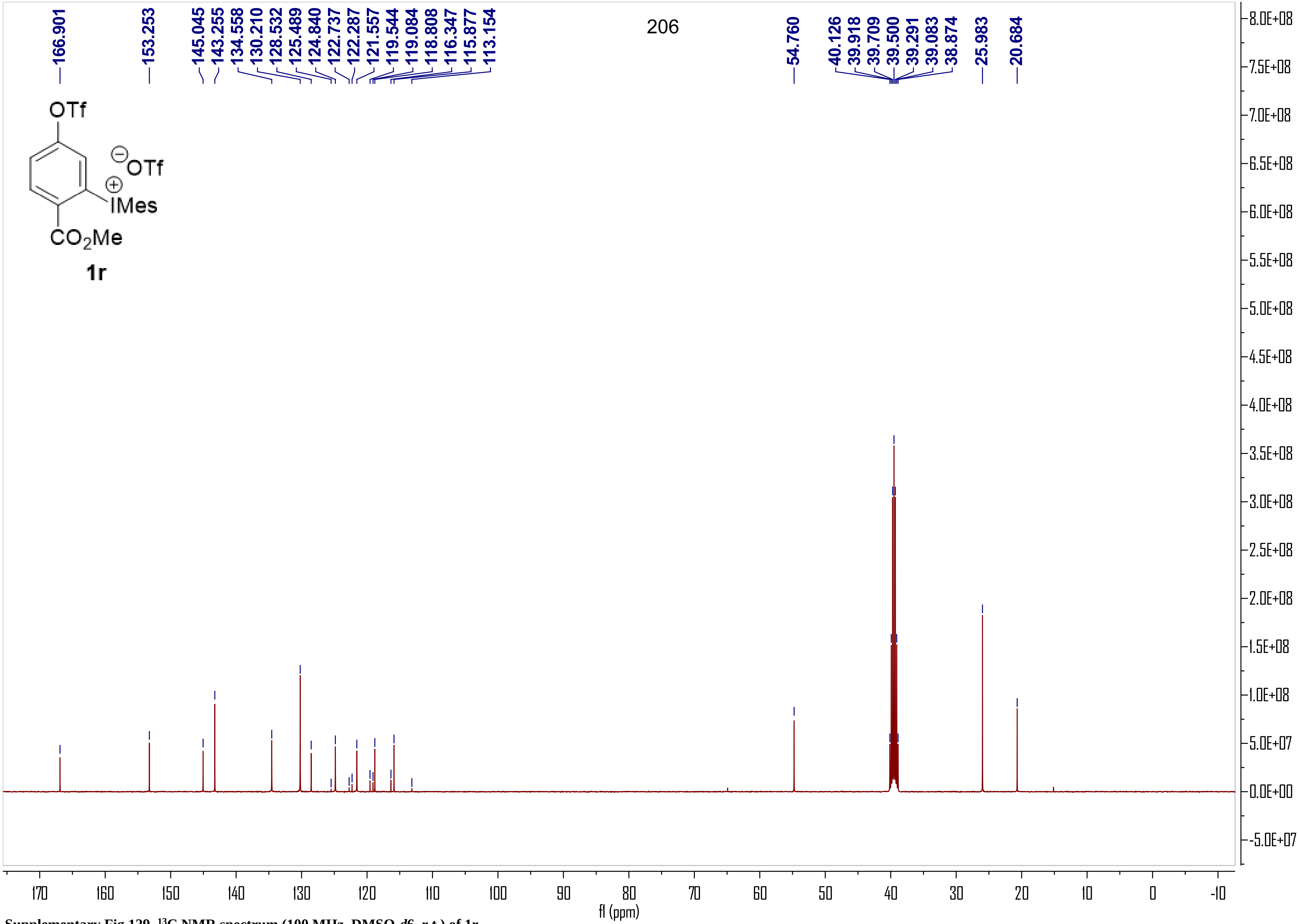

Supplementary Fig 129. **^{13}C NMR spectrum (100 MHz, DMSO-*d*6, r.t.) of 1r.**

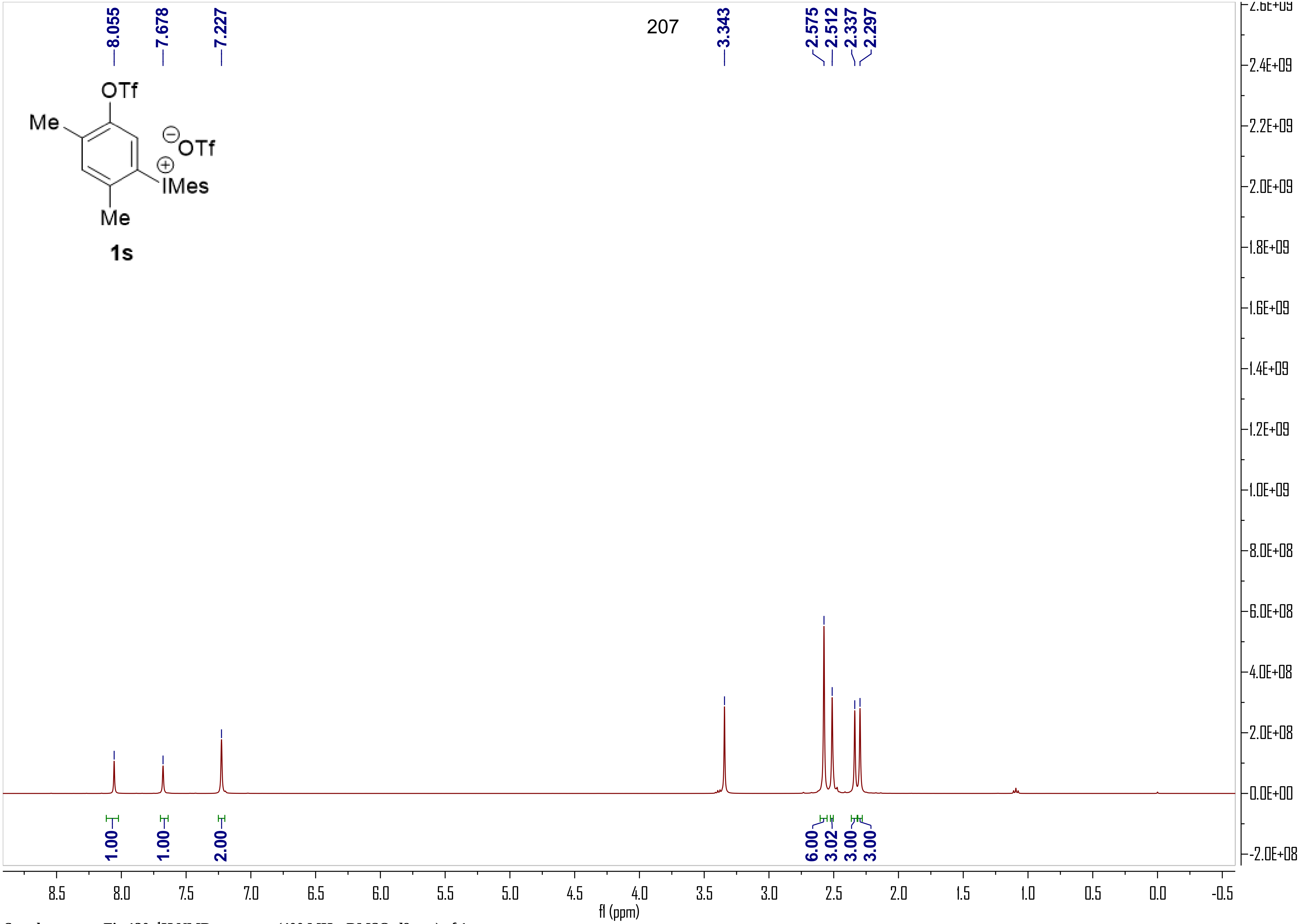

Supplementary Fig 130. **^{1}H NMR spectrum (400 MHz, DMSO-*d*6, r.t.) of 1s.**

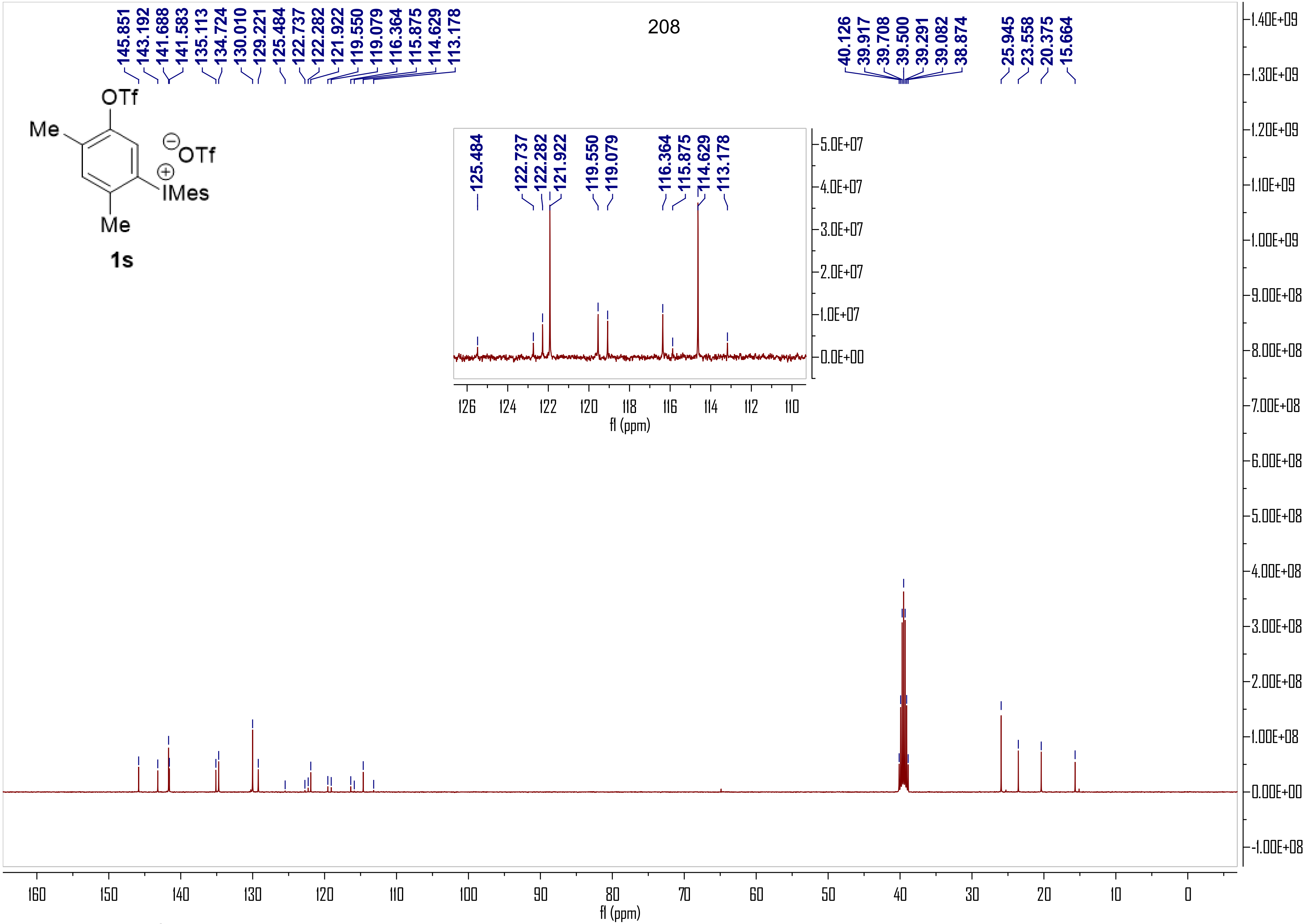

Supplementary Fig 131. ^{13}**C NMR spectrum (100 MHz, DMSO-*d*6, r.t.) of 1s.**

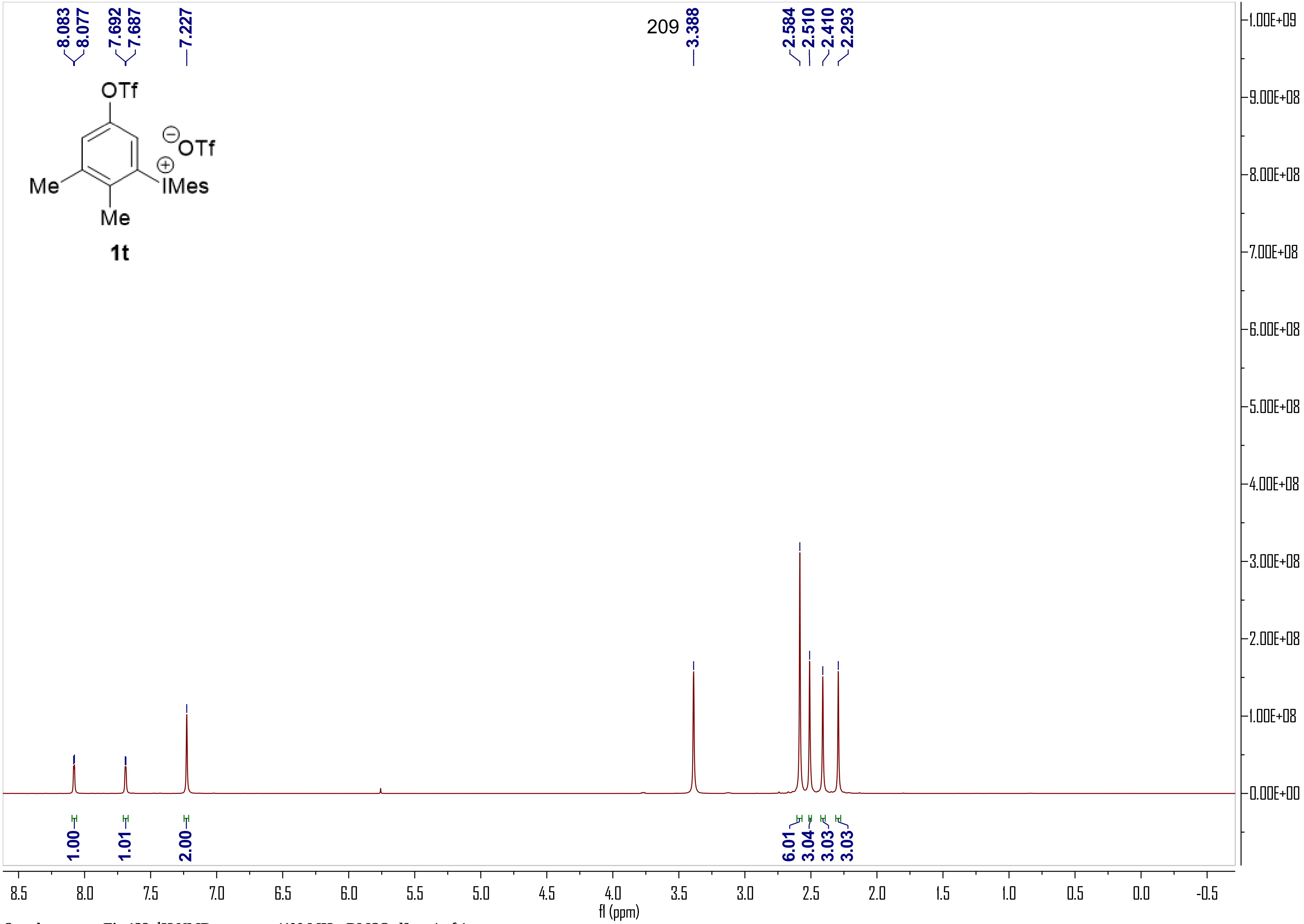

Supplementary Fig 132. ^{1}**H NMR spectrum (400 MHz, DMSO-*d*6, r.t.) of 1t.**

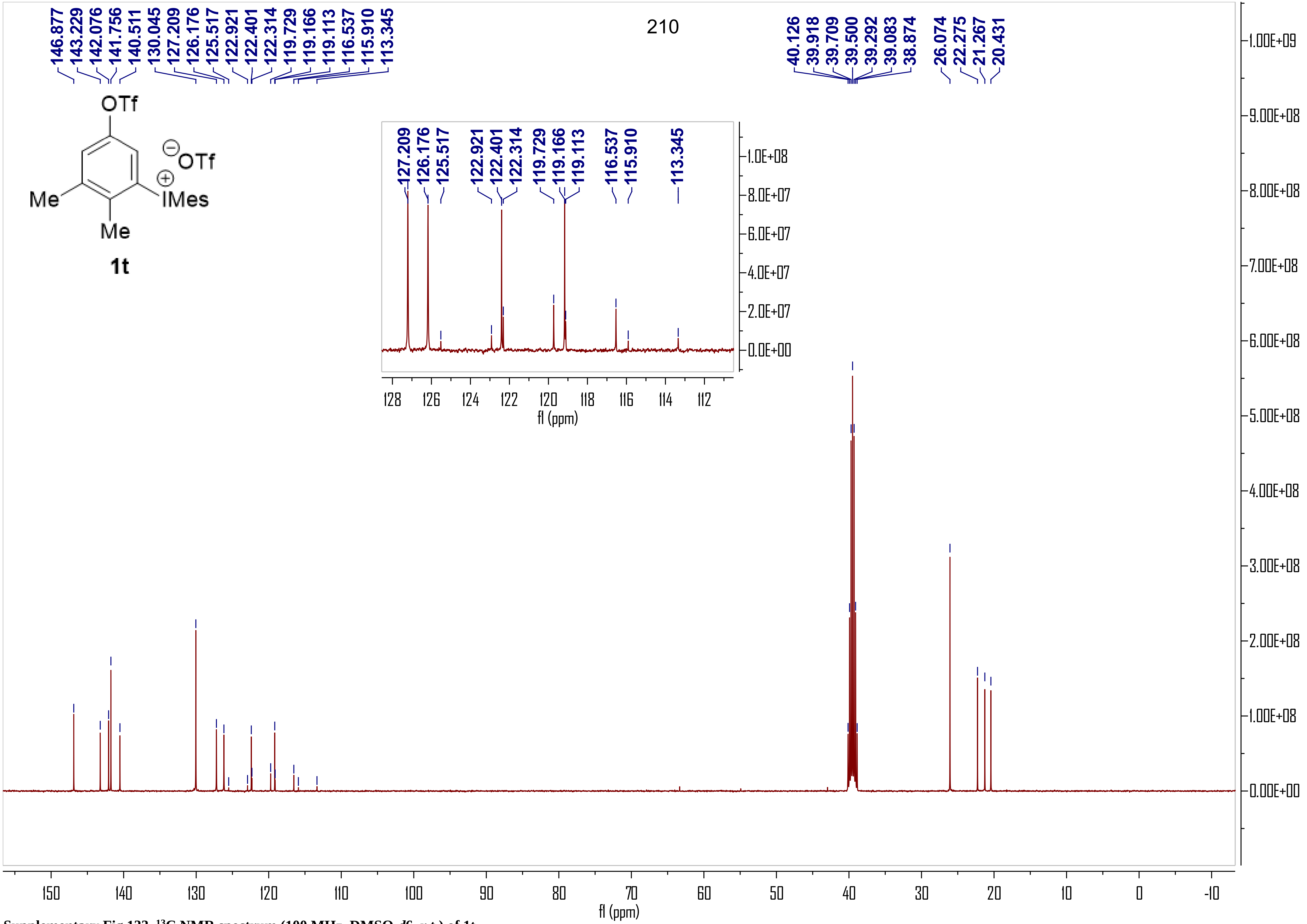

Supplementary Fig 133. ^{13}**C NMR spectrum (100 MHz, DMSO-*d*6, r.t.) of 1t.**

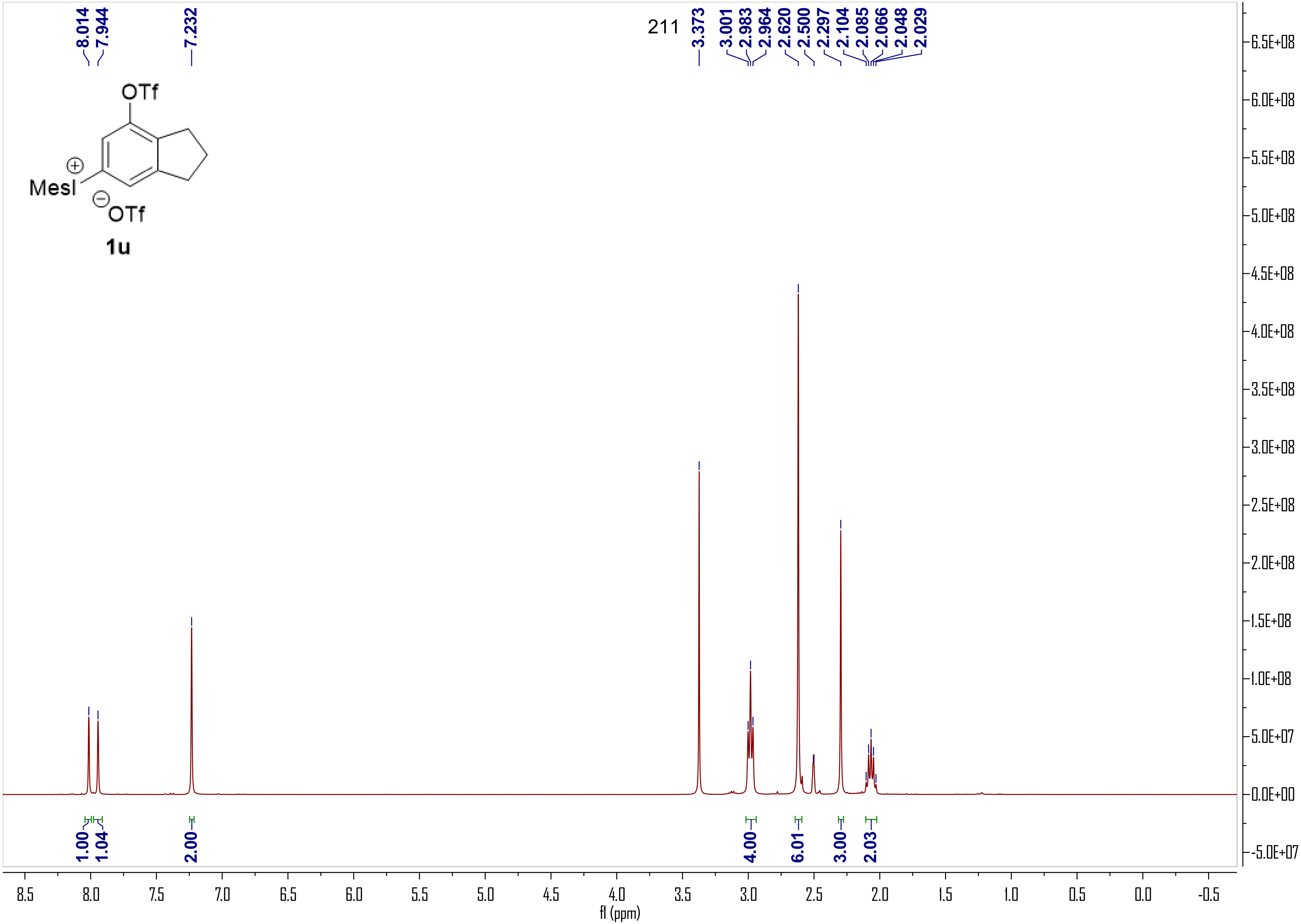

Supplementary Fig 134. **^{1}H NMR spectrum (400 MHz, DMSO-*d*6, r.t.) of 1u.**

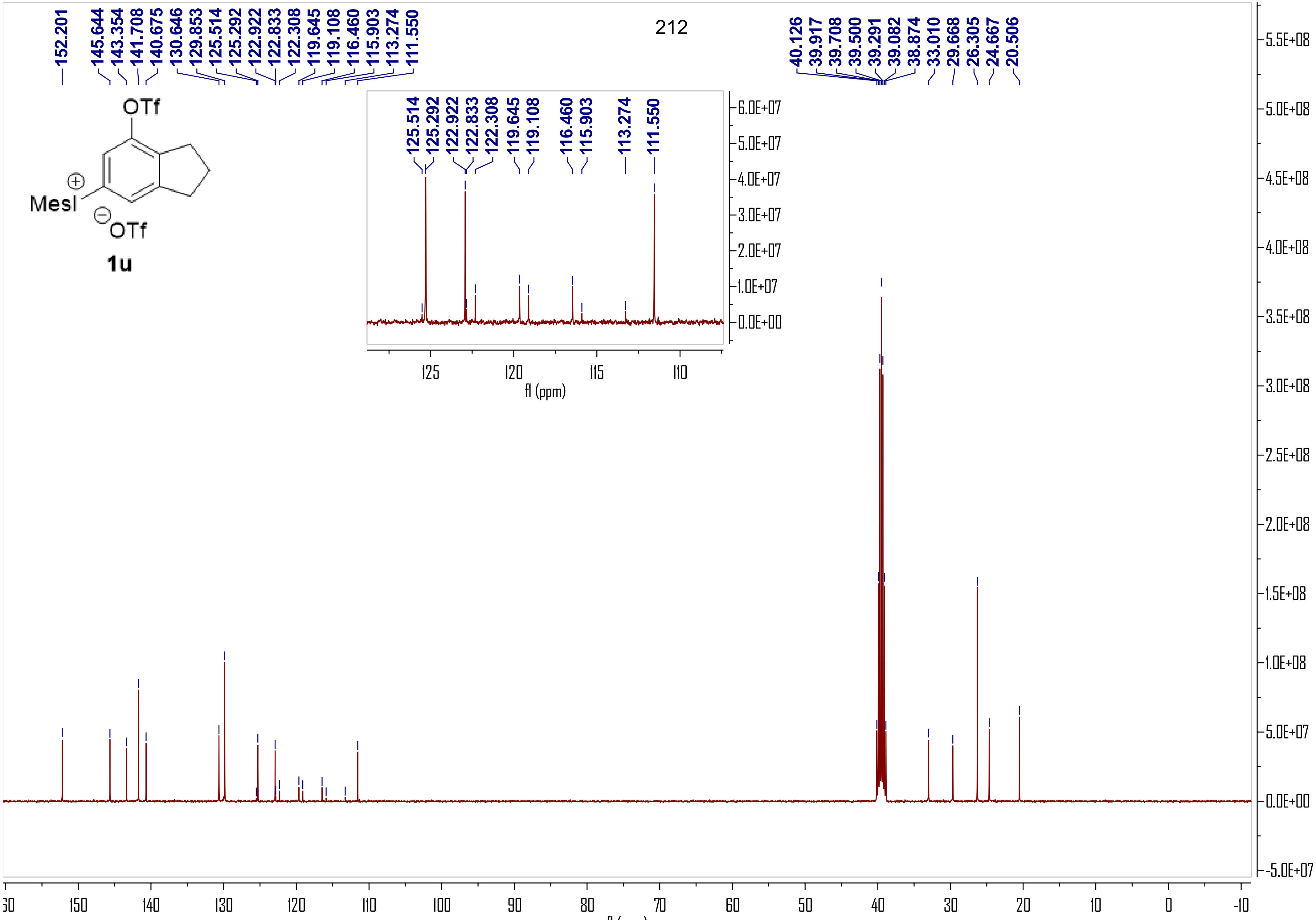

Supplementary Fig 135. ^{13}**C NMR spectrum (100 MHz, DMSO-*d*6, r.t.) of 1u.**

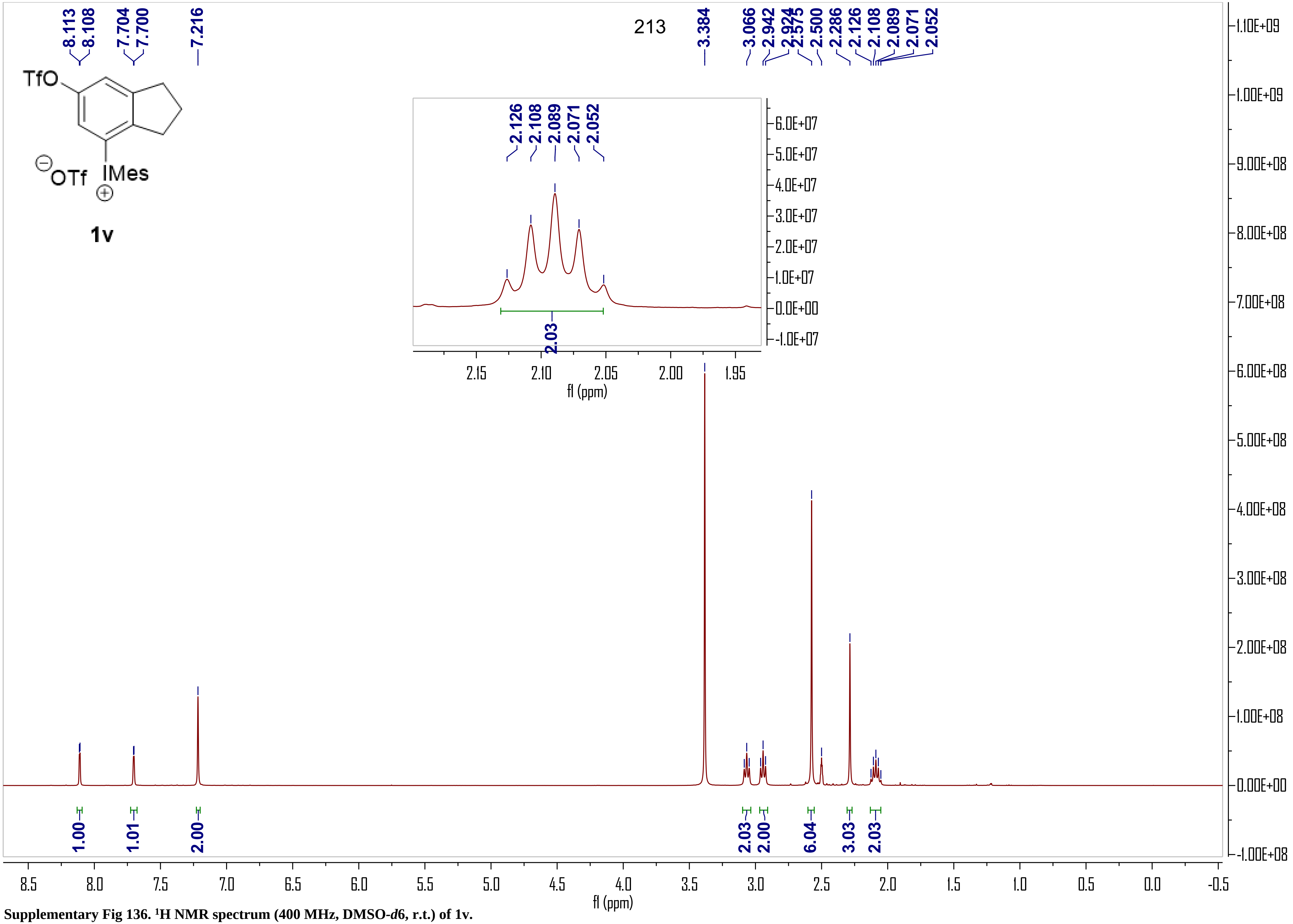

Supplementary Fig 136. ^{1}**H NMR spectrum (400 MHz, DMSO-*d*6, r.t.) of 1v.**

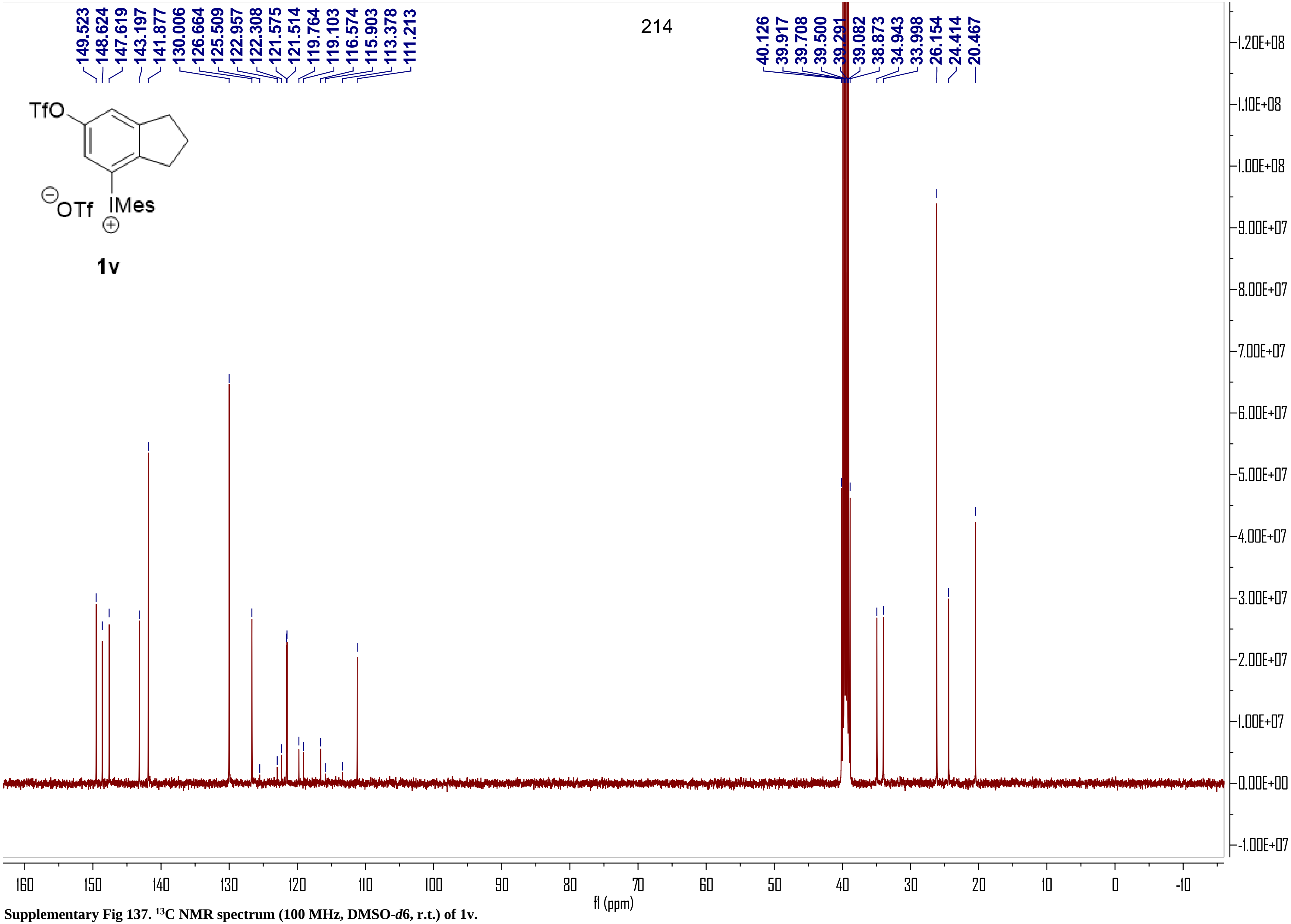

Supplementary Fig 137. ^{13}C NMR spectrum (100 MHz, DMSO-*d*6, r.t.) of 1v.

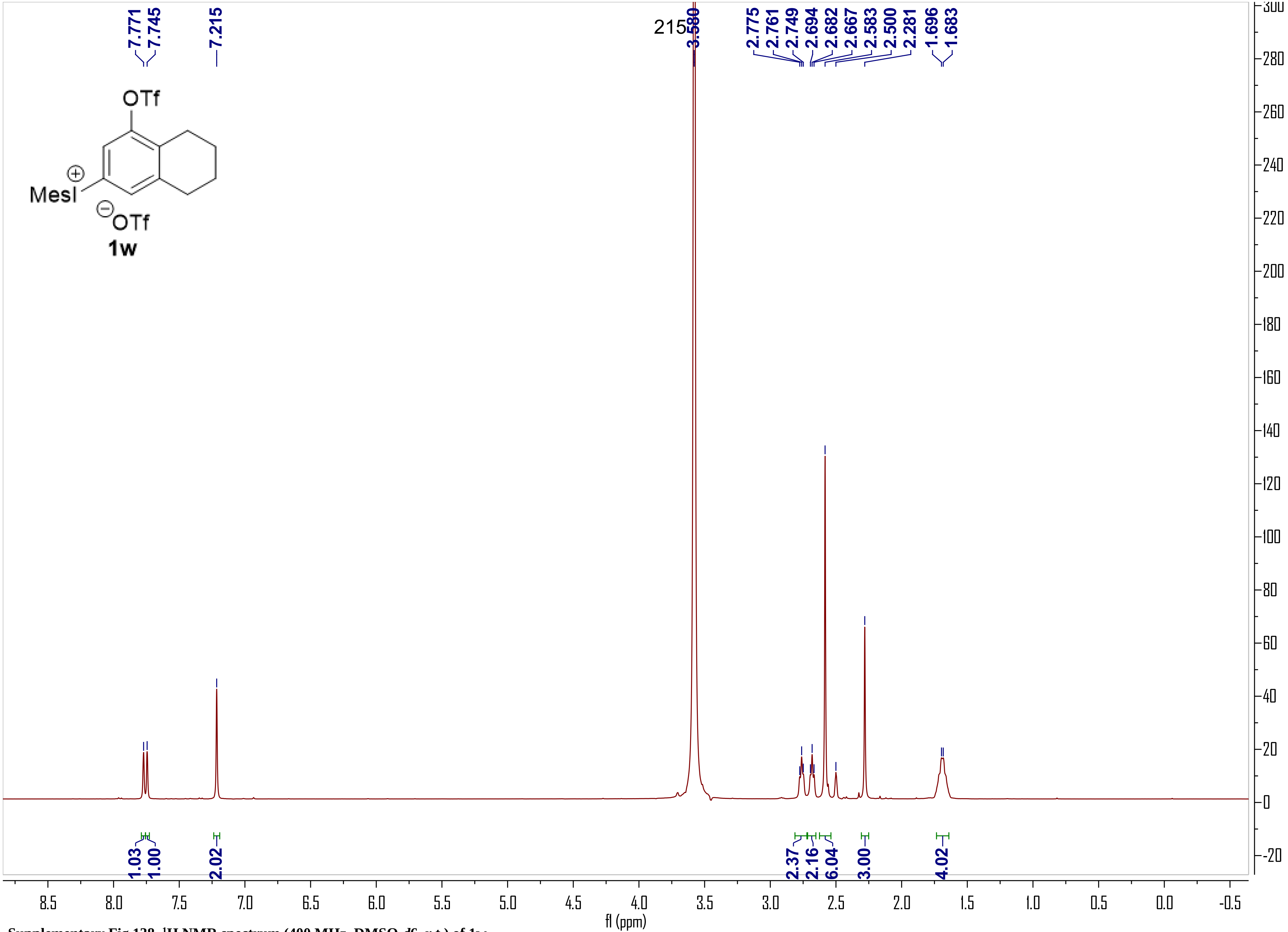

Supplementary Fig 138. ¹H NMR spectrum (400 MHz, DMSO-*d*6, r.t.) of 1w.

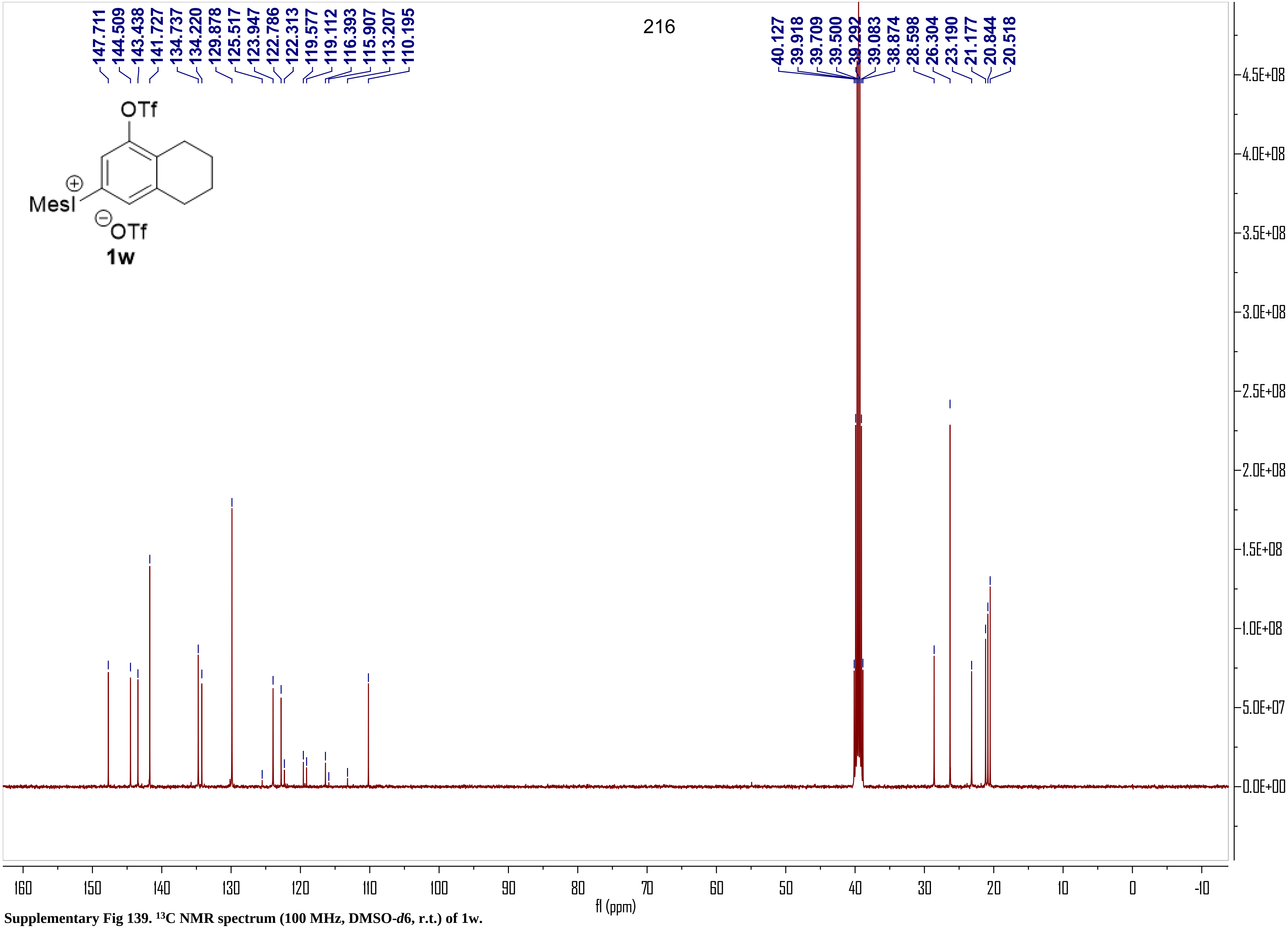

Supplementary Fig 139. ^{13}**C NMR spectrum (100 MHz, DMSO-*d*6, r.t.) of 1w.**

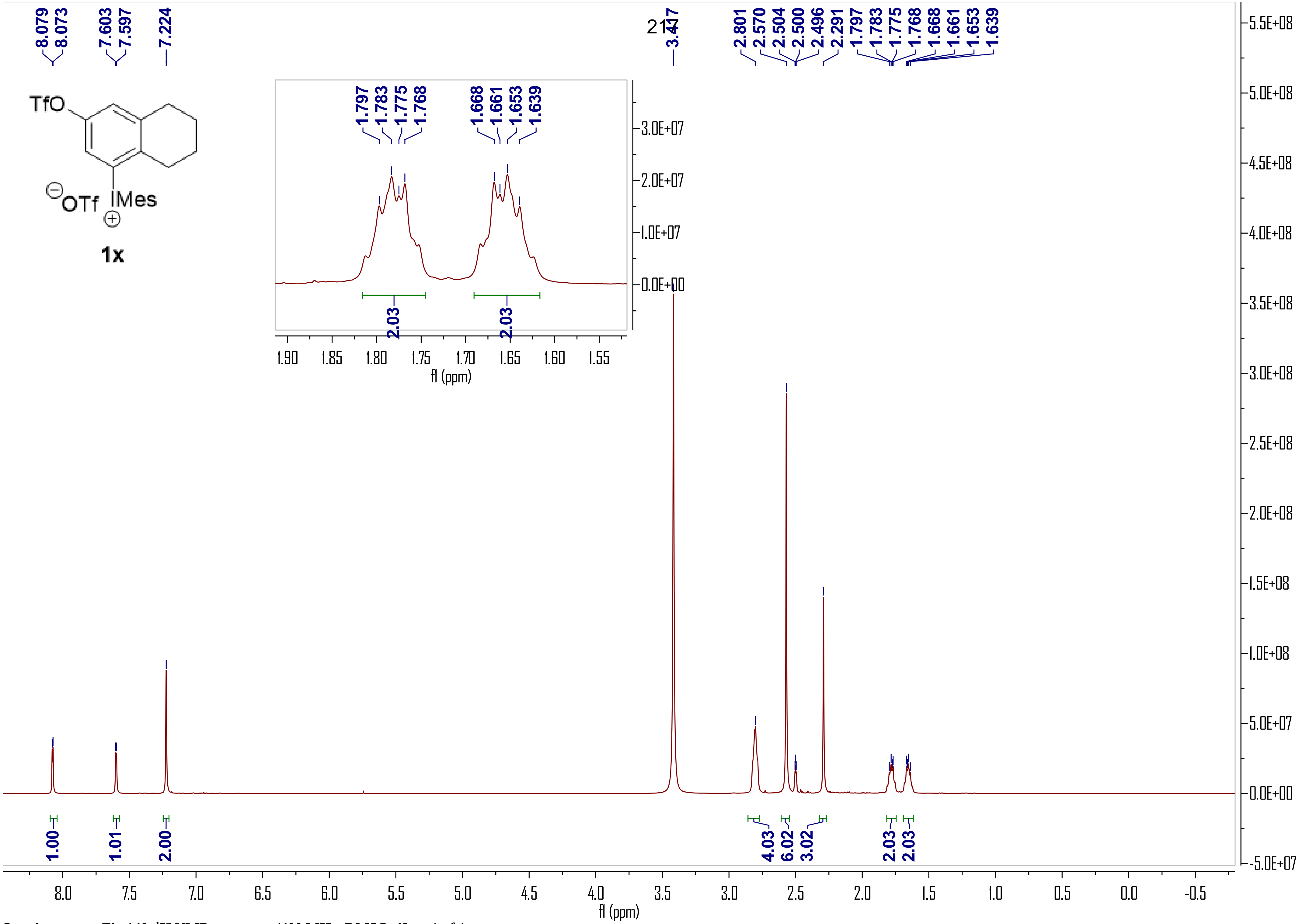

Supplementary Fig 140. ^{1}**H NMR spectrum (400 MHz, DMSO-*d*6, r.t.) of 1x.**

218

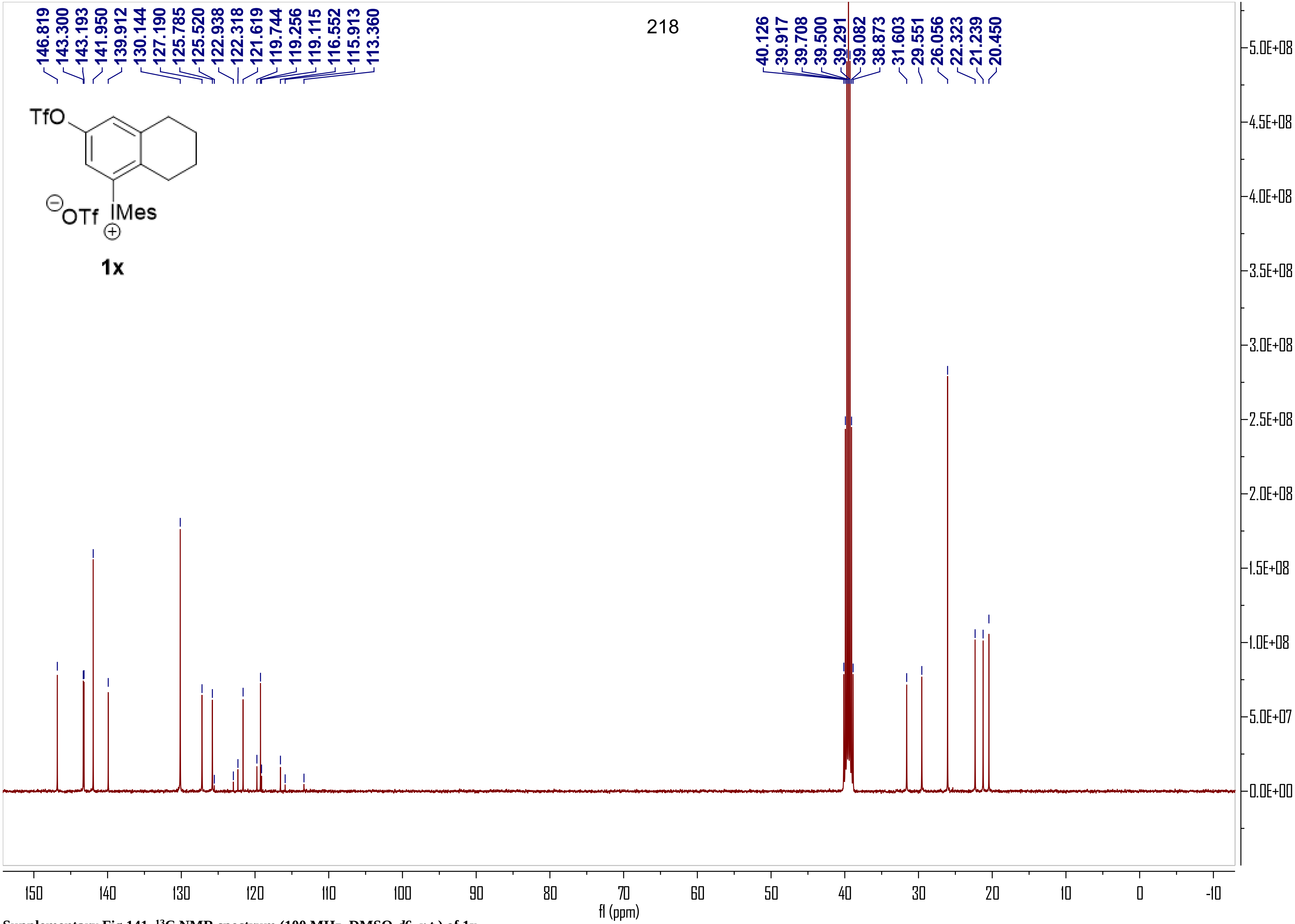

Supplementary Fig 141. ^{13}**C NMR spectrum (100 MHz, DMSO-*d*6, r.t.) of 1x.**

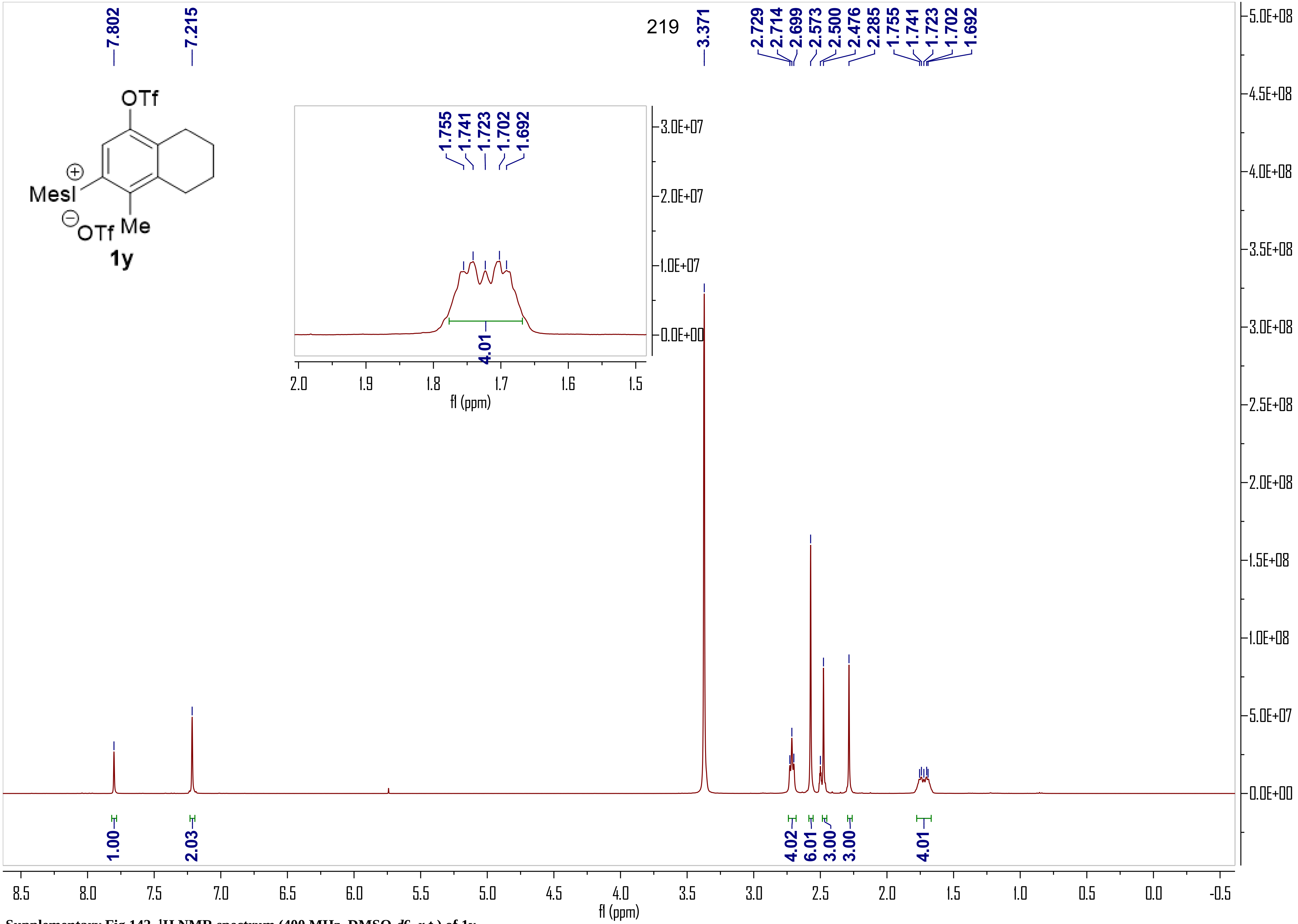

Supplementary Fig 142. **^{1}H NMR spectrum (400 MHz, DMSO-*d6*, r.t.) of 1y.**

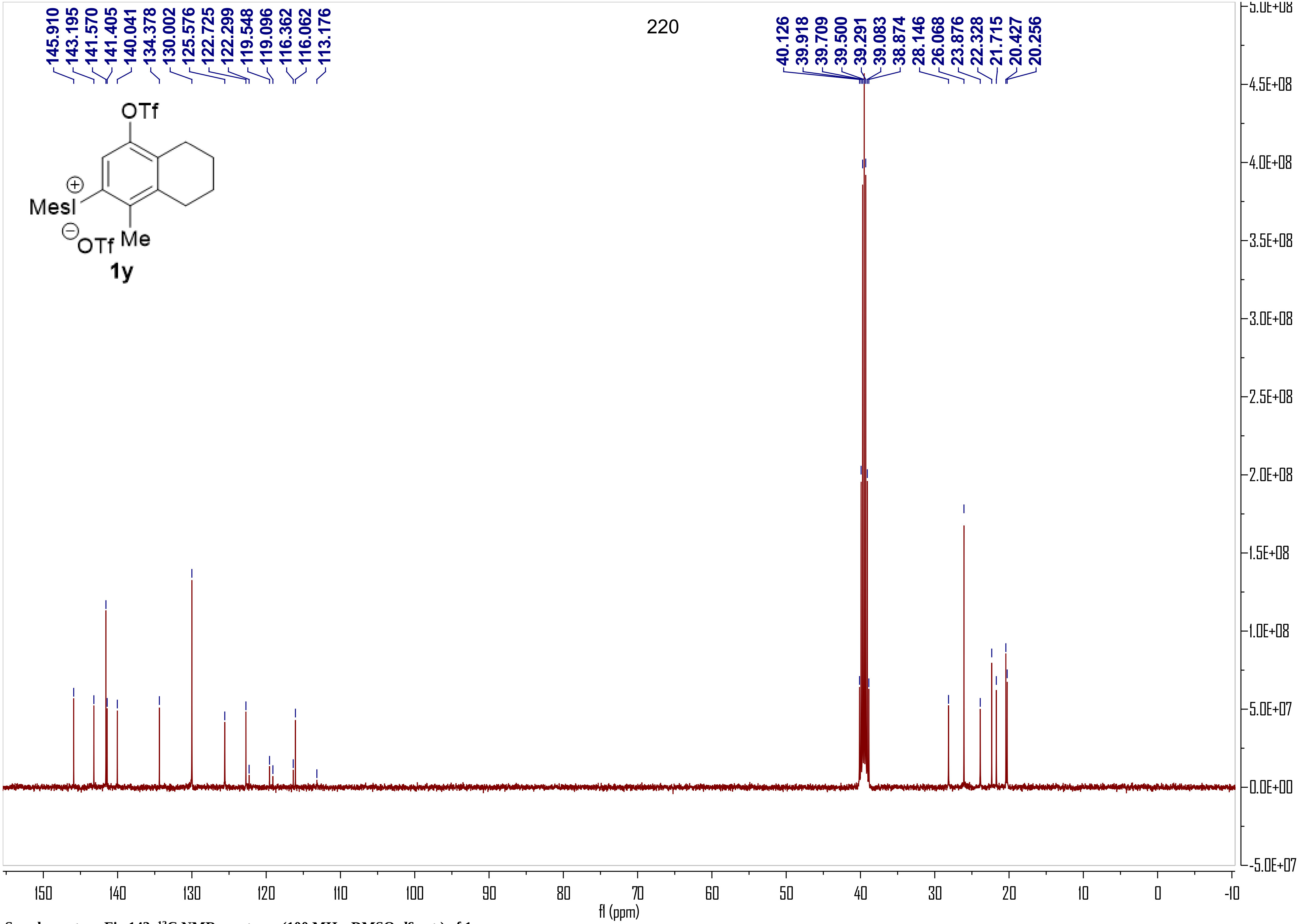

Supplementary Fig 143. **^{13}C NMR spectrum (100 MHz, DMSO-*d*6, r.t.) of 1y.**

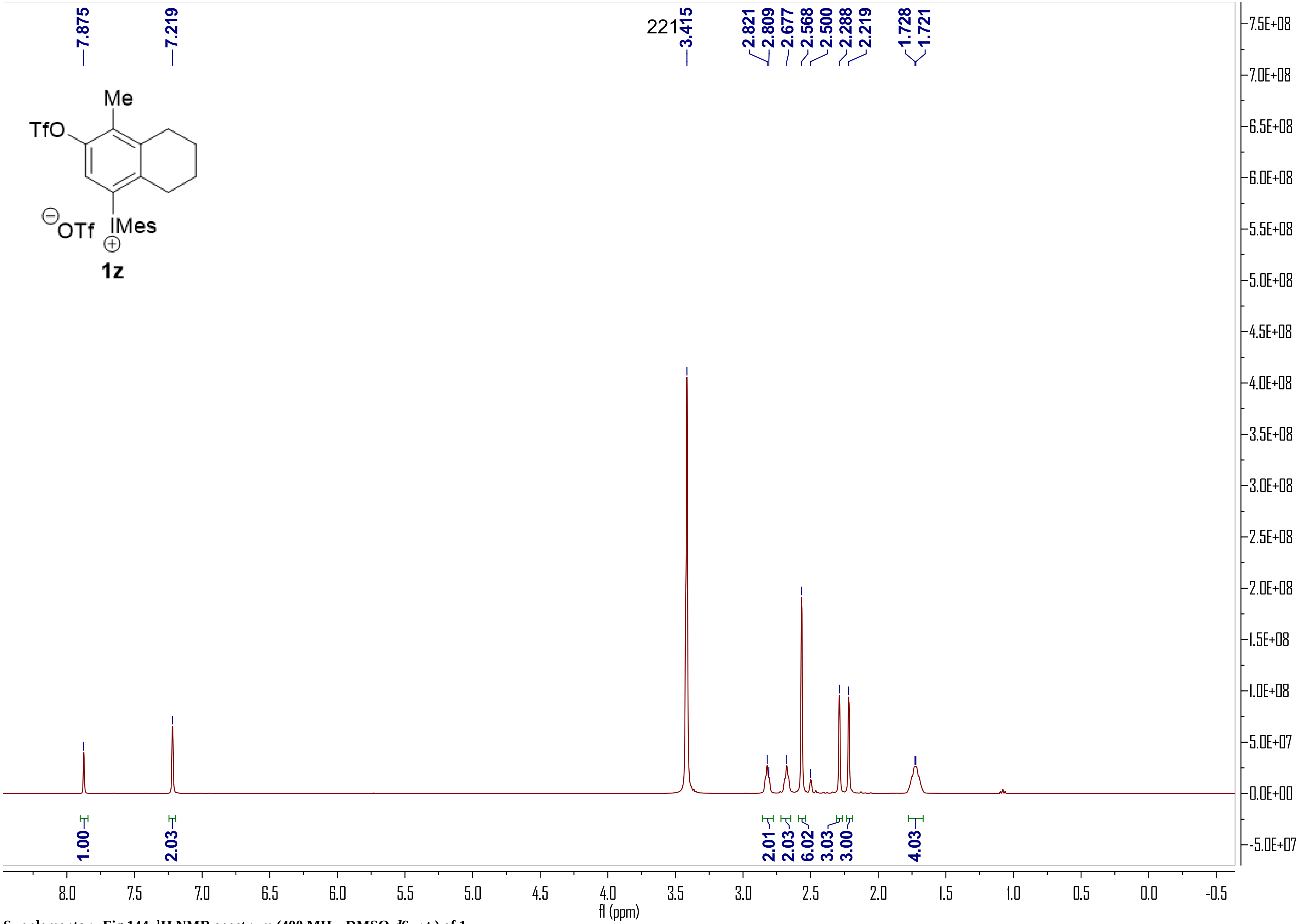

Supplementary Fig 144. **^{1}H NMR spectrum (400 MHz, DMSO-*d*6, r.t.) of 1z.**

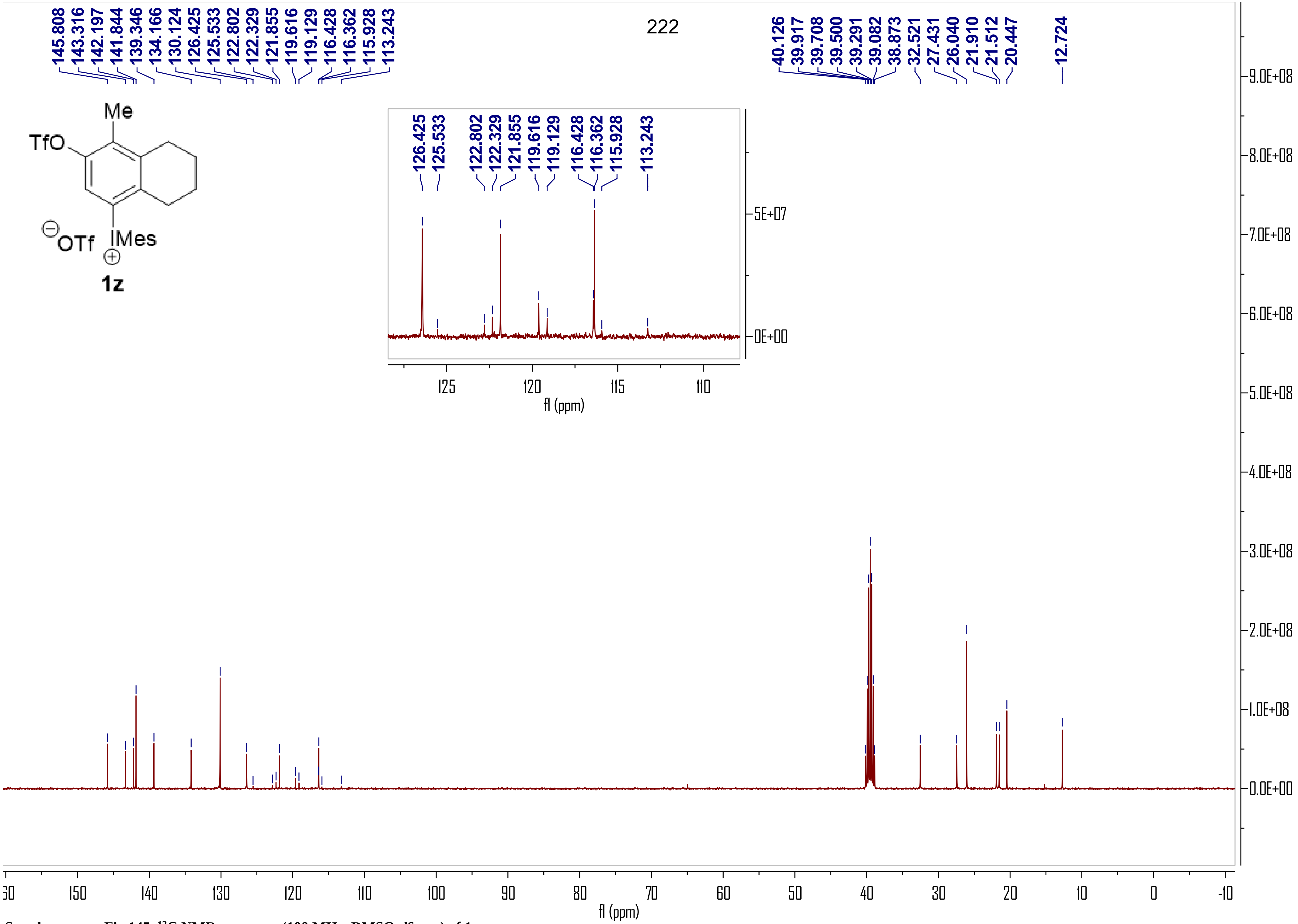

Supplementary Fig 145. **^{13}C NMR spectrum (100 MHz, DMSO-*d*6, r.t.) of 1z.**

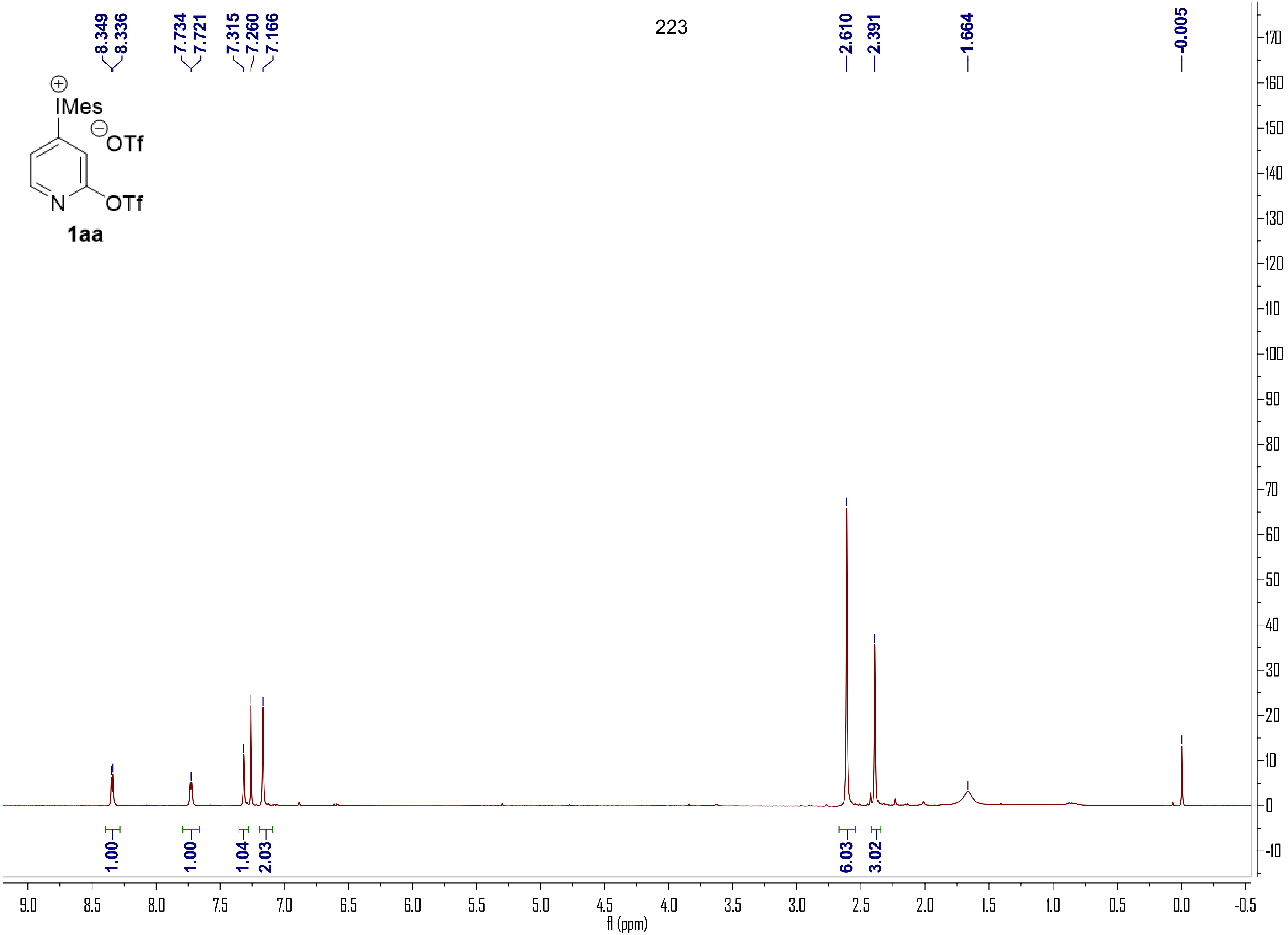

Supplementary Fig 146. **^{1}H NMR spectrum (400 MHz, $CDCl_3$, r.t.) of 1aa.**

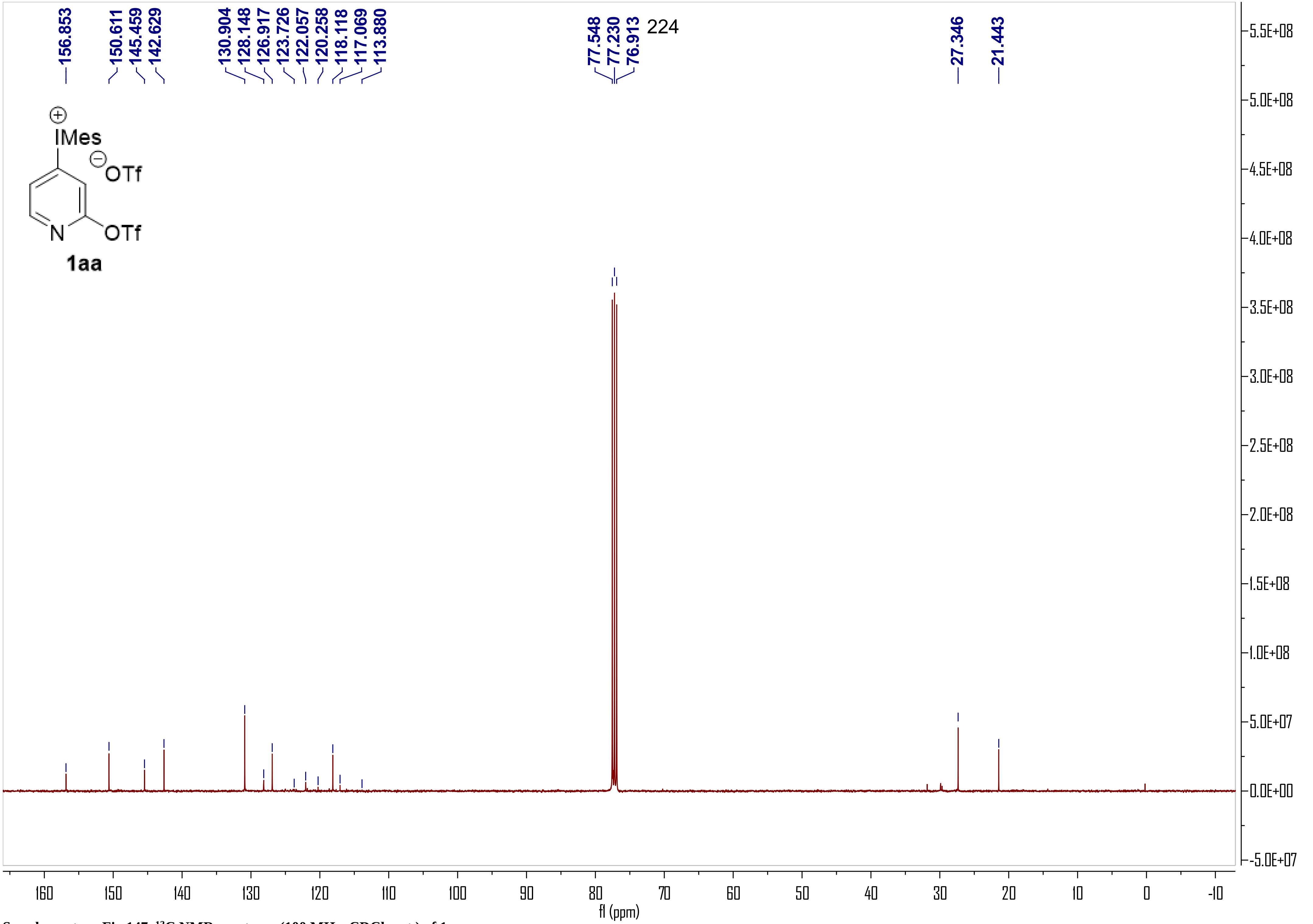

Supplementary Fig 147. **^{13}C NMR spectrum (100 MHz, $CDCl_3$, r.t.) of 1aa.**

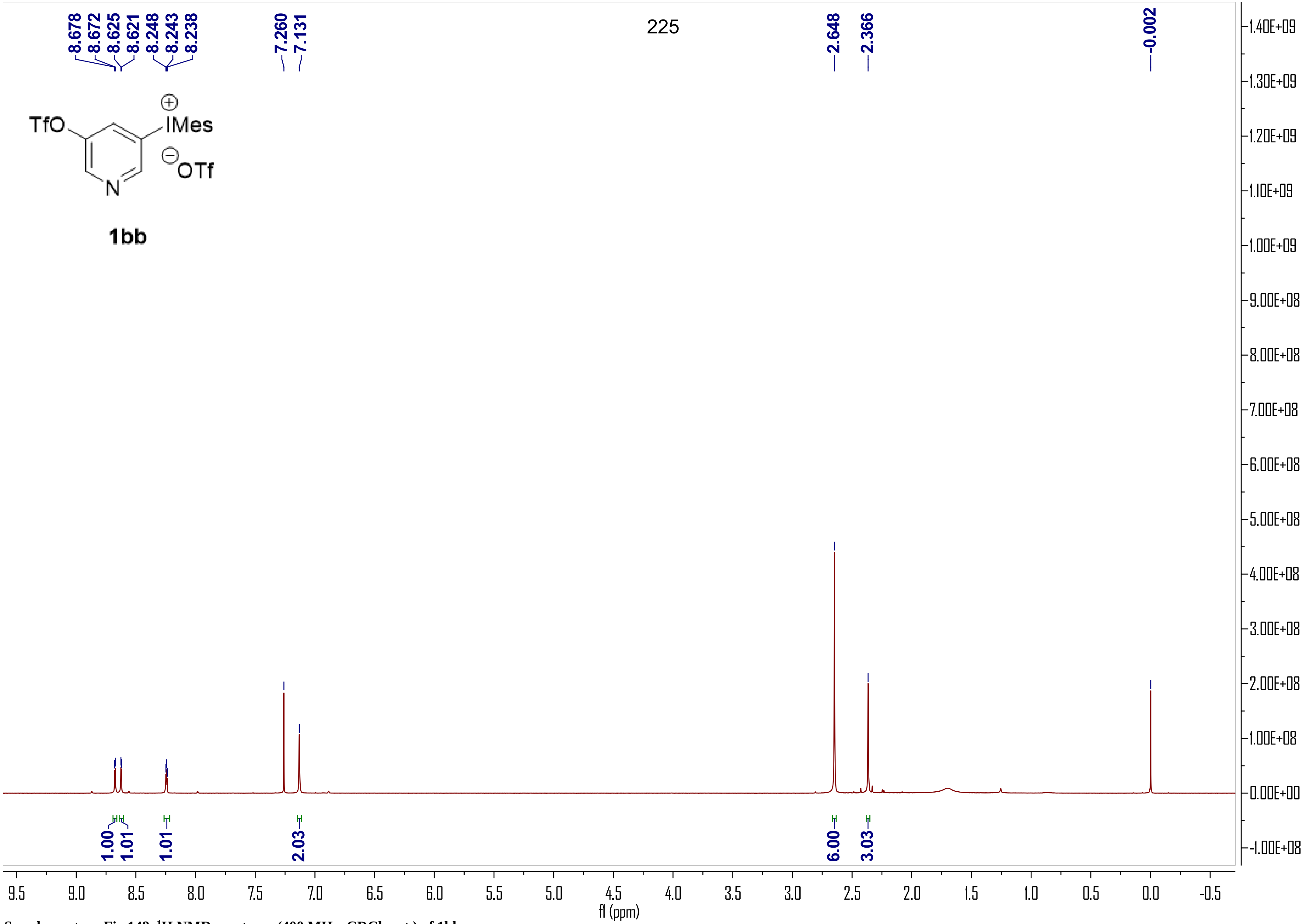

Supplementary Fig 148. ^{1}H NMR spectrum (400 MHz, $CDCl_3$, r.t.) of 1bb.

226

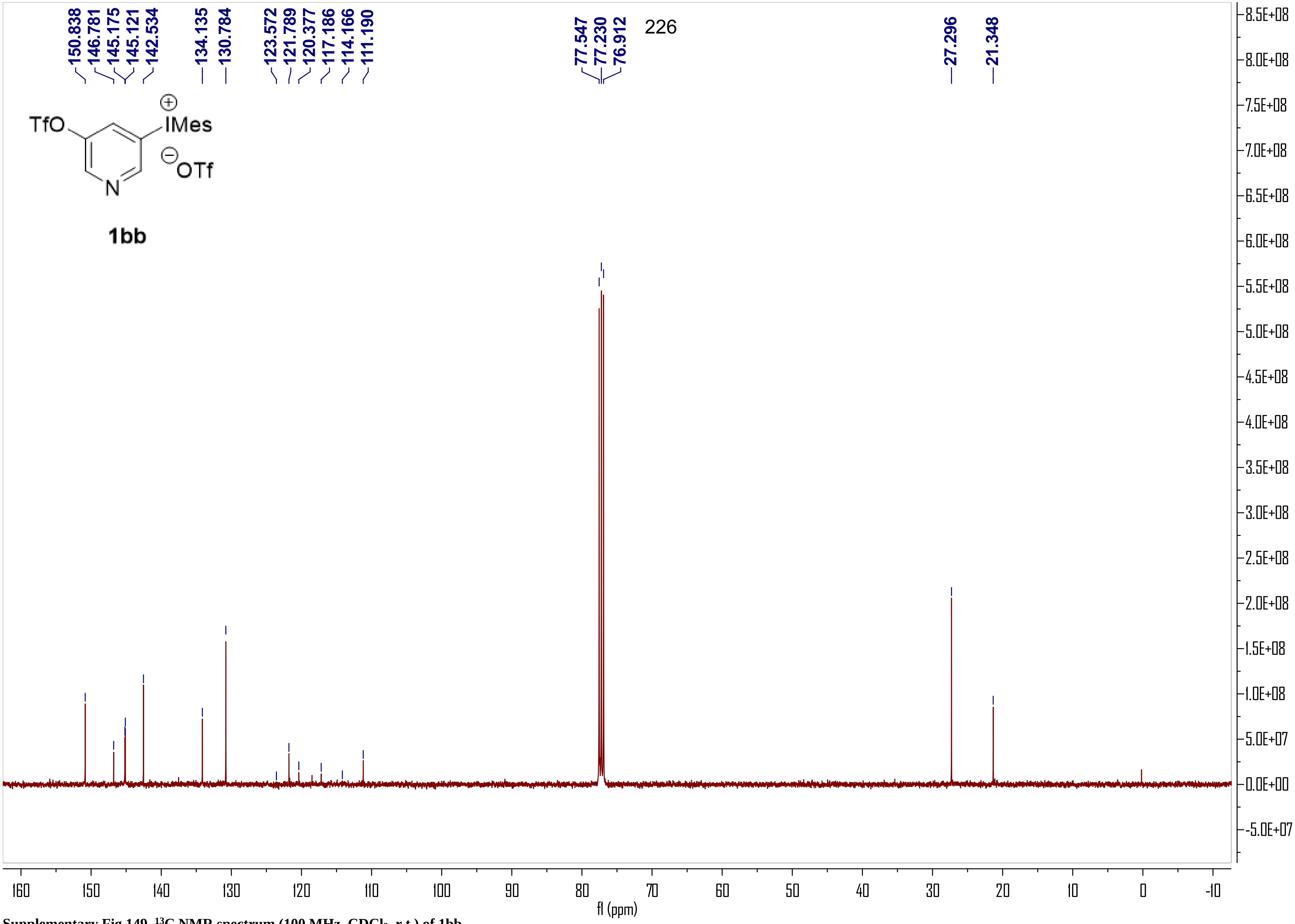

Supplementary Fig 149. ^{13}C **NMR spectrum (100 MHz, $CDCl_3$, r.t.) of 1bb.**

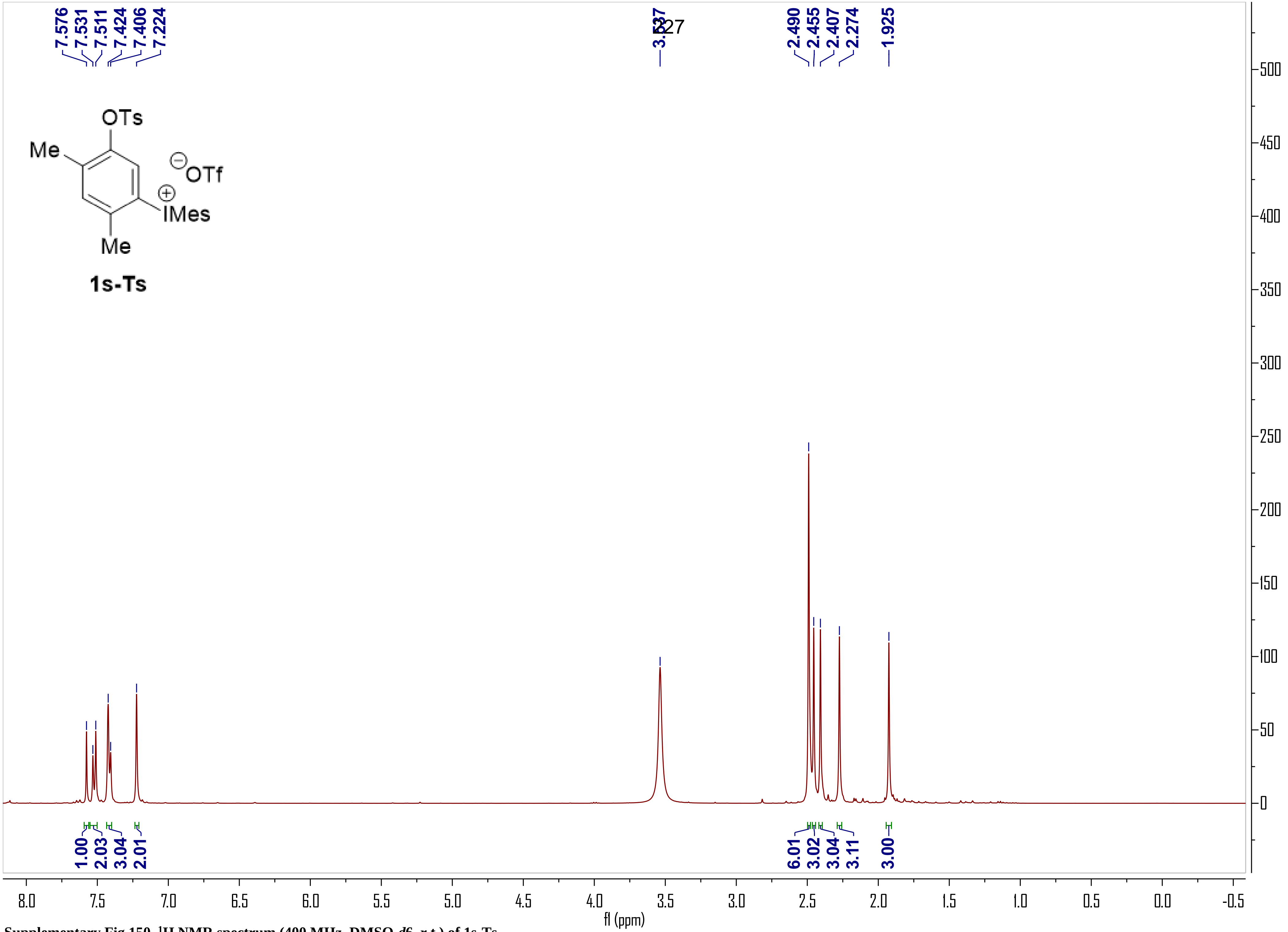

Supplementary Fig 150. **¹H NMR spectrum (400 MHz, DMSO-*d*6, r.t.) of 1s-Ts.**

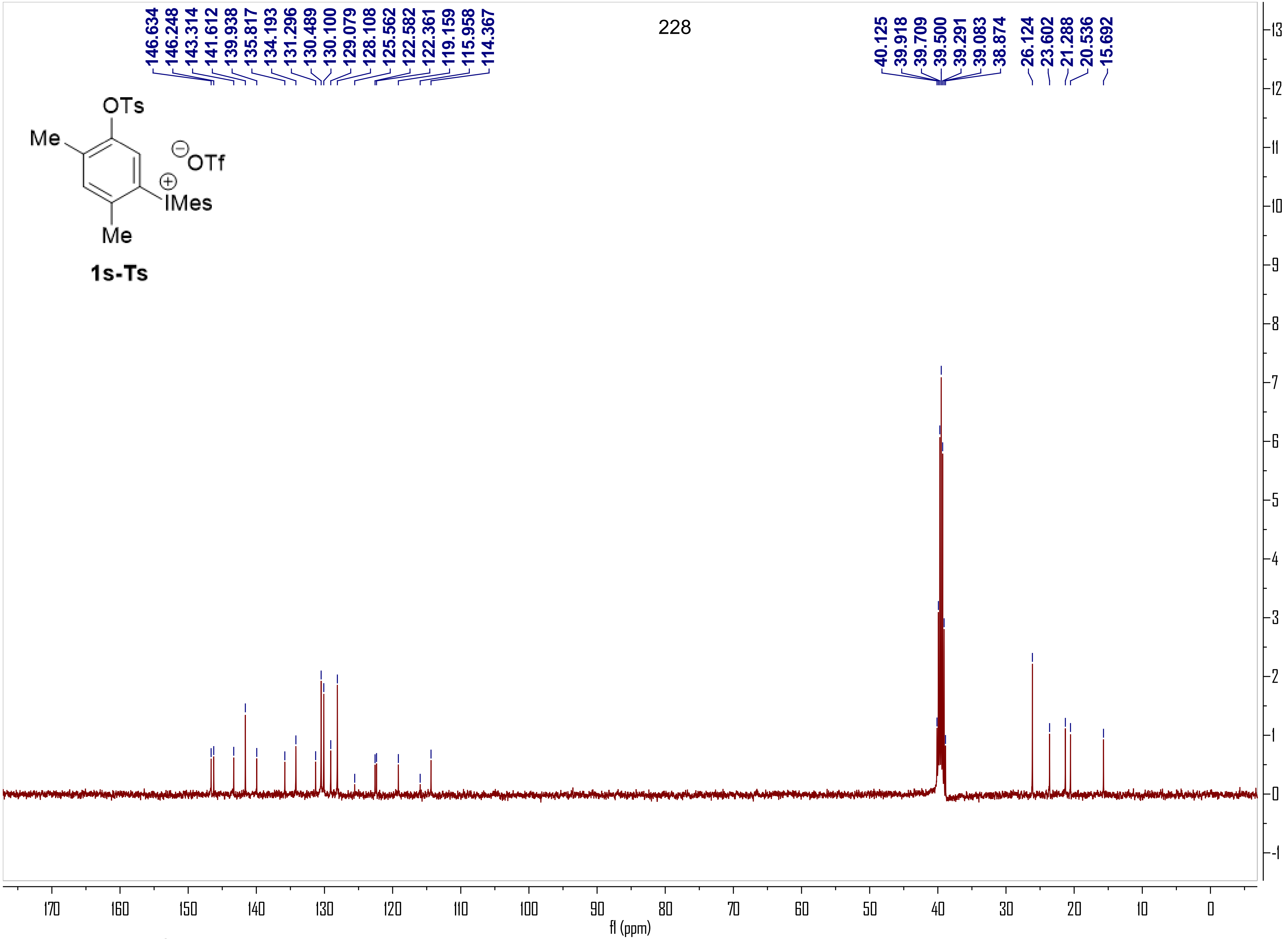

Supplementary Fig 151. ^{13}**C NMR spectrum (100 MHz, DMSO-*d*6, r.t.) of 1s-Ts.**

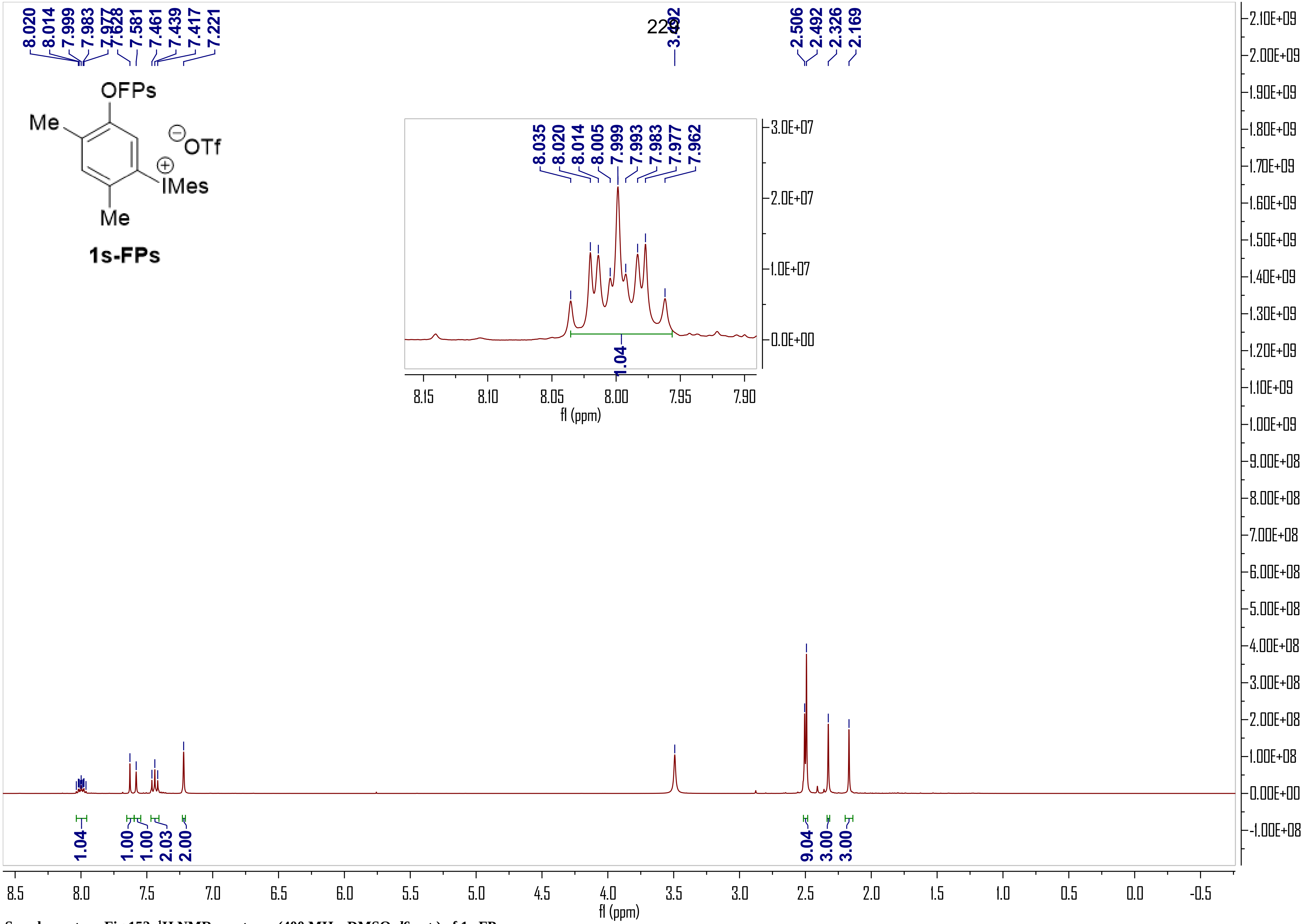

Supplementary Fig 152. ^{1}H NMR spectrum (400 MHz, DMSO-*d*6, r.t.) of 1s-FPs.

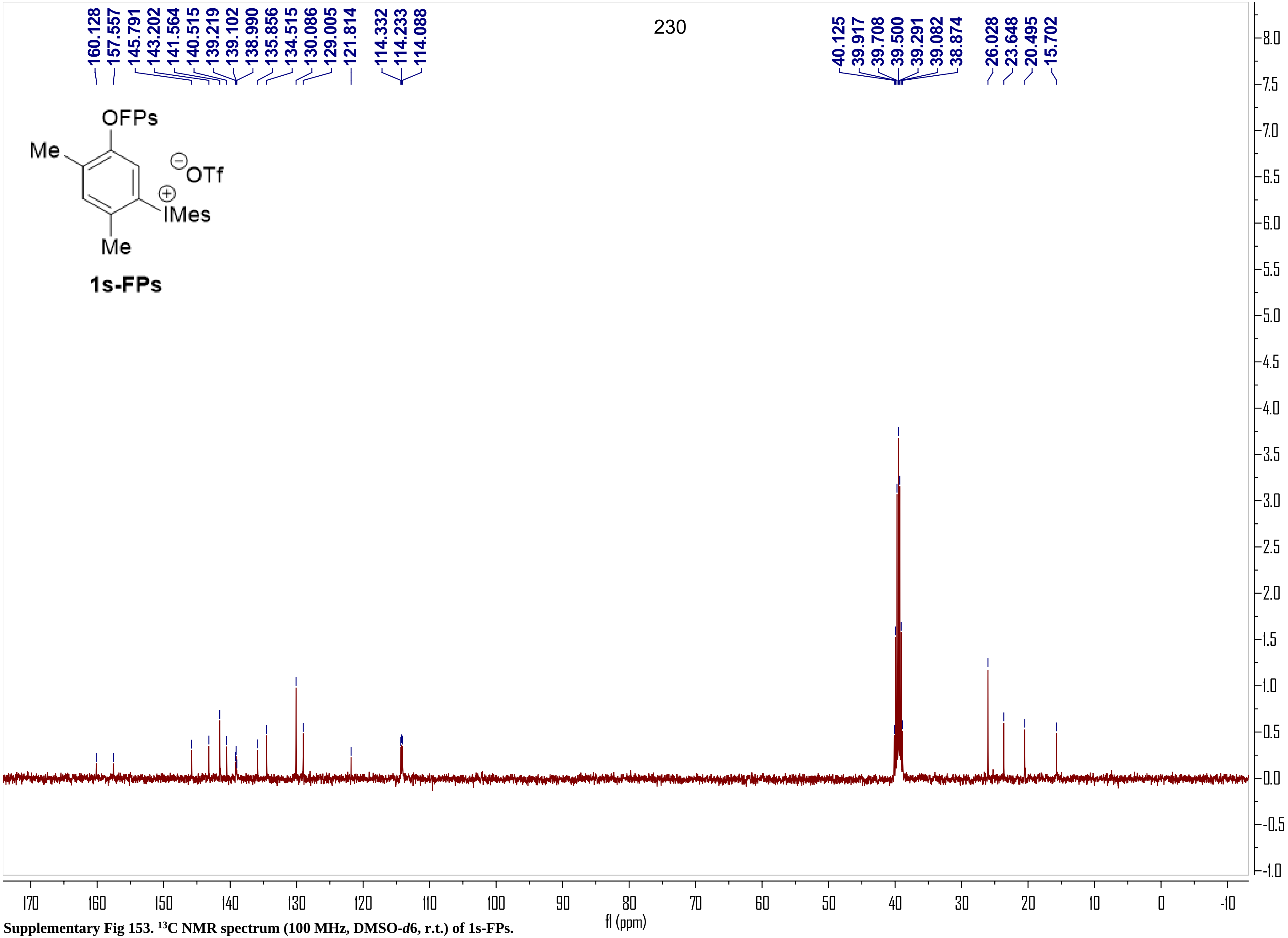

Supplementary Fig 153. **^{13}C NMR spectrum (100 MHz, DMSO-*d*6, r.t.) of 1s-FPs.**

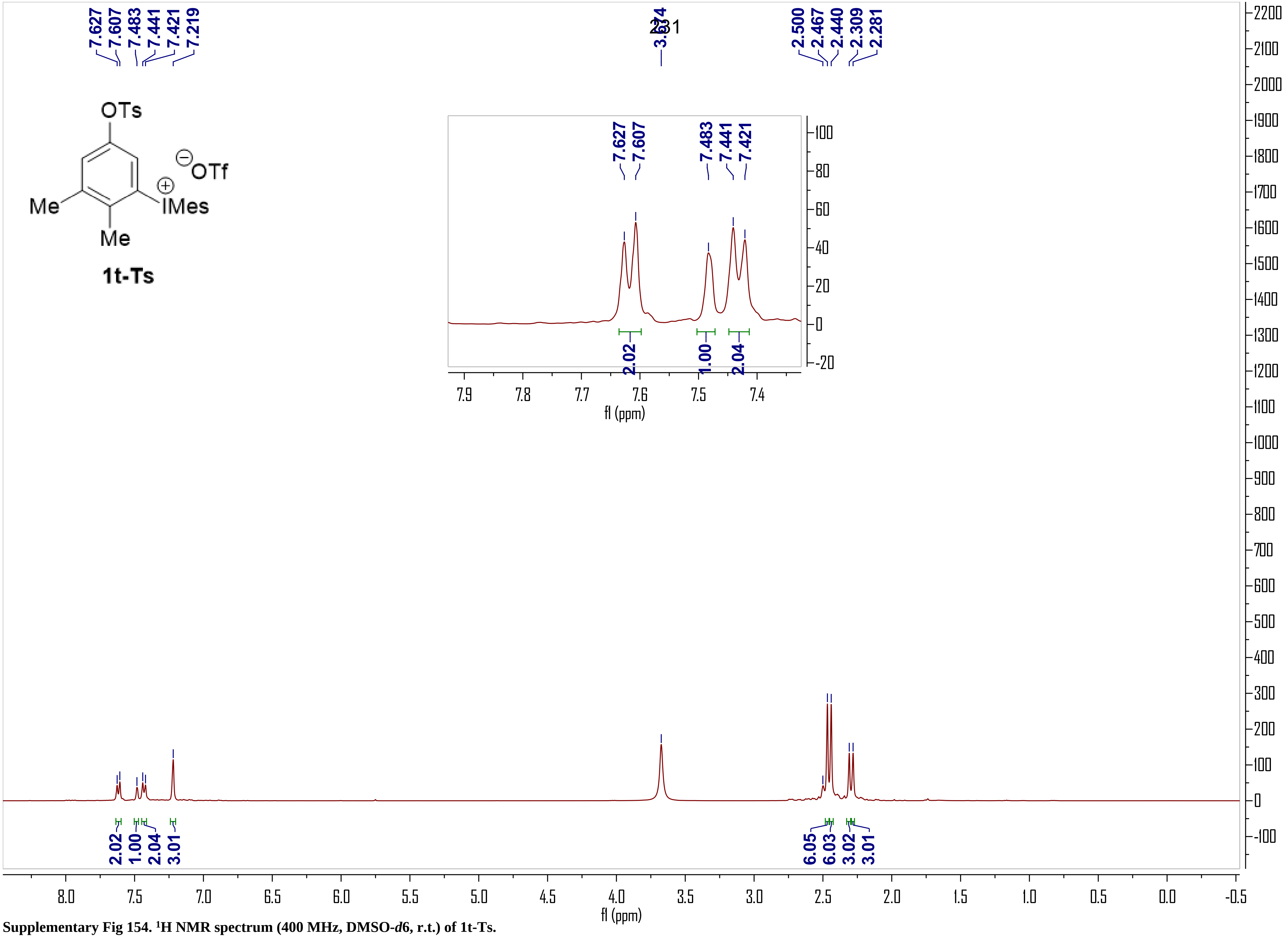

Supplementary Fig 154. **^{1}H NMR spectrum (400 MHz, DMSO-*d*6, r.t.) of 1t-Ts.**

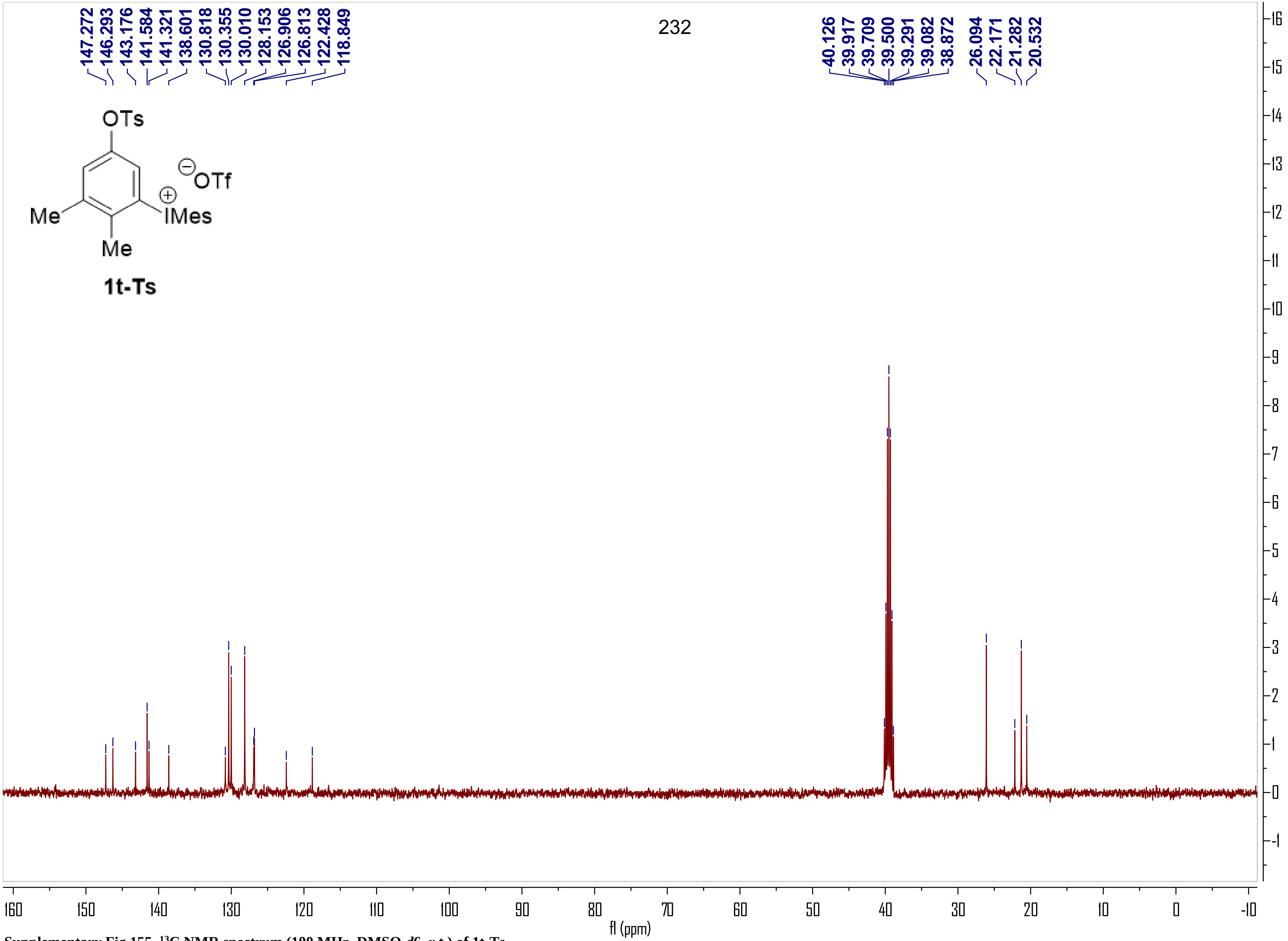

Supplementary Fig 155. **^{13}C NMR spectrum (100 MHz, DMSO-*d*6, r.t.) of 1t-Ts.**

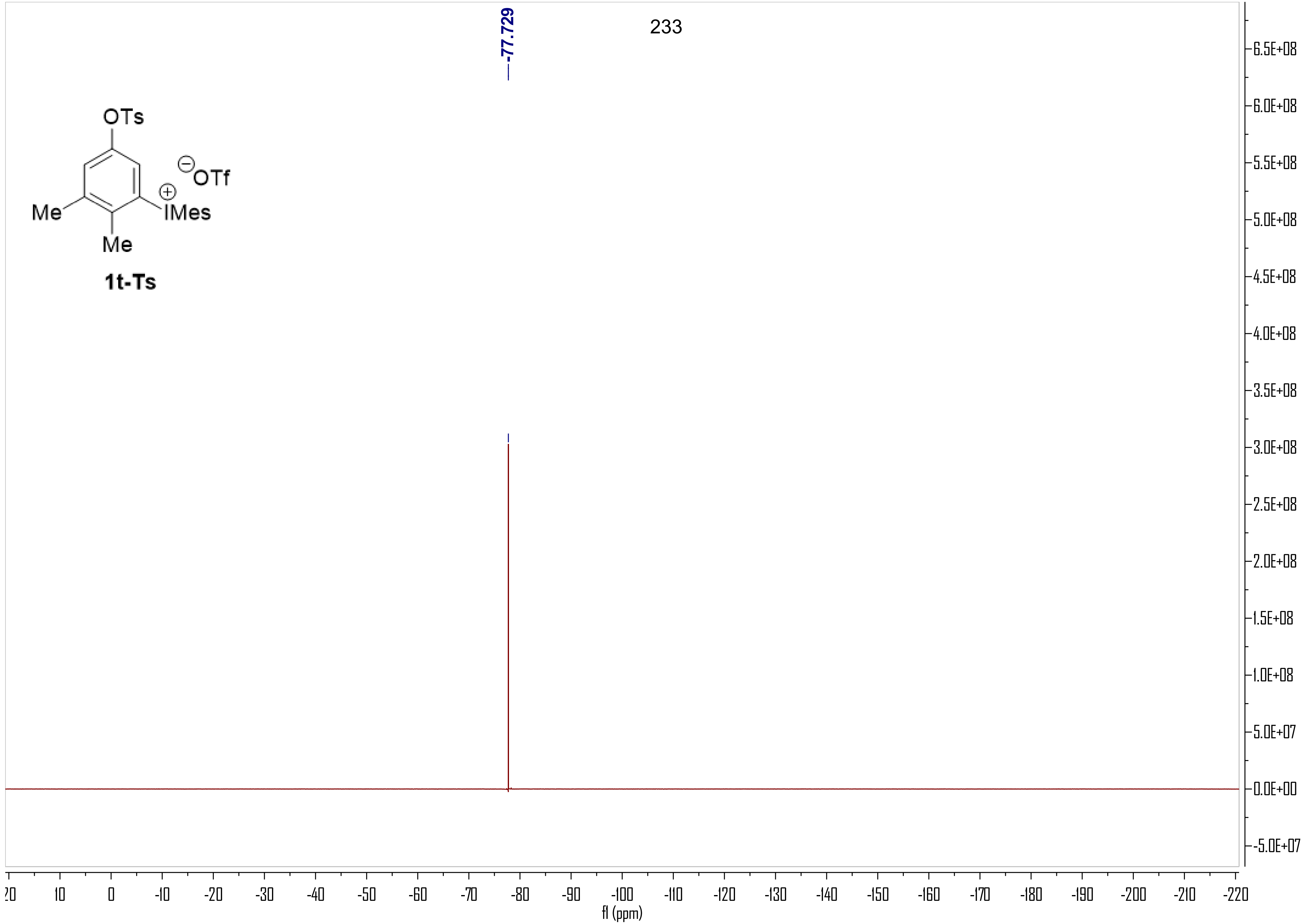

Supplementary Fig 156. ^{19}F NMR spectrum (400 MHz, DMSO-*d*6, r.t.) of 1t-Ts.

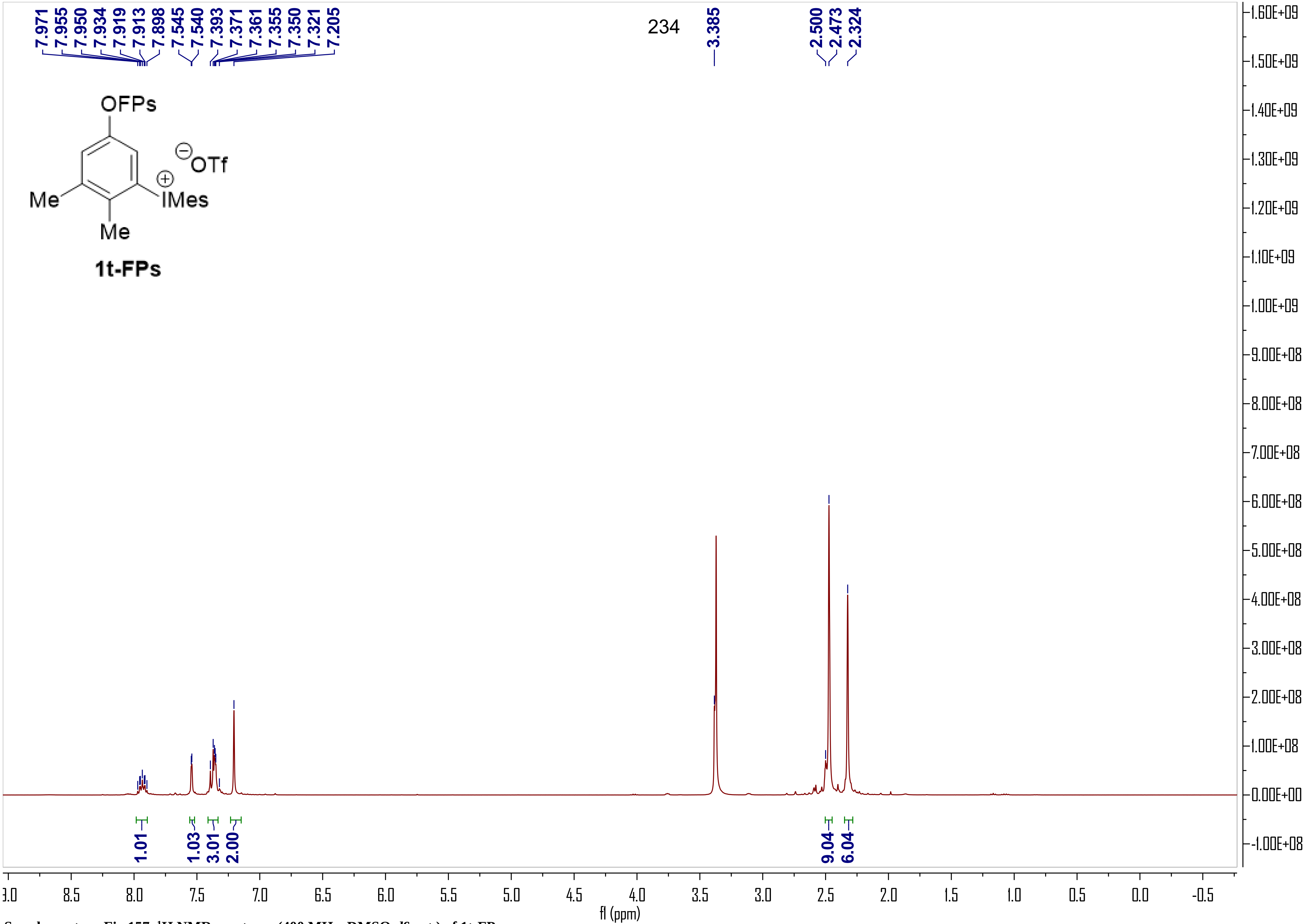

Supplementary Fig 157. **^{1}H NMR spectrum (400 MHz, DMSO-*d*6, r.t.) of 1t-FPs.**

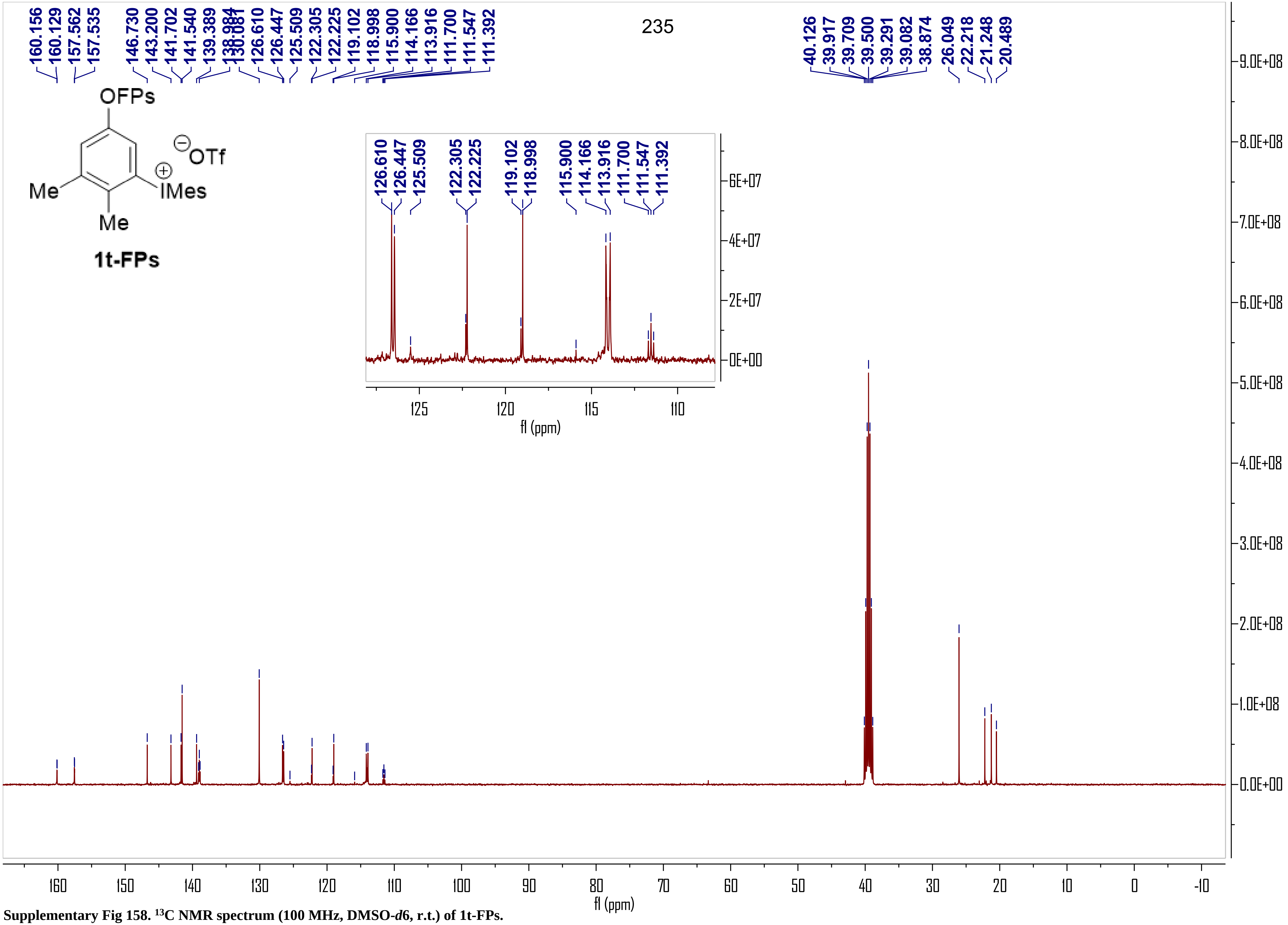

Supplementary Fig 158. ^{13}**C NMR spectrum (100 MHz, DMSO-*d*6, r.t.) of 1t-FPs.**

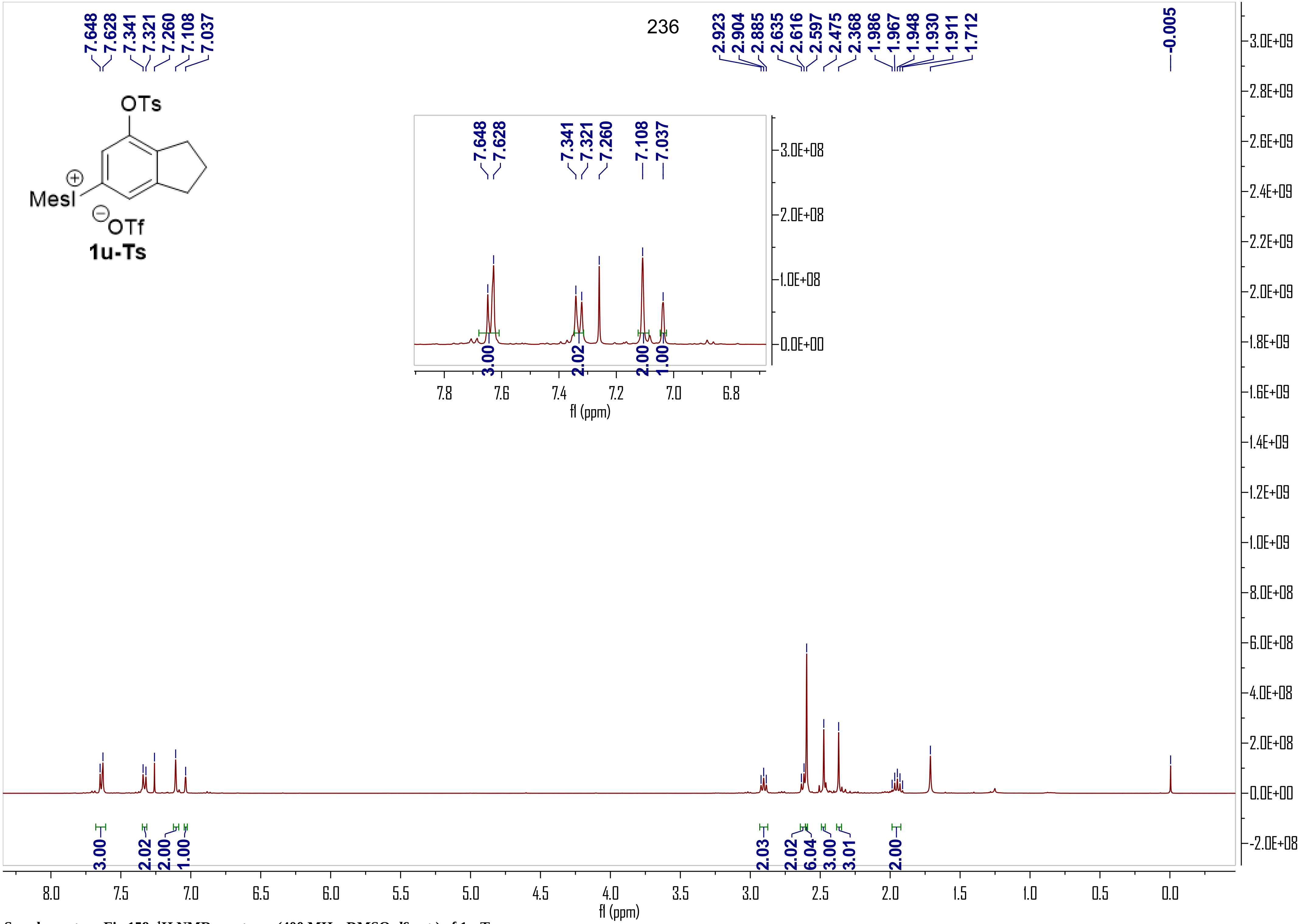

Supplementary Fig 159. **^{1}H NMR spectrum (400 MHz, DMSO-*d6*, r.t.) of 1u-Ts.**

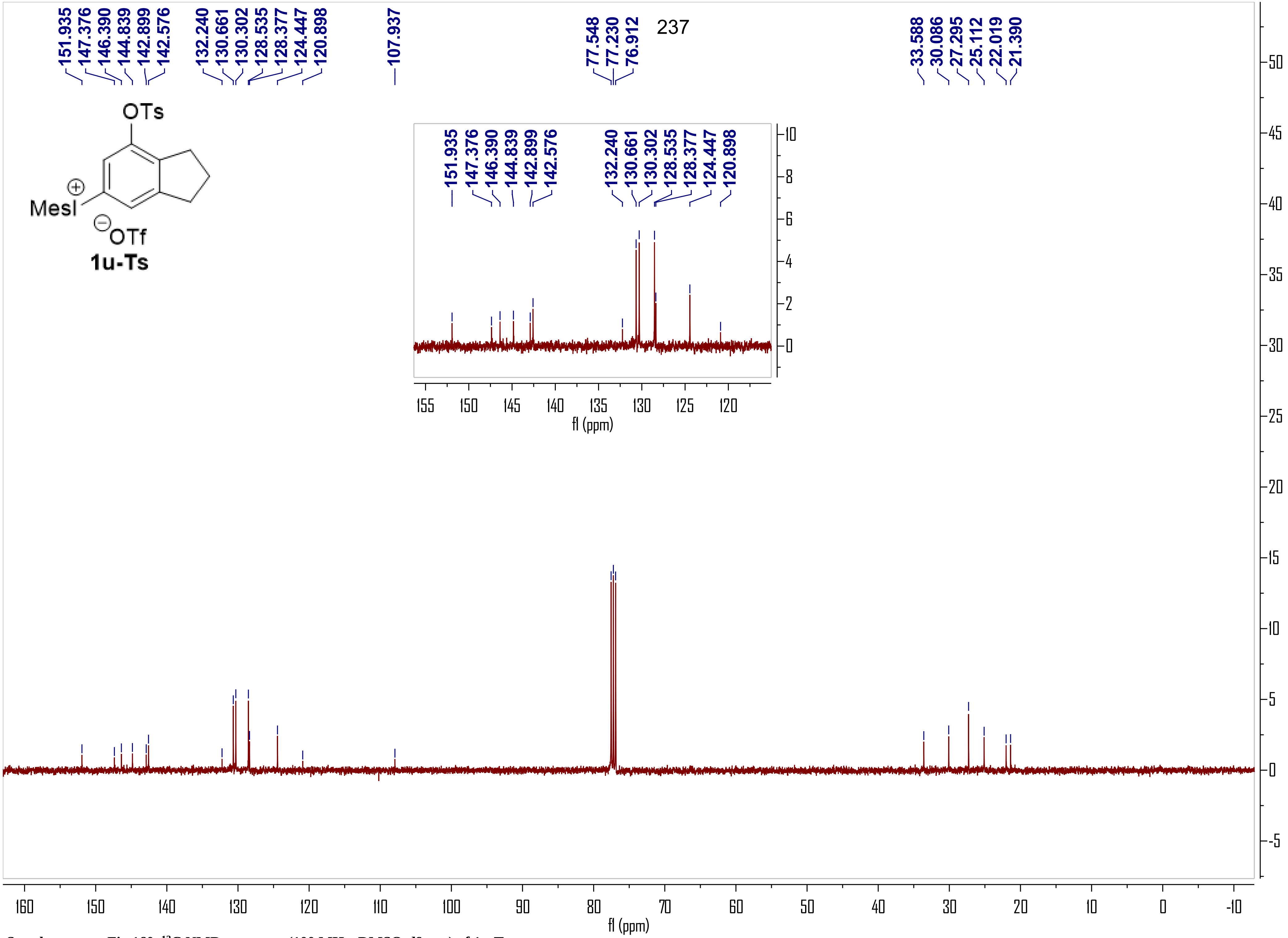

Supplementary Fig 160. **^{13}C NMR spectrum (100 MHz, DMSO-*d*6, r.t.) of 1u-Ts.**

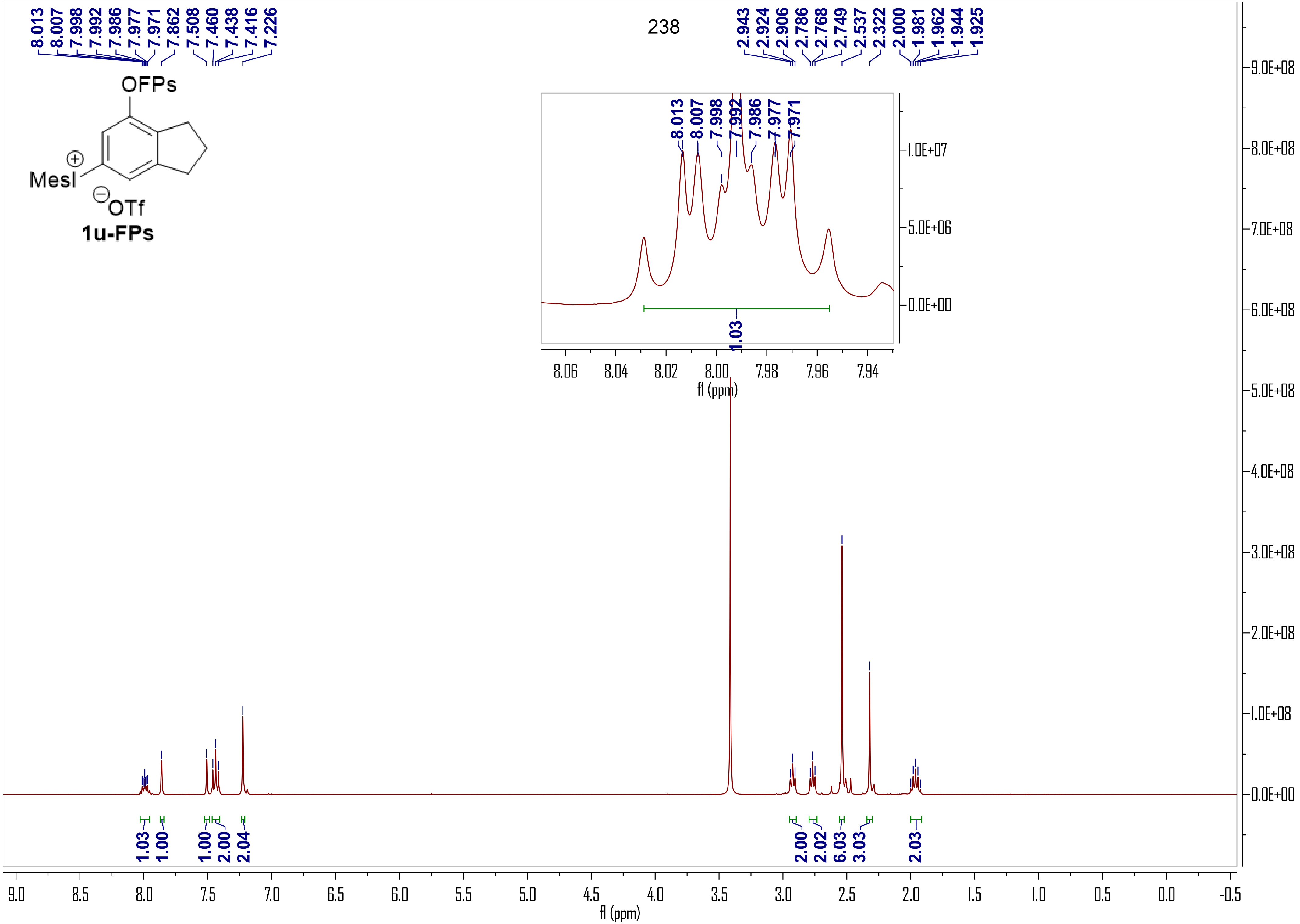

Supplementary Fig 161. **¹H NMR spectrum (400 MHz, DMSO-*d*6, r.t.) of 1u-FPs.**

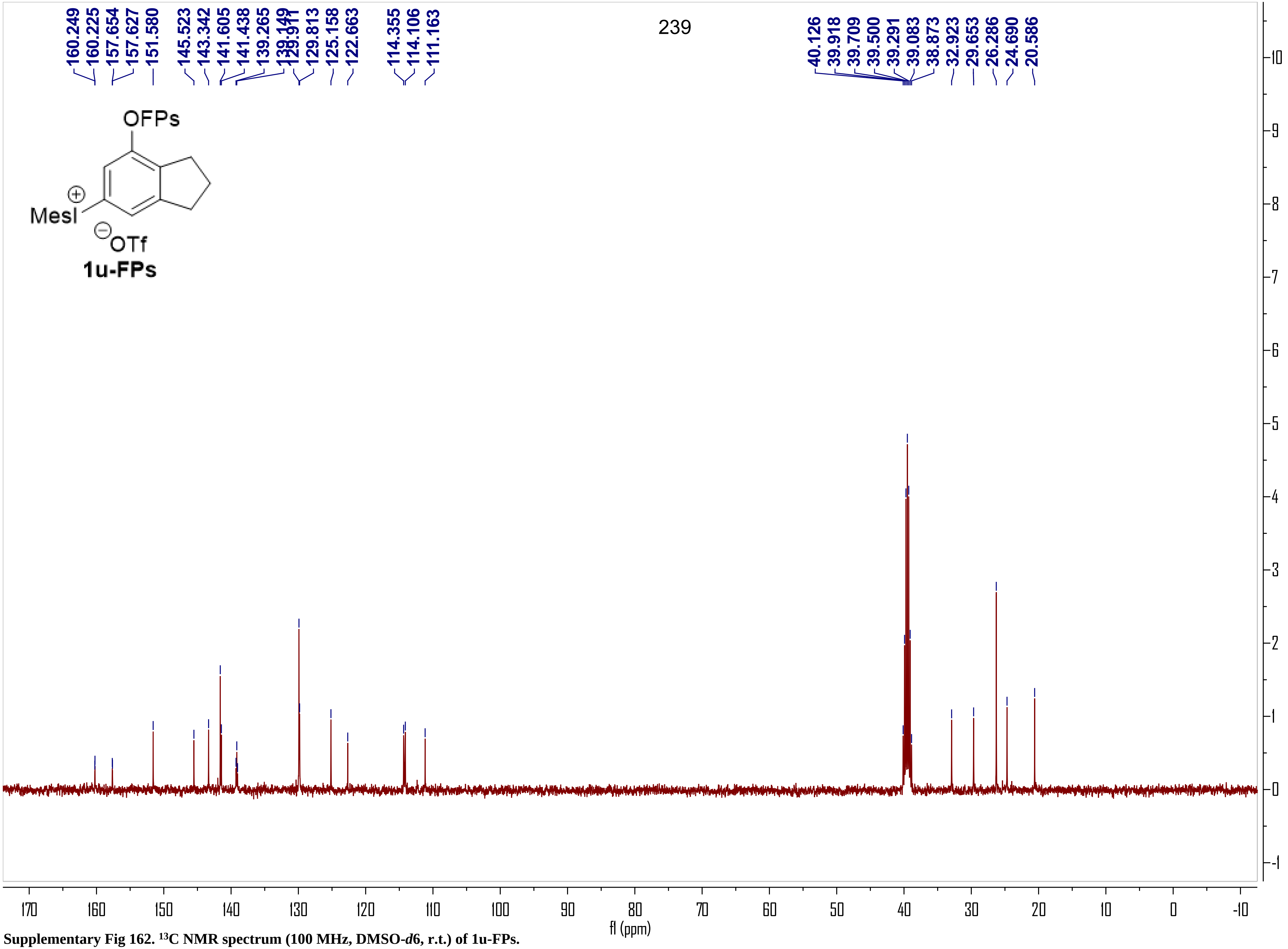

Supplementary Fig 162. **^{13}C NMR spectrum (100 MHz, DMSO-*d*6, r.t.) of 1u-FPs.**

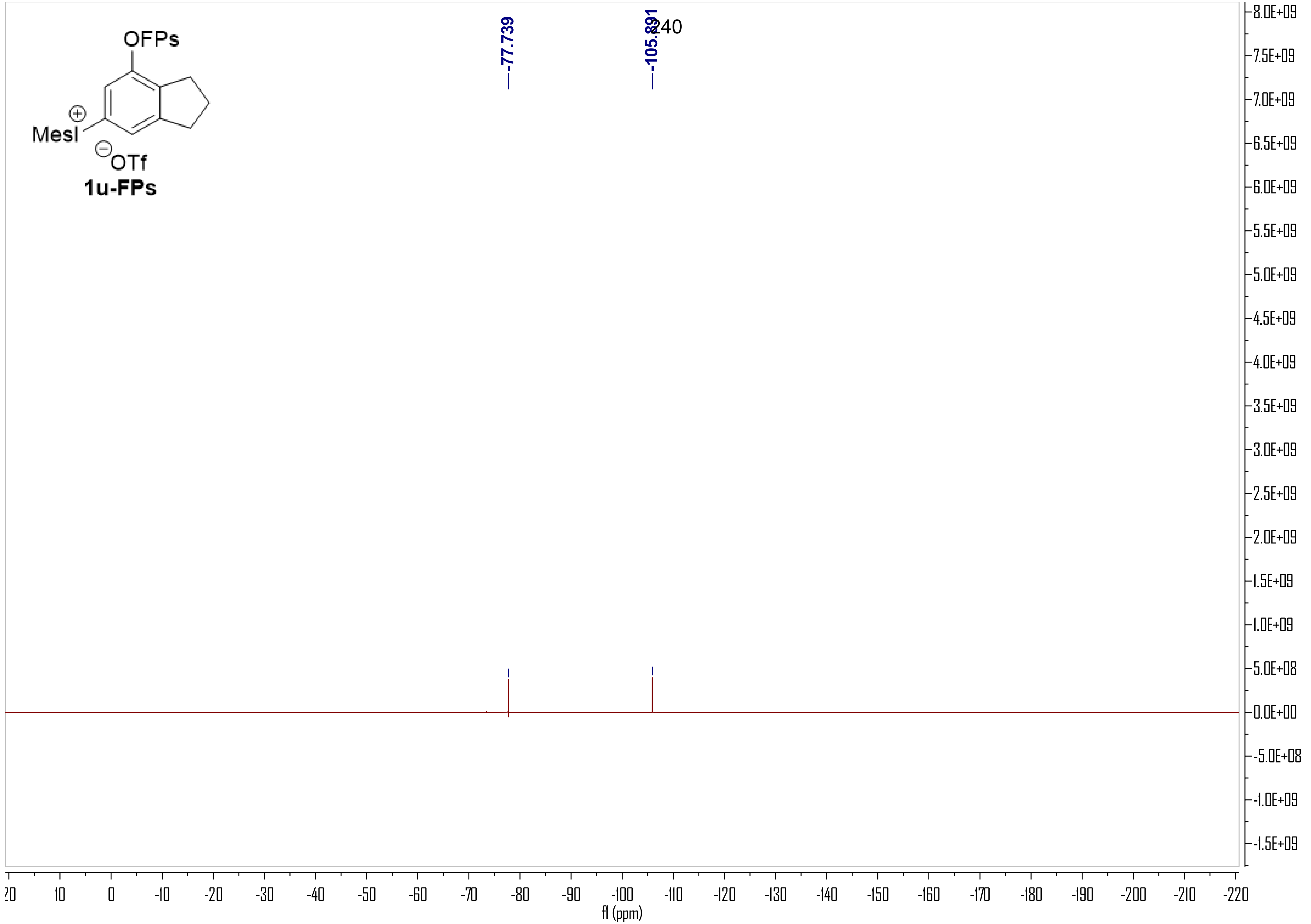

Supplementary Fig 163. ^{19}F NMR spectrum (400 MHz, DMSO-*d*6, r.t.) of 1u-FPs.

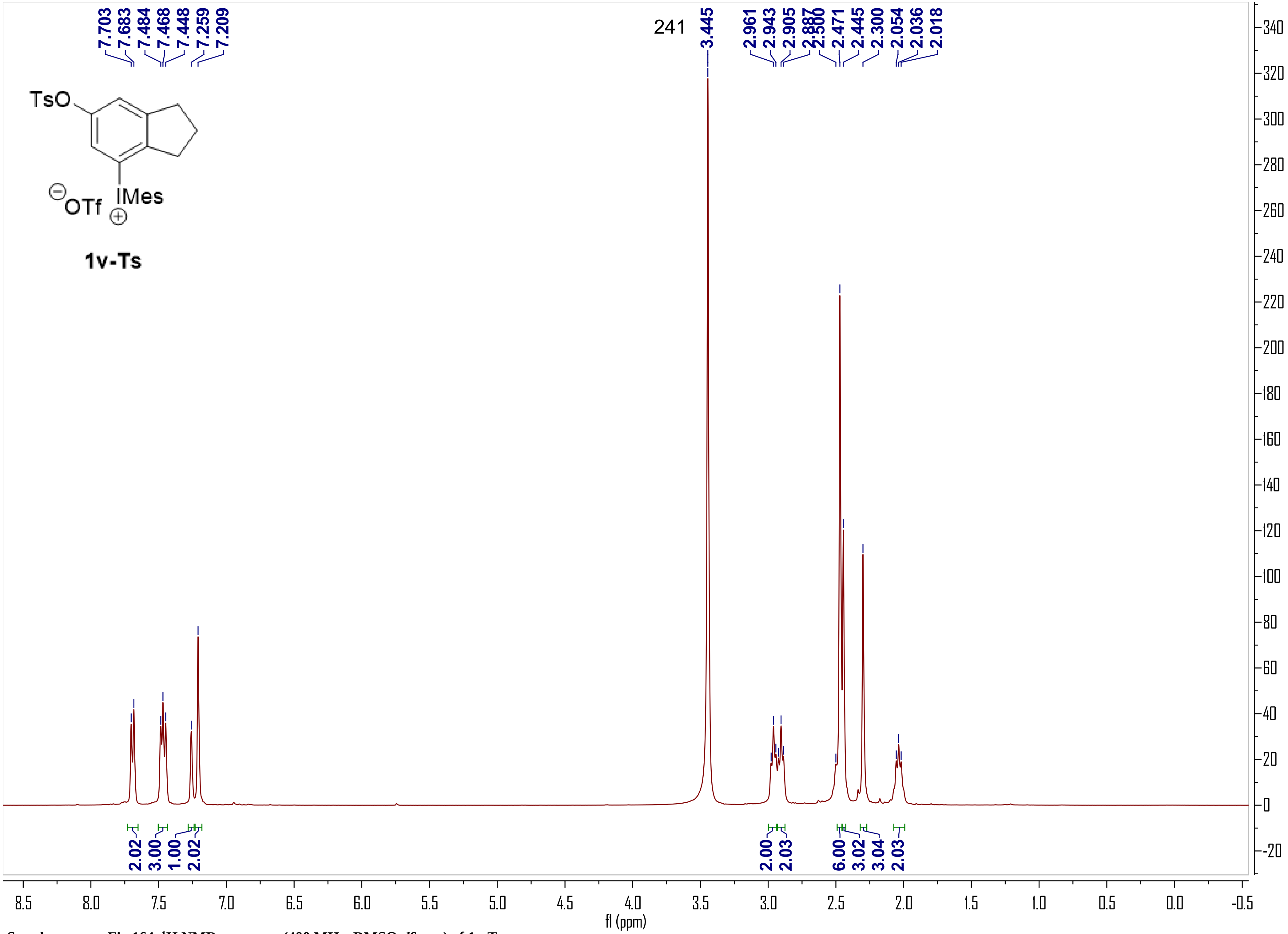

Supplementary Fig 164. **^{1}H NMR spectrum (400 MHz, DMSO-*d*6, r.t.) of 1v-Ts.**

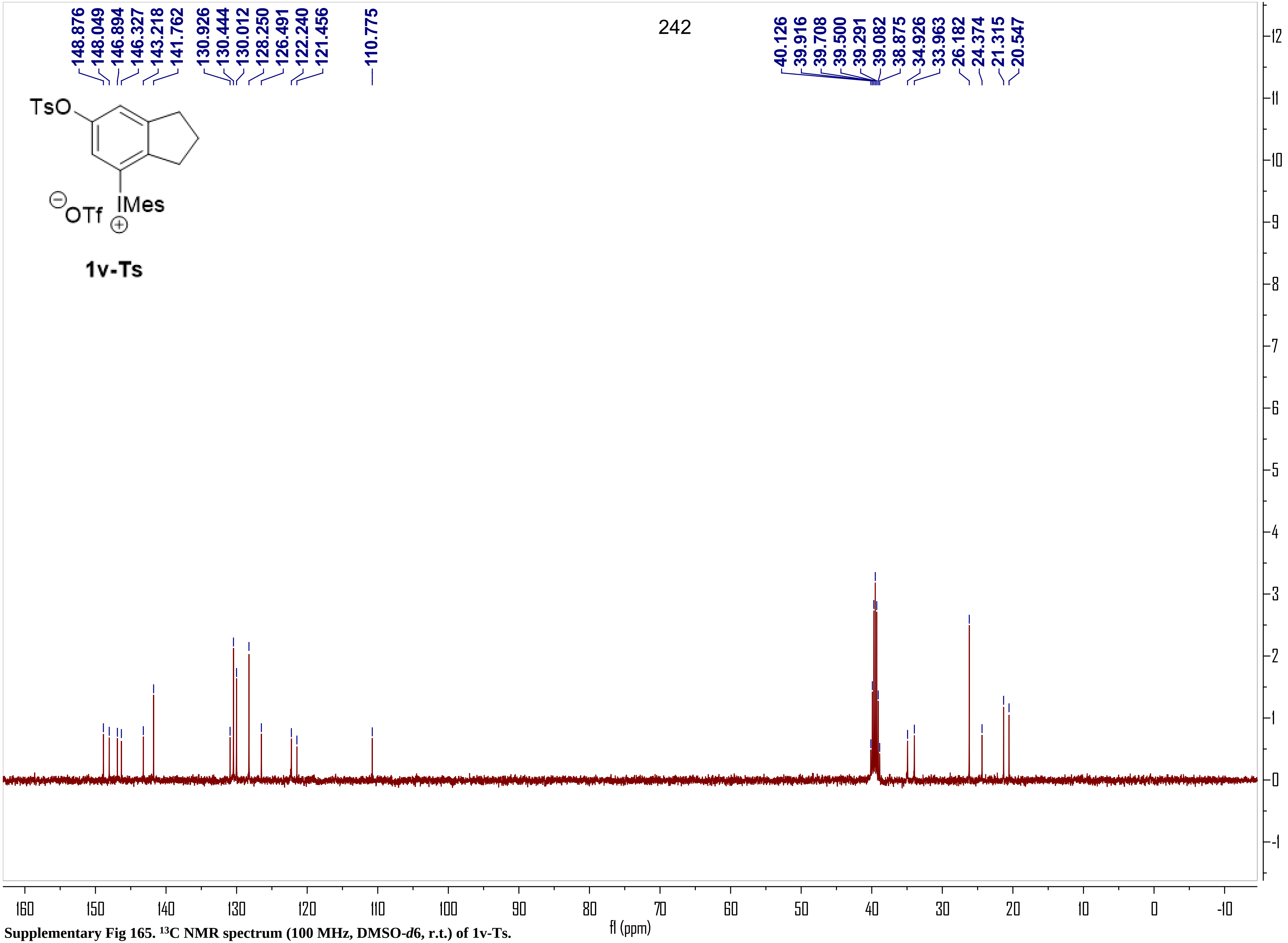

Supplementary Fig 165. ^{13}C NMR spectrum (100 MHz, DMSO-*d*6, r.t.) of 1v-Ts.

1v-Ts

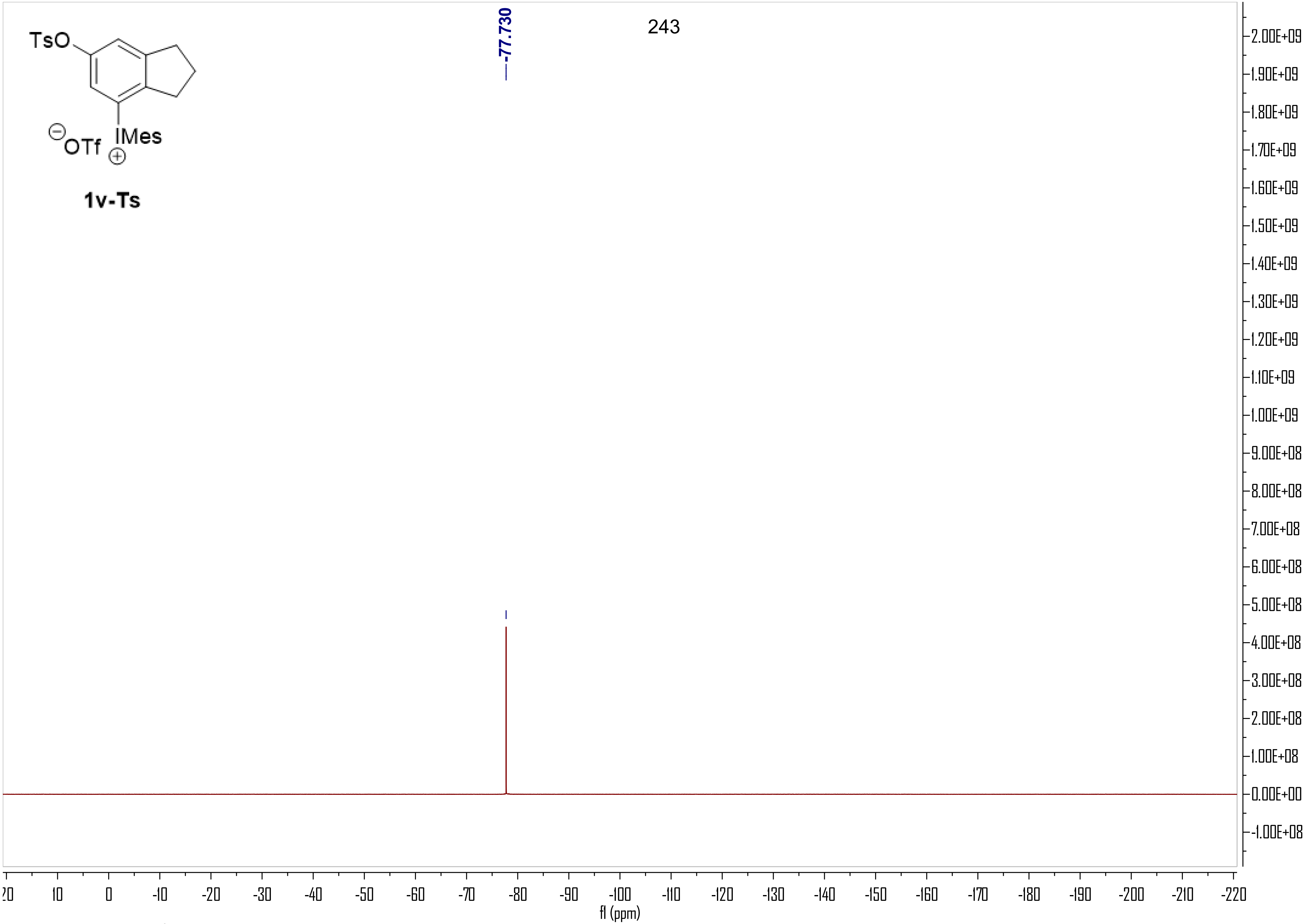

Supplementary Fig 166. ^{19}F **NMR spectrum (400 MHz, DMSO-*d*6, r.t.) of 1v-Ts.**

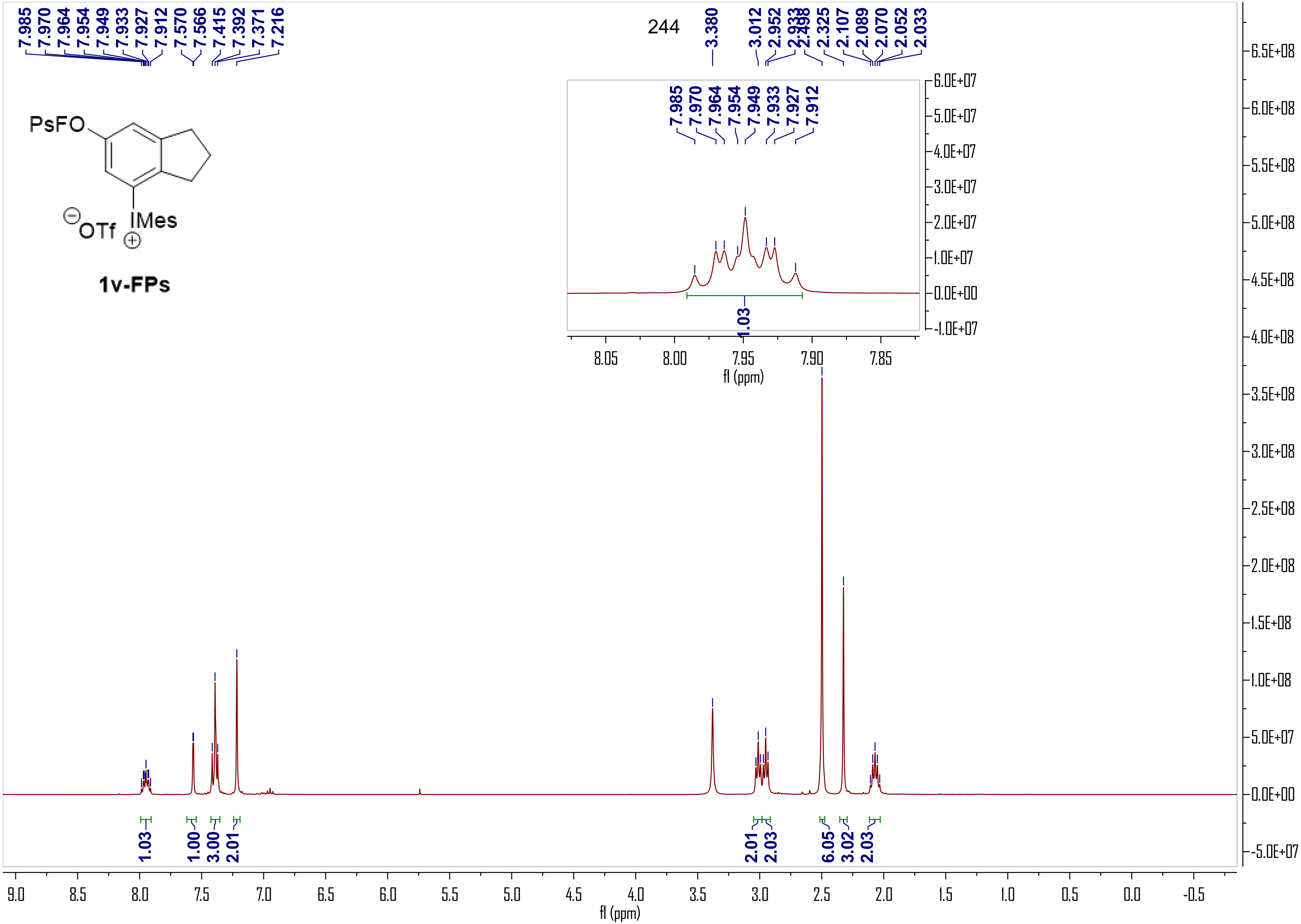

Supplementary Fig 167. **^{1}H NMR spectrum (400 MHz, DMSO-*d*6, r.t.) of 1v-FPs.**

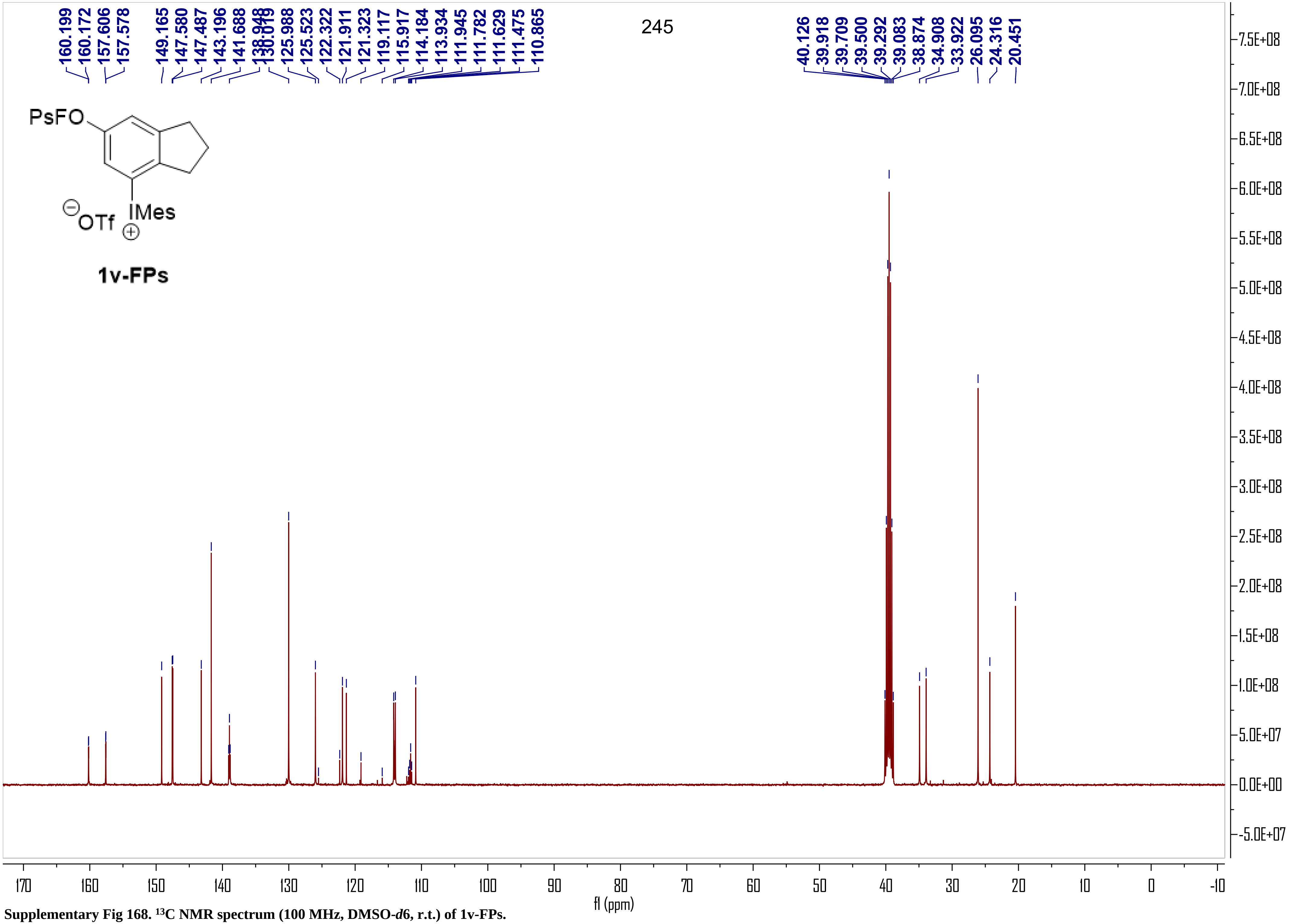

Supplementary Fig 168. ^{13}C NMR spectrum (100 MHz, DMSO-*d*6, r.t.) of 1v-FPs.

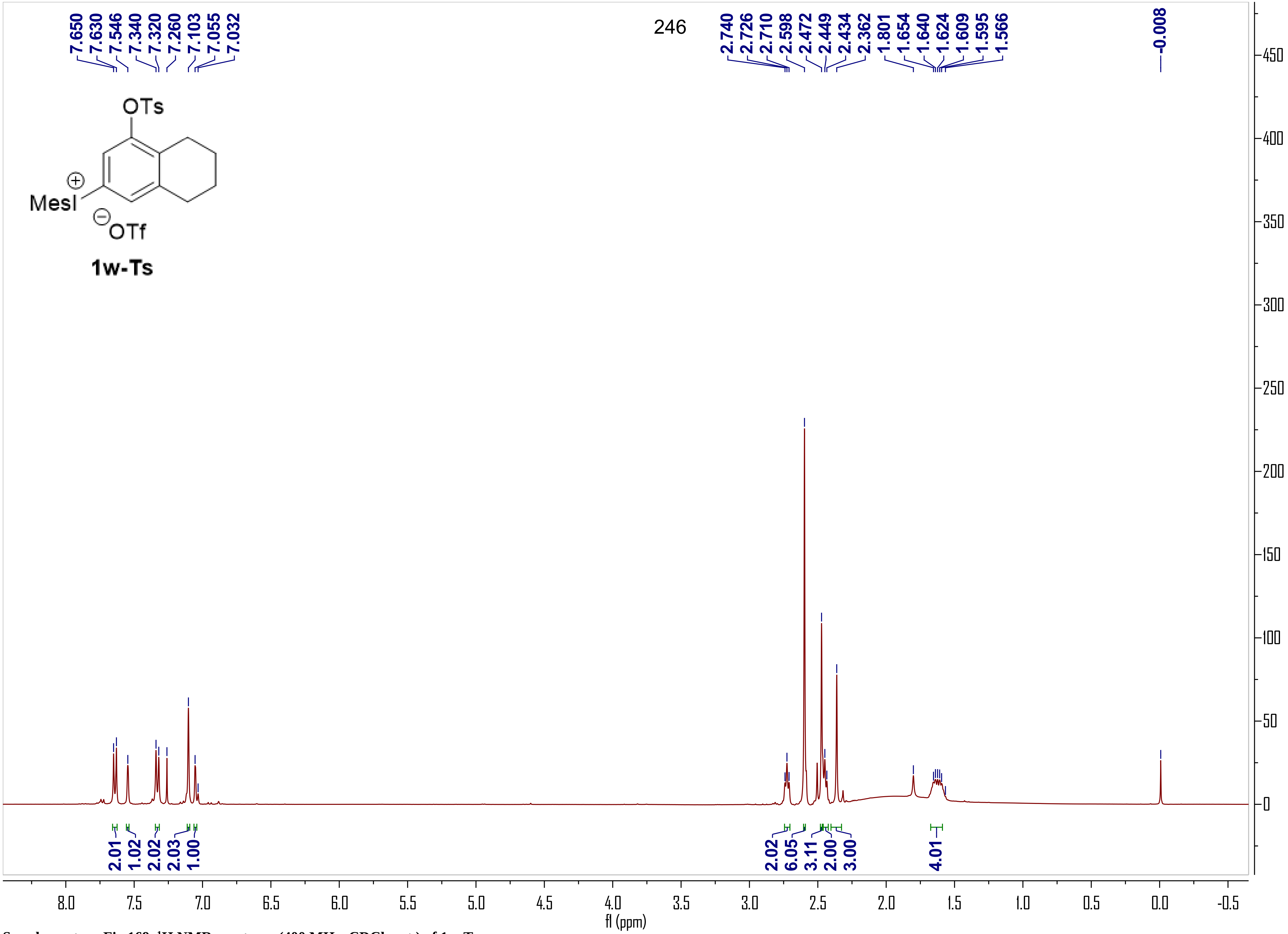

Supplementary Fig 169. ^{1}H NMR spectrum (400 MHz, $CDCl_3$, r.t.) of 1w-Ts.

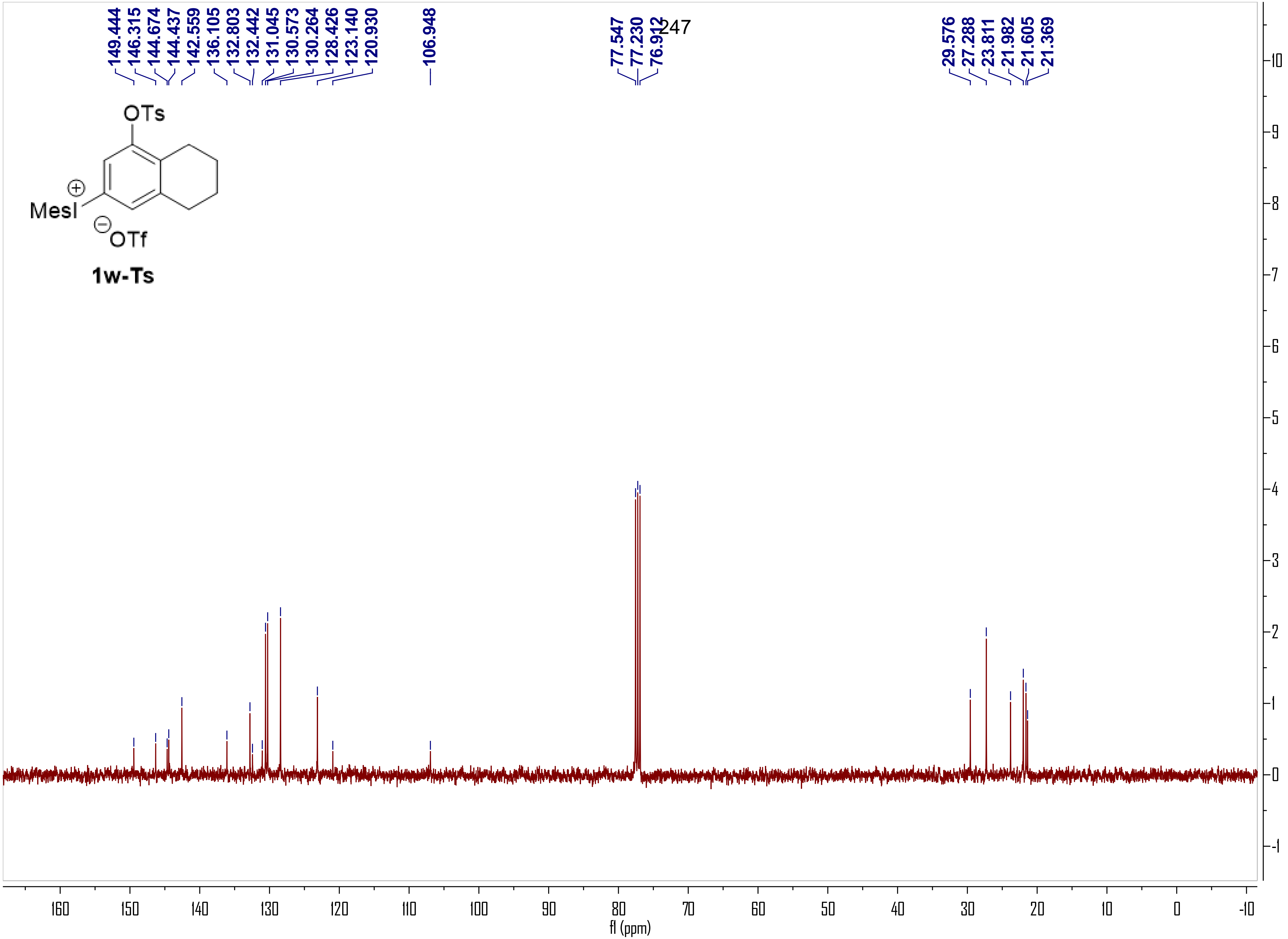

Supplementary Fig 170. ^{13}C **NMR spectrum (100 MHz,** $CDCl_3$**, r.t.) of 1w-Ts.**

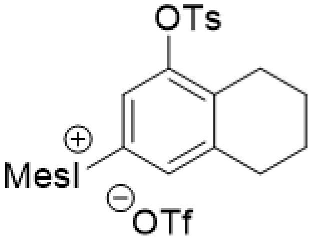

1w-Ts

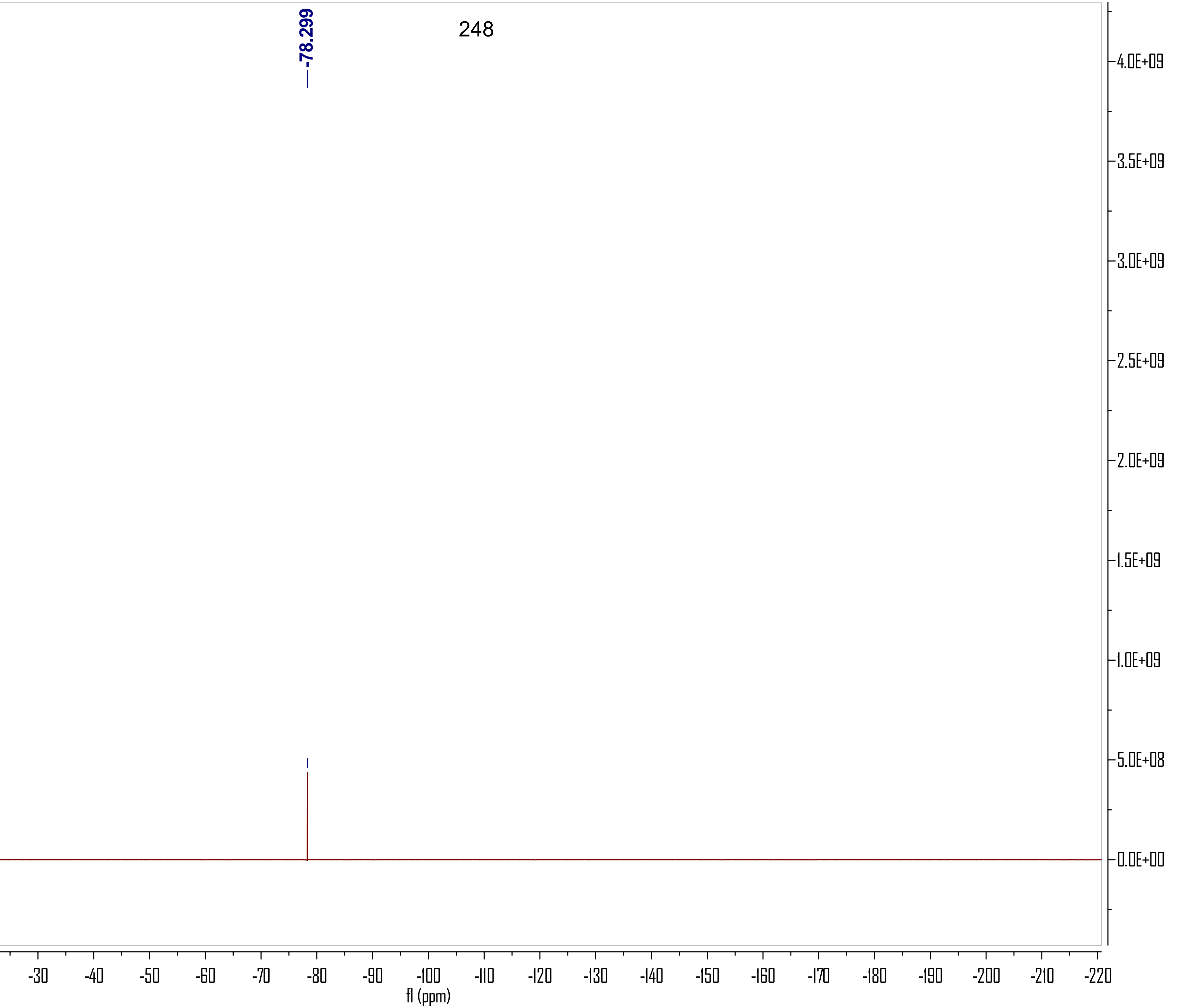

Supplementary Fig 171. ^{19}F NMR spectrum (400 MHz, $CDCl_3$, r.t.) of 1w-Ts.

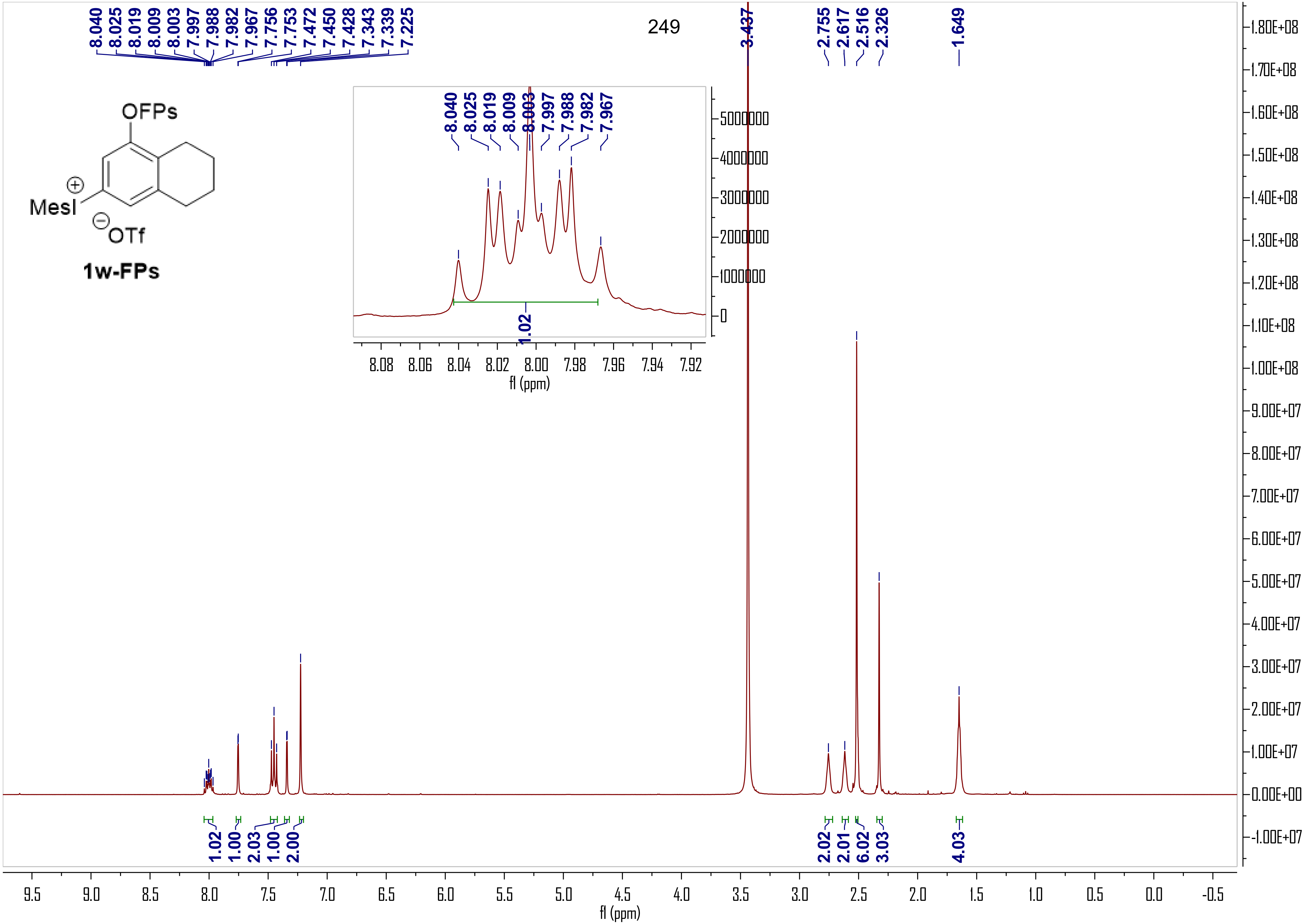

Supplementary Fig 172. **¹H NMR spectrum (400 MHz, DMSO-*d*6, r.t.) of 1w-FPs.**

250

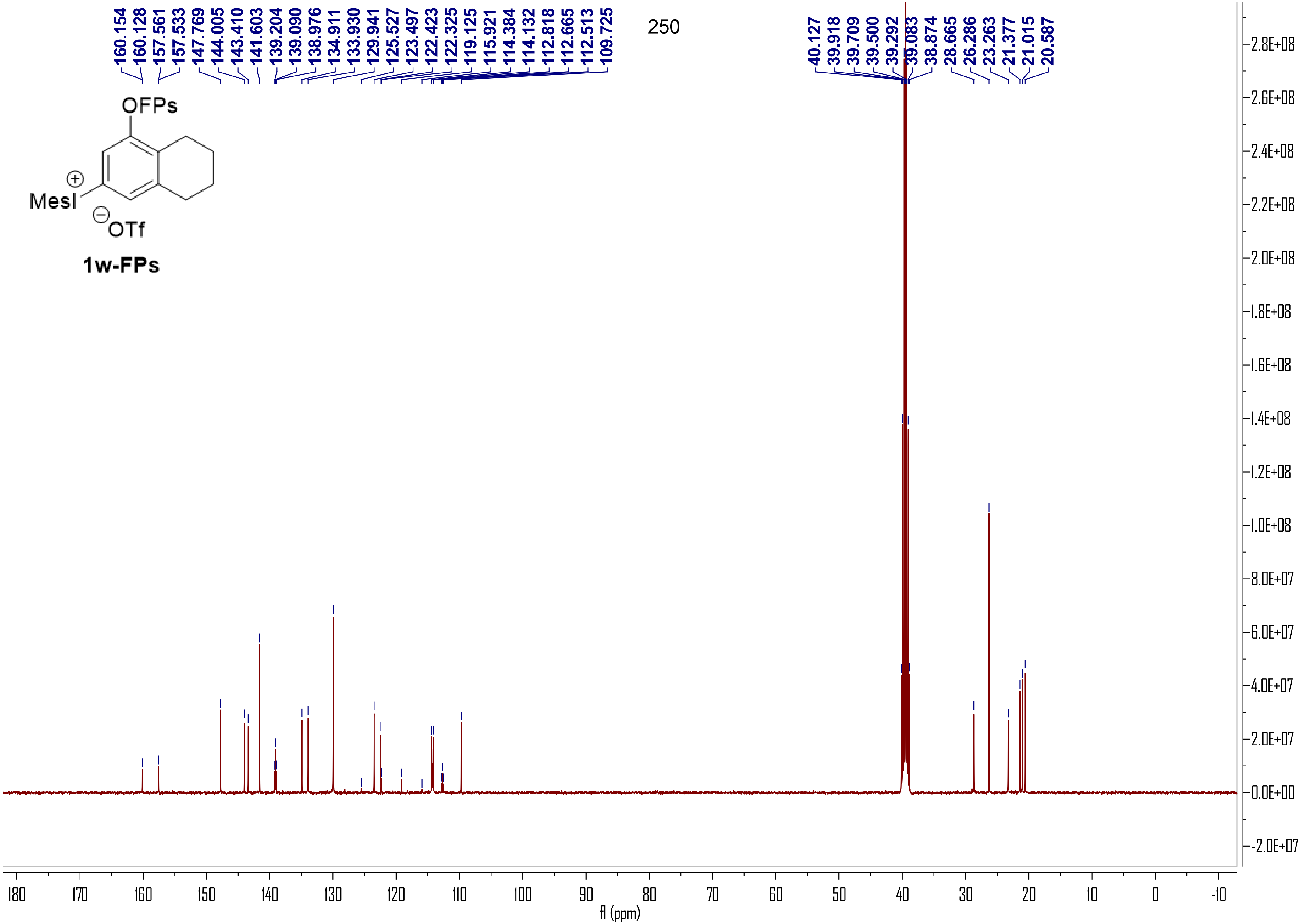

Supplementary Fig 173. **^{13}C NMR spectrum (100 MHz, DMSO-*d*6, r.t.) of 1v-FPs.**

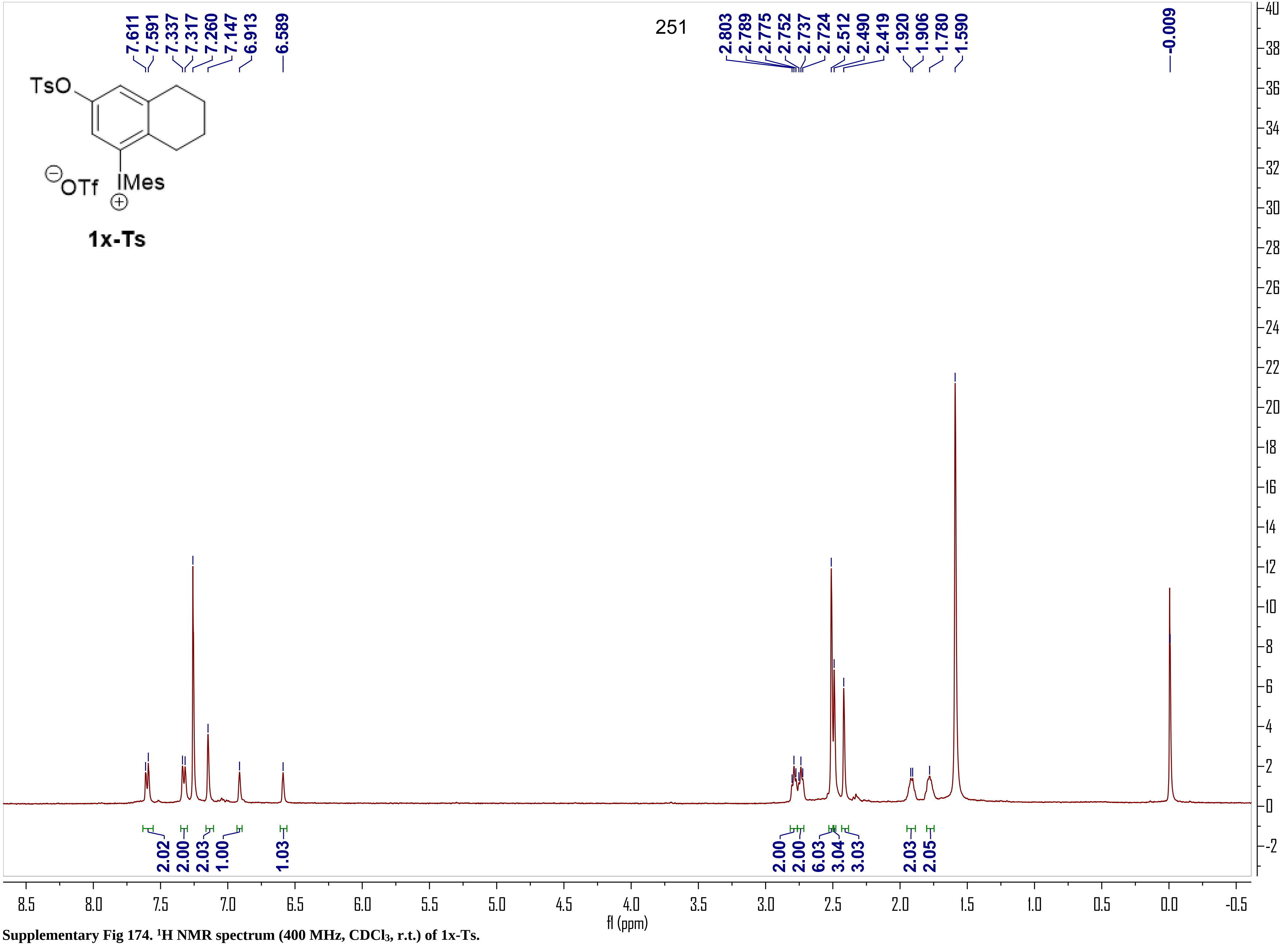

Supplementary Fig 174. ^{1}H **NMR spectrum (400 MHz,** $CDCl_3$**, r.t.) of 1x-Ts.**

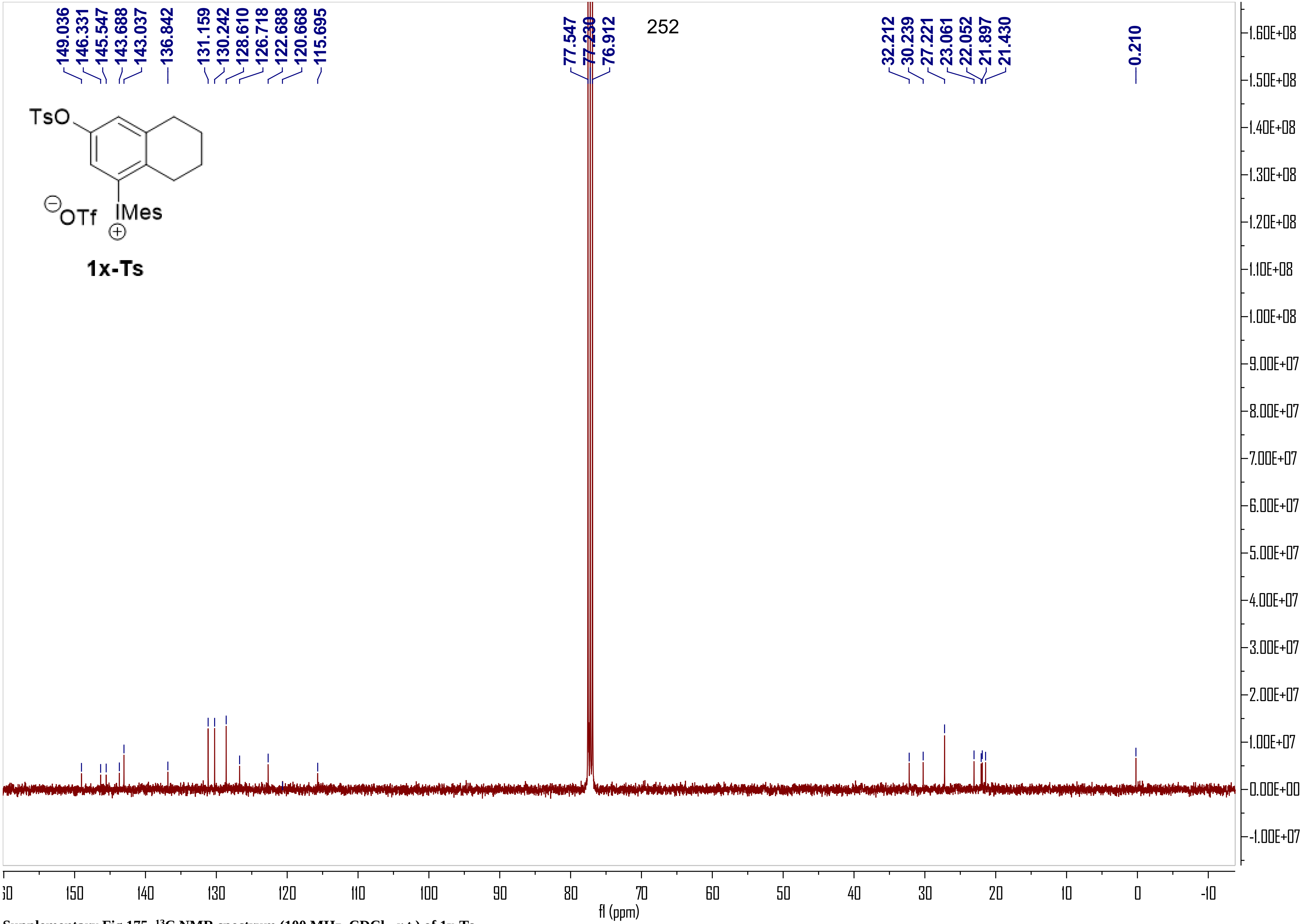

Supplementary Fig 175. ^{13}C **NMR spectrum (100 MHz,** $CDCl_3$**, r.t.) of 1x-Ts.**

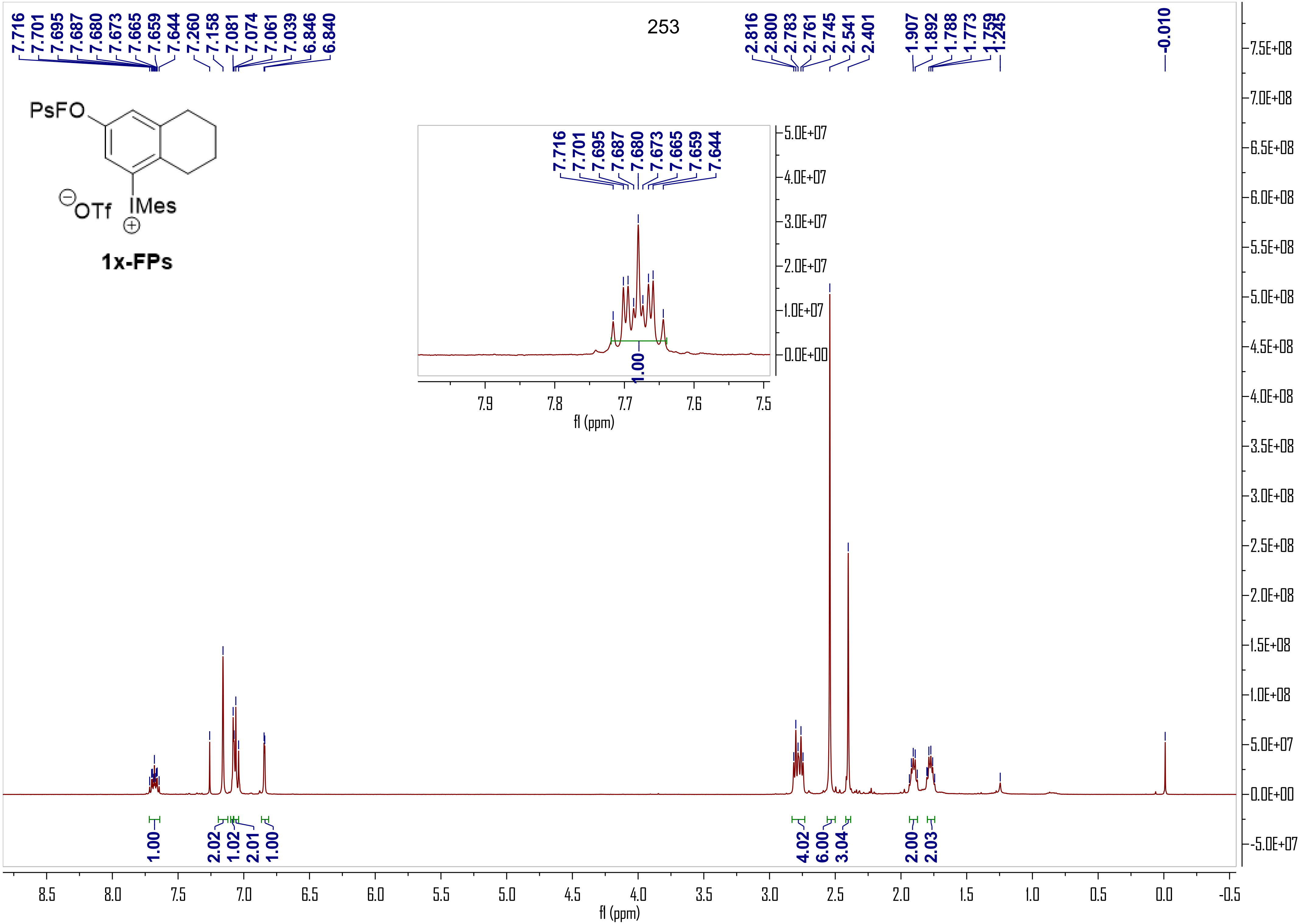

Supplementary Fig 176. ^{1}H NMR spectrum (400 MHz, $CDCl_3$, r.t.) of 1x-FPs.

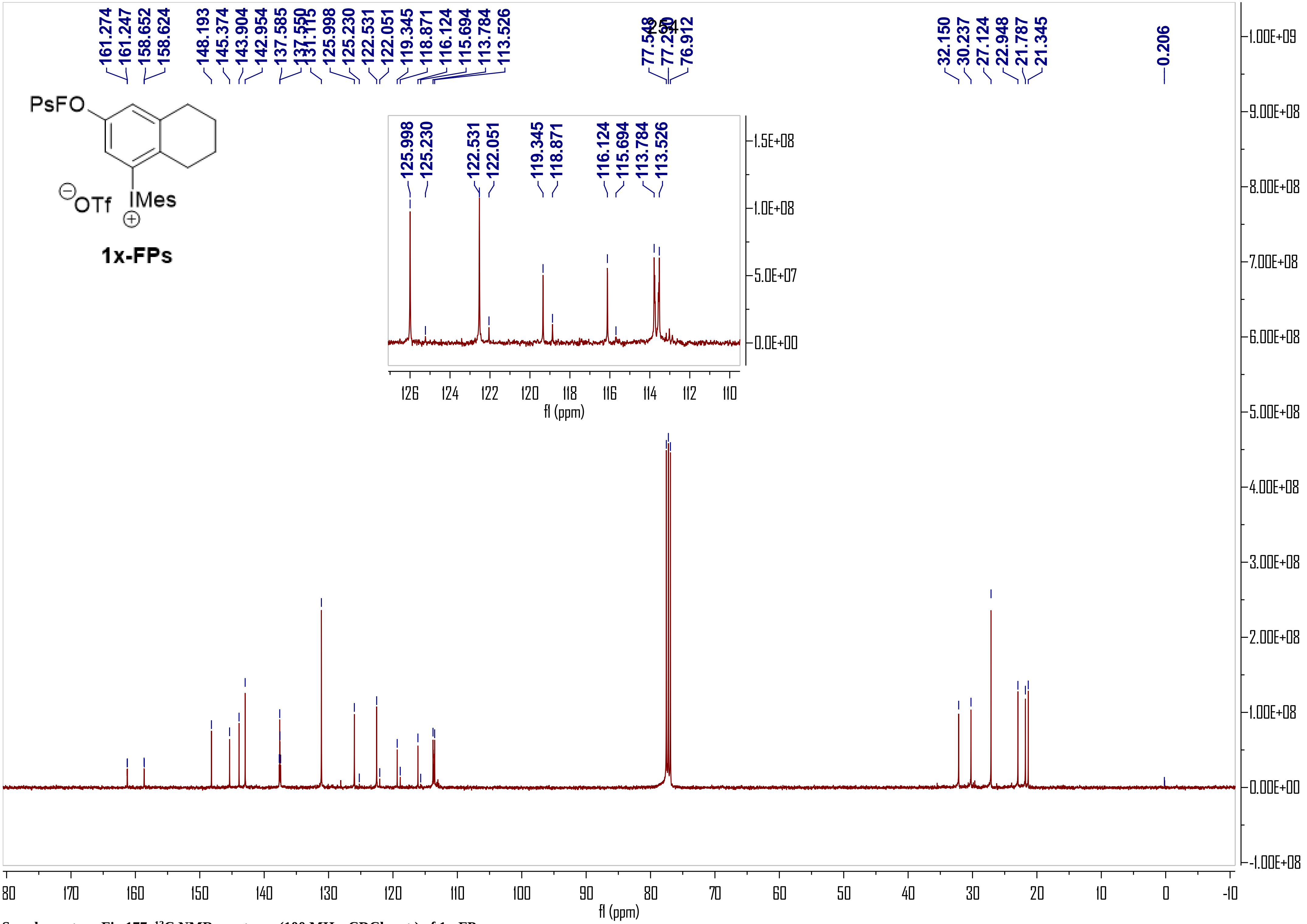

Supplementary Fig 177. ^{13}C NMR spectrum (100 MHz, $CDCl_3$, r.t.) of 1x-FPs.

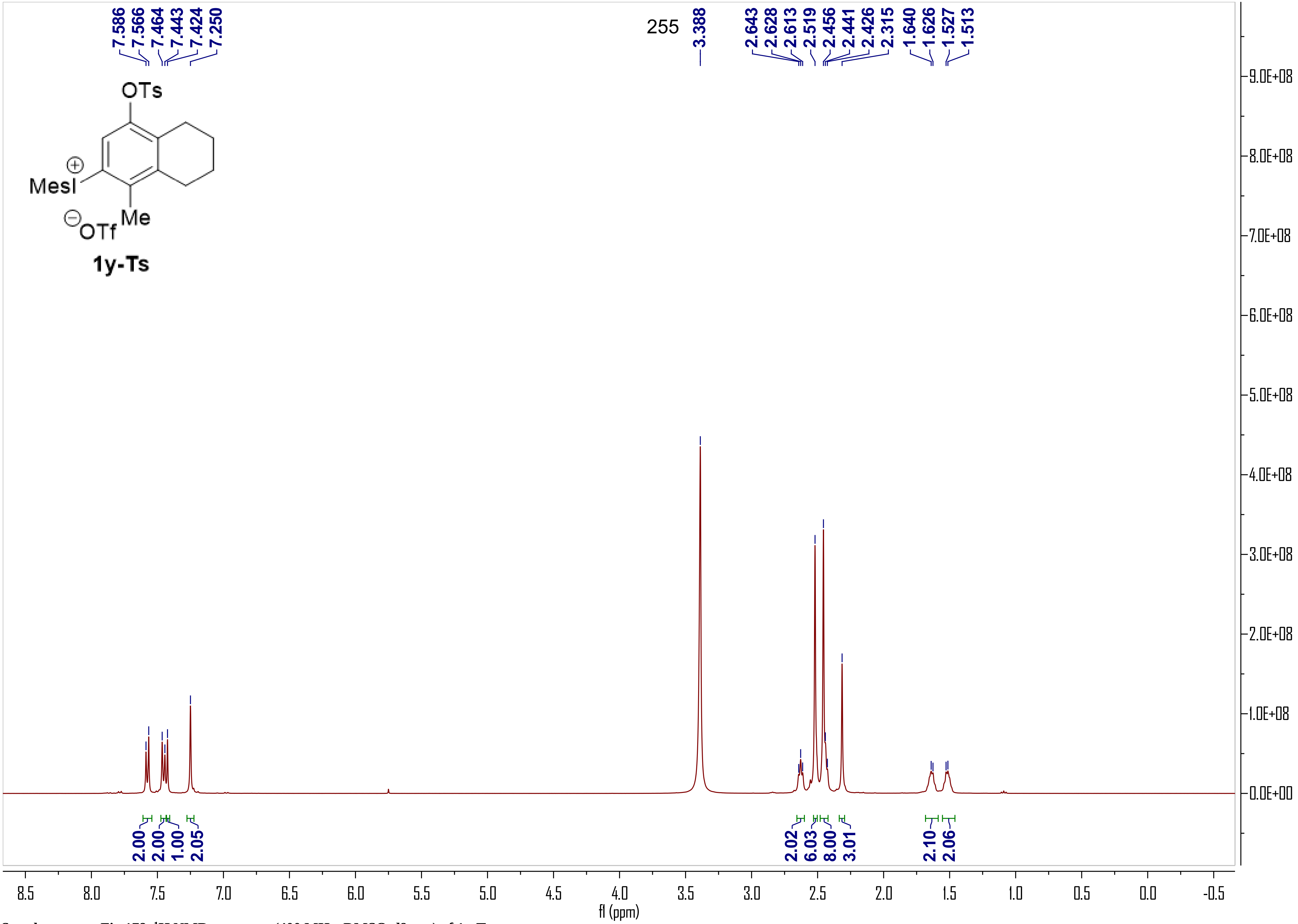

Supplementary Fig 178. ^{1}**H NMR spectrum (400 MHz, DMSO-*d*6, r.t.) of 1y-Ts.**

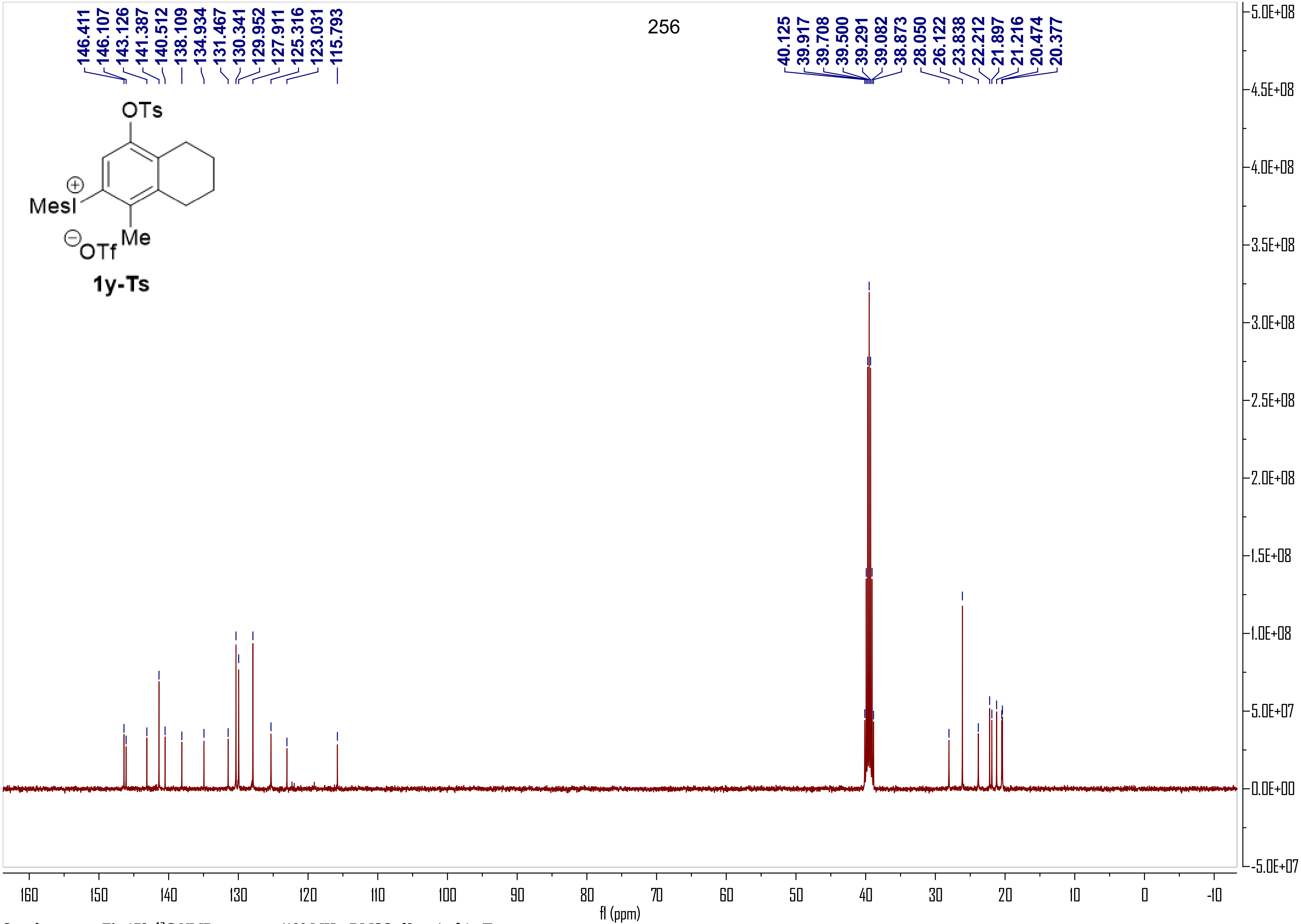

Supplementary Fig 179. ^{13}**C NMR spectrum (100 MHz, DMSO-*d*6, r.t.) of 1y-Ts.**

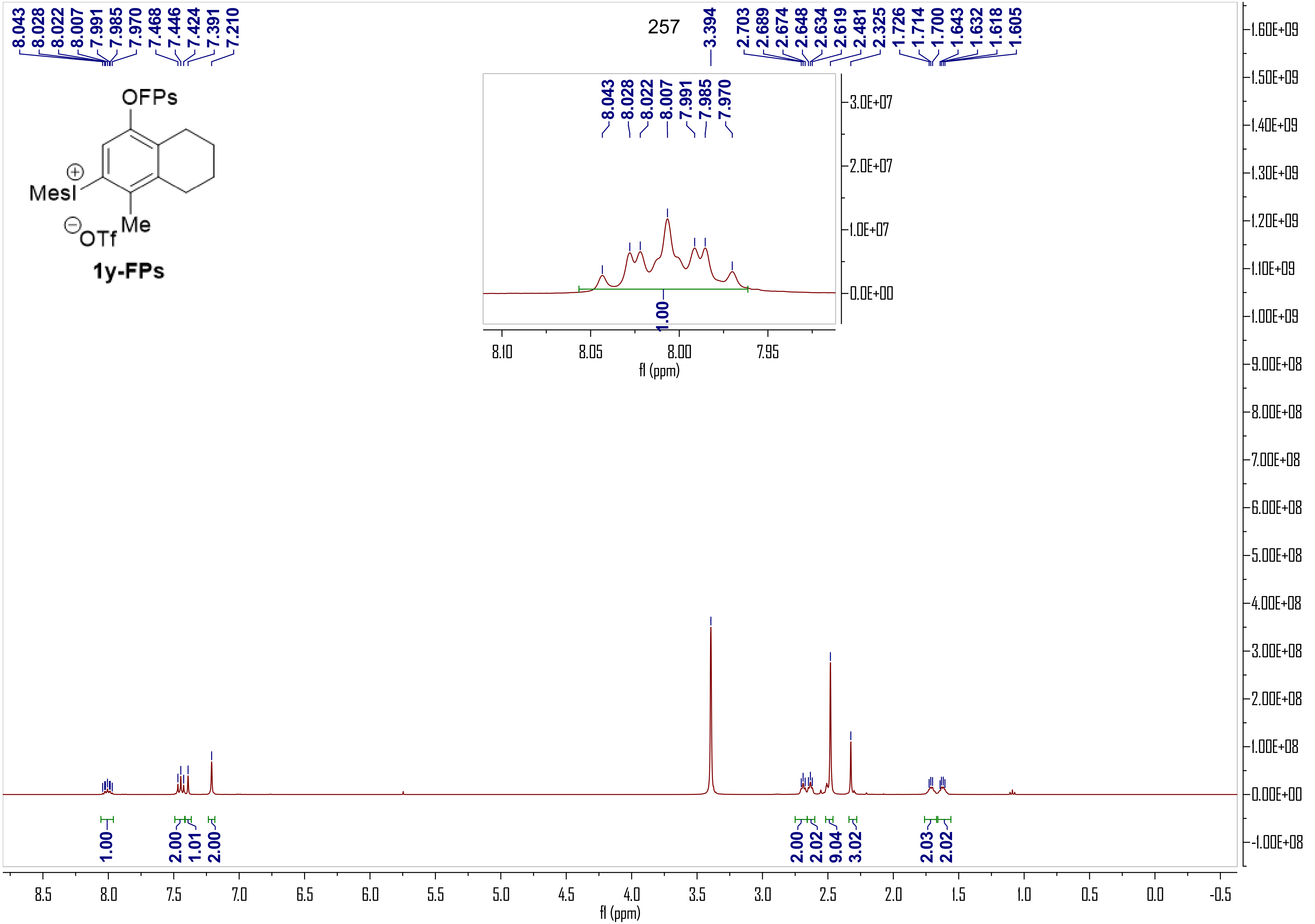

Supplementary Fig 180. **^{1}H NMR spectrum (400 MHz, DMSO-*d*6, r.t.) of 1y-FPs.**

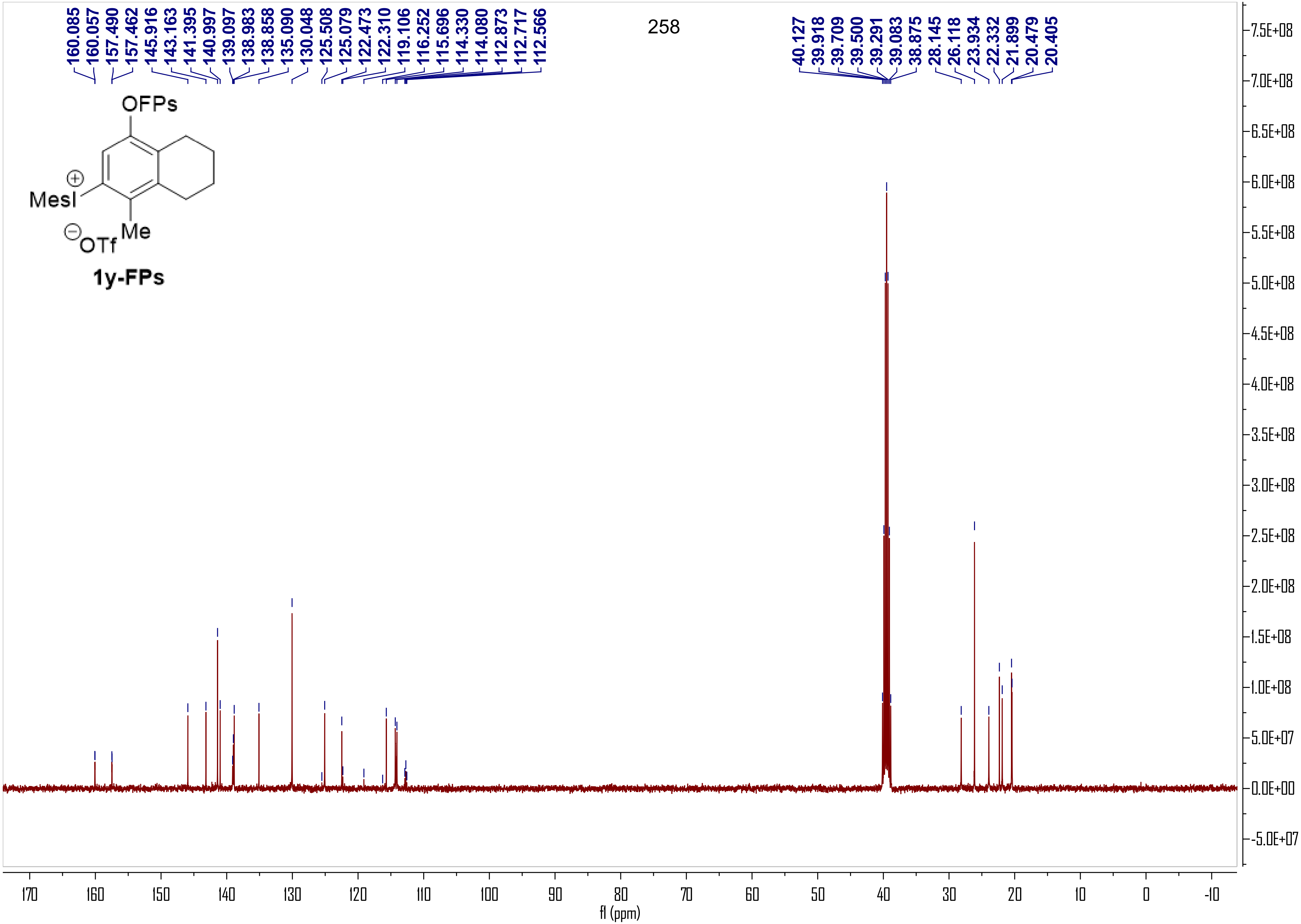

Supplementary Fig 181. ^{13}C **NMR spectrum (100 MHz, DMSO-*d*6, r.t.) of 1y-FPs.**

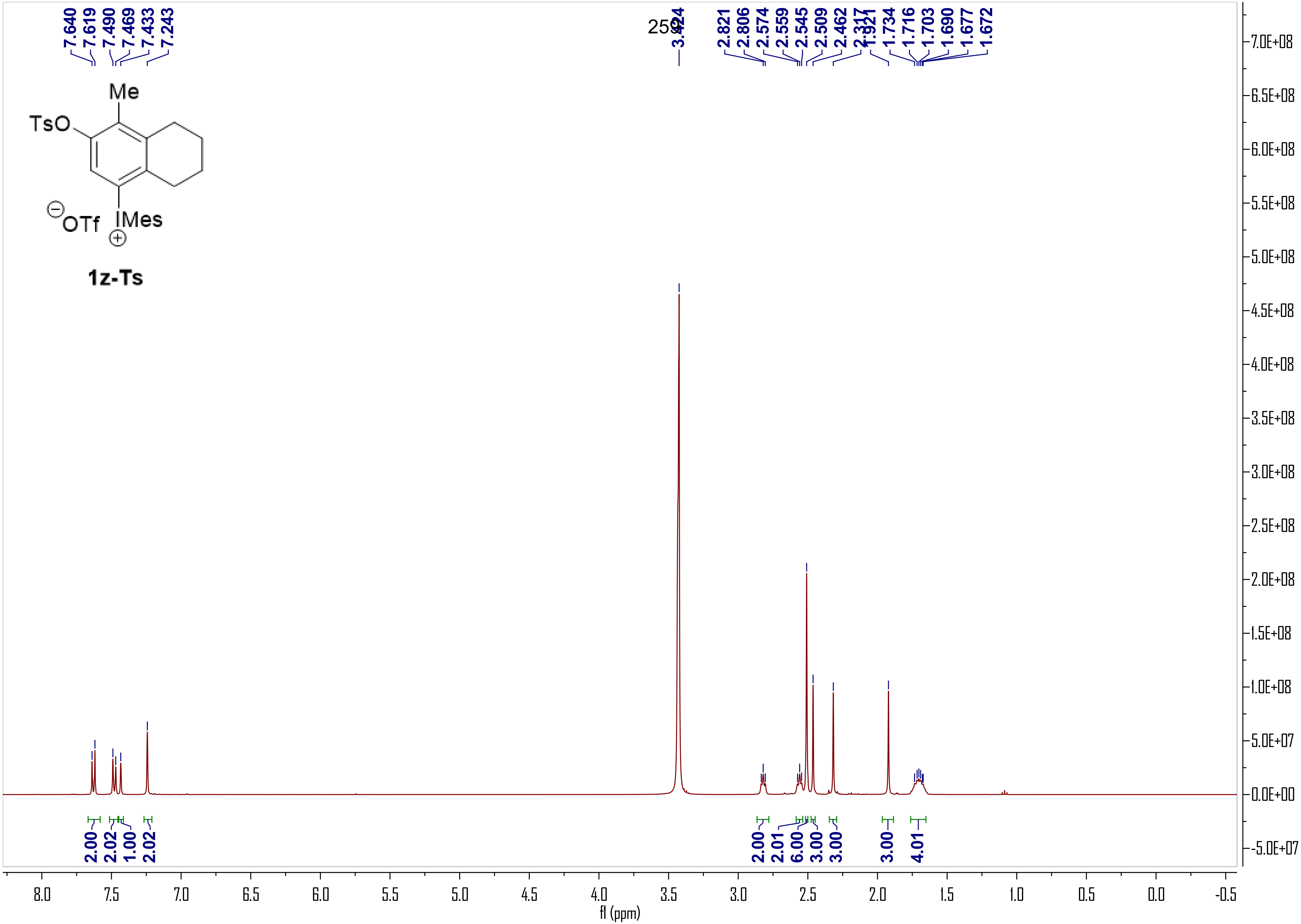

Supplementary Fig 182. [1]**H NMR spectrum (400 MHz, DMSO-*d*6, r.t.) of 1z-Ts.**

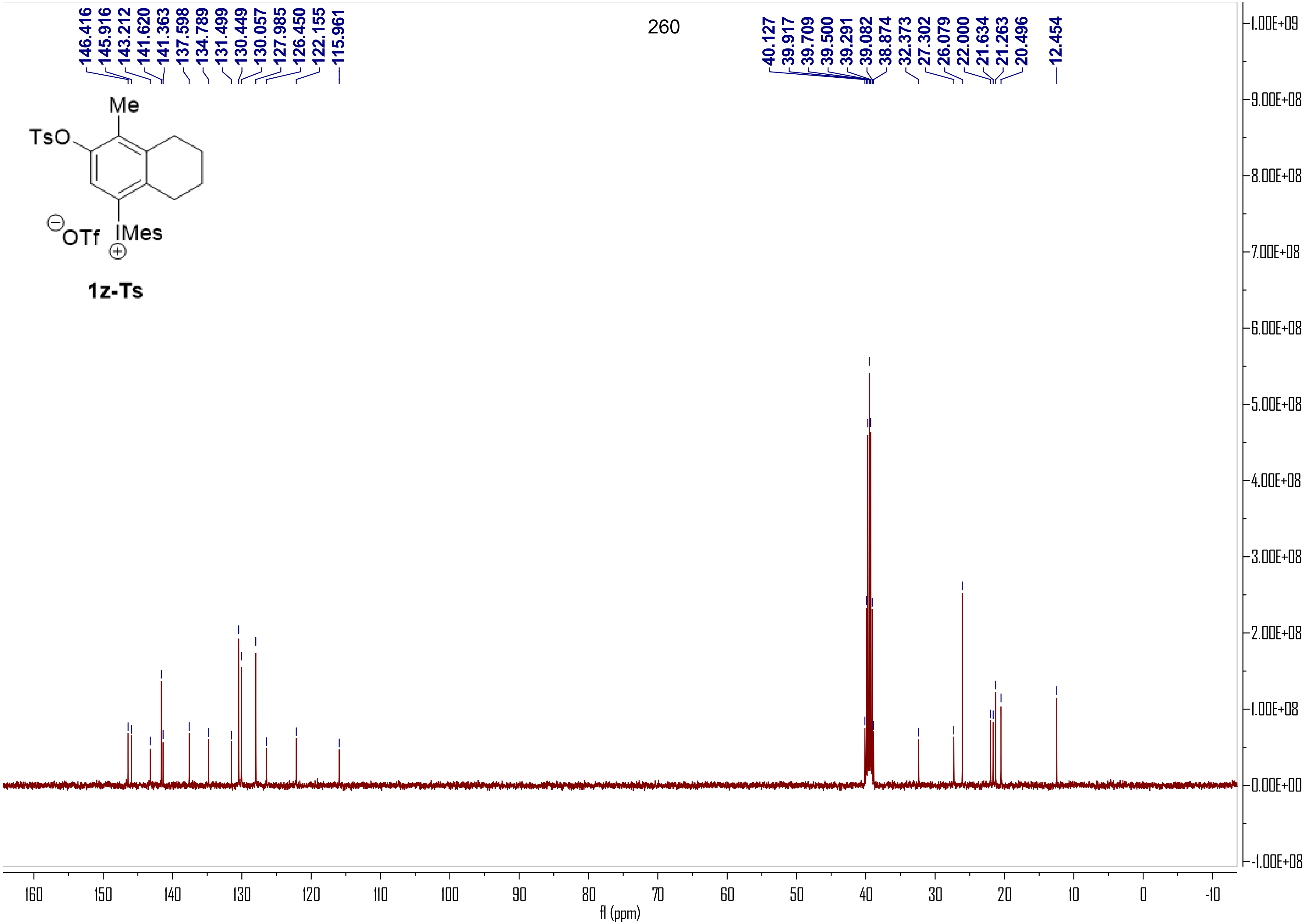

Supplementary Fig 183. ^{13}**C NMR spectrum (100 MHz, DMSO-*d*6, r.t.) of 1z-Ts.**

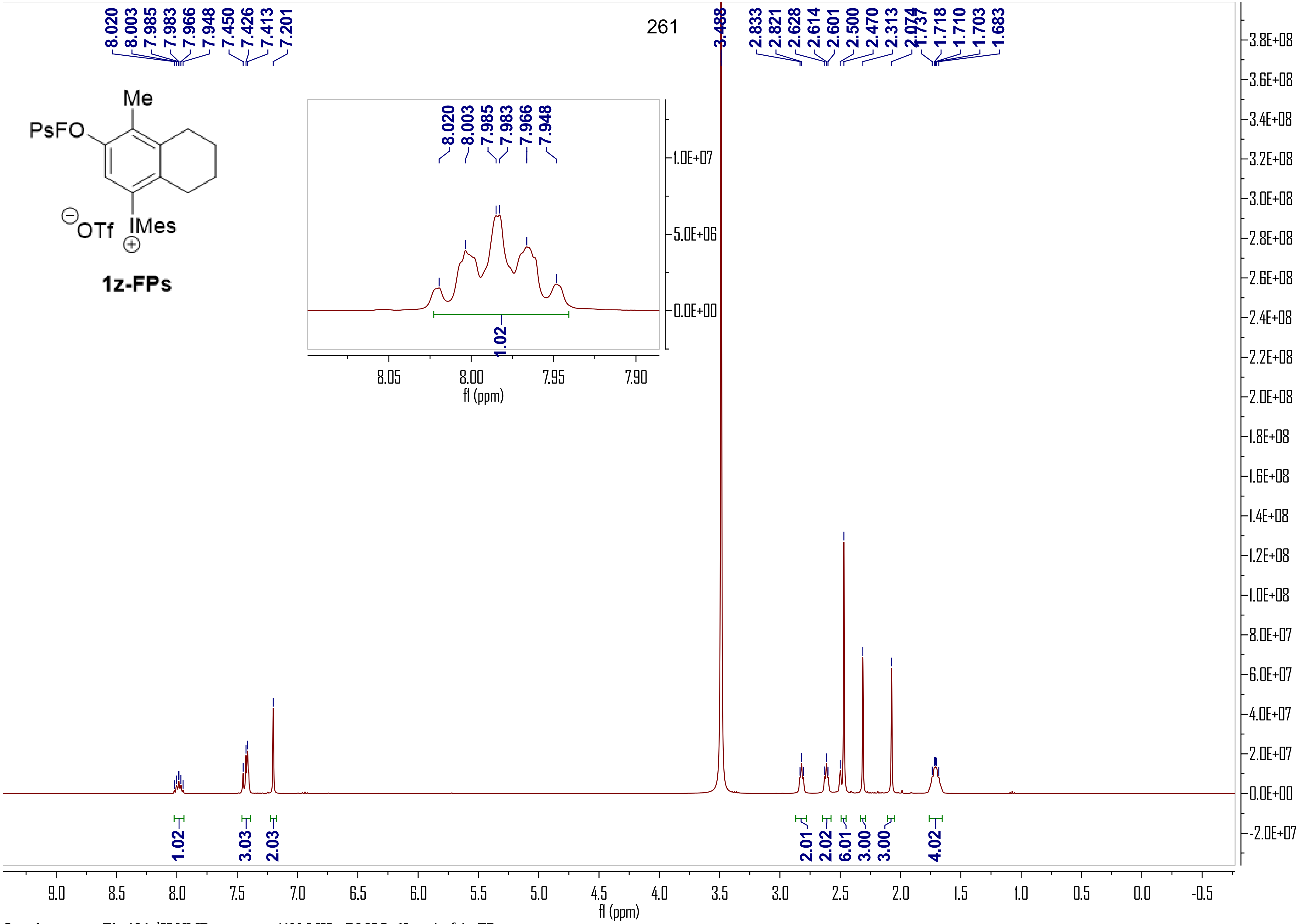

Supplementary Fig 184. **^{1}H NMR spectrum (400 MHz, DMSO-*d*6, r.t.) of 1z-FPs.**

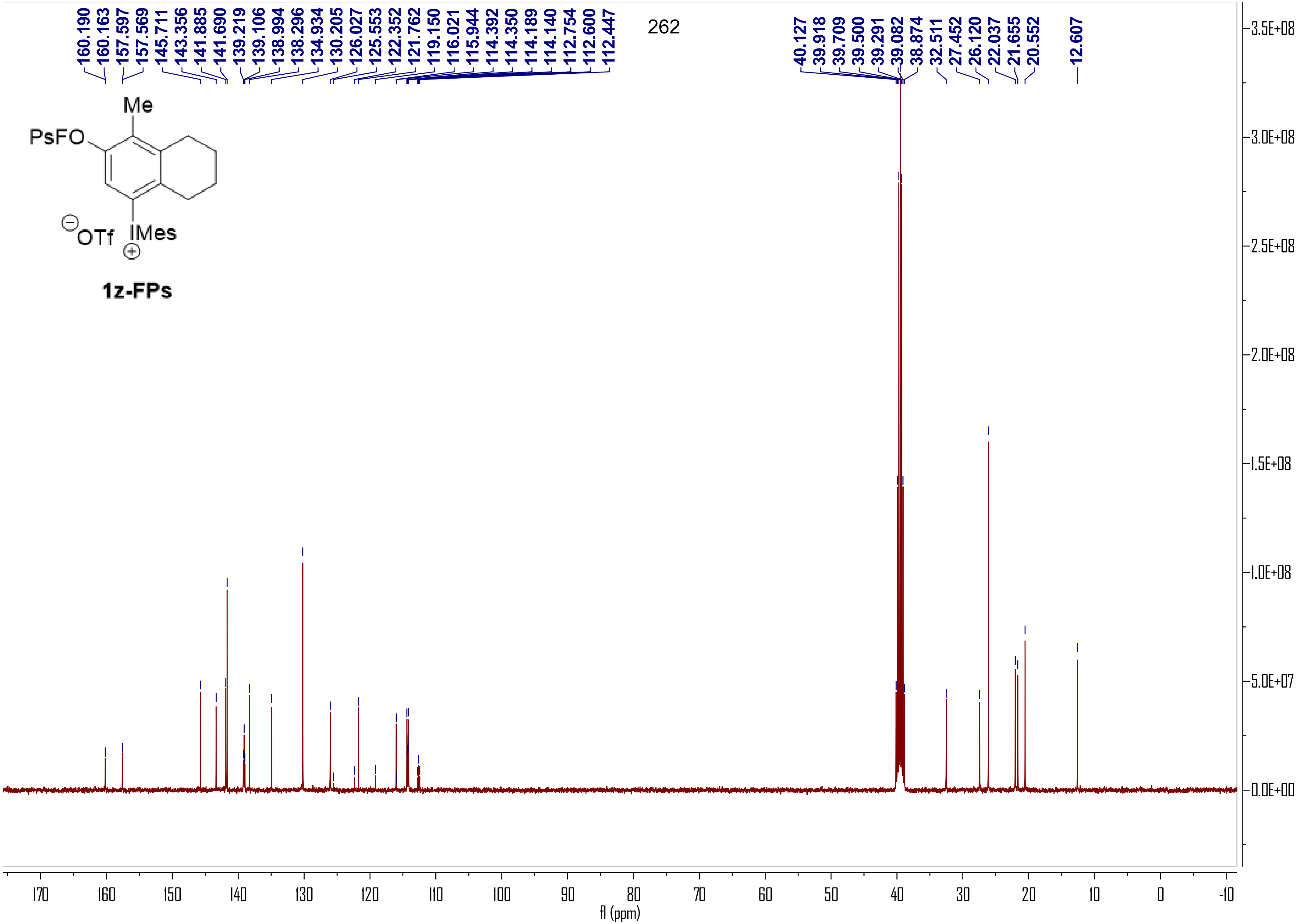

Supplementary Fig 185. **^{13}C NMR spectrum (100 MHz, DMSO-*d6*, r.t.) of 1z-FPs.**

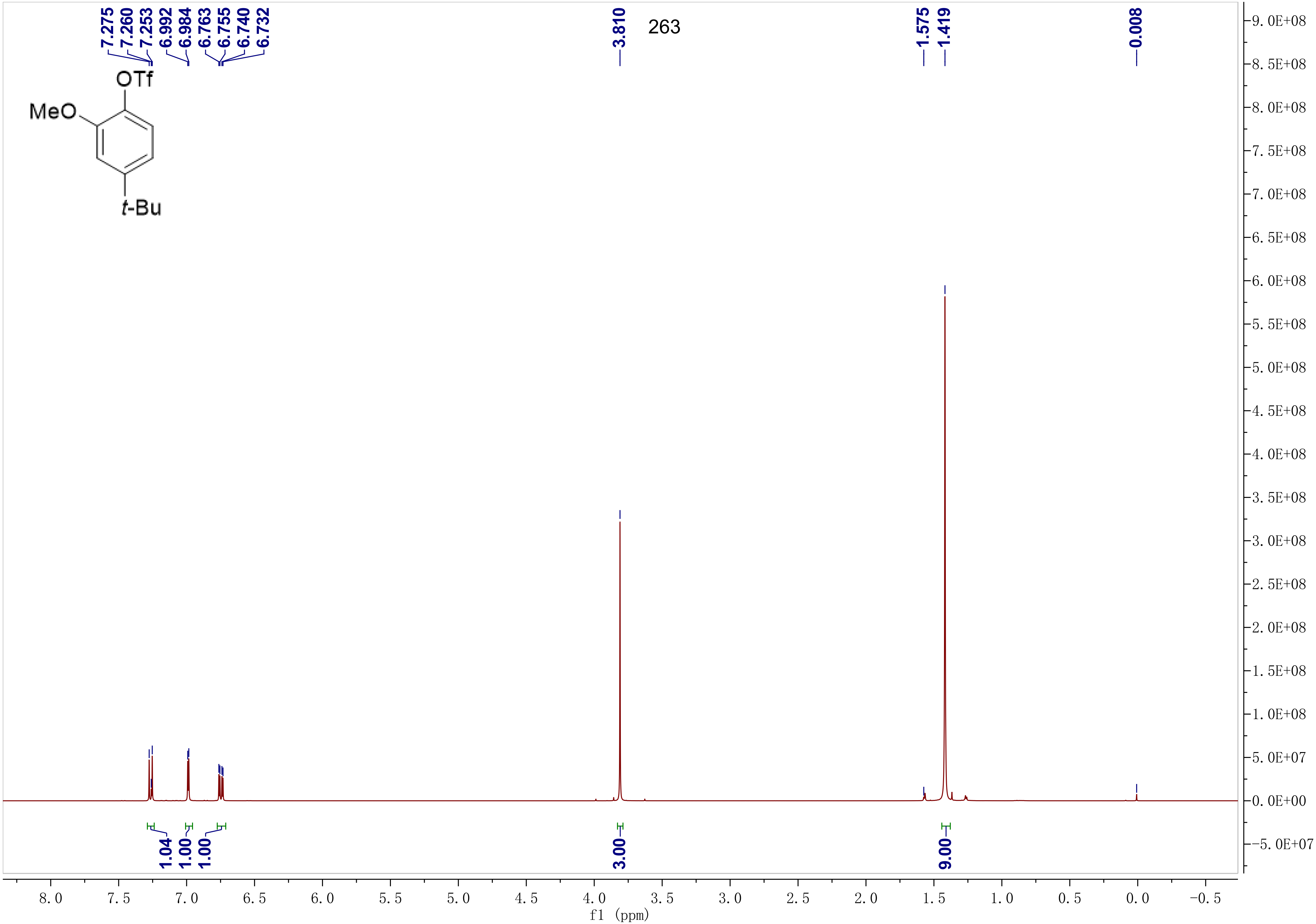

Supplementary Fig 186. **^{1}H NMR spectrum (400 MHz, $CDCl_3$, r.t.) of 4-(*tert*-butyl)-2-methoxyphenyl triflate as a colorless oil.**

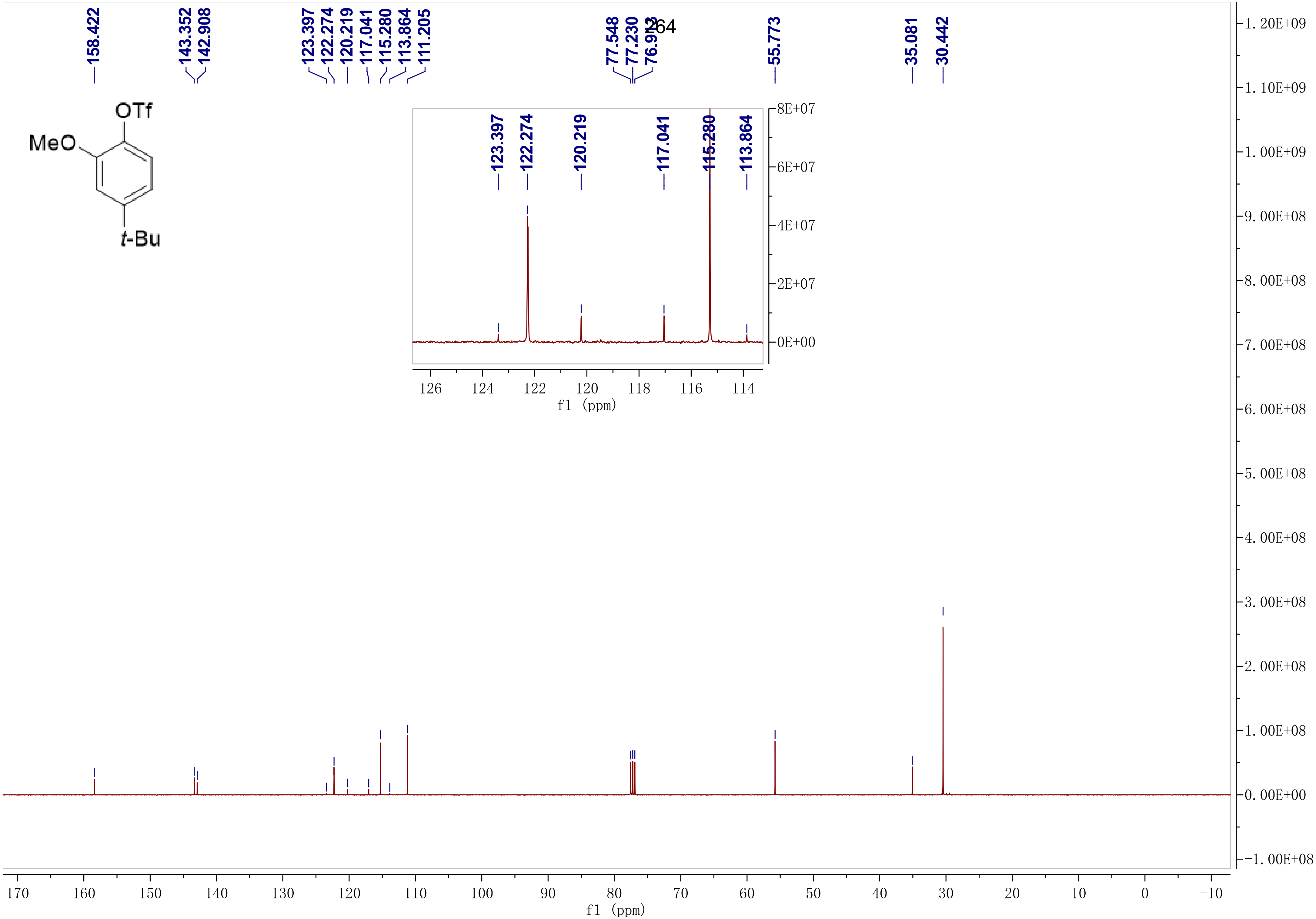

Supplementary Fig 187. ^{13}C **NMR spectrum (100 MHz,** $CDCl_3$**, r.t.) of 4-(*tert*-butyl)-2-methoxyphenyl triflate as a colorless oil.**

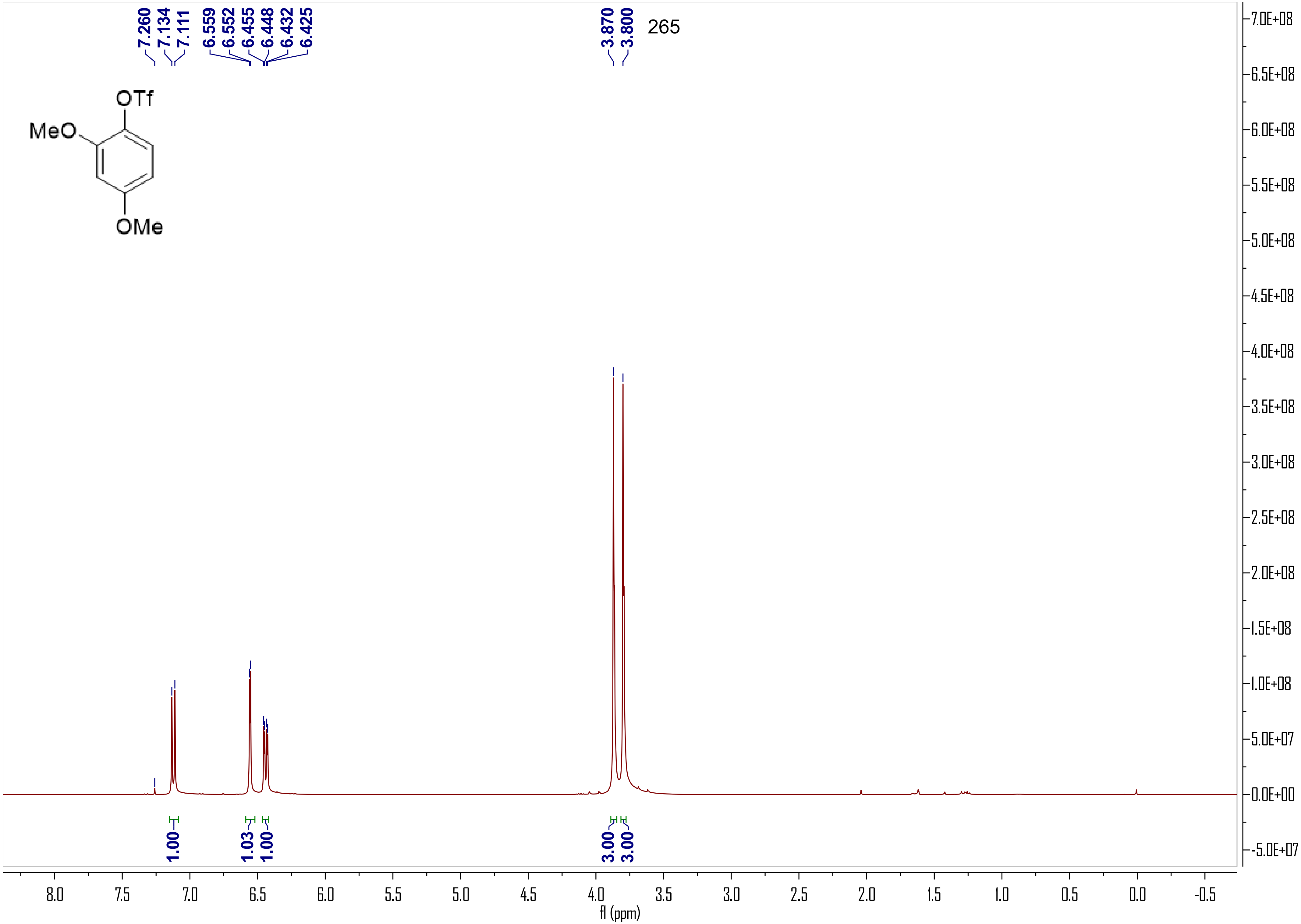

Supplementary Fig 188. ^{1}H NMR spectrum (400 MHz, $CDCl_3$, r.t.) of 2,4-dimethoxyphenyl triflate.

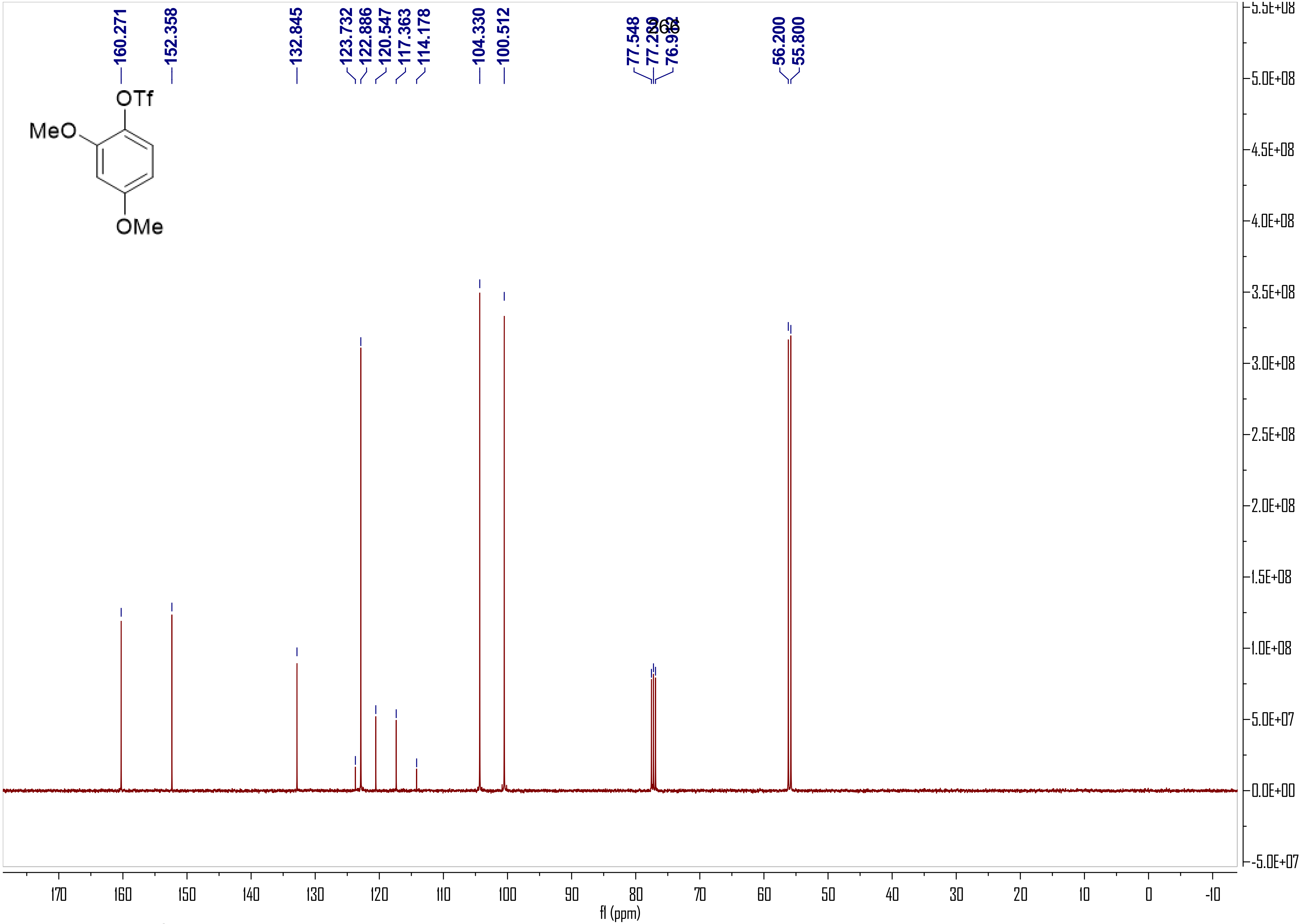

Supplementary Fig 189. ^{13}C **NMR spectrum (100 MHz, $CDCl_3$, r.t.) of 2,4-dimethoxyphenyl triflate.**

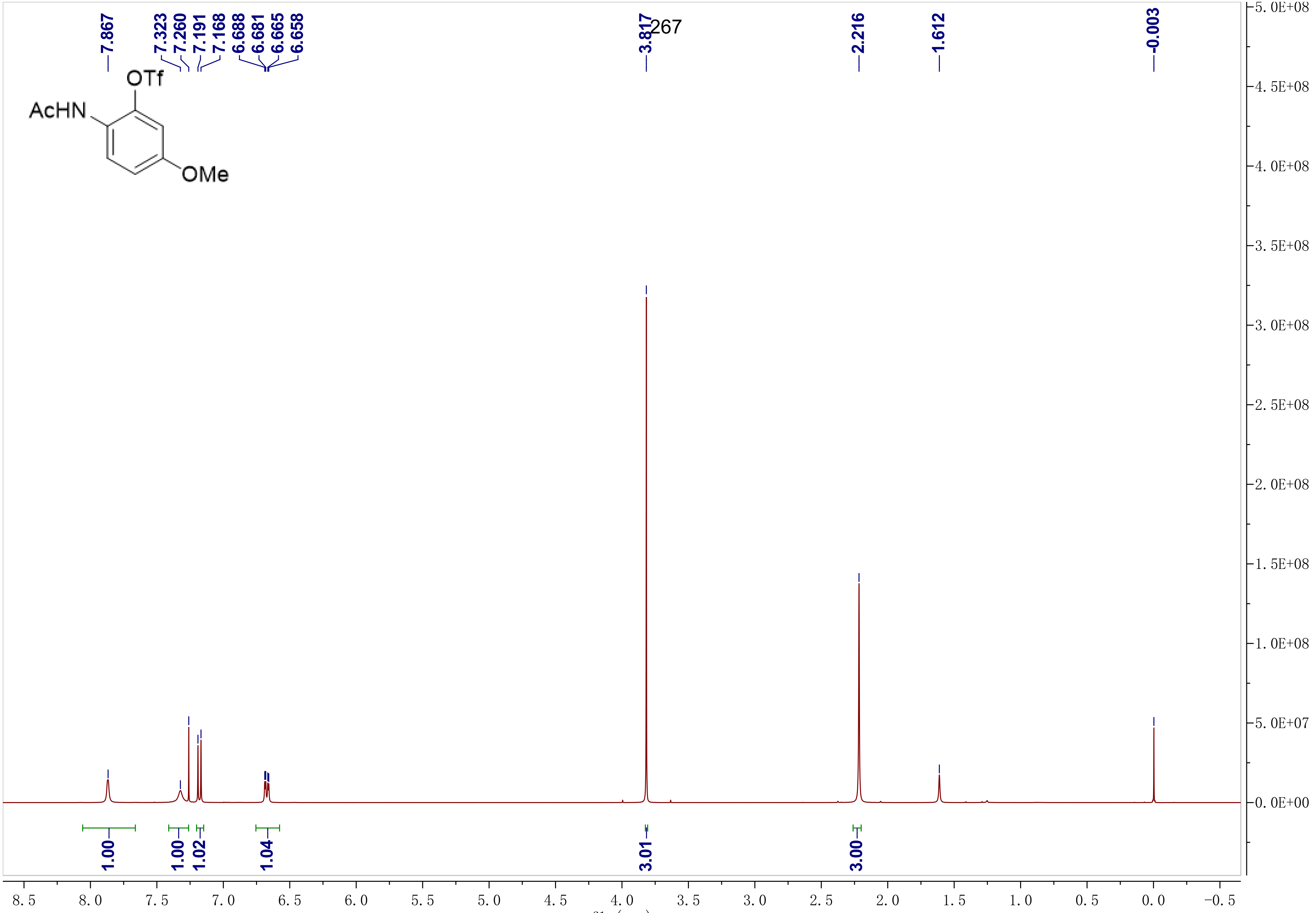

Supplementary Fig 190. ^{1}**H NMR spectrum (400 MHz,** $CDCl_3$**, r.t.) of 2-acetamido-5-methoxyphenyl triflate.**

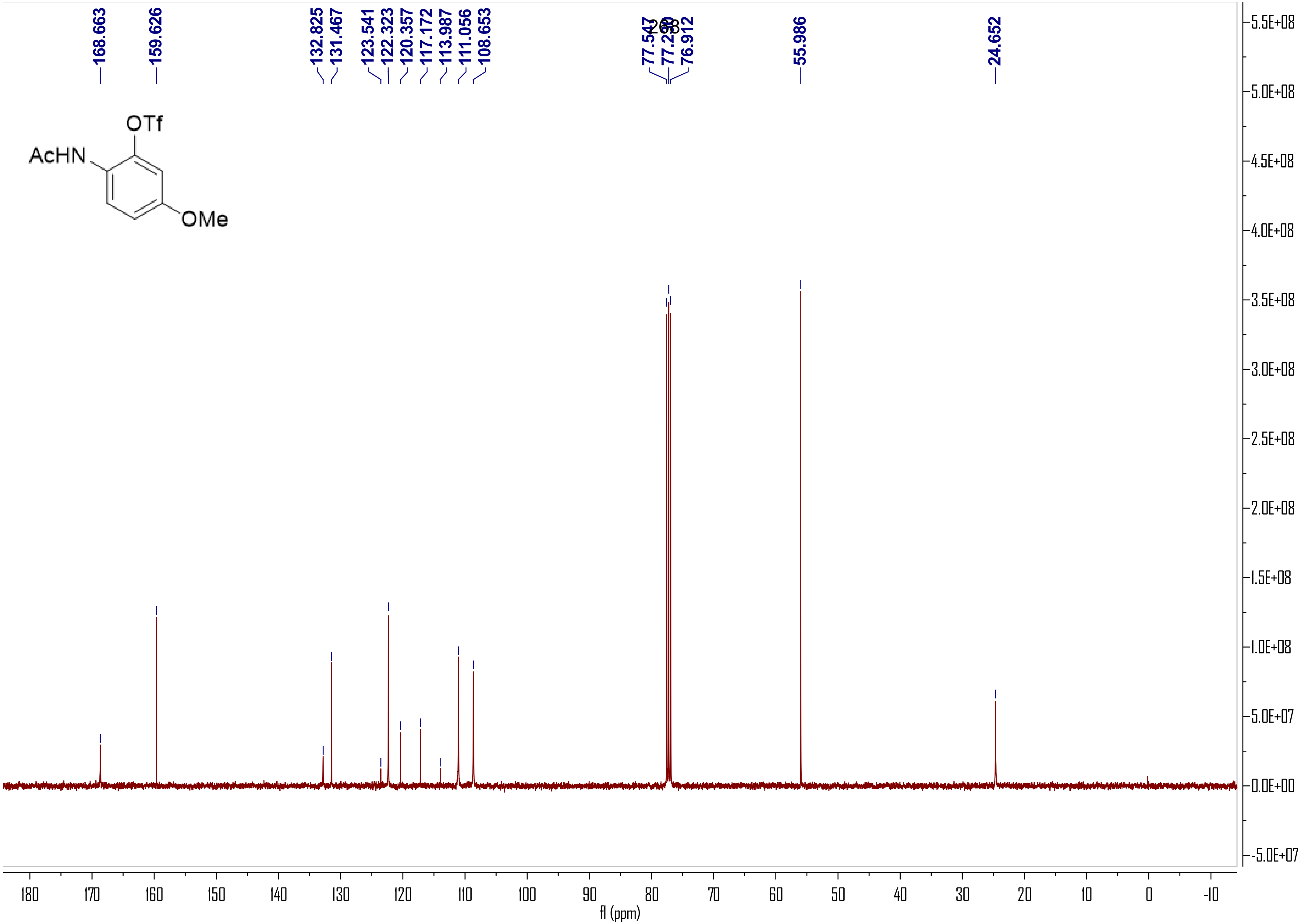

Supplementary Fig 191. **^{13}C NMR spectrum (100 MHz, $CDCl_3$, r.t.) of 2-acetamido-5-methoxyphenyl triflate.**

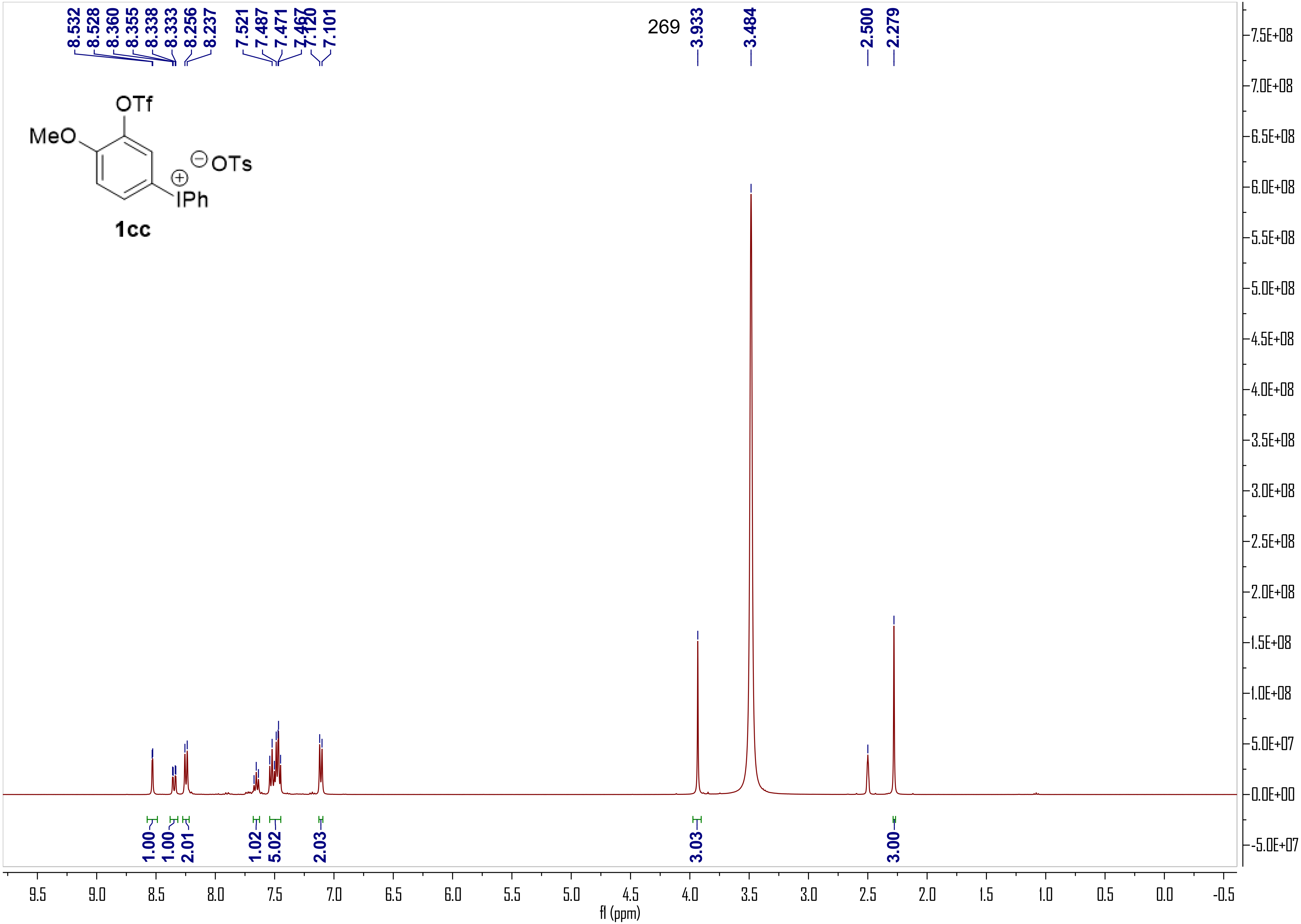

Supplementary Fig 192. ¹H NMR spectrum (400 MHz, DMSO-*d*6, r.t.) of 1cc.

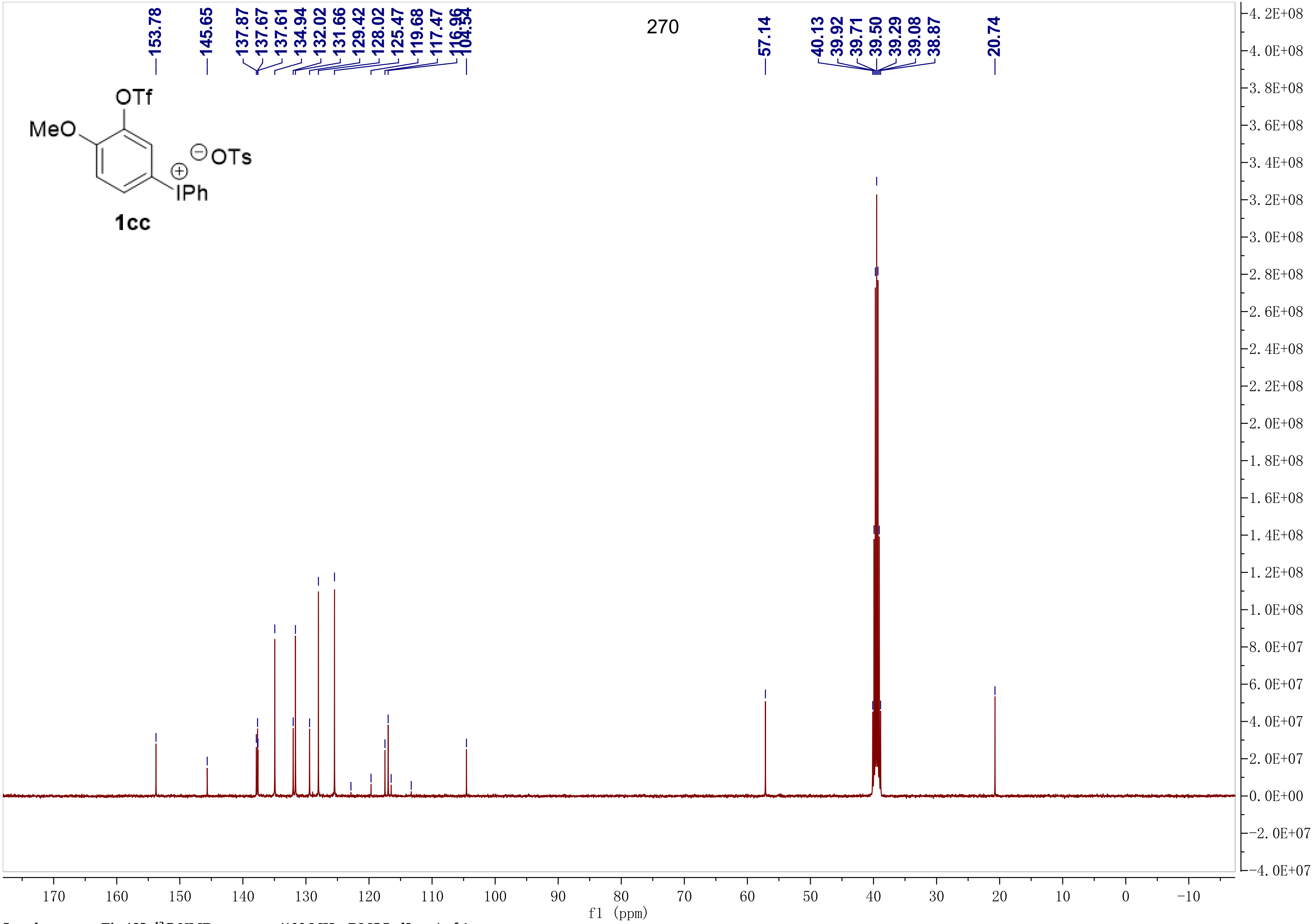

Supplementary Fig 193. ^{13}C NMR spectrum (100 MHz, DMSO-*d*6, r.t.) of 1cc.

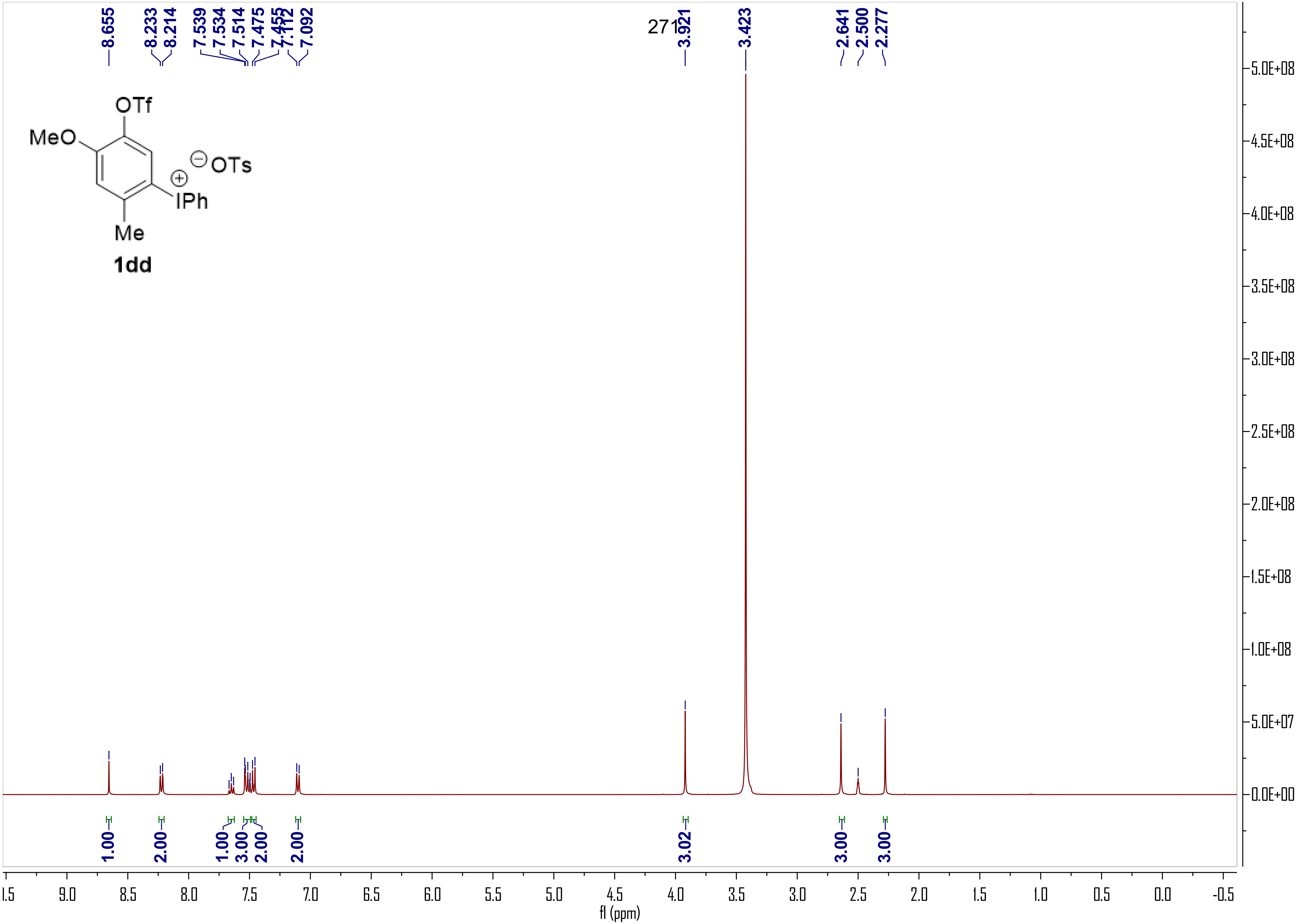

Supplementary Fig 194. **^{1}H NMR spectrum (400 MHz, DMSO-*d*6, r.t.) of 1dd.**

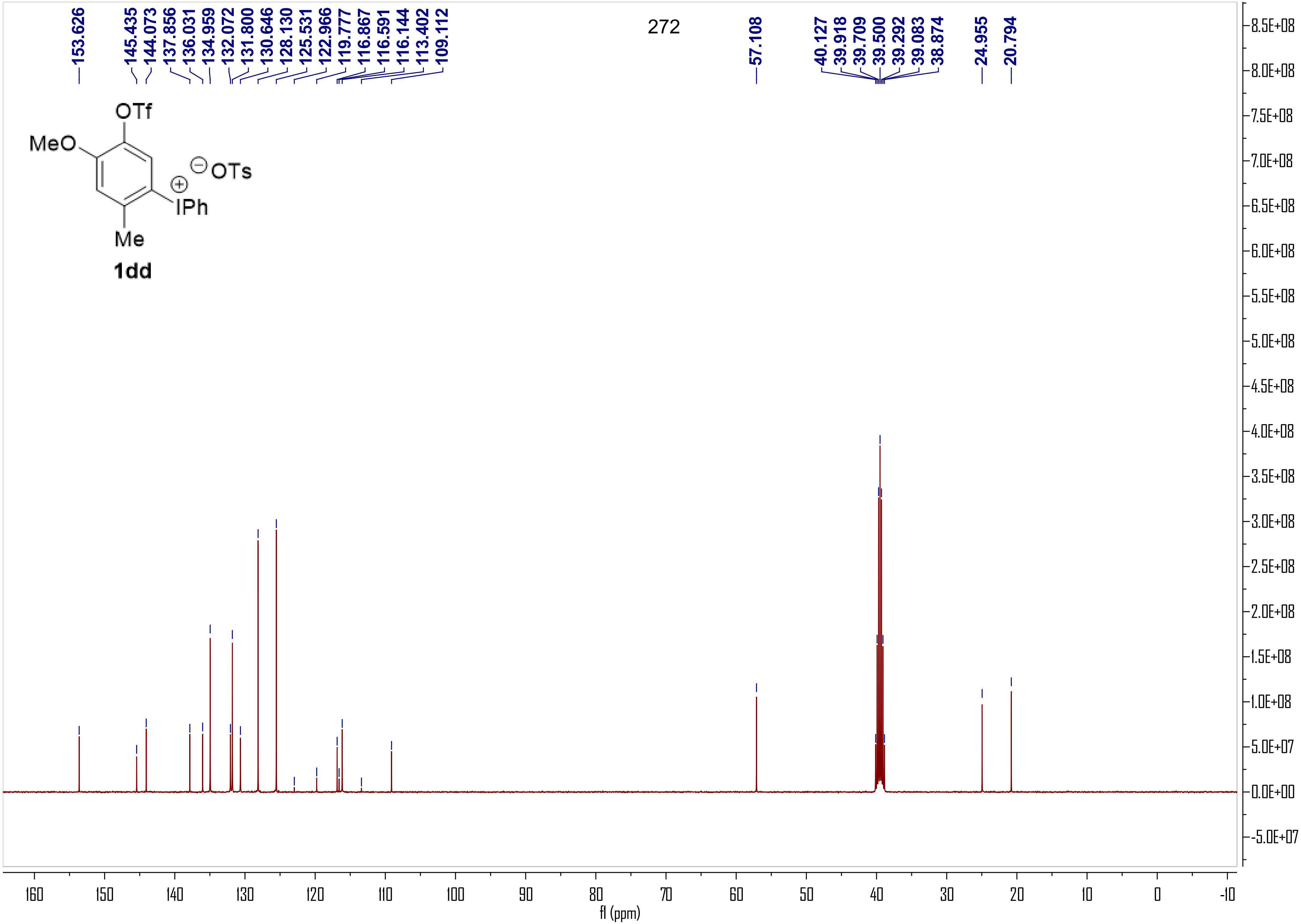

Supplementary Fig 195. ^{13}**C NMR spectrum (100 MHz, DMSO-*d*6, r.t.) of 1dd.**

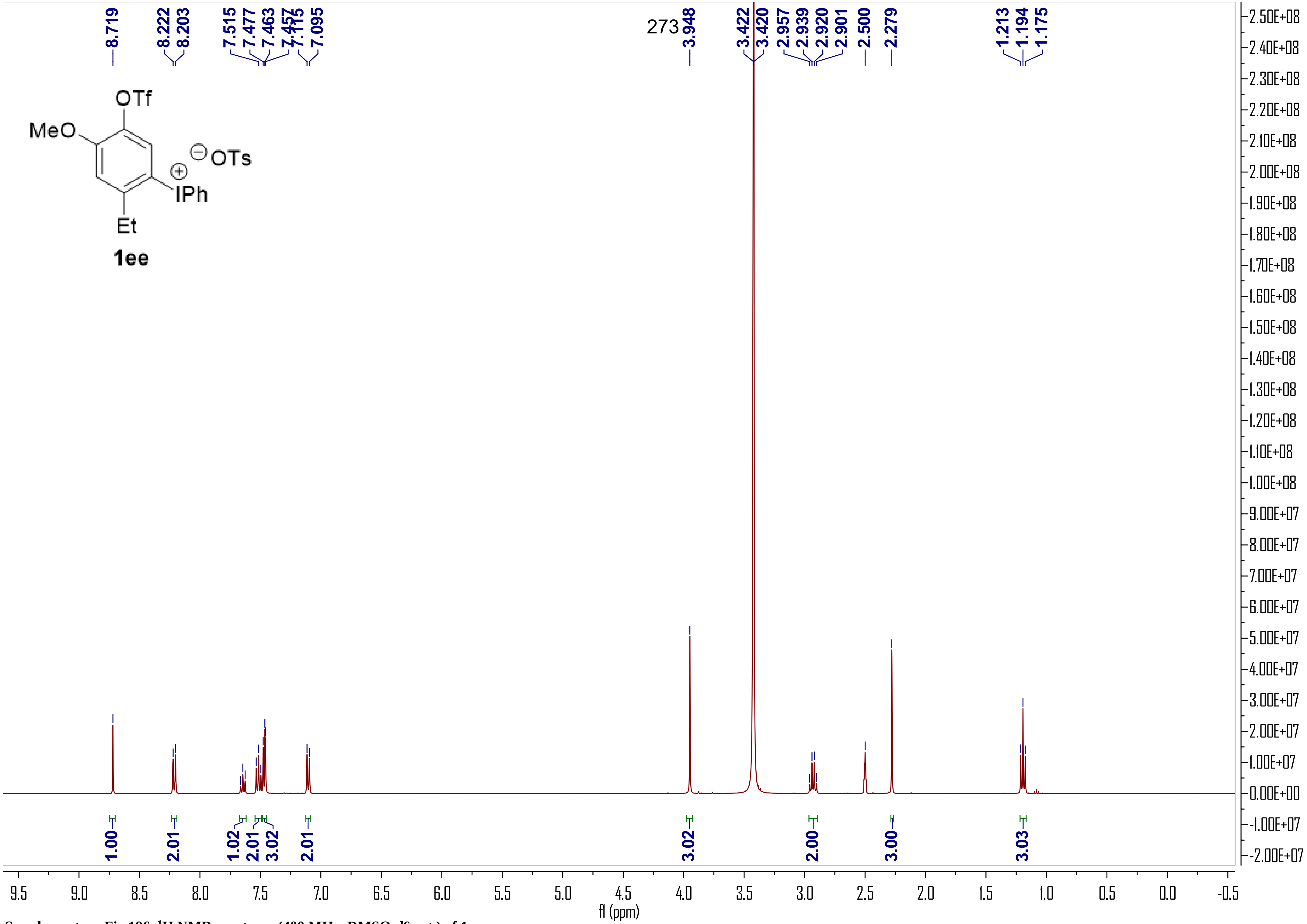

Supplementary Fig 196. ¹H NMR spectrum (400 MHz, DMSO-*d*6, r.t.) of 1ee.

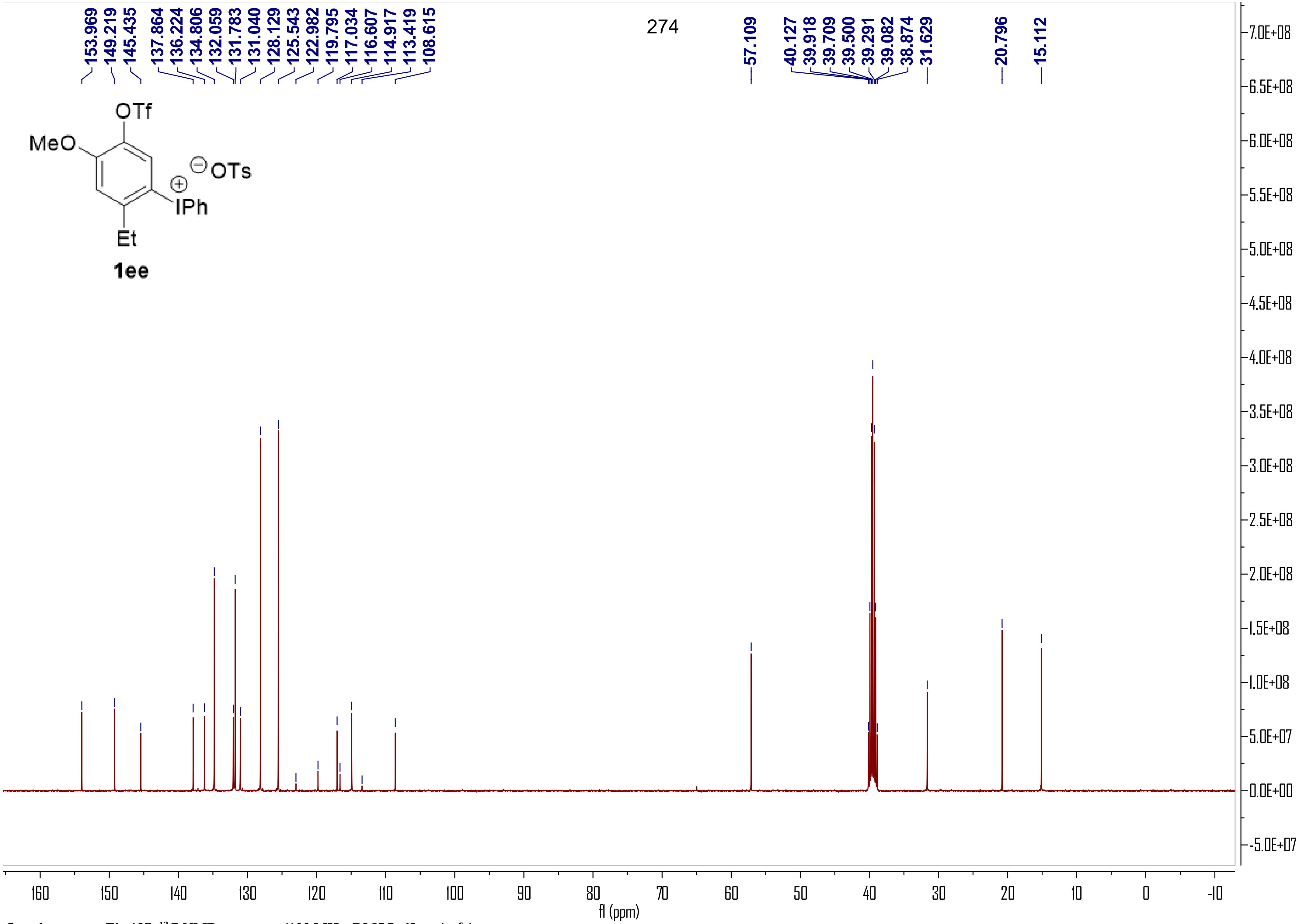

Supplementary Fig 197. ^{13}C NMR spectrum (100 MHz, DMSO-*d*6, r.t.) of 1ee.

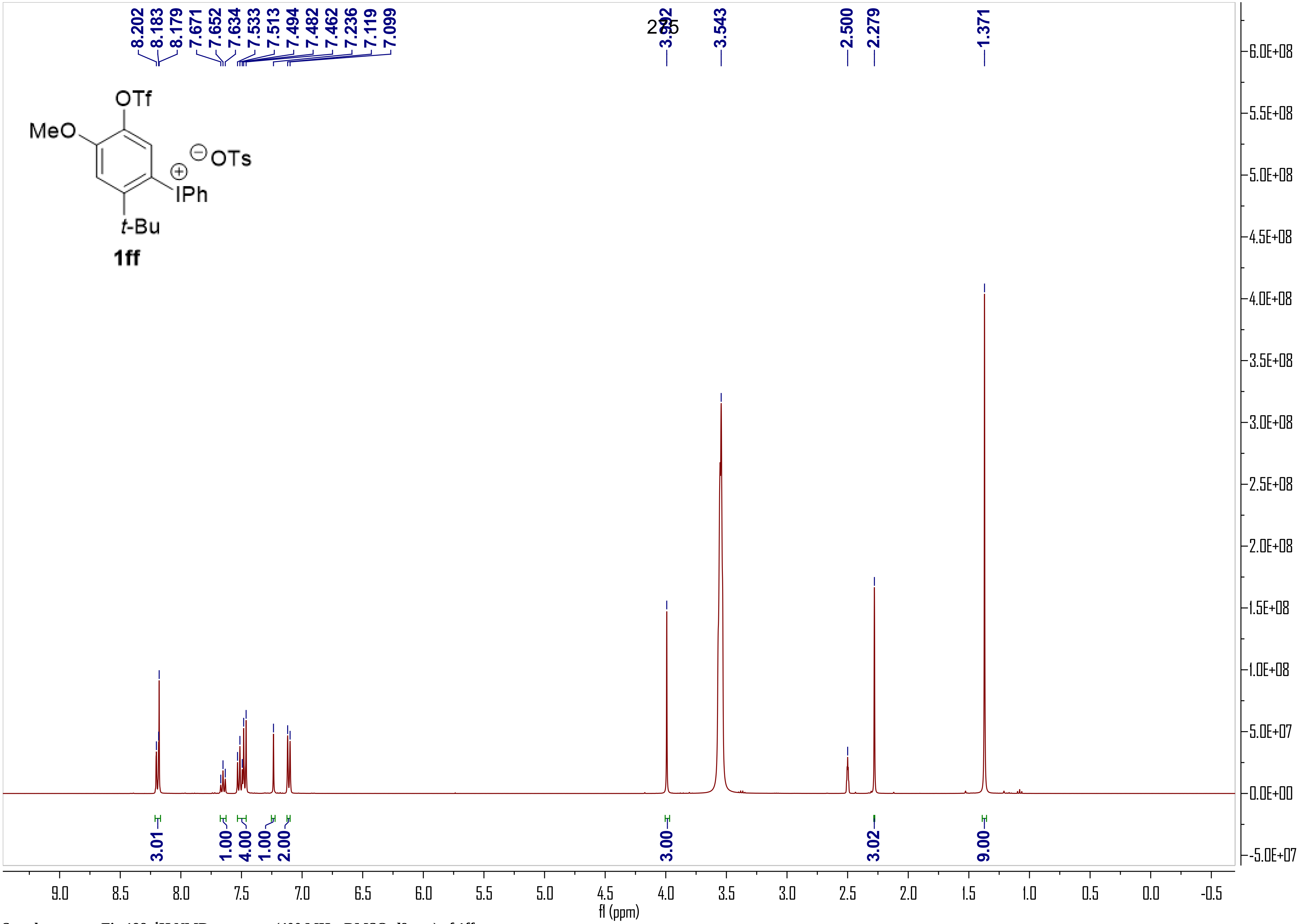

Supplementary Fig 198. **^{1}H NMR spectrum (400 MHz, DMSO-*d*6, r.t.) of 1ff.**

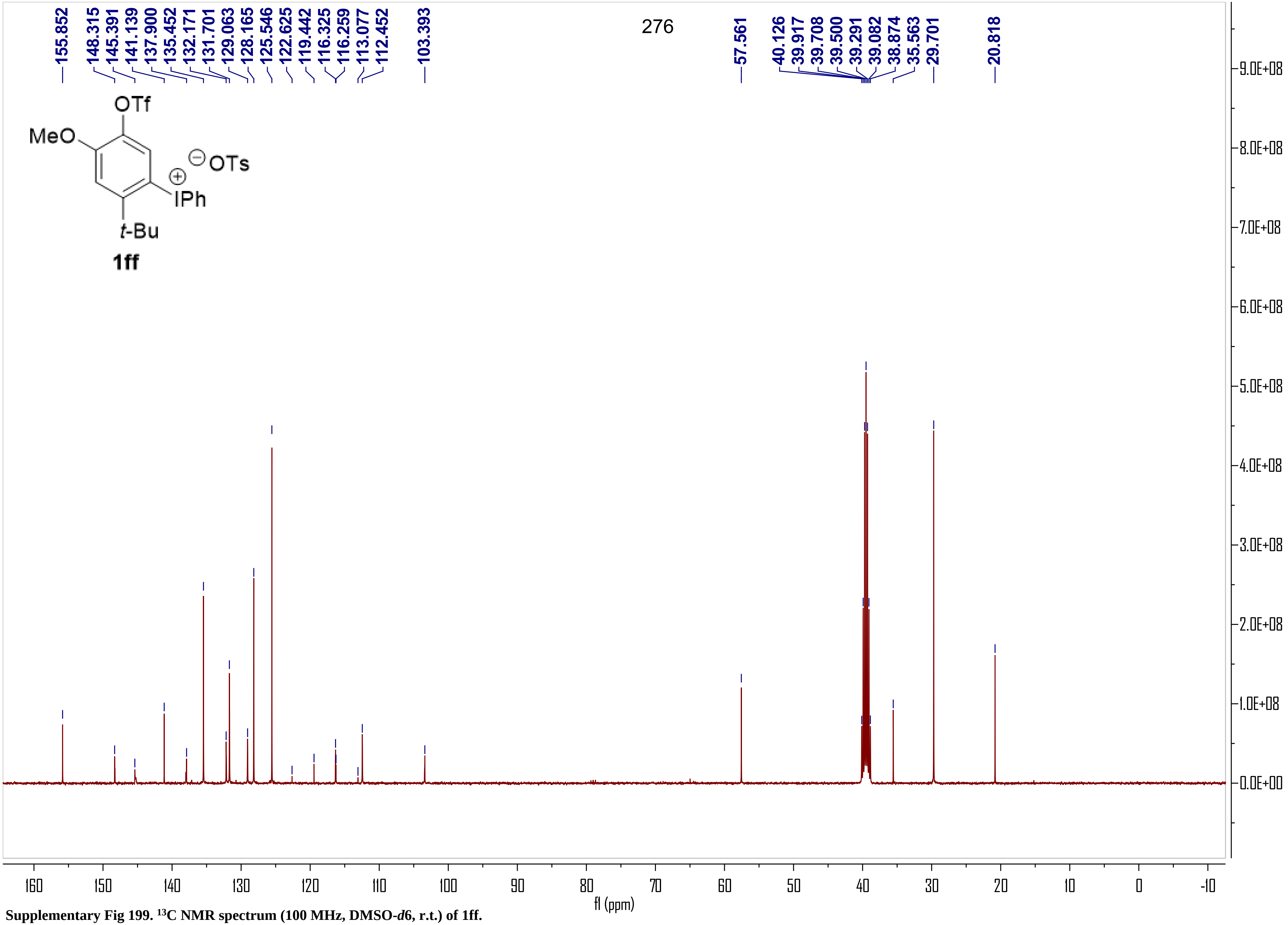

Supplementary Fig 199. ^{13}**C NMR spectrum (100 MHz, DMSO-*d*6, r.t.) of 1ff.**

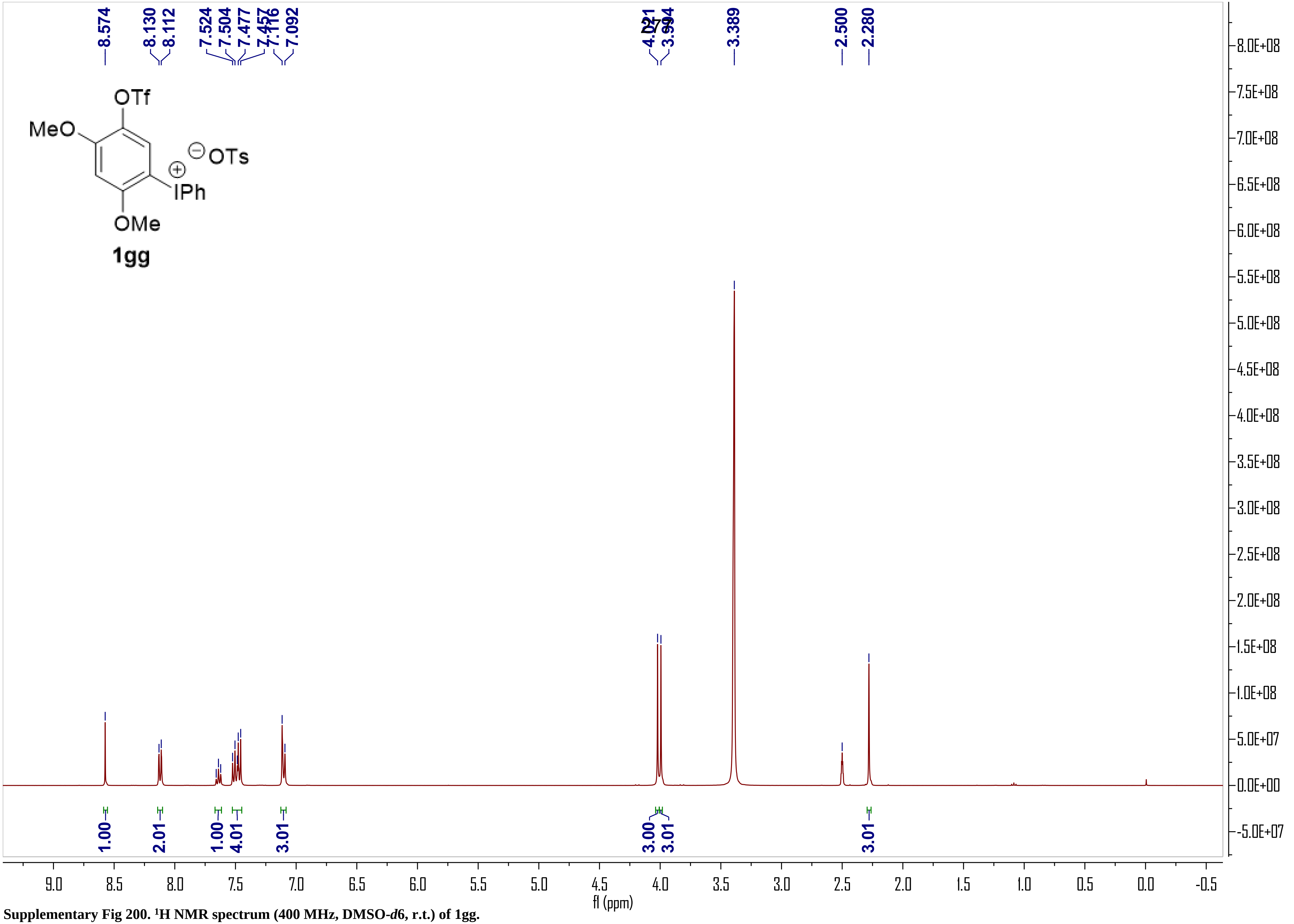

Supplementary Fig 200. **¹H NMR spectrum (400 MHz, DMSO-*d*6, r.t.) of 1gg.**

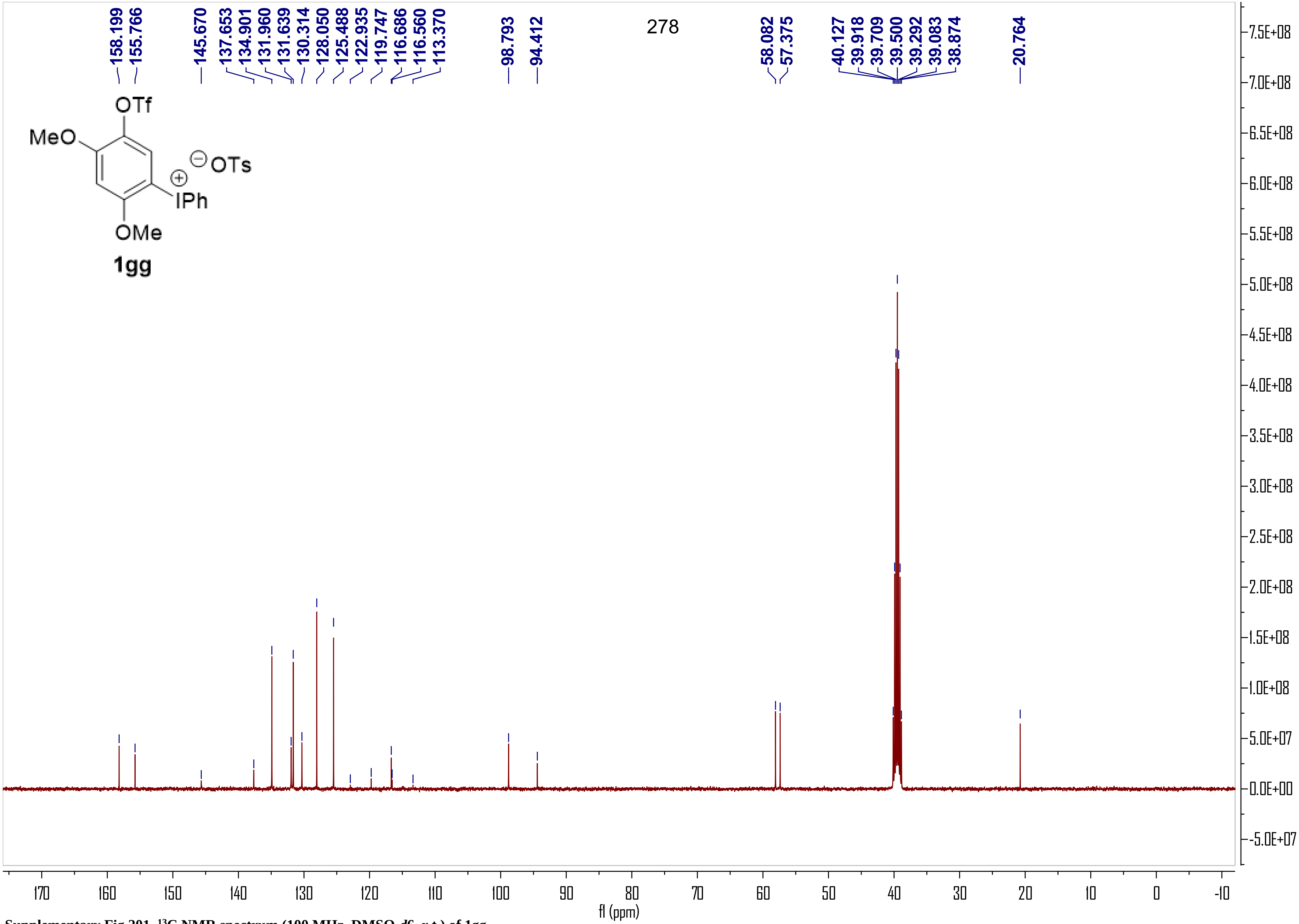

Supplementary Fig 201. ^{13}**C NMR spectrum (100 MHz, DMSO-*d*6, r.t.) of 1gg.**

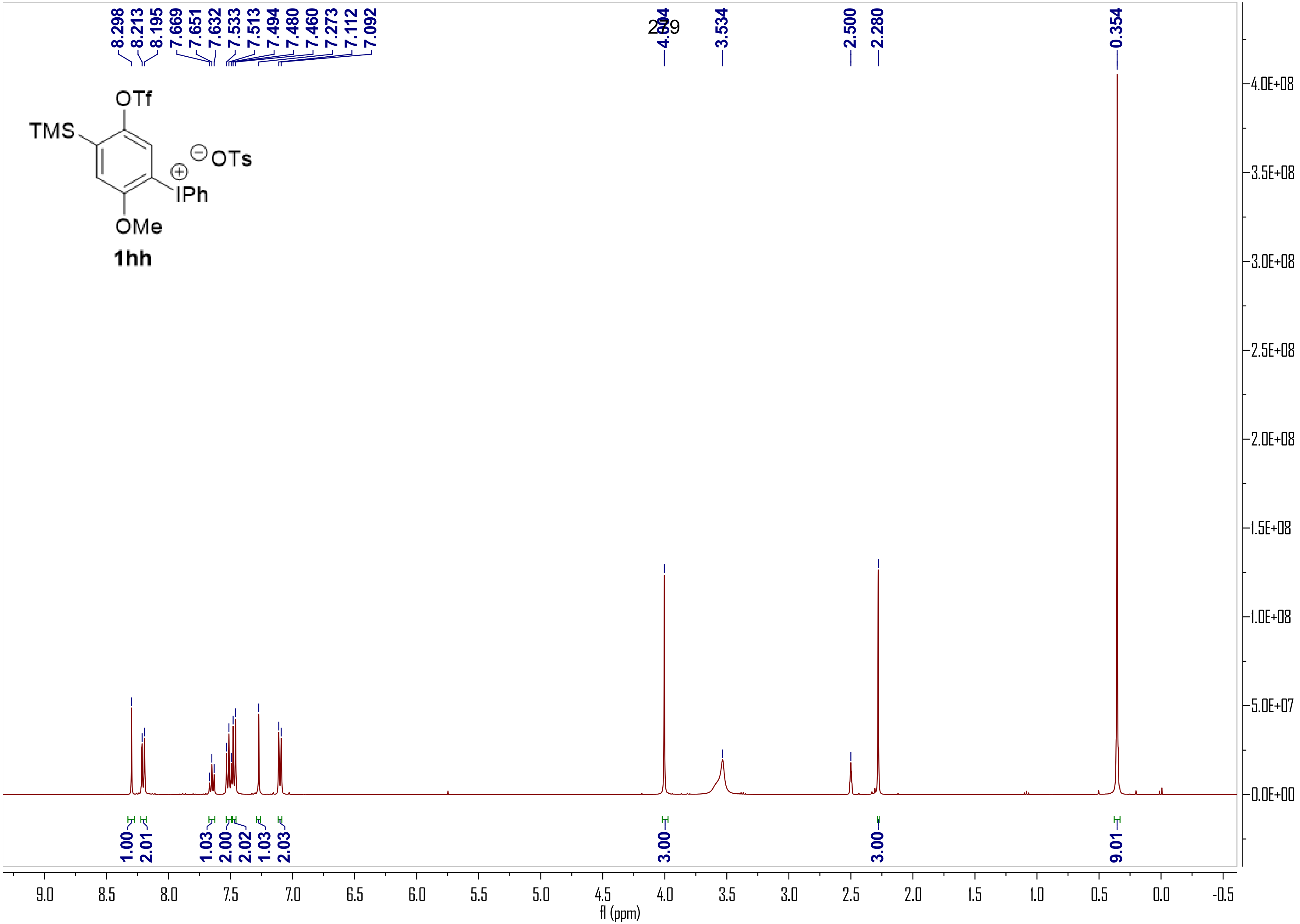

Supplementary Fig 202. **¹H NMR spectrum (400 MHz, DMSO-*d*6, r.t.) of 1hh.**

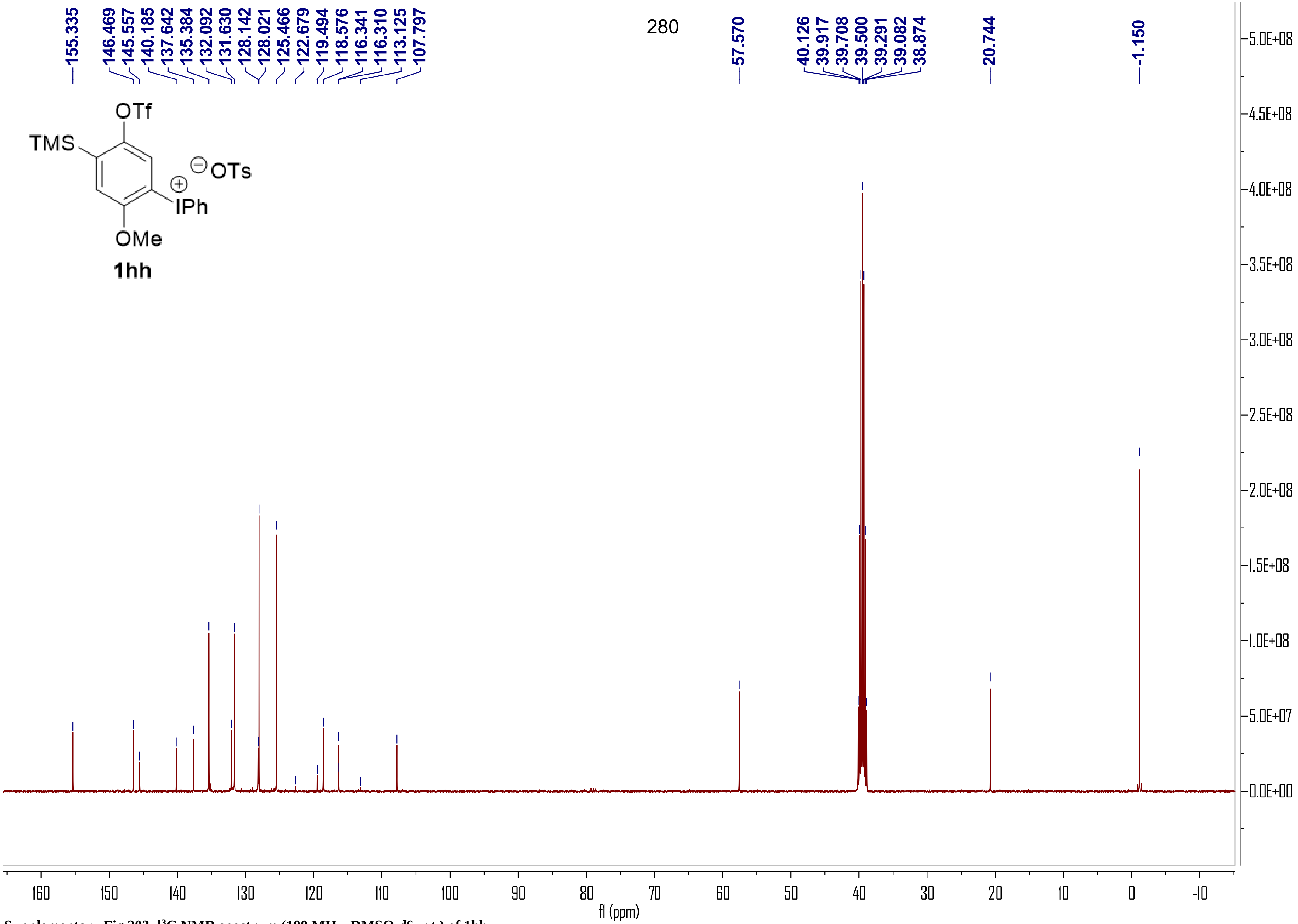

Supplementary Fig 203. ^{13}C NMR spectrum (100 MHz, DMSO-*d*6, r.t.) of 1hh.

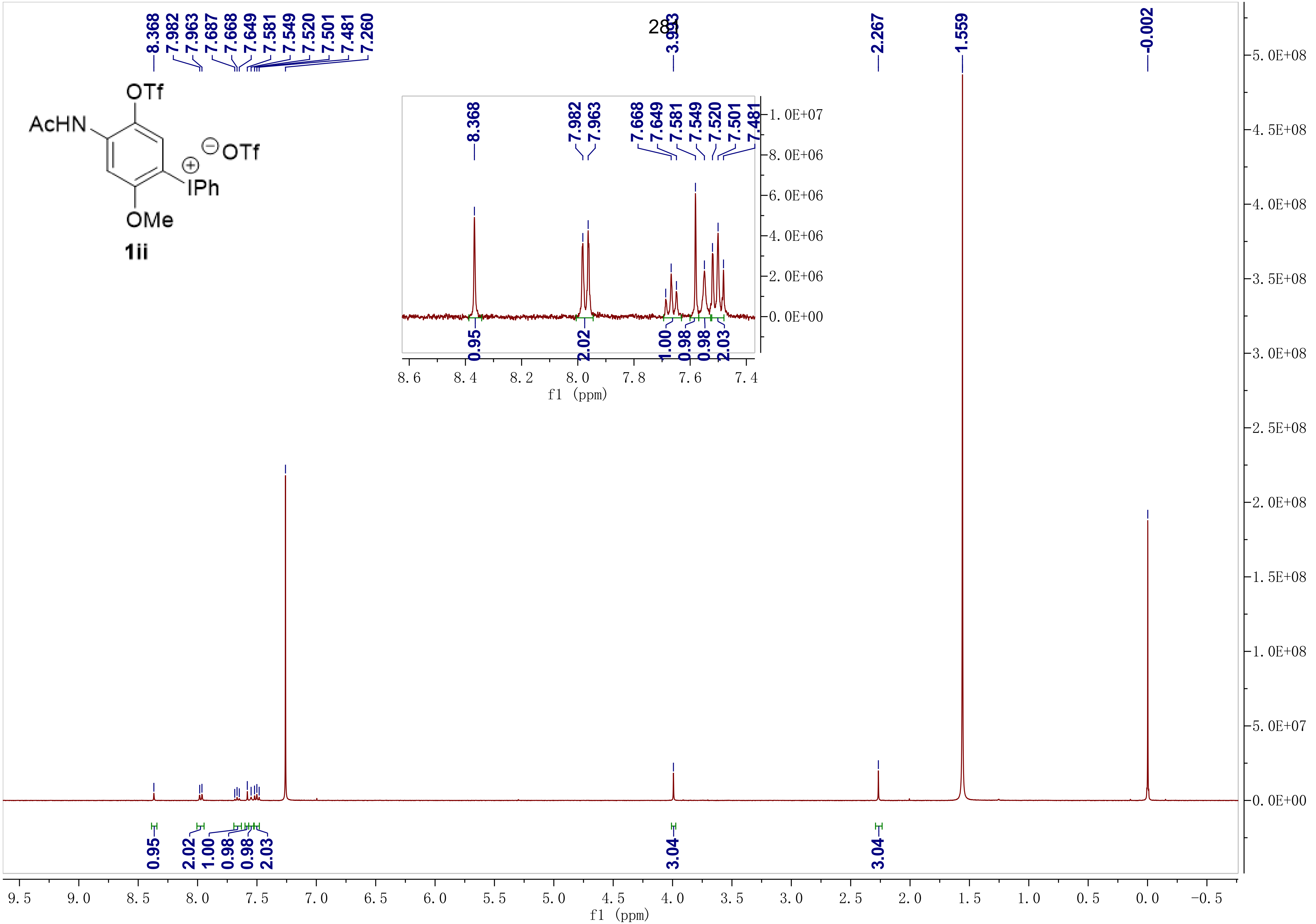

Supplementary Fig 204. **^{1}H NMR spectrum (400 MHz, DMSO-*d*6, r.t.) of 1ii.**

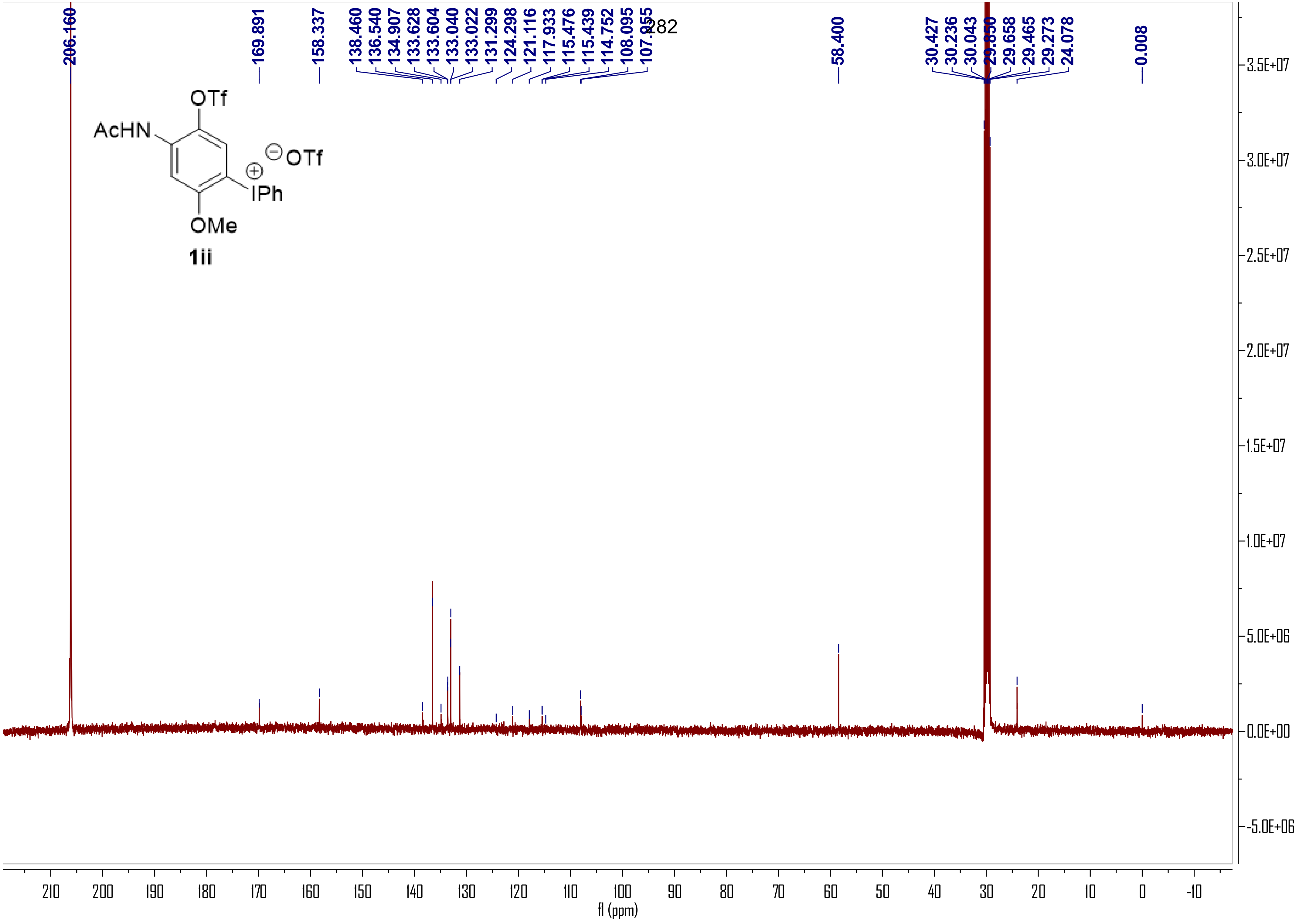

Supplementary Fig 205. ^{13}C NMR spectrum (100 MHz, DMSO-*d*6, r.t.) of 1ii.

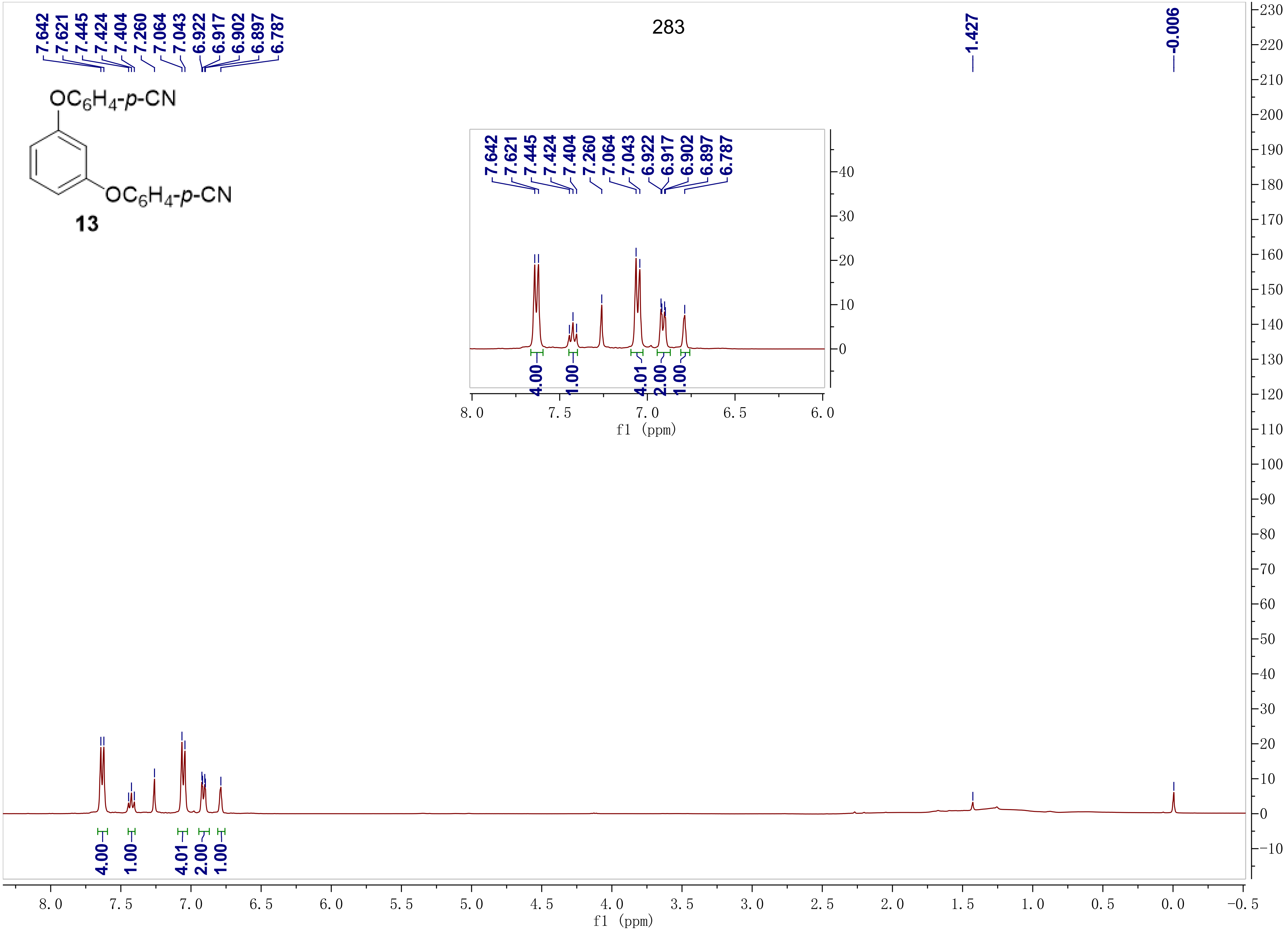

Supplementary Fig 206. ^{1}H NMR spectrum (400 MHz, $CDCl_3$, r.t.) of 13.

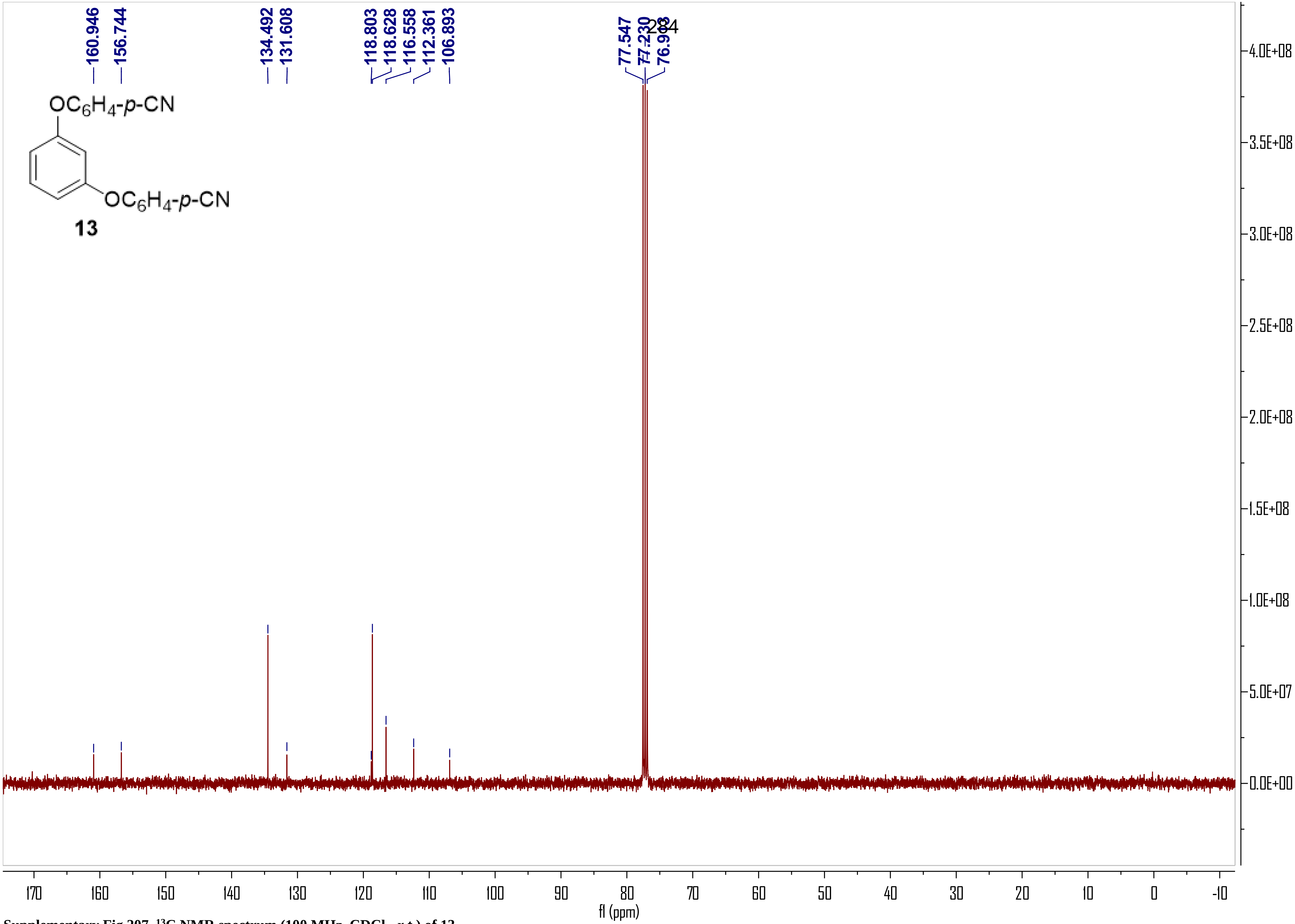

Supplementary Fig 207. ^{13}C **NMR spectrum (100 MHz,** $CDCl_3$**, r.t.) of 13.**

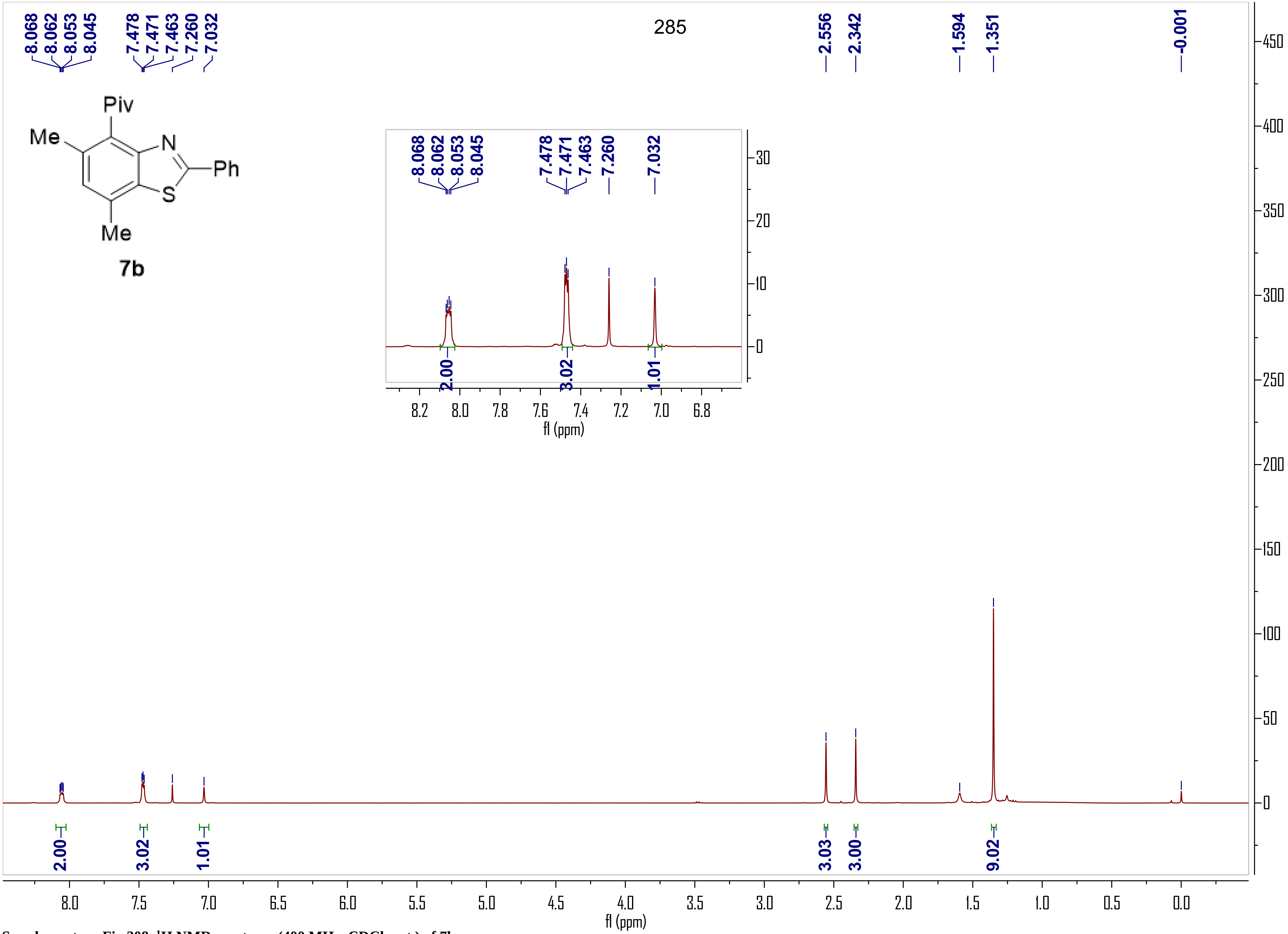

Supplementary Fig 208. **^{1}H NMR spectrum (400 MHz, $CDCl_3$, r.t.) of 7b.**

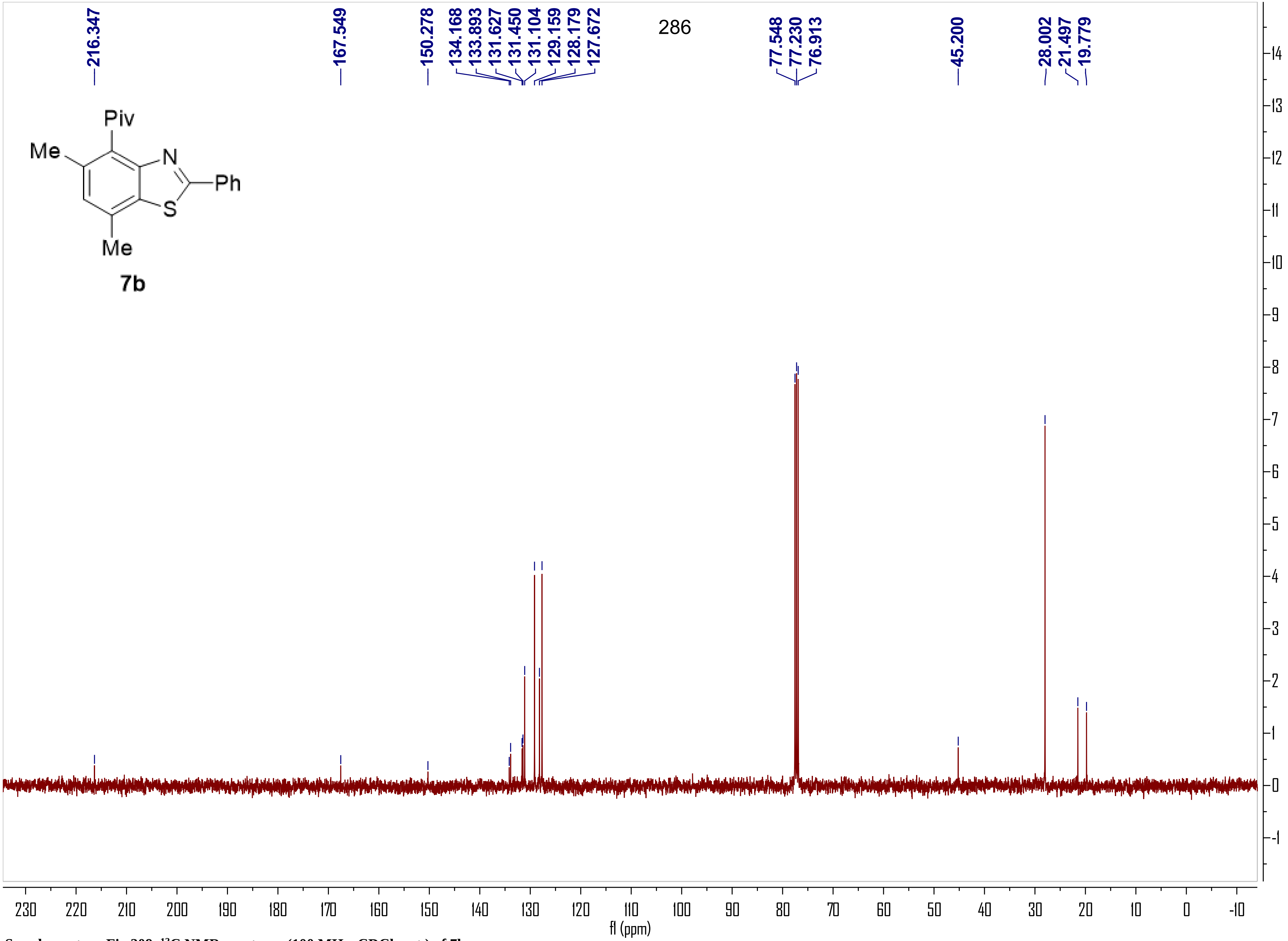

Supplementary Fig 209. ^{13}**C NMR spectrum (100 MHz, CDCl$_3$, r.t.) of 7b.**

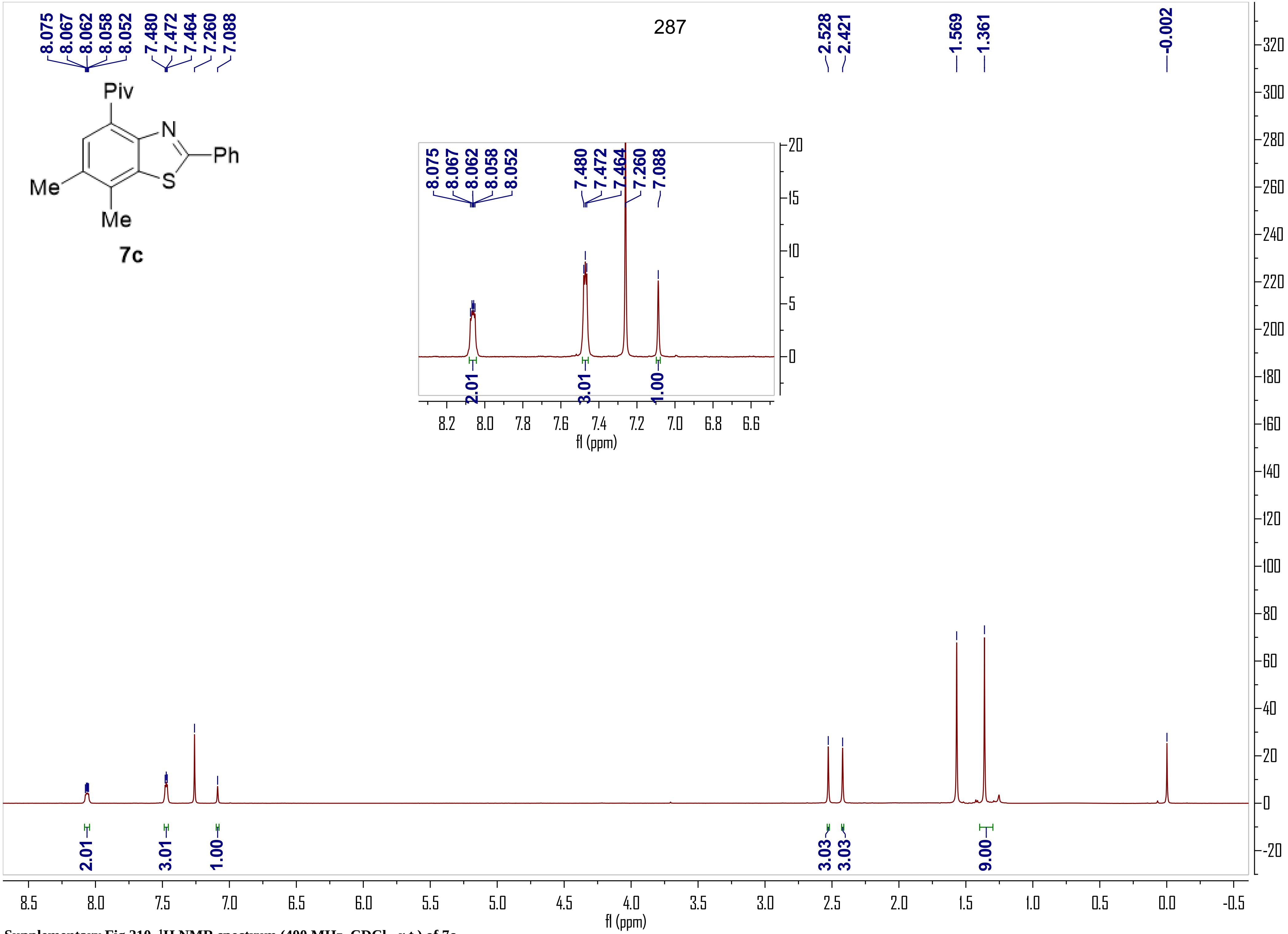

Supplementary Fig 210. ¹H NMR spectrum (400 MHz, $CDCl_3$, r.t.) of 7c.

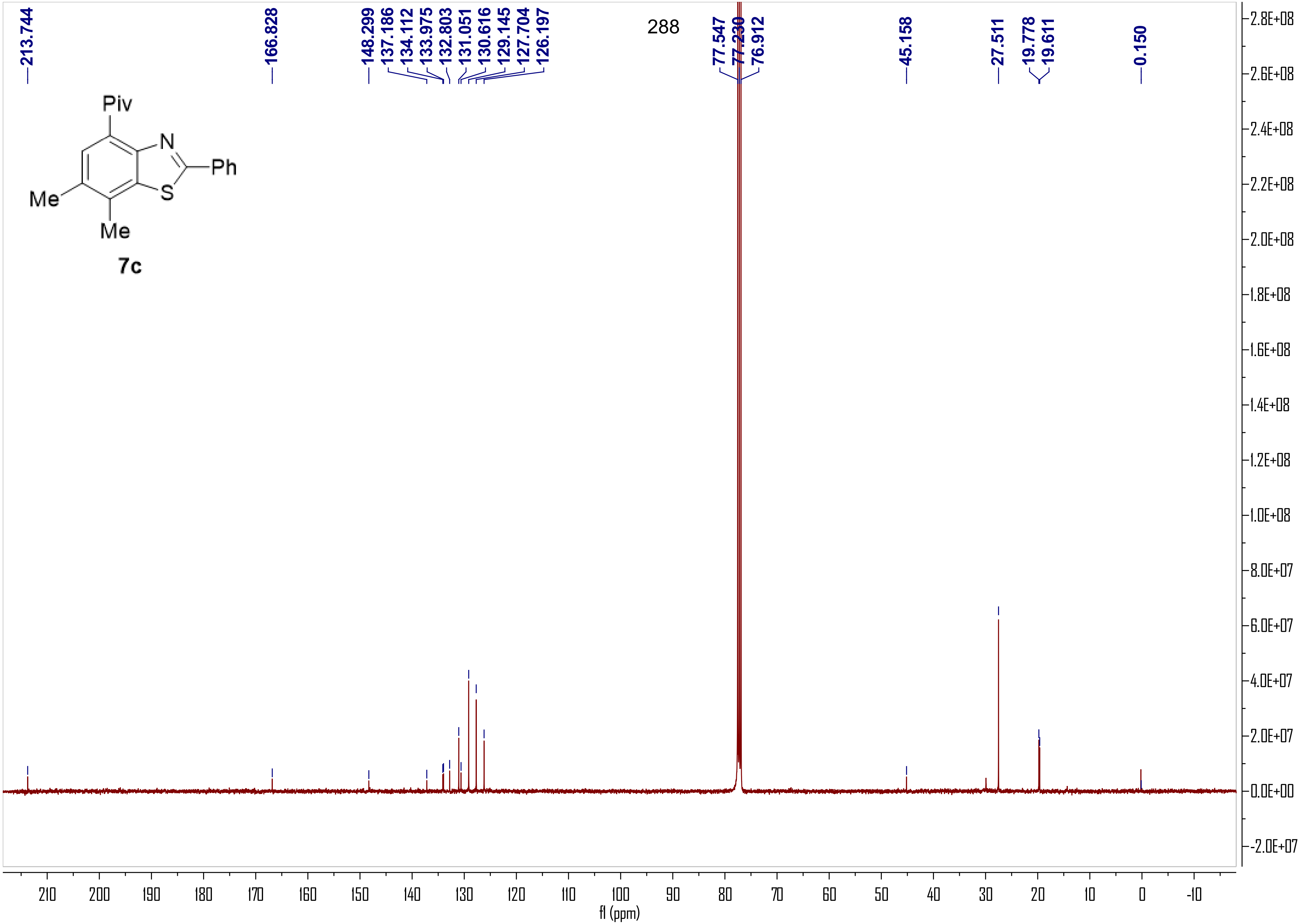

Supplementary Fig 211. **^{13}C NMR spectrum (100 MHz, $CDCl_3$, r.t.) of 7c.**

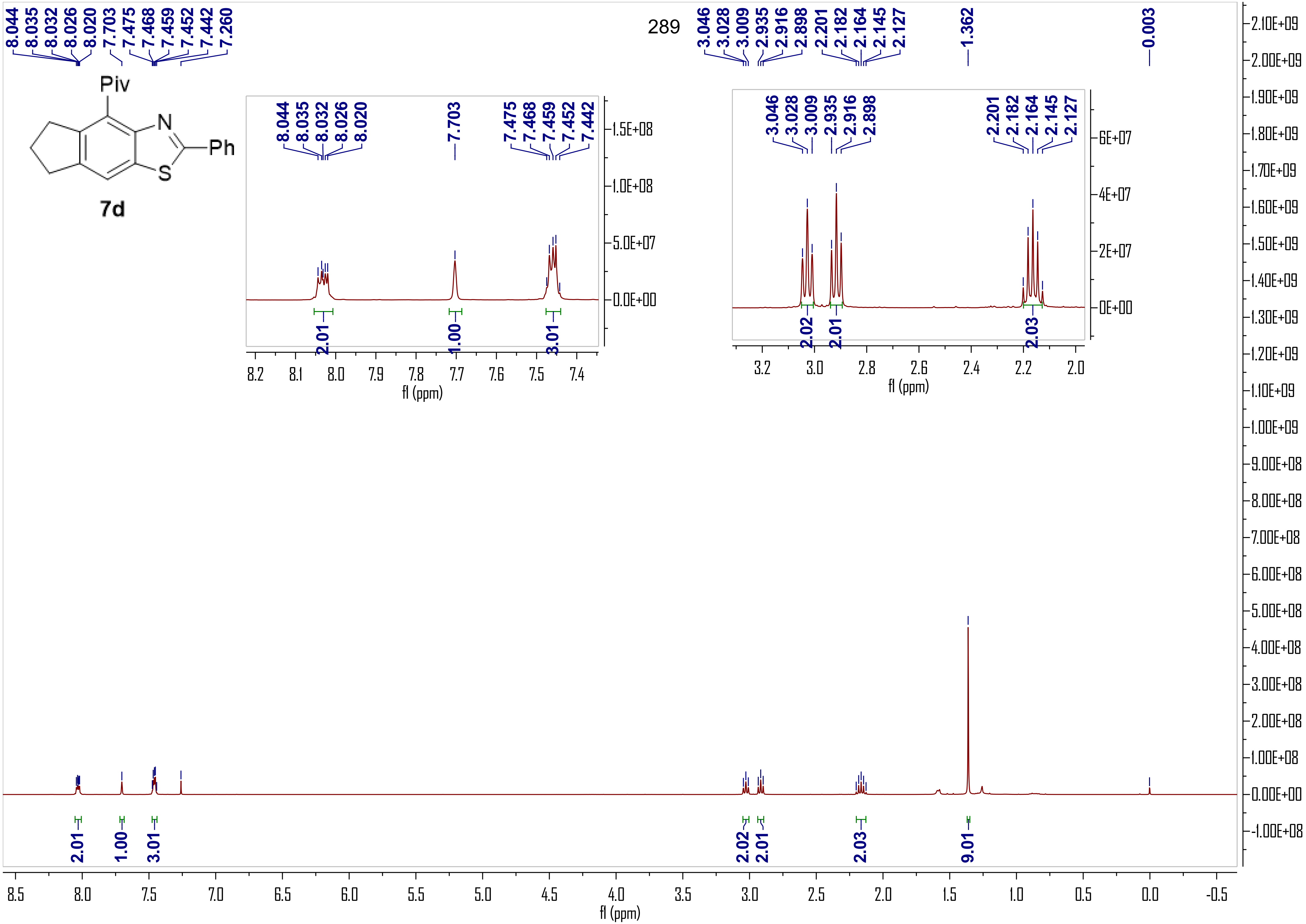

Supplementary Fig 212. ^{1}H NMR spectrum (400 MHz, $CDCl_3$, r.t.) of 7d.

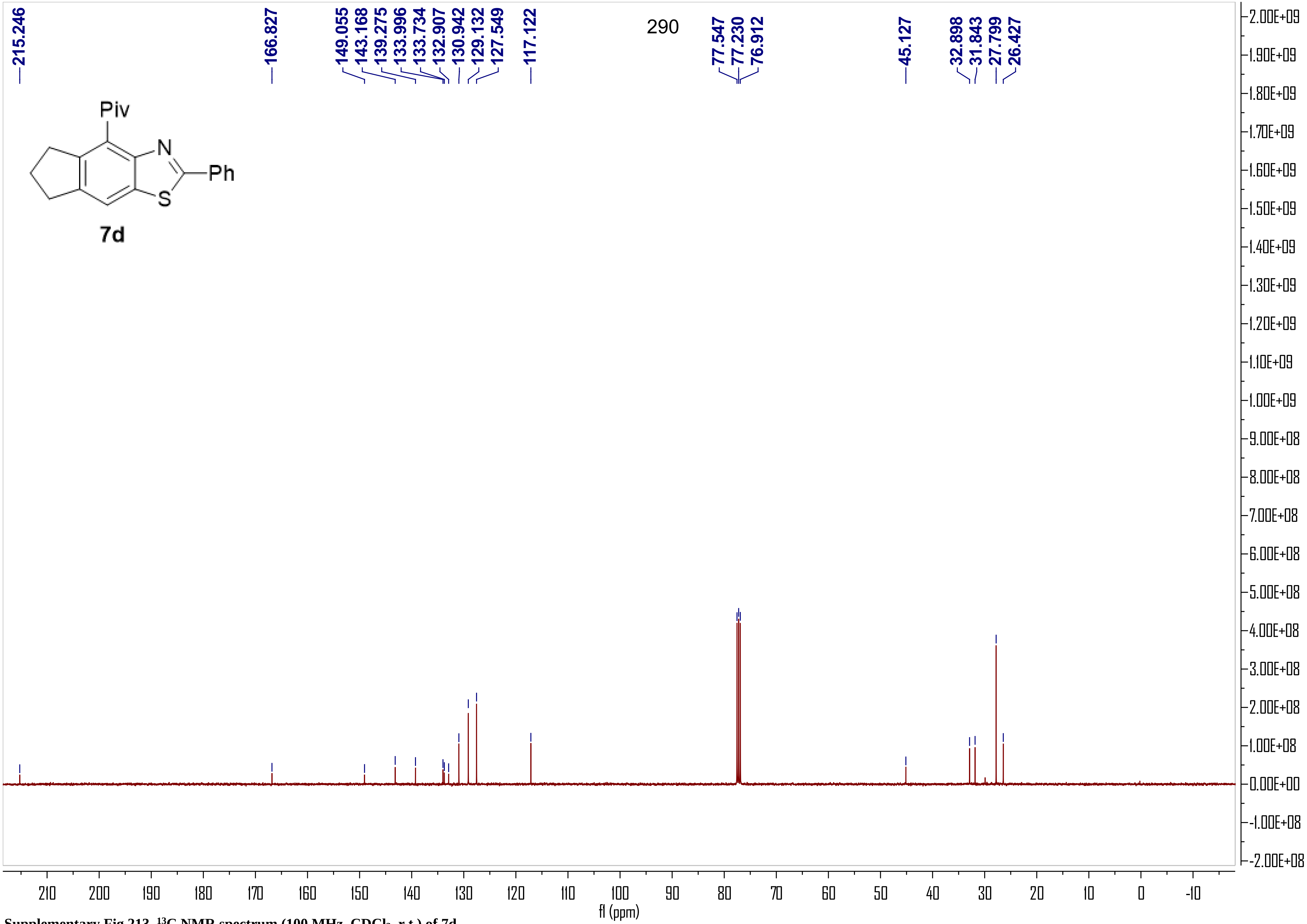

Supplementary Fig 213. **^{13}C NMR spectrum (100 MHz, $CDCl_3$, r.t.) of 7d.**

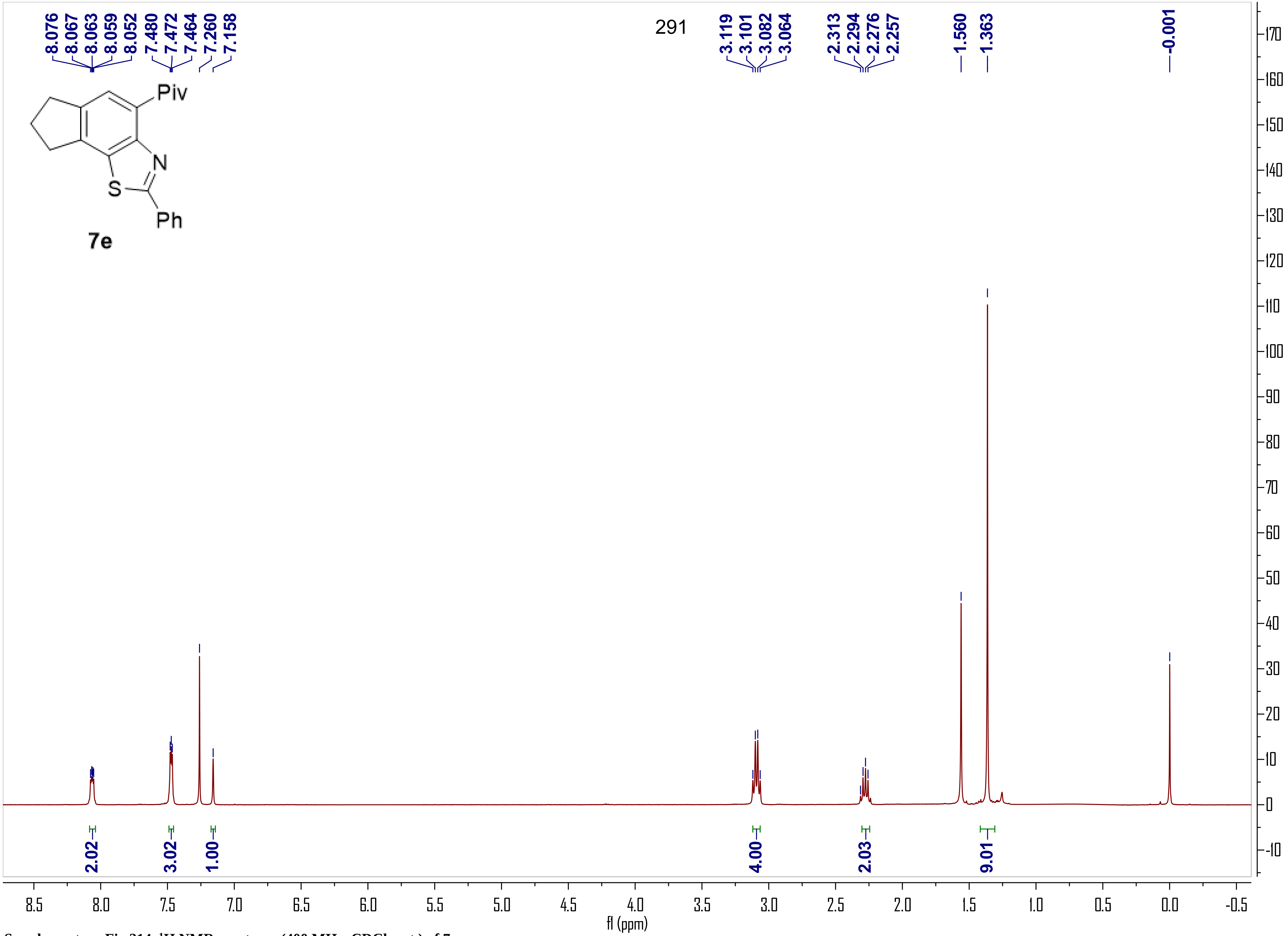

Supplementary Fig 214. **^{1}H NMR spectrum (400 MHz, $CDCl_3$, r.t.) of 7e.**

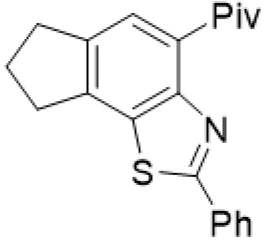

7e

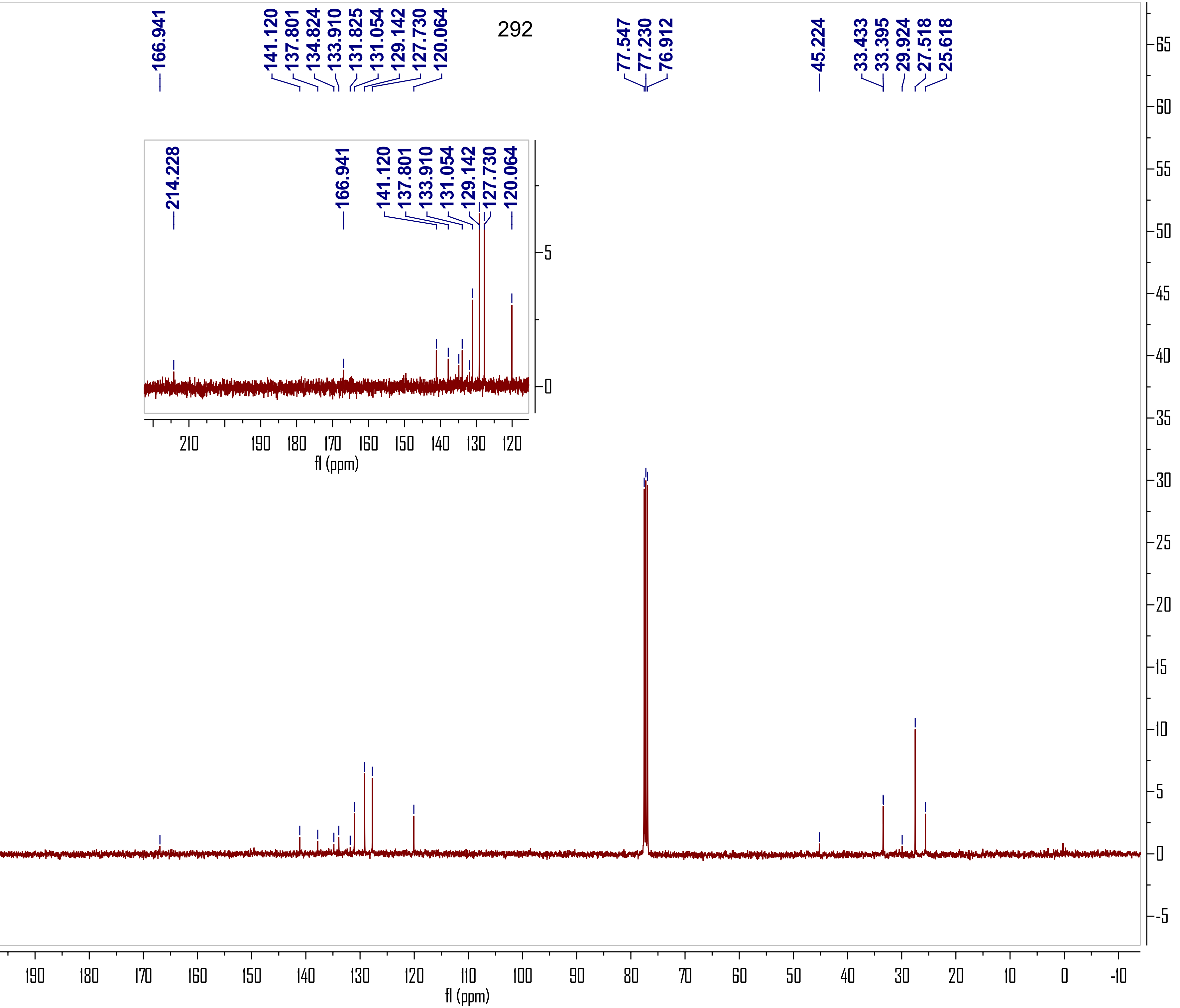

Supplementary Fig 215. ^{13}C NMR spectrum (100 MHz, $CDCl_3$, r.t.) of 7e.

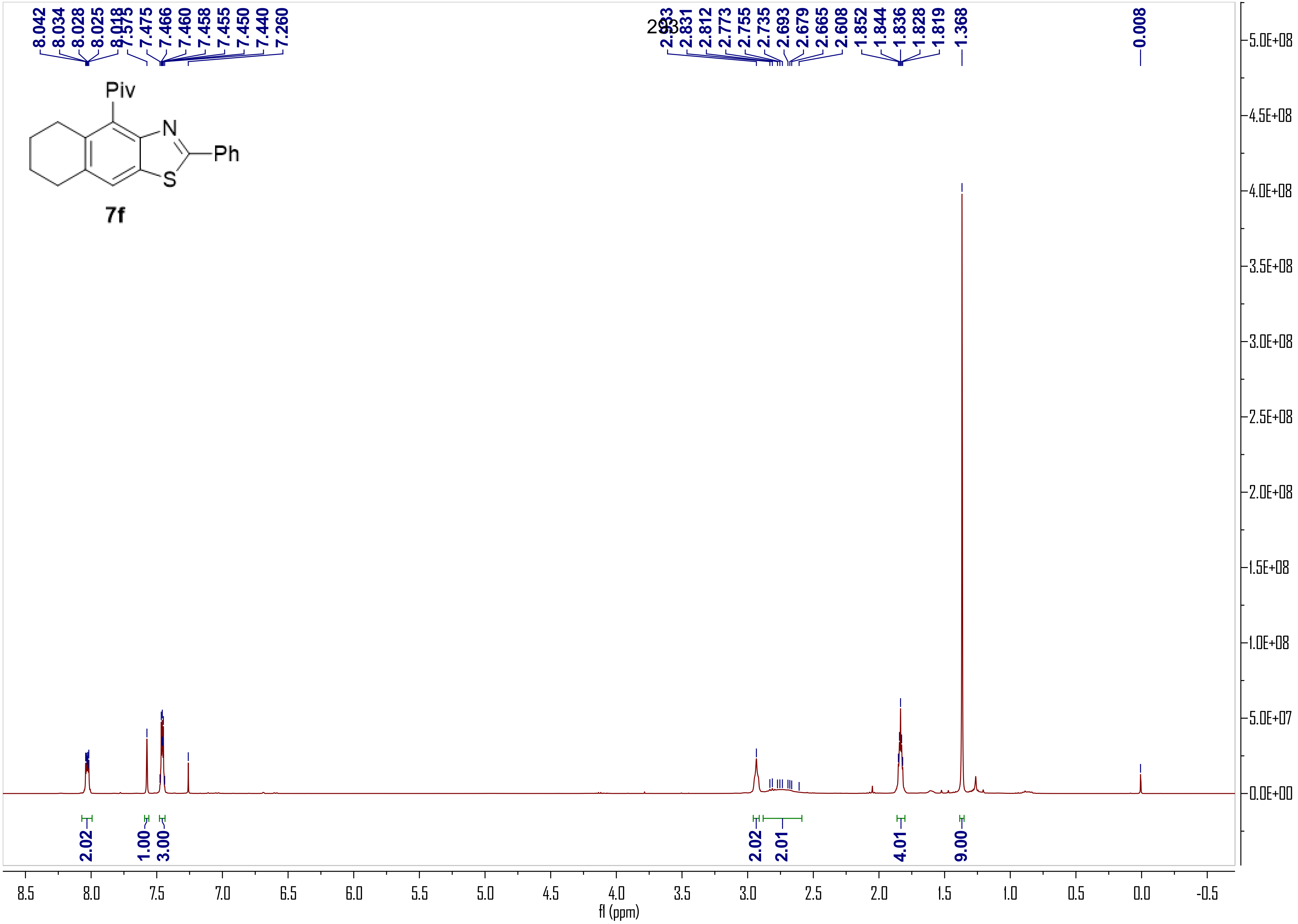

Supplementary Fig 216. ^{1}H NMR spectrum (400 MHz, $CDCl_3$, r.t.) of 7f.

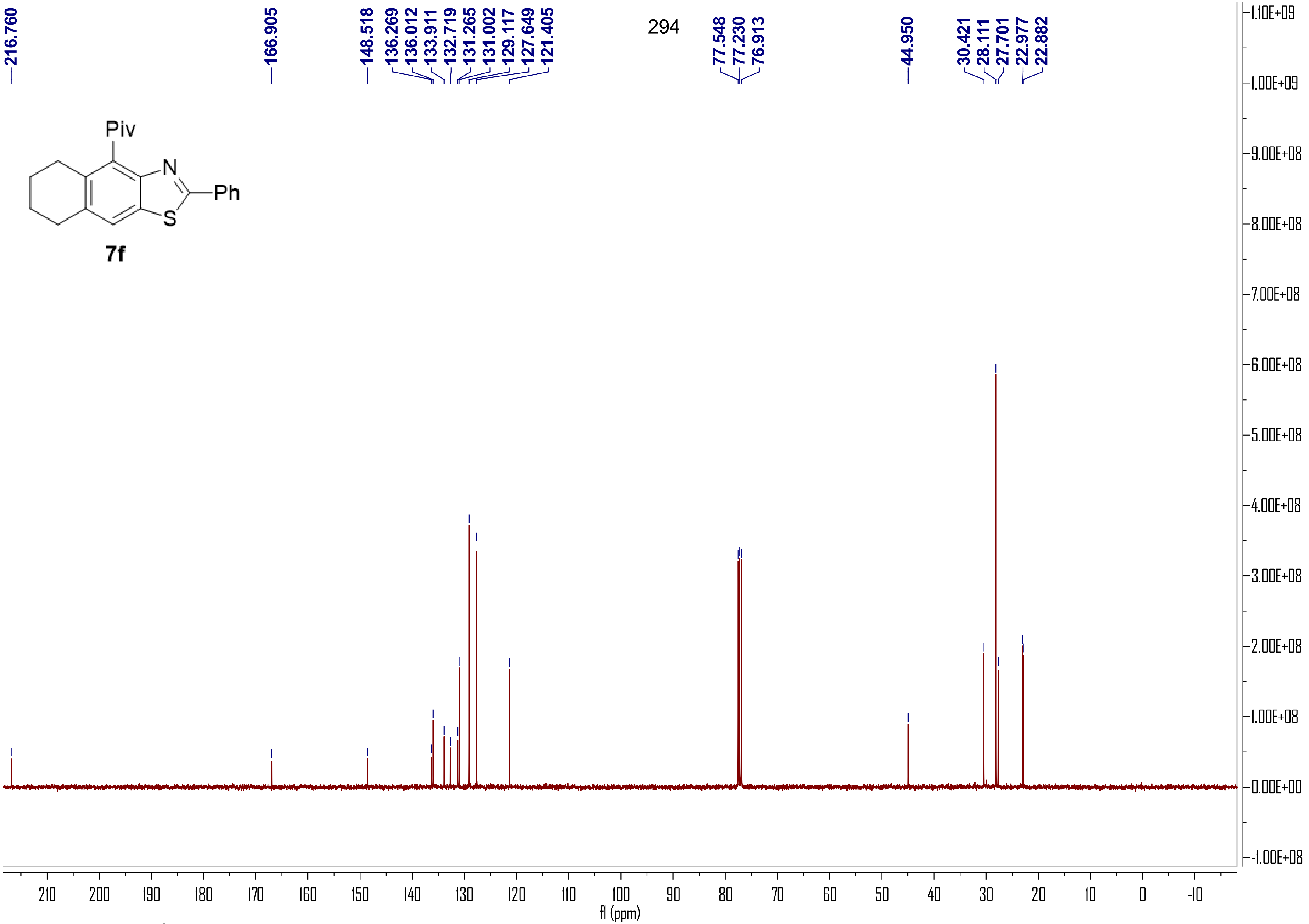

Supplementary Fig 217. ^{13}C **NMR spectrum (100 MHz, $CDCl_3$, r.t.) of 7f.**

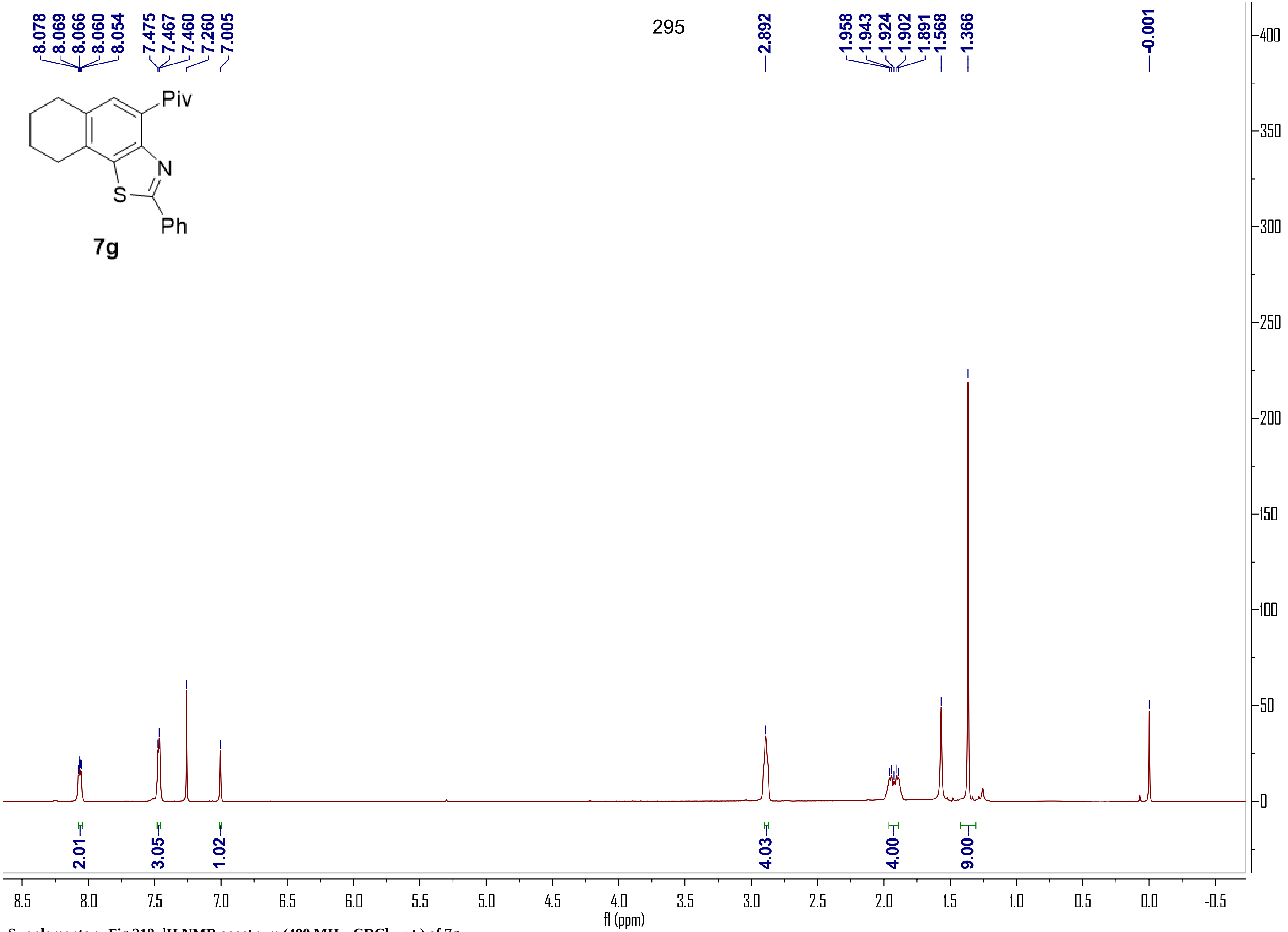

Supplementary Fig 218. ^{1}H NMR spectrum (400 MHz, $CDCl_3$, r.t.) of 7g.

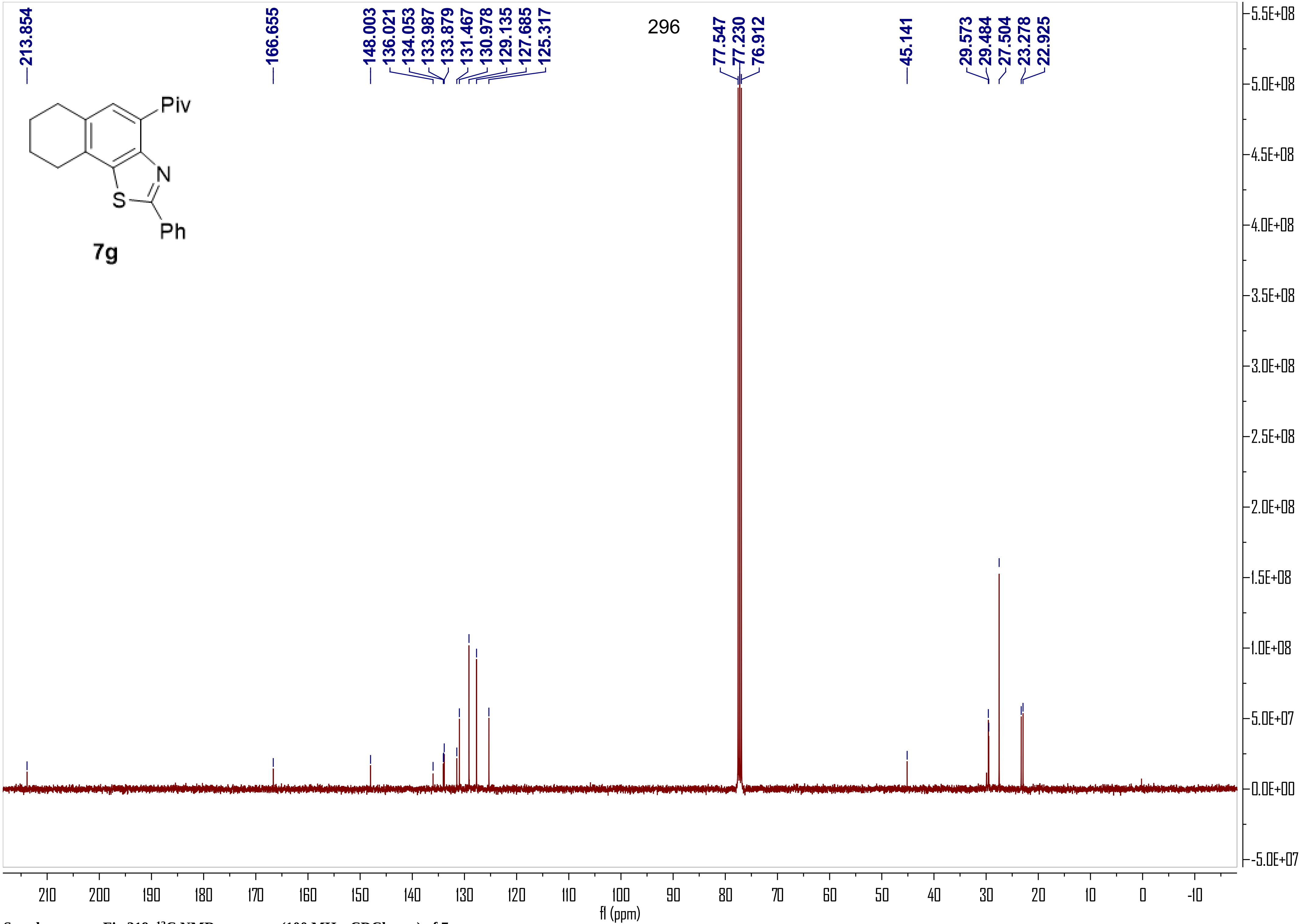

Supplementary Fig 219. ^{13}C **NMR spectrum (100 MHz, CDCl$_3$, r.t.) of 7g.**

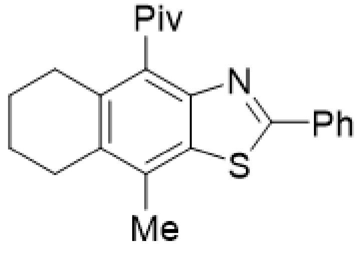

7h

297

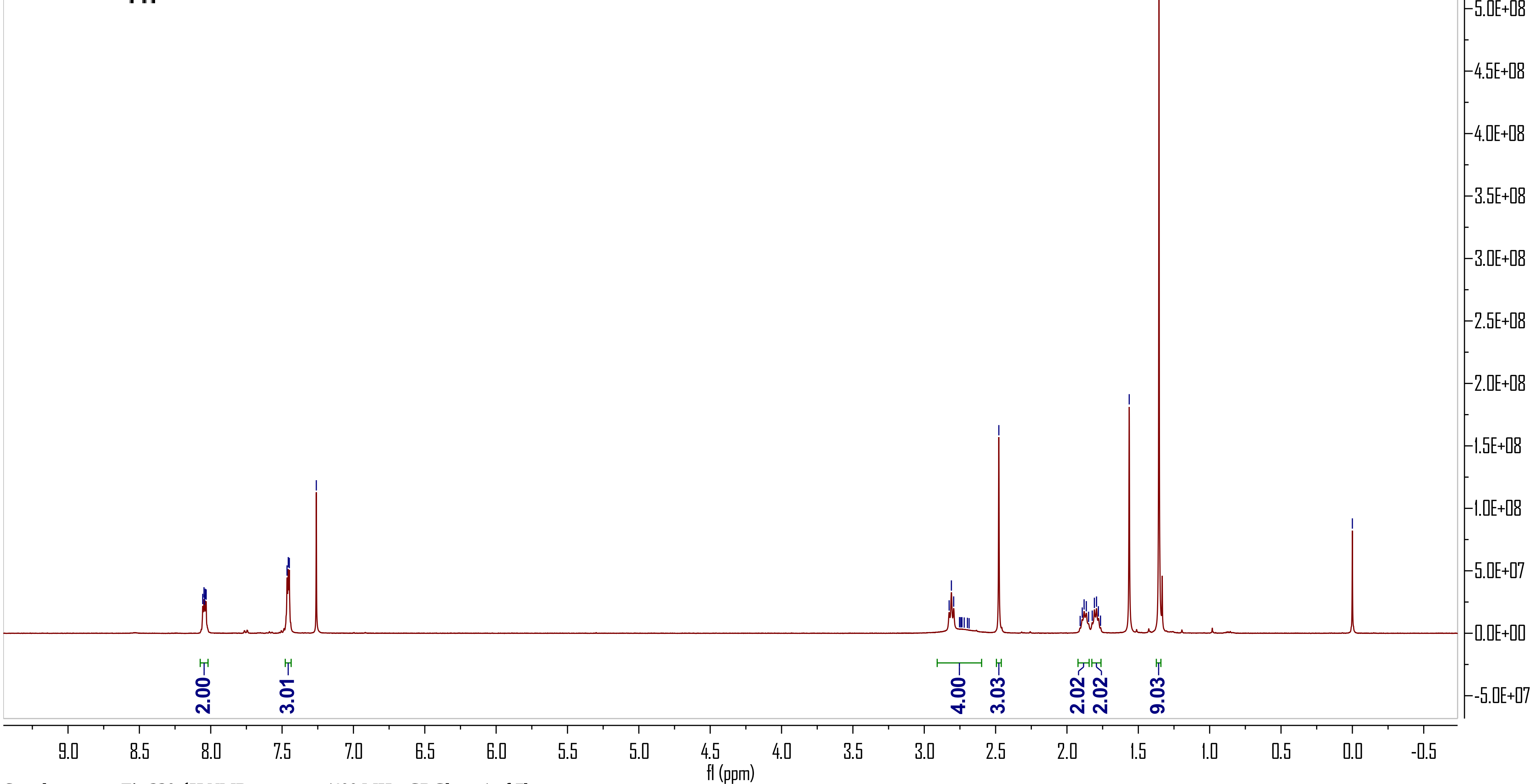

Supplementary Fig 220. ^{1}H NMR spectrum (400 MHz, $CDCl_3$, r.t.) of 7h.

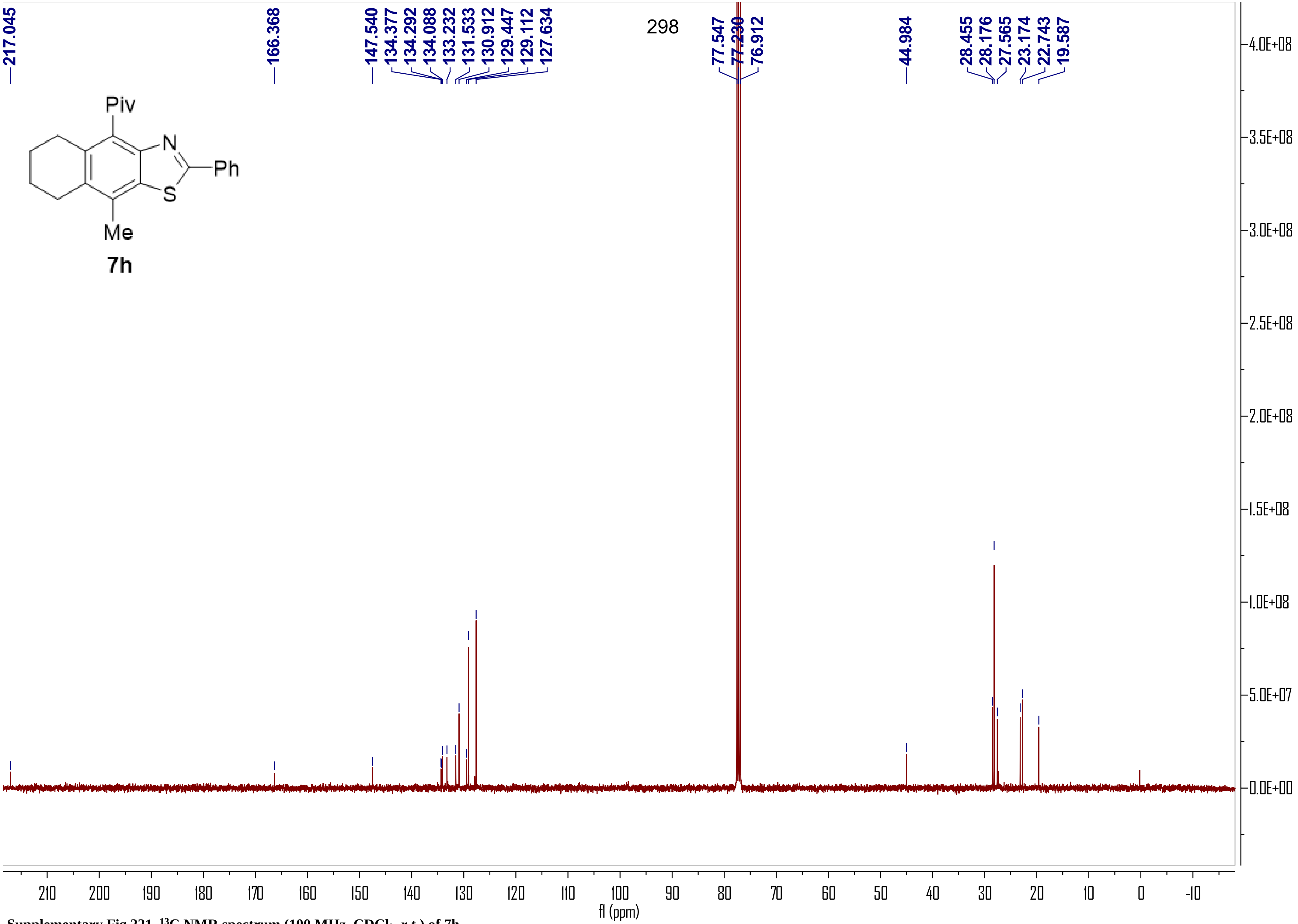

Supplementary Fig 221. ^{13}C NMR spectrum (100 MHz, $CDCl_3$, r.t.) of 7h.

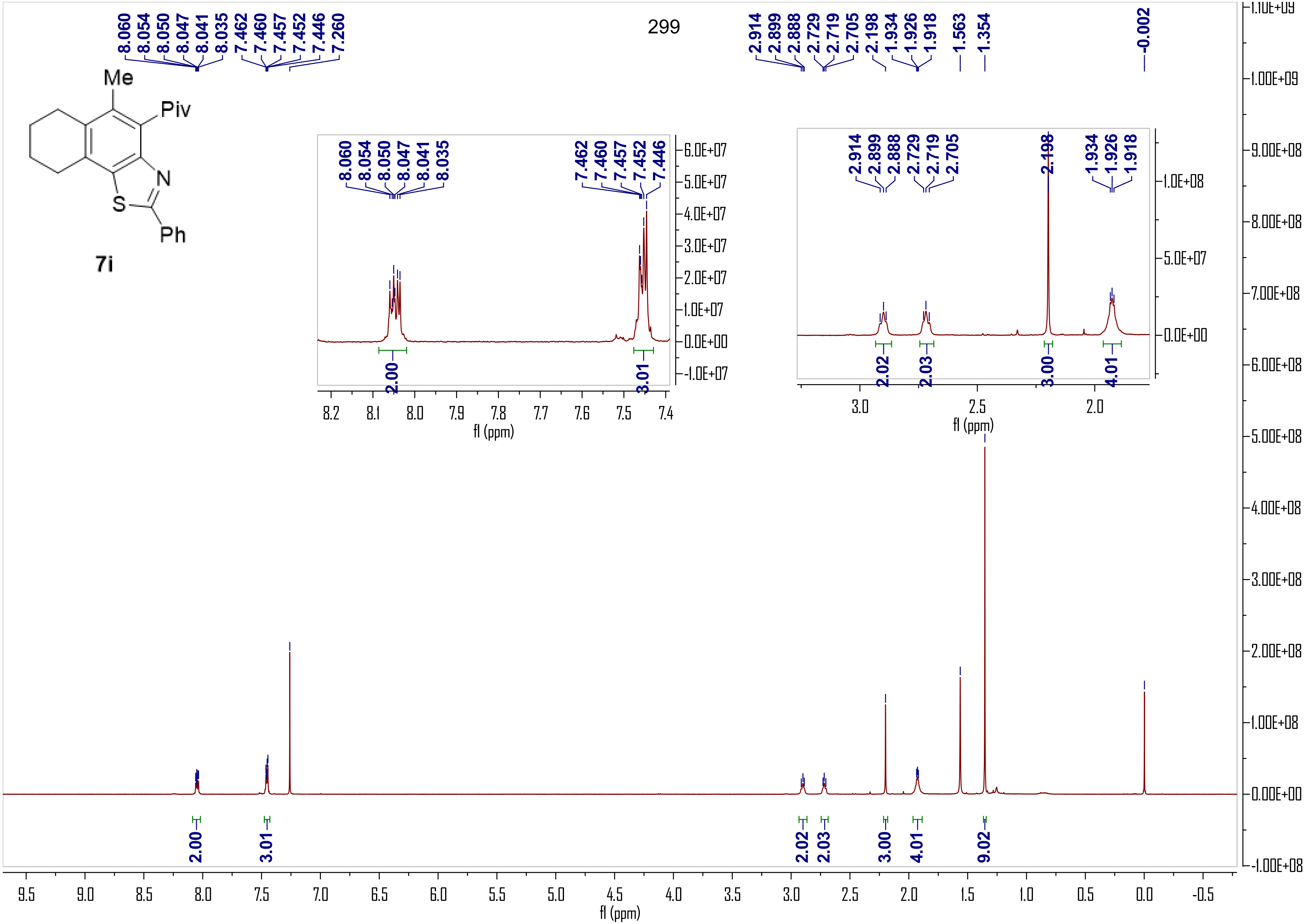

Supplementary Fig 222. **^{1}H NMR spectrum (400 MHz, $CDCl_3$, r.t.) of 7i.**

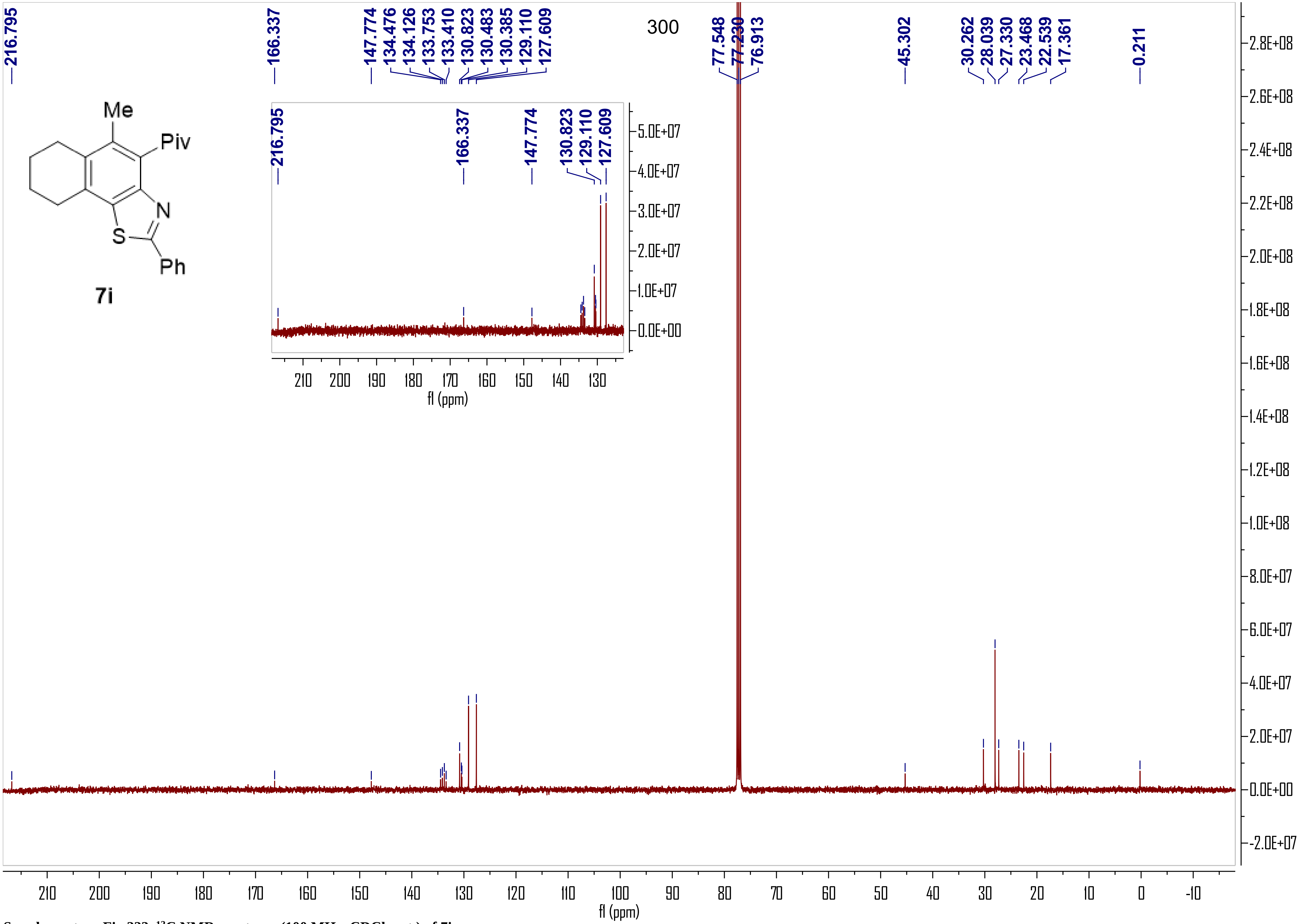

Supplementary Fig 223. **^{13}C NMR spectrum (100 MHz, $CDCl_3$, r.t.) of 7i.**

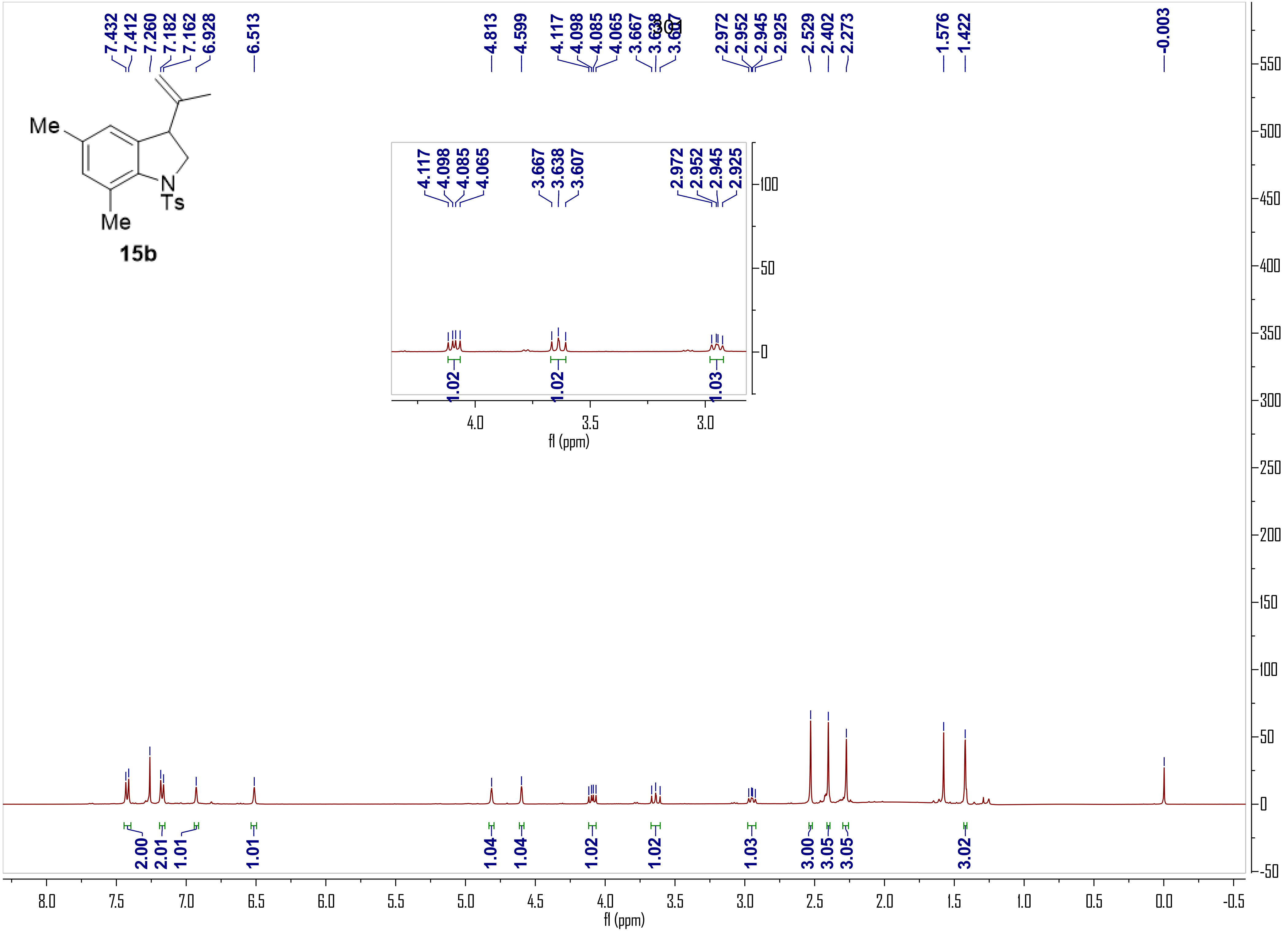

Supplementary Fig 224. **^{1}H NMR spectrum (400 MHz, $CDCl_3$, r.t.) of 15b.**

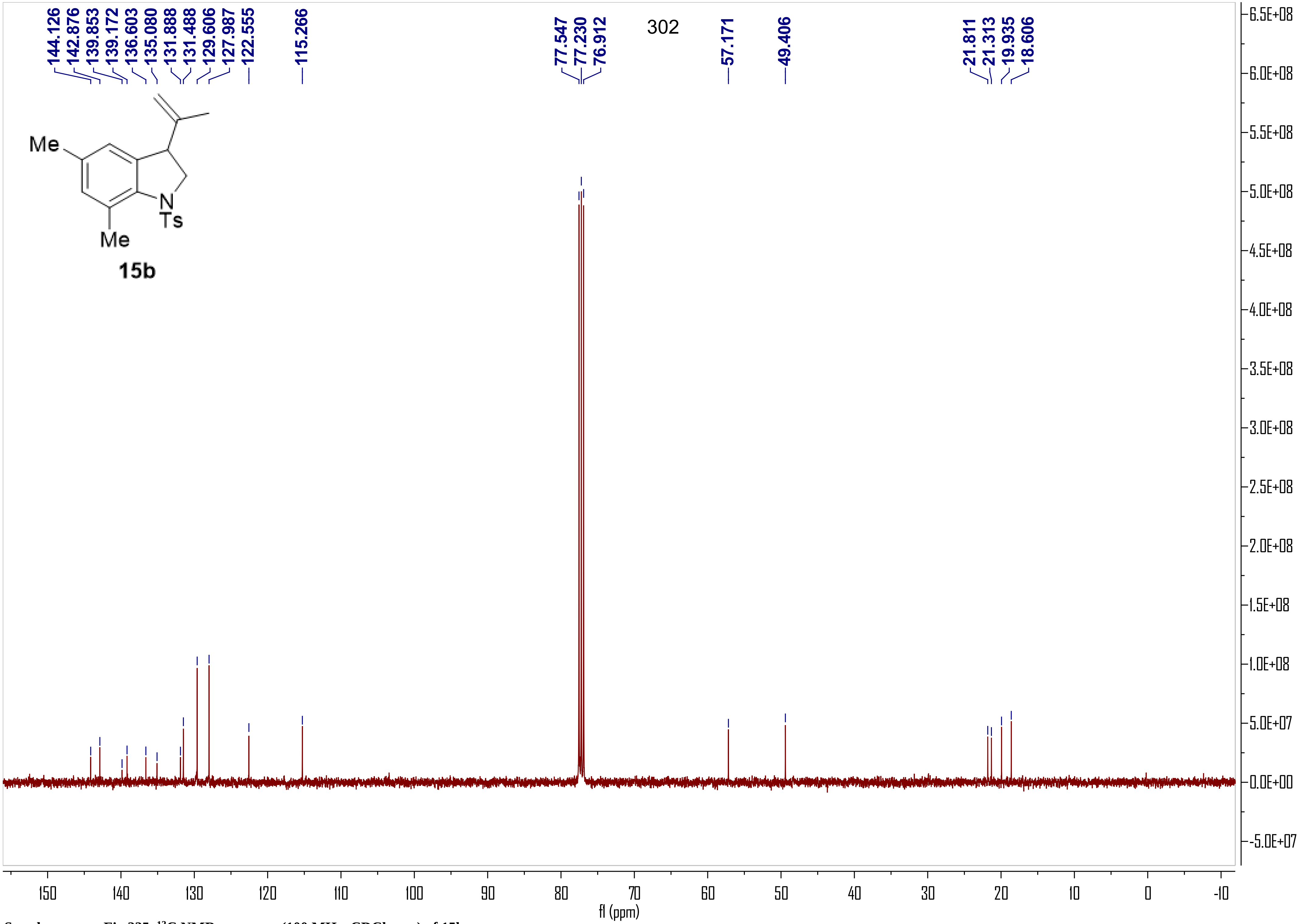

Supplementary Fig 225. **^{13}C NMR spectrum (100 MHz, $CDCl_3$, r.t.) of 15b.**

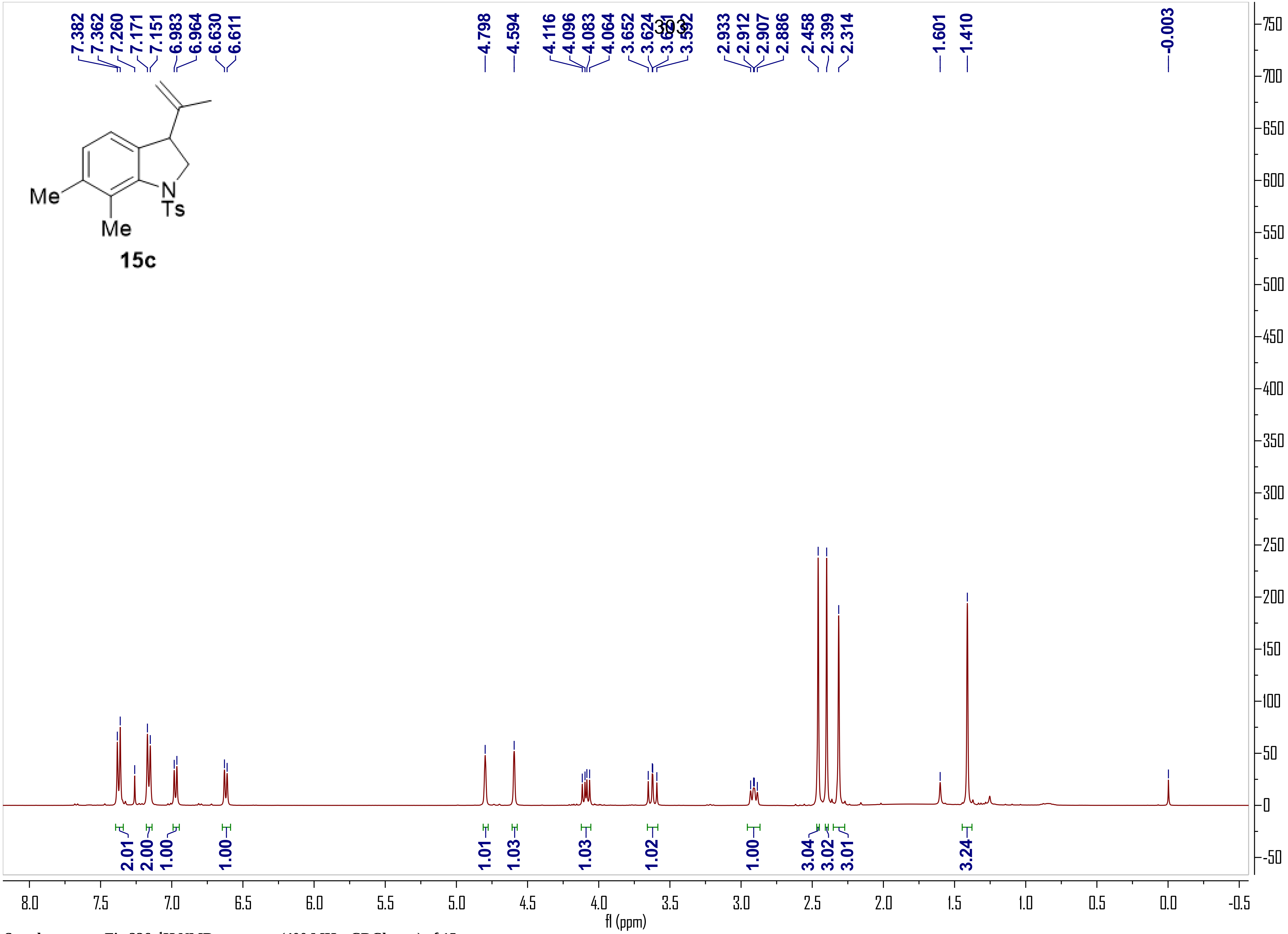

Supplementary Fig 226. ^{1}**H NMR spectrum (400 MHz,** $CDCl_3$**, r.t.) of 15c.**

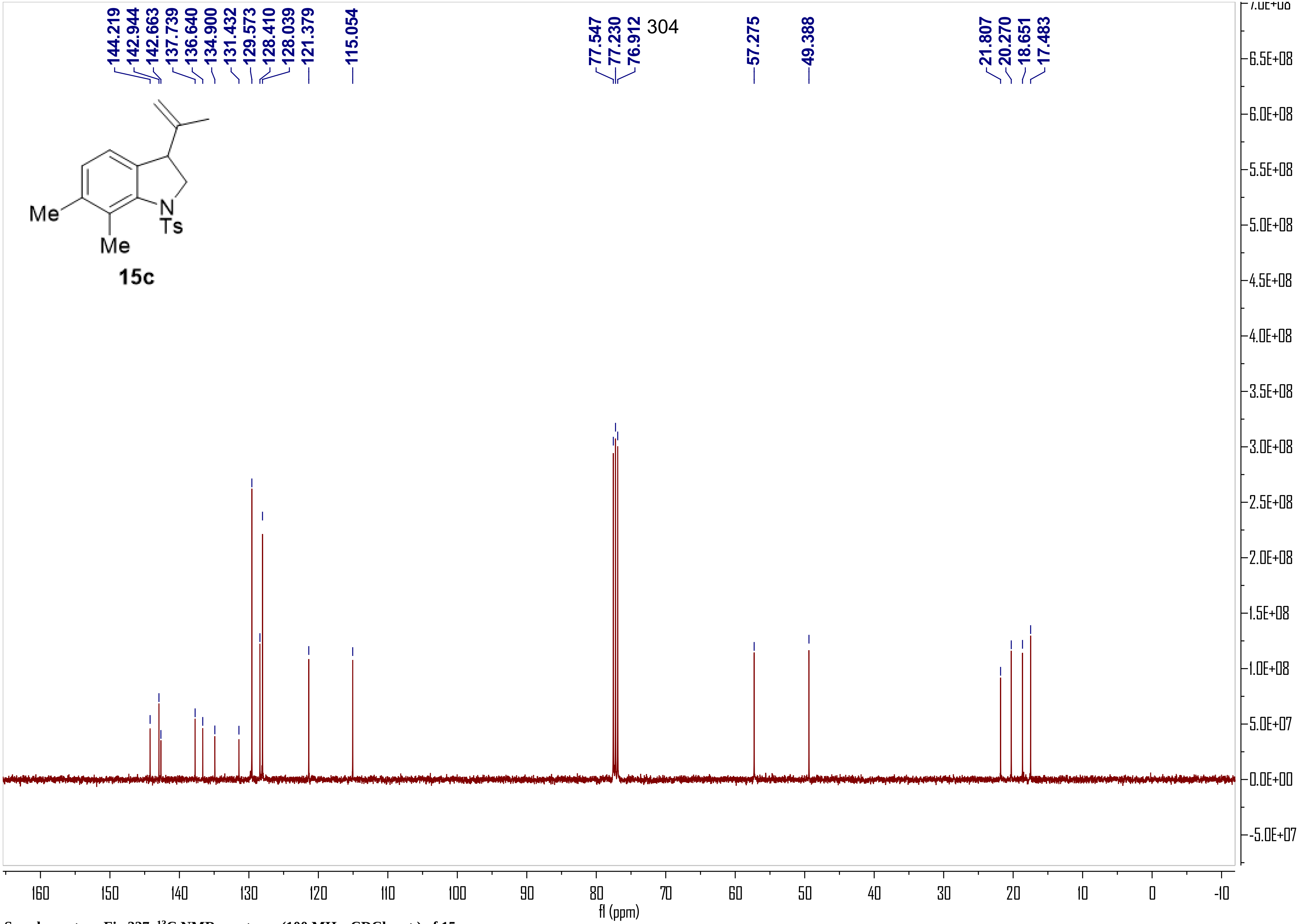

Supplementary Fig 227. ^{13}C **NMR spectrum (100 MHz,** $CDCl_3$**, r.t.) of 15c.**

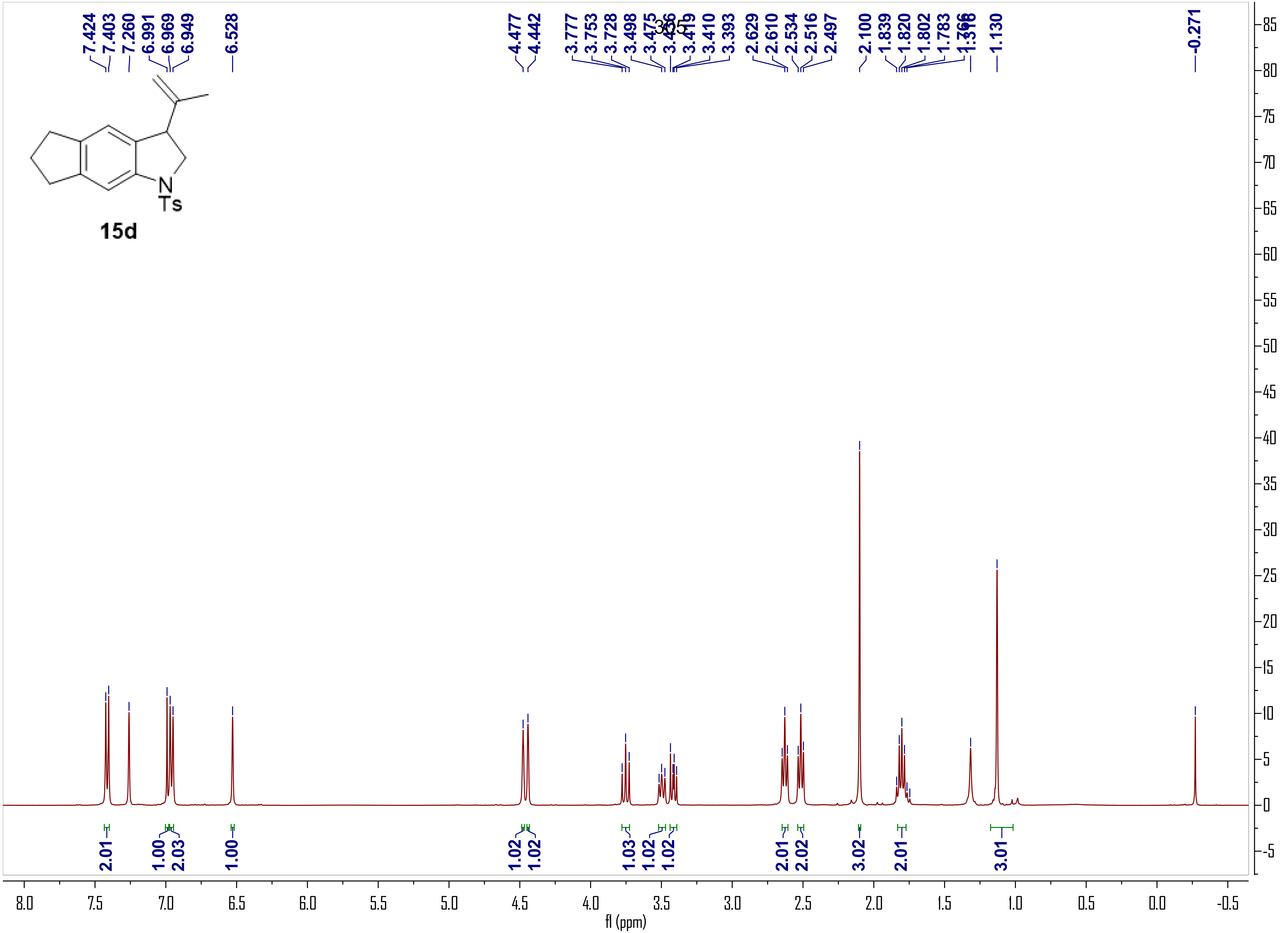

Supplementary Fig 228. **^{1}H NMR spectrum (400 MHz, $CDCl_3$, r.t.) of 15d.**

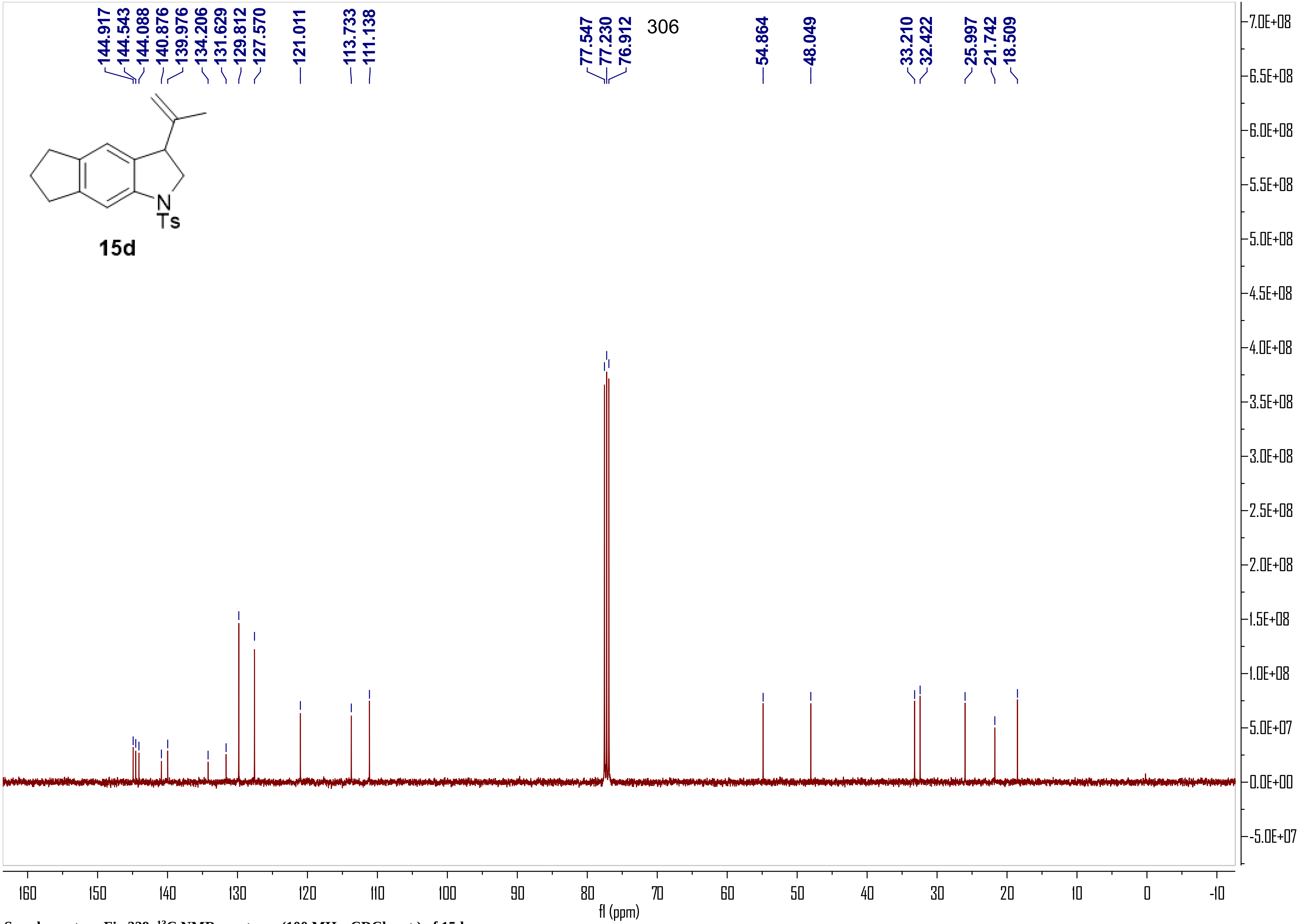

Supplementary Fig 229. **^{13}C NMR spectrum (100 MHz, $CDCl_3$, r.t.) of 15d.**

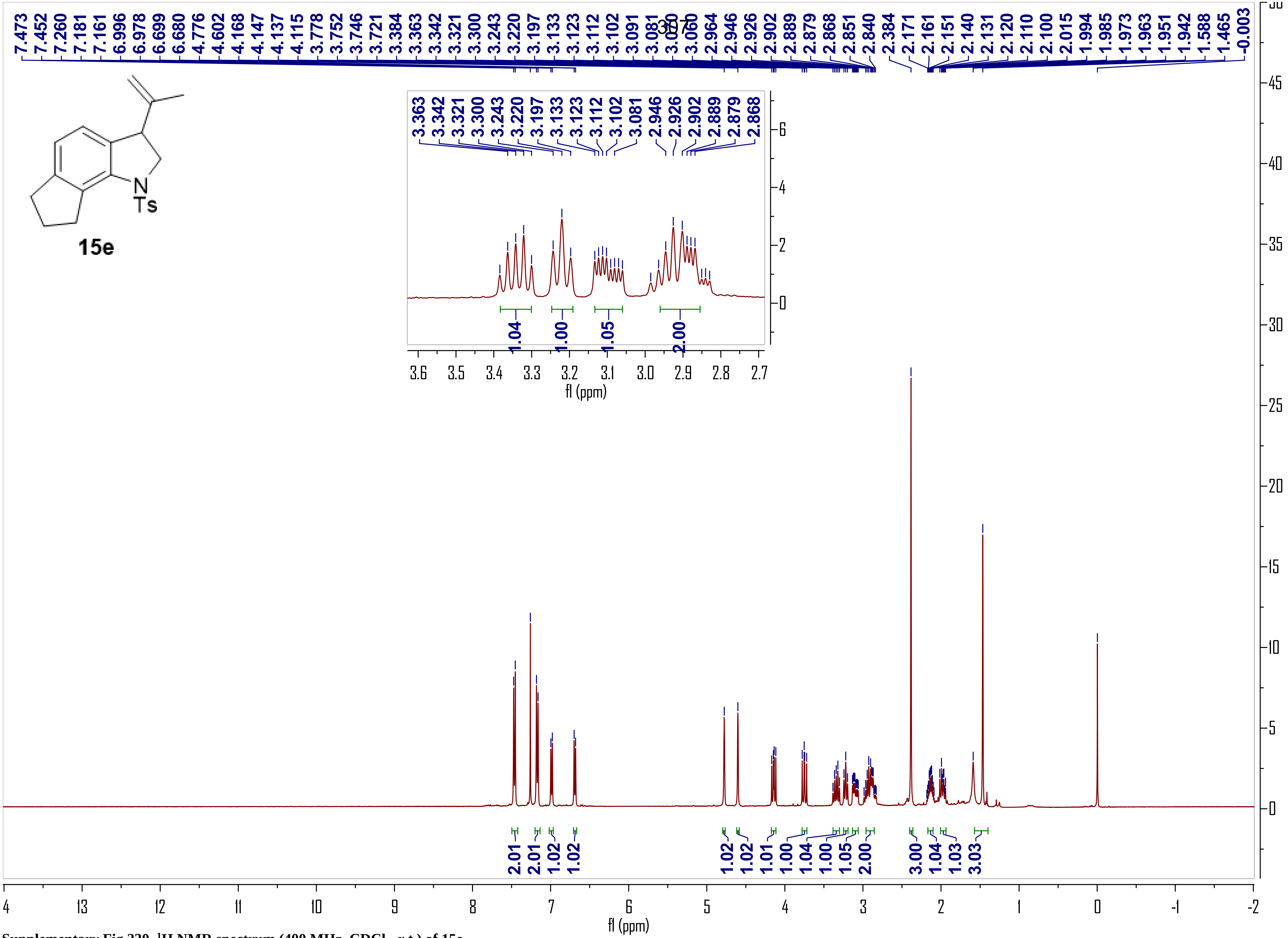

Supplementary Fig 230. ^{1}H NMR spectrum (400 MHz, $CDCl_3$, r.t.) of 15e.

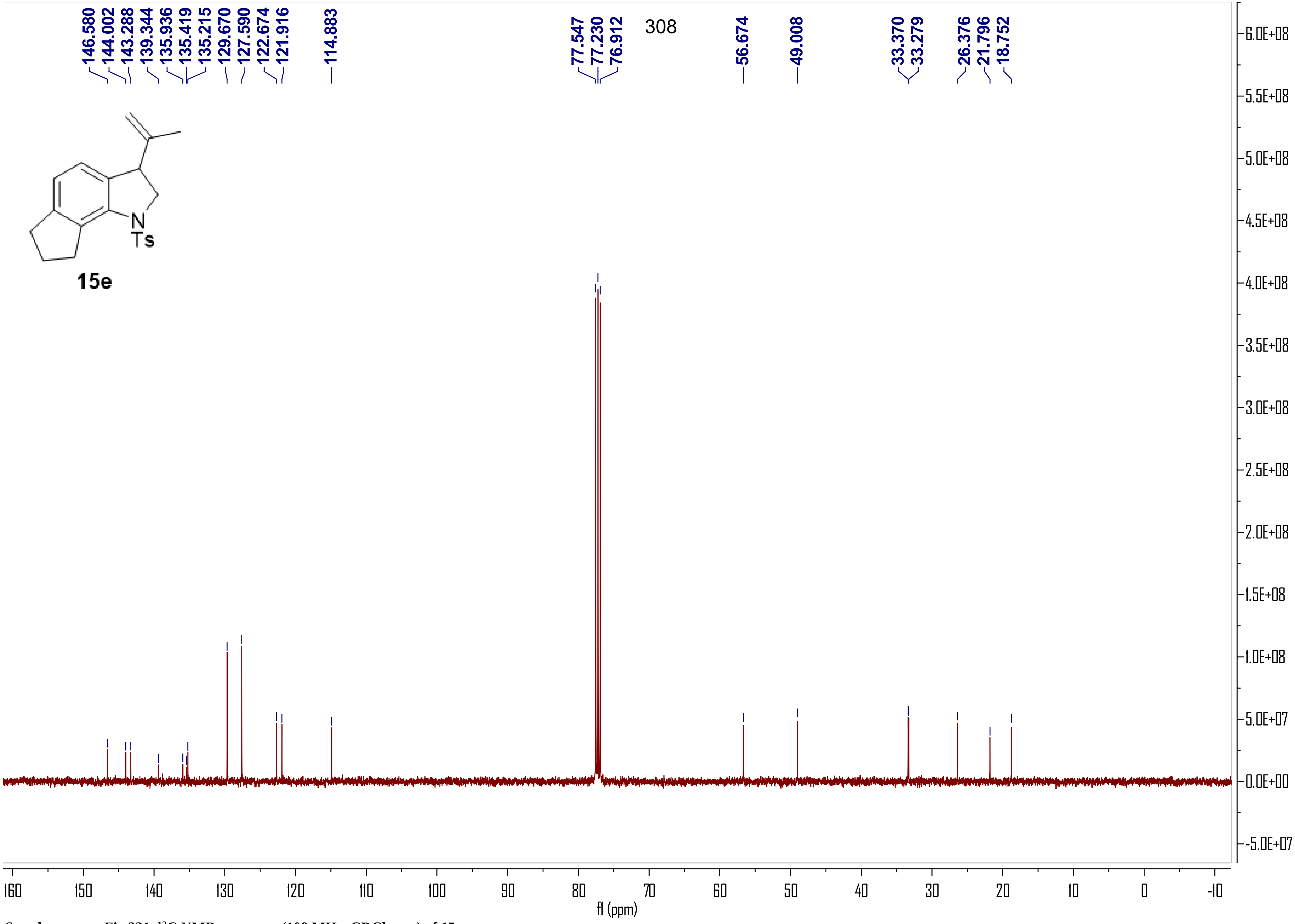

Supplementary Fig 231. **^{13}C NMR spectrum (100 MHz, $CDCl_3$, r.t.) of 15e.**

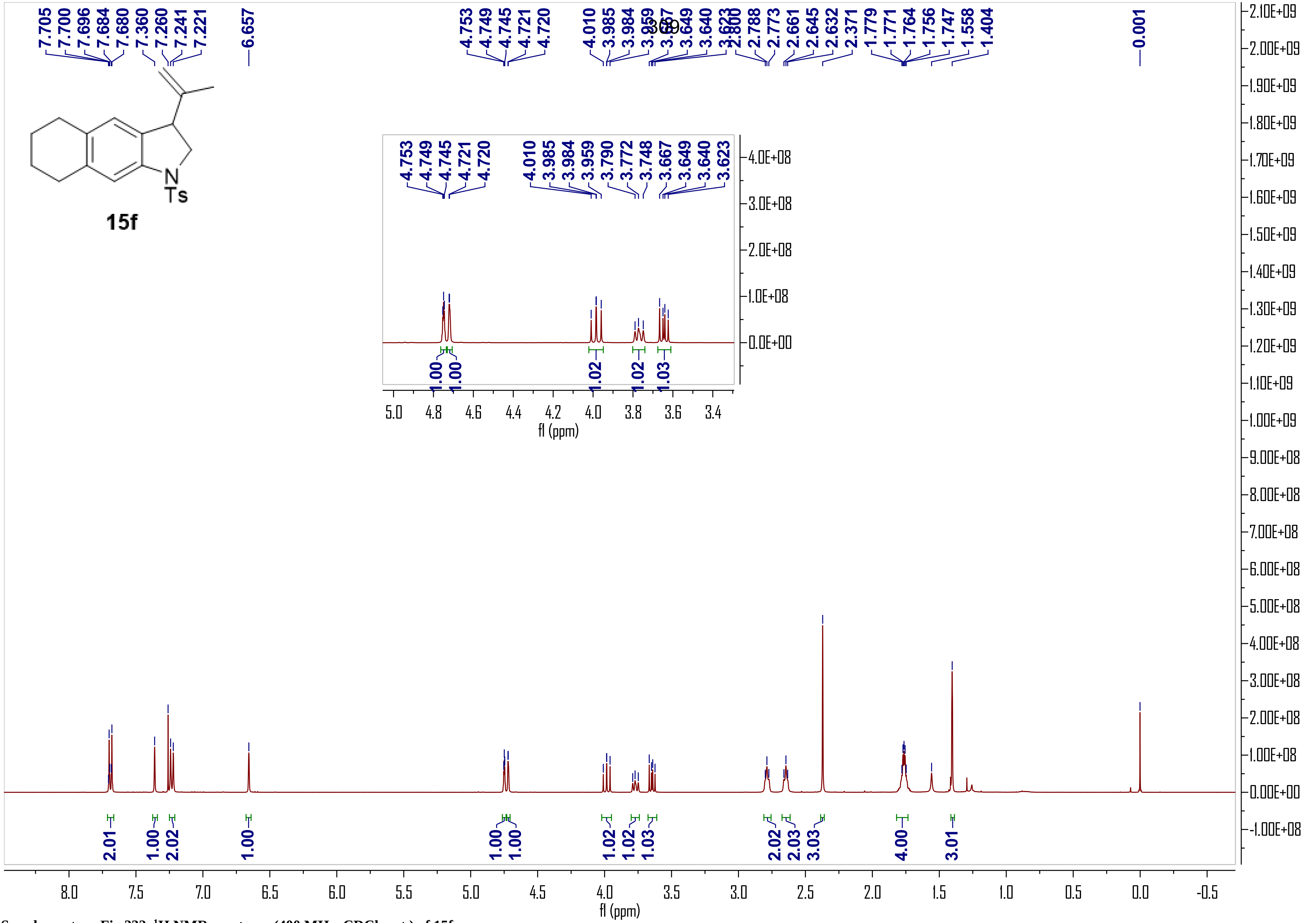

Supplementary Fig 232. ^{1}H NMR spectrum (400 MHz, $CDCl_3$, r.t.) of 15f.

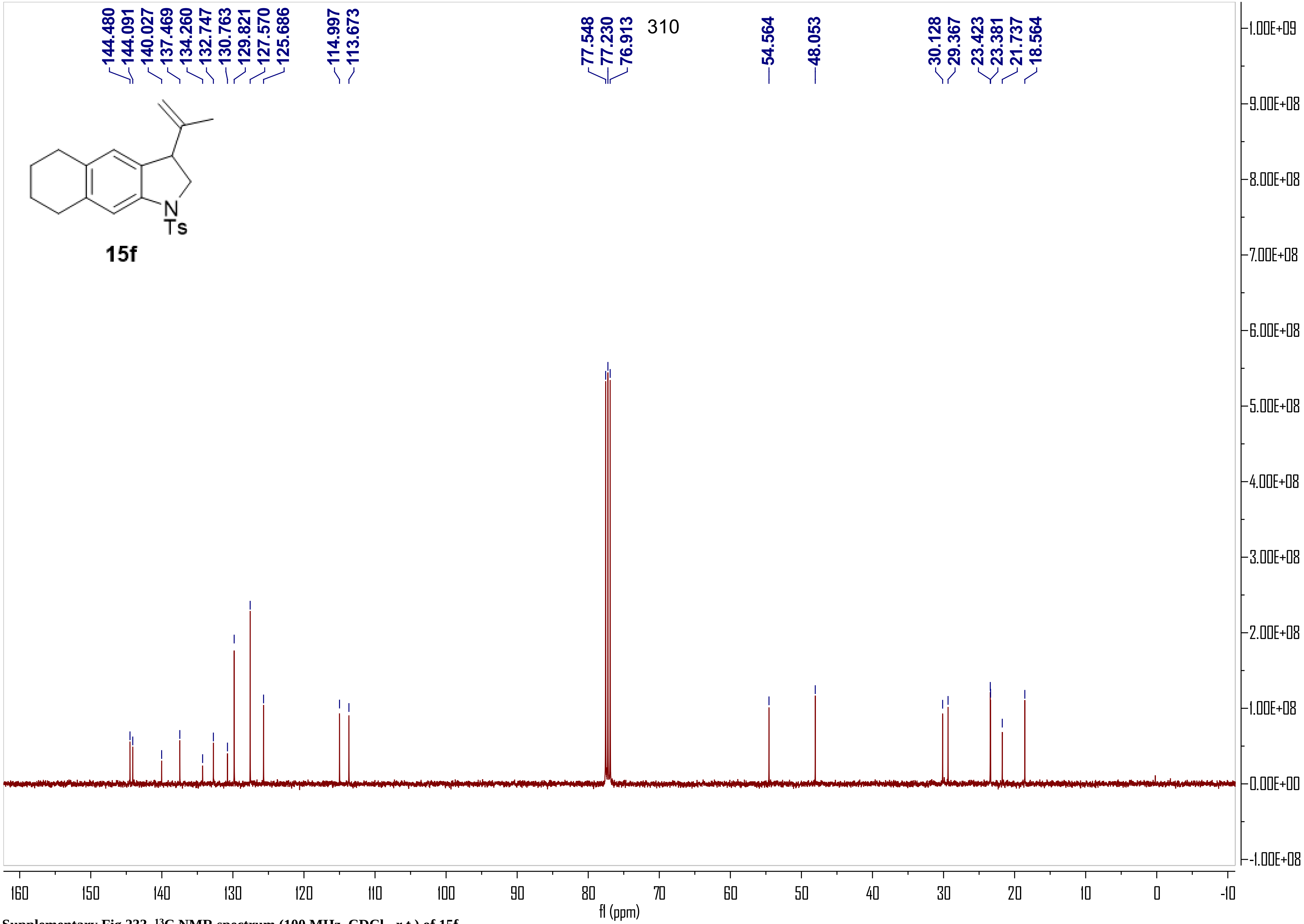

Supplementary Fig 233. **^{13}C NMR spectrum (100 MHz, $CDCl_3$, r.t.) of 15f.**

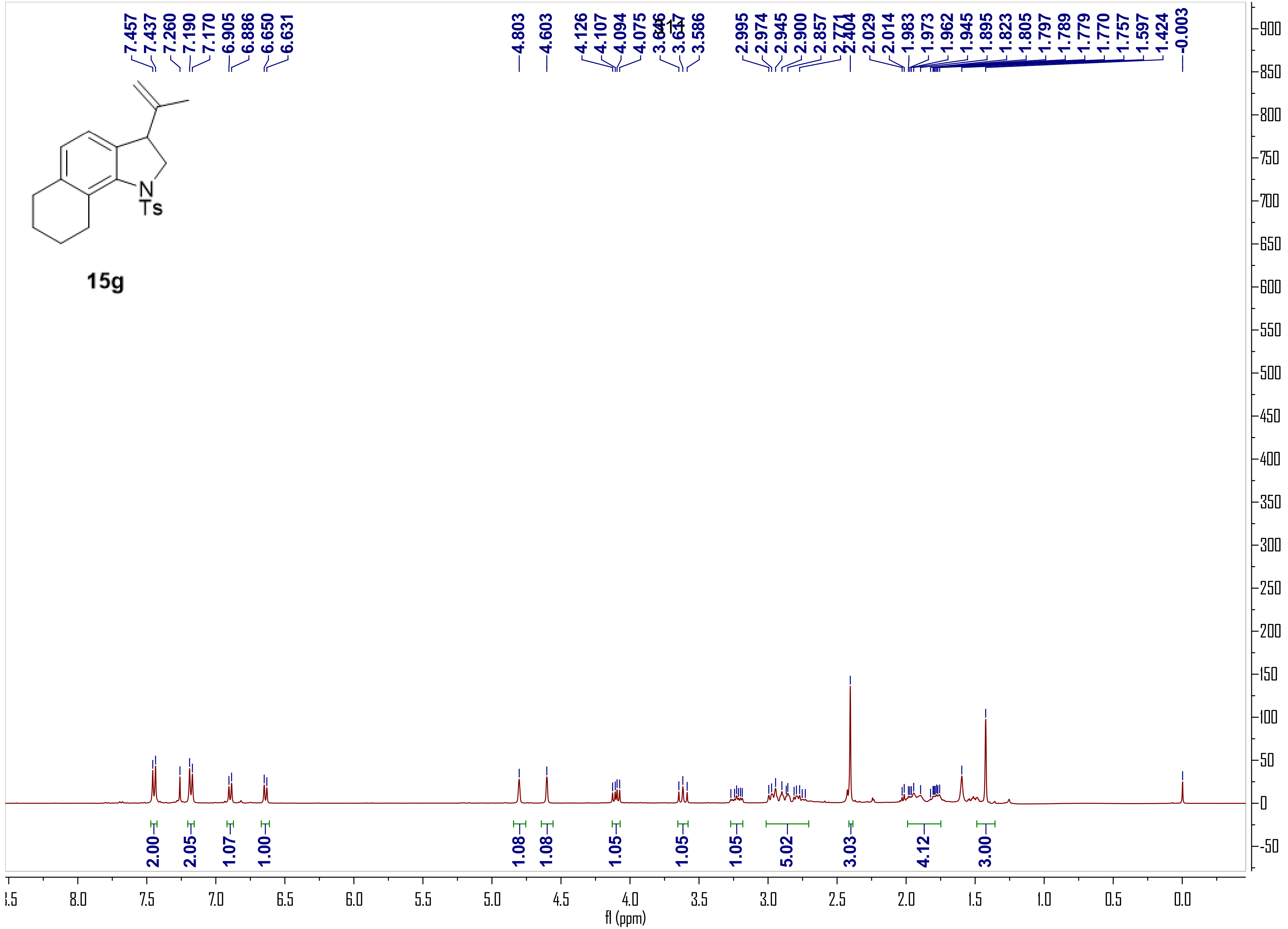

Supplementary Fig 234. ^1H NMR spectrum (400 MHz, $CDCl_3$, r.t.) of 15g.

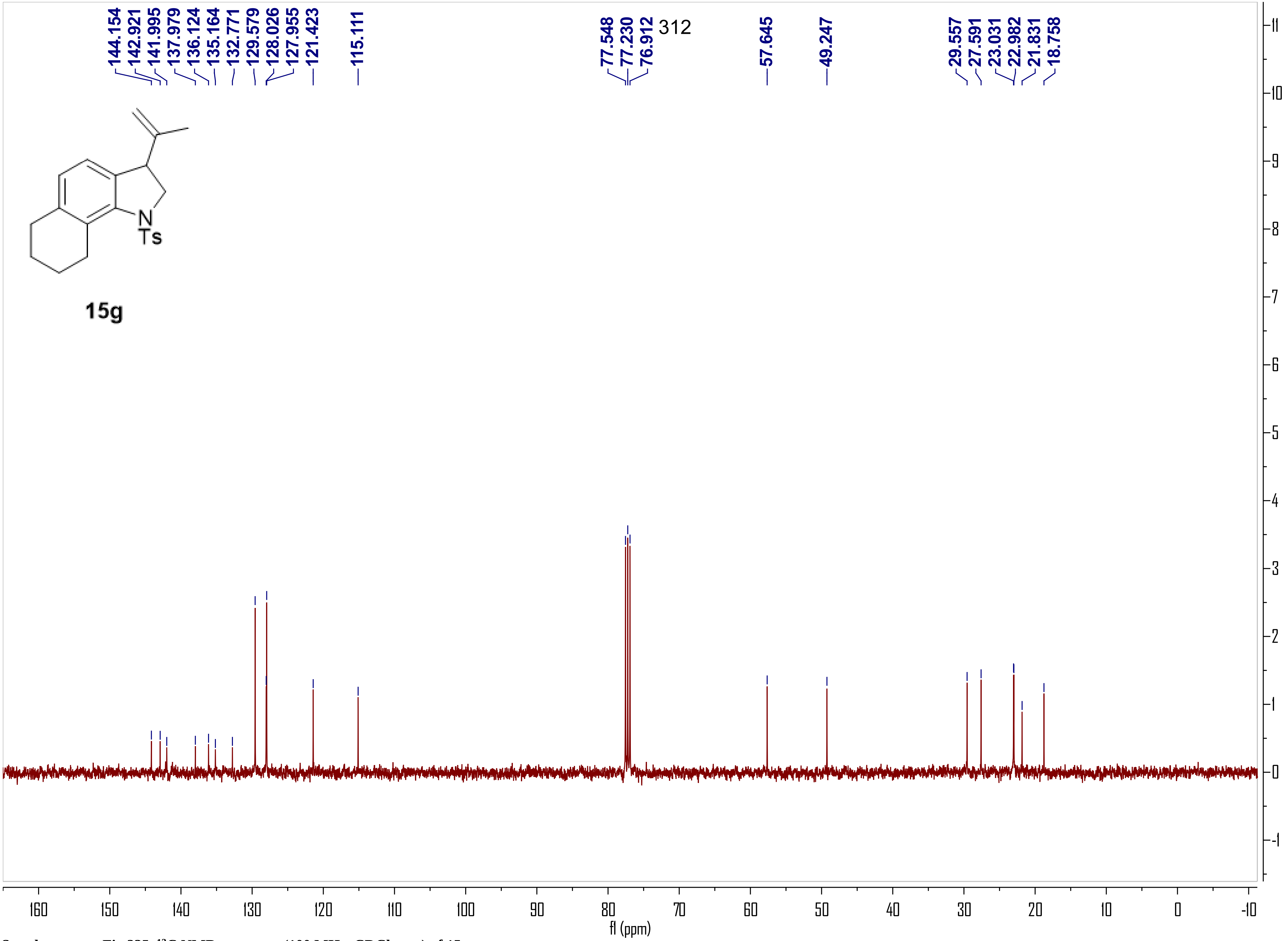

Supplementary Fig 235. ^{13}**C NMR spectrum (100 MHz, CDCl$_3$, r.t.) of 15g.**

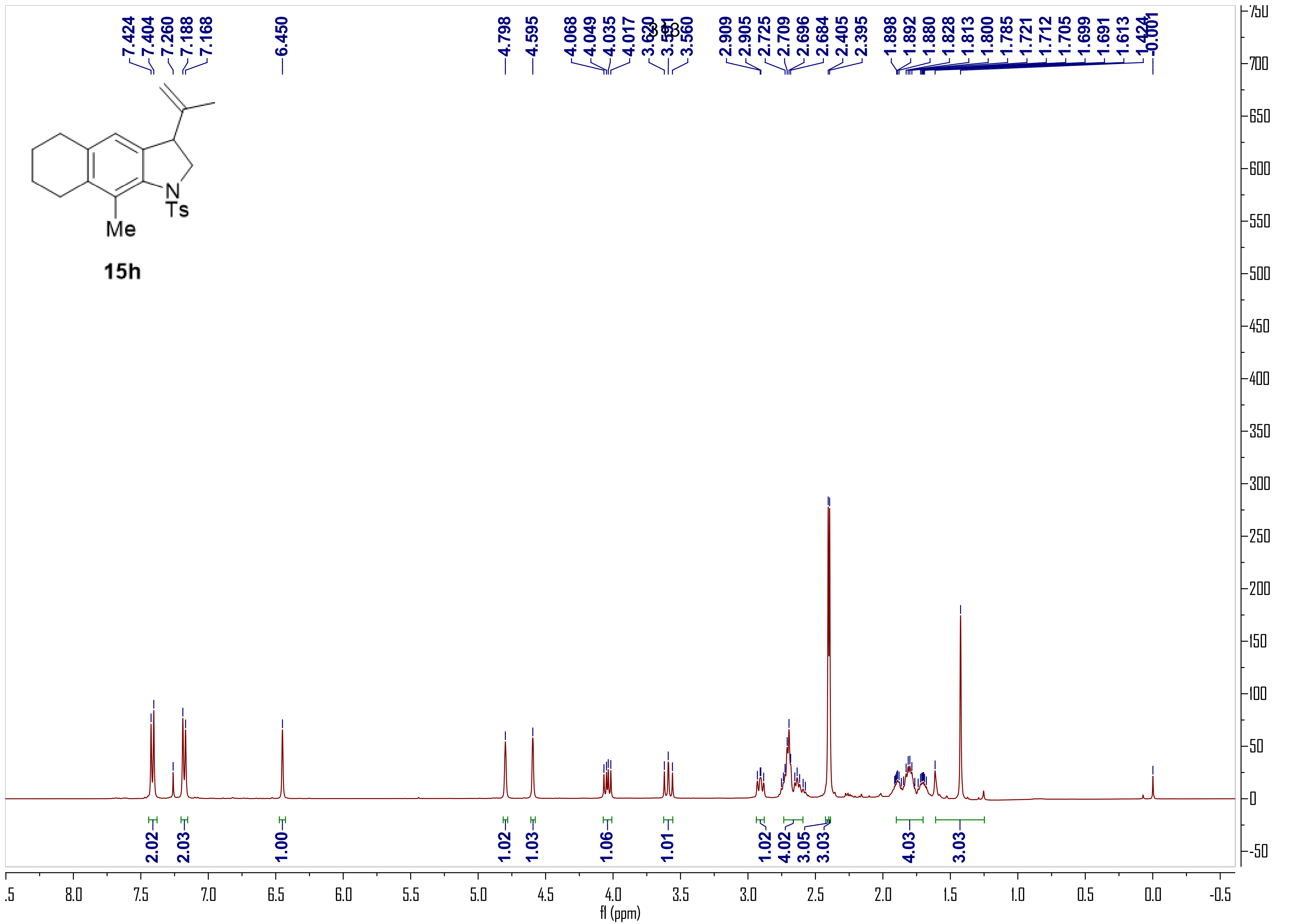

Supplementary Fig 236. ^{1}H NMR spectrum (400 MHz, $CDCl_3$, r.t.) of 15h.

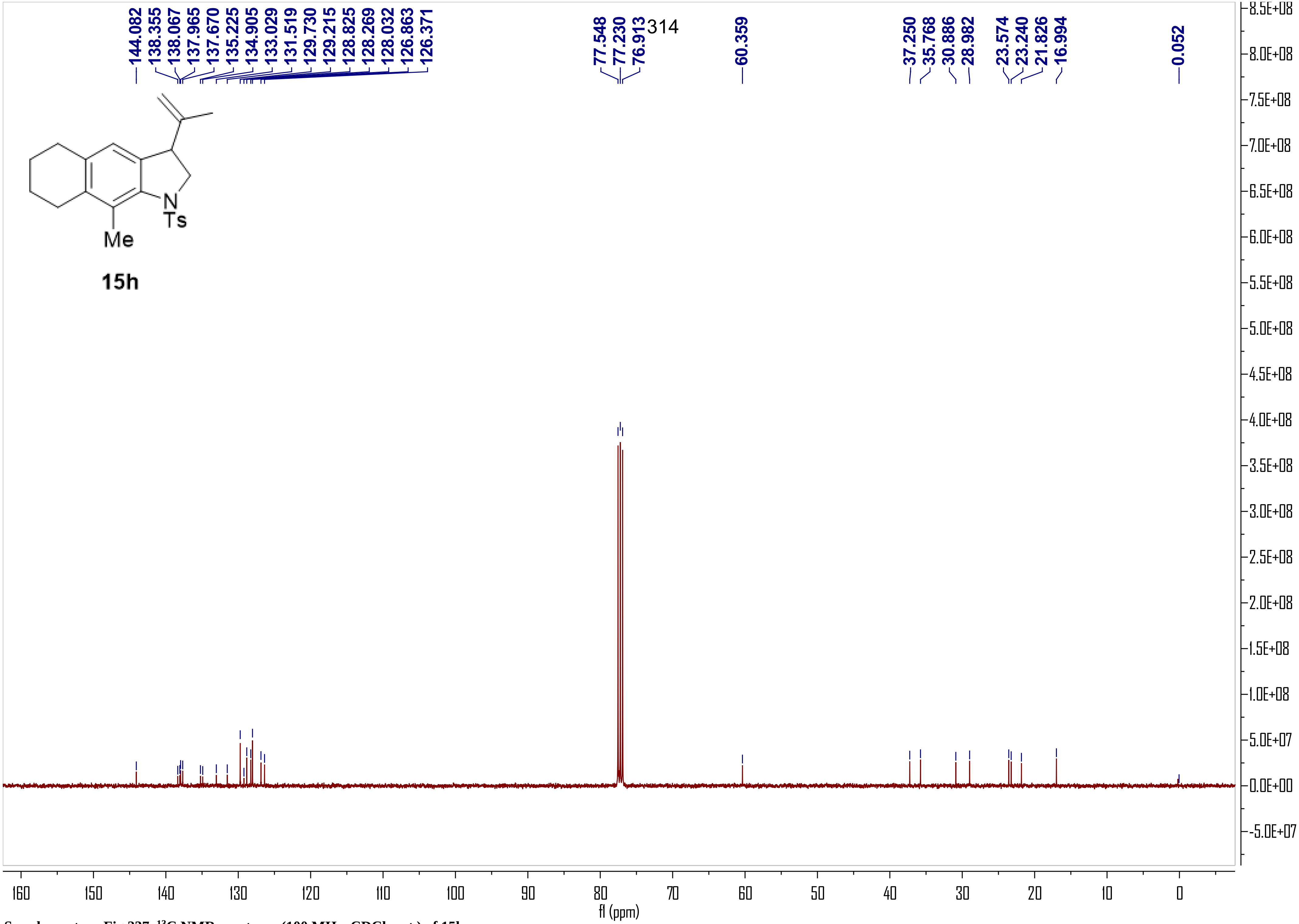

Supplementary Fig 237. ^{13}**C NMR spectrum (100 MHz, CDCl$_3$, r.t.) of 15h.**

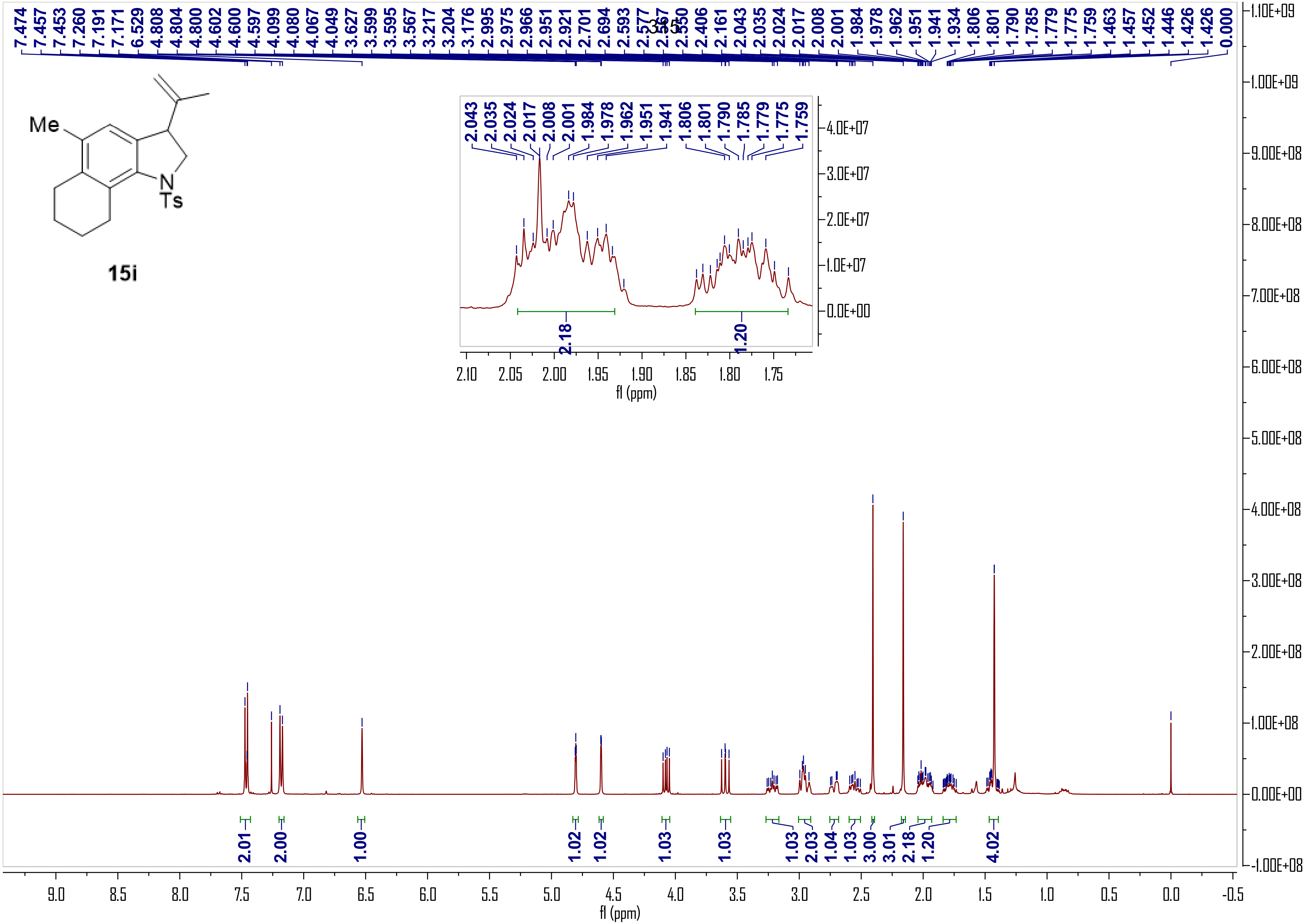

Supplementary Fig 238. ^{1}H NMR spectrum (400 MHz, $CDCl_3$, r.t.) of 15i.

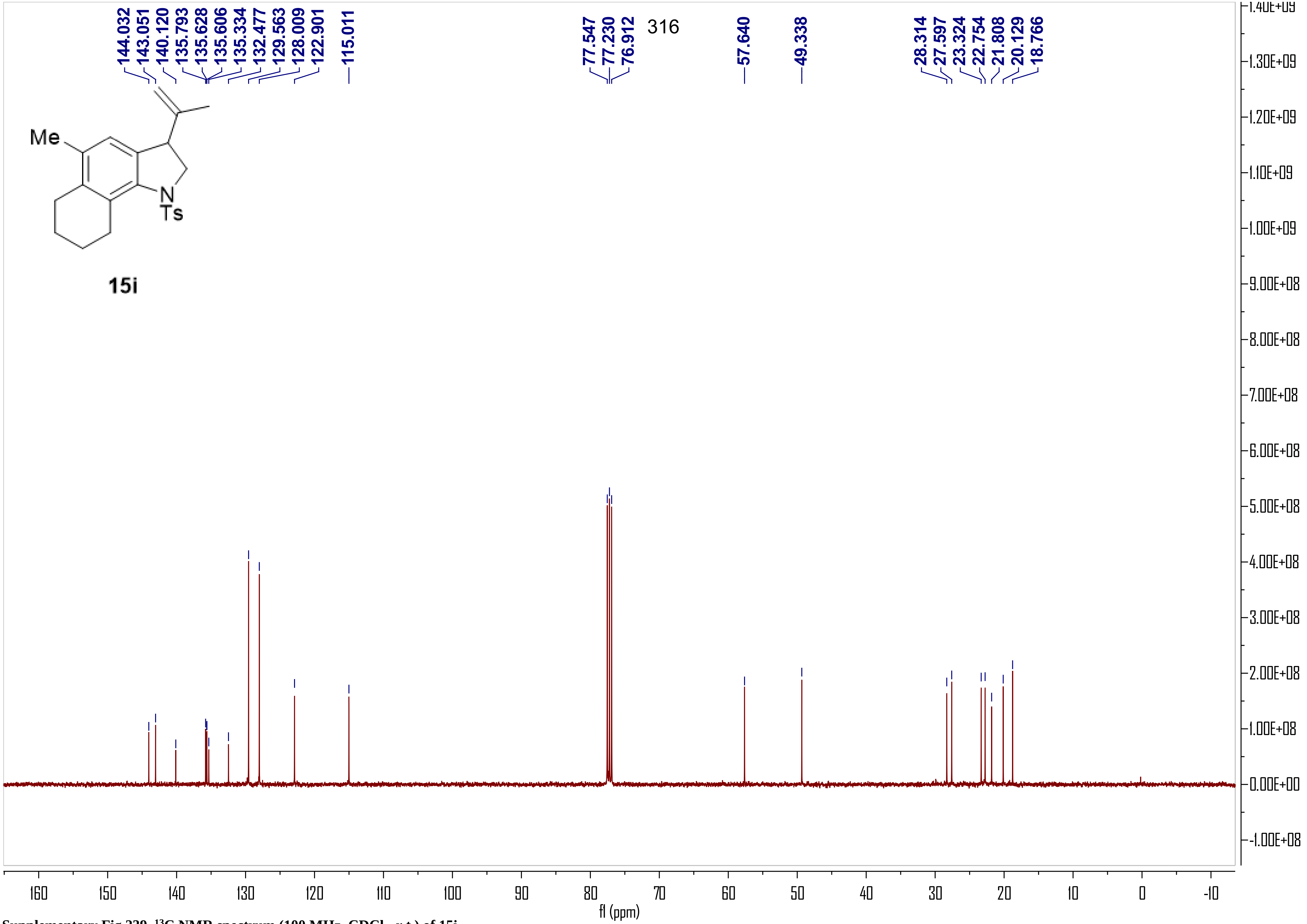

Supplementary Fig 239. ^{13}C NMR spectrum (100 MHz, $CDCl_3$, r.t.) of 15i.

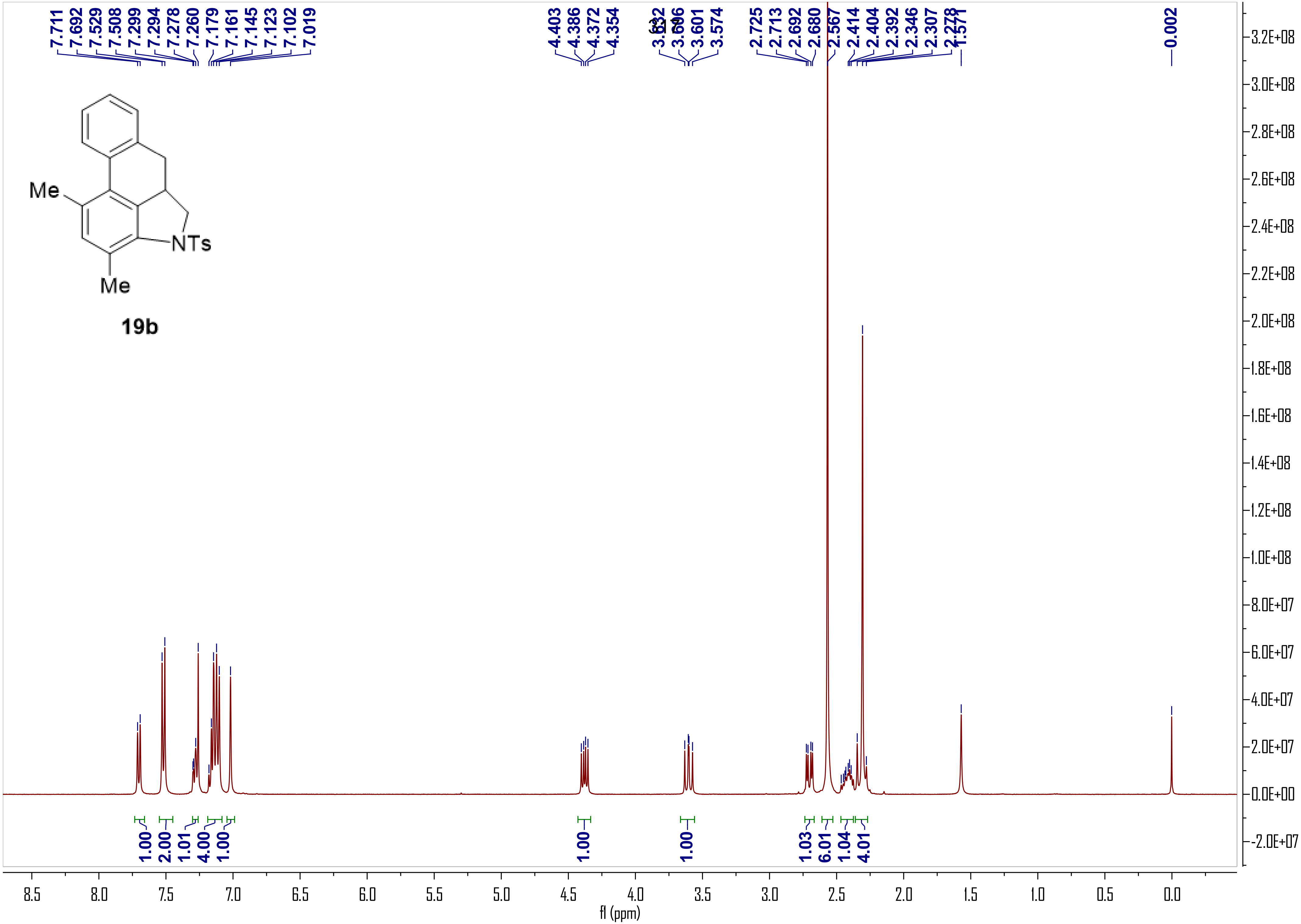

Supplementary Fig 240. **^{1}H NMR spectrum (400 MHz, $CDCl_3$, r.t.) of 19b.**

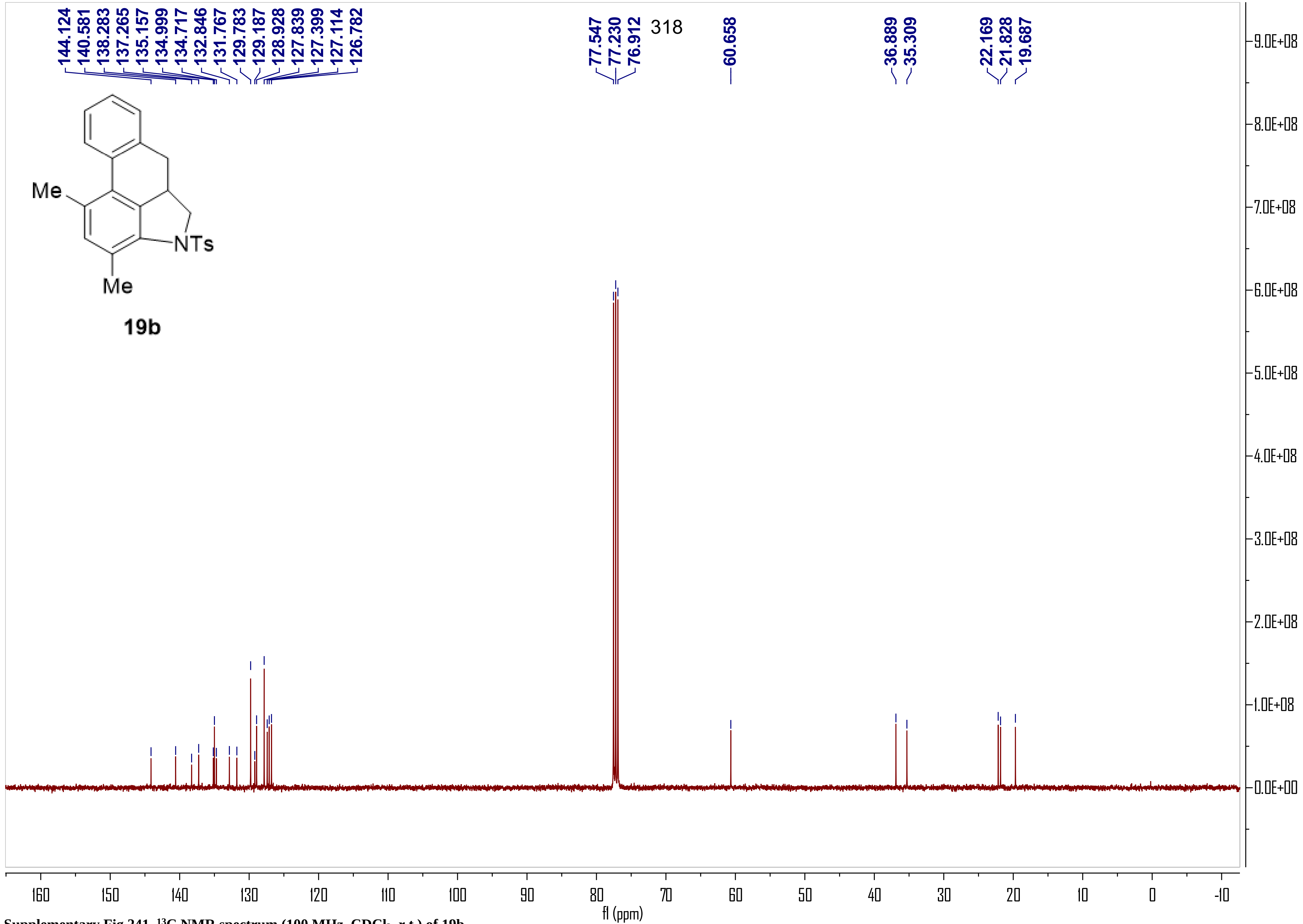

Supplementary Fig 241. ^{13}**C NMR spectrum (100 MHz, CDCl$_3$, r.t.) of 19b.**

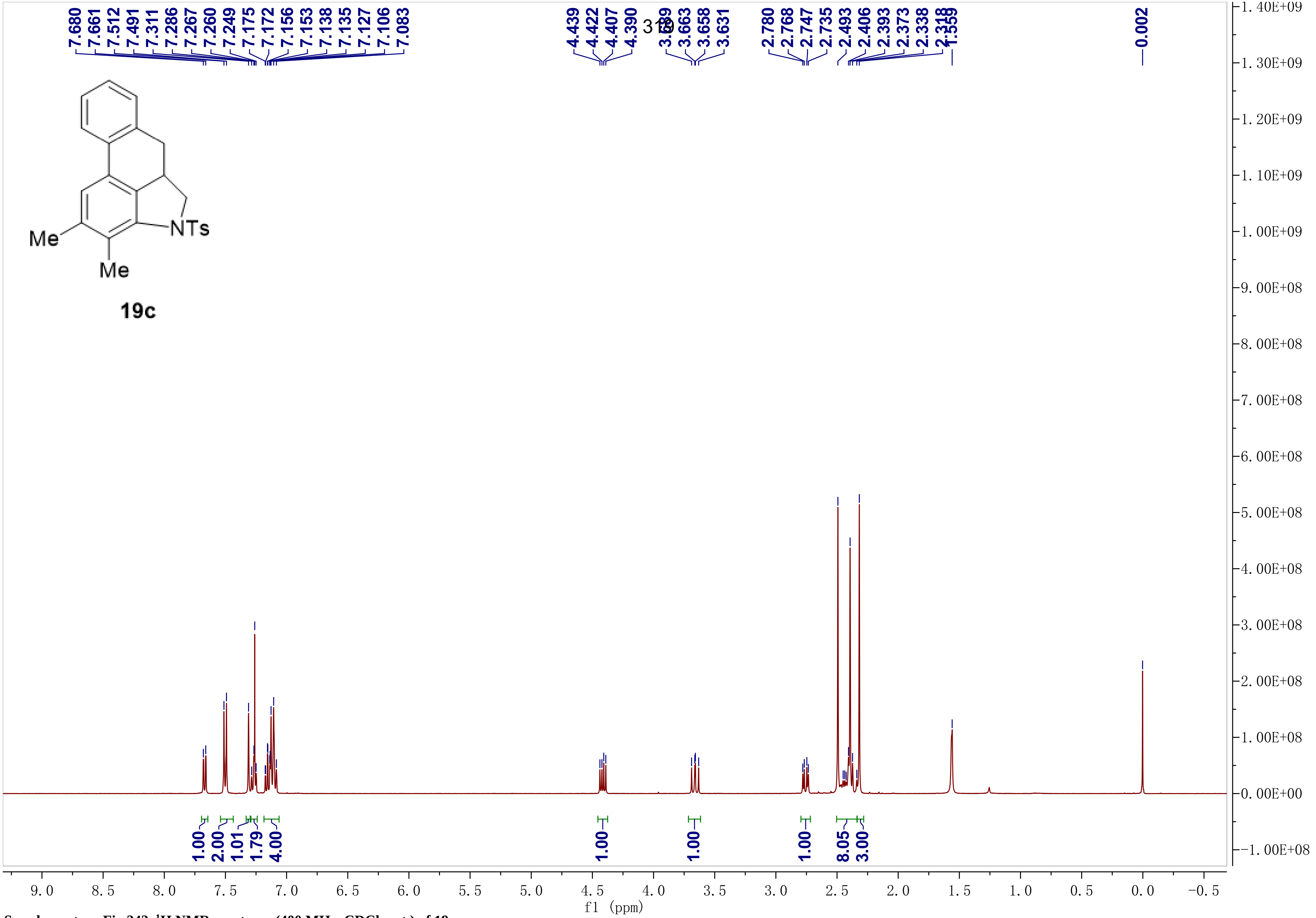

Supplementary Fig 242. ^{1}H NMR spectrum (400 MHz, $CDCl_3$, r.t.) of 19c.

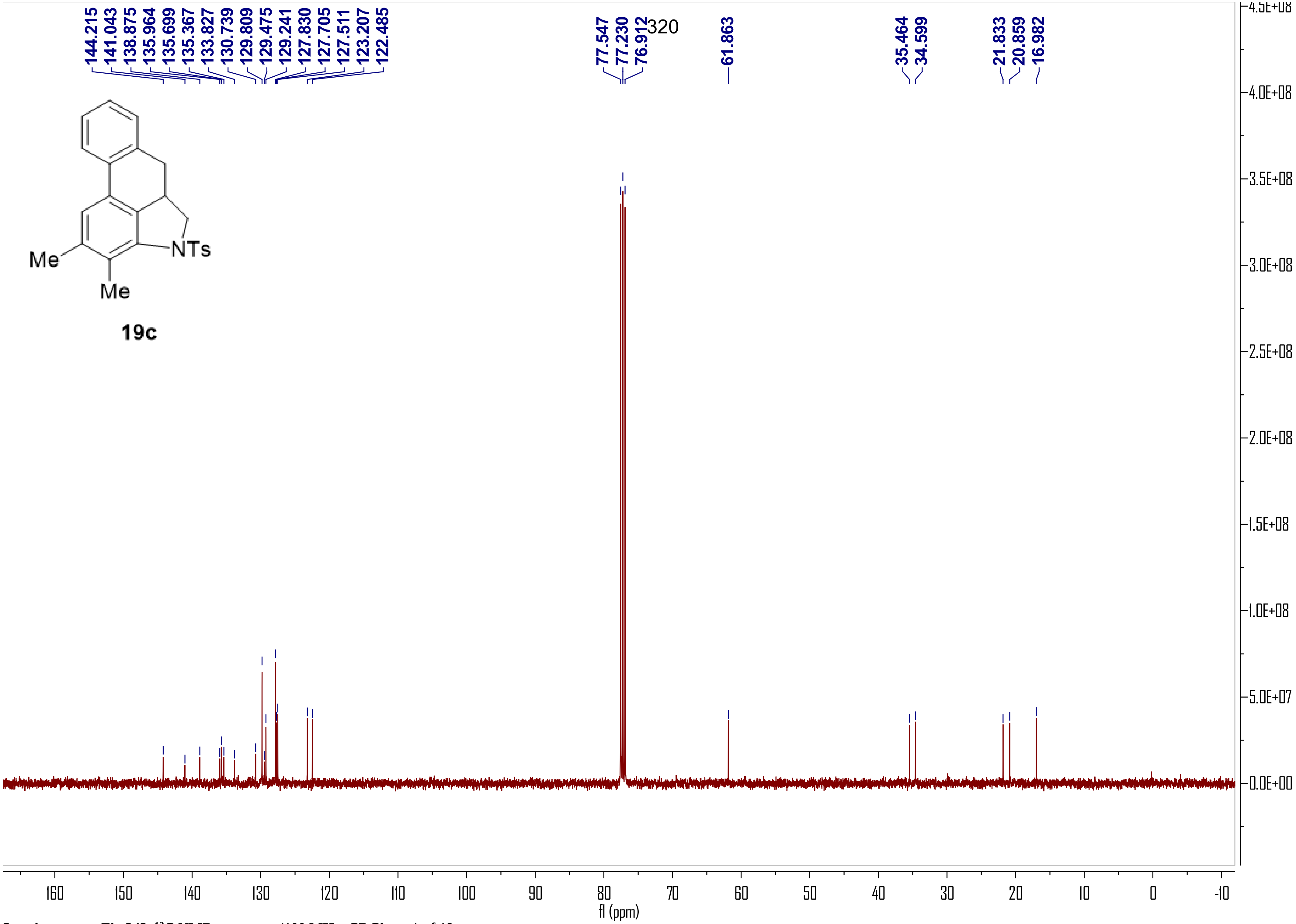

Supplementary Fig 243. ^{13}**C NMR spectrum (100 MHz, $CDCl_3$, r.t.) of 19c.**

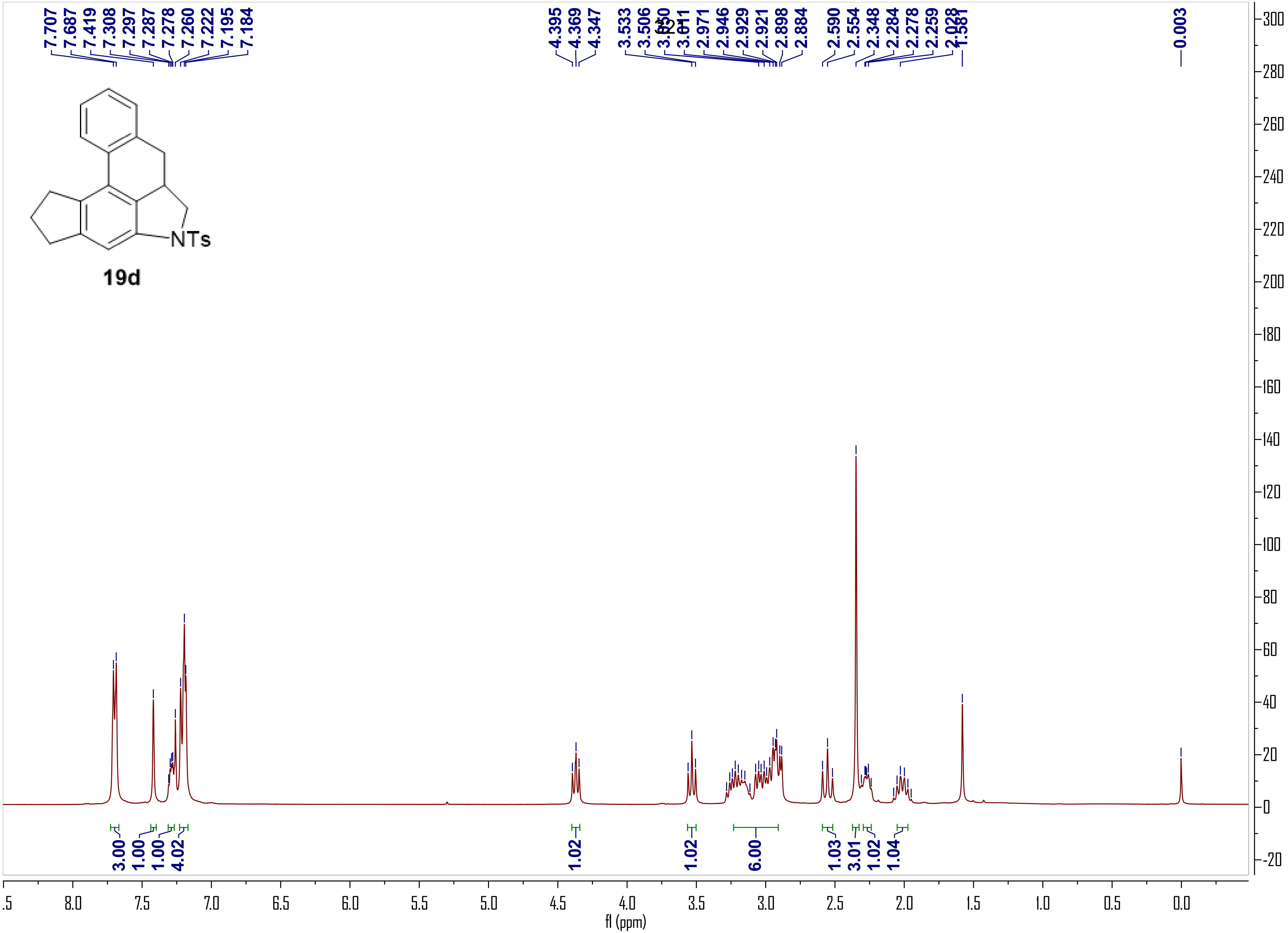

Supplementary Fig 244. **^{1}H NMR spectrum (400 MHz, $CDCl_3$, r.t.) of 19d.**

322

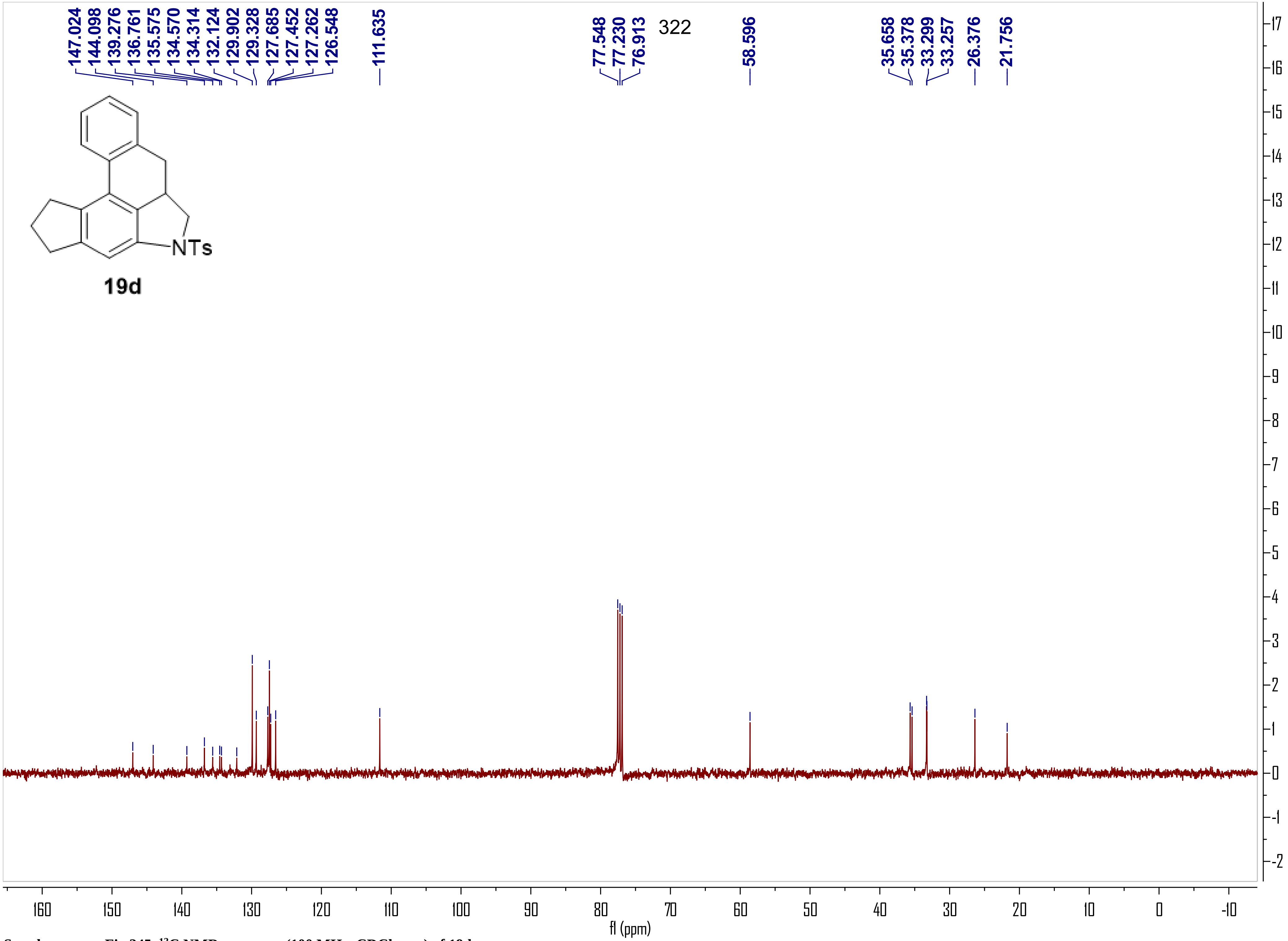

Supplementary Fig 245. ^{13}**C NMR spectrum (100 MHz, CDCl$_3$, r.t.) of 19d.**

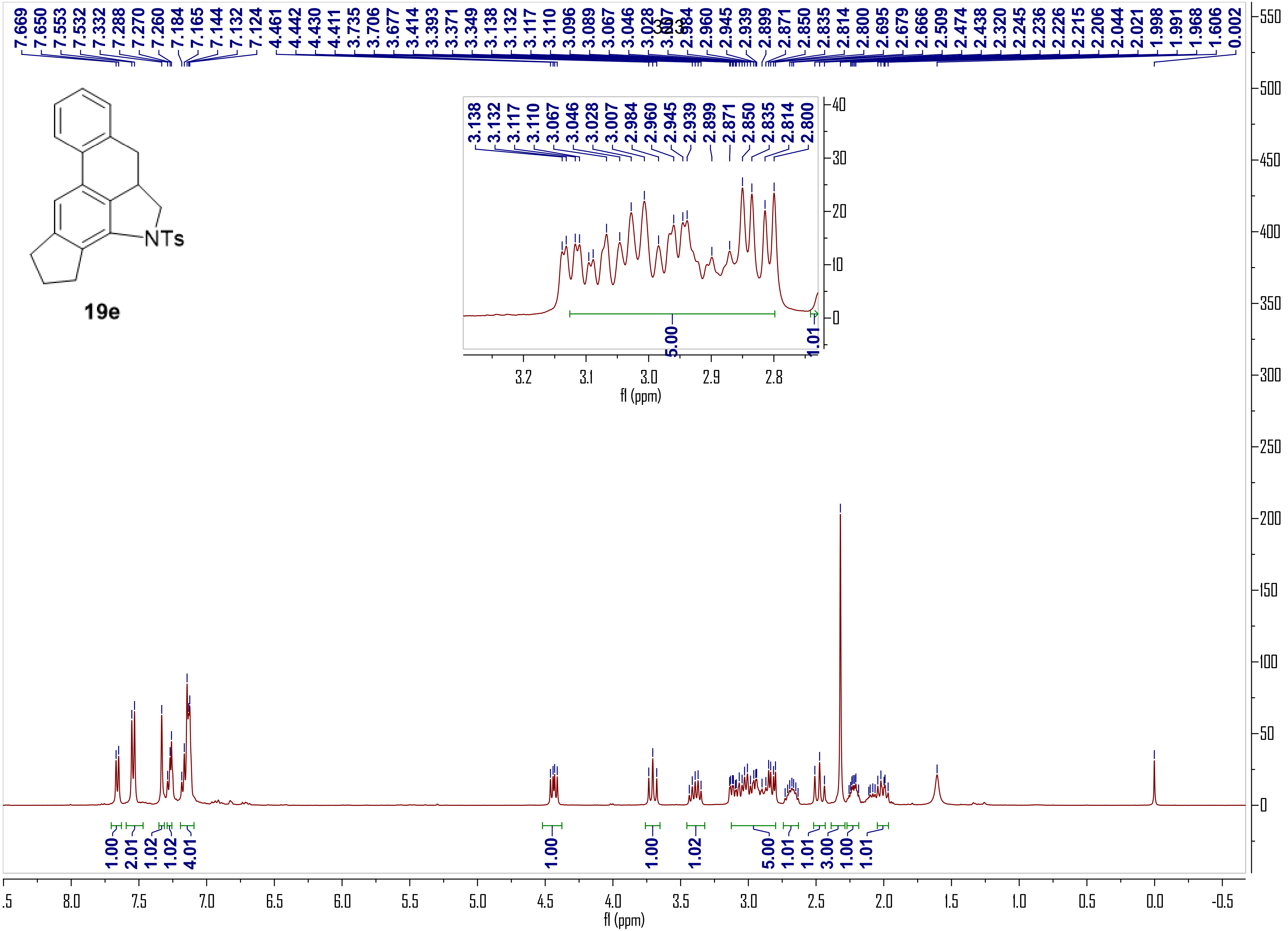

Supplementary Fig 246. **^{1}H NMR spectrum (400 MHz, $CDCl_3$, r.t.) of 19e.**

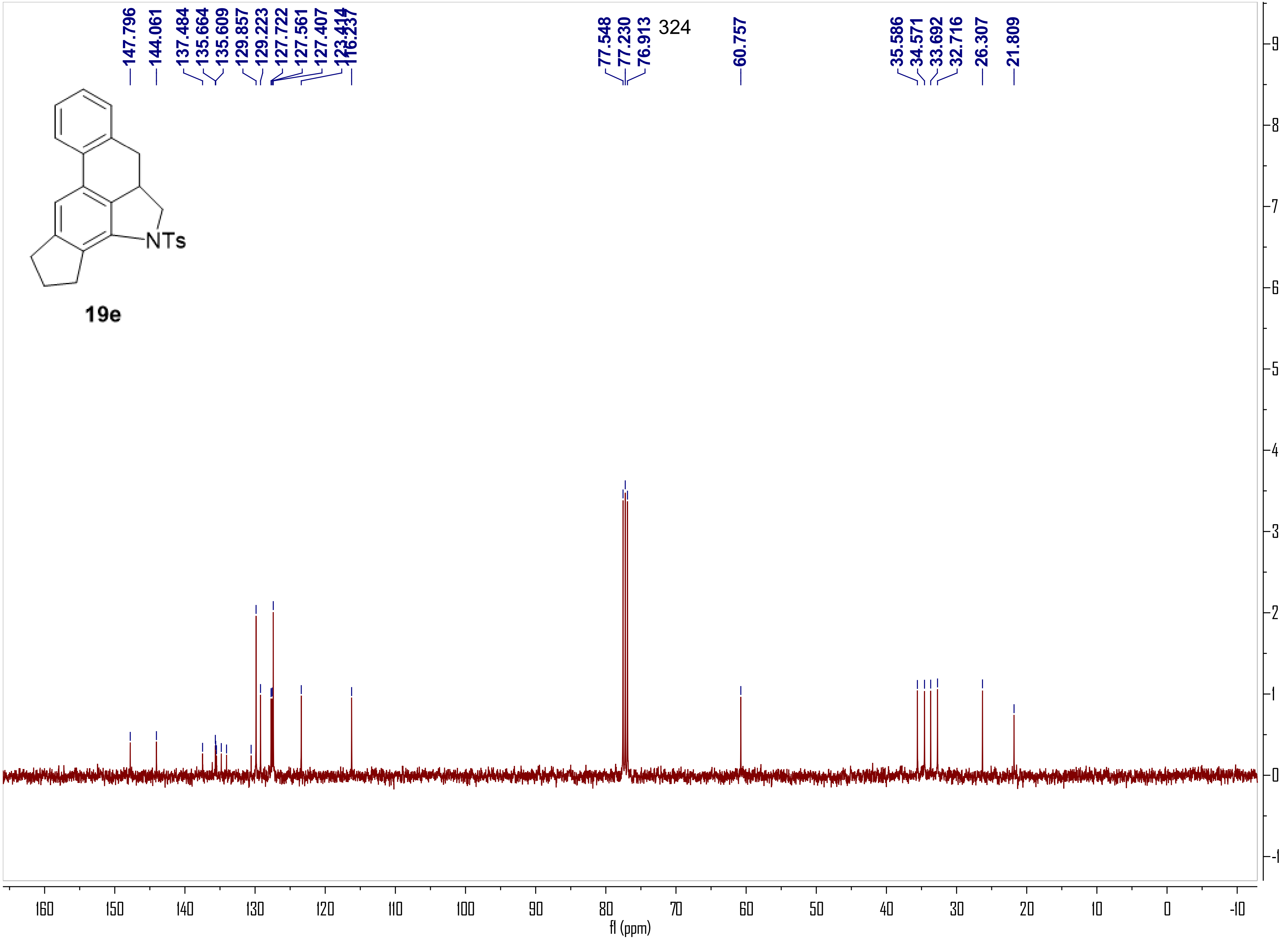

Supplementary Fig 247. **^{13}C NMR spectrum (100 MHz, $CDCl_3$, r.t.) of 19e.**

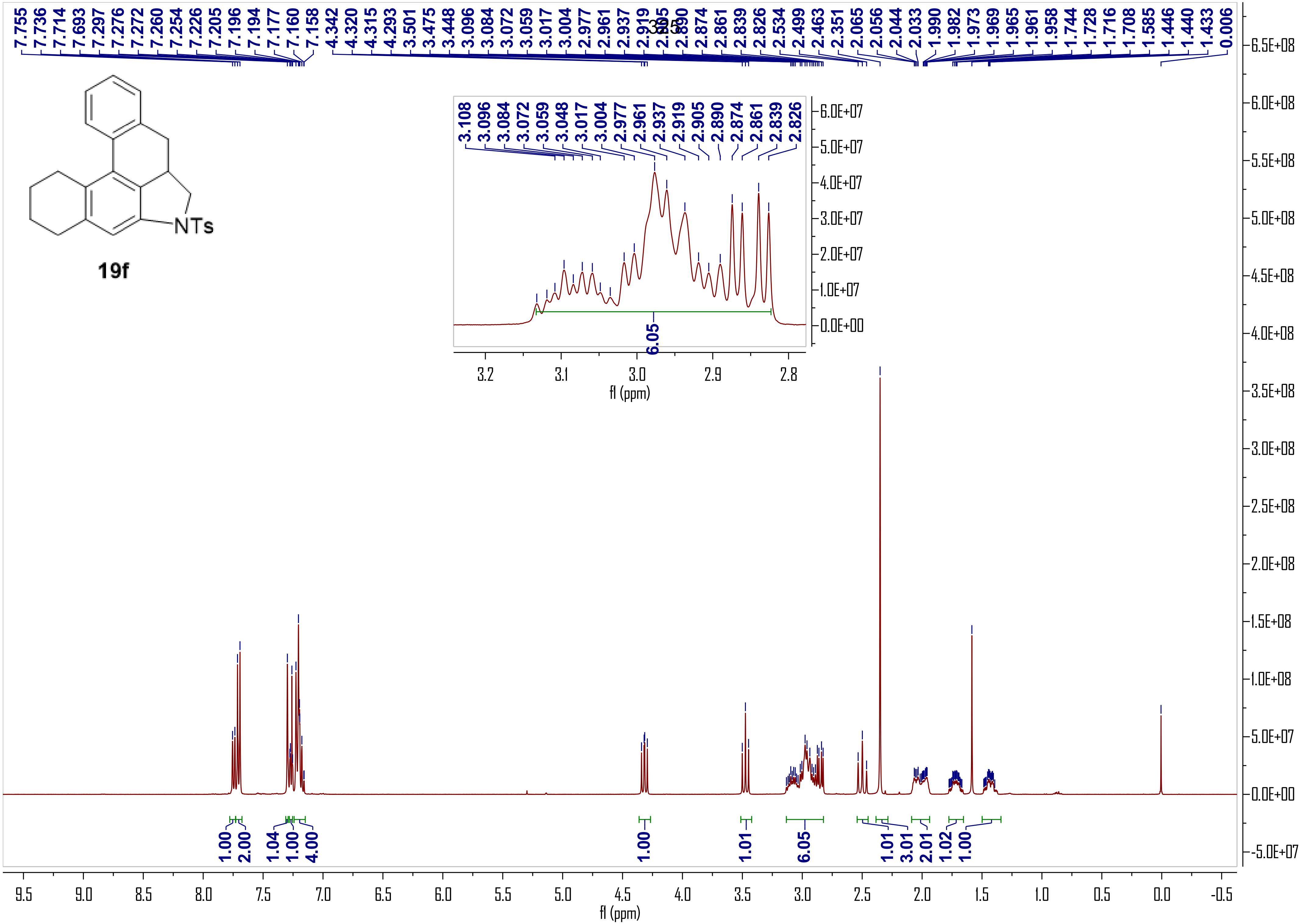

Supplementary Fig 248. ¹H NMR spectrum (400 MHz, $CDCl_3$, r.t.) of 19f.

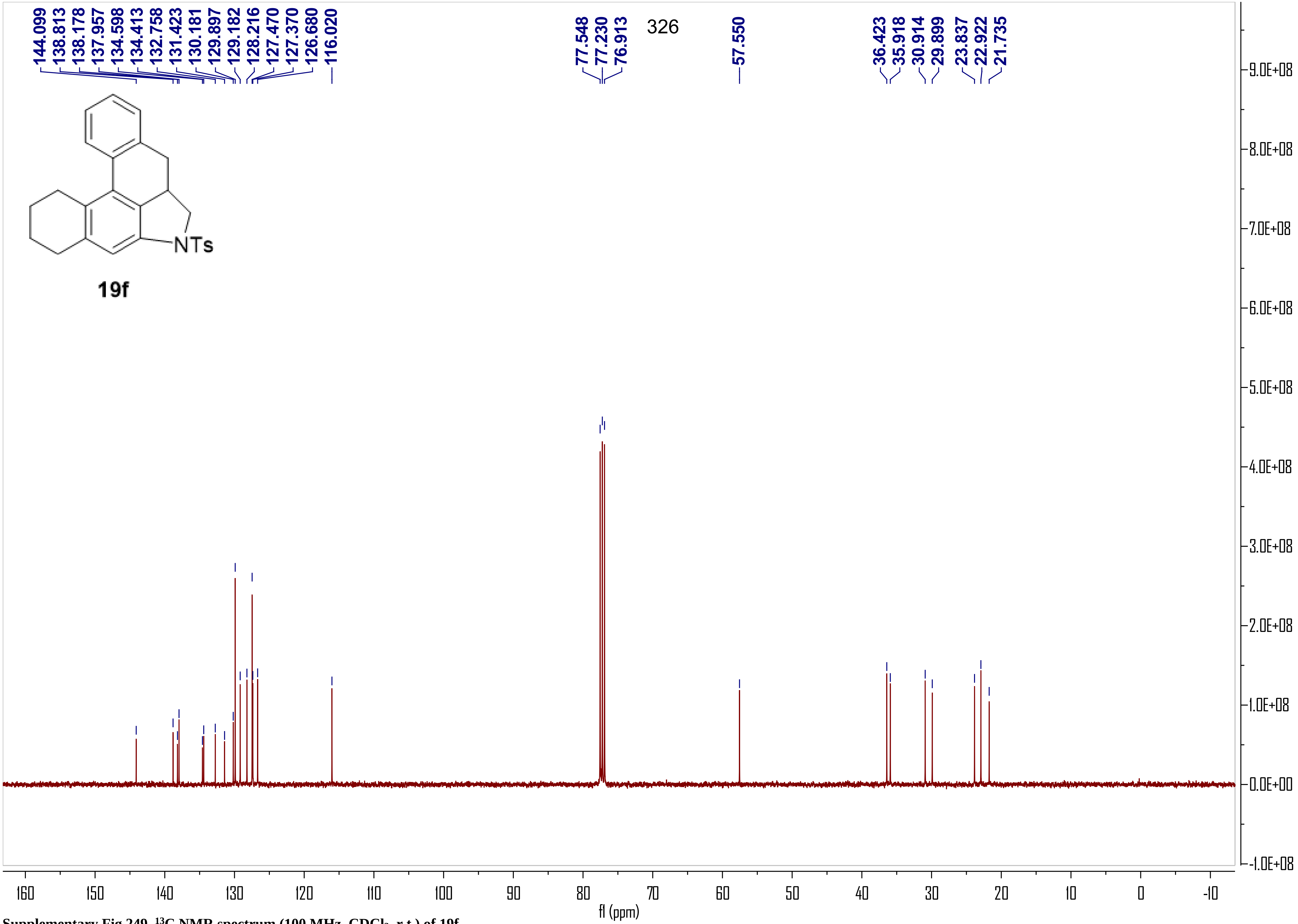

Supplementary Fig 249. ^{13}**C NMR spectrum (100 MHz, CDCl$_3$, r.t.) of 19f.**

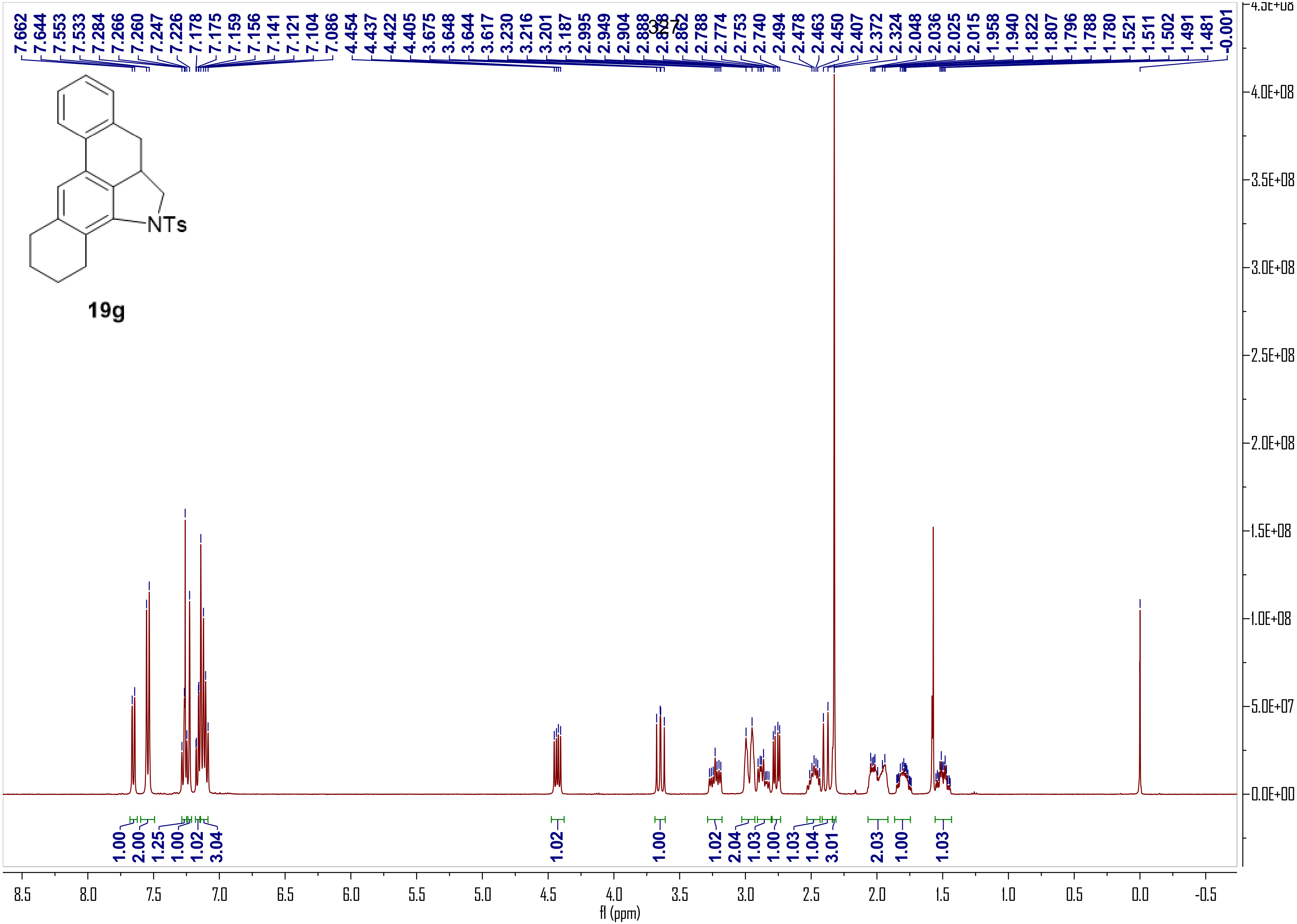

Supplementary Fig 250. ^{1}H NMR spectrum (400 MHz, $CDCl_3$, r.t.) of 19g.

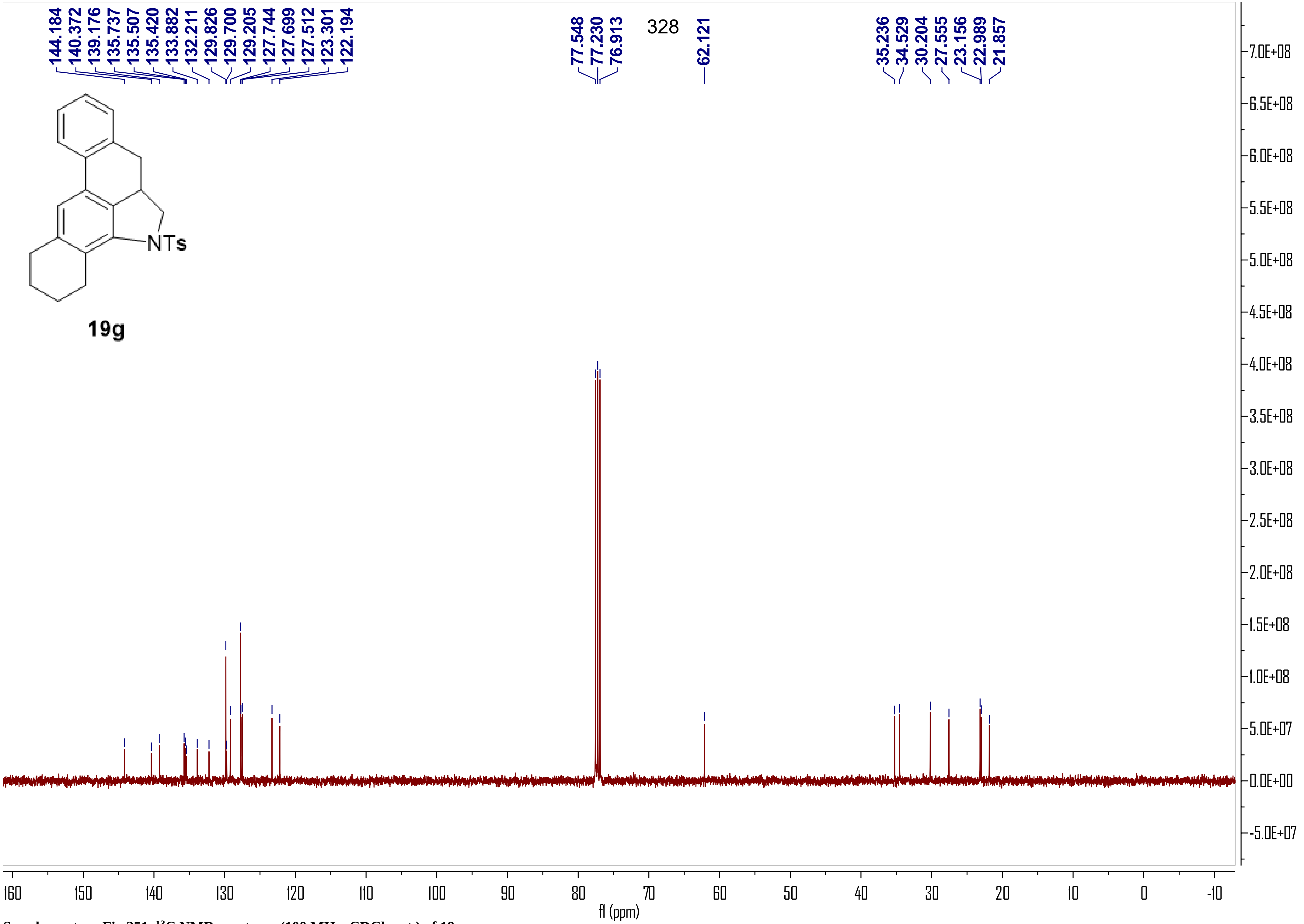

Supplementary Fig 251. ^{13}C **NMR spectrum (100 MHz,** $CDCl_3$**, r.t.) of 19g.**

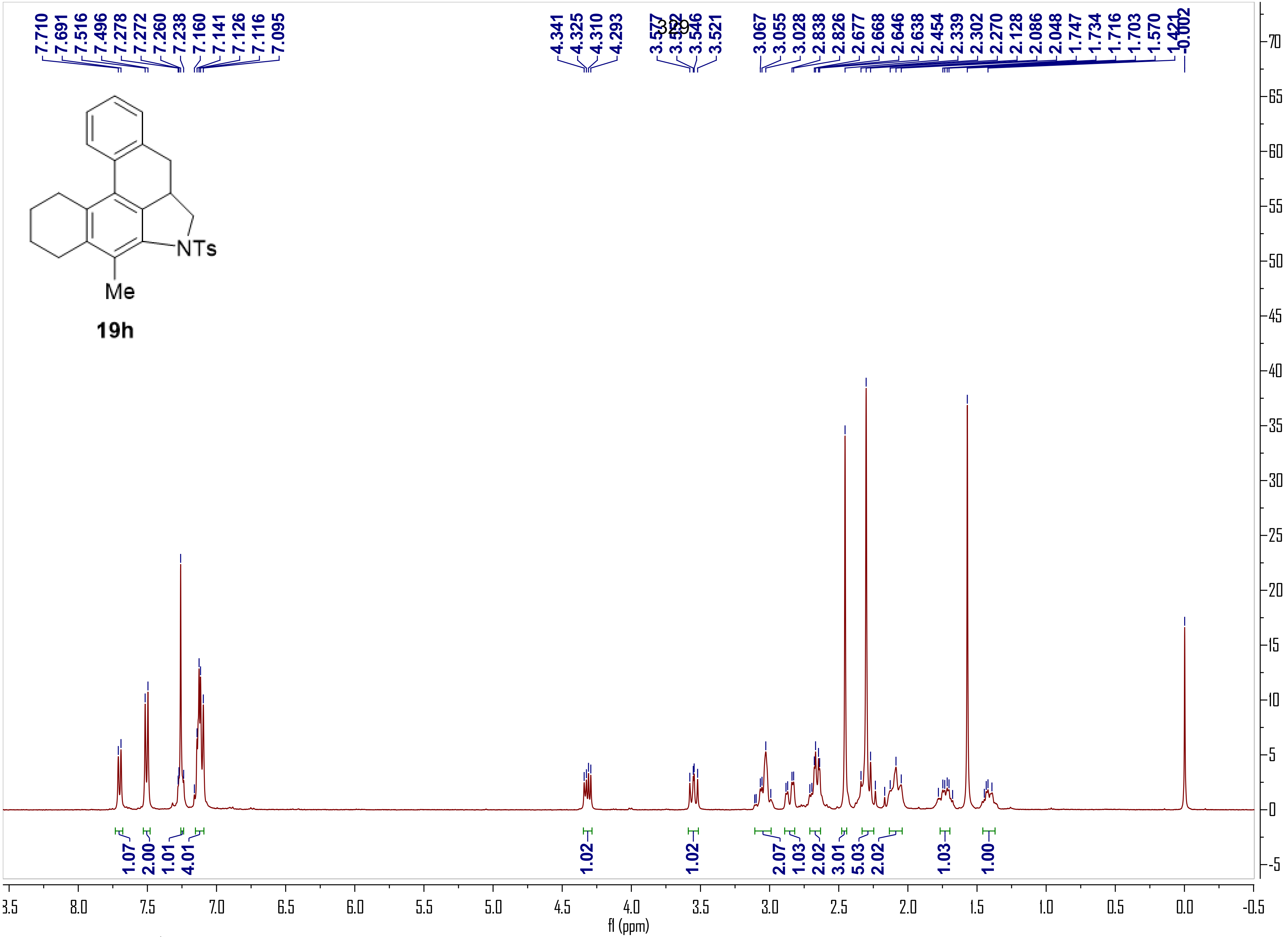

Supplementary Fig 252. ^{1}**H NMR spectrum (400 MHz, CDCl$_3$, r.t.) of 19h.**

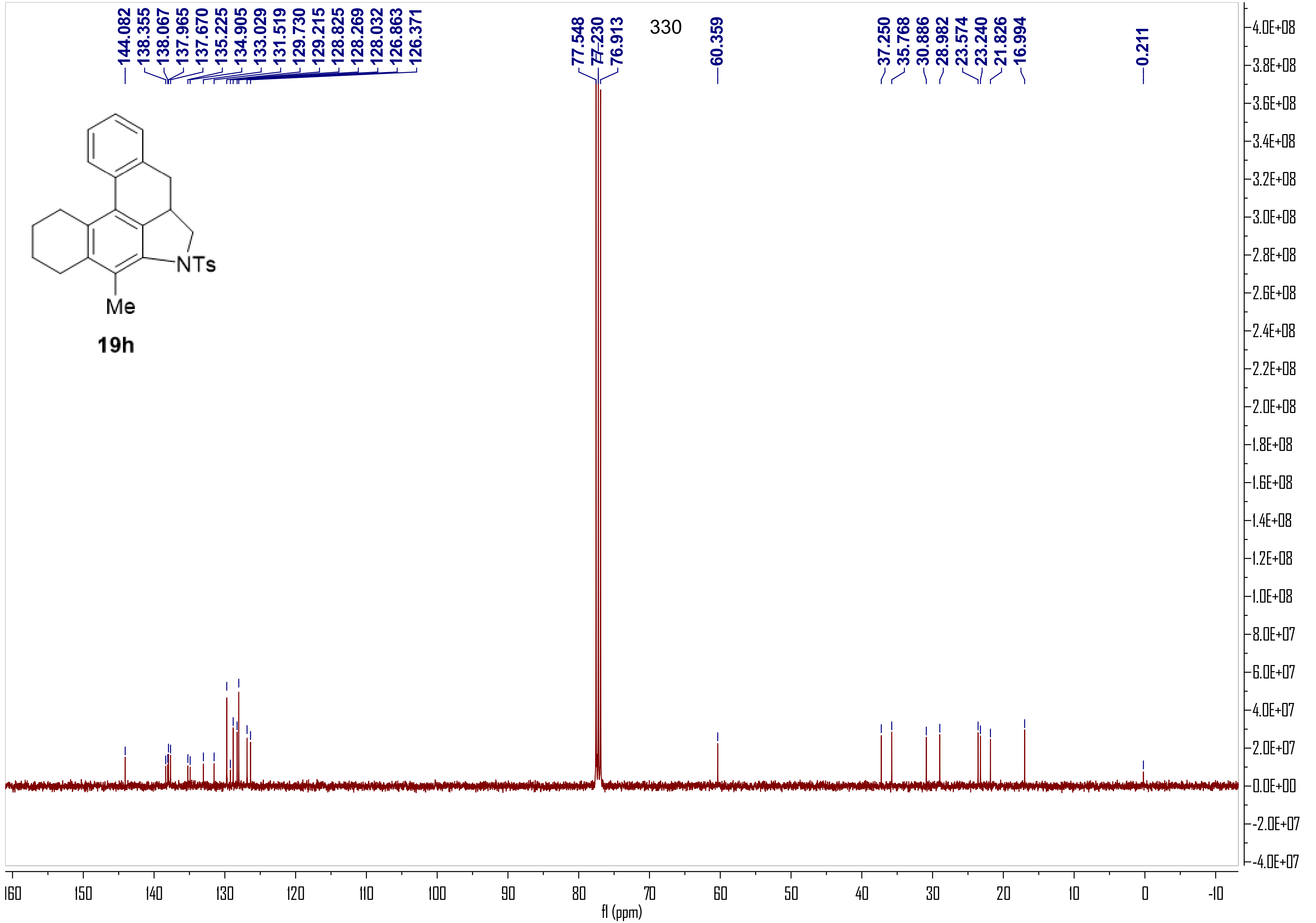

Supplementary Fig 253. ^{13}C **NMR spectrum (100 MHz,** $CDCl_3$**, r.t.) of 19h.**

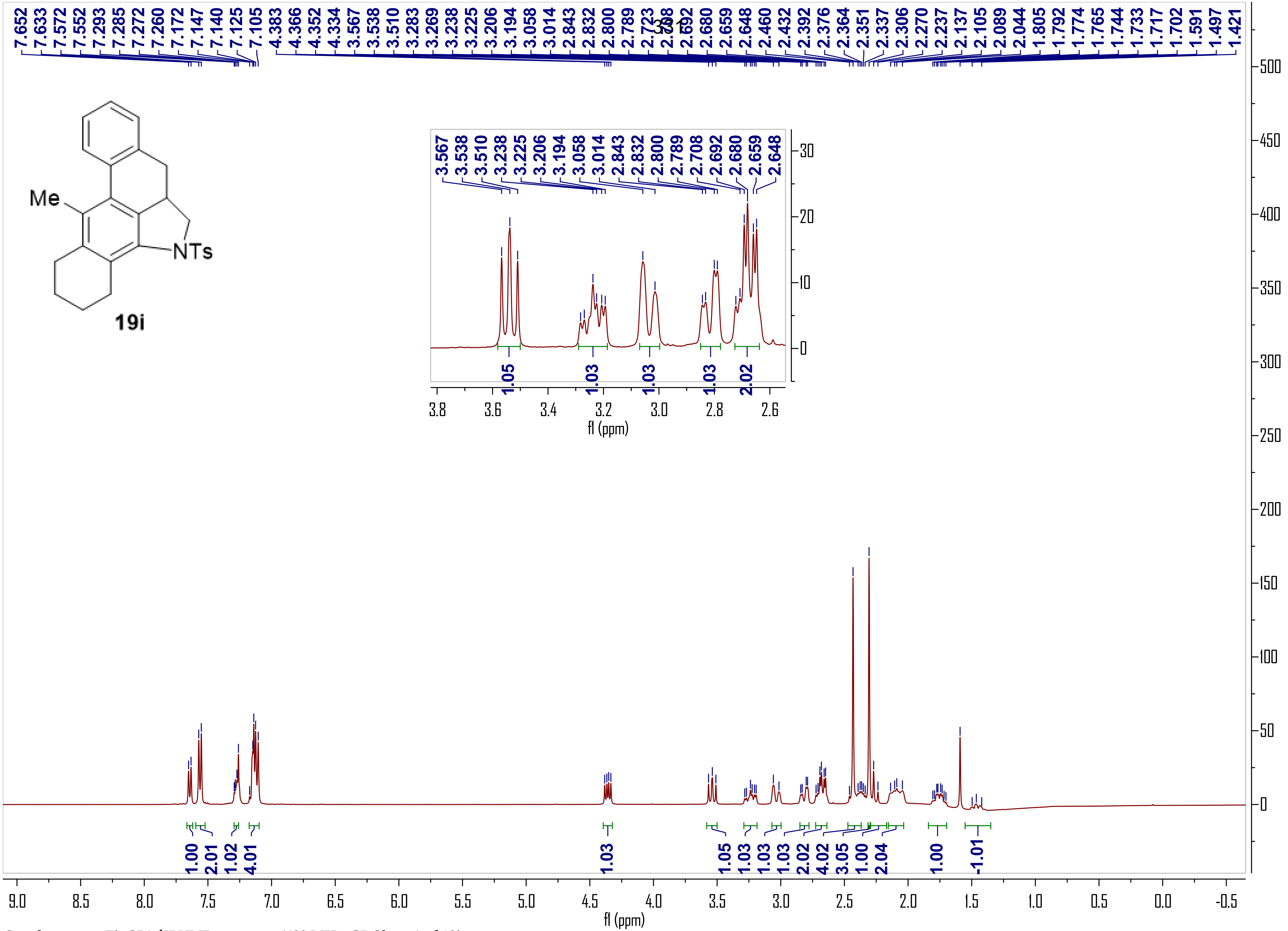

Supplementary Fig 254. ^{1}H NMR spectrum (400 MHz, $CDCl_3$, r.t.) of 19i.

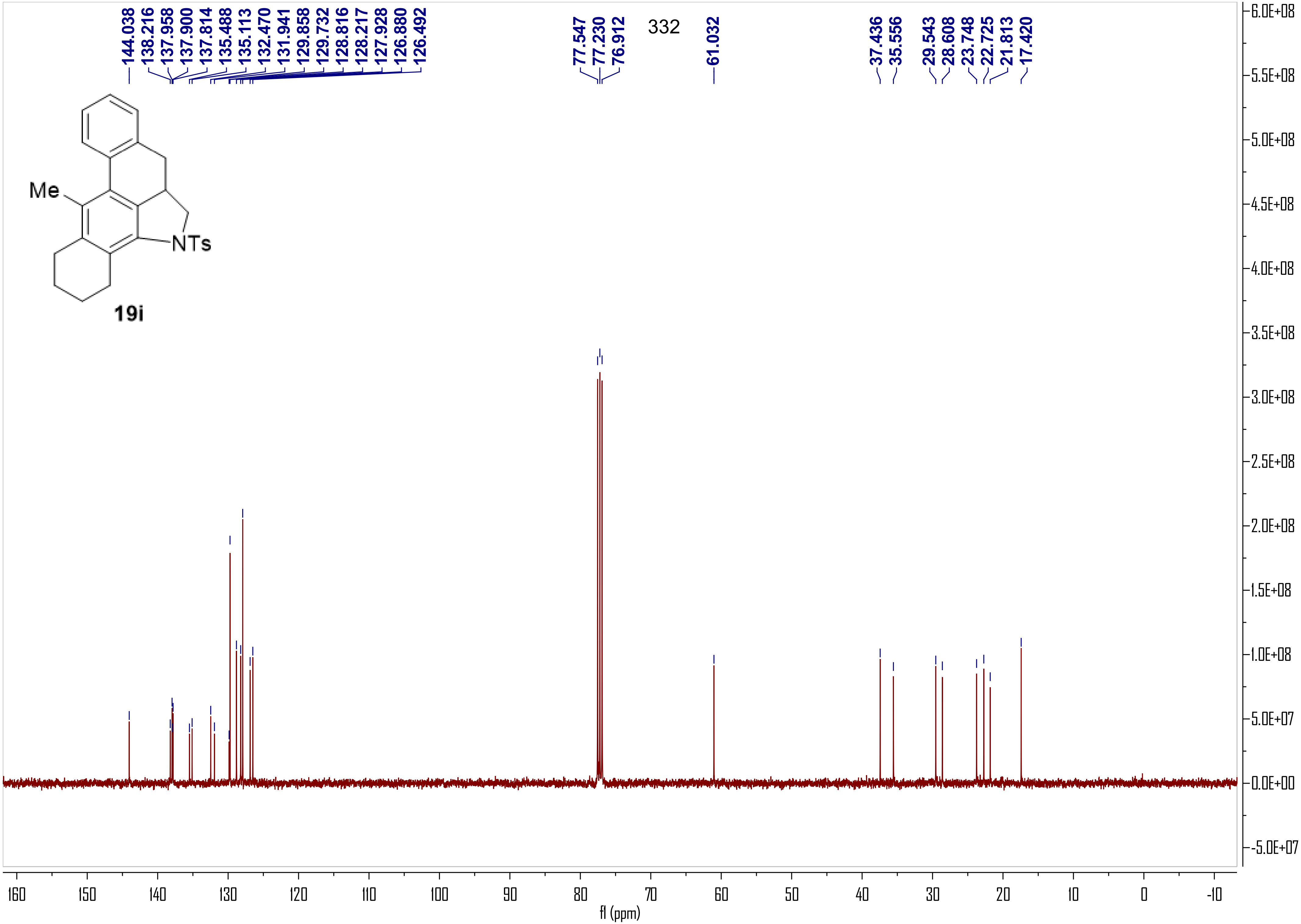

Supplementary Fig 255. ^{13}C NMR spectrum (100 MHz, $CDCl_3$, r.t.) of 19i.

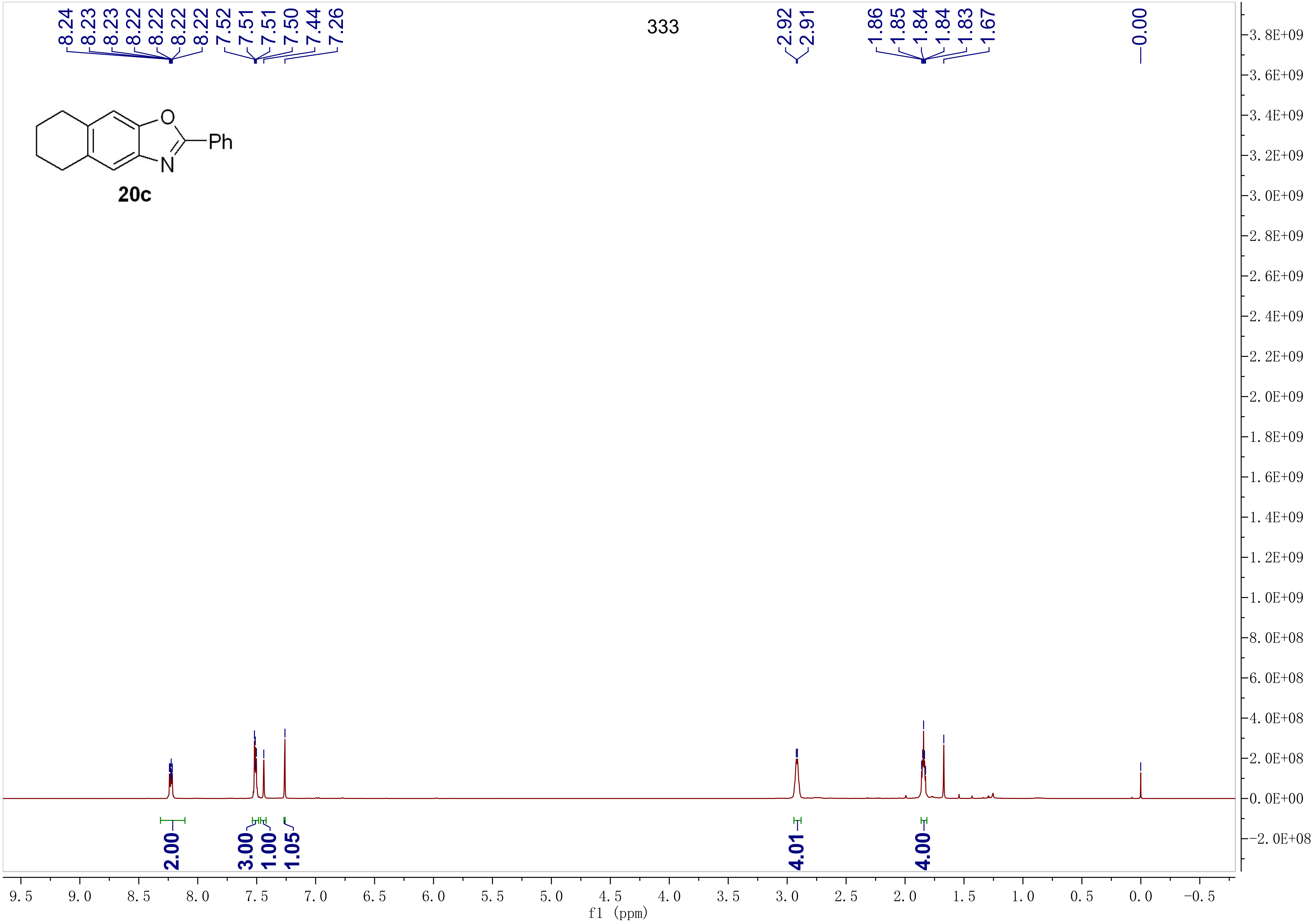

Supplementary Fig 256. ^{1}H NMR spectrum (400 MHz, $CDCl_3$, r.t.) of 20c.

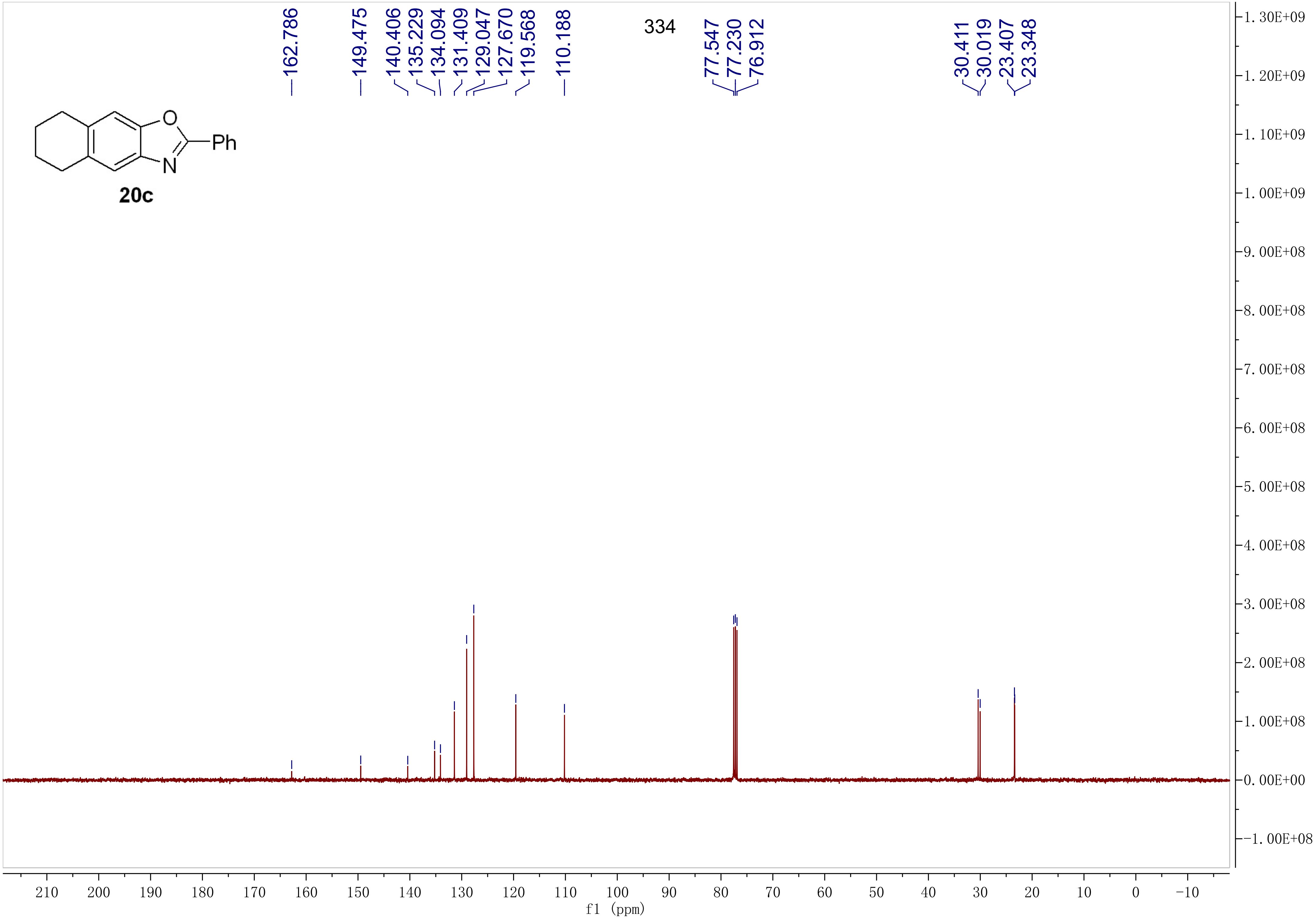

20c

Supplementary Fig 257. ^{13}C NMR spectrum (100 MHz, $CDCl_3$, r.t.) of 20c.

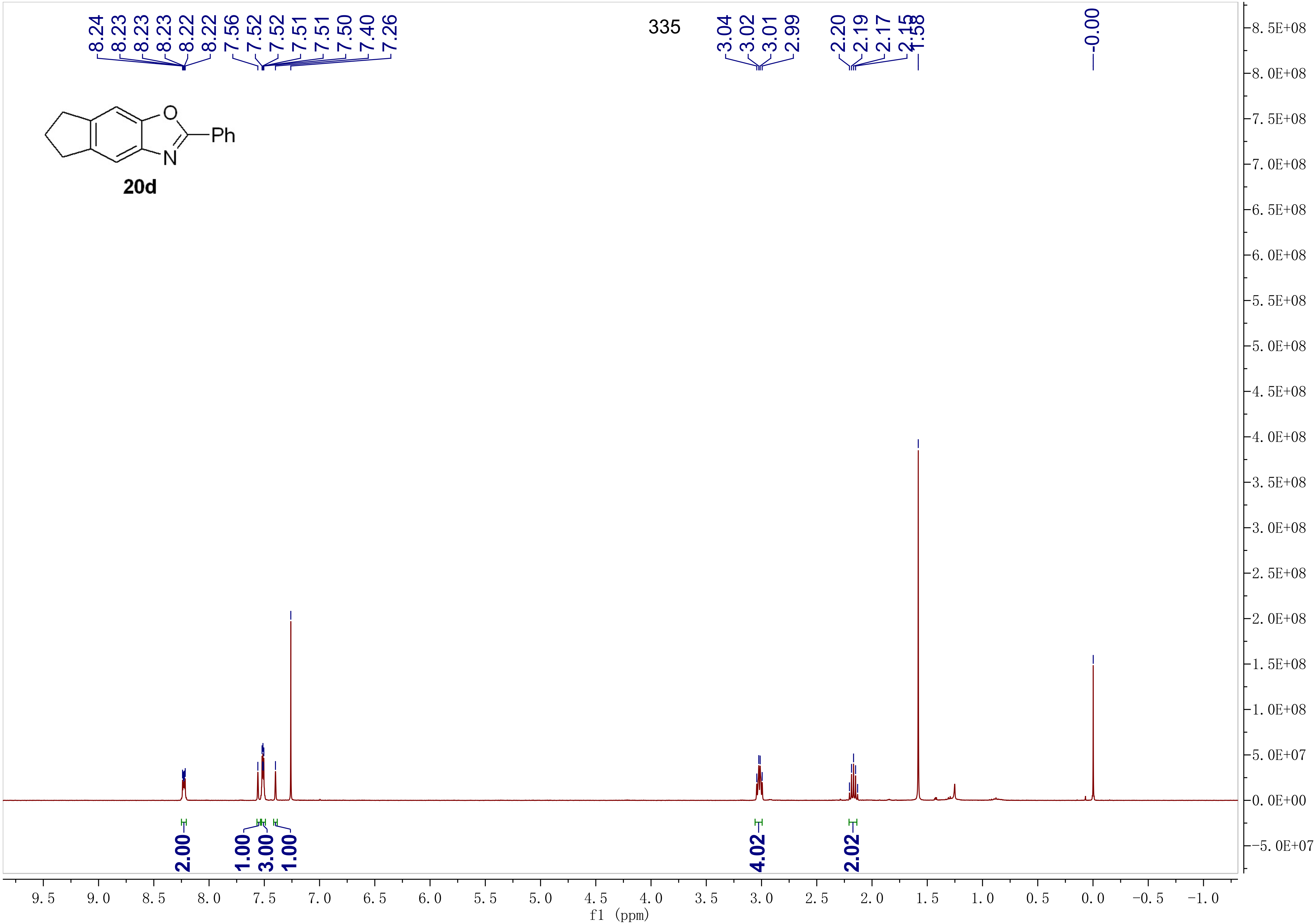

20d

Supplementary Fig 258. ^{1}H NMR spectrum (400 MHz, CDCl$_3$, r.t.) of 20d.

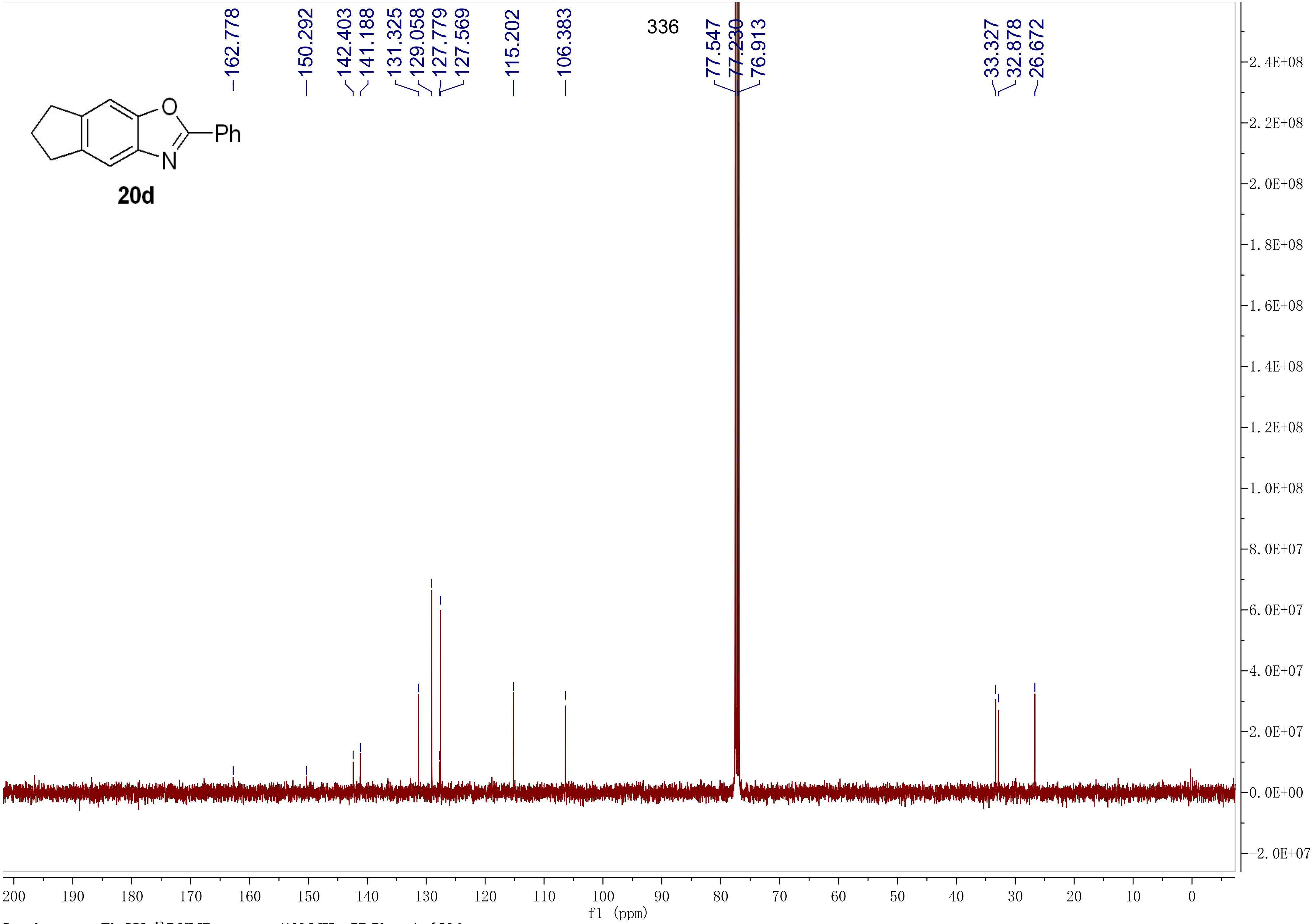

Supplementary Fig 259. **^{13}C NMR spectrum (100 MHz, $CDCl_3$, r.t.) of 20d.**

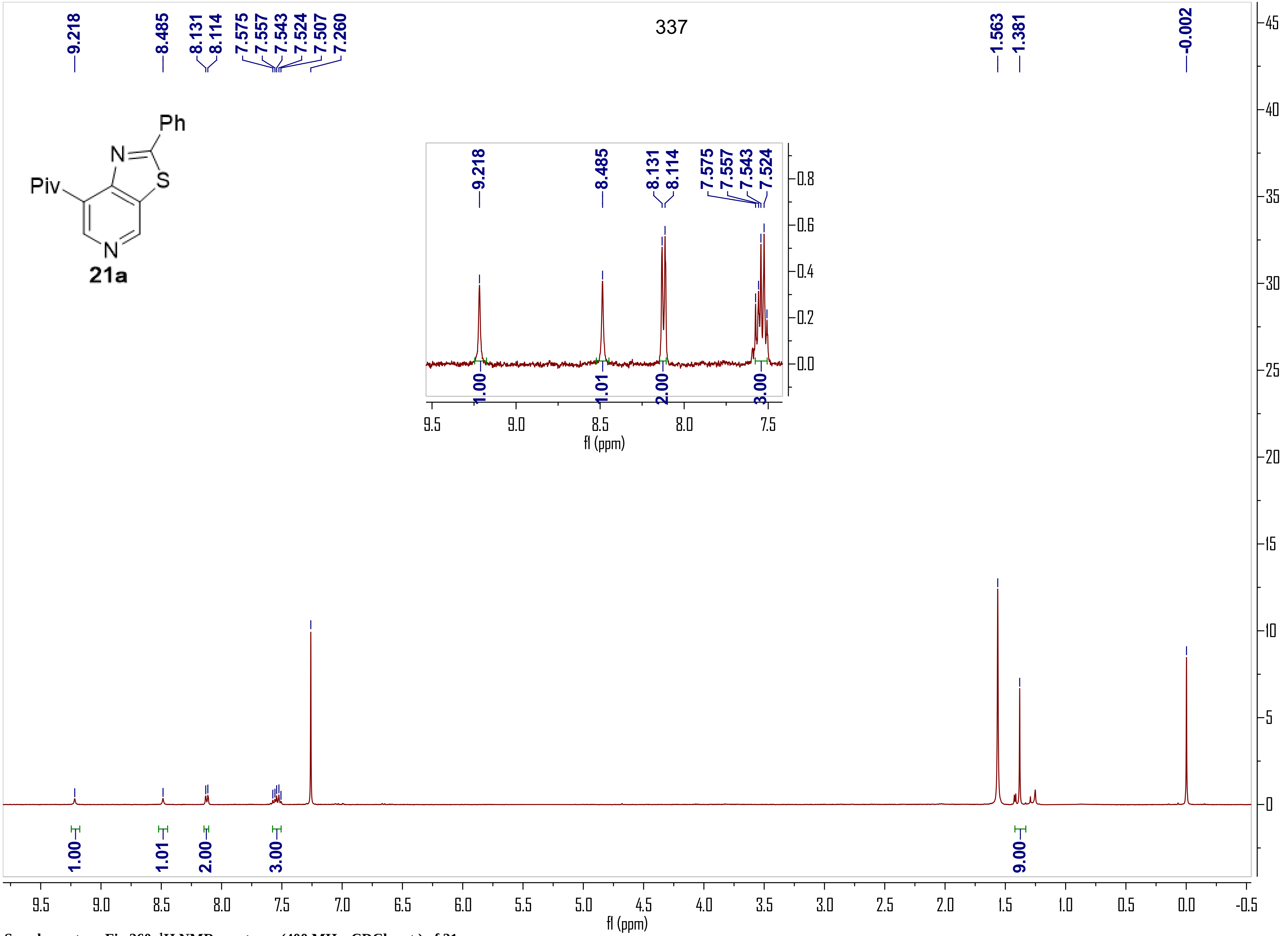

Supplementary Fig 260. ^{1}**H NMR spectrum (400 MHz, CDCl$_3$, r.t.) of 21a.**

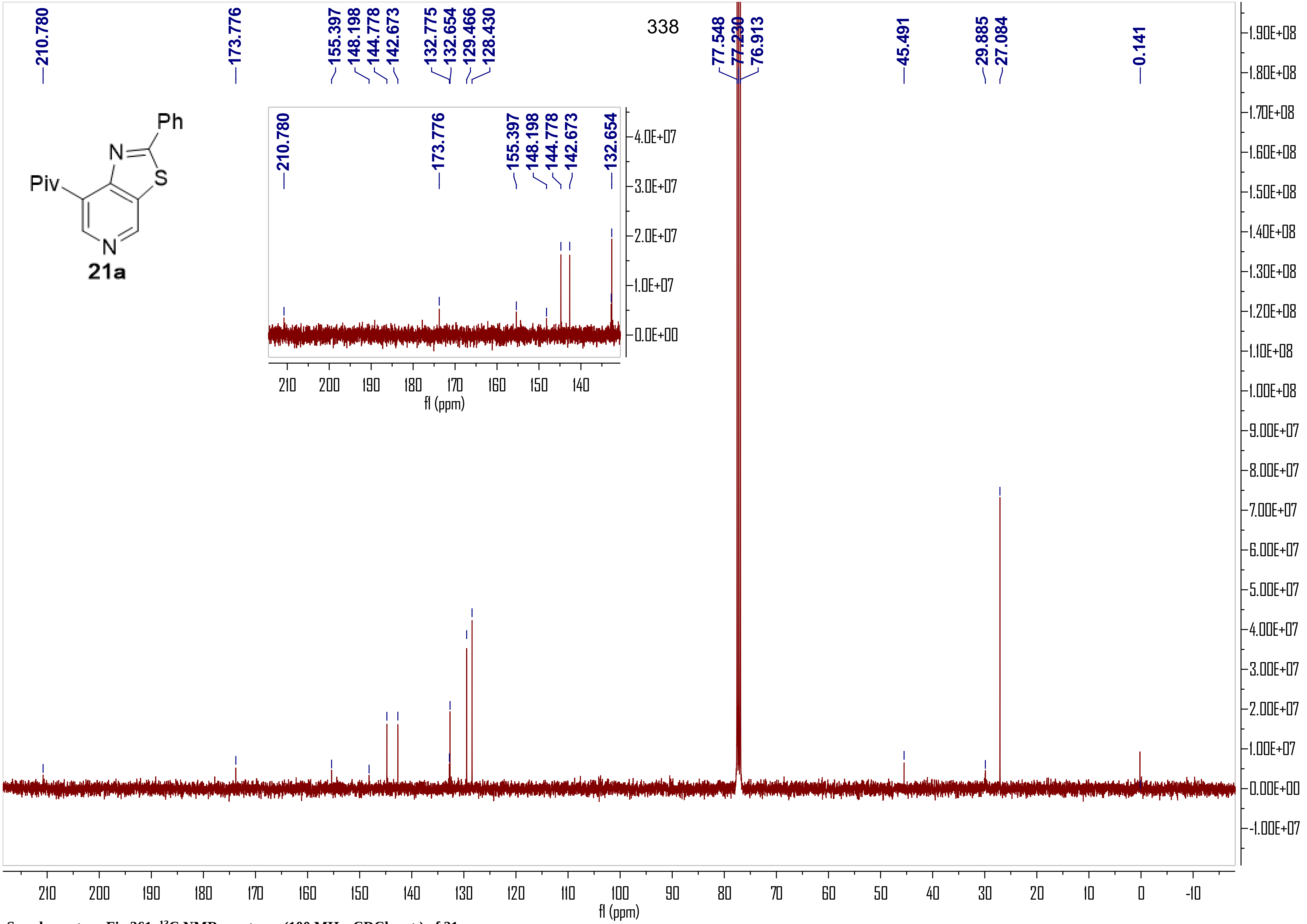

Supplementary Fig 261. ^{13}C NMR spectrum (100 MHz, $CDCl_3$, r.t.) of 21a.

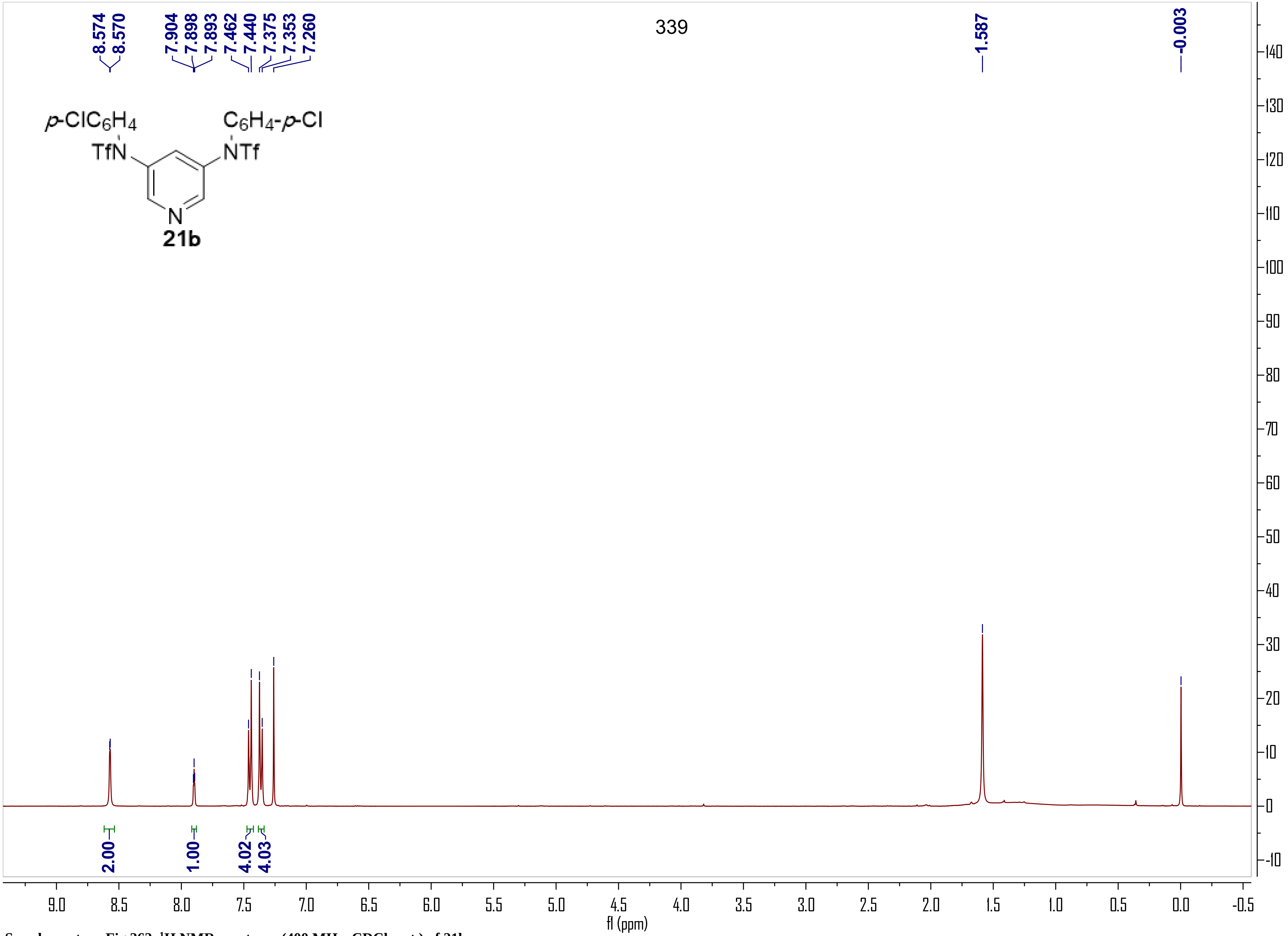

Supplementary Fig 262. ^{1}H NMR spectrum (400 MHz, $CDCl_3$, r.t.) of 21b.

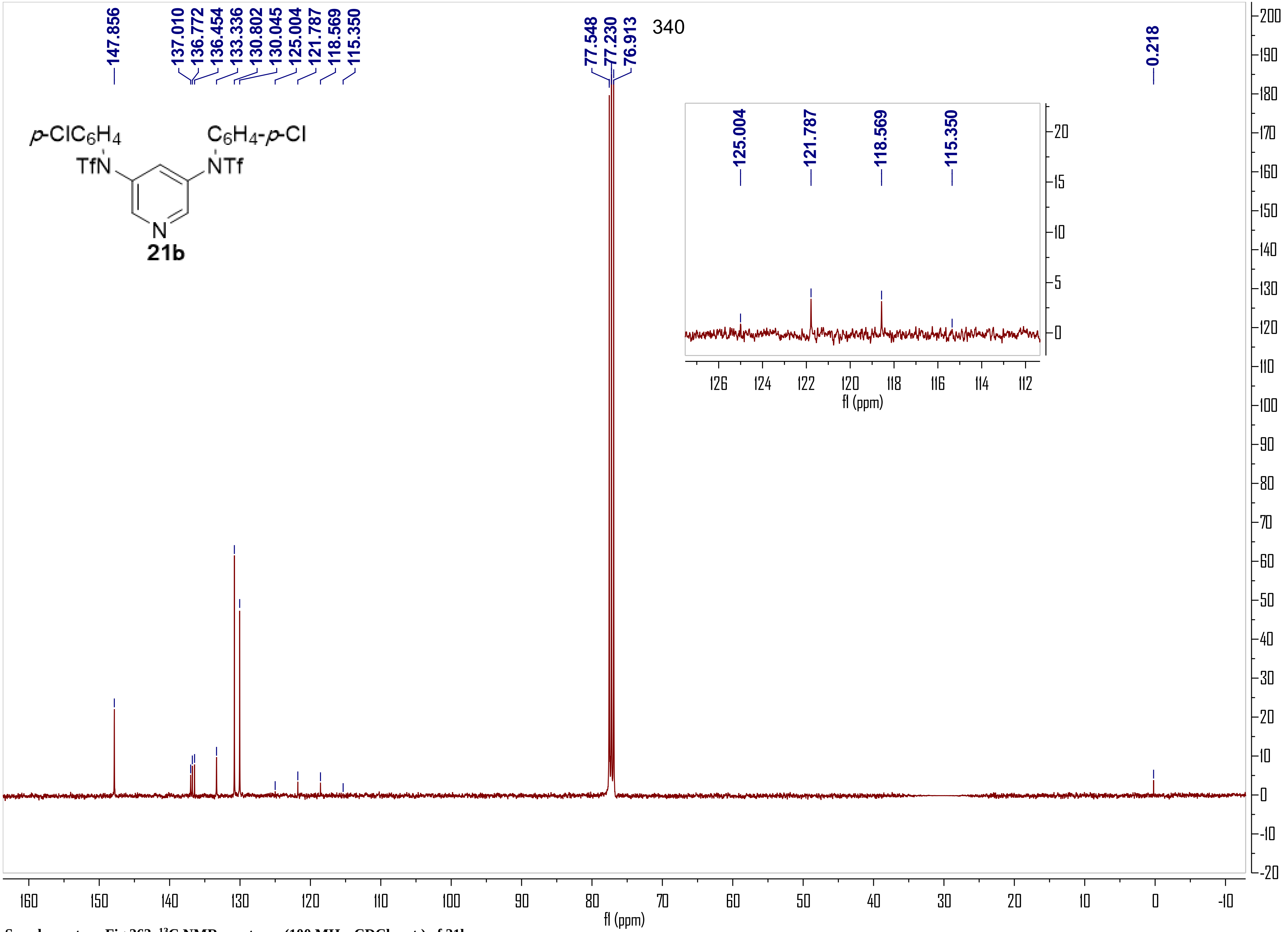

Supplementary Fig 263. ^{13}C NMR spectrum (100 MHz, $CDCl_3$, r.t.) of 21b.

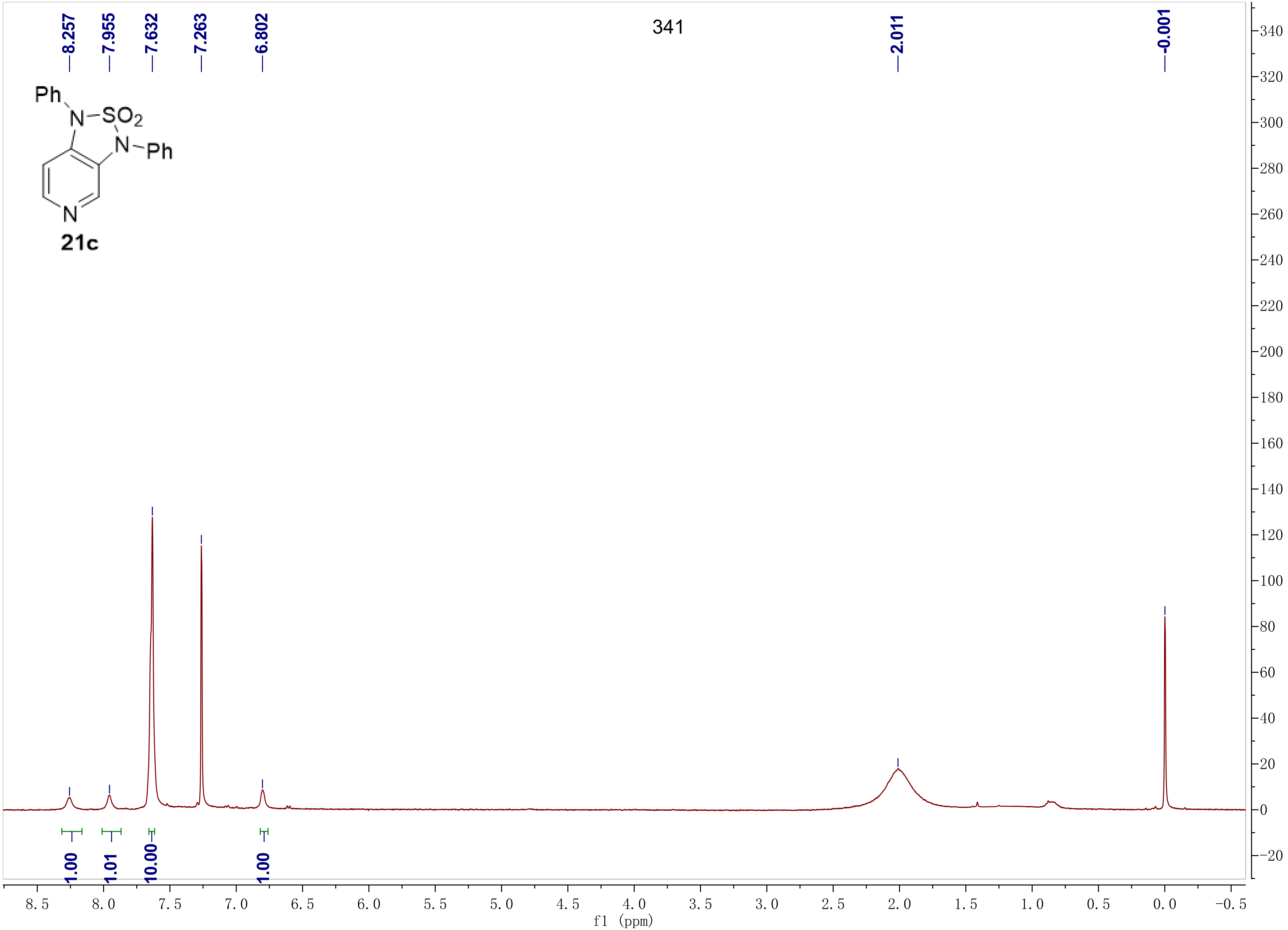

21c

Supplementary Fig 264. **^{1}H NMR spectrum (400 MHz, $CDCl_3$, r.t.) of 21c.**

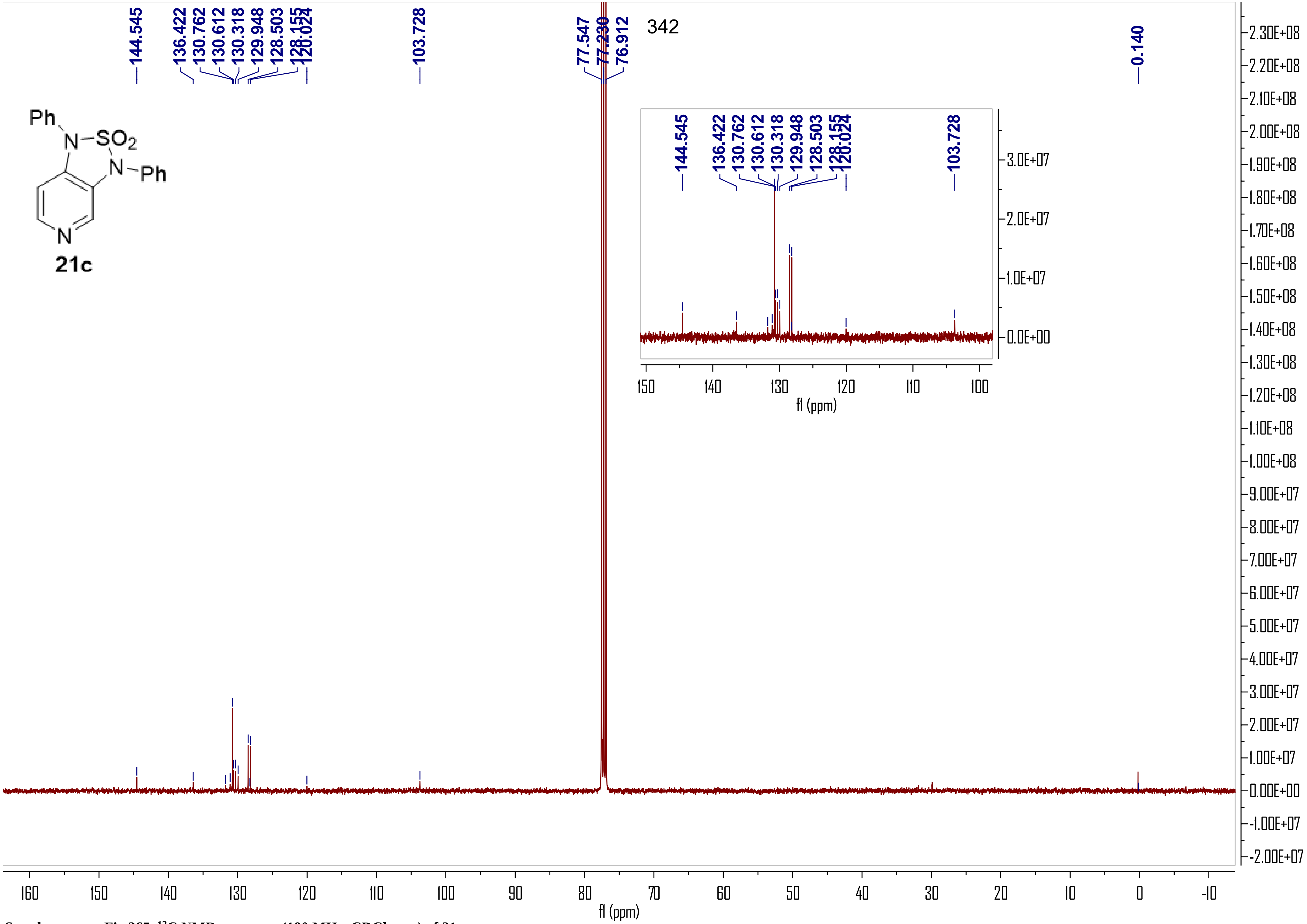

Supplementary Fig 265. ^{13}C NMR spectrum (100 MHz, $CDCl_3$, r.t.) of 21c.

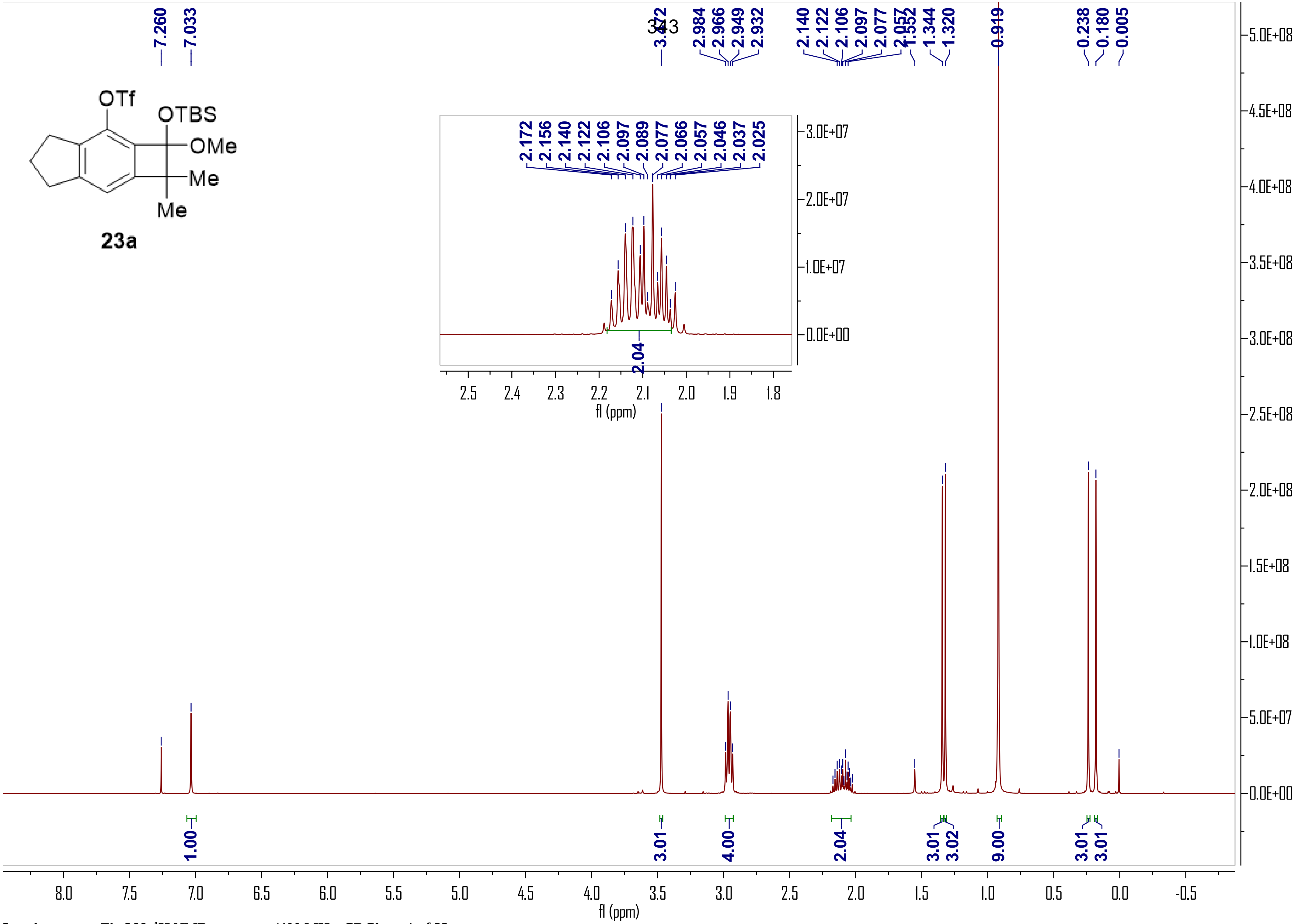

Supplementary Fig 266. ^{1}H **NMR spectrum (400 MHz,** $CDCl_3$**, r.t.) of 23a.**

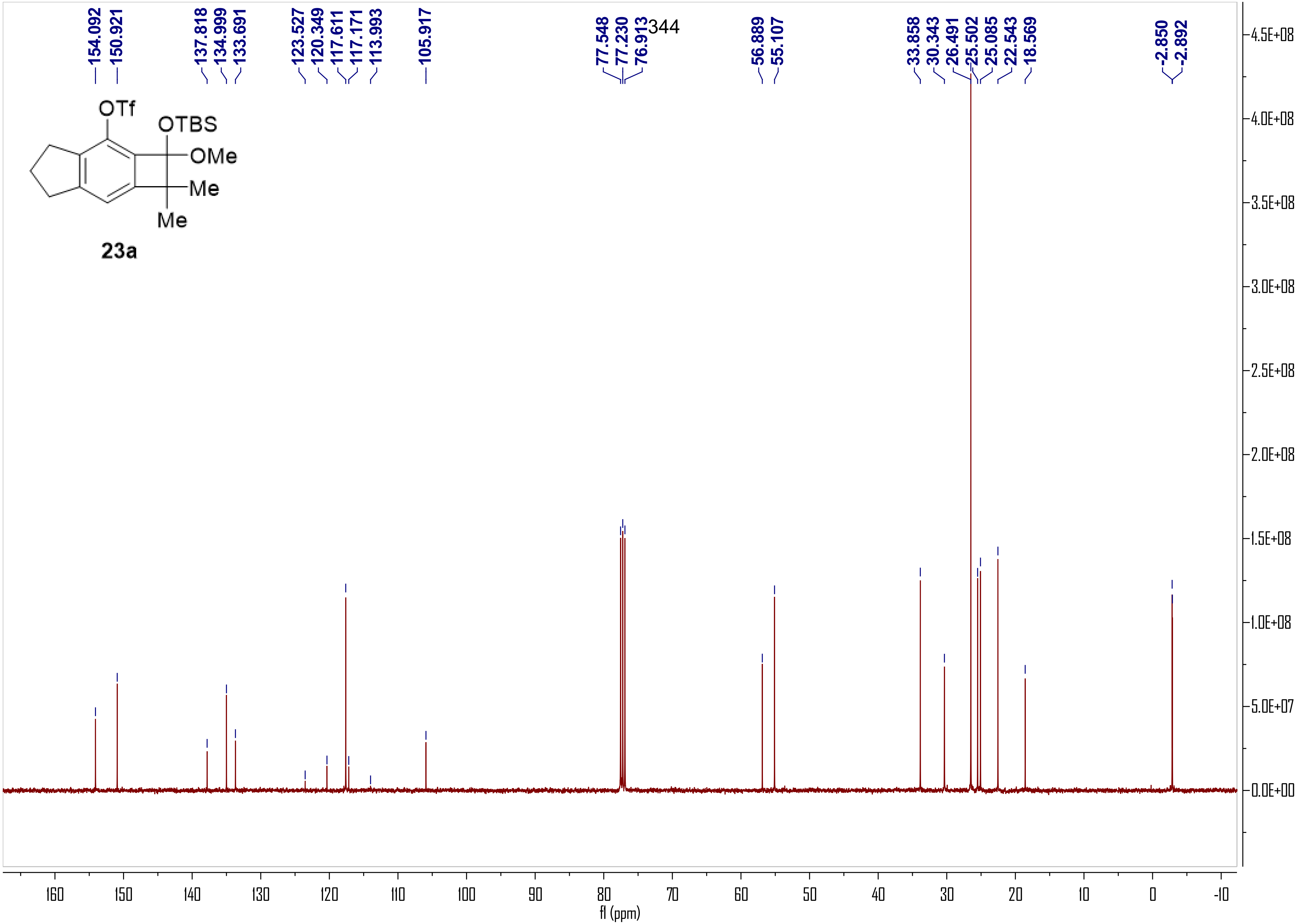

Supplementary Fig 267. ^{13}C NMR spectrum (100 MHz, $CDCl_3$, r.t.) of 23a.

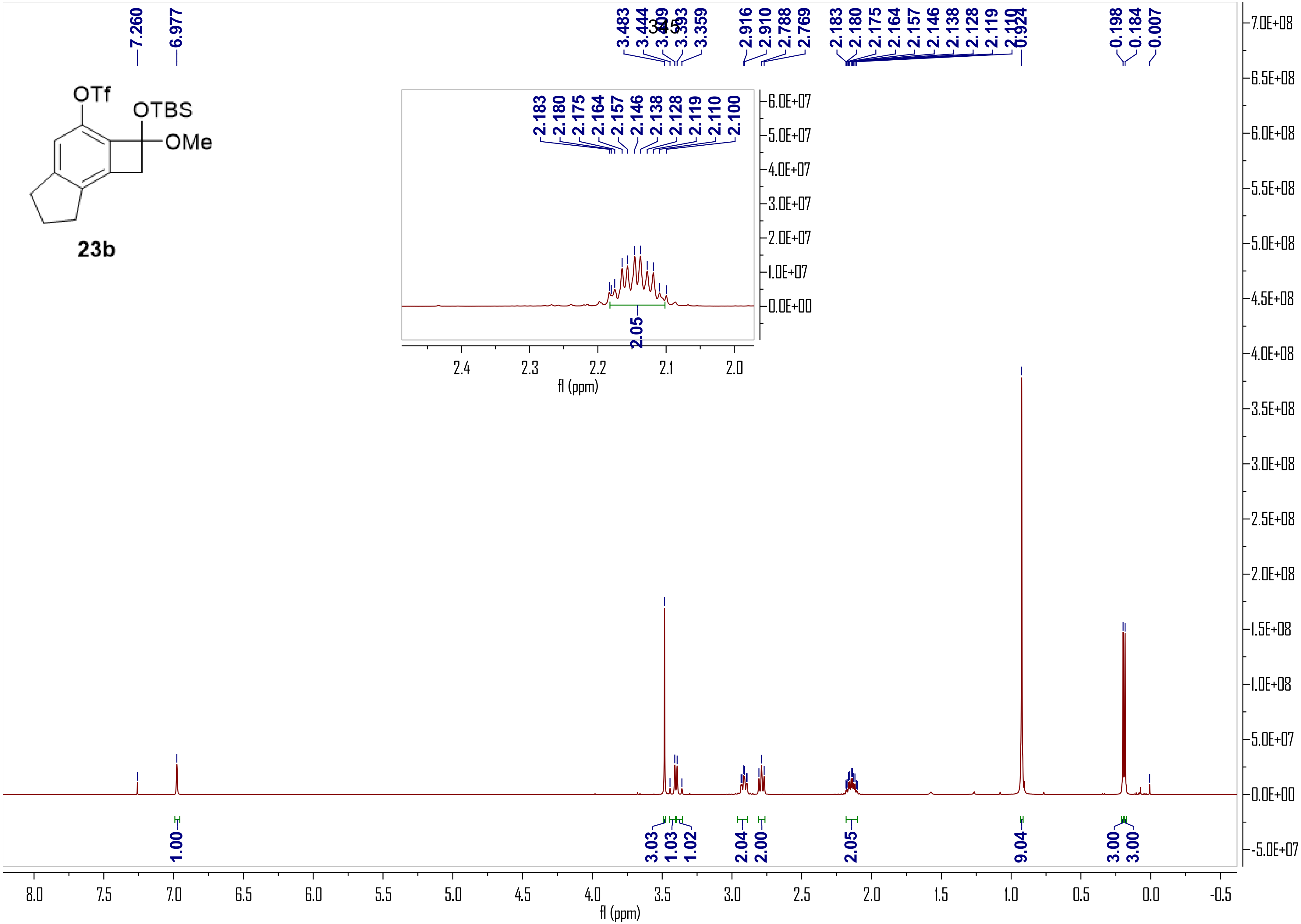

Supplementary Fig 268. **^{1}H NMR spectrum (400 MHz, $CDCl_3$, r.t.) of 23b.**

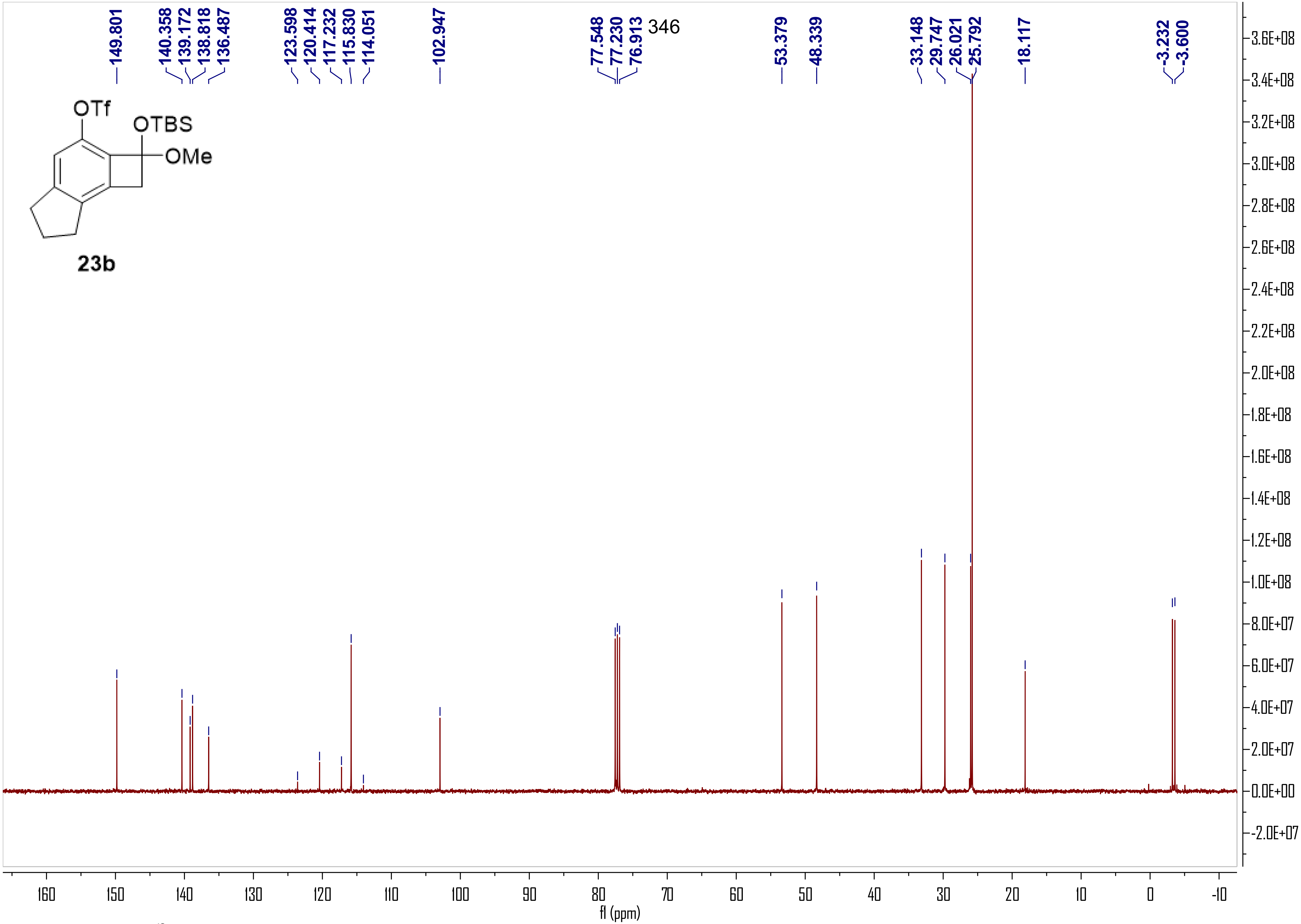

Supplementary Fig 269. **^{13}C NMR spectrum (100 MHz, $CDCl_3$, r.t.) of 23b.**

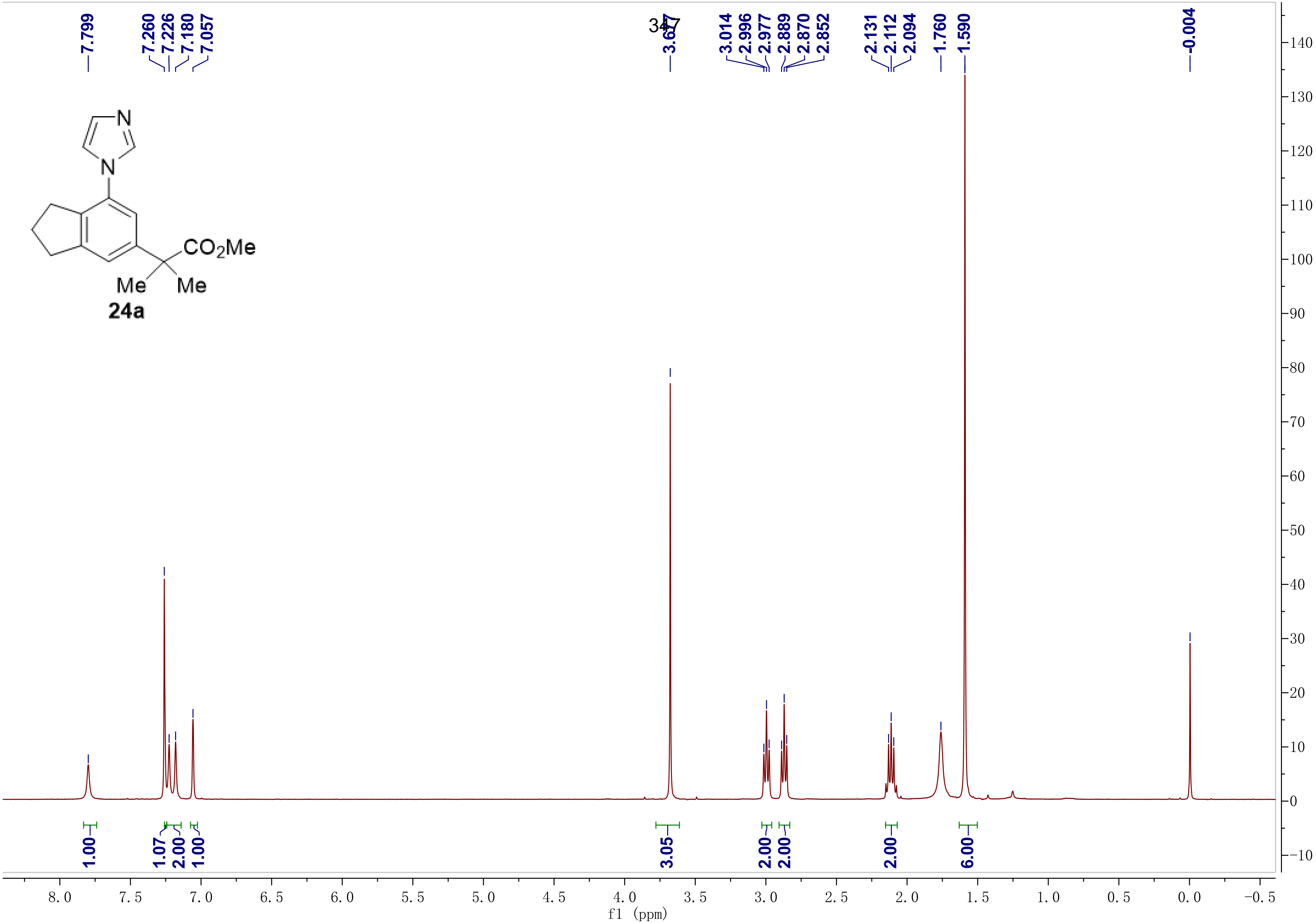

Supplementary Fig 270. ^{1}H NMR spectrum (400 MHz, $CDCl_3$, r.t.) of 24a.

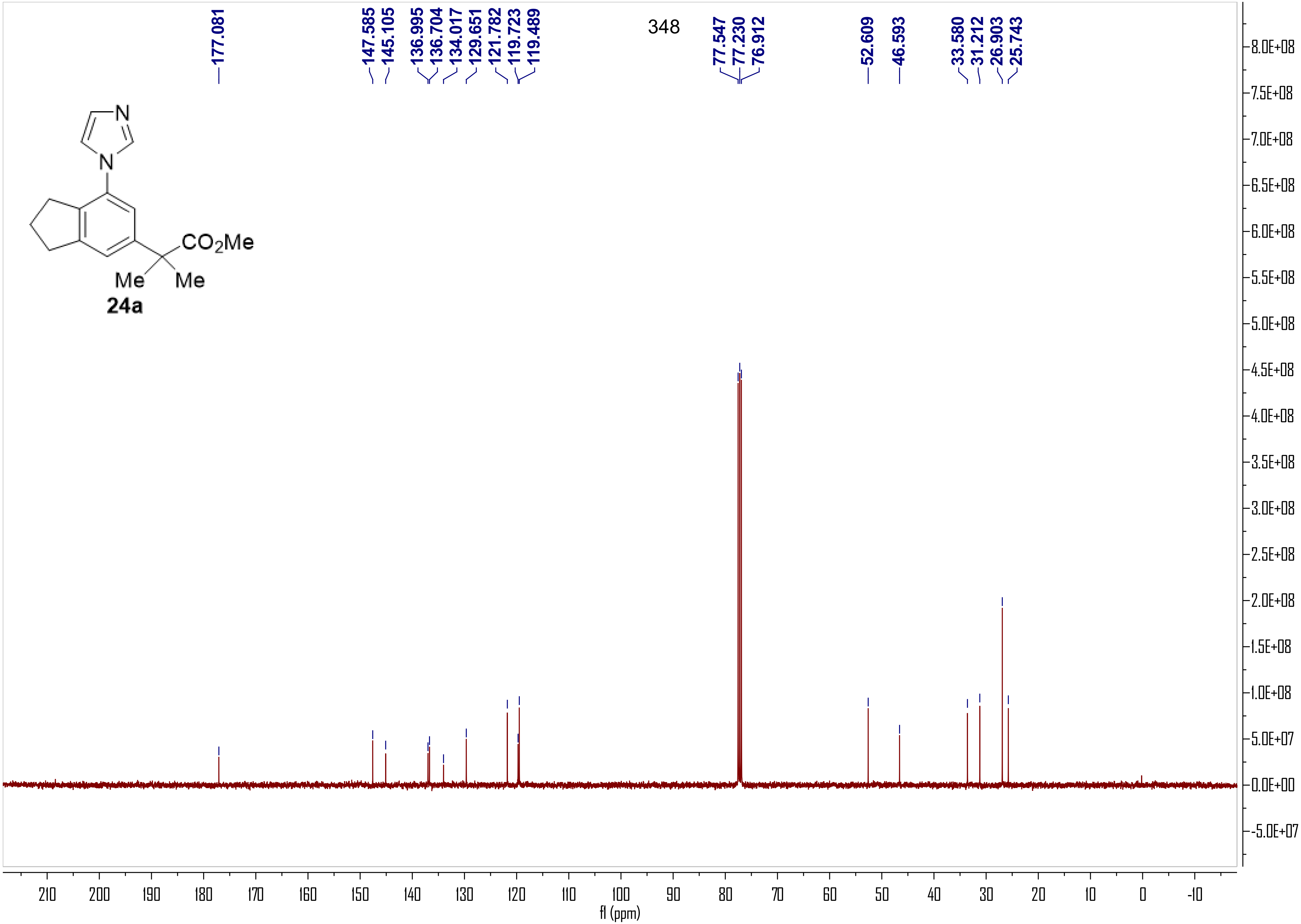

Supplementary Fig 271. **^{13}C NMR spectrum (100 MHz, $CDCl_3$, r.t.) of 24a.**

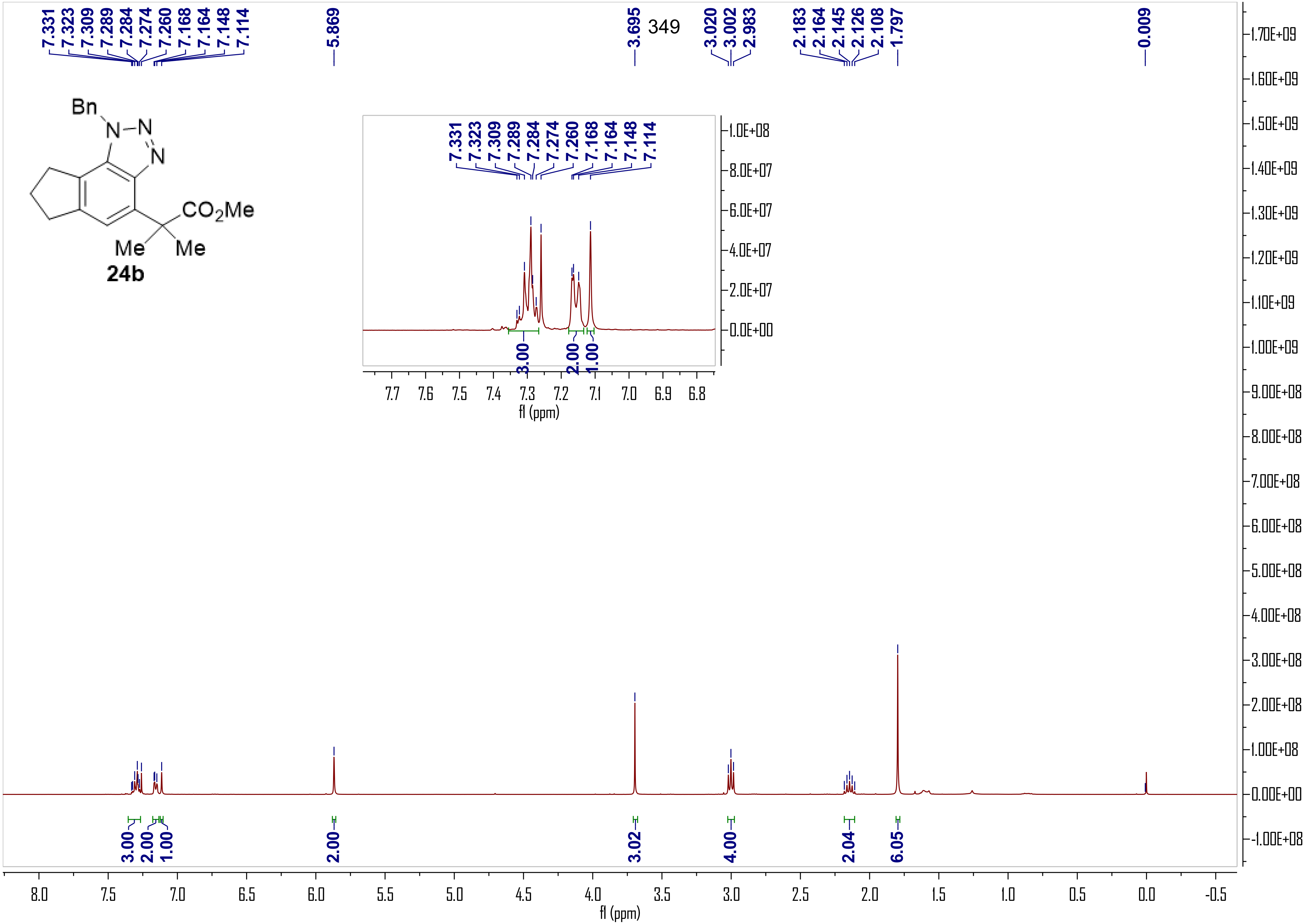

Supplementary Fig 272. ¹H NMR spectrum (400 MHz, CDCl₃, r.t.) of 24b.

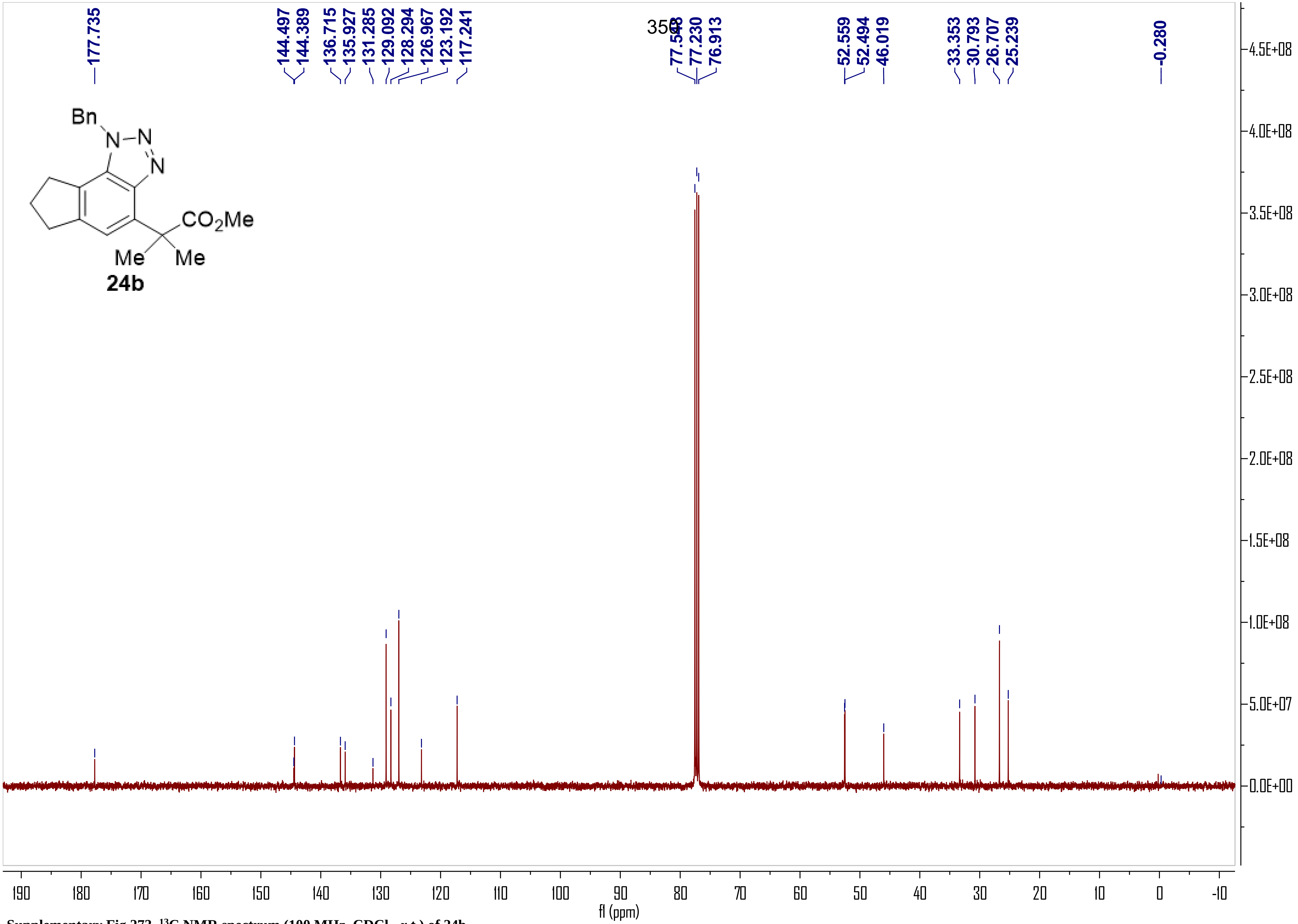

Supplementary Fig 273. ^{13}C **NMR spectrum (100 MHz,** $CDCl_3$**, r.t.) of 24b.**

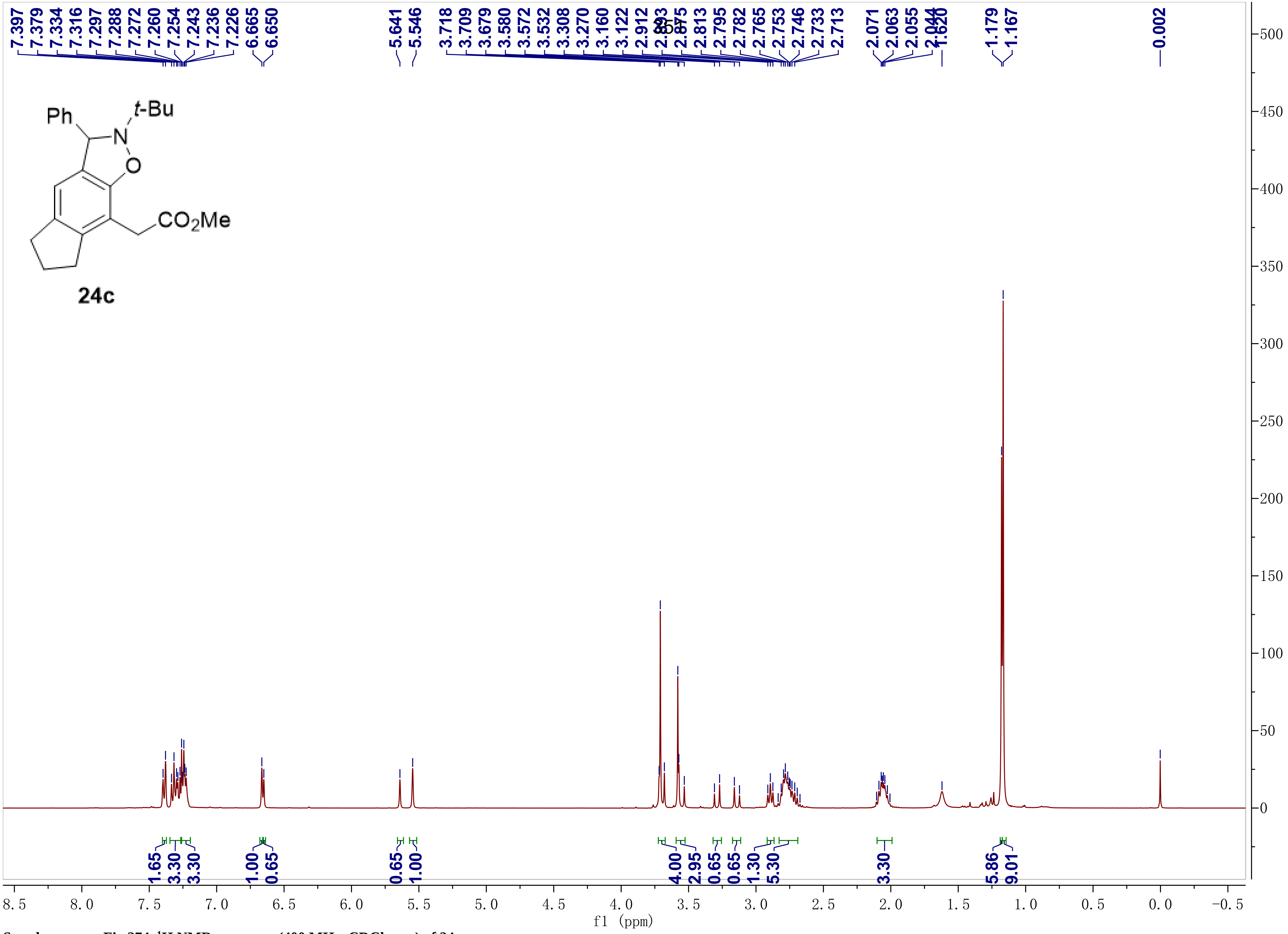

Supplementary Fig 274. ^{1}H NMR spectrum (400 MHz, $CDCl_3$, r.t.) of 24c.

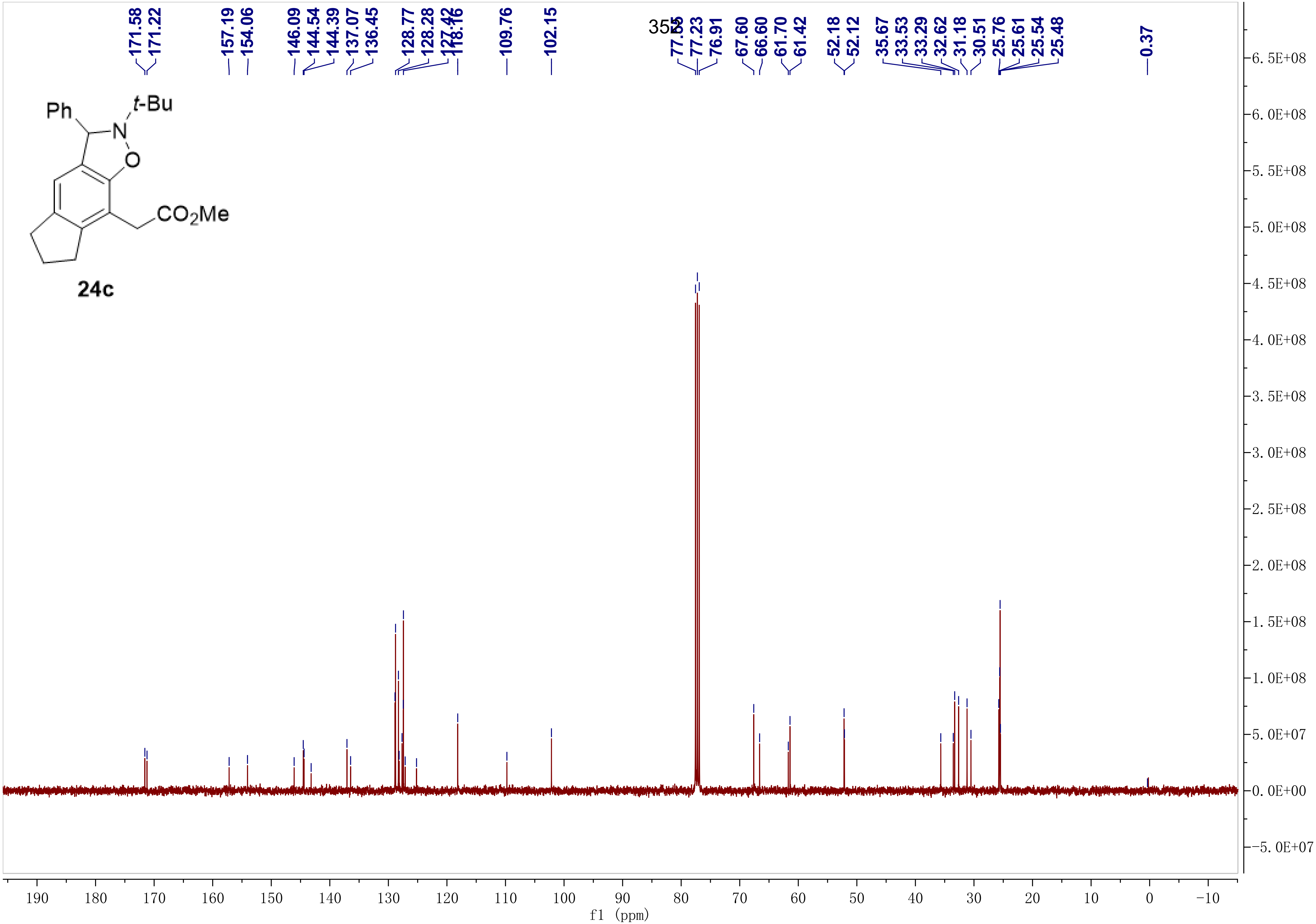

Supplementary Fig 275. ^{13}C NMR spectrum (100 MHz, $CDCl_3$, r.t.) of 24c.

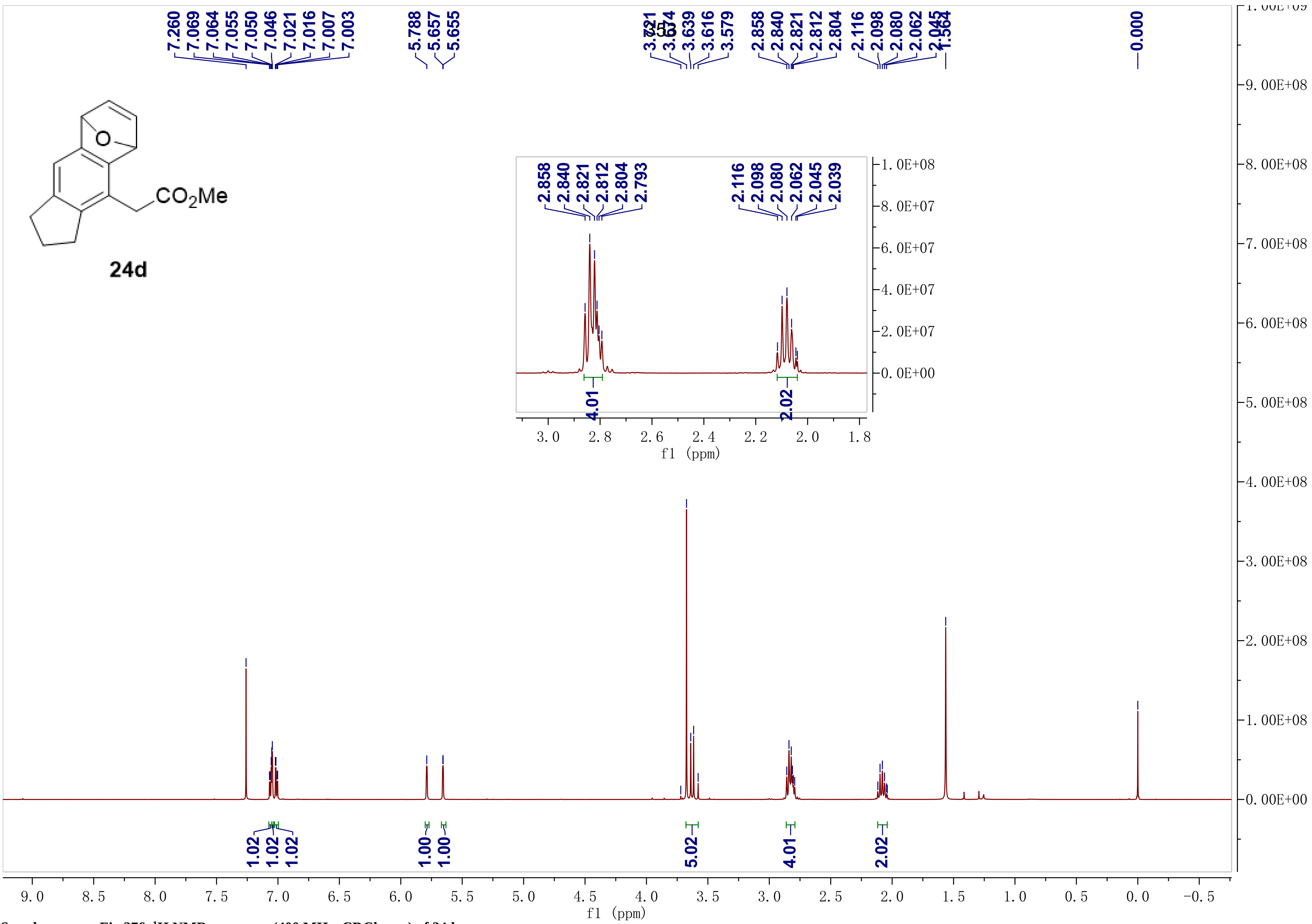

Supplementary Fig 276. ^{1}H NMR spectrum (400 MHz, $CDCl_3$, r.t.) of 24d.

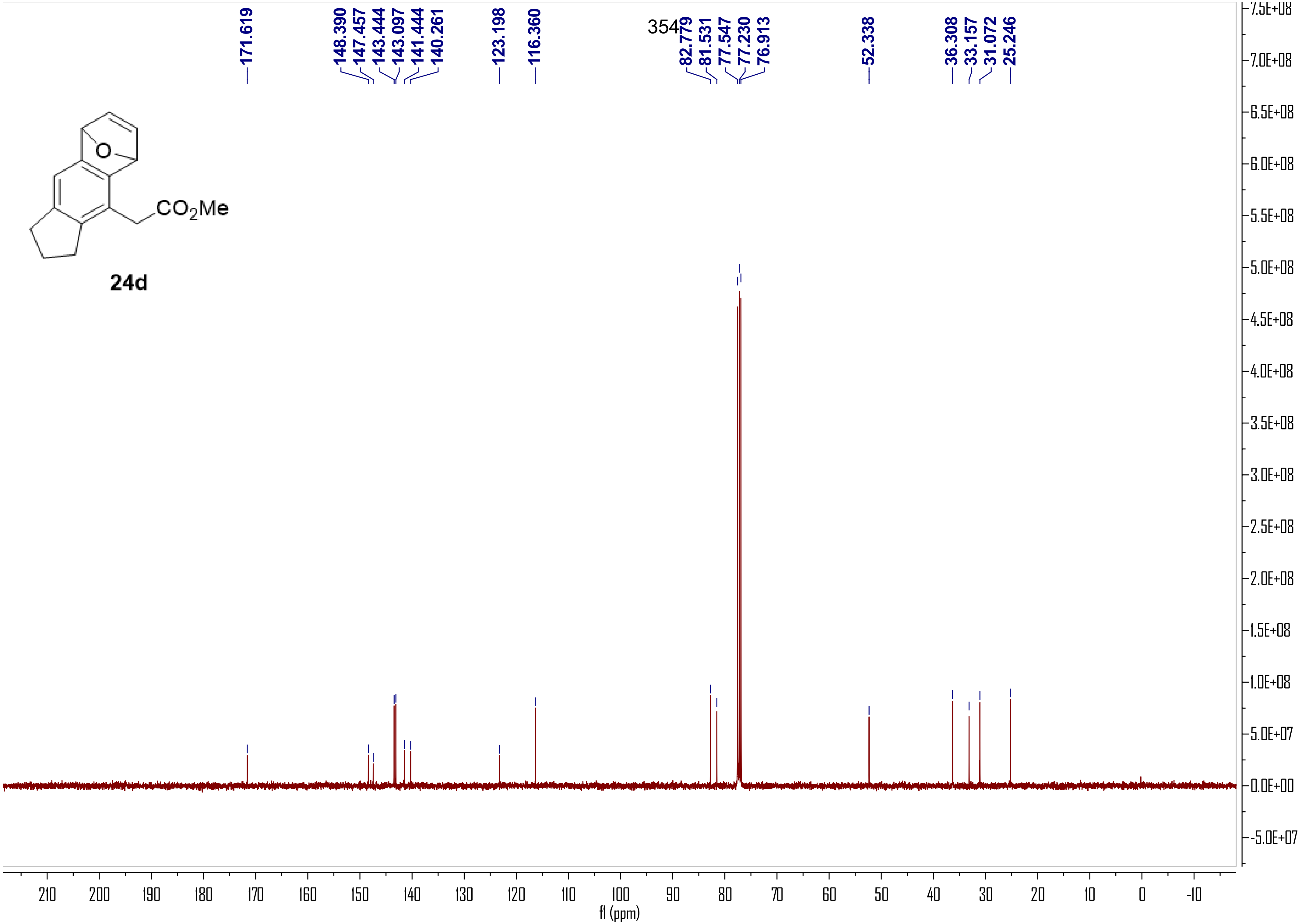

Supplementary Fig 277. **^{13}C NMR spectrum (100 MHz, $CDCl_3$, r.t.) of 24d.**

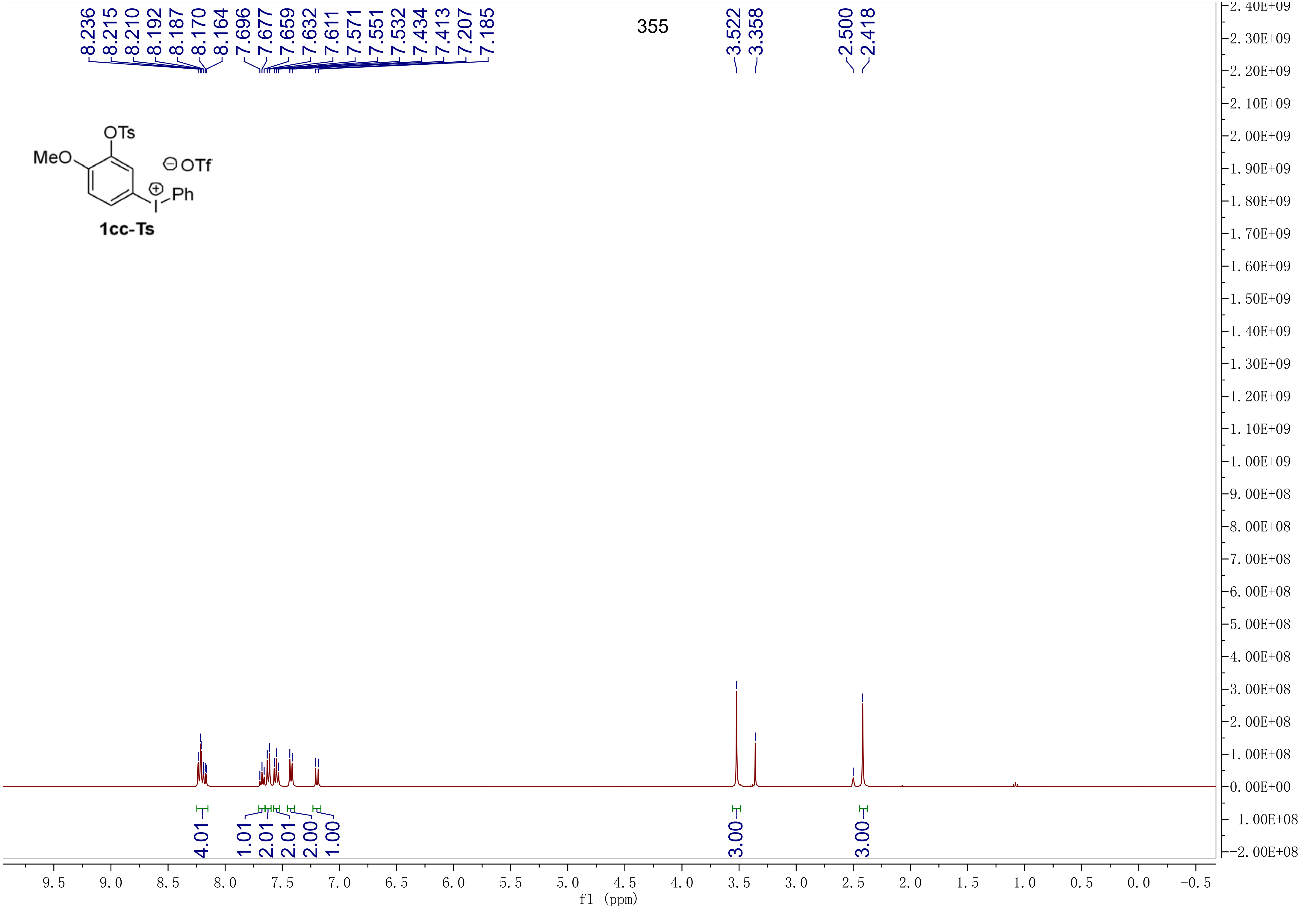

Supplementary Fig 278 ¹H NMR spectrum (400 MHz, DMSO-*d*6, r.t.) of 1cc-Ts.

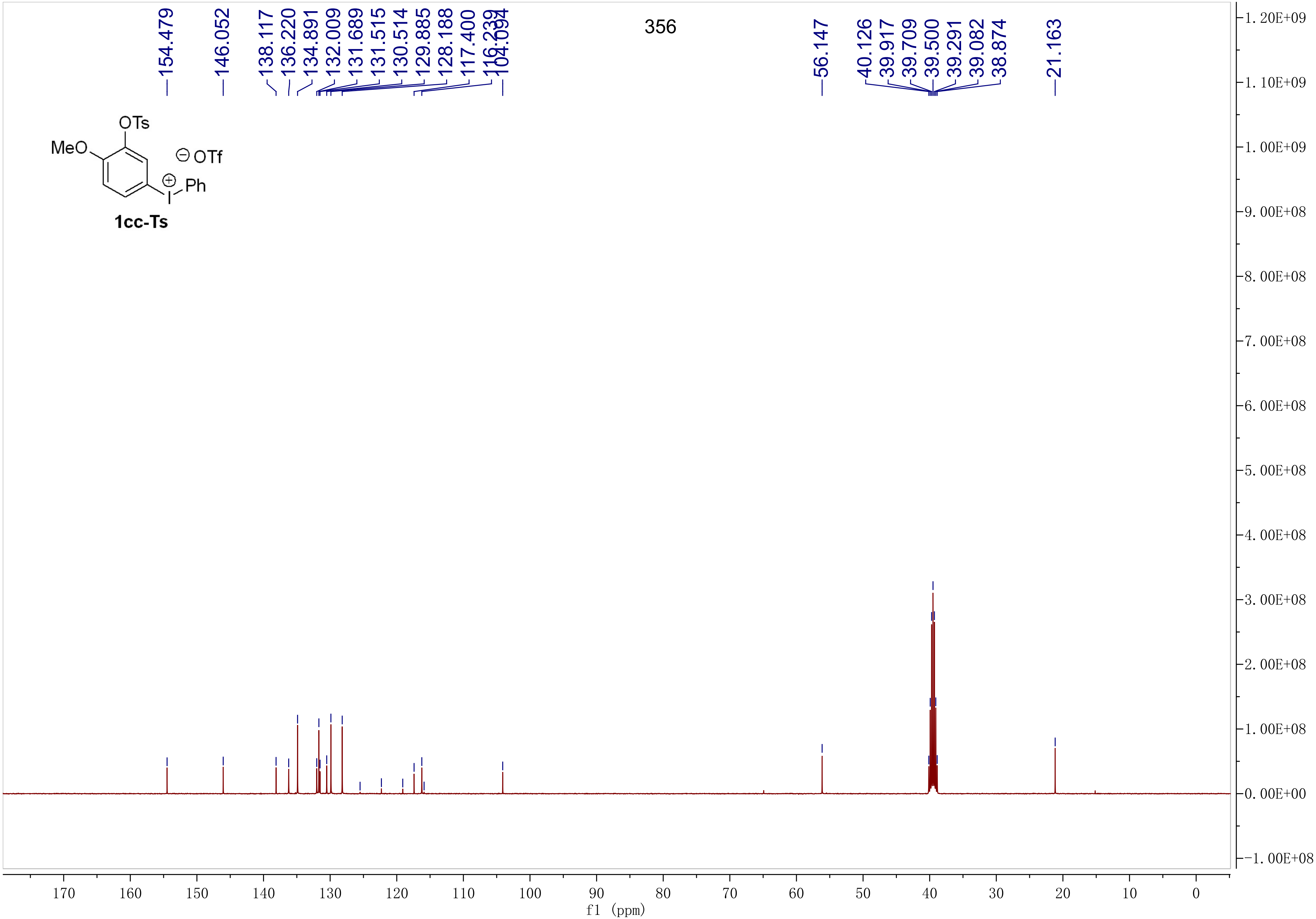

Supplementary Fig 279 ^{13}C NMR spectrum (100 MHz, DMSO-*d*6, r.t.) of 1cc-Ts.

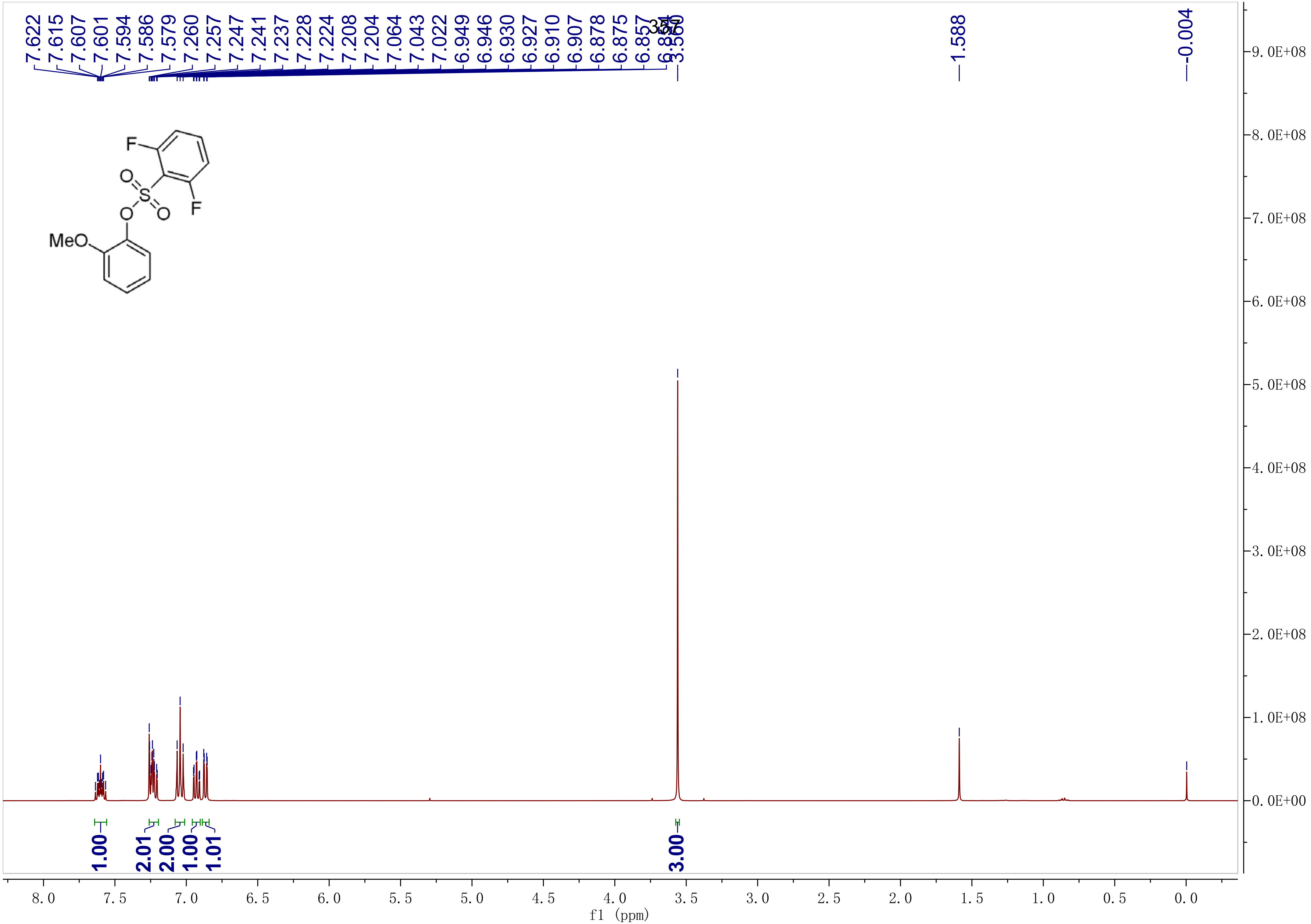

Supplementary Fig 280 ^{1}H NMR spectrum (400 MHz, $CDCl_3$, r.t.) of 2-methoxyphenyl 2,6-difluorobenzenesulfonate.

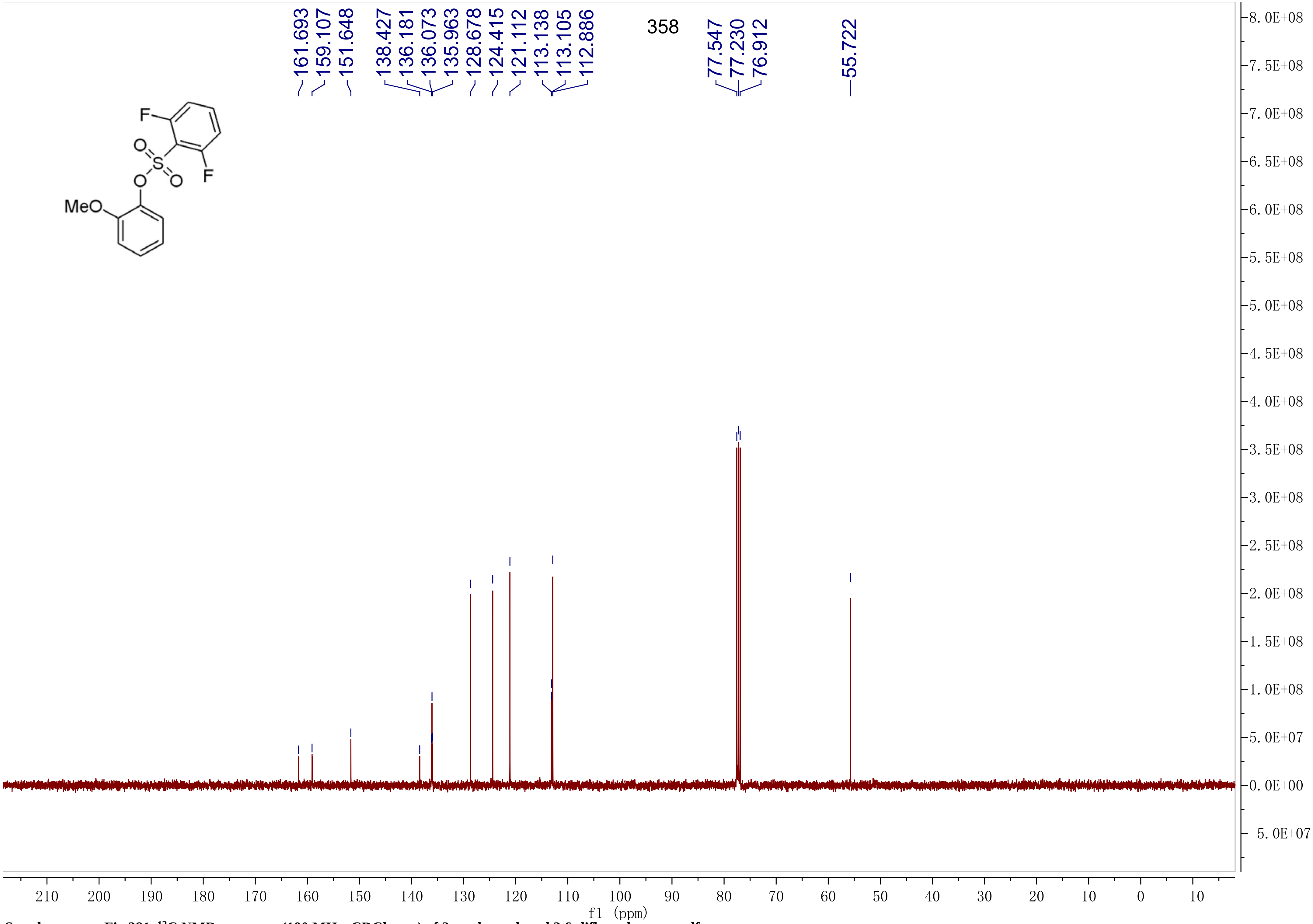

Supplementary Fig 281. ^{13}C NMR spectrum (100 MHz, $CDCl_3$, r.t.) of 2-methoxyphenyl 2,6-difluorobenzenesulfonate.

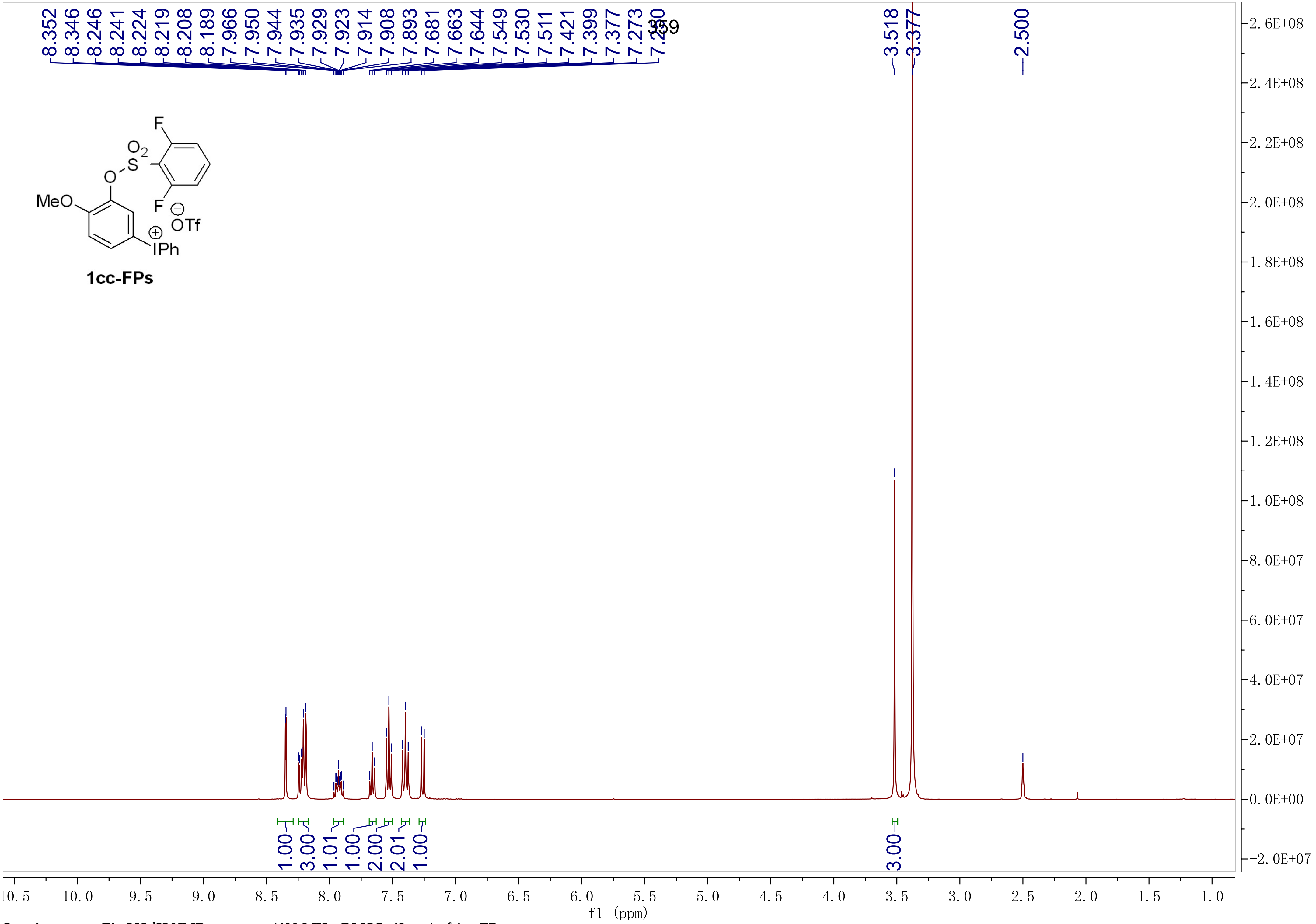

Supplementary Fig 282 ^{1}H NMR spectrum (400 MHz, DMSO-*d6*, r.t.) of 1cc-FPs.

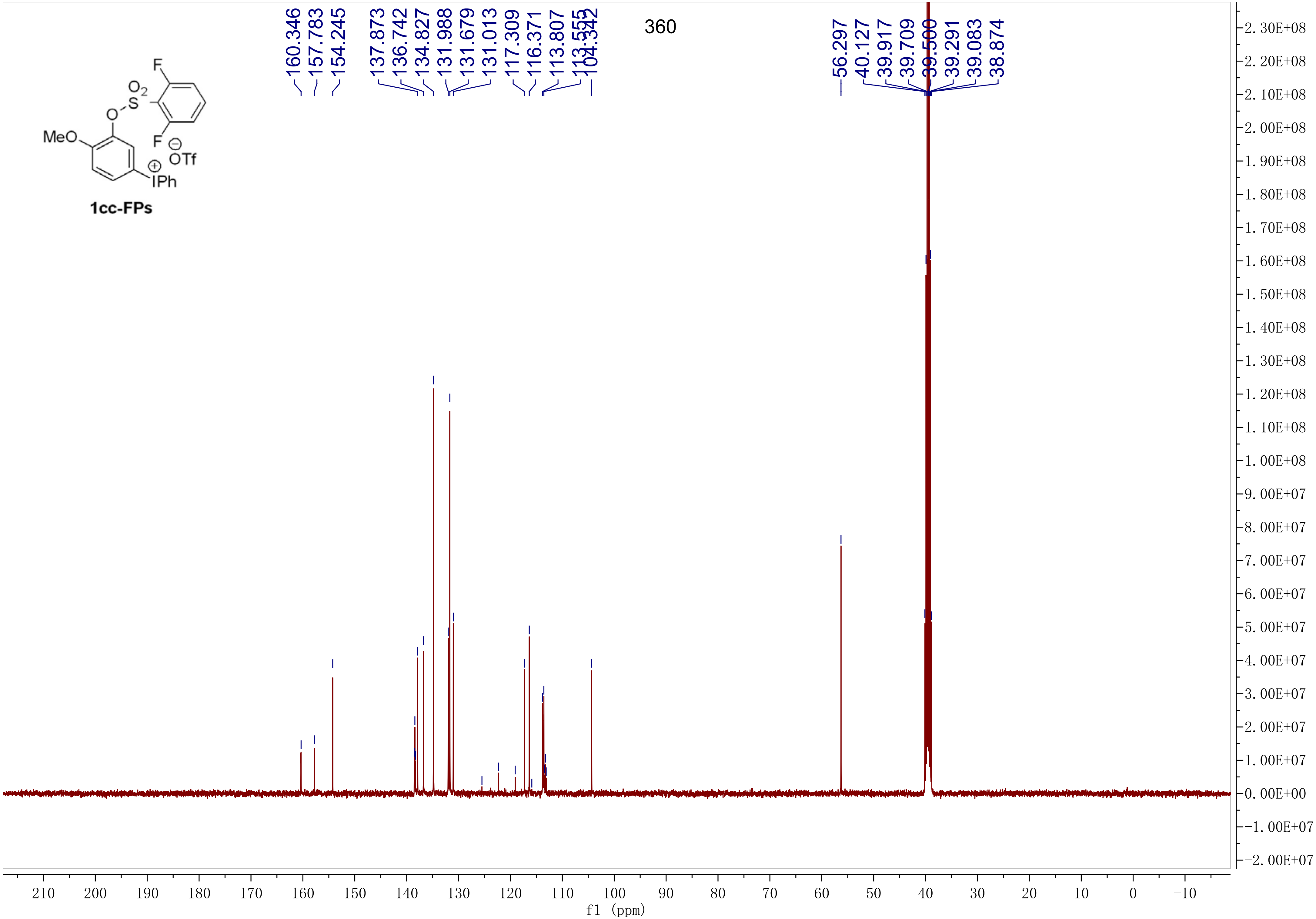

Supplementary Fig 283 ^{13}C NMR spectrum (100 MHz, DMSO-*d*6, r.t.) of 1cc-FPs.

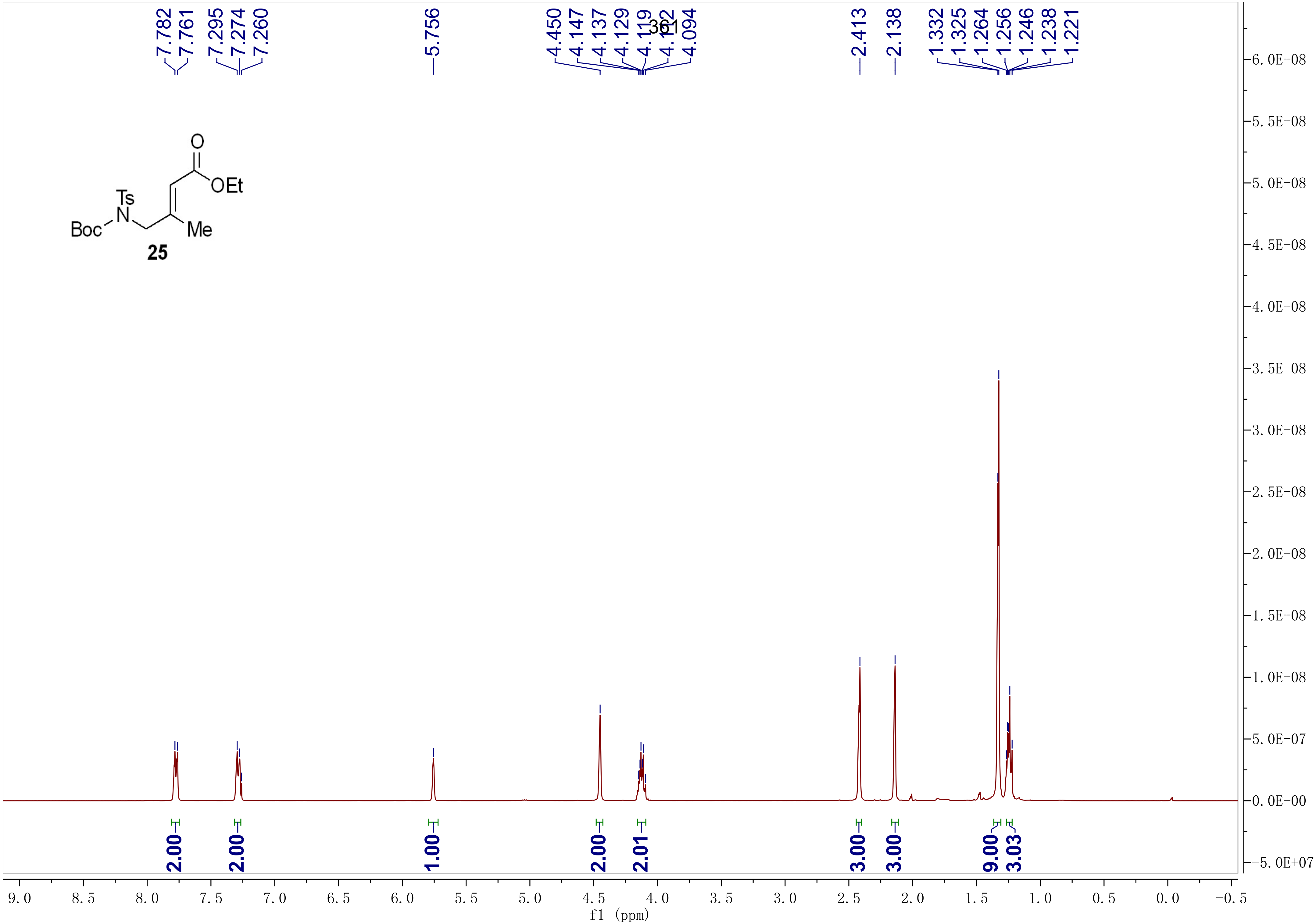

Supplementary Fig 284 ^{1}H NMR spectrum (400 MHz, $CDCl_3$, r.t.) of 25.

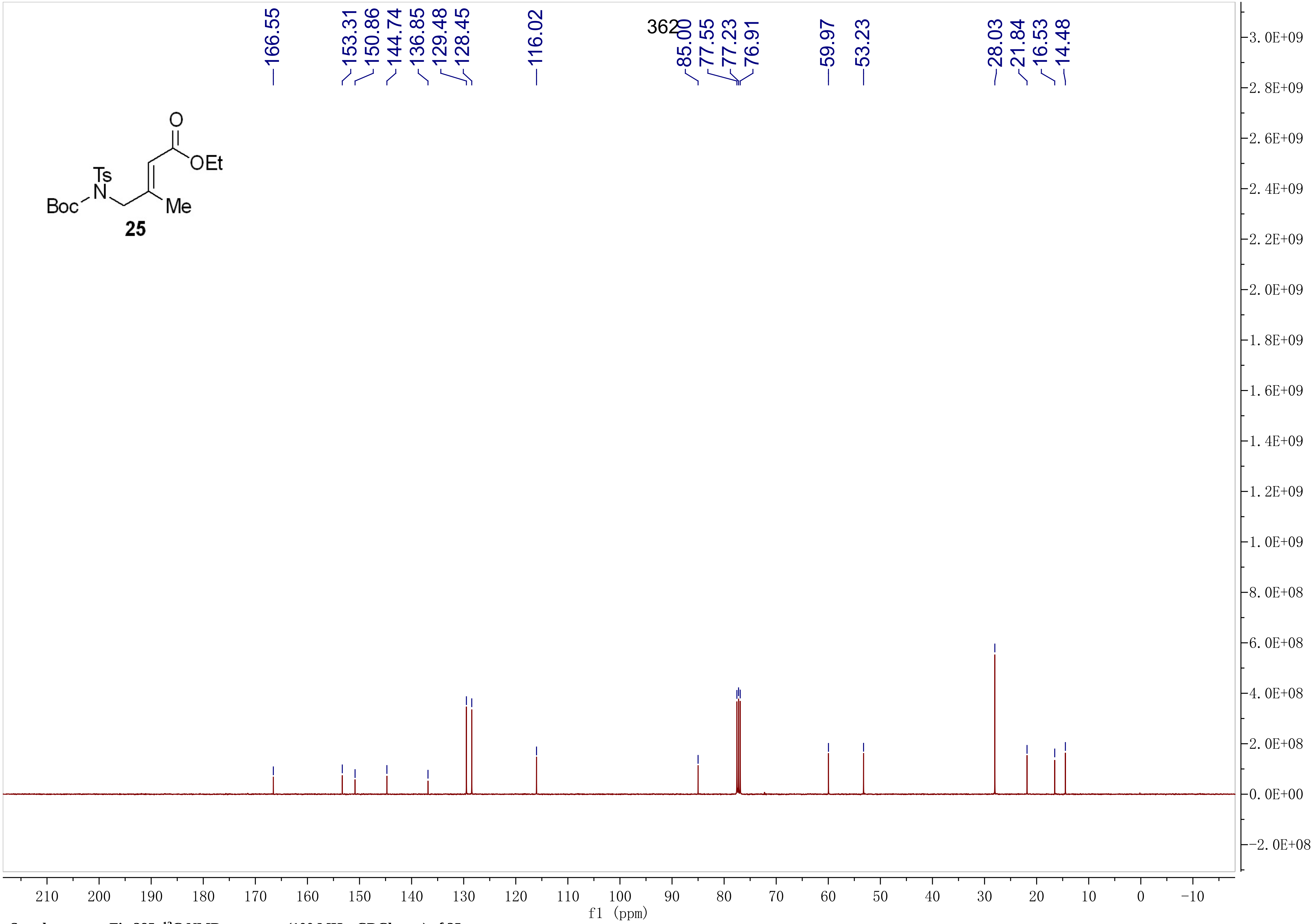

Supplementary Fig 285. ^{13}C **NMR spectrum (100 MHz,** $CDCl_3$**, r.t.) of 25.**

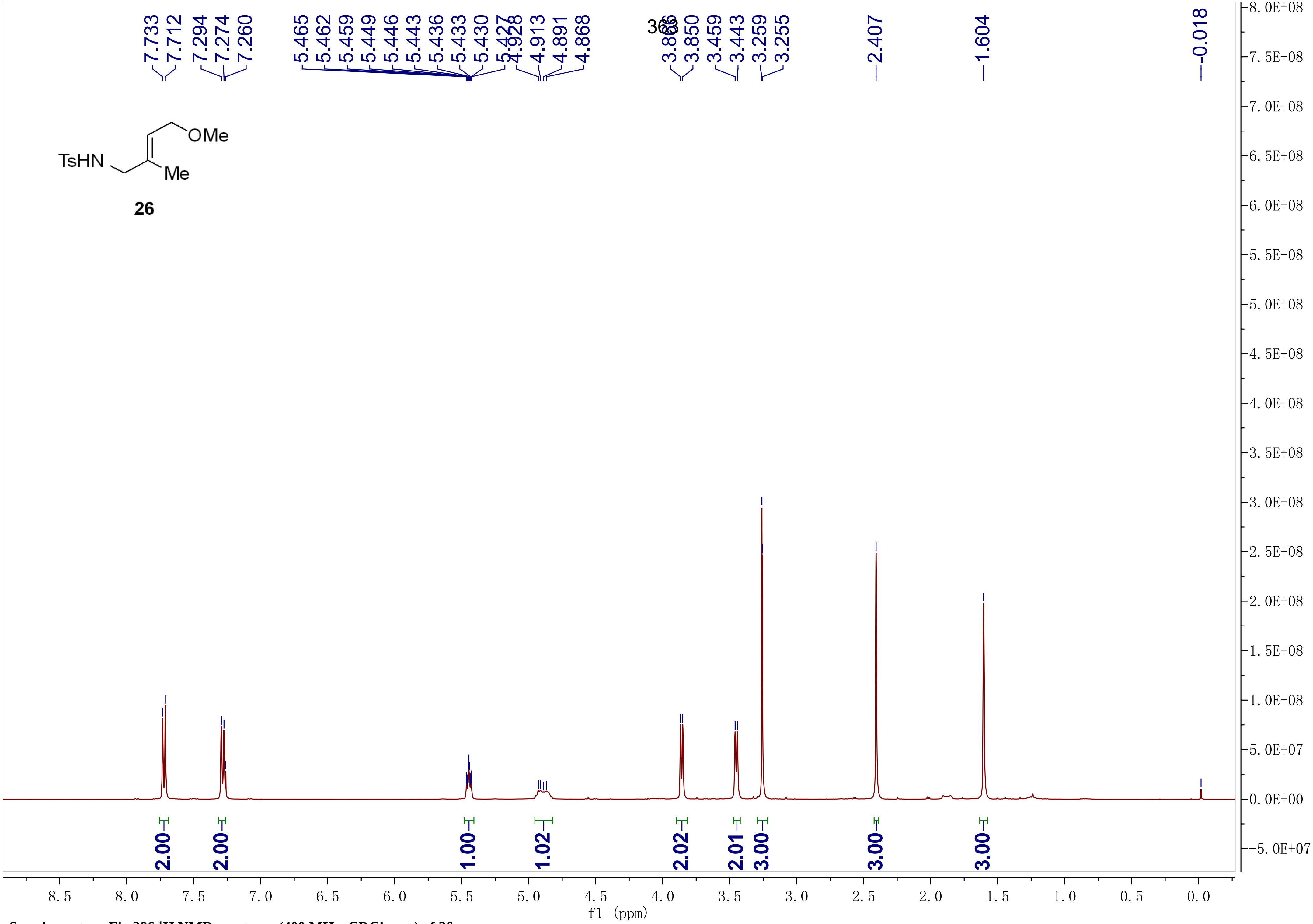

Supplementary Fig 286 ^{1}H NMR spectrum (400 MHz, $CDCl_3$, r.t.) of 26.

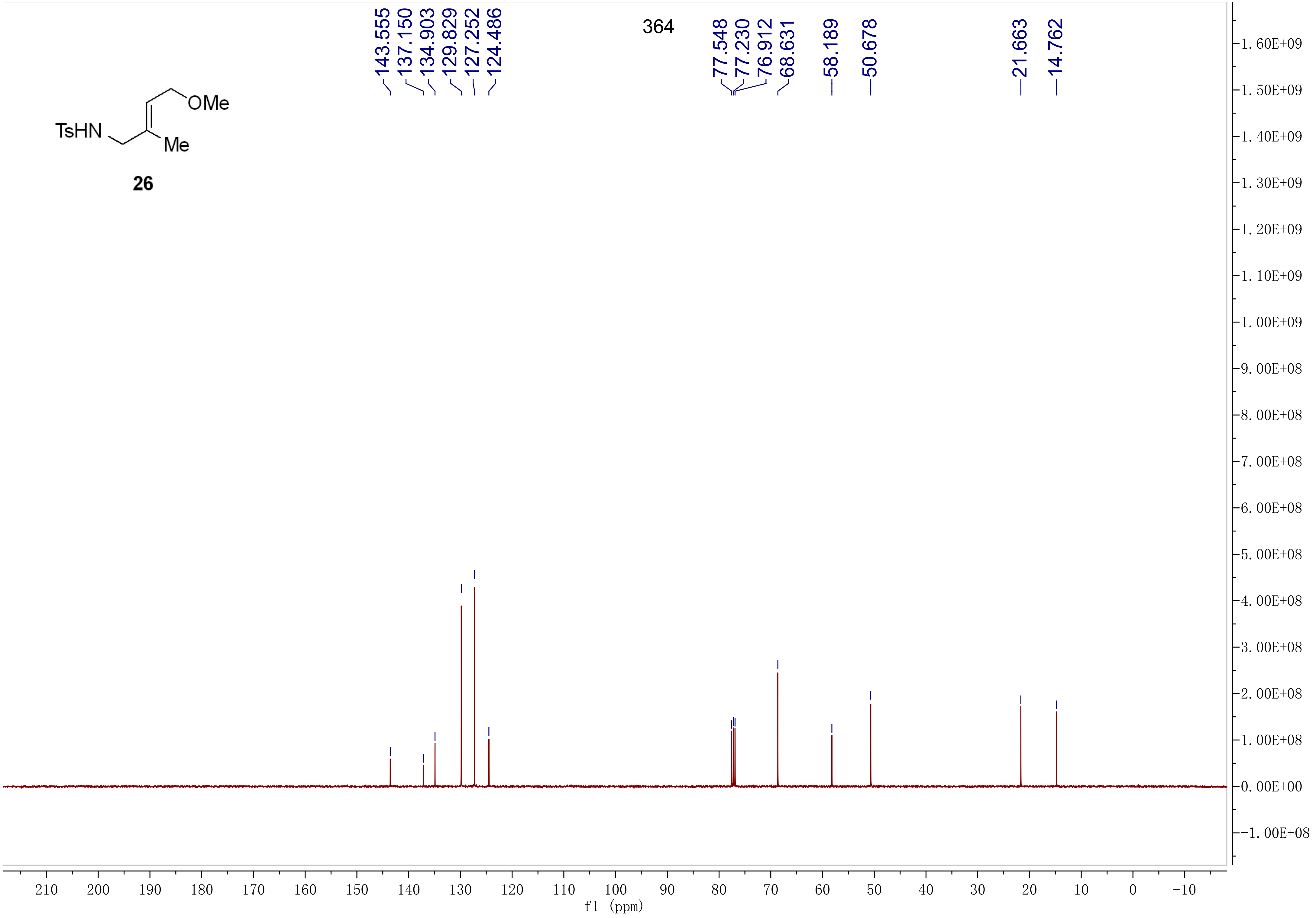

Supplementary Fig 287. ^{13}**C NMR spectrum (100 MHz,** $CDCl_3$**, r.t.) of 26.**

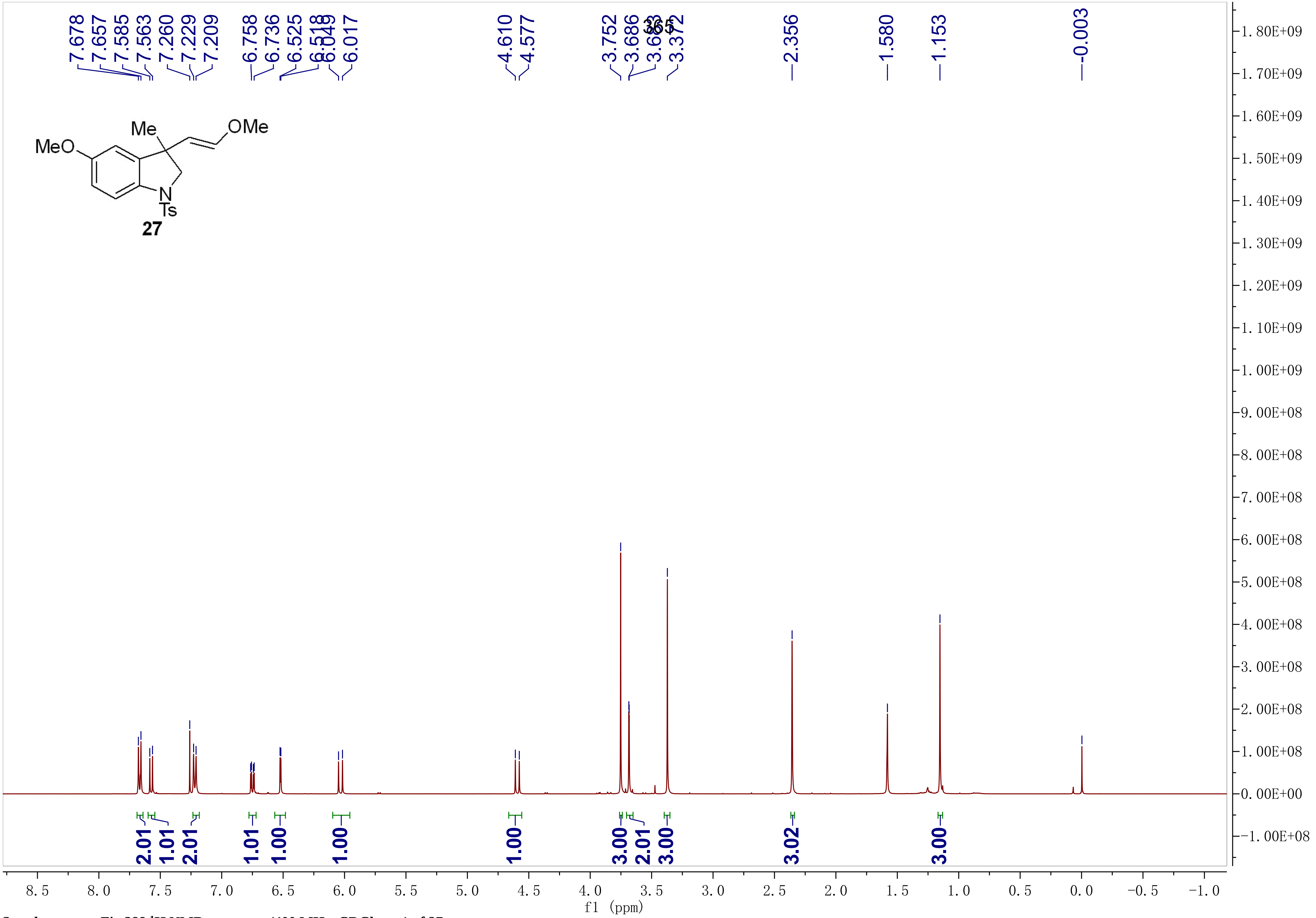

Supplementary Fig 288 ^{1}H NMR spectrum (400 MHz, $CDCl_3$, r.t.) of 27.

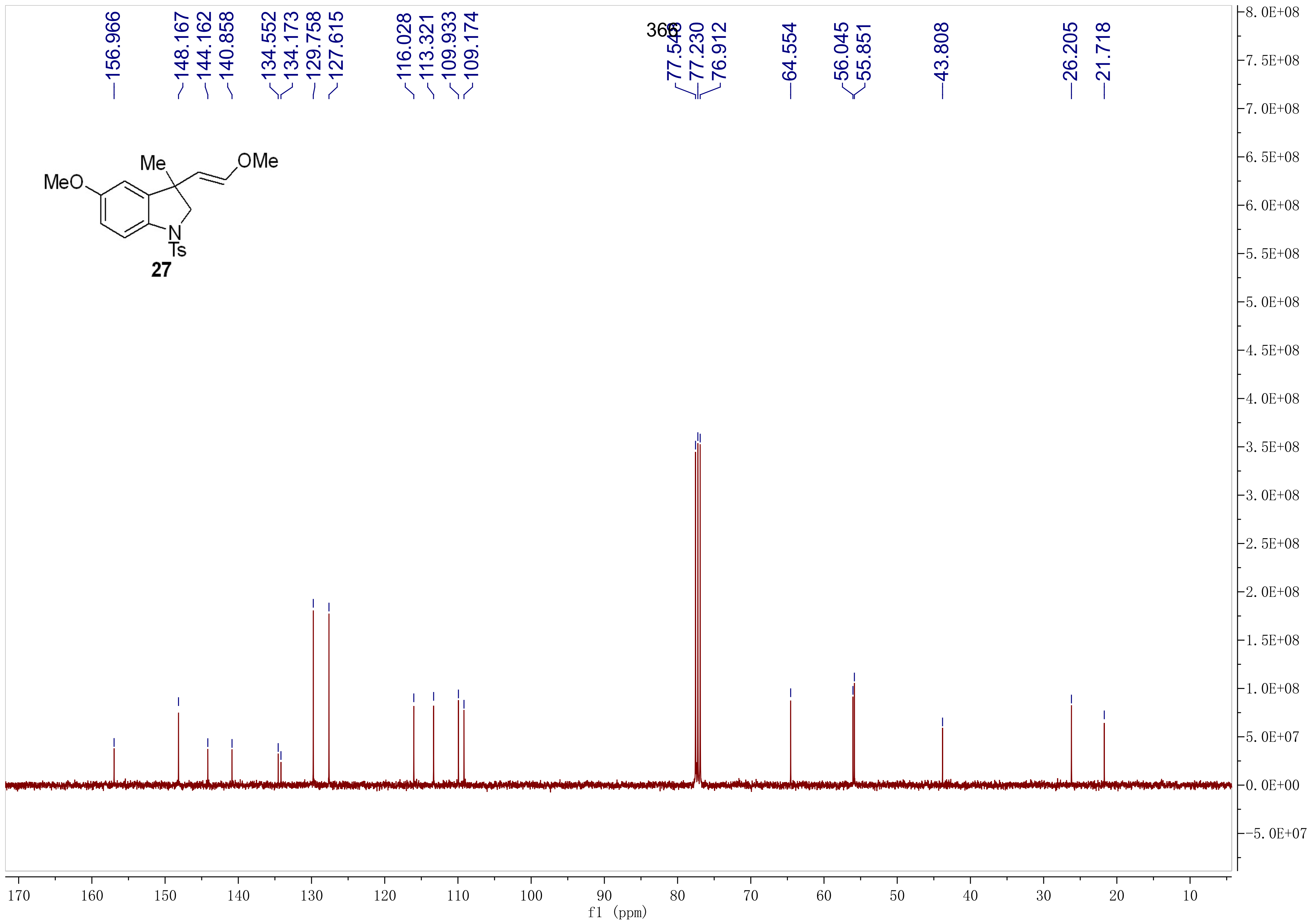

Supplementary Fig 289. ^{13}**C NMR spectrum (100 MHz, CDCl$_3$, r.t.) of 27.**

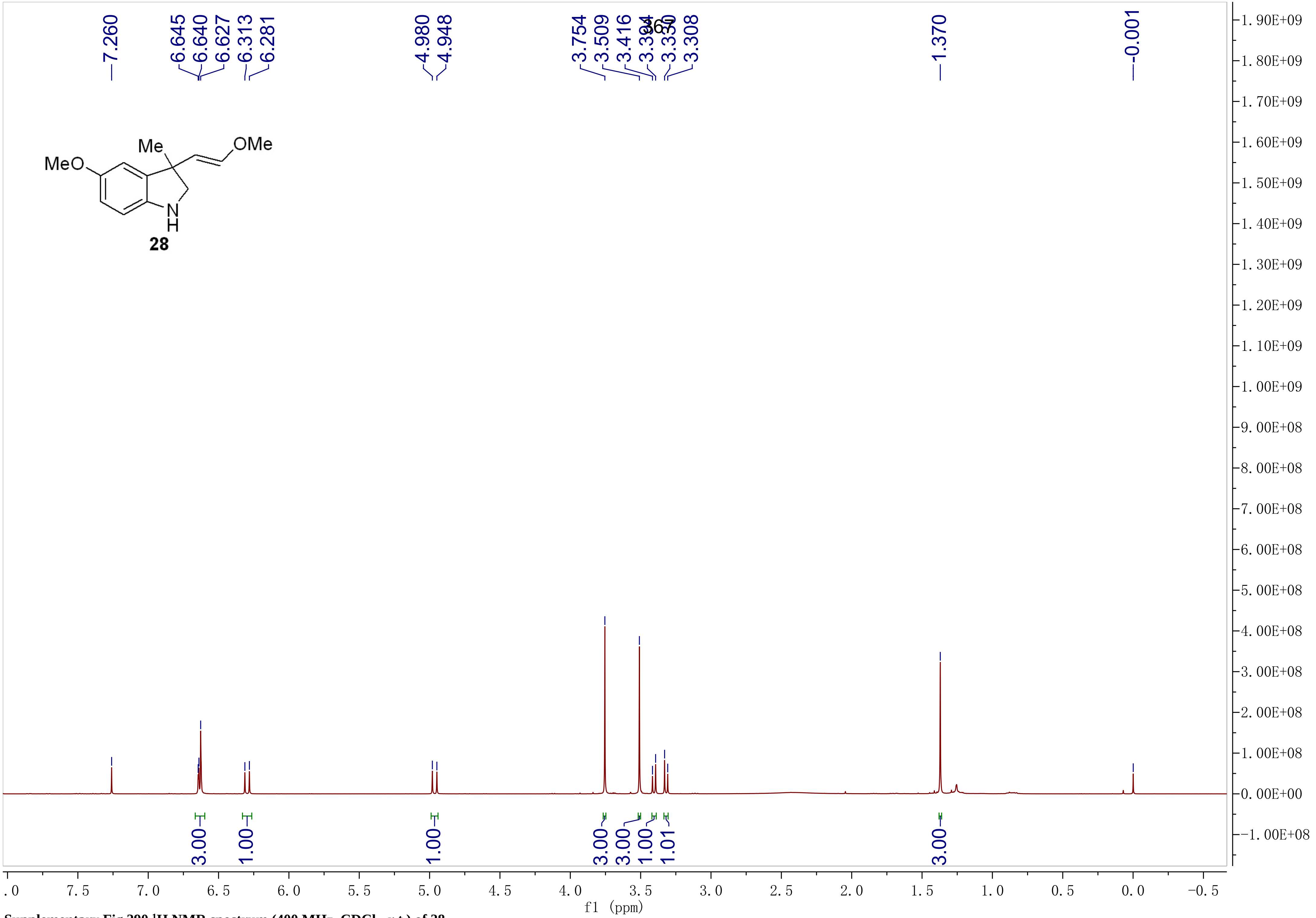

Supplementary Fig 290 ^{1}H NMR spectrum (400 MHz, $CDCl_3$, r.t.) of 28.

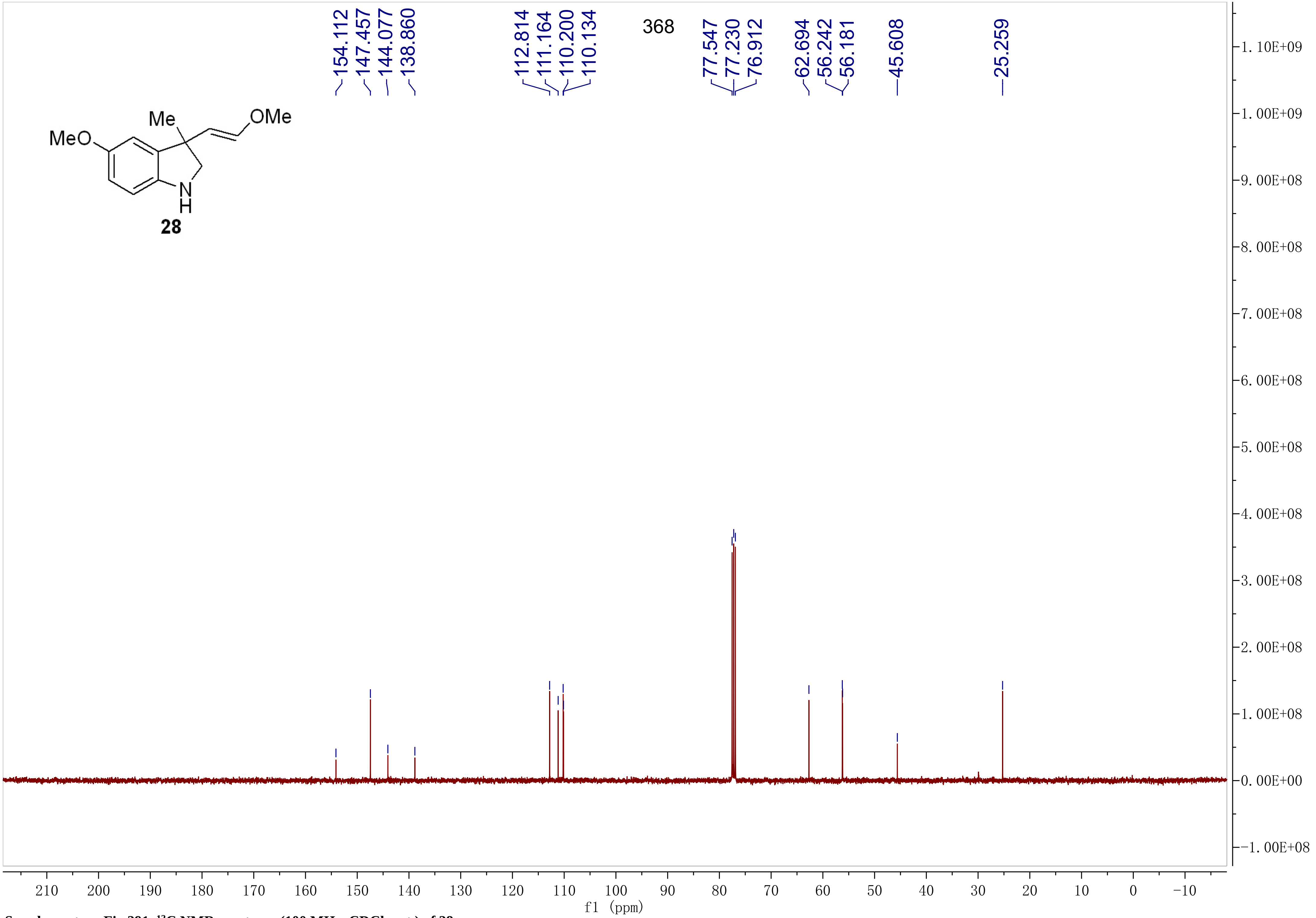

Supplementary Fig 291. ^{13}C NMR spectrum (100 MHz, $CDCl_3$, r.t.) of 28.

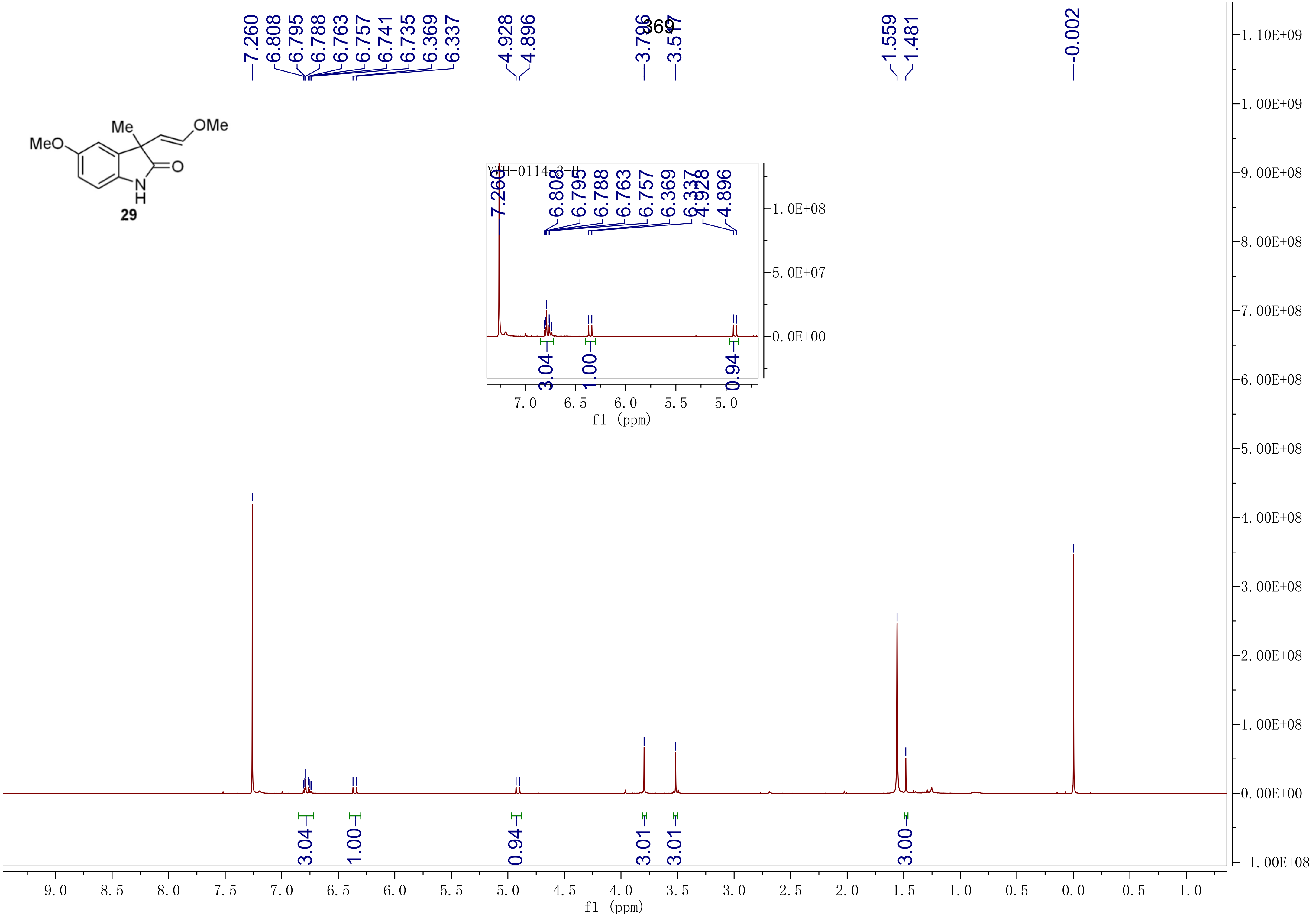

Supplementary Fig 292 ^{1}H NMR spectrum (400 MHz, $CDCl_3$, r.t.) of 29.

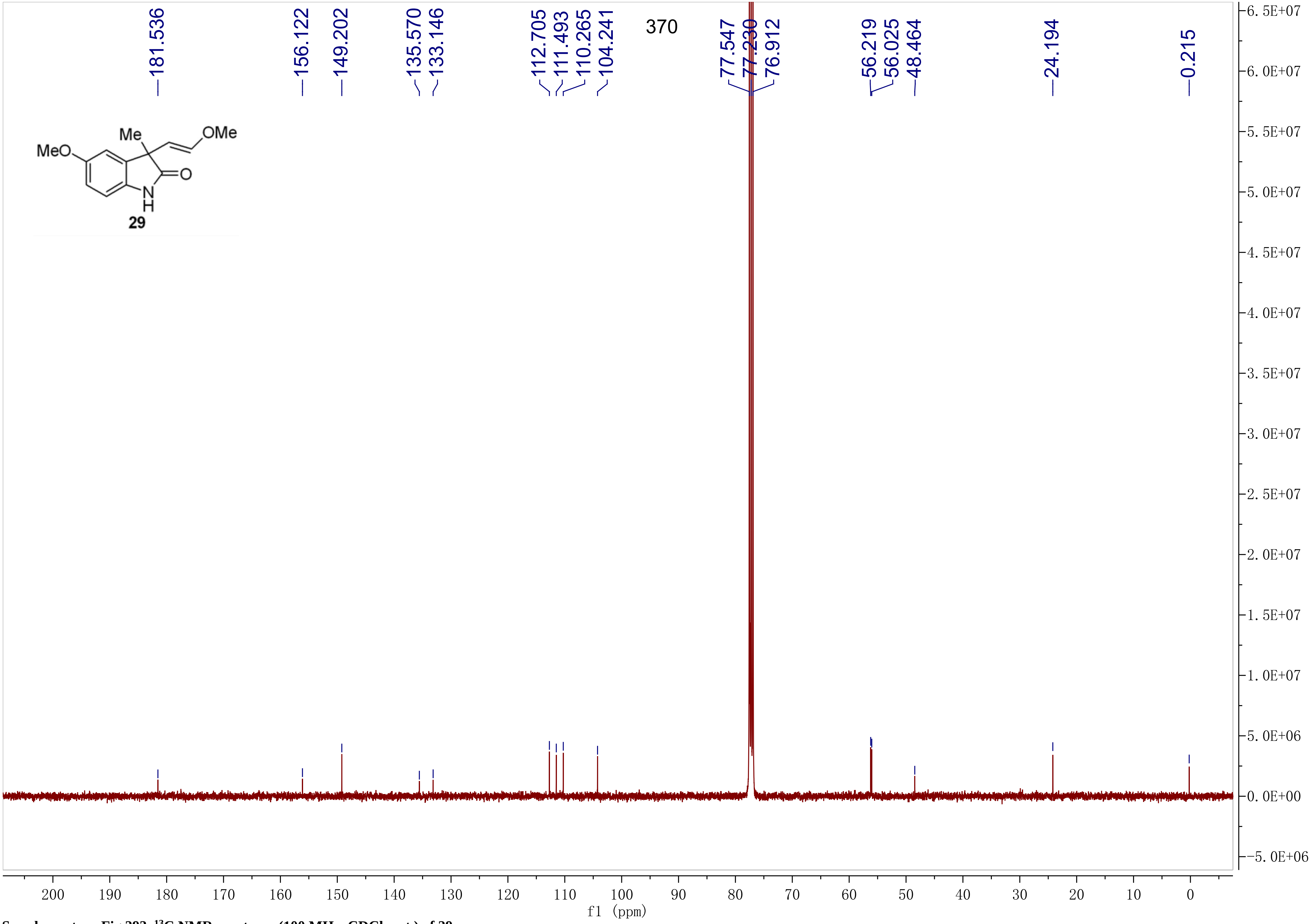

Supplementary Fig 293. ^{13}C NMR spectrum (100 MHz, $CDCl_3$, r.t.) of 29.

4. Supplementary references:

1. Cabrera-Afonso, M. J.; Lu, Z. P.; Kelly, C. B.; Lang, S. B.; Dykstra, R.; Gutierrez, O.; Molander, G. A. *Chem. Sci.* **2018**, *9*, 3186-3191.
2. Madden, K. S.; Laroche, B.; David, S.; Batsanov, A. S.; Thompson, D.; Knowles, J. P.; Whiting, A.; *Eur. J. Org. Chem.* **2018**, *2018*, 5312-5322.
3. Jester, S. S.; Sigmund, E.; Höger, S. *J. Am. Chem. Soc.* **2011**, *133*, 11062-11065.
4. Maddox, S. M.; Dinh, A. N.; Armenta, F.; Um, J.; Gustafson, J. L.; *Org. Lett.* **2016**, *18*, 5476-5479.
5. You, Y.; Kim, H. S.; Park, J. W.; Keum, G.; Jang, S. K.; Kim, B. M. *RSC. Adv.*, **2018**, *8*, 31803-31821.
6. Agejas-Chicharro, F. J.; Dressman, B. A.; Guitierrez, S. S.; Henry, S. S.; Martinez-Perez, J. A.; Massey, S. M.; Monn, J. A.; Zia-Ebrahimi, M. S. WO 2005094822, 2005.
7. Yang, Y.; Lin, Y.; Rao, Y. *Org. Lett.* **2012**, *14*, 2874-2877.
8. Maleczka, R. E.; Shi, F.; Holmes, D.; Smith, M. R. *J. Am. Chem. Soc.* **2003**, *125*, 7792-7793.
9. Šileikytė, J.; Devereaux, J.; Jong, J.; Schiavone, M.; Jones, K.; Nilsen, A.; Bernardi, P.; Forte, M.; Cohen, M. S. *Chem. Med. Chem.* **2019**, *14*, 1771-1782.
10. Jorgensen, W. L.; Bollini, M.; Thakur, V. V.; Domaoal, R. A.; Spasov, K. A.; Anderson, K. S. *J. Am. Chem. Soc.* **2011**, *133*, 15686-15696.
11. Alberico, D.; Rudolph, A.; Lautens, M. *J. Org. Chem.* **2007**, *72*, 775-781.
12. Amemiya, R.; Saito, N.; Yamaguchi, M. *J. Org. Chem.* **2008**, *73*, 7137-7144.
13. Ali, A.; Bohn, J.; Deng, Q.; Lu, Z.; Sinclair, P. J.; Thompson, C. F.; Quraishi, N. WO 2005100298, 2005.
14. Bookse, B. C.; Dang, Q.; Gibson, T. S.; Jiang, H.; Chung, D.; Bao, J.; Jiang, J.; Kassick, A.; Kekec, A.; Lan, P.; Lu, H.; Makara, G. M.; Romero, F. A.; Shbhat, L.; Wilson, D.; Wodka, D. WO 2010047982, 2010.
15. Hickenboth, C. R.; Rule, J. D.; Moore, J. S. *Tetrahedron*. **2008**, *64*, 8435-8448.
16. Maddox, S. M.; Dinh, A. N.; Armenta, F.; Um, J.; Gustafson, J. L. *Org. Lett.* **2016**, *18*, 5476-5479.
17. Suzuki, M.; Toyao, A.; Yokokawa, F. WO 2006069788, 2006.
18. Yu, P.; Morandi, B. *Angew. Chem. Int. Ed.* **2017**, *56*, 15693-15697.
19. Ledoussal, B.; Benarous, R. WO 2012137181, 2012.
20. Jolly, P. I.; Fleary-Roberts, N.; O'Sullivan, S.; Doni, E.; Zhou, S.; Murphy, J. A. *Org. Biomol. Chem.*, **2012**, *10*, 5807-5810.
21. Joseph, J. T.; Sajith, A. M.; Ningegowda, R. C.; Shashikanth, S. *Adv. Synth. Catal.* **2017**, *359*, 419-428.
22. He, Y.; Tang, J.; Luo, M.; Zeng, X.; *Org. Lett.* **2018**, *20*, 4159-4163.
23. Sharma, P.; Schuster-Klein, C. A.; Poitevin, C. WO 2021253095, 2021.
24. Kotrabasaiah, U. R.; Hosahalli, S.; Bejugam, M.; WO 2015101928, 2015.
25. Koren, A. O.; Horti, A. G.; Mukhin, A. G.; Gündisch, D.; Kimes, A. S.; Dannals, R. F.; London, E. D. *J. Med. Chem.* **1998**, *41*, 3690.
26. Zhang, K.; Christoffel, F.; Baudoin, O. *Angew. Chem. Int. Ed.* **2018**, *57*,

1982-1986.
27. Si, T.; Li, B.; Xiong, W.; Xu, B.; Tang, W. *Org. Biomol. Chem.*, **2017**, *15*, 9903-9909.
28. Chen, W.; Huang, Z.; Tay, N. E. S.; Giglio, B.; Wang, M.; Wang, H.; Wu, Z.; Nicewicz, D. A.; Li, Z. *Science*, **2019**, *364*, 1170-1174.
29. Zhang, Y.; Xiong, W.; Cen, J.; Wang, L.; Cheng, R.; Qi, C.; Wu, W.; Jiang, H. *Org. Lett.* **2019**, *21*, 345-349.
30. Shi, J.; Qiu, D.; Wang, J.; Xu, H.; Li, Y. *J. Am. Chem. Soc.* **2015**, *137*, 5670−5673.
31. Qiu, D.; He, J.; Yue, X.; Shi, J.; Li, Y. *Org. Lett.* **2016**, *18*, 3130-3133.
32. Li, L.; Qiu, D.; Shi, J.; Li, Y. *Org. Lett.* **2016**, *18*, 3726-3729.
33. Xu, H.; He, J.; Shi, J.; Tan, L.; Qiu, D.; Luo, X.; Li, Y. *J. Am. Chem. Soc.* **2018**, *140*, 3555-3559.
34. He, J.; Jia, Z.; Tan, H.; Luo, X.; Qiu, D.; Shi, J.; Xu, H.; Li, Y. *Angew. Chem. Int. Ed.* **2019**, *58*, 18513-18518.
35. Ueda, S.; Nagasawa, H. *Angew. Chem. Int. Ed.* **2008**, *47*, 6411-6413.
36. McManus. J. B.; Nicewicz, D. A. *J. Am. Chem. Soc.* **2017**, *139*, 2880-2883.
37. Wang, T.; Yao, W.; Zhong, F.; Pang, G. H.; Lu, Y. *Angew. Chem. Int. Ed.* **2014**, *53*, 2964-2968.
